# Residues of Fluoroquinolone Antibiotics Induce Carbonylation and Reduce In Vitro Digestion of Sarcoplasmic and Myofibrillar Beef Proteins

**DOI:** 10.3390/foods9020170

**Published:** 2020-02-11

**Authors:** Johana Márquez-Lázaro, Darío Méndez-Cuadro, Erika Rodríguez-Cavallo

**Affiliations:** Analytical Chemistry and Biomedicine Group, University of Cartagena, Cartagena de Indias 130001, Colombia; jmarquezl1@unicartagena.edu.co

**Keywords:** fluoroquinolones, MRL, protein carbonylation, beef, solubility, digestibility, sarcoplasmic and myofibrillar proteins

## Abstract

Although the impact of oxidation on human health has been of growing interest, the oxidation of proteins, major component of meat, has received little attention. This paper describes the in vitro effect of five fluoroquinolones (FQs) on carbonylation of sarcoplasmic and myofibrillar proteins of beef when found at concentrations close to the maximum residue limit (MRL). Samples were treated individually with the FQs, determining in each protein fraction the carbonyl index, protein content and oxidized proteins identification, using 2,4-dinitrophenyhydrazine (DNPH) alkaline assay, Western blot and Bradford methods, and mass spectrometry, respectively. Besides, the in vitro effect of these residues on gastric and duodenal digestion of proteins was evaluated. The carbonylation induced by FQs affected both protein fractions being significant with respect to the blank in 73.3% of cases. This damage was correlated with loss of solubility and digestibility, with sarcoplasmic proteins the most affected. Danofloxacin and enrofloxacin were the FQs with greatest oxidant effects, especially affecting glycolysis and glycogen proteins. Our results suggest that these residues induce irreversible oxidative damage on the main beef proteins and could affect their nutritional value.

## 1. Introduction

Beef production is generally derived from intensive animal farming, characterized by confinement of the animal during most of its life [1]. Under these conditions they are highly susceptible to diseases transmitted by microorganisms that can spread rapidly and cause important production losses. Thus, animals are routinely treated with antibiotics to prevent, treat or control disease [2] and in some countries such as USA and other developing countries, these are authorized as growth promoters [3]. Up to 80% of the important antibiotics in human medicine are used as growth promoters, generating high concern about the loss of therapeutic efficacy as a result of their use in animals. Fluoroquinolones (FQs) such as enrofloxacin (ENRO), danofloxacin (DANO) and difloxacin are antibiotics widely administered in cattle farms to treat respiratory and gastrointestinal diseases, mastitis and skin and urinary infections or growth promoters [4]. However, direct and indirect exposure of animals to FQs may promote its bioaccumulation in edible products such as meat [3]. The consumption of contaminated food is associated to allergic reactions, anaphylactic shock and alteration of the immune system, but bacterial resistance is the greatest concern with regard to the use of FQs in animal husbandry [5,6]. Moreover, FQs for exclusive use in humans as norfloxacin (NOR) and ciprofloxacin (CIPRO) have been detected in animal tissues as well as sarafloxacin (SARA) that is used in poultry [7,8,9,10,11]. The problem of FQs residues in edible products is linked to ineffective government policies and poor implementation of good agricultural practices in developing countries, which will be responsible for 76% of the output growth expected in 2027. Fact that will facilitates the appearance of these residues in beef, even at unsuspected concentrations [12].

To ensure consumer safety, international regulatory bodies such as the Food and Drug Administration (FDA), European Parliament and Codex Alimentarius have established the maximum residue levels (MRLs) by type of substance, tissue and animal species [6,13]. The MRL is defined as the maximum concentration of residue legally tolerated in a food product and that does not have a toxic effect on the consumer´s health. However, little is known about the impact of these concentrations on the digestibility and nutritional value of the beef. In fact, the determination of the MRL focuses mainly on the toxicological effect in humans, without knowing its impact on products of animal origin [6]. This inquiry is based on two aspects: the susceptibility of muscle proteins to oxidation reactions, and the demonstrated ability of FQs to induce oxidative stress in experimental models of mice, plants, fish and chickens [14,15,16,17]. Therefore, it is expected that, under the conditions of use of these substances in livestock, muscle proteins are subject to significant oxidative stress induced by the presence of FQs residues in muscle cells, even at MRL values or close to these. Recent research has demonstrated that the health of the consumer can be affected for the intake of oxidized proteins, by promoting inflammatory conditions in the intestine, as well as carcinogenic processes [18].

**Figure 1:**
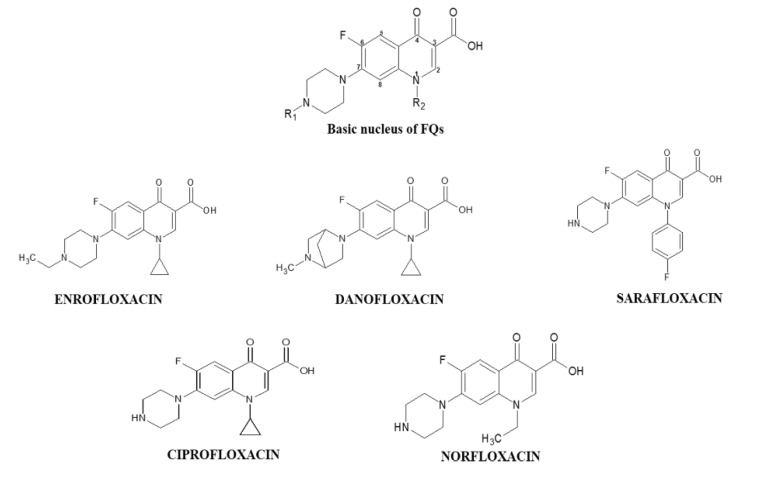
Chemical structure and basic nucleus of the fluoroquinolones (FQs) under study.

Protein oxidation in food is widely monitored through carbonylation, which is an irreversible modification on lateral chains of amino acid residues in the peptide chain. Loss of solubility, protein aggregation, fragmentation and decreased proteases susceptibility can be affected by the carbonylation, producing a negative impact on the technological, sensory, functional and nutritional properties of beef [19]. However, evaluating oxidative effect mediated by FQs under in vivo conditions is expensive and creates ethical conflicts. Hence, in vitro approaches as the reported by Wang et al., become a good alternative to study the impact of oxidative stress products in meat proteins [20]. Until now, the carbonylation of proteins of animal origin has been associated with factors such as the feeding, slaughtering, processing and storage of their products [19]. Therefore, the objective of this study was to quantify the in vitro carbonylation induced by residues of DANO, ENRO, CIPRO, NOR and SARA (Figure 1) at equivalent concentrations and close to the MRL on the sarcoplasmic and myofibrillar proteins of beef and evaluate their effects on techno-functional properties such as solubility and protein digestibility.

## 2. Materials and Methods

### 2.1. Reagents and Materials

The following reagents were of analytical standard grade and Sigma-Aldrich (Saint Louis, MO, USA) supplied: danofloxacin, enrofloxacin, ciprofloxacin, norfloxacin, sarafloxacin, bovine serum albumin (BSA), pepsin, α-chymotrypsin and trypsin. SDS (Pepyn, France) supplied methanol (MeOH) and acetonitrile (MeCN) (HPLC grade). PanReac^®^ (Barcelona, Spain) supplied 2,4-Dinitrophenylhydrazine, potassium phosphate, sodium azide, hydrochloric acid, sodium bicarbonate and calcium chloride. Sodium hydroxide, sodium chloride, potassium chloride, 2-mercaptoethanol, Coomassie blue-brillant G250 and trichloroacetic acid (TCA) (99.5%) were purchased from Merck^®^ (Darmstadt, Germany). Proteomic or molecular biology grade reagents: sodium dodecyl sulfate, Tris Base and Glycerol were supplied from Amresco^®^ (West Chester, PA, USA). Orthophosphoric acid, molecular weight marker Spectra Multicolor Broad Range, Rabbit anti-DNP monoclonal and goat anti-Rabbit HRP antibodies were purchased from Scharlab (Barcelona, Spain), Thermo Scientific (Waltham, MA, USA), Invitrogen (Carlsbad, MA, USA) and Bioss Inc. (Woburn, MA, USA), respectively. Water was purified with a Milli-Q system (Millipore, Bedford, MA, USA).

### 2.2. Experimental Design

#### 2.2.1. Meat Samples Obtention

*Longissimus thoracis* samples from beef were acquired in Arjona, Bolívar (Colombia). The samples were obtained from animals free of FQs administration during the three months prior to slaughter. Plastic polyethylene bags were used for transport, maintaining a cold chain (4 °C) to inactivate proteases. To ensure contact with FQs, beef samples free of fat and visible connective tissue, were mechanically homogenized (Powergen by Fisher) [21] and one-gram aliquots were weighed in falcon tubes and stored at −20 °C. The samples remained viable for two weeks.

#### 2.2.2. Fluoroquinolones Working Solutions

Stock solutions of DANO, ENRO, SARA, CIPRO and NOR at 100 µg·mL^−1^ were prepared using a mixture MeOH/TCA (0.1% *v*/*v*) and stored at 4 °C in the dark for up to one month. To avoid precipitation of the proteins in the samples by solvent effect, serial dilutions were prepared in water (working solutions), that were used in the obtention of each level of contamination (0.5, 1 and 1.5 times the MRL), in the treated samples.

#### 2.2.3. Preparation of Fluoroquinolones (FQs) Treated Samples

Beef aliquots (one gram) were randomly distributed to obtain blank samples and five groups of treated samples (one for each FQ). In each group, triplicates were prepared per level of contamination to be tested (0.5, 1 and 1.5 times the MRL values), using for this 200 µL of the corresponding FQ working solution. Thus, a total of 30 assays were performed to evaluate the oxidative effect of each fluoroquinolone on sarcoplasmic and myofibrillar proteins and its consequence on solubility and the in vitro digestion. The blank samples were prepared by addition of 200 µL of water (0.125% of MeOH), which was final concentration of the vehicle in FQs working solutions.

The MRL values considered were those established by Commission Regulation (EU) No.37/2010 (European Commission 2010) for bovine muscle (ENRO and CIPRO: 100 µg·Kg^−1^; DANO 200 µg·Kg^−1^). Since SARA and NOR are not regulated, it was decided to evaluate them at 100 µg·Kg^−1^, considering the level established for chicken muscle and the lowest concentration tested in this study, respectively. Both, the treated and blank samples were vortexed for 10 s (3000 rpm) and incubated in the dark at 25 °C for one hour [22]. Then, the samples were kept at 4 °C, protected from light, until the proteins of interest were extracted.

### 2.3. Protein Extraction and Obtaining Electrophoretic Profiles (Sodium Dodecyl Sulfate–Polyacrylamide Gel Electrophoresis, SDS-PAGE)

In the extraction of sarcoplasmic (SP) and myofibrillar (MP) proteins, the procedure described by Molette et al. [23] was followed with some modifications. Briefly, each type of sample was mixed with 10 mL of low ionic strength buffer (0.05 M K_3_PO_4_, 1 mM NaN_3_, 2 mM EDTA (Ethylenediaminetetraacetic acid), pH 7.3), applying vortex at 3000 rpm for 3.5 min, followed by centrifugation (11,150× *g*, 10 min, 1 °C). The supernatant, containing SPs, was collected and kept refrigerated. The pellet was resuspended with 5 mL of low ionic strength buffer, centrifuged and the obtained supernatant was combined with the SP fraction obtained above. The resulting pellet was resuspended with 1.5 mL of high ionic strength buffer (0.55 M KCl, 0.05 M K_3_PO_4_, 1mM NaN_3_, 2 mM EDTA, pH 7.3), applying a vortex at 3000 rpm for 3.5 min and centrifuging according to the conditions described above. The supernatant, containing MPs, was collected and kept refrigerated.

To determine the yielding, protein concentration was determined by the Bradford method, using BSA as a standard [24] and expressing the results as mg of protein·g^−1^ of processed meat. The yield and precision of the extraction method was evaluated, in terms of repeatability and intra-laboratory reproducibility, comparing the yields obtained from the analysis of blank samples (*n* = 5) and treated samples.

In order to verify the integrity of the protein fractions obtained, their protein profiles (of each group) in denaturing electrophoresis (10% sodium dodecyl sulfate–polyacrylamide gel electrophoresis, SDS-PAGE) were developed and compared against to corresponding blank. For this, the samples were mixed with loading buffer (SDS 2%, glycerol 10%, Tris-HCl, pH 6.8, bromophenol blue 0.1% and β-mercaptoethanol 3%), and heated at 100 °C for 3 min. 5 μg of protein was loaded on each gel line, placing the molecular weight marker on the first line. The gels were run at 120 V for 2 h, using a Mini-PROTEAN Tetra Cell camera system (Bio-Rad, Hercules, CA, USA), then stained with Comassie blue brilliant (CBB) G250 and the electrophoregrams were digitized in a ChemiDoc™ MP system (Bio-Rad, Hercules, CA, USA).

### 2.4. Determination of Carbonyl Index

Protein carbonyls were measured by estimating total carbonyl groups, according to Mesquita et al. [25] and incorporating some modifications. Briefly, 300 µL of 2,4-dinitrophenyhydrazine (DNPH, 10 mM in 0.5M H_3_PO_4_) was added to 300 µL of protein solution (150 µg), followed by incubation for 10 min in dark (25 °C). Then, 160 µL of this solution was placed in 96-well plates and 40 µL of 6 M NaOH were added. The mixture was incubated again for 10 min in the dark (25 °C) The absorbance of mixture was read at 450 nm, using a FLUOstar® Omega spectrophotometer (BMG-Lab Tech, Ortenberg, Germany). The carbonyl content was calculated using the DNPH molar extinction coefficient, corrected for microplates (ε = 11,154 μM^−1^·cm^−1^). The results were expressed as nmol of carbonyls·mg^−1^ of protein. Given the instability of dinitrophenylhydrazones in alkaline medium, the incubation time after adding the NaOH solution, was rigorously controlled.

### 2.5. Western Blot

The oxidative profile of sarcoplasmic and myofibrillar proteins was developed according the maximum carbonylation promoted by FQs. For this, the protein carbonyls of blank and treated samples were labeled with DNPH (10 mM in 2N HCl) and gel separated (10% SDS-PAGE). Then, 10 µg of SPs and 15 µg of MPs were loaded per line and molecular weight marker was loaded on the first line of each gel. The gels were run at 130 V for 2 h, and subsequently, the proteins were transferred to PVDF membranes by immuno-blot, using a semidry Blotting System Trans blot turbo (Biorad, Hercules, CA, USA) in standard mode. The membranes were blocked in 5% skim milk-phosphate buffered saline (PBS) for 2 h at room temperature and subsequent incubation with anti-DNP antibody (1:4000) for 2 h. Then, the membranes were washed twice with 5% skim milk-PBS for 5 min and incubated for 1 h with goat anti-rabbit-conjugated horseradish peroxidase-antibody (1:5000). Finally, the membranes were washed twice for five minutes, with 0.05% Tween 20 in 5% skim milk-PBS, 5% skim milk-PBS and PBS. The oxidized proteins were visualized and analyzed, using a ChemiDoc system and an Image Lab Software (Bio Rad, Hercules, CA, USA), respectively. Concomitantly with the Western blot assays, gels (10% SDS-PAGE) were run using proteins not derivatized from the same samples. Gels were fixed, stained with CBB and digitalized in the ChemiDoc system. Treated samples were loaded by duplicate and blank samples by triplicate.

Pairs of stained gels and oxyblots from sarcoplasmic and myofibrillar proteins were matched to select the carbonylated protein bands send to mass spectrometry assays. This procedure was carried out with images obtained in a ChemiDoc system (Bio Rad, Hercules, CA, USA). Subsequently, the selected bands were cut from SDS-PAGE gels and send to tandem mass spectrometry assays.

### 2.6. Identification of Proteins in Carbonylated Bands

The bands selected of gels were reduced, alkylated and digested with trypsin according to Sechi et al. [26]. For this, bands were reduced with dithioerytritol 10 mM in NH_4_HCO_3_ 25 mM for 30 min at 56 °C and subsequently alkylated with iodoacetamide 55 mM in NH_4_HCO_3_ 25 mM for 15 min in dark. Finally, these were digested with 12.5 ng·µL^−1^ sequencing grade bovine trypsin in NH_4_HCO_3_ 25 mM (pH 8.5) overnight at 37 °C. After digestion, the supernatant was collected and 1 µL was spotted onto a MALDI (Matrix-Assisted Laser Desorption/Ionization) target plate and allowed to air-dry at room temperature. Then, 0.8 µL of a solution 3 mg·mL^−1^ of α-cyano-4-hydroxy-cinnamic acid matrix in MeCN 50%, trifluoroacetic acid (TFA) 0.1% were added to the dried peptide and allowed again to air-dry at room temperature. MALDI-TOF MS (Matrix-Assisted Laser Desorption/Ionization -Time-Of-Flight- mass spectrometry) analyses were performed in a 4800 Plus Proteomics Analyzer MALDI-TOF/TOF mass spectrometer (Applied Biosystems, Toronto, Canada) at the Proteomics Unit of Complutense University of Madrid. MALDI-TOF/TOF was operated in positive reflector mode with an accelerating voltage of 20,000 V. All mass spectra were calibrated by default or internally using peptides from the auto digestion of trypsin when were appeared.

For protein identification searches for peptide mass fingerprints, tandem MS spectra and both combined were performed in the NCBInr database 20120508 (17919084 sequences; 6150218869: 18–23 July 2018), residues without taxonomy restriction, using MASCOT2.3 through the software Global Protein Server v3.6 (ABSciex, Washington, DC, USA). Search parameters were carbamidomethyl cystein as fixed modification and oxidized methionine as variable modification, Peptide mass tolerance, 50–100 ppm and one missed trypsin cleavage site. In all protein identification, the protein scores were greater than the score fixed by mascot as significant with a *p* < 0.05.

### 2.7. In Vitro Digestibility Test

SPs and MPs extracted from blank samples and treated with DANO and ENRO at the MRL level were carried out to in vitro digestion assay according reported by Hu et al. [27]. Briefly, for the gastric phase, individual solutions of SPs and MPs at 1.4 µg·µL^−1^ (1.1 mL of final volume) were acidified (pH 3.0) with 0.01 N HCl. Then, pepsin enzyme was added to achieve a concentration of 0.0027 U·mL^−1^ in the final mixture. Gastric digestion was performed during 30 min at 37 °C in a bath with constant stirring. Then, it was stopped by adjusting to pH 7.0 with 200 mM NaHCO_3_/50 mM CaCl_2_ and aliquots of the gastric phase were taken to examine it by SDS-PAGE. Before that, the mixture (gastric phase) was subjected to the intestinal digestion by adding 0.50 U·mL^−1^ of trypsin and 0.25 U·mL^−1^ of α-chymotrypsin and incubating at 37 °C (bath) for 30 min. This phase was stopped by heating at 80 °C for 5 min and aliquots were taken for its examination. The digestion of SP and MP from treated and blank samples were realized by duplicate.

At the end of digestion assay, aliquots (10 µL) of gastric and intestinal phase were individually loaded on 15% SDS-PAGE gels. Additionally, SP and MP from blank samples (10 µL) were loaded in these gels. The gels were run according protein type and jointly incorporating the gastric and intestinal phases. The gels were stained with CBB and digitalized in the ChemiDoc System. Then, a densitometry analysis of bands was performed. The percentage of digestibility and restriction of digestibility for selected bands were calculated based on Equations (1) and (2), respectively:

(1) $$\%\;Digestibility=\frac{Digested\;band\;intensity}{No\;digested\;band\;intensity}\times 100$$

(2) % Restriction digestibility=100−% digestion

No digested band intensity refers to bands from SP or MP of blank sample, which were not subjected to digestion.

### 2.8. Statistical Analysis

All values were reported as mean ± standard error of the mean (SEM) of three independent determinations. To assess the effect of FQ concentration on protein carbonylation, a unifactorial analysis of variance (ANOVA) with three levels and blank at 95% confidence was performed (GraphPad Prism V5.01, San Diego, CA, USA). Multiple comparisons of the means were made using the Tukey adjustment, when the ANOVA was significant (*p* < 0.05). Logarithmic regression (y = lna + b × lnx) and simple linear regression (y = mx + b) models were used to evaluate the effect of carbonylation (fixed effect) on the loss of solubility of sarcoplasmic and myofibrillar proteins, respectively (IBM-SPSS v219). In both, the carbonylation was independent variable and the loss of solubility, variable dependent.

## 3. Results and Discussion

### 3.1. Quantitation of Protein Carbonylation Induced by FQs Antibiotics

**Figure 2:**
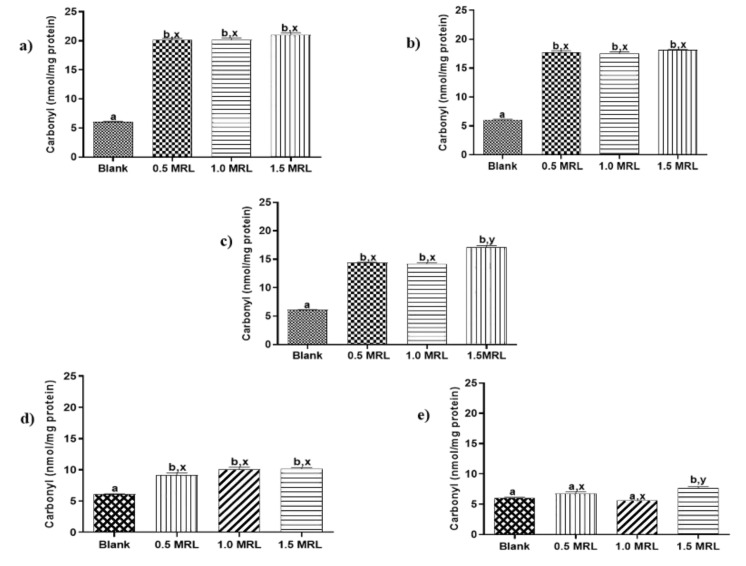
Changes in carbonyl content of sarcoplasmic proteins induced by (**a**) enrofloxacin (ENRO), (**b**) danofloxacin (DANO), (**c**) sarafloxacin (SARA), (**d**) ciprofloxacin (CIPRO) and (**e**) norfloxacin (NOR). Mean values ± standard error of the mean (SEM). Superscripts a, b and x, y show significant statistical differences between means of groups (*p* < 0.05): a, b show differences with respect to blank, while x, y differences between FQ concentrations.

**Figure 3:**
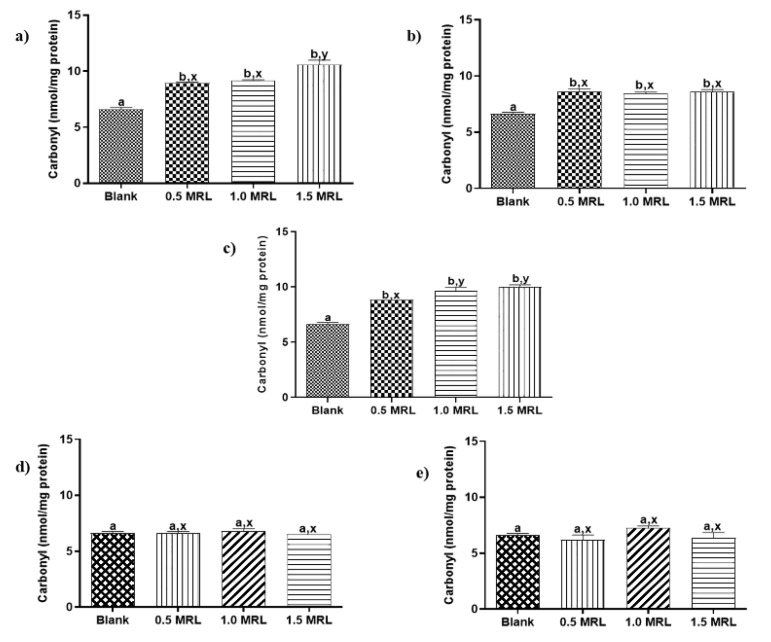
Changes in carbonyl content of myofibrillar proteins induced by (**a**) enrofloxacin (**b**) danofloxacin, (**c**) sarafloxacin, (**d**) ciprofloxacin y, (**e**) norfloxacin. Mean values ± SEM. Superscripts a, b and x, y show significant statistical differences between means of groups (*p < 0.05*): a, b show differences with respect to blank, while x, y differences between FQ concentrations.

Protein carbonylation in animal foods is recognized as one of the most remarkable chemical modifications in oxidized proteins and frequently it is attribute to aspects such as slaughter, storage or processing [19]. To the best of our knowledge, protein carbonylation induced by FQs used in veterinary medicine has not described yet. In order to determine oxidant effects induced by these drugs, beef samples were treated individually with five FQs antibiotics at 0.5, 1.0 and 1.5 MRL concentrations. Then, sarcoplasmic and myofibrillar proteins were extracted and quantified as previous step to measuring its carbonyl indexes. The results show that FQs under study induced in vitro carbonylation of SPs and MPs (Figure 2 and Figure 3), an effect that was observed in 22 of 30 assays carried out (73.3%).

The carbonylation induced in sarcoplasmic proteins from treated samples was significantly higher than those obtained in blank samples, even at the lowest treatment levels (*p* < 0.05). An exception was NOR at levels 0.5 and 1.0 MRL (Figure 2). The grade of carbonylation observed was not to significantly different with respect to concentrations assayed for ENRO, DANO and CIPRO. By contrast, the oxidation promoted by concentration 1.5 MRL in SARA was significantly higher than other levels of this FQ. In case of myofibrillar proteins, only ENRO, DANO and SARA significantly increased carbonylation with respect to blank samples (*p* < 0.05) (Figure 3). The grade of carbonylation induced with respect to FQ concentration assayed was similar for DANO (*p* > 0.05), but different for ENRO and SARA. Thus, the oxidation promoted by levels 1.5 MRL of ENRO and 0.5 MRL of SARA were significantly different with respect the other concentrations in each one.

According to the results described above, SPs were more susceptible than MPs to the effect of FQs; highlighting DANO, ENRO and SARA as those with the most oxidizing power. The mechanism of protein carbonylation followed by FQs has been proposed by some authors is related to the structural similarity of these compounds with the quinones of the electron transport chain (ETC) in mitochondria. This hypothesis suggests that FQs can act as inhibitors of the binding site of the transporting quinones in the ETC complex II, as observed in plants [15,28]. The disruption of ETC could increase oxidative stress, through the generation of free radicals (O^−2^, H_2_O_2_), which could oxidize proteins by direct attack.

### 3.2. Yielding of Protein Fractions is Affected by Protein Oxidation Induced by Fluoroquinolones

Different authors have related protein carbonylation in foods with alteration of their techno-functional properties, including loss of solubility and digestibility [19]. Therefore, after demonstrating the oxidative damage induced by FQs, their impact on solubility was evaluated in terms of yield obtained for each protein fraction. For this purpose, blank samples yielding were employed as reference values.

Thus, yields calculated for SPs and MPs in blank samples were 58.7 (Relative Standard Deviation RSDs < 4%) and 2.6 mg (RSDs < 5.9%) of protein·gram^−1^ of sample, respectively (Table 1). The evaluation of intra-laboratory reproducibility showed values of 59.1 and 2.7 mg with RSDs < 8.7% and 6.1%, respectively. These results evidenced the good repeatability and reproducibility of the extraction method used.

When comparing these results with those obtained from treated samples (Table 1), it was found that SARA, DANO and ENRO decreased the yield in both types of proteins (*p* < 0.05) at all concentrations assayed, into a values range between 66.3% and 75.6% of the amount obtained in blank samples. Instead, the treatments with CIPRO and NOR did not affect these yields, except the 1.5 MRL level of CIPRO.

From yield data, loss of solubility was calculated which resulted more marked in SPs reaching values between 27.6 and 34.6% with SD not greater than 1.3 and showing a slight concentration-effect relationship in those samples exposed to SARA and ENRO; whereas, in MPs the concentration-effect relationship only was observed in samples exposed to ENRO, whose loss of solubility were between 5 and 16.5% (SD < 0.54). In summary, residues of SARA, ENRO and DANO close to MRL induce high losses of solubility in the studied proteins, this effect being more marked in SPs.

**Figure 4:**
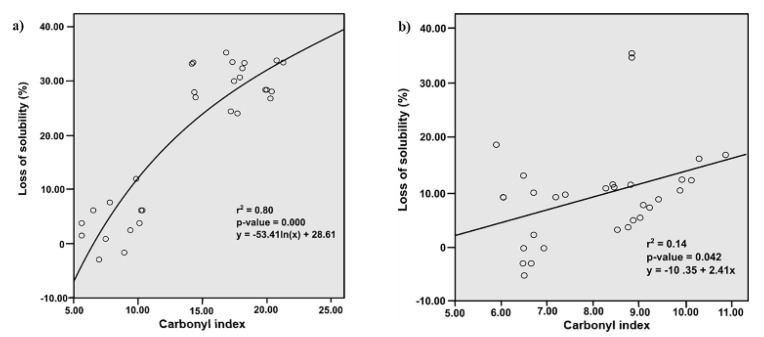
Effect of carbonylation on loss of solubility. It was evaluated through (**a**) logarithmic regression in sarcoplasmic proteins and (**b**) simple linear regression in myofibrillar proteins. The model was significant to *p* < 0.05.

Usually the loss of solubility is associated with carbonylation, thus, in order to identify this type of relationship, regression analysis was performed (Figure 4). The r^2^ value of the logarithmic regression and simple linear regression models indicated that the loss of solubility and carbonylation induced by FQs on SPs and MPs, have a positive correlation. The difference observed in the solubility behavior suggests that protein location in the muscle cell and the polarity of FQs are determining aspects in this effect. Since the SPs are located in the sarcoplasm (soluble fraction of myocyte), they could be more accessible to the ROS promoted by those FQs with better diffusion across the sarcolemma. Then, considering the order of increasing polarity of these substances (NOR < CIPRO < DANO < ENRO < SARA) [29], the FQs such as NOR and CIPRO would have more difficulty permeating the sarcoplasm, while SARA, ENRO and DANO would permeate it more easily, explaining their high impact on the solubility of these proteins [28,30,31,32,33,34,35,36,37].

### 3.3. Identification of Carbonylated Proteins

In order to identify proteins potentially carbonylated, electrophoretic profiles and oxyblots obtained in the Western blot assays were used to select oxidized protein bands from each fraction.

Initially, electrophoretic profiles of SPs and MPs from blank and treated samples were obtained to verify the profile similarity among them and respect to describe in literature (Figure S1). First, no changes in protein profiles of interest in any of the samples tested were observed, being, moreover, similar to those reported in the literature [33,34,35].

**Figure 5:**
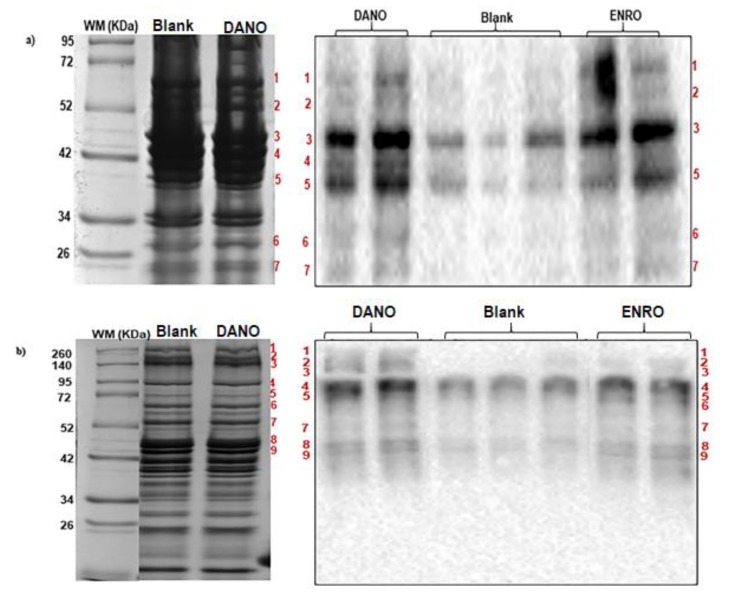
Carbonylation patterns (Western blot) and sodium dodecyl sulfate–polyacrylamide gel electrophoresis (SDS-PAGE) of beef proteins. 1D oxyblots (right) from (**a**) sarcoplasmic and (**b**) myofibrillar proteins were matched against SDS-PAGE (left) to identify carbonylated protein bands from blank and treated samples with danofloxacin and enrofloxacin at MRL contamination level (200 and 100 µg·Kg^−1^, respectively). Matched bands were excised from SDS-PAGE, digested and analyzed by mass spectrometry. Due protein profiles resulted similar in all cases by each protein fraction, only two lanes, with blank and DANO treated samples, are showed in the SDS-PAGE. In Western blot, treated and blank samples were loaded by duplicate and triplicate, respectively.

The next was to get the SPs and MPs carbonylation profile from blank samples and treated samples with DANO and ENRO at 1.0 MRL (Figure 5). Although protein carbonylated similar patterns were observed in all samples, the intensity of chemiluminescent signal were always greater in the carbonylated bands from treated samples.

Electrophoretic profiles (SDS-PAGE) and oxyblots (Western blot) were matched to select carbonylated bands in treated samples, using weight marker as reference. Matched bands were excised from SDS-PAGE, digested and analyzed by mass spectrometry. The identified proteins are listed in Table 2 and Table 3.

In sarcoplasmic fraction the most proteins identified belong to glycolysis pathway [38] (Table 2). The fructose bisphosphate aldolase was identified in bands 2, 3 and 5, showing similar behavior to that described by Shi et al. [39], which in our case could be related to the amount of carbonyl groups incorporated in this protein. In the bands 1, 6 and 7 were identified piruvate kinase, phosphoglycerate-mutase and triosaphosphate-isomerase, respectively [38,40]. The band 4, only was carbonylated by DANO, a fact that is important since creatine kinase M has an important role as an antioxidant in anaerobic metabolism after slaughter [41].

In the same way proteins of the glycolysis pathway were identified from myofibrillar fraction (Table 3), [38,42,43]. This event could be explained by the translocation phenomenon, which occurs mainly as a consequence of the denaturation of both soluble and structural proteins during the post mortem phase [44,45]. In bands 3 and 7 were identified myosin C binding protein and antiparallel actin dimer, respectively. These proteins participate in the organization and stabilization of thick filaments, as well as in the formation of cross bridges between myosin and actin [46].

As described above, the in vitro treatment of beef with fluoroquinolones induced carbonylation in proteins implicated in glycolysis pathway. This fact is interesting because under in vivo conditions, DANO or ENRO residues could affect the ATP production in muscle cells.

### 3.4. Effects of Carbonylation on the In Vitro Digestibility of Proteins

**Figure 6:**
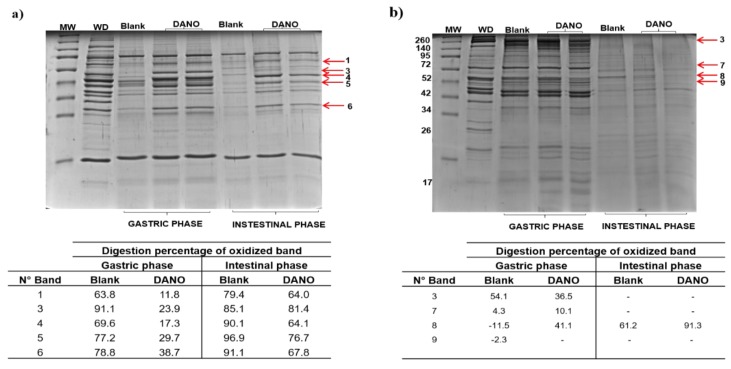
In vitro gastrointestinal digestion of beef proteins treated with danofloxacin at 1.0 maximum residue limit (MRL). (**a**) sarcoplasmic and (**b**) myofibrillar proteins extracted from blank and treated samples subjected to gastric and intestinal digestion. The digestion electrophoretic profiles were realized in 15% SDS-PAGE. Arrows indicate oxidized bands selected for the follow-up of their digestion. Tables show the digestion percentage of bands both blank and exposed to danofloxacin in gastric and intestinal phases. MW: molecular marker; WD: protein not digested.

**Figure 7:**
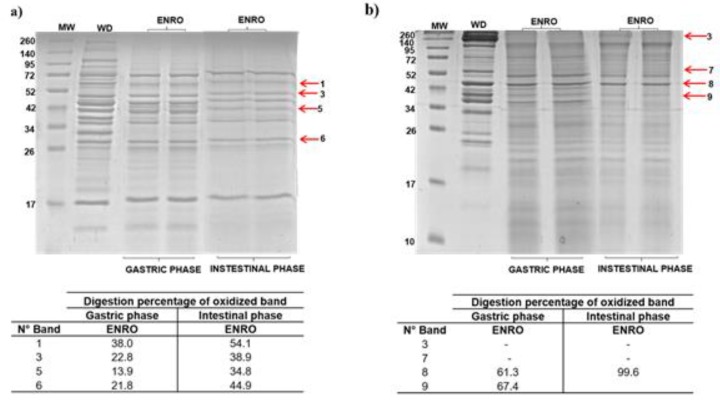
In vitro gastrointestinal digestion of beef proteins treated with enrofloxacin at 1.0 MRL. (**a**) sarcoplasmic and (**b**) myofibrillar proteins extracted treated samples subjected to gastric and intestinal digestion. The digestion electrophoretic profile was realized in 15% SDS-PAGE. Arrows indicate oxidized bands selected for the follow-up of their digestion. Tables show the digestion percentage of bands in gastric and intestinal phases. MW: molecular marker; WD: protein not digested.

In order to evaluate the effect of carbonylation induced by DANO and ENRO (1.0 MRL) on SPs and MPs, digestion was performed on an in vitro model of the gastric and intestinal phases. The results are shown in Figure 6 and Figure 7.

#### 3.4.1. In Vitro Digestibility of Sarcoplasmic and Myofibrillar Proteins Exposed to Danofloxacin (DANO)

To measure the effect induced on SPs digestion, five representative bands were selected (Figure 6a). The densitometric analysis of these bands respect to undigested protein bands (blank sample) showed an effect of decrease in digestibility for the fraction, in either gastric and intestinal phases (Figure 6a). In the case of the gastric phase, the percentage of digestibility was between 11.8% and 38.7%, while the control achieved a range from 63.8% to 91.1%. Pyruvate kinase with 11.8% (band 1) and creatine kinase with 17.3% (band 4), were identified as the proteins with the lowest digestibility and hence with the greatest restriction of digestibility to pepsin activity (88.2% and 82.7%, respectively). For the intestinal phase, the percentage of digestibility was between 64% and 81.4% compared to 79.4% to 96.9% obtained in the blank sample. In this case, pyruvate kinase (band 1), creatine kinase (band 4) and creatine kinase M + actin (band 6) were the proteins with the lower intestinal digestibility, reaching percentages of restriction of digestibility of 36%, 35.9% and 32.2%, respectively.

To evaluate carbonylation effect on MPs digestion were analyzed 4 representative bands. A decrease in digestibility was only observed in the gastric phase with respect to blank sample (Figure 6b). During this phase, 3 bands containing fructose bisphosphate aldolase (band 3), pyruvate kinase (band 7) and the Wh2-actin + beta-enolase domain mixture (band 8) showed a reduced digestion. The restriction of digestibility for these bands was 63.5%, 89.9% and 58.9%, respectively.

When comparing the results obtained for each protein fraction, it is established that the SPs were less digestible than the MPs, both in the gastric and intestinal phases, an effect that also coincides with the high oxidation induced by DANO in these proteins.

#### 3.4.2. In Vitro Digestibility of Sarcoplasmic and Myofibrillar Proteins Exposed to Enrofloxacin (ENRO)

The effects of carbonylation induced by ENRO on SPs digestibility were evaluated from four representative bands (Figure 7). In the gastric phase, digestibility percentages were obtained between 13.9% and 38.0% in contrast with the high digestibility from a blank sample (63.8–91.1%) (Figure 7a). The band 5 containing the fructose bisphosphate aldolase was identified as those with the highest restriction of digestibility (86.1%). Regarding the intestinal phase, the percentage of digestibility was between 34.8% and 54.1% (Figure 7a). Again, the highest restriction was found in the band 5 (65.2%). For MPs, the loss of digestibility were analyzed bands in four selected bands (Figure 7b). However, only band 8 showed restriction in both digestion phases (38.7%).

When comparing the effects induced by DANO and ENRO on the digestion of SPs, both FQs promoting a similar restriction to gastric and intestinal enzymatic activities on protein were analyzed. Due to DANO and ENRO being the FQs that induced the greater carbonylation, the loss of digestibility of this fraction could be related with incorporation of carbonyl groups at or near the cleavage sites of digestive enzymes and the protein aggregates formation [38,47]. In addition, the majority of SPs are globular [48] and their structures have polar amino acids with and without charge, such as tyrosine, arginine and lysine, which in turn correspond to the cleavage sites of digestive enzymes used in this study. Thus, for example, the carbonylation of arginine and lysine residues may affect the enzymatic action of trypsin [19]. In the case of MPs, their structures are mainly fibrous, rich in sulfhydryl groups (cysteine, methionine) and protonable amino acids (histidine, lysine and arginine), the latter being the trypsin cleavage sites [38,49]. These digestion profiles showed that myofibrils are more susceptible to in vitro digestion (Figure 7b), in accordance with the low levels of carbonylation promoted by DANO and ENRO for these proteins.

Thus, these results show that carbonylation induced by ENRO and DANO affect meanly in vitro gastric digestion for both sarcoplasmic and myofibrillar proteins.

## 4. Conclusions

Our results indicate that the presence of NOR, CIPRO, DANO, ENRO and SARA in concentrations close to its MRL promote the carbonylation in vitro of sarcoplasmic and myofibrillar beef proteins; affecting proteins implicated in glycolysis and glycogen. This oxidative effect was associated with loss of solubility and digestibility in both types of proteins. The FQs, DANO and ENRO induced high carbonylation, the SPs were more susceptible to oxidative damage and more resistant to the activity of digestive enzymes than MPs.

The potential effect of veterinary drug residues in food intended for human consumption on oxidative damage, functionality and the nutritional value of dietary proteins remains largely unknown. Therefore, the results of this study are also relevant from a biological point of view, given the implications of protein oxidation products for some human pathological conditions.

## Figures and Tables

**Table 1:** Loss of solubility calculated by protein fraction from blank and treated samples.

Assays	Concentration (µg·Kg^−1^)	SP		MP		Total Protein Loss ^3^ (%)
		Amount of Protein ^1^	Loss (%) ^2^	Amount of Protein ^1^	Loss (%) ^2^	
**BLANK**	---	58.7 ± 0.17	---	2.6 ± 0.01	---	---
**Sarafloxacin**	50	42.5 ± 0.26 ^†^	27.6 ± 0.45 ^a^	2.3 ± 0.04 ^†^	10.8 ± 1.38 ^a^	26.9
	100	39.1 ± 0.08 ^†^	33.4 ± 0.14 ^b^	2.4 ± 0.02 ^†^	9.4 ± 0.84 ^a^	32.4
	150	38.4 ± 0.54 ^†^	34.6 ± 0.92 ^b^	2.3 ± 0.00 ^†^	12.2 ± 0.00 ^a^	33.7
**Danofloxacin**	100	40.8 ± 0.24 ^†^	30.5 ± 0.41 ^c^	2.3 ± 0.00 ^†^	11.1 ± 0.04 ^b^	29.6
	200	44.4 ± 0.11 ^†^	24.3 ± 0.18 ^d^	2.3 ± 0.00 ^†^	10.7 ± 0.04 ^b^	23.8
	300	39.3 ± 0.26 ^†^	33.0 ± 0.45 ^e^	2.5 ± 0.01 ^†^	3.3 ± 0.27 ^c^	31.8
**Enrofloxacin**	50	42.5 ± 0.41 ^†^	27.6 ± 0.69 ^f^	2.5 ± 0.01 ^†^	5.0 ± 0.27 ^d^	26.7
	100	42.0 ± 0.11 ^†^	28.4 ± 0.18 ^f^	2.4 ± 0.01 ^†^	7.3 ± 0.19 ^e^	27.5
	150	38.9 ± 0.06 ^†^	33.8 ± 0.11 ^g^	2.2 ± 0.01 ^†^	16.5 ± 0.38 ^f^	33.0
**Ciprofloxacin**	50	53.4 ± 1.73	0.43 ± 2.07 ^h^	2.6 ± 0.20	2.2 ± 7.62 ^g^	8.8
	100	55.7 ± 0.74	9.1 ± 2.94 ^h^	2.7 ± 0.04	1.7 ± 1.36 ^g^	4.8
	150	38.9 ± 0.06 ^†^	5.1 ± 1.26 ^h^	2.7 ± 0.04	−1.7 ± 1.36 ^g^	32.3
**Norfloxacin**	50	57.7 ± 2.71	1.6 ± 4.62 ^i^	2.2 ± 0.07	15.9 ± 2.83 ^h^	2.2
	100	57.1 ± 0.72	2.7 ± 1.23 ^i^	2.4 ± 0.01	9.2 ± 0.26 ^h^	3.0
	150	56.1 ± 1.99	4.3 ± 3.39 ^i^	2.5 ± 0.09	5.5 ± 3.46 ^h^	4.4

^1^ Expressed as mg of protein·g^−1^ of processed tissue. (Mean ± standard deviation, SD). ^2^ Based on blank. ^3^ Calculated from the sum of sarcoplasmic (SP) and myofibrillar (MP) proteins obtained by assay and blank. Blank represents protein maximum amount extracted with the method described. ^†^ Values statistically different from the blank (*p* < 0.05). Different letters in loss data denote significant differences (*p* < 0.05) between contamination levels of a fluoroquinolone.

**Table 2:** Identification of oxidized sarcoplasmic proteins according bands selected by Western blot from blank and treated samples with danofloxacin or enrofloxacin.

N° Band	Protein Name	Accession Number ^a^	Biological Process ^b^	MW (Da)	Score ^c^	Sequence Coverage (%)	Sample
1	Pyruvate kinase isozymes M1/M2	gi|329664500	Glycolysis	68,416	138	22	dano-enro
2	Beta-enolase + Fructose-bisphosphate aldolase A	gi|77736349 + gi|156120479	Glycolysis	47,409 39,925	118	43 38	dano-enro
3	Fructose-bisphosphate aldolase A	gi|156120479	Glycolysis	39,925	121	45	all
4	Creatine kinase M	gi|4838363	Oxidative stress	43,172	257	40	dano
5	Fructose-bisphosphate aldolase A	gi|156120479	Glycolysis	39,925	130	53	all
6	Phosphoglycerate mutase 2	gi|84000195	Glycolysis	28,838	84	50	dano-enro
7	Triosephosphate isomerase	gi|61888856	Glycolysis	26,901	246	85	dano-enro

^a^ Protein name and accession number were derived from the NCBI database. ^b^ Biological process was derived of Uniprot database. ^c^ The MASCOT baseline significant score is 70, and for the proteins identified in more than one band, the highest score was presented. Supplementary Material containing mass spectrometry (MS) data is available.

**Table 3:** Identification of oxidized myofibrillar proteins according bands selected by Western blot from blank and treated samples with danofloxacin or enrofloxacin.

N° Band	Protein Name	Accession Number ^a^	Biological Process ^b^	MW (Da)	Score ^c^	Sequence Coverage (%)	Sample
3	Myosin-binding protein C	gi|160425243	Muscle contraction	134,923	111	20	dano-enro
4	Phosphorylase glycogen	gi|154426116	Glycogen metabolism	97,683	463	53	all
5	HSP 70 + +mCG5074, isoform CRA_a	gi|261825070	Oxidative stress	45,299 35,231	146	50 40	all
7	Pyruvate kinase	gi|329664500	Glycolysis	68,416	151	39	dano-enro
8	Actin-Bound Wh2 Domains + Beta-enolase	gi|297343122 + gi|77736349	Actin filament nucleation + Glycolisis	40,304 47,409	127	55 38	all
9	Creatine kinase M + Antiparallel Actin Dimer	gi|4838363 + gi|20664362	Oxidative stress + Actin filament nucleation	43,172 41,558	232	58-52	all

^a^ Protein name and accession number were derived from the NCBI database. ^b^ Biological process was derived of Uniprot database. ^c^ The MASCOT baseline significant score is 70, and for the proteins identified in more than one band, the highest score was presented. Supplementary Material containing MS data is available.

## Supplementary Materials

The following are available online at https://www.mdpi.com/2304-8158/9/2/170/s1: Figure S1: Representative electrophoregrams of beef proteins.
