# Supplementary material for: Residues of Fluoroquinolone Antibiotics Induce Carbonylation and Reduce In Vitro Digestion of Sarcoplasmic and Myofibrillar Beef Proteins
Source: Foods. 2020 Feb 11;9(2):170. doi: 10.3390/foods9020170 (PMC7074055; doi:10.3390/foods9020170)
Supplement: Supplementary file 1 [file foods-09-00170-s001.zip › Supplementary Material 1.pdf]

Supplementary Material 1

Residues of fluoroquinolone antibiotics induce carbonylation and reduce in vitro digestion of sarcoplasmic and myofibrillar beef proteins

Johana Márquez-Lázaro[1], Darío Méndez-Cuadro[1] a and Erika Rodríguez-Cavallo [1*]

[1]Analytical Chemistry and Biomedicine Group, University of Cartagena, Cartagena de Indias, Colombia; jmarquezl1@unicartagena.edu.co (J.M.-L); dmendezc@unicartagena.edu.co (D.M.-C); erodriguezc1@unicartagena.edu.co (E.R.-C)

*Correspondence: erodriguezc1@unicartagena.edu.co

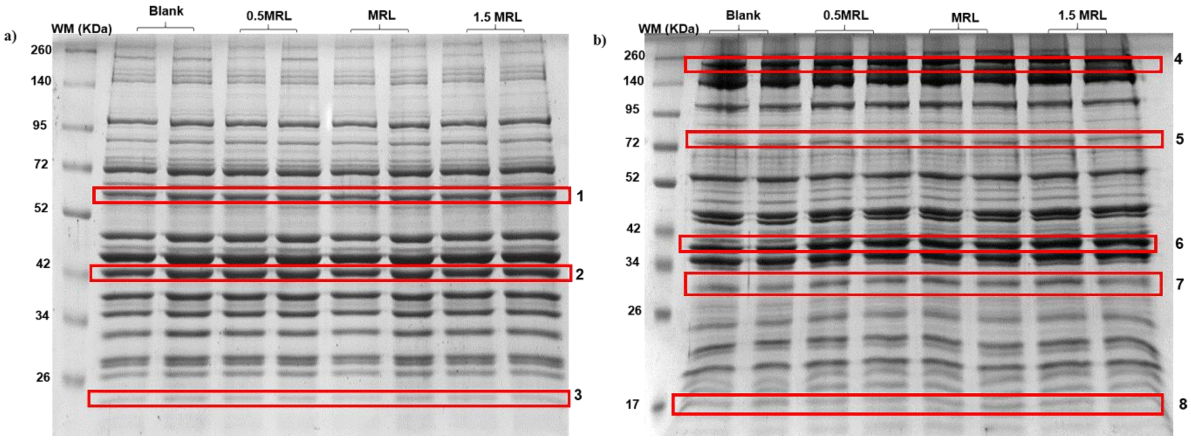

Figure S1. Representative electrophoregrams of beef proteins. a) sarcoplasmic and b) myofibrillar proteins obtained from blank and treated samples with enrofloxacin at 0.5, 1.0 and 1.5MRL (50, 100 and 150 μg.Kg^{-1}, respectively). WM: Weight marker. The gels were stained with Coomassie Blue Brilliant.

Electrophoretic profiles of SPs and MPs were compared againts those reported by *Malava et al.* [1] and *Marcos et al.* [2]. Results shown that carbonylation induced by FQs at its MRL did not induce changes in the migration pattern during SDS-PAGE. Evenmore, typical albumin , creatine kinase and myoglobin bands of SPs (numbered 1-3) were observed. Meanwhile, in the MPs were observed bands 4-8 corresponding to myosin , α-actinine , actin, tropomyosin and troponin C, respectively [1, 3].

References

1. della Malava, A.; Marino, R.; Santillo, A.; Annicchiarico, G.; Caroprese, M.; Sevi, A.; Albenzio, M. Proteomic approach to investigate the impact of different dietary supplementation on lamb meat tenderness. *Meat Sci.* 2017, **131**, 74–81.
2. Marcos, B.; Kerry, J. P; Mullen, A. M. High pressure induced changes on sarcoplasmic protein fraction and quality indicators. *Meat Sci.* 2010, **85**, 115–120.

3. Xia, X.; Kong, B.; Liu, Q.; Liu, J. Physicochemical change and protein oxidation in porcine *longissimus dorsi* as influenced by different freeze-thaw cycles. *Meat Sci.*2009, **83**, 239–245.
