# Supplementary material for: Residues of Fluoroquinolone Antibiotics Induce Carbonylation and Reduce In Vitro Digestion of Sarcoplasmic and Myofibrillar Beef Proteins
Source: Foods. 2020 Feb 11;9(2):170. doi: 10.3390/foods9020170 (PMC7074055; doi:10.3390/foods9020170)

Supplementary Material 2

Results of search in Mascot for Peptide Mass Fingerprint (PMF) and MS/MS Data of sarcoplasmic proteins from beef

Residues of fluoroquinolone antibiotics induce carbonylation and reduce in vitro digestion of sarcoplasmic and myofibrillar beef proteins

Johana Márquez-Lázaro[1], Darío Méndez-Cuadro[1] a and Erika Rodríguez-Cavallo [1*]

[1]Analytical Chemistry and Biomedicine Group, University of Cartagena, Cartagena de Indias, Colombia; jmarquezl1@unicartagena.edu.co (J.M.-L); dmendezc@unicartagena.edu.co (D.M.-C); erodriguezc1@unicartagena.edu.co (E.R.-C)

*Correspondence: erodriguezc1@unicartagena.edu.co

SARCOPLASMIC PROTEINS

BAND 1

Mascot Search Results

User :
Email :
Search title : SampleSetID: 825, AnalysisID: 7244, MaldiWellID: 70018, SpectrumID: 154626, Path=\180719\MSMS\18-106 combined NCBI Mammalia
Database : NCBInr 20120508 (17919084 sequences; 6150218869 residues)
Taxonomy : Mammalia (mammals) (1061927 sequences)
Timestamp : 19 Jul 2018 at 19:26:57 GMT
Warning : A Peptide summary report will usually give a much clearer picture of MS/MS search results.
Top Score : 312 for gi|74267962, ALB protein [Bos taurus]

Mascot Score Histogram

Protein score is -10*Log(P), where P is the probability that the observed match is a random event.
Protein scores greater than 73 are significant (p<0.05).
Protein scores are derived from ions scores as a non-probabilistic basis for ranking protein hits.

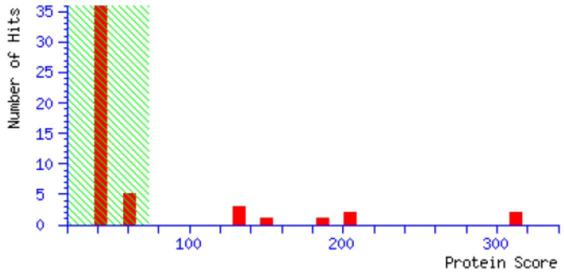

Protein Summary Report

Format As Protein Summary (deprecated) Help
Significance threshold p< 0.05 Max. number of hits 20
Re-Search All Search Unmatched

Index

Accession Mass Score Description
1. gi|74267962 71186 312 ALB protein [Bos taurus]
2. gi|154425704 71244 312 ALB protein [Bos taurus]
3. gi|30794280 71274 202 serum albumin precursor [Bos taurus]
4. gi|1351907 71244 202 RecName: Full=Serum albumin; AltName: Full=BSA; AltName: Allergen=Bos d 6; Flags: Precursor
5. gi|367460260 68416 195 Chain A, Crystal Structure Of Bovine Serum Albumin
6. gi|229552 68083 146 albumin
7. gi|329664500 58482 138 pyruvate kinase isozymes M1/M2 [Bos taurus]
8. gi|73587283 62016 137 PKM2 protein [Bos taurus]
9. gi|76445989 55487 137 serum albumin [Bos indicus]
10. gi|351716030 78894 55 Zinc finger protein 235 [Heterocephalus glaber]
11. gi|148688743 54505 53 mCG2023 [Mus musculus]
12. gi|7657067 54905 53 ERO1-like protein alpha precursor [Mus musculus]
13. gi|338197462 19820 52 CDK5 regulatory subunit associated protein 2 [Ateles paniscus]
14. gi|57164373 71139 52 serum albumin precursor [Ovis aries]
15. gi|2864832 19559 51 MHC class I heavy chain [Bos taurus]
16. gi|126337104 71623 50 PREDICTED: acetylserotonin O-methyltransferase-like [Monodelphis domestica]
17. gi|33504487 51074 49 zinc finger protein 113 [Mus musculus]
18. gi|146336929 13298 48 cryocrystalglobulin CC1 heavy chain variable region [Homo sapiens]
19. gi|338710007 73575 48 PREDICTED: zinc finger protein 30 [Equus caballus]
20. gi|13431676 227743 47 RecName: Full=Myosin-11; AltName: Full=Myosin heavy chain 11; AltName: Full=Myosin heavy chain, smooth muscle isoform; AltName: Full=SMMHC

Results List

1. gi|74267962 Mass: 71186 Score: 312 Expect: 6.7e-026 Matches: 15
ALB protein [Bos taurus]
Observed Mr(expt) Mr(calc) ppm Start End Miss Ions Peptide
1537.6849 1536.6776 1536.7936 -75.48 421 - 433 0 --- K.LGEYGFQNELIVR.Y
1537.6849 1536.6776 1536.7936 -75.48 421 - 433 0 84 K.LGEYGFQNELIVR.Y
1567.6309 1566.6236 1566.7354 -71.37 347 - 359 0 --- K.DAFLGSFLYEYSR.R
1567.6309 1566.6236 1566.7354 -71.37 347 - 359 0 123 K.DAFLGSFLYEYSR.R
1639.8317 1638.8244 1638.9305 -64.71 437 - 451 1 --- R.KVPQVSTPTLVEVSR.S
1694.7345 1693.7272 1693.8167 -52.84 469 - 482 0 --- R.MPCAEDYLSLILNR.L
1710.7329 1709.7256 1709.8116 -50.31 469 - 482 0 --- R.MPCAEDYLSLILNR.L + Oxidation (M)
1880.8608 1879.8535 1879.9138 -32.08 508 - 523 0 --- R.RPCFSALTPDETYVPK.A

1901.8647 1900.8574 1900.8625 -2.68 123 - 138 1 --- R.NECFLSHKDDSPDLPK.L
1907.8650 1906.8577 1906.9135 -29.26 529 - 544 0 --- K.LFTFHADICTLPDTEK.Q
2002.9919 2001.9846 2002.0928 -54.02 3 - 19 0 --- K.WVTFISLLLLFSSAYSR.G
2044.9976 2043.9903 2044.0206 -14.83 168 - 183 1 --- R.RHPYFYAPELLYYANK.Y
2044.9976 2043.9903 2044.0206 -14.83 168 - 183 1 42 R.RHPYFYAPELLYYANK.Y
2492.3208 2491.3135 2491.2570 22.7 45 - 65 0 --- K.GLVLIAFSQYLQQCPFDEHVK.L
2587.2981 2586.2908 2586.2173 28.4 413 - 433 1 --- K.QNCDQFEKLGEYGFQNELIVR.Y
No match to: 927.2980, 1163.4608, 1163.4608, 1293.5264, 1305.5636, 1439.6755, 1456.6536, 1473.6826, 1487.5756, 1496.7140, 1542.6377, 1565.7040, 1594.7124,
1615.7733, 1624.6637, 1642.6763, 1646.7184, 1687.8110, 1703.8551, 1708.7947, 1763.7225, 1782.8079, 1791.8397, 1804.8666, 1808.8822, 1810.8499, 1821.8394,
1837.8445, 1848.8938, 1858.8521, 1858.8521, 1864.8466, 1874.8480, 1896.9047, 1910.8745, 1947.9768, 2102.0327, 2105.0393, 2181.9829, 2225.1506, 2347.1633,
2407.2917, 2493.3369, 2541.2495, 2543.3213, 2570.2813, 2600.3518, 2624.4226, 2672.4282, 2786.4688, 2815.5120, 2981.6201, 3043.7085, 3045.7573, 3281.8982
2. gi|154425704 Mass: 71244 Score: 312 Expect: 6.7e-026 Matches: 15
ALB protein [Bos taurus]
Observed Mr(expt) Mr(calc) ppm Start End Miss Ions Peptide
1537.6849 1536.6776 1536.7936 -75.48 421 - 433 0 --- K.LGEYGFQNELIVR.Y
1537.6849 1536.6776 1536.7936 -75.48 421 - 433 0 84 K.LGEYGFQNELIVR.Y
1567.6309 1566.6236 1566.7354 -71.37 347 - 359 0 --- K.DAFLGSFLYEYSR.R
1567.6309 1566.6236 1566.7354 -71.37 347 - 359 0 123 K.DAFLGSFLYEYSR.R
1639.8317 1638.8244 1638.9305 -64.71 437 - 451 1 --- R.KVPQVSTPTLVEVSR.S
1694.7345 1693.7272 1693.8167 -52.84 469 - 482 0 --- R.MPCAEDYLSLILNR.L
1710.7329 1709.7256 1709.8116 -50.31 469 - 482 0 --- R.MPCAEDYLSLILNR.L + Oxidation (M)
1880.8608 1879.8535 1879.9138 -32.08 508 - 523 0 --- R.RPCFSALTPDETYVPK.A
1901.8647 1900.8574 1900.8625 -2.68 123 - 138 1 --- R.NECFLSHKDDSPDLPK.L
1907.8650 1906.8577 1906.9135 -29.26 529 - 544 0 --- K.LFTFHADICTLPDTEK.Q
2002.9919 2001.9846 2002.0928 -54.02 3 - 19 0 --- K.WVTFISLLLLFSSAYSR.G
2044.9976 2043.9903 2044.0206 -14.83 168 - 183 1 --- R.RHPYFYAPELLYYANK.Y
2044.9976 2043.9903 2044.0206 -14.83 168 - 183 1 42 R.RHPYFYAPELLYYANK.Y
2492.3208 2491.3135 2491.2570 22.7 45 - 65 0 --- K.GLVLIAFSQYLQQCPFDEHVK.L
2587.2981 2586.2908 2586.2173 28.4 413 - 433 1 --- K.QNCDQFEKLGEYGFQNELIVR.Y
No match to: 927.2980, 1163.4608, 1163.4608, 1293.5264, 1305.5636, 1439.6755, 1456.6536, 1473.6826, 1487.5756, 1496.7140, 1542.6377, 1565.7040, 1594.7124,
1615.7733, 1624.6637, 1642.6763, 1646.7184, 1687.8110, 1703.8551, 1708.7947, 1763.7225, 1782.8079, 1791.8397, 1804.8666, 1808.8822, 1810.8499, 1821.8394,
1837.8445, 1848.8938, 1858.8521, 1858.8521, 1864.8466, 1874.8480, 1896.9047, 1910.8745, 1947.9768, 2102.0327, 2105.0393, 2181.9829, 2225.1506, 2347.1633,
2407.2917, 2493.3369, 2541.2495, 2543.3213, 2570.2813, 2600.3518, 2624.4226, 2672.4282, 2786.4688, 2815.5120, 2981.6201, 3043.7085, 3045.7573, 3281.8982
3. gi|30794280 Mass: 71274 Score: 202 Expect: 6.7e-015 Matches: 10
serum albumin precursor [Bos taurus]
Observed Mr(expt) Mr(calc) ppm Start End Miss Ions Peptide
1567.6309 1566.6236 1566.7354 -71.37 347 - 359 0 --- K.DAFLGSFLYEYSR.R
1567.6309 1566.6236 1566.7354 -71.37 347 - 359 0 123 K.DAFLGSFLYEYSR.R
1639.8317 1638.8244 1638.9305 -64.71 437 - 451 1 --- R.KVPQVSTPTLVEVSR.S
1880.8608 1879.8535 1879.9138 -32.08 508 - 523 0 --- R.RPCFSALTPDETYVPK.A
1901.8647 1900.8574 1900.8625 -2.68 123 - 138 1 --- R.NECFLSHKDDSPDLPK.L
1907.8650 1906.8577 1906.9135 -29.26 529 - 544 0 --- K.LFTFHADICTLPDTEK.Q
2002.9919 2001.9846 2002.0928 -54.02 3 - 19 0 --- K.WVTFISLLLLFSSAYSR.G
2044.9976 2043.9903 2044.0206 -14.83 168 - 183 1 --- R.RHPYFYAPELLYYANK.Y
2044.9976 2043.9903 2044.0206 -14.83 168 - 183 1 42 R.RHPYFYAPELLYYANK.Y
2492.3208 2491.3135 2491.2570 22.7 45 - 65 0 --- K.GLVLIAFSQYLQQCPFDEHVK.L
No match to: 927.2980, 1163.4608, 1163.4608, 1293.5264, 1305.5636, 1439.6755, 1456.6536, 1473.6826, 1487.5756, 1496.7140, 1537.6849, 1537.6849, 1542.6377,
1565.7040, 1594.7124, 1615.7733, 1624.6637, 1642.6763, 1646.7184, 1687.8110, 1694.7345, 1703.8551, 1708.7947, 1710.7329, 1763.7225, 1782.8079, 1791.8397,
1804.8666, 1808.8822, 1810.8499, 1821.8394, 1837.8445, 1848.8938, 1858.8521, 1858.8521, 1864.8466, 1874.8480, 1896.9047, 1910.8745, 1947.9768, 2102.0327,
2105.0393, 2181.9829, 2225.1506, 2347.1633, 2407.2917, 2493.3369, 2541.2495, 2543.3213, 2570.2813, 2587.2981, 2600.3518, 2624.4226, 2672.4282, 2786.4688,
2815.5120, 2981.6201, 3043.7085, 3045.7573, 3281.8982
4. gi|1351907 Mass: 71244 Score: 202 Expect: 6.7e-015 Matches: 10
RecName: Full=Serum albumin; AltName: Full=BSA; AltName: Allergen=Bos d 6; Flags: Precursor
Observed Mr(expt) Mr(calc) ppm Start End Miss Ions Peptide
1567.6309 1566.6236 1566.7354 -71.37 347 - 359 0 --- K.DAFLGSFLYEYSR.R
1567.6309 1566.6236 1566.7354 -71.37 347 - 359 0 123 K.DAFLGSFLYEYSR.R
1639.8317 1638.8244 1638.9305 -64.71 437 - 451 1 --- R.KVPQVSTPTLVEVSR.S
1880.8608 1879.8535 1879.9138 -32.08 508 - 523 0 --- R.RPCFSALTPDETYVPK.A
1901.8647 1900.8574 1900.8625 -2.68 123 - 138 1 --- R.NECFLSHKDDSPDLPK.L
1907.8650 1906.8577 1906.9135 -29.26 529 - 544 0 --- K.LFTFHADICTLPDTEK.Q
2002.9919 2001.9846 2002.0928 -54.02 3 - 19 0 --- K.WVTFISLLLLFSSAYSR.G
2044.9976 2043.9903 2044.0206 -14.83 168 - 183 1 --- R.RHPYFYAPELLYYANK.Y
2044.9976 2043.9903 2044.0206 -14.83 168 - 183 1 42 R.RHPYFYAPELLYYANK.Y
2492.3208 2491.3135 2491.2570 22.7 45 - 65 0 --- K.GLVLIAFSQYLQQCPFDEHVK.L
No match to: 927.2980, 1163.4608, 1163.4608, 1293.5264, 1305.5636, 1439.6755, 1456.6536, 1473.6826, 1487.5756, 1496.7140, 1537.6849, 1537.6849, 1542.6377,
1565.7040, 1594.7124, 1615.7733, 1624.6637, 1642.6763, 1646.7184, 1687.8110, 1694.7345, 1703.8551, 1708.7947, 1710.7329, 1763.7225, 1782.8079, 1791.8397,
1804.8666, 1808.8822, 1810.8499, 1821.8394, 1837.8445, 1848.8938, 1858.8521, 1858.8521, 1864.8466, 1874.8480, 1896.9047, 1910.8745, 1947.9768, 2102.0327,
2105.0393, 2181.9829, 2225.1506, 2347.1633, 2407.2917, 2493.3369, 2541.2495, 2543.3213, 2570.2813, 2587.2981, 2600.3518, 2624.4226, 2672.4282, 2786.4688,
2815.5120, 2981.6201, 3043.7085, 3045.7573, 3281.8982
5. gi|367460260 Mass: 68416 Score: 195 Expect: 3.4e-014 Matches: 9
Chain A, Crystal Structure Of Bovine Serum Albumin
Observed Mr(expt) Mr(calc) ppm Start End Miss Ions Peptide
1567.6309 1566.6236 1566.7354 -71.37 323 - 335 0 --- K.DAFLGSFLYEYSR.R
1567.6309 1566.6236 1566.7354 -71.37 323 - 335 0 123 K.DAFLGSFLYEYSR.R
1639.8317 1638.8244 1638.9305 -64.71 413 - 427 1 --- R.KVPQVSTPTLVEVSR.S
1880.8608 1879.8535 1879.9138 -32.08 484 - 499 0 --- R.RPCFSALTPDETYVPK.A

1901.8647 1900.8574 1900.8625 -2.68 99 - 114 1 --- R.NECFLSHKDDSPDLPK.L
1907.8650 1906.8577 1906.9135 -29.26 505 - 520 0 --- K.LFTFHADICTLPDTEK.Q
2044.9976 2043.9903 2044.0206 -14.83 144 - 159 1 --- R.RHPYFYAPELLYYANK.Y
2044.9976 2043.9903 2044.0206 -14.83 144 - 159 1 42 R.RHPYFYAPELLYYANK.Y
2492.3208 2491.3135 2491.2570 22.7 21 - 41 0 --- K.GLVLIAFSQYLQQCPFDEHVK.L
No match to: 927.2980, 1163.4608, 1163.4608, 1293.5264, 1305.5636, 1439.6755, 1456.6536, 1473.6826, 1487.5756, 1496.7140, 1537.6849, 1537.6849, 1542.6377,
1565.7040, 1594.7124, 1615.7733, 1624.6637, 1642.6763, 1646.7184, 1687.8110, 1694.7345, 1703.8551, 1708.7947, 1710.7329, 1763.7225, 1782.8079, 1791.8397,
1804.8666, 1808.8822, 1810.8499, 1821.8394, 1837.8445, 1848.8938, 1858.8521, 1858.8521, 1864.8466, 1874.8480, 1896.9047, 1910.8745, 1947.9768, 2002.9919,
2102.0327, 2105.0393, 2181.9829, 2225.1506, 2347.1633, 2407.2917, 2493.3369, 2541.2495, 2543.3213, 2570.2813, 2587.2981, 2600.3518, 2624.4226, 2672.4282,
2786.4688, 2815.5120, 2981.6201, 3043.7085, 3045.7573, 3281.8982
6. gi|229552 Mass: 68083 Score: 146 Expect: 2.7e-009 Matches: 7
albumin
Observed Mr(expt) Mr(calc) ppm Start End Miss Ions Peptide
1567.6309 1566.6236 1566.7354 -71.37 322 - 334 0 --- K.DAFLGSFLYEYSR.R
1567.6309 1566.6236 1566.7354 -71.37 322 - 334 0 123 K.DAFLGSFLYEYSR.R
1639.8317 1638.8244 1638.9305 -64.71 411 - 425 1 --- R.KVPQVSTPTLVEVSR.S
1880.8608 1879.8535 1879.9138 -32.08 482 - 497 0 --- R.RPCFSALTPDETYVPK.A
1901.8647 1900.8574 1900.8625 -2.68 99 - 114 1 --- R.NECFLSHKDDSPDLPK.L
1907.8650 1906.8577 1906.9135 -29.26 503 - 518 0 --- K.LFTFHADICTLPDTEK.Q
2492.3208 2491.3135 2491.2570 22.7 21 - 41 0 --- K.GLVLIAFSQYLQQCPFDEHVK.L
No match to: 927.2980, 1163.4608, 1163.4608, 1293.5264, 1305.5636, 1439.6755, 1456.6536, 1473.6826, 1487.5756, 1496.7140, 1537.6849, 1537.6849, 1542.6377,
1565.7040, 1594.7124, 1615.7733, 1624.6637, 1642.6763, 1646.7184, 1687.8110, 1694.7345, 1703.8551, 1708.7947, 1710.7329, 1763.7225, 1782.8079, 1791.8397,
1804.8666, 1808.8822, 1810.8499, 1821.8394, 1837.8445, 1848.8938, 1858.8521, 1858.8521, 1864.8466, 1874.8480, 1896.9047, 1910.8745, 1947.9768, 2002.9919,
2044.9976, 2044.9976, 2102.0327, 2105.0393, 2181.9829, 2225.1506, 2347.1633, 2407.2917, 2493.3369, 2541.2495, 2543.3213, 2570.2813, 2587.2981, 2600.3518,
2624.4226, 2672.4282, 2786.4688, 2815.5120, 2981.6201, 3043.7085, 3045.7573, 3281.8982
7. gi|329664500 Mass: 58482 Score: 138 Expect: 1.7e-008 Matches: 13
pyruvate kinase isozymes M1/M2 [Bos taurus]
Observed Mr(expt) Mr(calc) ppm Start End Miss Ions Peptide
1642.6763 1641.6690 1641.7635 -57.52 476 - 489 0 --- K.DPVQEAWAEDVDLR.V
1687.8110 1686.8037 1686.9028 -58.74 462 - 475 1 --- R.QAHLYRGIFPVVCK.D
1821.8394 1820.8321 1820.9091 -42.25 279 - 294 1 --- R.RFDEILEASDGIMVAR.G
1837.8445 1836.8372 1836.9040 -36.34 279 - 294 1 --- R.RFDEILEASDGIMVAR.G + Oxidation (M)
1858.8521 1857.8448 1857.9084 -34.20 231 - 246 0 --- K.FGVEQNVDMVFASFIR.K
1858.8521 1857.8448 1857.9084 -34.20 231 - 246 0 80 K.FGVEQNVDMVFASFIR.K
1874.8480 1873.8407 1873.9033 -33.39 231 - 246 0 --- K.FGVEQNVDMVFASFIR.K + Oxidation (M)
2002.9919 2001.9846 2001.9982 -6.80 231 - 247 1 --- K.FGVEQNVDMVFASFIRK.A + Oxidation (M)
2407.2917 2406.2844 2406.2478 15.2 505 - 526 1 --- K.KGDVVIVLTGWRPGSGFTNTMR.V + Oxidation (M)
2493.3369 2492.3296 2492.2798 20.0 93 - 115 0 --- R.EATESFASDPILYRPVAVALDTK.G
2543.3213 2542.3140 2542.2526 24.2 468 - 489 1 --- R.GIFPVVCKDPVQEAWAEDVDLR.V
2587.2981 2586.2908 2586.2788 4.63 225 - 246 1 --- K.DIQDLKFGVEQNVDMVFASFIR.K + Oxidation (M)
3045.7573 3044.7500 3044.5818 55.2 93 - 120 1 --- R.EATESFASDPILYRPVAVALDTKGPEIR.T
No match to: 927.2980, 1163.4608, 1163.4608, 1293.5264, 1305.5636, 1439.6755, 1456.6536, 1473.6826, 1487.5756, 1496.7140, 1537.6849, 1537.6849, 1542.6377,
1565.7040, 1567.6309, 1567.6309, 1594.7124, 1615.7733, 1624.6637, 1639.8317, 1646.7184, 1694.7345, 1703.8551, 1708.7947, 1710.7329, 1763.7225, 1782.8079,
1791.8397, 1804.8666, 1808.8822, 1810.8499, 1848.8938, 1864.8466, 1880.8608, 1896.9047, 1901.8647, 1907.8650, 1910.8745, 1947.9768, 2044.9976, 2044.9976,
2102.0327, 2105.0393, 2181.9829, 2225.1506, 2347.1633, 2492.3208, 2541.2495, 2570.2813, 2600.3518, 2624.4226, 2672.4282, 2786.4688, 2815.5120, 2981.6201,
3043.7085, 3281.8982
8. gi|73587283 Mass: 62016 Score: 137 Expect: 2.1e-008 Matches: 13
PKM2 protein [Bos taurus]
Observed Mr(expt) Mr(calc) ppm Start End Miss Ions Peptide
1642.6763 1641.6690 1641.7635 -57.52 510 - 523 0 --- K.DPVQEAWAEDVDLR.V
1687.8110 1686.8037 1686.9028 -58.74 496 - 509 1 --- R.QAHLYRGIFPVVCK.D
1821.8394 1820.8321 1820.9091 -42.25 313 - 328 1 --- R.RFDEILEASDGIMVAR.G
1837.8445 1836.8372 1836.9040 -36.34 313 - 328 1 --- R.RFDEILEASDGIMVAR.G + Oxidation (M)
1858.8521 1857.8448 1857.9084 -34.20 265 - 280 0 --- K.FGVEQNVDMVFASFIR.K
1858.8521 1857.8448 1857.9084 -34.20 265 - 280 0 80 K.FGVEQNVDMVFASFIR.K
1874.8480 1873.8407 1873.9033 -33.39 265 - 280 0 --- K.FGVEQNVDMVFASFIR.K + Oxidation (M)
2002.9919 2001.9846 2001.9982 -6.80 265 - 281 1 --- K.FGVEQNVDMVFASFIRK.A + Oxidation (M)
2407.2917 2406.2844 2406.2478 15.2 539 - 560 1 --- K.KGDVVIVLTGWRPGSGFTNTMR.V + Oxidation (M)
2493.3369 2492.3296 2492.2798 20.0 127 - 149 0 --- R.EATESFASDPILYRPVAVALDTK.G
2543.3213 2542.3140 2542.2526 24.2 502 - 523 1 --- R.GIFPVVCKDPVQEAWAEDVDLR.V
2587.2981 2586.2908 2586.2788 4.63 259 - 280 1 --- K.DIQDLKFGVEQNVDMVFASFIR.K + Oxidation (M)
3045.7573 3044.7500 3044.5818 55.2 127 - 154 1 --- R.EATESFASDPILYRPVAVALDTKGPEIR.T
No match to: 927.2980, 1163.4608, 1163.4608, 1293.5264, 1305.5636, 1439.6755, 1456.6536, 1473.6826, 1487.5756, 1496.7140, 1537.6849, 1537.6849, 1542.6377,
1565.7040, 1567.6309, 1567.6309, 1594.7124, 1615.7733, 1624.6637, 1639.8317, 1646.7184, 1694.7345, 1703.8551, 1708.7947, 1710.7329, 1763.7225, 1782.8079,
1791.8397, 1804.8666, 1808.8822, 1810.8499, 1848.8938, 1864.8466, 1880.8608, 1896.9047, 1901.8647, 1907.8650, 1910.8745, 1947.9768, 2044.9976, 2044.9976,
2102.0327, 2105.0393, 2181.9829, 2225.1506, 2347.1633, 2492.3208, 2541.2495, 2570.2813, 2600.3518, 2624.4226, 2672.4282, 2786.4688, 2815.5120, 2981.6201,
3043.7085, 3281.8982
9. gi|76445989 Mass: 55487 Score: 137 Expect: 2.1e-008 Matches: 5
serum albumin [Bos indicus]
Observed Mr(expt) Mr(calc) ppm Start End Miss Ions Peptide
1567.6309 1566.6236 1566.7354 -71.37 216 - 228 0 --- K.DAFLGSFLYEYSR.R
1567.6309 1566.6236 1566.7354 -71.37 216 - 228 0 123 K.DAFLGSFLYEYSR.R
1639.8317 1638.8244 1638.9305 -64.71 306 - 320 1 --- R.KVPQVSTPTLVEVSR.S
1880.8608 1879.8535 1879.9138 -32.08 377 - 392 0 --- R.RPCFSALTPDETYVPK.A
1907.8650 1906.8577 1906.9135 -29.26 398 - 413 0 --- K.LFTFHADICTLPDTEK.Q

No match to: 927.2980, 1163.4608, 1163.4608, 1293.5264, 1305.5636, 1439.6755, 1456.6536, 1473.6826, 1487.5756, 1496.7140, 1537.6849, 1537.6849, 1542.6377,
1565.7040, 1594.7124, 1615.7733, 1624.6637, 1642.6763, 1646.7184, 1687.8110, 1694.7345, 1703.8551, 1708.7947, 1710.7329, 1763.7225, 1782.8079, 1791.8397,
1804.8666, 1808.8822, 1810.8499, 1821.8394, 1837.8445, 1848.8938, 1858.8521, 1858.8521, 1864.8466, 1874.8480, 1896.9047, 1901.8647, 1910.8745, 1947.9768,
2002.9919, 2044.9976, 2044.9976, 2102.0327, 2105.0393, 2181.9829, 2225.1506, 2347.1633, 2407.2917, 2492.3208, 2493.3369, 2541.2495, 2543.3213, 2570.2813,
2587.2981, 2600.3518, 2624.4226, 2672.4282, 2786.4688, 2815.5120, 2981.6201, 3043.7085, 3045.7573, 3281.8982
10. gi|351716030 Mass: 78894 Score: 55 Expect: 3.6 Matches: 12
Zinc finger protein 235 [Heterocephalus glaber]
Observed Mr(expt) Mr(calc) ppm Start End Miss Ions Peptide
927.2980 926.2907 926.3222 -33.98 2 - 8 0 --- M.ESSCCER.V
1305.5636 1304.5563 1304.6547 -75.40 88 - 97 0 --- K.LWMTEIQTQR.G
1473.6826 1472.6753 1472.6579 11.8 458 - 469 0 --- R.FSCSSNLHTHQR.V
1542.6377 1541.6304 1541.7011 -45.87 541 - 553 0 --- K.GFSQSSYFQAHQR.V
1565.7040 1564.6967 1564.7647 -43.46 570 - 581 0 --- R.FNWSLNLHNHQR.V
1791.8397 1790.8324 1790.8444 -6.67 554 - 568 1 --- R.VHTGEKPYKCEVCGK.R
1821.8394 1820.8321 1820.8185 7.46 582 - 596 1 --- R.VHTGEKPYKCEECGK.G
1858.8521 1857.8448 1857.8349 5.33 250 - 265 1 --- K.MEEAHSHSTCGKEVLK.V + Oxidation (M)
1858.8521 1857.8448 1857.8349 5.33 250 - 265 1 --- K.MEEAHSHSTCGKEVLK.V + Oxidation (M)
2347.1633 2346.1560 2346.1240 13.6 184 - 203 1 --- R.SNVIENQEFPTERAQSPWSK.I
2570.2813 2569.2740 2569.2594 5.68 204 - 224 1 --- K.IYLSETQNQQNNYKPTQMKNK.L
2672.4282 2671.4209 2671.3078 42.4 485 - 506 1 --- K.CFSLSFNLHSHQRVHTGEKPYK.C
No match to: 1163.4608, 1163.4608, 1293.5264, 1439.6755, 1456.6536, 1487.5756, 1496.7140, 1537.6849, 1537.6849, 1567.6309, 1567.6309, 1594.7124, 1615.7733,
1624.6637, 1639.8317, 1642.6763, 1646.7184, 1687.8110, 1694.7345, 1703.8551, 1708.7947, 1710.7329, 1763.7225, 1782.8079, 1804.8666, 1808.8822, 1810.8499,
1837.8445, 1848.8938, 1864.8466, 1874.8480, 1880.8608, 1896.9047, 1901.8647, 1907.8650, 1910.8745, 1947.9768, 2002.9919, 2044.9976, 2044.9976, 2102.0327,
2105.0393, 2181.9829, 2225.1506, 2407.2917, 2492.3208, 2493.3369, 2541.2495, 2543.3213, 2587.2981, 2600.3518, 2624.4226, 2786.4688, 2815.5120, 2981.6201,
3043.7085, 3045.7573, 3281.8982
11. gi|148688743 Mass: 54505 Score: 53 Expect: 5.4 Matches: 10
mCG2023 [Mus musculus]
Observed Mr(expt) Mr(calc) ppm Start End Miss Ions Peptide
1163.4608 1162.4535 1162.4924 -33.46 127 - 135 0 --- R.IEECEQAER.L
1163.4608 1162.4535 1162.4924 -33.46 127 - 135 0 --- R.IEECEQAER.L
1496.7140 1495.7067 1495.7347 -18.70 68 - 78 1 --- K.LLESDYFRYYK.V
1615.7733 1614.7660 1614.7317 21.3 376 - 388 1 --- R.NISRIMDCVGCFK.C + Oxidation (M)
1791.8397 1790.8324 1790.7981 19.2 83 - 96 0 --- K.KPCPFWNDINQCGR.R
1901.8647 1900.8574 1900.8665 -4.79 222 - 236 0 --- R.ENTFYNWLEGLCVEK.R
1947.9768 1946.9695 1946.8992 36.1 83 - 97 1 --- K.KPCPFWNDINQCGRR.D
2225.1506 2224.1433 2224.2229 -35.76 238 - 257 1 --- R.AFYRLISGLHASINVHLSAR.Y
2543.3213 2542.3140 2542.4536 -54.91 4 - 26 1 --- R.AWGLLVGLLGVVWLLRLGHGEER.R
2786.4688 2785.4615 2785.3996 22.2 406 - 429 1 --- K.ILFSEKLIANMPESGPSYEFQLTR.Q + Oxidation (M)
No match to: 927.2980, 1293.5264, 1305.5636, 1439.6755, 1456.6536, 1473.6826, 1487.5756, 1537.6849, 1537.6849, 1542.6377, 1565.7040, 1567.6309, 1567.6309,
1594.7124, 1624.6637, 1639.8317, 1642.6763, 1646.7184, 1687.8110, 1694.7345, 1703.8551, 1708.7947, 1710.7329, 1763.7225, 1782.8079, 1804.8666, 1808.8822,
1810.8499, 1821.8394, 1837.8445, 1848.8938, 1858.8521, 1858.8521, 1864.8466, 1874.8480, 1880.8608, 1896.9047, 1907.8650, 1910.8745, 2002.9919, 2044.9976,
2044.9976, 2102.0327, 2105.0393, 2181.9829, 2347.1633, 2407.2917, 2492.3208, 2493.3369, 2541.2495, 2570.2813, 2587.2981, 2600.3518, 2624.4226, 2672.4282,
2815.5120, 2981.6201, 3043.7085, 3045.7573, 3281.8982
12. gi|7657067 Mass: 54905 Score: 53 Expect: 5.8 Matches: 10
ERO1-like protein alpha precursor [Mus musculus]
Observed Mr(expt) Mr(calc) ppm Start End Miss Ions Peptide
1163.4608 1162.4535 1162.4924 -33.46 127 - 135 0 --- R.IEECEQAER.L
1163.4608 1162.4535 1162.4924 -33.46 127 - 135 0 --- R.IEECEQAER.L
1496.7140 1495.7067 1495.7347 -18.70 68 - 78 1 --- K.LLESDYFRYYK.V
1615.7733 1614.7660 1614.7317 21.3 380 - 392 1 --- R.NISRIMDCVGCFK.C + Oxidation (M)
1791.8397 1790.8324 1790.7981 19.2 83 - 96 0 --- K.KPCPFWNDINQCGR.R
1901.8647 1900.8574 1900.8665 -4.79 226 - 240 0 --- K.ENTFYNWLEGLCVEK.R
1947.9768 1946.9695 1946.8992 36.1 83 - 97 1 --- K.KPCPFWNDINQCGRR.D
2225.1506 2224.1433 2224.2229 -35.76 242 - 261 1 --- R.AFYRLISGLHASINVHLSAR.Y
2543.3213 2542.3140 2542.4536 -54.91 4 - 26 1 --- R.AWGLLVGLLGVVWLLRLGHGEER.R
2786.4688 2785.4615 2785.3996 22.2 410 - 433 1 --- K.ILFSEKLIANMPESGPSYEFQLTR.Q + Oxidation (M)
No match to: 927.2980, 1293.5264, 1305.5636, 1439.6755, 1456.6536, 1473.6826, 1487.5756, 1537.6849, 1537.6849, 1542.6377, 1565.7040, 1567.6309, 1567.6309,
1594.7124, 1624.6637, 1639.8317, 1642.6763, 1646.7184, 1687.8110, 1694.7345, 1703.8551, 1708.7947, 1710.7329, 1763.7225, 1782.8079, 1804.8666, 1808.8822,
1810.8499, 1821.8394, 1837.8445, 1848.8938, 1858.8521, 1858.8521, 1864.8466, 1874.8480, 1880.8608, 1896.9047, 1907.8650, 1910.8745, 2002.9919, 2044.9976,
2044.9976, 2102.0327, 2105.0393, 2181.9829, 2347.1633, 2407.2917, 2492.3208, 2493.3369, 2541.2495, 2570.2813, 2587.2981, 2600.3518, 2624.4226, 2672.4282,
2815.5120, 2981.6201, 3043.7085, 3045.7573, 3281.8982
13. gi|338197462 Mass: 19820 Score: 52 Expect: 6.4 Matches: 6
CDK5 regulatory subunit associated protein 2 [Ateles paniscus]
Observed Mr(expt) Mr(calc) ppm Start End Miss Ions Peptide
1542.6377 1541.6304 1541.7330 -66.54 131 - 143 0 --- K.ILHQLAPEMMDGR.T + 2 Oxidation (M)
1791.8397 1790.8324 1790.7741 32.6 109 - 124 0 --- R.GSDEEEMTSSSLHQVR.Y
1947.9768 1946.9695 1946.8752 48.4 108 - 124 1 --- K.RGSDEEEMTSSSLHQVR.Y
2181.9829 2180.9756 2181.0008 -11.54 109 - 127 1 --- R.GSDEEEMTSSSLHQVRYVK.H
2225.1506 2224.1433 2224.0980 20.4 131 - 149 1 --- K.ILHQLAPEMMDGRTPENLK.R + 2 Oxidation (M)
2786.4688 2785.4615 2785.3375 44.5 82 - 107 0 --- K.HSQCSEAIITVLCGTEGAQDGLSKPK.R
No match to: 927.2980, 1163.4608, 1163.4608, 1293.5264, 1305.5636, 1439.6755, 1456.6536, 1473.6826, 1487.5756, 1496.7140, 1537.6849, 1537.6849, 1565.7040,
1567.6309, 1567.6309, 1594.7124, 1615.7733, 1624.6637, 1639.8317, 1642.6763, 1646.7184, 1687.8110, 1694.7345, 1703.8551, 1708.7947, 1710.7329, 1763.7225,
1782.8079, 1804.8666, 1808.8822, 1810.8499, 1821.8394, 1837.8445, 1848.8938, 1858.8521, 1858.8521, 1864.8466, 1874.8480, 1880.8608, 1896.9047, 1901.8647,

1907.8650, 1910.8745, 2002.9919, 2044.9976, 2044.9976, 2102.0327, 2105.0393, 2347.1633, 2407.2917, 2492.3208, 2493.3369, 2541.2495, 2543.3213, 2570.2813,
2587.2981, 2600.3518, 2624.4226, 2672.4282, 2815.5120, 2981.6201, 3043.7085, 3045.7573, 3281.8982
14. gi|57164373 Mass: 71139 Score: 52 Expect: 6.7 Matches: 4
serum albumin precursor [Ovis aries]
Observed Mr(expt) Mr(calc) ppm Start End Miss Ions Peptide
2002.9919 2001.9846 2002.0928 -54.02 3 - 19 0 --- K.WVTFISLLLLFSSAYSR.G
2044.9976 2043.9903 2044.0206 -14.83 168 - 183 1 --- R.RHPYFYAPELLYYANK.Y
2044.9976 2043.9903 2044.0206 -14.83 168 - 183 1 42 R.RHPYFYAPELLYYANK.Y
2981.6201 2980.6128 2980.4124 67.2 310 - 336 1 --- K.SHCIAEVDKDAVPENLPPLTADFAEDK.E
No match to: 927.2980, 1163.4608, 1163.4608, 1293.5264, 1305.5636, 1439.6755, 1456.6536, 1473.6826, 1487.5756, 1496.7140, 1537.6849, 1537.6849, 1542.6377,
1565.7040, 1567.6309, 1567.6309, 1594.7124, 1615.7733, 1624.6637, 1639.8317, 1642.6763, 1646.7184, 1687.8110, 1694.7345, 1703.8551, 1708.7947, 1710.7329,
1763.7225, 1782.8079, 1791.8397, 1804.8666, 1808.8822, 1810.8499, 1821.8394, 1837.8445, 1848.8938, 1858.8521, 1858.8521, 1864.8466, 1874.8480, 1880.8608,
1896.9047, 1901.8647, 1907.8650, 1910.8745, 1947.9768, 2102.0327, 2105.0393, 2181.9829, 2225.1506, 2347.1633, 2407.2917, 2492.3208, 2493.3369, 2541.2495,
2543.3213, 2570.2813, 2587.2981, 2600.3518, 2624.4226, 2672.4282, 2786.4688, 2815.5120, 3043.7085, 3045.7573, 3281.8982
15. gi|2864832 Mass: 19559 Score: 51 Expect: 7.9 Matches: 7
MHC class I heavy chain [Bos taurus]
Observed Mr(expt) Mr(calc) ppm Start End Miss Ions Peptide
1487.5756 1486.5683 1486.6801 -75.15 31 - 43 1 --- R.FDSDSPNPRAEPR.A
1567.6309 1566.6236 1566.7137 -57.46 153 - 164 0 --- R.NYLEGECVEWLR.R
1567.6309 1566.6236 1566.7137 -57.46 153 - 164 0 --- R.NYLEGECVEWLR.R
1708.7947 1707.7874 1707.8580 -41.33 2 - 16 0 --- R.YFSTAVSRPGLEEPR.F
1864.8466 1863.8393 1863.9591 -64.27 1 - 16 1 --- -.RYFSTAVSRPGLEEPR.F
2492.3208 2491.3135 2491.1478 66.5 44 - 63 1 --- R.APWMEQEGPEYWDEQTRIVK.D
2624.4226 2623.4153 2623.2765 52.9 117 - 139 1 --- R.DYLALNEDLRSWTAGETEAQITK.R
No match to: 927.2980, 1163.4608, 1163.4608, 1293.5264, 1305.5636, 1439.6755, 1456.6536, 1473.6826, 1496.7140, 1537.6849, 1537.6849, 1542.6377, 1565.7040,
1594.7124, 1615.7733, 1624.6637, 1639.8317, 1642.6763, 1646.7184, 1687.8110, 1694.7345, 1703.8551, 1710.7329, 1763.7225, 1782.8079, 1791.8397, 1804.8666,
1808.8822, 1810.8499, 1821.8394, 1837.8445, 1848.8938, 1858.8521, 1858.8521, 1874.8480, 1880.8608, 1896.9047, 1901.8647, 1907.8650, 1910.8745, 1947.9768,
2002.9919, 2044.9976, 2044.9976, 2102.0327, 2105.0393, 2181.9829, 2225.1506, 2347.1633, 2407.2917, 2493.3369, 2541.2495, 2543.3213, 2570.2813, 2587.2981,
2600.3518, 2672.4282, 2786.4688, 2815.5120, 2981.6201, 3043.7085, 3045.7573, 3281.8982
16. gi|126337104 Mass: 71623 Score: 50 Expect: 10 Matches: 11
PREDICTED: acetylserotonin O-methyltransferase-like [Monodelphis domestica]
Observed Mr(expt) Mr(calc) ppm Start End Miss Ions Peptide
1439.6755 1438.6682 1438.6147 37.2 258 - 271 0 --- K.GESAFSCGDPGSLR.K
1473.6826 1472.6753 1472.7908 -78.43 351 - 363 0 --- R.LLDACTALELLNK.T
1565.7040 1564.6967 1564.7676 -45.31 439 - 450 1 --- K.LRFMNAMHCIIK.V + 2 Oxidation (M)
1567.6309 1566.6236 1566.7097 -54.91 258 - 272 1 --- K.GESAFSCGDPGSLRK.K
1567.6309 1566.6236 1566.7097 -54.91 258 - 272 1 --- K.GESAFSCGDPGSLRK.K
1791.8397 1790.8324 1790.8250 4.12 136 - 149 0 --- K.QLETEVFEFYEETK.V
1804.8666 1803.8593 1803.9189 -33.03 581 - 596 0 --- R.SAWLQSLNMLVQTEGK.E
1810.8499 1809.8426 1809.9155 -40.29 334 - 350 1 --- K.AKDVAAHINASVCGIER.L
1910.8745 1909.8672 1910.0149 -77.32 539 - 554 1 --- R.ILHDWSDDKIDILLSK.I
2541.2495 2540.2422 2540.1417 39.6 152 - 172 0 --- K.FSDLSEELLWEYIHSGEPMDK.A + Oxidation (M)
2570.2813 2569.2740 2569.3224 -18.82 610 - 631 1 --- R.HGFRNVQIVQTGNWLDAILCTK.C
No match to: 927.2980, 1163.4608, 1163.4608, 1293.5264, 1305.5636, 1456.6536, 1487.5756, 1496.7140, 1537.6849, 1537.6849, 1542.6377, 1594.7124, 1615.7733,
1624.6637, 1639.8317, 1642.6763, 1646.7184, 1687.8110, 1694.7345, 1703.8551, 1708.7947, 1710.7329, 1763.7225, 1782.8079, 1808.8822, 1821.8394, 1837.8445,
1848.8938, 1858.8521, 1858.8521, 1864.8466, 1874.8480, 1880.8608, 1896.9047, 1901.8647, 1907.8650, 1947.9768, 2002.9919, 2044.9976, 2044.9976, 2102.0327,
2105.0393, 2181.9829, 2225.1506, 2347.1633, 2407.2917, 2492.3208, 2493.3369, 2543.3213, 2587.2981, 2600.3518, 2624.4226, 2672.4282, 2786.4688, 2815.5120,
2981.6201, 3043.7085, 3045.7573, 3281.8982
17. gi|33504487 Mass: 51074 Score: 49 Expect: 15 Matches: 8
zinc finger protein 113 [Mus musculus]
Observed Mr(expt) Mr(calc) ppm Start End Miss Ions Peptide
1763.7225 1762.7152 1762.7767 -34.86 306 - 320 0 --- R.IHTGEKPYACNECGK.A
1821.8394 1820.8321 1820.8185 7.46 250 - 264 1 --- R.IHTGEKPYECKDCGK.T
1848.8938 1847.8865 1847.7964 48.8 194 - 208 1 --- R.LQMGDKPHKCDECSK.S + Oxidation (M)
2002.9919 2001.9846 2001.8990 42.8 79 - 96 0 --- R.DVMLENYGNVFSLDGDSK.T
2102.0327 2101.0254 2101.0520 -12.67 48 - 64 0 --- K.SQELVTFEDVAVYFIWR.E
2181.9829 2180.9756 2180.9976 -10.10 97 - 116 1 --- K.TGNDRVISEGMGSCEMILGR.F
2225.1506 2224.1433 2224.0153 57.5 306 - 324 1 --- R.IHTGEKPYACNECGKAFSR.S
3281.8982 3280.8909 3280.6285 80.0 1 - 31 1 --- -.METQADHASQAPLPLLESALSSSKVPSFPDK.D
No match to: 927.2980, 1163.4608, 1163.4608, 1293.5264, 1305.5636, 1439.6755, 1456.6536, 1473.6826, 1487.5756, 1496.7140, 1537.6849, 1537.6849, 1542.6377,
1565.7040, 1567.6309, 1567.6309, 1594.7124, 1615.7733, 1624.6637, 1639.8317, 1642.6763, 1646.7184, 1687.8110, 1694.7345, 1703.8551, 1708.7947, 1710.7329,
1782.8079, 1791.8397, 1804.8666, 1808.8822, 1810.8499, 1837.8445, 1858.8521, 1858.8521, 1864.8466, 1874.8480, 1880.8608, 1896.9047, 1901.8647, 1907.8650,
1910.8745, 1947.9768, 2044.9976, 2044.9976, 2105.0393, 2347.1633, 2407.2917, 2492.3208, 2493.3369, 2541.2495, 2543.3213, 2570.2813, 2587.2981, 2600.3518,
2624.4226, 2672.4282, 2786.4688, 2815.5120, 2981.6201, 3043.7085, 3045.7573
18. gi|146336929 Mass: 13298 Score: 48 Expect: 15 Matches: 5
cryocrystalglobulin CC1 heavy chain variable region [Homo sapiens]
Observed Mr(expt) Mr(calc) ppm Start End Miss Ions Peptide
1782.8079 1781.8006 1781.8730 -40.62 73 - 87 1 --- R.DNSKNTVYLQMNSLR.A
1791.8397 1790.8324 1790.8331 -0.38 88 - 102 1 --- R.AEDTALYYCAKMLSR.S
1907.8650 1906.8577 1906.9061 -25.39 103 - 120 0 --- R.SSHFLDSWGQGTLVTVSS.-
2624.4226 2623.4153 2623.2410 66.4 77 - 98 1 --- K.NTVYLQMNSLRAEDTALYYCAK.M
2672.4282 2671.4209 2671.2424 66.8 20 - 43 1 --- R.LSCAASGFTFSNYGMHWVRQAPGK.G
No match to: 927.2980, 1163.4608, 1163.4608, 1293.5264, 1305.5636, 1439.6755, 1456.6536, 1473.6826, 1487.5756, 1496.7140, 1537.6849, 1537.6849, 1542.6377,

1565.7040, 1567.6309, 1567.6309, 1594.7124, 1615.7733, 1624.6637, 1639.8317, 1642.6763, 1646.7184, 1687.8110, 1694.7345, 1703.8551, 1708.7947, 1710.7329,
1763.7225, 1804.8666, 1808.8822, 1810.8499, 1821.8394, 1837.8445, 1848.8938, 1858.8521, 1858.8521, 1864.8466, 1874.8480, 1880.8608, 1896.9047, 1901.8647,
1910.8745, 1947.9768, 2002.9919, 2044.9976, 2044.9976, 2102.0327, 2105.0393, 2181.9829, 2225.1506, 2347.1633, 2407.2917, 2492.3208, 2493.3369, 2541.2495,
2543.3213, 2570.2813, 2587.2981, 2600.3518, 2786.4688, 2815.5120, 2981.6201, 3043.7085, 3045.7573, 3281.8982
19. gi|338710007 Mass: 73575 Score: 48 Expect: 15 Matches: 10
PREDICTED: zinc finger protein 30 [Equus caballus]
Observed Mr(expt) Mr(calc) ppm Start End Miss Ions Peptide
1694.7345 1693.7272 1693.8359 -64.14 251 - 264 0 --- R.HQNIHTGEKPFVCK.E
1703.8551 1702.8478 1702.8283 11.5 366 - 380 1 --- R.IHAGVKPYGCKECGK.A
1708.7947 1707.7874 1707.7933 -3.46 195 - 208 1 --- K.HGRIHTGEKPCECK.E
1710.7329 1709.7256 1709.8420 -68.06 307 - 320 1 --- K.HQRIHTGEKPYGCK.E
1763.7225 1762.7152 1762.8131 -55.50 310 - 324 1 --- R.IHTGEKPYGCKECGK.A
1821.8394 1820.8321 1820.8185 7.46 506 - 520 1 --- R.IHTGEKPYECKDCGK.A
1848.8938 1847.8865 1847.8294 30.9 226 - 240 1 --- R.IHNGEKPYECKECGK.A
1874.8480 1873.8407 1873.9178 -41.16 478 - 492 1 --- K.IHINVKPYECKECGK.T
1907.8650 1906.8577 1906.8189 20.3 422 - 436 0 --- R.IHTGEKPYECQECEK.A
2347.1633 2346.1560 2346.1651 -3.88 442 - 460 1 --- R.HQLTVHQRVHTGEKPYECK.D
No match to: 927.2980, 1163.4608, 1163.4608, 1293.5264, 1305.5636, 1439.6755, 1456.6536, 1473.6826, 1487.5756, 1496.7140, 1537.6849, 1537.6849, 1542.6377,
1565.7040, 1567.6309, 1567.6309, 1594.7124, 1615.7733, 1624.6637, 1639.8317, 1642.6763, 1646.7184, 1687.8110, 1782.8079, 1791.8397, 1804.8666, 1808.8822,
1810.8499, 1837.8445, 1858.8521, 1858.8521, 1864.8466, 1880.8608, 1896.9047, 1901.8647, 1910.8745, 1947.9768, 2002.9919, 2044.9976, 2044.9976, 2102.0327,
2105.0393, 2181.9829, 2225.1506, 2407.2917, 2492.3208, 2493.3369, 2541.2495, 2543.3213, 2570.2813, 2587.2981, 2600.3518, 2624.4226, 2672.4282, 2786.4688,
2815.5120, 2981.6201, 3043.7085, 3045.7573, 3281.8982
20. gi|13431676 Mass: 227743 Score: 47 Expect: 19 Matches: 19
RecName: Full=Myosin-11; AltName: Full=Myosin heavy chain 11; AltName: Full=Myosin heavy chain, smooth muscle isoform; AltName: Full=SMMHC
Observed Mr(expt) Mr(calc) ppm Start End Miss Ions Peptide
1496.7140 1495.7067 1495.7262 -13.01 1508 - 1520 1 --- K.MLKAEMEDLVSSK.D + Oxidation (M)
1567.6309 1566.6236 1566.6467 -14.76 1906 - 1919 0 --- R.ELDEATESNEAMGR.E + Oxidation (M)
1567.6309 1566.6236 1566.6467 -14.76 1906 - 1919 0 --- R.ELDEATESNEAMGR.E + Oxidation (M)
1642.6763 1641.6690 1641.7668 -59.55 918 - 930 0 --- K.QELEEILHEMEAR.L + Oxidation (M)
1694.7345 1693.7272 1693.7101 10.1 1947 - 1961 0 --- R.VIENTDGSEEEMDAR.D
1710.7329 1709.7256 1709.7050 12.1 1947 - 1961 0 --- R.VIENTDGSEEEMDAR.D + Oxidation (M)
1782.8079 1781.8006 1781.7447 31.4 850 - 863 1 --- R.QEEEMQAKEEEMQK.I + Oxidation (M)
1837.8445 1836.8372 1836.8651 -15.17 19 - 34 1 --- K.NFMNSPMAQADWVAKK.L
1901.8647 1900.8574 1900.9854 -67.30 1761 - 1777 1 --- R.KATLQAEQLSNELATER.S
1907.8650 1906.8577 1906.8280 15.6 1546 - 1562 0 --- K.TQLEESEDDVQATEDAK.L
1947.9768 1946.9695 1946.9255 22.6 1285 - 1302 0 --- K.LQNEVESVTGMLNEAEGK.A
2002.9919 2001.9846 2001.9677 8.47 1684 - 1701 0 --- K.SLEADLMQLQEDLAAAER.A
2044.9976 2043.9903 2043.9279 30.5 1182 - 1198 1 --- K.ALDEETRSHEAQVQEMR.Q + Oxidation (M)
2044.9976 2043.9903 2043.9279 30.5 1182 - 1198 1 --- K.ALDEETRSHEAQVQEMR.Q + Oxidation (M)
2407.2917 2406.2844 2406.1042 74.9 947 - 966 1 --- K.KMAQQMLDLEEQLEEEEAAR.Q + Oxidation (M)
2541.2495 2540.2422 2540.2394 1.10 1060 - 1082 0 --- K.LEGDASDFHEQIADLQAQIAELK.M
2543.3213 2542.3140 2542.1492 64.8 918 - 937 1 --- K.QELEEILHEMEARLEEEEDR.R + Oxidation (M)
2672.4282 2671.4209 2671.3664 20.4 1306 - 1329 1 --- K.LAKDVASLGSQLQDTQELLQEETR.Q
2786.4688 2785.4615 2785.4497 4.23 207 - 232 1 --- K.DSSITGELEKQLLQANPILEAFGNAK.T
No match to: 927.2980, 1163.4608, 1163.4608, 1293.5264, 1305.5636, 1439.6755, 1456.6536, 1473.6826, 1487.5756, 1537.6849, 1537.6849, 1542.6377, 1565.7040,
1594.7124, 1615.7733, 1624.6637, 1639.8317, 1646.7184, 1687.8110, 1703.8551, 1708.7947, 1763.7225, 1791.8397, 1804.8666, 1808.8822, 1810.8499, 1821.8394,
1848.8938, 1858.8521, 1858.8521, 1864.8466, 1874.8480, 1880.8608, 1896.9047, 1910.8745, 2102.0327, 2105.0393, 2181.9829, 2225.1506, 2347.1633, 2492.3208,
2493.3369, 2570.2813, 2587.2981, 2600.3518, 2624.4226, 2815.5120, 2981.6201, 3043.7085, 3045.7573, 3281.8982

Search Parameters

Type of search : Sequence Query
Enzyme : Trypsin
Fixed modifications : Carbamidomethyl (C)
Variable modifications : Oxidation (M)
Mass values : Monoisotopic
Protein Mass : Unrestricted
Peptide Mass Tolerance : ± 80 ppm
Fragment Mass Tolerance: ± 0.3 Da
Max Missed Cleavages : 1
Instrument type : MALDI-TOF-TOF
Query1 (927.2980,1+) : <no title>
Query2 (1163.4608,1+) : <no title>
Query3 (1163.4608,1+) : MaldiWellID: 70018, SpectrumID: 154631,
Query4 (1293.5264,1+) : <no title>
Query5 (1305.5636,1+) : <no title>
Query6 (1439.6755,1+) : <no title>
Query7 (1456.6536,1+) : <no title>
Query8 (1473.6826,1+) : <no title>
Query9 (1487.5756,1+) : <no title>
Query10 (1496.7140,1+) : <no title>
Query11 (1537.6849,1+) : <no title>
Query12 (1537.6849,1+) : MaldiWellID: 70018, SpectrumID: 154628,
Query13 (1542.6377,1+) : <no title>
Query14 (1565.7040,1+) : <no title>
Query15 (1567.6309,1+) : <no title>
Query16 (1567.6309,1+) : MaldiWellID: 70018, SpectrumID: 154627,
Query17 (1594.7124,1+) : <no title>
Query18 (1615.7733,1+) : <no title>
Query19 (1624.6637,1+) : <no title>
Query20 (1639.8317,1+) : <no title>
Query21 (1642.6763,1+) : <no title>

Query22 (1646.7184,1+) : <no title>
Query23 (1687.8110,1+) : <no title>
Query24 (1694.7345,1+) : <no title>
Query25 (1703.8551,1+) : <no title>
Query26 (1708.7947,1+) : <no title>
Query27 (1710.7329,1+) : <no title>
Query28 (1763.7225,1+) : <no title>
Query29 (1782.8079,1+) : <no title>
Query30 (1791.8397,1+) : <no title>
Query31 (1804.8666,1+) : <no title>
Query32 (1808.8822,1+) : <no title>
Query33 (1810.8499,1+) : <no title>
Query34 (1821.8394,1+) : <no title>
Query35 (1837.8445,1+) : <no title>
Query36 (1848.8938,1+) : <no title>
Query37 (1858.8521,1+) : <no title>
Query38 (1858.8521,1+) : MaldiWellID: 70018, SpectrumID: 154629,
Query39 (1864.8466,1+) : <no title>
Query40 (1874.8480,1+) : <no title>
Query41 (1880.8608,1+) : <no title>
Query42 (1896.9047,1+) : <no title>
Query43 (1901.8647,1+) : <no title>
Query44 (1907.8650,1+) : <no title>
Query45 (1910.8745,1+) : <no title>
Query46 (1947.9768,1+) : <no title>
Query47 (2002.9919,1+) : <no title>
Query48 (2044.9976,1+) : <no title>
Query49 (2044.9976,1+) : MaldiWellID: 70018, SpectrumID: 154630,
Query50 (2102.0327,1+) : <no title>
Query51 (2105.0393,1+) : <no title>
Query52 (2181.9829,1+) : <no title>
Query53 (2225.1506,1+) : <no title>
Query54 (2347.1633,1+) : <no title>
Query55 (2407.2917,1+) : <no title>
Query56 (2492.3208,1+) : <no title>
Query57 (2493.3369,1+) : <no title>
Query58 (2541.2495,1+) : <no title>
Query59 (2543.3213,1+) : <no title>
Query60 (2570.2813,1+) : <no title>
Query61 (2587.2981,1+) : <no title>
Query62 (2600.3518,1+) : <no title>
Query63 (2624.4226,1+) : <no title>
Query64 (2672.4282,1+) : <no title>
Query65 (2786.4688,1+) : <no title>
Query66 (2815.5120,1+) : <no title>
Query67 (2981.6201,1+) : <no title>
Query68 (3043.7085,1+) : <no title>
Query69 (3045.7573,1+) : <no title>
Query70 (3281.8982,1+) : <no title>

Mascot: http://www.matrixscience.com/

COVERAGE BAND 1

Mascot Search Results

Protein View

Match to: **gi|329664500** **Score**: 138 Expect: 1.7e-008
pyruvate kinase isozymes M1/M2 [Bos taurus]
Nominal mass (Mr): 58482; Calculated pI value: 7.96
NCBI BLAST search of gi|329664500 against nr
Unformatted sequence string for pasting into other applications
Taxonomy: Bos taurus
Links to retrieve other entries containing this sequence from NCBI Entrez:
gi|146231736 from Bos taurus
gi|296483716 from Bos taurus
Fixed modifications: Carbamidomethyl (C)
Variable modifications: Oxidation (M)
Cleavage by Trypsin: cuts C-term side of KR unless next residue is P
Sequence Coverage: 22%
Matched peptides shown in Bold Red

1 MSKHHSDAGT AFIQTQQLHA AMADTFLEHM CRLDIDSPPI TARNTGIICT
51 IGPASRAVET LKEMIKSGMN VARLNFSHGT HEYHAETIKN VREATESFAS
101 DPILYRPVAV ALDTKGPEIR TGLIKGSGTA EVELKKGATL KITLDNAYME
151 KCDENILWLD YKNICKVVDV GSKIYVDDGL ISLLVKQKGP DFLVTEVENG
201 GSLGSKKGVN LPGAAVDLPA VSEKDIQDLK FGVEQNVDMV FASFIRKASD
251 VHEVRKVLGE KGKNIKIISK IENHEGVRRF DEILEASDGI MVARGDLGIE
301 IPAEKVFLAQ KMMIGRCNRA GKPVICATQM LESMIKKPRP TRAEGSDVAN
351 AVLDGADCIM LSGETAKGDY PLEAVRMQHL IAREAEAAIY HLQLFEELRR
401 LSPITSDPTE AAAVGAVEAS FKCCSGAIIV LTKSGRSAHQ VARYRPRAPI
451 IAVTRNHQTA RQAHLYRGIF PVVCKDPVQE AWAEDVDLRV NLAMNVGKAR
501 GFFKKGDVVI VLTGWRPGSG FTNTMRVVPV P

Show predicted peptides also

Sort Peptides By Residue Number Increasing Mass Decreasing Mass

```
Start - End Observed Mr(expt) Mr(calc) ppm Miss Sequence
93 - 115 2493.3369 2492.3296 2492.2798 20 0 R.EATESFASDPILYRPVAVALDTK.G (No match)
93 - 120 3045.7573 3044.7500 3044.5818 55 1 R.EATESFASDPILYRPVAVALDTKGPEIR.T (No match)
225 - 246 2587.2981 2586.2908 2586.2788 5 1 K.DIQDLKFGVEQNVDMVFASFIR.K Oxidation (M) (No
match)
231 - 246 1858.8521 1857.8448 1857.9084 -34 0 K.FGVEQNVDMVFASFIR.K (No match)
231 - 246 1858.8521 1857.8448 1857.9084 -34 0 K.FGVEQNVDMVFASFIR.K (Ions score 80)
231 - 246 1874.8480 1873.8407 1873.9033 -33 0 K.FGVEQNVDMVFASFIR.K Oxidation (M) (No match)
231 - 247 2002.9919 2001.9846 2001.9982 -7 1 K.FGVEQNVDMVFASFIRK.A Oxidation (M) (No match)
279 - 294 1821.8394 1820.8321 1820.9091 -42 1 R.RFDEILEASDGIMVAR.G (No match)
279 - 294 1837.8445 1836.8372 1836.9040 -36 1 R.RFDEILEASDGIMVAR.G Oxidation (M) (No match)
462 - 475 1687.8110 1686.8037 1686.9028 -59 1 R.QAHLYRGIFPVVCK.D (No match)
468 - 489 2543.3213 2542.3140 2542.2526 24 1 R.GIFPVVCKDPVQEAWAEDVDLR.V (No match)
476 - 489 1642.6763 1641.6690 1641.7635 -58 0 K.DPVQEAWAEDVDLR.V (No match)
505 - 526 2407.2917 2406.2844 2406.2478 15 1 K.KGDVVIVLTGWRPGSGFTNTMR.V Oxidation (M) (No
match)
```

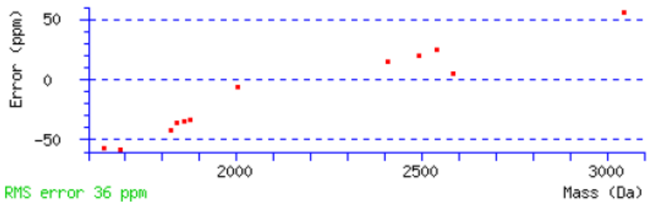

Mascot: http://www.matrixscience.com/

BAND 2

Mascot Search Results

```
User :
Email :
Search title : SampleSetID: 824, AnalysisID: 7242, MaldiWellID: 69623, SpectrumID: 154548, Path=\180719\MS\18-106 NCBI
Mammalia
Database : NCBInr 20120508 (17919084 sequences; 6150218869 residues)
Taxonomy : Mammalia (mammals) (1061927 sequences)
Timestamp : 19 Jul 2018 at 12:46:38 GMT
Top Score : 118 for Mixture 1, gi|156120479 + gi|77736349
```

Mascot Score Histogram

Protein score is -10*Log(P), where P is the probability that the observed match is a random event.
Protein scores greater than 73 are significant (p<0.05).

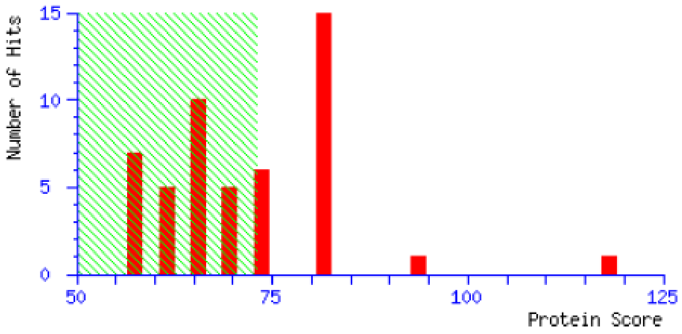

Protein Summary Report

Format As Protein Summary Help

Significance threshold p< 0.05 Max. number of hits 20

Re-Search All Search Unmatched

Index

Accession Mass Score Description
1. Mixture 1 118 gi|156120479 + gi|77736349
2. gi|156120479 39925 92 fructose-bisphosphate aldolase A [Bos taurus]
3. gi|192988474 37434 83 Chain A, Crystal Structure Of A Rabbit Muscle Fructose-1,6- Bisphosphate Aldolase A Dimer Variant
4. gi|77736349 47409 83 beta-enolase [Bos taurus]
5. gi|194219069 39889 81 PREDICTED: fructose-bisphosphate aldolase A isoform 1 [Equus caballus]
6. gi|4557976 39720 80 Chain A, Human Muscle Fructose 1,6-Bisphosphate Aldolase Complexed With Fructose 1,6-Bisphosphate
7. gi|2781027 39647 80 Chain A, Fructose 1,6-Bisphosphate Aldolase From Rabbit Muscle
8. gi|13096351 39646 80 Chain A, Fructose 1,6-Bisphosphate Aldolase From Rabbit Muscle
9. gi|13096347 39636 80 Chain A, Fructose 1,6-Bisphosphate Aldolase From Rabbit Muscle
10. gi|6730618 39586 80 Chain A, Rabbit Muscle Aldolase AFRUCTOSE-1,6-Bisphosphate Complex
11. gi|67464529 39643 80 Chain A, Fructose-1,6-Bisphosphate Aldolase From Rabbit Muscle
12. gi|158430448 39646 80 Chain A, Dihydroxyacetone Phosphate Schiff Base Intermediate In Mutant Fructose-1,6-Bisphosphate Aldolase From Rabbit Muscle
13. gi|228311978 39642 80 Chain A, D33n Mutant Fructose-1,6-Bisphosphate Aldolase From Rabbit Muscle
14. gi|228311995 39615 80 Chain A, D33s Mutant Fructose-1,6-Bisphosphate Aldolase From Rabbit Muscle
15. gi|126722869 39774 80 fructose-bisphosphate aldolase A [Oryctolagus cuniculus]
16. gi|4557305 39851 80 fructose-bisphosphate aldolase A isoform 1 [Homo sapiens]
17. gi|90078570 39860 80 unnamed protein product [Macaca fascicularis]
18. gi|338712747 45488 76 PREDICTED: fructose-bisphosphate aldolase A isoform 4 [Equus caballus]
19. gi|342187211 45688 75 fructose-bisphosphate aldolase A isoform 2 [Homo sapiens]
20. gi|332265962 45656 75 PREDICTED: fructose-bisphosphate aldolase A isoform 4 [Nomascus leucogenys]

Results List

1. Mixture 1 Total score: 118 Expect: 1.7e-006 Matches: 25
Components: 1. gi|156120479 fructose-bisphosphate aldolase A [Bos taurus]
2. gi|77736349 beta-enolase [Bos taurus]
Observed Mr(expt) Mr(calc) ppm Start End Miss Comp Peptide
1093.5521 1092.5448 1092.5563 -10.54 323 - 331 1 1 K.AAQEEYVKR.A
1231.6282 1230.6209 1230.6720 -41.54 121 - 132 1 2 K.AGAAEKGVPLYR.H
1342.7113 1341.7040 1341.7041 -0.06 88 - 99 0 1 K.ADDGRPFPQVIK.A
1475.7905 1474.7832 1474.7780 3.56 413 - 426 1 2 R.IEEALGDKAVFAGR.K
1556.7980 1555.7907 1555.7705 13.0 240 - 253 0 2 K.VVIGMDVAASEFYR.N
1608.7802 1607.7729 1607.7579 9.31 349 - 364 0 1 K.AGAAASESLFISNHAY.-
1671.8528 1670.8455 1670.8702 -14.75 202 - 215 1 1 R.CQYVTEKVLAAVYK.A
1691.8510 1690.8437 1690.8348 5.26 244 - 258 0 1 K.YSHEEIAMATVTALR.R
1804.9508 1803.9435 1803.9366 3.81 33 - 50 0 2 R.AAVPSGASTGIYEALELR.D
1808.9581 1807.9508 1807.9443 3.59 290 - 304 0 1 K.CPLLKPWALTFSYGR.A
1847.9513 1846.9440 1846.9359 4.38 244 - 259 1 1 K.YSHEEIAMATVTALRR.T
1896.9766 1895.9693 1895.9637 2.94 163 - 179 0 2 K.LAMQEFMILPVGASSFR.E
1912.9733 1911.9660 1911.9587 3.85 163 - 179 0 2 K.LAMQEFMILPVGASSFR.E + Oxidation (M)
1928.9700 1927.9627 1927.9536 4.74 163 - 179 0 2 K.LAMQEFMILPVGASSFR.E + 2 Oxidation (M)
2088.0723 2087.0650 2087.0874 -10.71 70 - 87 0 1 R.VNPCIGGVILFHETLYQK.A
2123.0967 2122.0894 2122.0840 2.53 154 - 173 0 1 K.IGEHTPSSLAIMENANVLAR.Y
2139.0969 2138.0896 2138.0790 4.99 154 - 173 0 1 K.IGEHTPSSLAIMENANVLAR.Y + Oxidation (M)
2353.1675 2352.1602 2352.1519 3.53 373 - 394 0 2 R.SGETEDTFIADLVVGLCTGQIK.T
2672.3284 2671.3211 2671.3316 -3.91 229 - 253 1 2 K.TAIQAAGYPDKVVIGMDVAASEFYR.N
2688.3247 2687.3174 2687.3265 -3.37 229 - 253 1 2 K.TAIQAAGYPDKVVIGMDVAASEFYR.N + Oxidation (M)
2743.3467 2742.3394 2742.3348 1.69 203 - 228 0 2 K.DATNVGDEGGFAPNILENNEALELLK.T
3035.6221 3034.6148 3034.5988 5.27 133 - 162 0 2 R.HIADLAGNPELILPVPAFNVINGGSHAGNK.L
3091.5088 3090.5015 3090.5145 -4.21 200 - 228 1 2 K.YGKDATNVGDEGGFAPNILENNEALELLK.T
3113.6228 3112.6155 3112.6379 -7.20 61 - 87 1 1 R.QLLLTADDRVNPCIGGVILFHETLYQK.A
3176.5583 3175.5510 3175.5972 -14.54 174 - 201 1 1 R.YASICQQNGIVPIVEPEILPDGDHDLKR.C
No match to: 1157.5472, 1269.6932, 1456.7847, 1473.8116, 1507.7166, 1542.7548, 1609.7577, 1615.8827, 1634.8130, 1640.7159, 1643.8223, 1703.9562,
1719.9460, 1763.8135, 1769.0023, 1790.9148, 1791.9279, 1821.9209, 1832.9840, 1837.9141, 1848.9746, 1858.9263, 1864.9580, 1874.9208, 1994.9536,
2051.0608, 2072.0630, 2105.0698, 2347.1169, 2493.2927, 2501.2639, 2543.2542, 2595.1992, 2618.3757, 2624.3384, 2927.3965, 2943.3967, 3036.5813,
3045.5872, 3153.4709
2. gi|156120479 Mass: 39925 Score: 92 Expect: 0.00061 Matches: 12
fructose-bisphosphate aldolase A [Bos taurus]
Observed Mr(expt) Mr(calc) ppm Start End Miss Peptide
1093.5521 1092.5448 1092.5563 -10.54 323 - 331 1 K.AAQEEYVKR.A
1342.7113 1341.7040 1341.7041 -0.06 88 - 99 0 K.ADDGRPFPQVIK.A
1608.7802 1607.7729 1607.7579 9.31 349 - 364 0 K.AGAAASESLFISNHAY.-
1671.8528 1670.8455 1670.8702 -14.75 202 - 215 1 R.CQYVTEKVLAAVYK.A
1691.8510 1690.8437 1690.8348 5.26 244 - 258 0 K.YSHEEIAMATVTALR.R
1808.9581 1807.9508 1807.9443 3.59 290 - 304 0 K.CPLLKPWALTFSYGR.A
1847.9513 1846.9440 1846.9359 4.38 244 - 259 1 K.YSHEEIAMATVTALRR.T
2088.0723 2087.0650 2087.0874 -10.71 70 - 87 0 R.VNPCIGGVILFHETLYQK.A
2123.0967 2122.0894 2122.0840 2.53 154 - 173 0 K.IGEHTPSSLAIMENANVLAR.Y
2139.0969 2138.0896 2138.0790 4.99 154 - 173 0 K.IGEHTPSSLAIMENANVLAR.Y + Oxidation (M)
3113.6228 3112.6155 3112.6379 -7.20 61 - 87 1 R.QLLLTADDRVNPCIGGVILFHETLYQK.A
3176.5583 3175.5510 3175.5972 -14.54 174 - 201 1 R.YASICQQNGIVPIVEPEILPDGDHDLKR.C
No match to: 1157.5472, 1231.6282, 1269.6932, 1456.7847, 1473.8116, 1475.7905, 1507.7166, 1542.7548, 1556.7980, 1609.7577, 1615.8827, 1634.8130,
1640.7159, 1643.8223, 1703.9562, 1719.9460, 1763.8135, 1769.0023, 1790.9148, 1791.9279, 1804.9508, 1821.9209, 1832.9840, 1837.9141, 1848.9746,
1858.9263, 1864.9580, 1874.9208, 1896.9766, 1912.9733, 1928.9700, 1994.9536, 2051.0608, 2072.0630, 2105.0698, 2347.1169, 2353.1675, 2493.2927,
2501.2639, 2543.2542, 2595.1992, 2618.3757, 2624.3384, 2672.3284, 2688.3247, 2743.3467, 2927.3965, 2943.3967, 3035.6221, 3036.5813, 3045.5872,

3091.5088, 3153.4709
3. gi|192988474 Mass: 37434 Score: 83 Expect: 0.0056 Matches: 10
Chain A, Crystal Structure Of A Rabbit Muscle Fructose-1,6- Bisphosphate Aldolase A Dimer Variant
Observed Mr(expt) Mr(calc) ppm Start End Miss Peptide
1093.5521 1092.5448 1092.5563 -10.54 319 - 327 1 K.AAQEEYVKR.A
1342.7113 1341.7040 1341.7041 -0.06 84 - 95 0 K.ADDGRPFPQVIK.S
1671.8528 1670.8455 1670.8702 -14.75 198 - 211 1 R.CQYVTEKVLAAVYK.A
1691.8510 1690.8437 1690.8348 5.26 240 - 254 0 K.YSHEEIAMATVTALR.R
1808.9581 1807.9508 1807.9443 3.59 286 - 300 0 K.CPLLKPWALTFSYGR.A
1847.9513 1846.9440 1846.9359 4.38 240 - 255 1 K.YSHEEIAMATVTALRR.T
2088.0723 2087.0650 2087.0874 -10.71 66 - 83 0 R.VNPCIGGVILFHETLYQK.A
2123.0967 2122.0894 2122.0840 2.53 150 - 169 0 K.IGEHTPSALAIMENANVLAR.Y + Oxidation (M)
3113.6228 3112.6155 3112.6379 -7.20 57 - 83 1 R.QLLLTADDRVNPCIGGVILFHETLYQK.A
3176.5583 3175.5510 3175.5972 -14.54 170 - 197 1 R.YASICQQNGIVPIVEPEILPDGDHDLKR.C
No match to: 1157.5472, 1231.6282, 1269.6932, 1456.7847, 1473.8116, 1475.7905, 1507.7166, 1542.7548, 1556.7980, 1608.7802, 1609.7577, 1615.8827,
1634.8130, 1640.7159, 1643.8223, 1703.9562, 1719.9460, 1763.8135, 1769.0023, 1790.9148, 1791.9279, 1804.9508, 1821.9209, 1832.9840, 1837.9141,
1848.9746, 1858.9263, 1864.9580, 1874.9208, 1896.9766, 1912.9733, 1928.9700, 1994.9536, 2051.0608, 2072.0630, 2105.0698, 2139.0969, 2347.1169,
2353.1675, 2493.2927, 2501.2639, 2543.2542, 2595.1992, 2618.3757, 2624.3384, 2672.3284, 2688.3247, 2743.3467, 2927.3965, 2943.3967, 3035.6221,
3036.5813, 3045.5872, 3091.5088, 3153.4709
4. gi|77736349 Mass: 47409 Score: 83 Expect: 0.0058 Matches: 13
beta-enolase [Bos taurus]
Observed Mr(expt) Mr(calc) ppm Start End Miss Peptide
1231.6282 1230.6209 1230.6720 -41.54 121 - 132 1 K.AGAAEKGVPLYR.H
1475.7905 1474.7832 1474.7780 3.56 413 - 426 1 R.IEEALGDKAVFAGR.K
1556.7980 1555.7907 1555.7705 13.0 240 - 253 0 K.VVIGMDVAASEFYR.N
1804.9508 1803.9435 1803.9366 3.81 33 - 50 0 R.AAVPSGASTGIYEALELR.D
1896.9766 1895.9693 1895.9637 2.94 163 - 179 0 K.LAMQEFMILPVGASSFR.E
1912.9733 1911.9660 1911.9587 3.85 163 - 179 0 K.LAMQEFMILPVGASSFR.E + Oxidation (M)
1928.9700 1927.9627 1927.9536 4.74 163 - 179 0 K.LAMQEFMILPVGASSFR.E + 2 Oxidation (M)
2353.1675 2352.1602 2352.1519 3.53 373 - 394 0 R.SGETEDTFIADLVVGLCTGQIK.T
2672.3284 2671.3211 2671.3316 -3.91 229 - 253 1 K.TAIQAAGYPDKVVIGMDVAASEFYR.N
2688.3247 2687.3174 2687.3265 -3.37 229 - 253 1 K.TAIQAAGYPDKVVIGMDVAASEFYR.N + Oxidation (M)
2743.3467 2742.3394 2742.3348 1.69 203 - 228 0 K.DATNVGDEGGFAPNILENNEALELLK.T
3035.6221 3034.6148 3034.5988 5.27 133 - 162 0 R.HIADLAGNPELILPVPAFNVINGGSHAGNK.L
3091.5088 3090.5015 3090.5145 -4.21 200 - 228 1 K.YGKDATNVGDEGGFAPNILENNEALELLK.T
No match to: 1093.5521, 1157.5472, 1269.6932, 1342.7113, 1456.7847, 1473.8116, 1507.7166, 1542.7548, 1608.7802, 1609.7577, 1615.8827, 1634.8130,
1640.7159, 1643.8223, 1671.8528, 1691.8510, 1703.9562, 1719.9460, 1763.8135, 1769.0023, 1790.9148, 1791.9279, 1808.9581, 1821.9209, 1832.9840,
1837.9141, 1847.9513, 1848.9746, 1858.9263, 1864.9580, 1874.9208, 1994.9536, 2051.0608, 2072.0630, 2088.0723, 2105.0698, 2123.0967, 2139.0969,
2347.1169, 2493.2927, 2501.2639, 2543.2542, 2595.1992, 2618.3757, 2624.3384, 2927.3965, 2943.3967, 3036.5813, 3045.5872, 3113.6228, 3153.4709,
3176.5583
5. gi|194219069 Mass: 39889 Score: 81 Expect: 0.0086 Matches: 11
PREDICTED: fructose-bisphosphate aldolase A isoform 1 [Equus caballus]
Observed Mr(expt) Mr(calc) ppm Start End Miss Peptide
1093.5521 1092.5448 1092.5563 -10.54 323 - 331 1 K.AAQEEYVKR.A
1342.7113 1341.7040 1341.7041 -0.06 88 - 99 0 K.ADDGRPFPQVIK.S
1671.8528 1670.8455 1670.8702 -14.75 202 - 215 1 R.CQYVTEKVLAAVYK.A
1691.8510 1690.8437 1690.8348 5.26 244 - 258 0 K.YSHEEIAMATVTALR.R
1808.9581 1807.9508 1807.9443 3.59 290 - 304 0 K.CPLLKPWALTFSYGR.A
1847.9513 1846.9440 1846.9359 4.38 244 - 259 1 K.YSHEEIAMATVTALRR.T
2088.0723 2087.0650 2087.0874 -10.71 70 - 87 0 R.VNPCIGGVILFHETLYQK.A
2123.0967 2122.0894 2122.0840 2.53 154 - 173 0 K.IGEHTPSSLAIMENANVLAR.Y
2139.0969 2138.0896 2138.0790 4.99 154 - 173 0 K.IGEHTPSSLAIMENANVLAR.Y + Oxidation (M)
3113.6228 3112.6155 3112.6379 -7.20 61 - 87 1 R.QLLLTADDRVNPCIGGVILFHETLYQK.A
3176.5583 3175.5510 3175.5972 -14.54 174 - 201 1 R.YASICQQNGIVPIVEPEILPDGDHDLKR.C
No match to: 1157.5472, 1231.6282, 1269.6932, 1456.7847, 1473.8116, 1475.7905, 1507.7166, 1542.7548, 1556.7980, 1608.7802, 1609.7577, 1615.8827,
1634.8130, 1640.7159, 1643.8223, 1703.9562, 1719.9460, 1763.8135, 1769.0023, 1790.9148, 1791.9279, 1804.9508, 1821.9209, 1832.9840, 1837.9141,
1848.9746, 1858.9263, 1864.9580, 1874.9208, 1896.9766, 1912.9733, 1928.9700, 1994.9536, 2051.0608, 2072.0630, 2105.0698, 2347.1169, 2353.1675,
2493.2927, 2501.2639, 2543.2542, 2595.1992, 2618.3757, 2624.3384, 2672.3284, 2688.3247, 2743.3467, 2927.3965, 2943.3967, 3035.6221, 3036.5813,
3045.5872, 3091.5088, 3153.4709
6. gi|4557976 Mass: 39720 Score: 80 Expect: 0.0099 Matches: 10
Chain A, Human Muscle Fructose 1,6-Bisphosphate Aldolase Complexed With Fructose 1,6-Bisphosphate
Observed Mr(expt) Mr(calc) ppm Start End Miss Peptide
1093.5521 1092.5448 1092.5563 -10.54 322 - 330 1 K.AAQEEYVKR.A
1342.7113 1341.7040 1341.7041 -0.06 87 - 98 0 K.ADDGRPFPQVIK.S
1671.8528 1670.8455 1670.8702 -14.75 201 - 214 1 R.CQYVTEKVLAAVYK.A
1691.8510 1690.8437 1690.8348 5.25 243 - 257 0 K.FSHEEIAMATVTALR.R + Oxidation (M)
1808.9581 1807.9508 1807.9443 3.59 289 - 303 0 K.CPLLKPWALTFSYGR.A
1847.9513 1846.9440 1846.9359 4.37 243 - 258 1 K.FSHEEIAMATVTALRR.T + Oxidation (M)
2088.0723 2087.0650 2087.0874 -10.71 69 - 86 0 R.VNPCIGGVILFHETLYQK.A
2123.0967 2122.0894 2122.0840 2.53 153 - 172 0 K.IGEHTPSALAIMENANVLAR.Y + Oxidation (M)
3113.6228 3112.6155 3112.6379 -7.20 60 - 86 1 R.QLLLTADDRVNPCIGGVILFHETLYQK.A
3176.5583 3175.5510 3175.5972 -14.54 173 - 200 1 R.YASICQQNGIVPIVEPEILPDGDHDLKR.C

No match to: 1157.5472, 1231.6282, 1269.6932, 1456.7847, 1473.8116, 1475.7905, 1507.7166, 1542.7548, 1556.7980, 1608.7802, 1609.7577, 1615.8827,
1634.8130, 1640.7159, 1643.8223, 1703.9562, 1719.9460, 1763.8135, 1769.0023, 1790.9148, 1791.9279, 1804.9508, 1821.9209, 1832.9840, 1837.9141,
1848.9746, 1858.9263, 1864.9580, 1874.9208, 1896.9766, 1912.9733, 1928.9700, 1994.9536, 2051.0608, 2072.0630, 2105.0698, 2139.0969, 2347.1169,
2353.1675, 2493.2927, 2501.2639, 2543.2542, 2595.1992, 2618.3757, 2624.3384, 2672.3284, 2688.3247, 2743.3467, 2927.3965, 2943.3967, 3035.6221,
3036.5813, 3045.5872, 3091.5088, 3153.4709
7. gi|2781027 Mass: 39647 Score: 80 Expect: 0.0099 Matches: 10
Chain A, Fructose 1,6-Bisphosphate Aldolase From Rabbit Muscle
Observed Mr(expt) Mr(calc) ppm Start End Miss Peptide
1093.5521 1092.5448 1092.5563 -10.54 322 - 330 1 K.AAQEEYVKR.A
1342.7113 1341.7040 1341.7041 -0.06 87 - 98 0 K.ADDGRPFPQVIK.S
1671.8528 1670.8455 1670.8702 -14.75 201 - 214 1 R.CQYVTEKVLAAVYK.A
1691.8510 1690.8437 1690.8348 5.26 243 - 257 0 K.YSHEEIAMATVTALR.R
1808.9581 1807.9508 1807.9443 3.59 289 - 303 0 K.CPLLKPWALTFSYGR.A
1847.9513 1846.9440 1846.9359 4.38 243 - 258 1 K.YSHEEIAMATVTALRR.T
2088.0723 2087.0650 2087.0874 -10.71 69 - 86 0 R.VNPCIGGVILFHETLYQK.A
2123.0967 2122.0894 2122.0840 2.53 153 - 172 0 K.IGEHTPSALAIMENANVLAR.Y + Oxidation (M)
3113.6228 3112.6155 3112.6379 -7.20 60 - 86 1 R.QLLLTADDRVNPCIGGVILFHETLYQK.A
3176.5583 3175.5510 3175.5972 -14.54 173 - 200 1 R.YASICQQNGIVPIVEPEILPDGDHDLKR.C
No match to: 1157.5472, 1231.6282, 1269.6932, 1456.7847, 1473.8116, 1475.7905, 1507.7166, 1542.7548, 1556.7980, 1608.7802, 1609.7577, 1615.8827,
1634.8130, 1640.7159, 1643.8223, 1703.9562, 1719.9460, 1763.8135, 1769.0023, 1790.9148, 1791.9279, 1804.9508, 1821.9209, 1832.9840, 1837.9141,
1848.9746, 1858.9263, 1864.9580, 1874.9208, 1896.9766, 1912.9733, 1928.9700, 1994.9536, 2051.0608, 2072.0630, 2105.0698, 2139.0969, 2347.1169,
2353.1675, 2493.2927, 2501.2639, 2543.2542, 2595.1992, 2618.3757, 2624.3384, 2672.3284, 2688.3247, 2743.3467, 2927.3965, 2943.3967, 3035.6221,
3036.5813, 3045.5872, 3091.5088, 3153.4709
8. gi|13096351 Mass: 39646 Score: 80 Expect: 0.0099 Matches: 10
Chain A, Fructose 1,6-Bisphosphate Aldolase From Rabbit Muscle
Observed Mr(expt) Mr(calc) ppm Start End Miss Peptide
1093.5521 1092.5448 1092.5563 -10.54 322 - 330 1 K.AAQEEYVKR.A
1342.7113 1341.7040 1341.7041 -0.06 87 - 98 0 K.ADDGRPFPQVIK.S
1671.8528 1670.8455 1670.8702 -14.75 201 - 214 1 R.CQYVTEKVLAAVYK.A
1691.8510 1690.8437 1690.8348 5.26 243 - 257 0 K.YSHEEIAMATVTALR.R
1808.9581 1807.9508 1807.9443 3.59 289 - 303 0 K.CPLLKPWALTFSYGR.A
1847.9513 1846.9440 1846.9359 4.38 243 - 258 1 K.YSHEEIAMATVTALRR.T
2088.0723 2087.0650 2087.0874 -10.71 69 - 86 0 R.VNPCIGGVILFHETLYQK.A
2123.0967 2122.0894 2122.0840 2.53 153 - 172 0 K.IGEHTPSALAIMENANVLAR.Y + Oxidation (M)
3113.6228 3112.6155 3112.6379 -7.20 60 - 86 1 R.QLLLTADDRVNPCIGGVILFHETLYQK.A
3176.5583 3175.5510 3175.5972 -14.54 173 - 200 1 R.YASICQQNGIVPIVEPEILPDGDHDLKR.C
No match to: 1157.5472, 1231.6282, 1269.6932, 1456.7847, 1473.8116, 1475.7905, 1507.7166, 1542.7548, 1556.7980, 1608.7802, 1609.7577, 1615.8827,
1634.8130, 1640.7159, 1643.8223, 1703.9562, 1719.9460, 1763.8135, 1769.0023, 1790.9148, 1791.9279, 1804.9508, 1821.9209, 1832.9840, 1837.9141,
1848.9746, 1858.9263, 1864.9580, 1874.9208, 1896.9766, 1912.9733, 1928.9700, 1994.9536, 2051.0608, 2072.0630, 2105.0698, 2139.0969, 2347.1169,
2353.1675, 2493.2927, 2501.2639, 2543.2542, 2595.1992, 2618.3757, 2624.3384, 2672.3284, 2688.3247, 2743.3467, 2927.3965, 2943.3967, 3035.6221,
3036.5813, 3045.5872, 3091.5088, 3153.4709
9. gi|13096347 Mass: 39636 Score: 80 Expect: 0.0099 Matches: 10
Chain A, Fructose 1,6-Bisphosphate Aldolase From Rabbit Muscle
Observed Mr(expt) Mr(calc) ppm Start End Miss Peptide
1093.5521 1092.5448 1092.5563 -10.54 322 - 330 1 K.AAQEEYVKR.A
1342.7113 1341.7040 1341.7041 -0.06 87 - 98 0 K.ADDGRPFPQVIK.S
1671.8528 1670.8455 1670.8702 -14.75 201 - 214 1 R.CQYVTEKVLAAVYK.A
1691.8510 1690.8437 1690.8348 5.26 243 - 257 0 K.YSHEEIAMATVTALR.R
1808.9581 1807.9508 1807.9443 3.59 289 - 303 0 K.CPLLKPWALTFSYGR.A
1847.9513 1846.9440 1846.9359 4.38 243 - 258 1 K.YSHEEIAMATVTALRR.T
2088.0723 2087.0650 2087.0874 -10.71 69 - 86 0 R.VNPCIGGVILFHETLYQK.A
2123.0967 2122.0894 2122.0840 2.53 153 - 172 0 K.IGEHTPSALAIMENANVLAR.Y + Oxidation (M)
3113.6228 3112.6155 3112.6379 -7.20 60 - 86 1 R.QLLLTADDRVNPCIGGVILFHETLYQK.A
3176.5583 3175.5510 3175.5972 -14.54 173 - 200 1 R.YASICQQNGIVPIVEPEILPDGDHDLKR.C
No match to: 1157.5472, 1231.6282, 1269.6932, 1456.7847, 1473.8116, 1475.7905, 1507.7166, 1542.7548, 1556.7980, 1608.7802, 1609.7577, 1615.8827,
1634.8130, 1640.7159, 1643.8223, 1703.9562, 1719.9460, 1763.8135, 1769.0023, 1790.9148, 1791.9279, 1804.9508, 1821.9209, 1832.9840, 1837.9141,
1848.9746, 1858.9263, 1864.9580, 1874.9208, 1896.9766, 1912.9733, 1928.9700, 1994.9536, 2051.0608, 2072.0630, 2105.0698, 2139.0969, 2347.1169,
2353.1675, 2493.2927, 2501.2639, 2543.2542, 2595.1992, 2618.3757, 2624.3384, 2672.3284, 2688.3247, 2743.3467, 2927.3965, 2943.3967, 3035.6221,
3036.5813, 3045.5872, 3091.5088, 3153.4709
10. gi|6730618 Mass: 39586 Score: 80 Expect: 0.0099 Matches: 10
Chain A, Rabbit Muscle Aldolase AFRUCTOSE-1,6-Bisphosphate Complex
Observed Mr(expt) Mr(calc) ppm Start End Miss Peptide
1093.5521 1092.5448 1092.5563 -10.54 322 - 330 1 K.AAQEEYVKR.A
1342.7113 1341.7040 1341.7041 -0.06 87 - 98 0 K.ADDGRPFPQVIK.S
1671.8528 1670.8455 1670.8702 -14.75 201 - 214 1 R.CQYVTEKVLAAVYK.A
1691.8510 1690.8437 1690.8348 5.26 243 - 257 0 K.YSHEEIAMATVTALR.R
1808.9581 1807.9508 1807.9443 3.59 289 - 303 0 K.CPLLKPWALTFSYGR.A
1847.9513 1846.9440 1846.9359 4.38 243 - 258 1 K.YSHEEIAMATVTALRR.T
2088.0723 2087.0650 2087.0874 -10.71 69 - 86 0 R.VNPCIGGVILFHETLYQK.A

2123.0967 2122.0894 2122.0840 2.53 153 - 172 0 K.IGEHTPSALAIMENANVLAR.Y + Oxidation (M)
3113.6228 3112.6155 3112.6379 -7.20 60 - 86 1 R.QLLLTADDRVNPCIGGVILFHETLYQK.A
3176.5583 3175.5510 3175.5972 -14.54 173 - 200 1 R.YASICQQNGIVPIVEPEILPDGDHDLKR.C
No match to: 1157.5472, 1231.6282, 1269.6932, 1456.7847, 1473.8116, 1475.7905, 1507.7166, 1542.7548, 1556.7980, 1608.7802, 1609.7577, 1615.8827,
1634.8130, 1640.7159, 1643.8223, 1703.9562, 1719.9460, 1763.8135, 1769.0023, 1790.9148, 1791.9279, 1804.9508, 1821.9209, 1832.9840, 1837.9141,
1848.9746, 1858.9263, 1864.9580, 1874.9208, 1896.9766, 1912.9733, 1928.9700, 1994.9536, 2051.0608, 2072.0630, 2105.0698, 2139.0969, 2347.1169,
2353.1675, 2493.2927, 2501.2639, 2543.2542, 2595.1992, 2618.3757, 2624.3384, 2672.3284, 2688.3247, 2743.3467, 2927.3965, 2943.3967, 3035.6221,
3036.5813, 3045.5872, 3091.5088, 3153.4709
11. gi|67464529 Mass: 39643 Score: 80 Expect: 0.0099 Matches: 10
Chain A, Fructose-1,6-Bisphosphate Aldolase From Rabbit Muscle
Observed Mr(expt) Mr(calc) ppm Start End Miss Peptide
1093.5521 1092.5448 1092.5563 -10.54 322 - 330 1 K.AAQEEYVKR.A
1342.7113 1341.7040 1341.7041 -0.06 87 - 98 0 K.ADDGRPFPQVIK.S
1671.8528 1670.8455 1670.8702 -14.75 201 - 214 1 R.CQYVTEKVLAAVYK.A
1691.8510 1690.8437 1690.8348 5.26 243 - 257 0 K.YSHEEIAMATVTALR.R
1808.9581 1807.9508 1807.9443 3.59 289 - 303 0 K.CPLLKPWALTFSYGR.A
1847.9513 1846.9440 1846.9359 4.38 243 - 258 1 K.YSHEEIAMATVTALRR.T
2088.0723 2087.0650 2087.0874 -10.71 69 - 86 0 R.VNPCIGGVILFHETLYQK.A
2123.0967 2122.0894 2122.0840 2.53 153 - 172 0 K.IGEHTPSALAIMENANVLAR.Y + Oxidation (M)
3113.6228 3112.6155 3112.6379 -7.20 60 - 86 1 R.QLLLTADDRVNPCIGGVILFHETLYQK.A
3176.5583 3175.5510 3175.5972 -14.54 173 - 200 1 R.YASICQQNGIVPIVEPEILPDGDHDLKR.C
No match to: 1157.5472, 1231.6282, 1269.6932, 1456.7847, 1473.8116, 1475.7905, 1507.7166, 1542.7548, 1556.7980, 1608.7802, 1609.7577, 1615.8827,
1634.8130, 1640.7159, 1643.8223, 1703.9562, 1719.9460, 1763.8135, 1769.0023, 1790.9148, 1791.9279, 1804.9508, 1821.9209, 1832.9840, 1837.9141,
1848.9746, 1858.9263, 1864.9580, 1874.9208, 1896.9766, 1912.9733, 1928.9700, 1994.9536, 2051.0608, 2072.0630, 2105.0698, 2139.0969, 2347.1169,
2353.1675, 2493.2927, 2501.2639, 2543.2542, 2595.1992, 2618.3757, 2624.3384, 2672.3284, 2688.3247, 2743.3467, 2927.3965, 2943.3967, 3035.6221,
3036.5813, 3045.5872, 3091.5088, 3153.4709
12. gi|158430448 Mass: 39646 Score: 80 Expect: 0.0099 Matches: 10
Chain A, Dihydroxyacetone Phosphate Schiff Base Intermediate In Mutant Fructose-1,6-Bisphosphate Aldolase From Rabbit Muscle
Observed Mr(expt) Mr(calc) ppm Start End Miss Peptide
1093.5521 1092.5448 1092.5563 -10.54 322 - 330 1 K.AAQEEYVKR.A
1342.7113 1341.7040 1341.7041 -0.06 87 - 98 0 K.ADDGRPFPQVIK.S
1671.8528 1670.8455 1670.8702 -14.75 201 - 214 1 R.CQYVTEKVLAAVYK.A
1691.8510 1690.8437 1690.8348 5.26 243 - 257 0 K.YSHEEIAMATVTALR.R
1808.9581 1807.9508 1807.9443 3.59 289 - 303 0 K.CPLLKPWALTFSYGR.A
1847.9513 1846.9440 1846.9359 4.38 243 - 258 1 K.YSHEEIAMATVTALRR.T
2088.0723 2087.0650 2087.0874 -10.71 69 - 86 0 R.VNPCIGGVILFHETLYQK.A
2123.0967 2122.0894 2122.0840 2.53 153 - 172 0 K.IGEHTPSALAIMENANVLAR.Y + Oxidation (M)
3113.6228 3112.6155 3112.6379 -7.20 60 - 86 1 R.QLLLTADDRVNPCIGGVILFHETLYQK.A
3176.5583 3175.5510 3175.5972 -14.54 173 - 200 1 R.YASICQQNGIVPIVEPEILPDGDHDLKR.C
No match to: 1157.5472, 1231.6282, 1269.6932, 1456.7847, 1473.8116, 1475.7905, 1507.7166, 1542.7548, 1556.7980, 1608.7802, 1609.7577, 1615.8827,
1634.8130, 1640.7159, 1643.8223, 1703.9562, 1719.9460, 1763.8135, 1769.0023, 1790.9148, 1791.9279, 1804.9508, 1821.9209, 1832.9840, 1837.9141,
1848.9746, 1858.9263, 1864.9580, 1874.9208, 1896.9766, 1912.9733, 1928.9700, 1994.9536, 2051.0608, 2072.0630, 2105.0698, 2139.0969, 2347.1169,
2353.1675, 2493.2927, 2501.2639, 2543.2542, 2595.1992, 2618.3757, 2624.3384, 2672.3284, 2688.3247, 2743.3467, 2927.3965, 2943.3967, 3035.6221,
3036.5813, 3045.5872, 3091.5088, 3153.4709
13. gi|228311978 Mass: 39642 Score: 80 Expect: 0.0099 Matches: 10
Chain A, D33n Mutant Fructose-1,6-Bisphosphate Aldolase From Rabbit Muscle
Observed Mr(expt) Mr(calc) ppm Start End Miss Peptide
1093.5521 1092.5448 1092.5563 -10.54 322 - 330 1 K.AAQEEYVKR.A
1342.7113 1341.7040 1341.7041 -0.06 87 - 98 0 K.ADDGRPFPQVIK.S
1671.8528 1670.8455 1670.8702 -14.75 201 - 214 1 R.CQYVTEKVLAAVYK.A
1691.8510 1690.8437 1690.8348 5.26 243 - 257 0 K.YSHEEIAMATVTALR.R
1808.9581 1807.9508 1807.9443 3.59 289 - 303 0 K.CPLLKPWALTFSYGR.A
1847.9513 1846.9440 1846.9359 4.38 243 - 258 1 K.YSHEEIAMATVTALRR.T
2088.0723 2087.0650 2087.0874 -10.71 69 - 86 0 R.VNPCIGGVILFHETLYQK.A
2123.0967 2122.0894 2122.0840 2.53 153 - 172 0 K.IGEHTPSALAIMENANVLAR.Y + Oxidation (M)
3113.6228 3112.6155 3112.6379 -7.20 60 - 86 1 R.QLLLTADDRVNPCIGGVILFHETLYQK.A
3176.5583 3175.5510 3175.5972 -14.54 173 - 200 1 R.YASICQQNGIVPIVEPEILPDGDHDLKR.C
No match to: 1157.5472, 1231.6282, 1269.6932, 1456.7847, 1473.8116, 1475.7905, 1507.7166, 1542.7548, 1556.7980, 1608.7802, 1609.7577, 1615.8827,
1634.8130, 1640.7159, 1643.8223, 1703.9562, 1719.9460, 1763.8135, 1769.0023, 1790.9148, 1791.9279, 1804.9508, 1821.9209, 1832.9840, 1837.9141,
1848.9746, 1858.9263, 1864.9580, 1874.9208, 1896.9766, 1912.9733, 1928.9700, 1994.9536, 2051.0608, 2072.0630, 2105.0698, 2139.0969, 2347.1169,
2353.1675, 2493.2927, 2501.2639, 2543.2542, 2595.1992, 2618.3757, 2624.3384, 2672.3284, 2688.3247, 2743.3467, 2927.3965, 2943.3967, 3035.6221,
3036.5813, 3045.5872, 3091.5088, 3153.4709
14. gi|228311995 Mass: 39615 Score: 80 Expect: 0.0099 Matches: 10
Chain A, D33s Mutant Fructose-1,6-Bisphosphate Aldolase From Rabbit Muscle
Observed Mr(expt) Mr(calc) ppm Start End Miss Peptide
1093.5521 1092.5448 1092.5563 -10.54 322 - 330 1 K.AAQEEYVKR.A
1342.7113 1341.7040 1341.7041 -0.06 87 - 98 0 K.ADDGRPFPQVIK.S
1671.8528 1670.8455 1670.8702 -14.75 201 - 214 1 R.CQYVTEKVLAAVYK.A

1691.8510 1690.8437 1690.8348 5.26 243 - 257 0 K.YSHEEIAMATVTALR.R
1808.9581 1807.9508 1807.9443 3.59 289 - 303 0 K.CPLLKPWALTFSYGR.A
1847.9513 1846.9440 1846.9359 4.38 243 - 258 1 K.YSHEEIAMATVTALRR.T
2088.0723 2087.0650 2087.0874 -10.71 69 - 86 0 R.VNPCIGGVILFHETLYQK.A
2123.0967 2122.0894 2122.0840 2.53 153 - 172 0 K.IGEHTPSALAIMENANVLAR.Y + Oxidation (M)
3113.6228 3112.6155 3112.6379 -7.20 60 - 86 1 R.QLLLTADDRVNPCIGGVILFHETLYQK.A
3176.5583 3175.5510 3175.5972 -14.54 173 - 200 1 R.YASICQQNGIVPIVEPEILPDGDHDLKR.C
No match to: 1157.5472, 1231.6282, 1269.6932, 1456.7847, 1473.8116, 1475.7905, 1507.7166, 1542.7548, 1556.7980, 1608.7802, 1609.7577, 1615.8827,
1634.8130, 1640.7159, 1643.8223, 1703.9562, 1719.9460, 1763.8135, 1769.0023, 1790.9148, 1791.9279, 1804.9508, 1821.9209, 1832.9840, 1837.9141,
1848.9746, 1858.9263, 1864.9580, 1874.9208, 1896.9766, 1912.9733, 1928.9700, 1994.9536, 2051.0608, 2072.0630, 2105.0698, 2139.0969, 2347.1169,
2353.1675, 2493.2927, 2501.2639, 2543.2542, 2595.1992, 2618.3757, 2624.3384, 2672.3284, 2688.3247, 2743.3467, 2927.3965, 2943.3967, 3035.6221,
3036.5813, 3045.5872, 3091.5088, 3153.4709
15. gi|126722869 Mass: 39774 Score: 80 Expect: 0.01 Matches: 10
fructose-bisphosphate aldolase A [Oryctolagus cuniculus]
Observed Mr(expt) Mr(calc) ppm Start End Miss Peptide
1093.5521 1092.5448 1092.5563 -10.54 323 - 331 1 K.AAQEEYVKR.A
1342.7113 1341.7040 1341.7041 -0.06 88 - 99 0 K.ADDGRPFPQVIK.S
1671.8528 1670.8455 1670.8702 -14.75 202 - 215 1 R.CQYVTEKVLAAVYK.A
1691.8510 1690.8437 1690.8348 5.26 244 - 258 0 K.YSHEEIAMATVTALR.R
1808.9581 1807.9508 1807.9443 3.59 290 - 304 0 K.CPLLKPWALTFSYGR.A
1847.9513 1846.9440 1846.9359 4.38 244 - 259 1 K.YSHEEIAMATVTALRR.T
2088.0723 2087.0650 2087.0874 -10.71 70 - 87 0 R.VNPCIGGVILFHETLYQK.A
2123.0967 2122.0894 2122.0840 2.53 154 - 173 0 K.IGEHTPSALAIMENANVLAR.Y + Oxidation (M)
3113.6228 3112.6155 3112.6379 -7.20 61 - 87 1 R.QLLLTADDRVNPCIGGVILFHETLYQK.A
3176.5583 3175.5510 3175.5972 -14.54 174 - 201 1 R.YASICQQNGIVPIVEPEILPDGDHDLKR.C
No match to: 1157.5472, 1231.6282, 1269.6932, 1456.7847, 1473.8116, 1475.7905, 1507.7166, 1542.7548, 1556.7980, 1608.7802, 1609.7577, 1615.8827,
1634.8130, 1640.7159, 1643.8223, 1703.9562, 1719.9460, 1763.8135, 1769.0023, 1790.9148, 1791.9279, 1804.9508, 1821.9209, 1832.9840, 1837.9141,
1848.9746, 1858.9263, 1864.9580, 1874.9208, 1896.9766, 1912.9733, 1928.9700, 1994.9536, 2051.0608, 2072.0630, 2105.0698, 2139.0969, 2347.1169,
2353.1675, 2493.2927, 2501.2639, 2543.2542, 2595.1992, 2618.3757, 2624.3384, 2672.3284, 2688.3247, 2743.3467, 2927.3965, 2943.3967, 3035.6221,
3036.5813, 3045.5872, 3091.5088, 3153.4709
16. gi|4557305 Mass: 39851 Score: 80 Expect: 0.01 Matches: 10
fructose-bisphosphate aldolase A isoform 1 [Homo sapiens]
Observed Mr(expt) Mr(calc) ppm Start End Miss Peptide
1093.5521 1092.5448 1092.5563 -10.54 323 - 331 1 K.AAQEEYVKR.A
1342.7113 1341.7040 1341.7041 -0.06 88 - 99 0 K.ADDGRPFPQVIK.S
1671.8528 1670.8455 1670.8702 -14.75 202 - 215 1 R.CQYVTEKVLAAVYK.A
1691.8510 1690.8437 1690.8348 5.25 244 - 258 0 K.FSHEEIAMATVTALR.R + Oxidation (M)
1808.9581 1807.9508 1807.9443 3.59 290 - 304 0 K.CPLLKPWALTFSYGR.A
1847.9513 1846.9440 1846.9359 4.37 244 - 259 1 K.FSHEEIAMATVTALRR.T + Oxidation (M)
2088.0723 2087.0650 2087.0874 -10.71 70 - 87 0 R.VNPCIGGVILFHETLYQK.A
2123.0967 2122.0894 2122.0840 2.53 154 - 173 0 K.IGEHTPSALAIMENANVLAR.Y + Oxidation (M)
3113.6228 3112.6155 3112.6379 -7.20 61 - 87 1 R.QLLLTADDRVNPCIGGVILFHETLYQK.A
3176.5583 3175.5510 3175.5972 -14.54 174 - 201 1 R.YASICQQNGIVPIVEPEILPDGDHDLKR.C
No match to: 1157.5472, 1231.6282, 1269.6932, 1456.7847, 1473.8116, 1475.7905, 1507.7166, 1542.7548, 1556.7980, 1608.7802, 1609.7577, 1615.8827,
1634.8130, 1640.7159, 1643.8223, 1703.9562, 1719.9460, 1763.8135, 1769.0023, 1790.9148, 1791.9279, 1804.9508, 1821.9209, 1832.9840, 1837.9141,
1848.9746, 1858.9263, 1864.9580, 1874.9208, 1896.9766, 1912.9733, 1928.9700, 1994.9536, 2051.0608, 2072.0630, 2105.0698, 2139.0969, 2347.1169,
2353.1675, 2493.2927, 2501.2639, 2543.2542, 2595.1992, 2618.3757, 2624.3384, 2672.3284, 2688.3247, 2743.3467, 2927.3965, 2943.3967, 3035.6221,
3036.5813, 3045.5872, 3091.5088, 3153.4709
17. gi|90078570 Mass: 39860 Score: 80 Expect: 0.01 Matches: 10
unnamed protein product [Macaca fascicularis]
Observed Mr(expt) Mr(calc) ppm Start End Miss Peptide
1093.5521 1092.5448 1092.5563 -10.54 323 - 331 1 K.AAQEEYVKR.A
1342.7113 1341.7040 1341.7041 -0.06 88 - 99 0 K.ADDGRPFPQVIK.S
1671.8528 1670.8455 1670.8702 -14.75 202 - 215 1 R.CQYVTEKVLAAVYK.A
1691.8510 1690.8437 1690.8348 5.25 244 - 258 0 K.FSHEEIAMATVTALR.R + Oxidation (M)
1808.9581 1807.9508 1807.9443 3.59 290 - 304 0 K.CPLLKPWALTFSYGR.A
1847.9513 1846.9440 1846.9359 4.37 244 - 259 1 K.FSHEEIAMATVTALRR.T + Oxidation (M)
2088.0723 2087.0650 2087.0874 -10.71 70 - 87 0 R.VNPCIGGVILFHETLYQK.A
2123.0967 2122.0894 2122.0840 2.53 154 - 173 0 K.IGEHTPSALAIMENANVLAR.Y + Oxidation (M)
3113.6228 3112.6155 3112.6379 -7.20 61 - 87 1 R.QLLLTADDRVNPCIGGVILFHETLYQK.A
3176.5583 3175.5510 3175.5972 -14.54 174 - 201 1 R.YASICQQNGIVPIVEPEILPDGDHDLKR.C
No match to: 1157.5472, 1231.6282, 1269.6932, 1456.7847, 1473.8116, 1475.7905, 1507.7166, 1542.7548, 1556.7980, 1608.7802, 1609.7577, 1615.8827,
1634.8130, 1640.7159, 1643.8223, 1703.9562, 1719.9460, 1763.8135, 1769.0023, 1790.9148, 1791.9279, 1804.9508, 1821.9209, 1832.9840, 1837.9141,
1848.9746, 1858.9263, 1864.9580, 1874.9208, 1896.9766, 1912.9733, 1928.9700, 1994.9536, 2051.0608, 2072.0630, 2105.0698, 2139.0969, 2347.1169,
2353.1675, 2493.2927, 2501.2639, 2543.2542, 2595.1992, 2618.3757, 2624.3384, 2672.3284, 2688.3247, 2743.3467, 2927.3965, 2943.3967, 3035.6221,
3036.5813, 3045.5872, 3091.5088, 3153.4709
18. gi|338712747 Mass: 45488 Score: 76 Expect: 0.03 Matches: 11
PREDICTED: fructose-bisphosphate aldolase A isoform 4 [Equus caballus]
Observed Mr(expt) Mr(calc) ppm Start End Miss Peptide

1093.5521 1092.5448 1092.5563 -10.54 375 - 383 1 K.AAQEEYVKR.A
1342.7113 1341.7040 1341.7041 -0.06 140 - 151 0 K.ADDGRPFPQVIK.S
1671.8528 1670.8455 1670.8702 -14.75 254 - 267 1 R.CQYVTEKVLAAVYK.A
1691.8510 1690.8437 1690.8348 5.26 296 - 310 0 K.YSHEEIAMATVTALR.R
1808.9581 1807.9508 1807.9443 3.59 342 - 356 0 K.CPLLKPWALTFSYGR.A
1847.9513 1846.9440 1846.9359 4.38 296 - 311 1 K.YSHEEIAMATVTALRR.T
2088.0723 2087.0650 2087.0874 -10.71 122 - 139 0 R.VNPCIGGVILFHETLYQK.A
2123.0967 2122.0894 2122.0840 2.53 206 - 225 0 K.IGEHTPSSLAIMENANVLAR.Y
2139.0969 2138.0896 2138.0790 4.99 206 - 225 0 K.IGEHTPSSLAIMENANVLAR.Y + Oxidation (M)
3113.6228 3112.6155 3112.6379 -7.20 113 - 139 1 R.QLLLTADDRVNPCIGGVILFHETLYQK.A
3176.5583 3175.5510 3175.5972 -14.54 226 - 253 1 R.YASICQQNGIVPIVEPEILPDGDHDLKR.C
No match to: 1157.5472, 1231.6282, 1269.6932, 1456.7847, 1473.8116, 1475.7905, 1507.7166, 1542.7548, 1556.7980, 1608.7802, 1609.7577, 1615.8827,
1634.8130, 1640.7159, 1643.8223, 1703.9562, 1719.9460, 1763.8135, 1769.0023, 1790.9148, 1791.9279, 1804.9508, 1821.9209, 1832.9840, 1837.9141,
1848.9746, 1858.9263, 1864.9580, 1874.9208, 1896.9766, 1912.9733, 1928.9700, 1994.9536, 2051.0608, 2072.0630, 2105.0698, 2347.1169, 2353.1675,
2493.2927, 2501.2639, 2543.2542, 2595.1992, 2618.3757, 2624.3384, 2672.3284, 2688.3247, 2743.3467, 2927.3965, 2943.3967, 3035.6221, 3036.5813,
3045.5872, 3091.5088, 3153.4709
19. gi|342187211 Mass: 45688 Score: 75 Expect: 0.036 Matches: 10
fructose-bisphosphate aldolase A isoform 2 [Homo sapiens]
Observed Mr(expt) Mr(calc) ppm Start End Miss Peptide
1093.5521 1092.5448 1092.5563 -10.54 377 - 385 1 K.AAQEEYVKR.A
1342.7113 1341.7040 1341.7041 -0.06 142 - 153 0 K.ADDGRPFPQVIK.S
1671.8528 1670.8455 1670.8702 -14.75 256 - 269 1 R.CQYVTEKVLAAVYK.A
1691.8510 1690.8437 1690.8348 5.25 298 - 312 0 K.FSHEEIAMATVTALR.R + Oxidation (M)
1808.9581 1807.9508 1807.9443 3.59 344 - 358 0 K.CPLLKPWALTFSYGR.A
1847.9513 1846.9440 1846.9359 4.37 298 - 313 1 K.FSHEEIAMATVTALRR.T + Oxidation (M)
2088.0723 2087.0650 2087.0874 -10.71 124 - 141 0 R.VNPCIGGVILFHETLYQK.A
2123.0967 2122.0894 2122.0840 2.53 208 - 227 0 K.IGEHTPSALAIMENANVLAR.Y + Oxidation (M)
3113.6228 3112.6155 3112.6379 -7.20 115 - 141 1 R.QLLLTADDRVNPCIGGVILFHETLYQK.A
3176.5583 3175.5510 3175.5972 -14.54 228 - 255 1 R.YASICQQNGIVPIVEPEILPDGDHDLKR.C
No match to: 1157.5472, 1231.6282, 1269.6932, 1456.7847, 1473.8116, 1475.7905, 1507.7166, 1542.7548, 1556.7980, 1608.7802, 1609.7577, 1615.8827,
1634.8130, 1640.7159, 1643.8223, 1703.9562, 1719.9460, 1763.8135, 1769.0023, 1790.9148, 1791.9279, 1804.9508, 1821.9209, 1832.9840, 1837.9141,
1848.9746, 1858.9263, 1864.9580, 1874.9208, 1896.9766, 1912.9733, 1928.9700, 1994.9536, 2051.0608, 2072.0630, 2105.0698, 2139.0969, 2347.1169,
2353.1675, 2493.2927, 2501.2639, 2543.2542, 2595.1992, 2618.3757, 2624.3384, 2672.3284, 2688.3247, 2743.3467, 2927.3965, 2943.3967, 3035.6221,
3036.5813, 3045.5872, 3091.5088, 3153.4709
20. gi|332265962 Mass: 45656 Score: 75 Expect: 0.036 Matches: 10
PREDICTED: fructose-bisphosphate aldolase A isoform 4 [Nomascus leucogenys]
Observed Mr(expt) Mr(calc) ppm Start End Miss Peptide
1093.5521 1092.5448 1092.5563 -10.54 377 - 385 1 K.AAQEEYVKR.A
1342.7113 1341.7040 1341.7041 -0.06 142 - 153 0 K.ADDGRPFPQVIK.S
1671.8528 1670.8455 1670.8702 -14.75 256 - 269 1 R.CQYVTEKVLAAVYK.A
1691.8510 1690.8437 1690.8348 5.25 298 - 312 0 K.FSHEEIAMATVTALR.R + Oxidation (M)
1808.9581 1807.9508 1807.9443 3.59 344 - 358 0 K.CPLLKPWALTFSYGR.A
1847.9513 1846.9440 1846.9359 4.37 298 - 313 1 K.FSHEEIAMATVTALRR.T + Oxidation (M)
2088.0723 2087.0650 2087.0874 -10.71 124 - 141 0 R.VNPCIGGVILFHETLYQK.A
2123.0967 2122.0894 2122.0840 2.53 208 - 227 0 K.IGEHTPSALAIMENANVLAR.Y + Oxidation (M)
3113.6228 3112.6155 3112.6379 -7.20 115 - 141 1 R.QLLLTADDRVNPCIGGVILFHETLYQK.A
3176.5583 3175.5510 3175.5972 -14.54 228 - 255 1 R.YASICQQNGIVPIVEPEILPDGDHDLKR.C
No match to: 1157.5472, 1231.6282, 1269.6932, 1456.7847, 1473.8116, 1475.7905, 1507.7166, 1542.7548, 1556.7980, 1608.7802, 1609.7577, 1615.8827,
1634.8130, 1640.7159, 1643.8223, 1703.9562, 1719.9460, 1763.8135, 1769.0023, 1790.9148, 1791.9279, 1804.9508, 1821.9209, 1832.9840, 1837.9141,
1848.9746, 1858.9263, 1864.9580, 1874.9208, 1896.9766, 1912.9733, 1928.9700, 1994.9536, 2051.0608, 2072.0630, 2105.0698, 2139.0969, 2347.1169,
2353.1675, 2493.2927, 2501.2639, 2543.2542, 2595.1992, 2618.3757, 2624.3384, 2672.3284, 2688.3247, 2743.3467, 2927.3965, 2943.3967, 3035.6221,
3036.5813, 3045.5872, 3091.5088, 3153.4709

Search Parameters

Type of search : Peptide Mass Fingerprint
Enzyme : Trypsin
Fixed modifications : Carbamidomethyl (C)
Variable modifications : Oxidation (M)
Mass values : Monoisotopic
Protein Mass : Unrestricted
Peptide Mass Tolerance : ± 50 ppm
Peptide Charge State : 1+
Max Missed Cleavages : 1
Number of queries : 65

Mascot: http://www.matrixscience.com/

COVERAGE BAND 2-1

Mascot Search Results

Protein View

Match to: gi|156120479 Score: 92 Expect: 0.00061
fructose-bisphosphate aldolase A [Bos taurus]
Nominal mass (Mr): 39925; Calculated pI value: 8.45
NCBI BLAST search of gi|156120479 against nr
Unformatted sequence string for pasting into other applications
Taxonomy: Bos taurus
Links to retrieve other entries containing this sequence from NCBI Entrez:
gi|151554749 from Bos taurus
gi|296473262 from Bos taurus
Fixed modifications: Carbamidomethyl (C)
Variable modifications: Oxidation (M)
Cleavage by Trypsin: cuts C-term side of KR unless next residue is P
Number of mass values searched: 65
Number of mass values matched: 12
Sequence Coverage: 43%
Matched peptides shown in **Bold Red**

```
1 MPHQYPALTP EQKKELCDIA HRIVAPGKGI LAADESTGSI AKRLQSIGTE
51 NTEENRRFYR QLLLTADDRV NPCIGGVILF HETLYQKADD GRPFPQVIKA
101 KGGVVGIKVD KGVVPLAGTN GETTTQGLDG LSERCAQYKK DGADFAKWRC
151 VLKIGEHTPS SLAIMENANV LARYASICQQ NGIVPIVEPE ILPDGDHDLK
201 RCQYVTEKVL AAVYKALSDH HIYLEGTLLK PNMVTPGHAC TQKYSHEEIA
251 MATVTALRRT VPPAVPGITF LSGGQSEEEA SINLNAINKC PLLKPWALTF
301 SYGRALQASA LKAWGGKKEN LKAAQEEYVK RALANSLACQ GKYTPSGKAG
351 AAASESLFIS NHAY
```

Show predicted peptides also
Sort Peptides By Residue Number Increasing Mass Decreasing Mass

```
Start - End Observed Mr(expt) Mr(calc) ppm Miss Sequence
61 - 87 3113.6228 3112.6155 3112.6379 -7 1 R.QLLLTADDRVNPCIGGVILFHETLYQK.A
70 - 87 2088.0723 2087.0650 2087.0874 -11 0 R.VNPCIGGVILFHETLYQK.A
88 - 99 1342.7113 1341.7040 1341.7041 -0 0 K.ADDGRPFPQVIK.A
154 - 173 2123.0967 2122.0894 2122.0840 3 0 K.IGEHTPSSLAIMENANVLAR.Y
154 - 173 2139.0969 2138.0896 2138.0790 5 0 K.IGEHTPSSLAIMENANVLAR.Y Oxidation (M)
174 - 201 3176.5583 3175.5510 3175.5972 -15 1 R.YASICQQNGIVPIVEPEILPDGDHDLKR.C
202 - 215 1671.8528 1670.8455 1670.8702 -15 1 R.CQYVTEKVLAAVYK.A
244 - 258 1691.8510 1690.8437 1690.8348 5 0 K.YSHEEIAMATVTALR.R
244 - 259 1847.9513 1846.9440 1846.9359 4 1 K.YSHEEIAMATVTALRR.T
290 - 304 1808.9581 1807.9508 1807.9443 4 0 K.CPLLKPWALTFSYGR.A
323 - 331 1093.5521 1092.5448 1092.5563 -11 1 K.AAQEEYVKR.A
349 - 364 1608.7802 1607.7729 1607.7579 9 0 K.AGAAASESLFISNHAY.-
No match to: 1157.5472, 1231.6282, 1269.6932, 1456.7847, 1473.8116, 1475.7905, 1507.7166, 1542.7548,
1556.7980, 1609.7577, 1615.
```

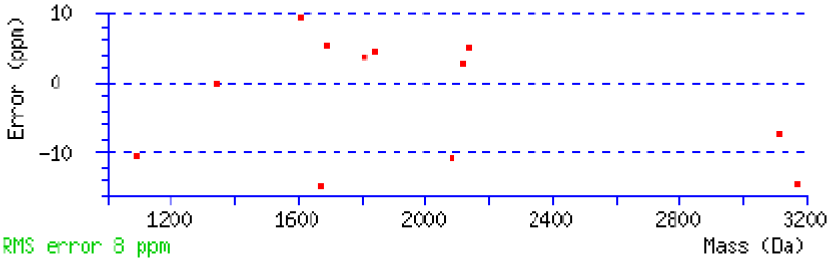

COVERAGE BAND 2-2

Mascot Search Results

Protein View

Match to: gi|77736349 Score: 83 Expect: 0.0058
beta-enolase [Bos taurus]
Nominal mass (Mr): 47409; Calculated pI value: 7.60
NCBI BLAST search of gi|77736349 against nr
Unformatted sequence string for pasting into other applications
Taxonomy: Bos taurus
Links to retrieve other entries containing this sequence from NCBI Entrez:
gi|122140864 from Bos taurus

gi|73587037 from Bos taurus
gi|296476763 from Bos taurus
Fixed modifications: Carbamidomethyl (C)
Variable modifications: Oxidation (M)
Cleavage by Trypsin: cuts C-term side of KR unless next residue is P
Number of mass values searched: 65
Number of mass values matched: 13
Sequence Coverage: 38%
Matched peptides shown in **Bold Red**

```
1 MAMQKIFARE ILDSRGNPTV EVDLHTAKGR FRAAVPSGAS TGIYEALELR
51 DGDKSRYLGK GVLKAVEHIN KTLGPALLEK KLSVVDQEKV DKFMIELDGT
101 ENKSKFGANA ILGVSLAVCK AGAAEKGVPL YRHIADLAGN PELILPVPAF
151 NVINGGSHAG NKLAMQEFMI LPVGASSFRE AMRIGAEVYH HLKGVIKAKY
201 GKDATNVGDE GGFAPNILEN NEALELLKTA IQAAGYPDKV VIGMDVAASE
251 FYRNGKYDLD FKSPDDPARH ISGEKLGELY KNFIKNYPVV SIEDPFDQDD
301 WATWTSFLSG VNIQIVGDDL TVTNPKRIAQ AVEKKACNCL LLKVNQIGSV
351 TESIQACKLA QSNGWGVMVS HRSGETEDTF IADLVVGLCT GQIKTGAPCR
401 SERLAKYNQL MRIEEALGDK AVFAGRKFRN PKAK
```

Show predicted peptides also
Sort Peptides By Residue Number Increasing Mass Decreasing Mass

Start	-	End	Observed	Mr(expt)	Mr(calc)	ppm	Miss	Sequence
33	-	50	1804.9508	1803.9435	1803.9366	4	0	R.AAVPSGASTGIYEALELR.D
121	-	132	1231.6282	1230.6209	1230.6720	-42	1	K.AGAAEKGVPLYR.H
133	-	162	3035.6221	3034.6148	3034.5988	5	0	R.HIADLAGNPELILPVPAFNVINGGSHAGNK.L
163	-	179	1896.9766	1895.9693	1895.9637	3	0	K.LAMQEFMILPVGASSFR.E
163	-	179	1912.9733	1911.9660	1911.9587	4	0	K.LAMQEFMILPVGASSFR.E Oxidation (M)
163	-	179	1928.9700	1927.9627	1927.9536	5	0	K.LAMQEFMILPVGASSFR.E 2 Oxidation (M)
200	-	228	3091.5088	3090.5015	3090.5145	-4	1	K.YGKDATNVGDEGGFAPNILENNEALELLK.T
203	-	228	2743.3467	2742.3394	2742.3348	2	0	K.DATNVGDEGGFAPNILENNEALELLK.T
229	-	253	2672.3284	2671.3211	2671.3316	-4	1	K.TAIQAAGYPDKVVIGMDVAASEFYR.N
229	-	253	2688.3247	2687.3174	2687.3265	-3	1	K.TAIQAAGYPDKVVIGMDVAASEFYR.N Oxidation (M)
240	-	253	1556.7980	1555.7907	1555.7705	13	0	K.VVIGMDVAASEFYR.N
373	-	394	2353.1675	2352.1602	2352.1519	4	0	R.SGETEDTFIADLVVGLCTGQIK.T
413	-	426	1475.7905	1474.7832	1474.7780	4	1	R.IEEALGDKAVFAGR.K

No match to: 1093.5521, 1157.5472, 1269.6932, 1342.7113, 1456.7847, 1473.8116, 1507.7166, 1542.7548, 1608.7802, 1609.7577, 1615.

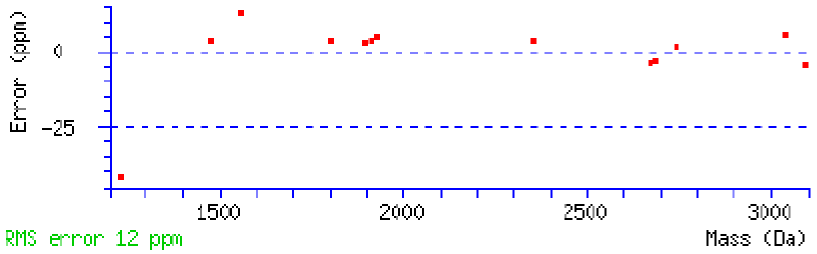

BAND 3

Mascot Search Results

User :
Email :
Search title : SampleSetID: 824, AnalysisID: 7270, MaldiWellID: 69638, SpectrumID: 154836, Path=\180719\MS\18-106 NCBI Mammalia run4
Database : NCBInr 20120508 (17919084 sequences; 6150218869 residues)
Taxonomy : Mammalia (mammals) (1061927 sequences)
Timestamp : 23 Jul 2018 at 17:10:40 GMT
Top Score : 121 for Mixture 1, gi|156120479 + gi|126344225

Mascot Score Histogram

Protein score is -10*Log(P), where P is the probability that the observed match is a random event.
Protein scores greater than 73 are significant ($p<0.05$).

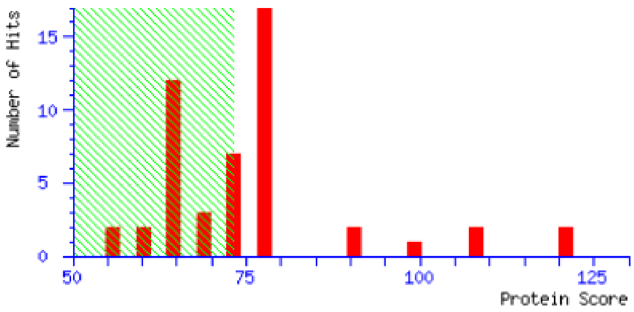

Protein Summary Report

Format As Protein Summary Help

Significance threshold p< 0.05 Max. number of hits 20

Re-Search All Search Unmatched

Index

Accession Mass Score Description

1. Mixture 1 121 gi|156120479 + gi|126344225
2. Mixture 2 120 gi|156120479 + gi|4838363
3. Mixture 3 109 gi|228311978 + gi|126344225
4. Mixture 4 108 gi|228311978 + gi|4838363
5. gi|156120479 39925 101 fructose-bisphosphate aldolase A [Bos taurus]
6. gi|228311978 39642 91 Chain A, D33n Mutant Fructose-1,6-Bisphosphate Aldolase From Rabbit Muscle
7. gi|194219069 39889 90 PREDICTED: fructose-bisphosphate aldolase A isoform 1 [Equus caballus]
8. gi|348584274 39838 79 PREDICTED: fructose-bisphosphate aldolase A-like [Cavia porcellus]
9. gi|4557976 39720 78 Chain A, Human Muscle Fructose 1,6-Bisphosphate Aldolase Complexed With Fructose 1,6-Bisphosphate
10. gi|2781027 39647 78 Chain A, Fructose 1,6-Bisphosphate Aldolase From Rabbit Muscle
11. gi|13096351 39646 78 Chain A, Fructose 1,6-Bisphosphate Aldolase From Rabbit Muscle
12. gi|13096347 39636 78 Chain A, Fructose 1,6-Bisphosphate Aldolase From Rabbit Muscle
13. gi|6730618 39586 78 Chain A, Rabbit Muscle Aldolase AFRUCTOSE-1,6-Bisphosphate Complex
14. gi|67464529 39643 78 Chain A, Fructose-1,6-Bisphosphate Aldolase From Rabbit Muscle
15. gi|160286558 39642 78 Chain A, Fructose 1,6-Bisphosphate Aldolase From Rabbit Muscle
16. gi|158430448 39646 78 Chain A, Dihydroxyacetone Phosphate Schiff Base Intermediate In Mutant Fructose-1,6-Bisphosphate Aldolase From Rabbit Muscle
17. gi|228311995 39615 78 Chain A, D33s Mutant Fructose-1,6-Bisphosphate Aldolase From Rabbit Muscle
18. gi|253722152 39585 78 Chain A, Fructose 1,6-Bisphosphate Aldolase From Rabbit Muscle
19. gi|126722869 39774 78 fructose-bisphosphate aldolase A [Oryctolagus cuniculus]
20. gi|4557305 39851 78 fructose-bisphosphate aldolase A isoform 1 [Homo sapiens]

Results List

1. Mixture 1 Total score: 121 Expect: 8.4e-007 Matches: 24
Components: 1. gi|156120479 fructose-bisphosphate aldolase A [Bos taurus]
2. gi|126344225 PREDICTED: creatine kinase M-type-like [Monodelphis domestica]

Observed Mr(expt) Mr(calc) ppm Start End Miss Comp Peptide
907.5164 906.5091 906.4811 31.0 308 - 314 0 2 K.FEEILTR.L
1007.5800 1006.5727 1006.5923 -19.48 299 - 307 1 2 K.LANLSKHPK.F
1093.6052 1092.5979 1092.5563 38.1 323 - 331 1 1 K.AAQEEYVKR.A
1231.6832 1230.6759 1230.6245 41.8 87 - 96 0 2 K.DLFDPIIQDR.H
1269.7450 1268.7377 1268.6877 39.4 305 - 314 1 2 K.HPKFEEILTR.L
1342.7589 1341.7516 1341.7041 35.4 88 - 99 0 1 K.ADDGRPFPQVIK.A
1408.7764 1407.7691 1407.7146 38.7 2 - 13 0 1 M.PHQYPALTPEQK.K
1507.7773 1506.7700 1506.6951 49.8 117 - 130 0 2 K.GGDDLDPNYVLSSR.V
1515.8118 1514.8045 1514.7664 25.2 136 - 148 1 2 R.SIKGYTLPPHCSR.G
1541.8221 1540.8148 1540.8362 -13.85 88 - 101 1 1 K.ADDGRPFPQVIKAK.G
1643.8777 1642.8704 1642.8103 36.6 224 - 236 0 2 K.SFLVWVNEEDHLR.V
1646.8860 1645.8787 1645.8019 46.7 44 - 57 1 1 R.LQSIGTENTEENRR.F
1671.9034 1670.8961 1670.8702 15.5 202 - 215 1 1 R.CQYVTEKVLAAVYK.A
1691.8939 1690.8866 1690.8348 30.6 244 - 258 0 1 K.YSHEEIAMATVTALR.R
1809.0049 1807.9976 1807.9443 29.5 290 - 304 0 1 K.CPLLKPWALTFSYGR.A
1848.0056 1846.9983 1846.9359 33.8 244 - 259 1 1 K.YSHEEIAMATVTALRR.T
1995.0031 1993.9958 1993.9342 30.9 321 - 341 0 2 R.GTGGVDTAAVGSVFDVSNADR.L
2123.1536 2122.1463 2122.0840 29.3 154 - 173 0 1 K.IGEHTPSSLAIMENANVLAR.Y
2139.1492 2138.1419 2138.0790 29.4 154 - 173 0 1 K.IGEHTPSSLAIMENANVLAR.Y + Oxidation (M)
2272.2007 2271.1934 2271.1343 26.0 112 - 134 0 1 K.GVVPLAGTNGETTTQGLDGLSER.C
2572.3237 2571.3164 2571.2201 37.5 108 - 130 1 2 K.TDLNHENLKGGDDLDPNYVLSSR.V
2608.3215 2607.3142 2607.2619 20.1 216 - 236 1 2 R.GIWHNDNKSFLVWVNEEDHLR.V
2927.4697 2926.4624 2926.4007 21.1 267 - 292 0 2 K.AGHPFMWNEHLGYVLTCPSNLGTGLR.G
3113.6899 3112.6826 3112.6379 14.4 61 - 87 1 1 R.QLLLTADDRVNPCIGGVILFHETLYQK.A

No match to: 1033.6152, 1157.5983, 1198.7487, 1475.8400, 1503.8708, 1542.8124, 1556.8605, 1558.8262, 1609.8159, 1615.9413, 1633.8643, 1634.8557,
1763.8562, 1769.0546, 1784.9873, 1790.9573, 1791.9679, 1797.0793, 1801.0057, 1805.0021, 1833.0280, 1838.9164, 1849.0176, 1862.0222, 1865.0133,
1897.0234, 1913.0203, 1929.0089, 1954.0553, 2075.1692, 2105.1135, 2151.1697, 2333.2825, 2463.3313, 2615.3911, 2624.3879, 2672.3879, 2688.3962,
3036.6570, 3177.6499, 3645.8721

2. Mixture 2 Total score: 120 Expect: 1.1e-006 Matches: 24
Components: 1. gi|156120479 fructose-bisphosphate aldolase A [Bos taurus]
2. gi|4838363 creatine kinase M chain [Bos taurus]

Observed Mr(expt) Mr(calc) ppm Start End Miss Comp Peptide
907.5164 906.5091 906.4811 31.0 308 - 314 0 2 K.FEEILTR.L
1007.5800 1006.5727 1006.5382 34.3 252 - 259 1 2 R.RFCVGLQK.I
1093.6052 1092.5979 1092.5563 38.1 323 - 331 1 1 K.AAQEEYVKR.A
1157.5983 1156.5910 1156.5448 40.0 139 - 148 0 2 K.GYALPPHCSR.G
1231.6832 1230.6759 1230.6245 41.8 87 - 96 0 2 K.DLFDPIIQDR.H
1269.7450 1268.7377 1268.6877 39.4 305 - 314 1 2 K.HPKFEEILTR.L
1342.7589 1341.7516 1341.7041 35.4 88 - 99 0 1 K.ADDGRPFPQVIK.A
1408.7764 1407.7691 1407.7146 38.7 2 - 13 0 1 M.PHQYPALTPEQK.K
1507.7773 1506.7700 1506.7930 -15.22 157 - 170 0 2 K.LSVEALNSLTGEFK.G
1541.8221 1540.8148 1540.8362 -13.85 88 - 101 1 1 K.ADDGRPFPQVIKAK.G
1643.8777 1642.8704 1642.8103 36.6 224 - 236 0 2 K.SFLVWVNEEDHLR.V
1646.8860 1645.8787 1645.8019 46.7 44 - 57 1 1 R.LQSIGTENTEENRR.F
1671.9034 1670.8961 1670.8702 15.5 202 - 215 1 1 R.CQYVTEKVLAAVYK.A
1691.8939 1690.8866 1690.8348 30.6 244 - 258 0 1 K.YSHEEIAMATVTALR.R
1809.0049 1807.9976 1807.9443 29.5 290 - 304 0 1 K.CPLLKPWALTFSYGR.A
1848.0056 1846.9983 1846.9359 33.8 244 - 259 1 1 K.YSHEEIAMATVTALRR.T
1995.0031 1993.9958 1993.9342 30.9 321 - 341 0 2 R.GTGGVDTAAVGSVFDVSNADR.L
2123.1536 2122.1463 2122.0840 29.3 154 - 173 0 1 K.IGEHTPSSLAIMENANVLAR.Y
2139.1492 2138.1419 2138.0790 29.4 154 - 173 0 1 K.IGEHTPSSLAIMENANVLAR.Y + Oxidation (M)
2272.2007 2271.1934 2271.1343 26.0 112 - 134 0 1 K.GVVPLAGTNGETTTQGLDGLSER.C
2572.3237 2571.3164 2571.2201 37.5 108 - 130 1 2 K.TDLNHENLKGGDDLDPNYVLSSR.V
2608.3215 2607.3142 2607.2619 20.1 216 - 236 1 2 R.GIWHNDNKSFLVWVNEEDHLR.V
2927.4697 2926.4624 2926.4007 21.1 267 - 292 0 2 K.AGHPFMWNEHLGYVLTCPSNLGTGLR.G
3113.6899 3112.6826 3112.6379 14.4 61 - 87 1 1 R.QLLLTADDRVNPCIGGVILFHETLYQK.A
No match to: 1033.6152, 1198.7487, 1475.8400, 1503.8708, 1515.8118, 1542.8124, 1556.8605, 1558.8262, 1609.8159, 1615.9413, 1633.8643, 1634.8557,
1763.8562, 1769.0546, 1784.9873, 1790.9573, 1791.9679, 1797.0793, 1801.0057, 1805.0021, 1833.0280, 1838.9164, 1849.0176, 1862.0222, 1865.0133,
1897.0234, 1913.0203, 1929.0089, 1954.0553, 2075.1692, 2105.1135, 2151.1697, 2333.2825, 2463.3313, 2615.3911, 2624.3879, 2672.3879, 2688.3962,
3036.6570, 3177.6499, 3645.8721
3. Mixture 3 Total score: 109 Expect: 1.3e-005 Matches: 22
Components: 1. gi|228311978 Chain A, D33n Mutant Fructose-1,6-Bisphosphate Aldolase From Rabbit Muscle
2. gi|126344225 PREDICTED: creatine kinase M-type-like [Monodelphis domestica]
Observed Mr(expt) Mr(calc) ppm Start End Miss Comp Peptide
907.5164 906.5091 906.4811 31.0 308 - 314 0 2 K.FEEILTR.L
1007.5800 1006.5727 1006.5923 -19.48 299 - 307 1 2 K.LANLSKHPK.F
1093.6052 1092.5979 1092.5563 38.1 322 - 330 1 1 K.AAQEEYVKR.A
1231.6832 1230.6759 1230.6245 41.8 87 - 96 0 2 K.DLFDPIIQDR.H
1269.7450 1268.7377 1268.6877 39.4 305 - 314 1 2 K.HPKFEEILTR.L
1342.7589 1341.7516 1341.7041 35.4 87 - 98 0 1 K.ADDGRPFPQVIK.S
1507.7773 1506.7700 1506.6951 49.8 117 - 130 0 2 K.GGDDLDPNYVLSSR.V
1515.8118 1514.8045 1514.7664 25.2 136 - 148 1 2 R.SIKGYTLPPHCSR.G
1643.8777 1642.8704 1642.8103 36.6 224 - 236 0 2 K.SFLVWVNEEDHLR.V
1646.8860 1645.8787 1645.8019 46.7 43 - 56 1 1 R.LQSIGTENTEENRR.F
1671.9034 1670.8961 1670.8702 15.5 201 - 214 1 1 R.CQYVTEKVLAAVYK.A
1691.8939 1690.8866 1690.8348 30.6 243 - 257 0 1 K.YSHEEIAMATVTALR.R
1809.0049 1807.9976 1807.9443 29.5 289 - 303 0 1 K.CPLLKPWALTFSYGR.A
1848.0056 1846.9983 1846.9359 33.8 243 - 258 1 1 K.YSHEEIAMATVTALRR.T
1897.0234 1896.0161 1896.0680 -27.36 22 - 41 1 1 R.IVAPGKGILAANESTGSIAK.R
1995.0031 1993.9958 1993.9342 30.9 321 - 341 0 2 R.GTGGVDTAAVGSVFDVSNADR.L
2123.1536 2122.1463 2122.0840 29.3 153 - 172 0 1 K.IGEHTPSALAIMENANVLAR.Y + Oxidation (M)
2272.2007 2271.1934 2271.1343 26.0 111 - 133 0 1 K.GVVPLAGTNGETTTQGLDGLSER.C
2572.3237 2571.3164 2571.2201 37.5 108 - 130 1 2 K.TDLNHENLKGGDDLDPNYVLSSR.V
2608.3215 2607.3142 2607.2619 20.1 216 - 236 1 2 R.GIWHNDNKSFLVWVNEEDHLR.V
2927.4697 2926.4624 2926.4007 21.1 267 - 292 0 2 K.AGHPFMWNEHLGYVLTCPSNLGTGLR.G
3113.6899 3112.6826 3112.6379 14.4 60 - 86 1 1 R.QLLLTADDRVNPCIGGVILFHETLYQK.A
No match to: 1033.6152, 1157.5983, 1198.7487, 1408.7764, 1475.8400, 1503.8708, 1541.8221, 1542.8124, 1556.8605, 1558.8262, 1609.8159, 1615.9413,
1633.8643, 1634.8557, 1763.8562, 1769.0546, 1784.9873, 1790.9573, 1791.9679, 1797.0793, 1801.0057, 1805.0021, 1833.0280, 1838.9164, 1849.0176,
1862.0222, 1865.0133, 1913.0203, 1929.0089, 1954.0553, 2075.1692, 2105.1135, 2139.1492, 2151.1697, 2333.2825, 2463.3313, 2615.3911, 2624.3879,
2672.3879, 2688.3962, 3036.6570, 3177.6499, 3645.8721
4. Mixture 4 Total score: 108 Expect: 1.7e-005 Matches: 22
Components: 1. gi|228311978 Chain A, D33n Mutant Fructose-1,6-Bisphosphate Aldolase From Rabbit Muscle
2. gi|4838363 creatine kinase M chain [Bos taurus]
Observed Mr(expt) Mr(calc) ppm Start End Miss Comp Peptide
907.5164 906.5091 906.4811 31.0 308 - 314 0 2 K.FEEILTR.L
1007.5800 1006.5727 1006.5382 34.3 252 - 259 1 2 R.RFCVGLQK.I
1093.6052 1092.5979 1092.5563 38.1 322 - 330 1 1 K.AAQEEYVKR.A
1157.5983 1156.5910 1156.5448 40.0 139 - 148 0 2 K.GYALPPHCSR.G
1231.6832 1230.6759 1230.6245 41.8 87 - 96 0 2 K.DLFDPIIQDR.H
1269.7450 1268.7377 1268.6877 39.4 305 - 314 1 2 K.HPKFEEILTR.L
1342.7589 1341.7516 1341.7041 35.4 87 - 98 0 1 K.ADDGRPFPQVIK.S
1507.7773 1506.7700 1506.7930 -15.22 157 - 170 0 2 K.LSVEALNSLTGEFK.G
1643.8777 1642.8704 1642.8103 36.6 224 - 236 0 2 K.SFLVWVNEEDHLR.V
1646.8860 1645.8787 1645.8019 46.7 43 - 56 1 1 R.LQSIGTENTEENRR.F
1671.9034 1670.8961 1670.8702 15.5 201 - 214 1 1 R.CQYVTEKVLAAVYK.A
1691.8939 1690.8866 1690.8348 30.6 243 - 257 0 1 K.YSHEEIAMATVTALR.R
1809.0049 1807.9976 1807.9443 29.5 289 - 303 0 1 K.CPLLKPWALTFSYGR.A
1848.0056 1846.9983 1846.9359 33.8 243 - 258 1 1 K.YSHEEIAMATVTALRR.T
1897.0234 1896.0161 1896.0680 -27.36 22 - 41 1 1 R.IVAPGKGILAANESTGSIAK.R
1995.0031 1993.9958 1993.9342 30.9 321 - 341 0 2 R.GTGGVDTAAVGSVFDVSNADR.L

2123.1536 2122.1463 2122.0840 29.3 153 - 172 0 1 K.IGEHTPSALAIMENANVLAR.Y + Oxidation (M)
2272.2007 2271.1934 2271.1343 26.0 111 - 133 0 1 K.GVVPLAGTNGETTTQGLDGLSER.C
2572.3237 2571.3164 2571.2201 37.5 108 - 130 1 2 K.TDLNHENLKGGDDLDPNYVLSSR.V
2608.3215 2607.3142 2607.2619 20.1 216 - 236 1 2 R.GIWHNDNKSFLVWVNEEDHLR.V
2927.4697 2926.4624 2926.4007 21.1 267 - 292 0 2 K.AGHPFMWNEHLGYVLTCPSNLGTGLR.G
3113.6899 3112.6826 3112.6379 14.4 60 - 86 1 1 R.QLLLTADDRVNPCIGGVILFHETLYQK.A
No match to: 1033.6152, 1198.7487, 1408.7764, 1475.8400, 1503.8708, 1515.8118, 1541.8221, 1542.8124, 1556.8605, 1558.8262, 1609.8159, 1615.9413,
1633.8643, 1634.8557, 1763.8562, 1769.0546, 1784.9873, 1790.9573, 1791.9679, 1797.0793, 1801.0057, 1805.0021, 1833.0280, 1838.9164, 1849.0176,
1862.0222, 1865.0133, 1913.0203, 1929.0089, 1954.0553, 2075.1692, 2105.1135, 2139.1492, 2151.1697, 2333.2825, 2463.3313, 2615.3911, 2624.3879,
2672.3879, 2688.3962, 3036.6570, 3177.6499, 3645.8721
5. gi|156120479 Mass: 39925 Score: 101 Expect: 8.4e-005 Matches: 13
fructose-bisphosphate aldolase A [Bos taurus]
Observed Mr(expt) Mr(calc) ppm Start End Miss Peptide
1093.6052 1092.5979 1092.5563 38.1 323 - 331 1 K.AAQEEYVKR.A
1342.7589 1341.7516 1341.7041 35.4 88 - 99 0 K.ADDGRPFPQVIK.A
1408.7764 1407.7691 1407.7146 38.7 2 - 13 0 M.PHQYPALTPEQK.K
1541.8221 1540.8148 1540.8362 -13.85 88 - 101 1 K.ADDGRPFPQVIKAK.G
1646.8860 1645.8787 1645.8019 46.7 44 - 57 1 R.LQSIGTENTEENRR.F
1671.9034 1670.8961 1670.8702 15.5 202 - 215 1 R.CQYVTEKVLAAVYK.A
1691.8939 1690.8866 1690.8348 30.6 244 - 258 0 K.YSHEEIAMATVTALR.R
1809.0049 1807.9976 1807.9443 29.5 290 - 304 0 K.CPLLKPWALTFSYGR.A
1848.0056 1846.9983 1846.9359 33.8 244 - 259 1 K.YSHEEIAMATVTALRR.T
2123.1536 2122.1463 2122.0840 29.3 154 - 173 0 K.IGEHTPSSLAIMENANVLAR.Y
2139.1492 2138.1419 2138.0790 29.4 154 - 173 0 K.IGEHTPSSLAIMENANVLAR.Y + Oxidation (M)
2272.2007 2271.1934 2271.1343 26.0 112 - 134 0 K.GVVPLAGTNGETTTQGLDGLSER.C
3113.6899 3112.6826 3112.6379 14.4 61 - 87 1 R.QLLLTADDRVNPCIGGVILFHETLYQK.A
No match to: 907.5164, 1007.5800, 1033.6152, 1157.5983, 1198.7487, 1231.6832, 1269.7450, 1475.8400, 1503.8708, 1507.7773, 1515.8118, 1542.8124,
1556.8605, 1558.8262, 1609.8159, 1615.9413, 1633.8643, 1634.8557, 1643.8777, 1763.8562, 1769.0546, 1784.9873, 1790.9573, 1791.9679, 1797.0793,
1801.0057, 1805.0021, 1833.0280, 1838.9164, 1849.0176, 1862.0222, 1865.0133, 1897.0234, 1913.0203, 1929.0089, 1954.0553, 1995.0031, 2075.1692,
2105.1135, 2151.1697, 2333.2825, 2463.3313, 2572.3237, 2608.3215, 2615.3911, 2624.3879, 2672.3879, 2688.3962, 2927.4697, 3036.6570, 3177.6499,
3645.8721
6. gi|228311978 Mass: 39642 Score: 91 Expect: 0.00088 Matches: 11
Chain A, D33n Mutant Fructose-1,6-Bisphosphate Aldolase From Rabbit Muscle
Observed Mr(expt) Mr(calc) ppm Start End Miss Peptide
1093.6052 1092.5979 1092.5563 38.1 322 - 330 1 K.AAQEEYVKR.A
1342.7589 1341.7516 1341.7041 35.4 87 - 98 0 K.ADDGRPFPQVIK.S
1646.8860 1645.8787 1645.8019 46.7 43 - 56 1 R.LQSIGTENTEENRR.F
1671.9034 1670.8961 1670.8702 15.5 201 - 214 1 R.CQYVTEKVLAAVYK.A
1691.8939 1690.8866 1690.8348 30.6 243 - 257 0 K.YSHEEIAMATVTALR.R
1809.0049 1807.9976 1807.9443 29.5 289 - 303 0 K.CPLLKPWALTFSYGR.A
1848.0056 1846.9983 1846.9359 33.8 243 - 258 1 K.YSHEEIAMATVTALRR.T
1897.0234 1896.0161 1896.0680 -27.36 22 - 41 1 R.IVAPGKGILAANESTGSIAK.R
2123.1536 2122.1463 2122.0840 29.3 153 - 172 0 K.IGEHTPSALAIMENANVLAR.Y + Oxidation (M)
2272.2007 2271.1934 2271.1343 26.0 111 - 133 0 K.GVVPLAGTNGETTTQGLDGLSER.C
3113.6899 3112.6826 3112.6379 14.4 60 - 86 1 R.QLLLTADDRVNPCIGGVILFHETLYQK.A
No match to: 907.5164, 1007.5800, 1033.6152, 1157.5983, 1198.7487, 1231.6832, 1269.7450, 1408.7764, 1475.8400, 1503.8708, 1507.7773, 1515.8118,
1541.8221, 1542.8124, 1556.8605, 1558.8262, 1609.8159, 1615.9413, 1633.8643, 1634.8557, 1643.8777, 1763.8562, 1769.0546, 1784.9873, 1790.9573,
1791.9679, 1797.0793, 1801.0057, 1805.0021, 1833.0280, 1838.9164, 1849.0176, 1862.0222, 1865.0133, 1913.0203, 1929.0089, 1954.0553, 1995.0031,
2075.1692, 2105.1135, 2139.1492, 2151.1697, 2333.2825, 2463.3313, 2572.3237, 2608.3215, 2615.3911, 2624.3879, 2672.3879, 2688.3962, 2927.4697,
3036.6570, 3177.6499, 3645.8721
7. gi|194219069 Mass: 39889 Score: 90 Expect: 0.0011 Matches: 12
PREDICTED: fructose-bisphosphate aldolase A isoform 1 [Equus caballus]
Observed Mr(expt) Mr(calc) ppm Start End Miss Peptide
1093.6052 1092.5979 1092.5563 38.1 323 - 331 1 K.AAQEEYVKR.A
1342.7589 1341.7516 1341.7041 35.4 88 - 99 0 K.ADDGRPFPQVIK.S
1408.7764 1407.7691 1407.7146 38.7 2 - 13 0 M.PHQYPALTPEQK.K
1646.8860 1645.8787 1645.8019 46.7 44 - 57 1 R.LQSIGTENTEENRR.F
1671.9034 1670.8961 1670.8702 15.5 202 - 215 1 R.CQYVTEKVLAAVYK.A
1691.8939 1690.8866 1690.8348 30.6 244 - 258 0 K.YSHEEIAMATVTALR.R
1809.0049 1807.9976 1807.9443 29.5 290 - 304 0 K.CPLLKPWALTFSYGR.A
1848.0056 1846.9983 1846.9359 33.8 244 - 259 1 K.YSHEEIAMATVTALRR.T
2123.1536 2122.1463 2122.0840 29.3 154 - 173 0 K.IGEHTPSSLAIMENANVLAR.Y
2139.1492 2138.1419 2138.0790 29.4 154 - 173 0 K.IGEHTPSSLAIMENANVLAR.Y + Oxidation (M)
2272.2007 2271.1934 2271.1343 26.0 112 - 134 0 K.GVVPLAGTNGETTTQGLDGLSER.C
3113.6899 3112.6826 3112.6379 14.4 61 - 87 1 R.QLLLTADDRVNPCIGGVILFHETLYQK.A
No match to: 907.5164, 1007.5800, 1033.6152, 1157.5983, 1198.7487, 1231.6832, 1269.7450, 1475.8400, 1503.8708, 1507.7773, 1515.8118, 1541.8221,
1542.8124, 1556.8605, 1558.8262, 1609.8159, 1615.9413, 1633.8643, 1634.8557, 1643.8777, 1763.8562, 1769.0546, 1784.9873, 1790.9573, 1791.9679,
1797.0793, 1801.0057, 1805.0021, 1833.0280, 1838.9164, 1849.0176, 1862.0222, 1865.0133, 1897.0234, 1913.0203, 1929.0089, 1954.0553, 1995.0031,
2075.1692, 2105.1135, 2151.1697, 2333.2825, 2463.3313, 2572.3237, 2608.3215, 2615.3911, 2624.3879, 2672.3879, 2688.3962, 2927.4697, 3036.6570,
3177.6499, 3645.8721

8. gi|348584274 Mass: 39838 Score: 79 Expect: 0.014 Matches: 10
PREDICTED: fructose-bisphosphate aldolase A-like [Cavia porcellus]
Observed Mr(expt) Mr(calc) ppm Start End Miss Peptide
1342.7589 1341.7516 1341.7041 35.4 88 - 99 0 K.ADDGRPFPQVIK.S
1408.7764 1407.7691 1407.7146 38.7 2 - 13 0 M.PHQYPALTPEQK.K
1646.8860 1645.8787 1645.8019 46.7 44 - 57 1 R.LQSIGTENTEENRR.F
1671.9034 1670.8961 1670.8702 15.5 202 - 215 1 R.CQYVTEKVLAAVYK.A
1691.8939 1690.8866 1690.8348 30.6 244 - 258 0 K.YSHEEIAMATVTALR.R
1809.0049 1807.9976 1807.9443 29.5 290 - 304 0 K.CPLLKPWALTFSYGR.A
1848.0056 1846.9983 1846.9359 33.8 244 - 259 1 K.YSHEEIAMATVTALRR.T
2123.1536 2122.1463 2122.0840 29.3 154 - 173 0 K.IGEHTPSALAIMENANVLAR.Y + Oxidation (M)
2272.2007 2271.1934 2271.1343 26.0 112 - 134 0 K.GVVPLAGTNGETTTQGLDGLSER.C
3113.6899 3112.6826 3112.6379 14.4 61 - 87 1 R.QLLLTADDRVNPCIGGVILFHETLYQK.A
No match to: 907.5164, 1007.5800, 1033.6152, 1093.6052, 1157.5983, 1198.7487, 1231.6832, 1269.7450, 1475.8400, 1503.8708,
1507.7773, 1515.8118,
1541.8221, 1542.8124, 1556.8605, 1558.8262, 1609.8159, 1615.9413, 1633.8643, 1634.8557, 1643.8777, 1763.8562, 1769.0546,
1784.9873, 1790.9573,
1791.9679, 1797.0793, 1801.0057, 1805.0021, 1833.0280, 1838.9164, 1849.0176, 1862.0222, 1865.0133, 1897.0234, 1913.0203,
1929.0089, 1954.0553,
1995.0031, 2075.1692, 2105.1135, 2139.1492, 2151.1697, 2333.2825, 2463.3313, 2572.3237, 2608.3215, 2615.3911, 2624.3879,
2672.3879, 2688.3962,
2927.4697, 3036.6570, 3177.6499, 3645.8721
9. gi|4557976 Mass: 39720 Score: 78 Expect: 0.015 Matches: 10
Chain A, Human Muscle Fructose 1,6-Bisphosphate Aldolase Complexed With Fructose 1,6-Bisphosphate
Observed Mr(expt) Mr(calc) ppm Start End Miss Peptide
1093.6052 1092.5979 1092.5563 38.1 322 - 330 1 K.AAQEEYVKR.A
1342.7589 1341.7516 1341.7041 35.4 87 - 98 0 K.ADDGRPFPQVIK.S
1646.8860 1645.8787 1645.8019 46.7 43 - 56 1 R.LQSIGTENTEENRR.F
1671.9034 1670.8961 1670.8702 15.5 201 - 214 1 R.CQYVTEKVLAAVYK.A
1691.8939 1690.8866 1690.8348 30.6 243 - 257 0 K.FSHEEIAMATVTALR.R + Oxidation (M)
1809.0049 1807.9976 1807.9443 29.5 289 - 303 0 K.CPLLKPWALTFSYGR.A
1848.0056 1846.9983 1846.9359 33.8 243 - 258 1 K.FSHEEIAMATVTALRR.T + Oxidation (M)
2123.1536 2122.1463 2122.0840 29.3 153 - 172 0 K.IGEHTPSALAIMENANVLAR.Y + Oxidation (M)
2272.2007 2271.1934 2271.1343 26.0 111 - 133 0 K.GVVPLAGTNGETTTQGLDGLSER.C
3113.6899 3112.6826 3112.6379 14.4 60 - 86 1 R.QLLLTADDRVNPCIGGVILFHETLYQK.A
No match to: 907.5164, 1007.5800, 1033.6152, 1157.5983, 1198.7487, 1231.6832, 1269.7450, 1408.7764, 1475.8400, 1503.8708,
1507.7773, 1515.8118,
1541.8221, 1542.8124, 1556.8605, 1558.8262, 1609.8159, 1615.9413, 1633.8643, 1634.8557, 1643.8777, 1763.8562, 1769.0546,
1784.9873, 1790.9573,
1791.9679, 1797.0793, 1801.0057, 1805.0021, 1833.0280, 1838.9164, 1849.0176, 1862.0222, 1865.0133, 1897.0234, 1913.0203,
1929.0089, 1954.0553,
1995.0031, 2075.1692, 2105.1135, 2139.1492, 2151.1697, 2333.2825, 2463.3313, 2572.3237, 2608.3215, 2615.3911, 2624.3879,
2672.3879, 2688.3962,
2927.4697, 3036.6570, 3177.6499, 3645.8721
10. gi|2781027 Mass: 39647 Score: 78 Expect: 0.015 Matches: 10
Chain A, Fructose 1,6-Bisphosphate Aldolase From Rabbit Muscle
Observed Mr(expt) Mr(calc) ppm Start End Miss Peptide
1093.6052 1092.5979 1092.5563 38.1 322 - 330 1 K.AAQEEYVKR.A
1342.7589 1341.7516 1341.7041 35.4 87 - 98 0 K.ADDGRPFPQVIK.S
1646.8860 1645.8787 1645.8019 46.7 43 - 56 1 R.LQSIGTENTEENRR.F
1671.9034 1670.8961 1670.8702 15.5 201 - 214 1 R.CQYVTEKVLAAVYK.A
1691.8939 1690.8866 1690.8348 30.6 243 - 257 0 K.YSHEEIAMATVTALR.R
1809.0049 1807.9976 1807.9443 29.5 289 - 303 0 K.CPLLKPWALTFSYGR.A
1848.0056 1846.9983 1846.9359 33.8 243 - 258 1 K.YSHEEIAMATVTALRR.T
2123.1536 2122.1463 2122.0840 29.3 153 - 172 0 K.IGEHTPSALAIMENANVLAR.Y + Oxidation (M)
2272.2007 2271.1934 2271.1343 26.0 111 - 133 0 K.GVVPLAGTNGETTTQGLDGLSER.C
3113.6899 3112.6826 3112.6379 14.4 60 - 86 1 R.QLLLTADDRVNPCIGGVILFHETLYQK.A
No match to: 907.5164, 1007.5800, 1033.6152, 1157.5983, 1198.7487, 1231.6832, 1269.7450, 1408.7764, 1475.8400, 1503.8708,
1507.7773, 1515.8118,
1541.8221, 1542.8124, 1556.8605, 1558.8262, 1609.8159, 1615.9413, 1633.8643, 1634.8557, 1643.8777, 1763.8562, 1769.0546,
1784.9873, 1790.9573,
1791.9679, 1797.0793, 1801.0057, 1805.0021, 1833.0280, 1838.9164, 1849.0176, 1862.0222, 1865.0133, 1897.0234, 1913.0203,
1929.0089, 1954.0553,
1995.0031, 2075.1692, 2105.1135, 2139.1492, 2151.1697, 2333.2825, 2463.3313, 2572.3237, 2608.3215, 2615.3911, 2624.3879,
2672.3879, 2688.3962,
2927.4697, 3036.6570, 3177.6499, 3645.8721
11. gi|13096351 Mass: 39646 Score: 78 Expect: 0.015 Matches: 10
Chain A, Fructose 1,6-Bisphosphate Aldolase From Rabbit Muscle
Observed Mr(expt) Mr(calc) ppm Start End Miss Peptide
1093.6052 1092.5979 1092.5563 38.1 322 - 330 1 K.AAQEEYVKR.A
1342.7589 1341.7516 1341.7041 35.4 87 - 98 0 K.ADDGRPFPQVIK.S
1646.8860 1645.8787 1645.8019 46.7 43 - 56 1 R.LQSIGTENTEENRR.F
1671.9034 1670.8961 1670.8702 15.5 201 - 214 1 R.CQYVTEKVLAAVYK.A
1691.8939 1690.8866 1690.8348 30.6 243 - 257 0 K.YSHEEIAMATVTALR.R
1809.0049 1807.9976 1807.9443 29.5 289 - 303 0 K.CPLLKPWALTFSYGR.A
1848.0056 1846.9983 1846.9359 33.8 243 - 258 1 K.YSHEEIAMATVTALRR.T
2123.1536 2122.1463 2122.0840 29.3 153 - 172 0 K.IGEHTPSALAIMENANVLAR.Y + Oxidation (M)
2272.2007 2271.1934 2271.1343 26.0 111 - 133 0 K.GVVPLAGTNGETTTQGLDGLSER.C
3113.6899 3112.6826 3112.6379 14.4 60 - 86 1 R.QLLLTADDRVNPCIGGVILFHETLYQK.A
No match to: 907.5164, 1007.5800, 1033.6152, 1157.5983, 1198.7487, 1231.6832, 1269.7450, 1408.7764, 1475.8400, 1503.8708,
1507.7773, 1515.8118,
1541.8221, 1542.8124, 1556.8605, 1558.8262, 1609.8159, 1615.9413, 1633.8643, 1634.8557, 1643.8777, 1763.8562, 1769.0546,
1784.9873, 1790.9573,
1791.9679, 1797.0793, 1801.0057, 1805.0021, 1833.0280, 1838.9164, 1849.0176, 1862.0222, 1865.0133, 1897.0234, 1913.0203,
1929.0089, 1954.0553,

1995.0031, 2075.1692, 2105.1135, 2139.1492, 2151.1697, 2333.2825, 2463.3313, 2572.3237, 2608.3215, 2615.3911, 2624.3879,
2672.3879, 2688.3962,
2927.4697, 3036.6570, 3177.6499, 3645.8721
12. gi|13096347 Mass: 39636 Score: 78 Expect: 0.015 Matches: 10
Chain A, Fructose 1,6-Bisphosphate Aldolase From Rabbit Muscle
Observed Mr(expt) Mr(calc) ppm Start End Miss Peptide
1093.6052 1092.5979 1092.5563 38.1 322 - 330 1 K.AAQEEYVKR.A
1342.7589 1341.7516 1341.7041 35.4 87 - 98 0 K.ADDGRPFPQVIK.S
1646.8860 1645.8787 1645.8019 46.7 43 - 56 1 R.LQSIGTENTEENRR.F
1671.9034 1670.8961 1670.8702 15.5 201 - 214 1 R.CQYVTEKVLAAVYK.A
1691.8939 1690.8866 1690.8348 30.6 243 - 257 0 K.YSHEEIAMATVTALR.R
1809.0049 1807.9976 1807.9443 29.5 289 - 303 0 K.CPLLKPWALTFSYGR.A
1848.0056 1846.9983 1846.9359 33.8 243 - 258 1 K.YSHEEIAMATVTALRR.T
2123.1536 2122.1463 2122.0840 29.3 153 - 172 0 K.IGEHTPSALAIMENANVLAR.Y + Oxidation (M)
2272.2007 2271.1934 2271.1343 26.0 111 - 133 0 K.GVVPLAGTNGETTTQGLDGLSER.C
3113.6899 3112.6826 3112.6379 14.4 60 - 86 1 R.QLLLTADDRVNPCIGGVILFHETLYQK.A
No match to: 907.5164, 1007.5800, 1033.6152, 1157.5983, 1198.7487, 1231.6832, 1269.7450, 1408.7764, 1475.8400, 1503.8708,
1507.7773, 1515.8118,
1541.8221, 1542.8124, 1556.8605, 1558.8262, 1609.8159, 1615.9413, 1633.8643, 1634.8557, 1643.8777, 1763.8562, 1769.0546,
1784.9873, 1790.9573,
1791.9679, 1797.0793, 1801.0057, 1805.0021, 1833.0280, 1838.9164, 1849.0176, 1862.0222, 1865.0133, 1897.0234, 1913.0203,
1929.0089, 1954.0553,
1995.0031, 2075.1692, 2105.1135, 2139.1492, 2151.1697, 2333.2825, 2463.3313, 2572.3237, 2608.3215, 2615.3911, 2624.3879,
2672.3879, 2688.3962,
2927.4697, 3036.6570, 3177.6499, 3645.8721
13. gi|6730618 Mass: 39586 Score: 78 Expect: 0.015 Matches: 10
Chain A, Rabbit Muscle Aldolase AFRUCTOSE-1,6-Bisphosphate Complex
Observed Mr(expt) Mr(calc) ppm Start End Miss Peptide
1093.6052 1092.5979 1092.5563 38.1 322 - 330 1 K.AAQEEYVKR.A
1342.7589 1341.7516 1341.7041 35.4 87 - 98 0 K.ADDGRPFPQVIK.S
1646.8860 1645.8787 1645.8019 46.7 43 - 56 1 R.LQSIGTENTEENRR.F
1671.9034 1670.8961 1670.8702 15.5 201 - 214 1 R.CQYVTEKVLAAVYK.A
1691.8939 1690.8866 1690.8348 30.6 243 - 257 0 K.YSHEEIAMATVTALR.R
1809.0049 1807.9976 1807.9443 29.5 289 - 303 0 K.CPLLKPWALTFSYGR.A
1848.0056 1846.9983 1846.9359 33.8 243 - 258 1 K.YSHEEIAMATVTALRR.T
2123.1536 2122.1463 2122.0840 29.3 153 - 172 0 K.IGEHTPSALAIMENANVLAR.Y + Oxidation (M)
2272.2007 2271.1934 2271.1343 26.0 111 - 133 0 K.GVVPLAGTNGETTTQGLDGLSER.C
3113.6899 3112.6826 3112.6379 14.4 60 - 86 1 R.QLLLTADDRVNPCIGGVILFHETLYQK.A
No match to: 907.5164, 1007.5800, 1033.6152, 1157.5983, 1198.7487, 1231.6832, 1269.7450, 1408.7764, 1475.8400, 1503.8708,
1507.7773, 1515.8118,
1541.8221, 1542.8124, 1556.8605, 1558.8262, 1609.8159, 1615.9413, 1633.8643, 1634.8557, 1643.8777, 1763.8562, 1769.0546,
1784.9873, 1790.9573,
1791.9679, 1797.0793, 1801.0057, 1805.0021, 1833.0280, 1838.9164, 1849.0176, 1862.0222, 1865.0133, 1897.0234, 1913.0203,
1929.0089, 1954.0553,
1995.0031, 2075.1692, 2105.1135, 2139.1492, 2151.1697, 2333.2825, 2463.3313, 2572.3237, 2608.3215, 2615.3911, 2624.3879,
2672.3879, 2688.3962,
2927.4697, 3036.6570, 3177.6499, 3645.8721
14. gi|67464529 Mass: 39643 Score: 78 Expect: 0.015 Matches: 10
Chain A, Fructose-1,6-Bisphosphate Aldolase From Rabbit Muscle
Observed Mr(expt) Mr(calc) ppm Start End Miss Peptide
1093.6052 1092.5979 1092.5563 38.1 322 - 330 1 K.AAQEEYVKR.A
1342.7589 1341.7516 1341.7041 35.4 87 - 98 0 K.ADDGRPFPQVIK.S
1646.8860 1645.8787 1645.8019 46.7 43 - 56 1 R.LQSIGTENTEENRR.F
1671.9034 1670.8961 1670.8702 15.5 201 - 214 1 R.CQYVTEKVLAAVYK.A
1691.8939 1690.8866 1690.8348 30.6 243 - 257 0 K.YSHEEIAMATVTALR.R
1809.0049 1807.9976 1807.9443 29.5 289 - 303 0 K.CPLLKPWALTFSYGR.A
1848.0056 1846.9983 1846.9359 33.8 243 - 258 1 K.YSHEEIAMATVTALRR.T
2123.1536 2122.1463 2122.0840 29.3 153 - 172 0 K.IGEHTPSALAIMENANVLAR.Y + Oxidation (M)
2272.2007 2271.1934 2271.1343 26.0 111 - 133 0 K.GVVPLAGTNGETTTQGLDGLSER.C
3113.6899 3112.6826 3112.6379 14.4 60 - 86 1 R.QLLLTADDRVNPCIGGVILFHETLYQK.A
No match to: 907.5164, 1007.5800, 1033.6152, 1157.5983, 1198.7487, 1231.6832, 1269.7450, 1408.7764, 1475.8400, 1503.8708,
1507.7773, 1515.8118,
1541.8221, 1542.8124, 1556.8605, 1558.8262, 1609.8159, 1615.9413, 1633.8643, 1634.8557, 1643.8777, 1763.8562, 1769.0546,
1784.9873, 1790.9573,
1791.9679, 1797.0793, 1801.0057, 1805.0021, 1833.0280, 1838.9164, 1849.0176, 1862.0222, 1865.0133, 1897.0234, 1913.0203,
1929.0089, 1954.0553,
1995.0031, 2075.1692, 2105.1135, 2139.1492, 2151.1697, 2333.2825, 2463.3313, 2572.3237, 2608.3215, 2615.3911, 2624.3879,
2672.3879, 2688.3962,
2927.4697, 3036.6570, 3177.6499, 3645.8721
15. gi|160286558 Mass: 39642 Score: 78 Expect: 0.015 Matches: 10
Chain A, Fructose 1,6-Bisphosphate Aldolase From Rabbit Muscle
Observed Mr(expt) Mr(calc) ppm Start End Miss Peptide
1093.6052 1092.5979 1092.5563 38.1 322 - 330 1 K.AAQEEYVKR.A
1342.7589 1341.7516 1341.7041 35.4 87 - 98 0 K.ADDGRPFPQVIK.S
1646.8860 1645.8787 1645.8019 46.7 43 - 56 1 R.LQSIGTENTEENRR.F
1671.9034 1670.8961 1670.8702 15.5 201 - 214 1 R.CQYVTEKVLAAVYK.A
1691.8939 1690.8866 1690.8348 30.6 243 - 257 0 K.YSHEEIAMATVTALR.R
1809.0049 1807.9976 1807.9443 29.5 289 - 303 0 K.CPLLKPWALTFSYGR.A
1848.0056 1846.9983 1846.9359 33.8 243 - 258 1 K.YSHEEIAMATVTALRR.T
2123.1536 2122.1463 2122.0840 29.3 153 - 172 0 K.IGEHTPSALAIMENANVLAR.Y + Oxidation (M)
2272.2007 2271.1934 2271.1343 26.0 111 - 133 0 K.GVVPLAGTNGETTTQGLDGLSER.C
3113.6899 3112.6826 3112.6379 14.4 60 - 86 1 R.QLLLTADDRVNPCIGGVILFHETLYQK.A
No match to: 907.5164, 1007.5800, 1033.6152, 1157.5983, 1198.7487, 1231.6832, 1269.7450, 1408.7764, 1475.8400, 1503.8708,
1507.7773, 1515.8118,

1541.8221, 1542.8124, 1556.8605, 1558.8262, 1609.8159, 1615.9413, 1633.8643, 1634.8557, 1643.8777, 1763.8562, 1769.0546, 1784.9873, 1790.9573,
1791.9679, 1797.0793, 1801.0057, 1805.0021, 1833.0280, 1838.9164, 1849.0176, 1862.0222, 1865.0133, 1897.0234, 1913.0203, 1929.0089, 1954.0553,
1995.0031, 2075.1692, 2105.1135, 2139.1492, 2151.1697, 2333.2825, 2463.3313, 2572.3237, 2608.3215, 2615.3911, 2624.3879, 2672.3879, 2688.3962,
2927.4697, 3036.6570, 3177.6499, 3645.8721
16. gi|158430448 Mass: 39646 Score: 78 Expect: 0.015 Matches: 10
Chain A, Dihydroxyacetone Phosphate Schiff Base Intermediate In Mutant Fructose-1,6-Bisphosphate Aldolase From Rabbit Muscle
Observed Mr(expt) Mr(calc) ppm Start End Miss Peptide
1093.6052 1092.5979 1092.5563 38.1 322 - 330 1 K.AAQEEYVKR.A
1342.7589 1341.7516 1341.7041 35.4 87 - 98 0 K.ADDGRPFPQVIK.S
1646.8860 1645.8787 1645.8019 46.7 43 - 56 1 R.LQSIGTENTEENRR.F
1671.9034 1670.8961 1670.8702 15.5 201 - 214 1 R.CQYVTEKVLAAVYK.A
1691.8939 1690.8866 1690.8348 30.6 243 - 257 0 K.YSHEEIAMATVTALR.R
1809.0049 1807.9976 1807.9443 29.5 289 - 303 0 K.CPLLKPWALTFSYGR.A
1848.0056 1846.9983 1846.9359 33.8 243 - 258 1 K.YSHEEIAMATVTALRR.T
2123.1536 2122.1463 2122.0840 29.3 153 - 172 0 K.IGEHTPSALAIMENANVLAR.Y + Oxidation (M)
2272.2007 2271.1934 2271.1343 26.0 111 - 133 0 K.GVVPLAGTNGETTTQGLDGLSER.C
3113.6899 3112.6826 3112.6379 14.4 60 - 86 1 R.QLLLTADDRVNPCIGGVILFHETLYQK.A
No match to: 907.5164, 1007.5800, 1033.6152, 1157.5983, 1198.7487, 1231.6832, 1269.7450, 1408.7764, 1475.8400, 1503.8708, 1507.7773, 1515.8118,
1541.8221, 1542.8124, 1556.8605, 1558.8262, 1609.8159, 1615.9413, 1633.8643, 1634.8557, 1643.8777, 1763.8562, 1769.0546, 1784.9873, 1790.9573,
1791.9679, 1797.0793, 1801.0057, 1805.0021, 1833.0280, 1838.9164, 1849.0176, 1862.0222, 1865.0133, 1897.0234, 1913.0203, 1929.0089, 1954.0553,
1995.0031, 2075.1692, 2105.1135, 2139.1492, 2151.1697, 2333.2825, 2463.3313, 2572.3237, 2608.3215, 2615.3911, 2624.3879, 2672.3879, 2688.3962,
2927.4697, 3036.6570, 3177.6499, 3645.8721
17. gi|228311995 Mass: 39615 Score: 78 Expect: 0.015 Matches: 10
Chain A, D33s Mutant Fructose-1,6-Bisphosphate Aldolase From Rabbit Muscle
Observed Mr(expt) Mr(calc) ppm Start End Miss Peptide
1093.6052 1092.5979 1092.5563 38.1 322 - 330 1 K.AAQEEYVKR.A
1342.7589 1341.7516 1341.7041 35.4 87 - 98 0 K.ADDGRPFPQVIK.S
1646.8860 1645.8787 1645.8019 46.7 43 - 56 1 R.LQSIGTENTEENRR.F
1671.9034 1670.8961 1670.8702 15.5 201 - 214 1 R.CQYVTEKVLAAVYK.A
1691.8939 1690.8866 1690.8348 30.6 243 - 257 0 K.YSHEEIAMATVTALR.R
1809.0049 1807.9976 1807.9443 29.5 289 - 303 0 K.CPLLKPWALTFSYGR.A
1848.0056 1846.9983 1846.9359 33.8 243 - 258 1 K.YSHEEIAMATVTALRR.T
2123.1536 2122.1463 2122.0840 29.3 153 - 172 0 K.IGEHTPSALAIMENANVLAR.Y + Oxidation (M)
2272.2007 2271.1934 2271.1343 26.0 111 - 133 0 K.GVVPLAGTNGETTTQGLDGLSER.C
3113.6899 3112.6826 3112.6379 14.4 60 - 86 1 R.QLLLTADDRVNPCIGGVILFHETLYQK.A
No match to: 907.5164, 1007.5800, 1033.6152, 1157.5983, 1198.7487, 1231.6832, 1269.7450, 1408.7764, 1475.8400, 1503.8708, 1507.7773, 1515.8118,
1541.8221, 1542.8124, 1556.8605, 1558.8262, 1609.8159, 1615.9413, 1633.8643, 1634.8557, 1643.8777, 1763.8562, 1769.0546, 1784.9873, 1790.9573,
1791.9679, 1797.0793, 1801.0057, 1805.0021, 1833.0280, 1838.9164, 1849.0176, 1862.0222, 1865.0133, 1897.0234, 1913.0203, 1929.0089, 1954.0553,
1995.0031, 2075.1692, 2105.1135, 2139.1492, 2151.1697, 2333.2825, 2463.3313, 2572.3237, 2608.3215, 2615.3911, 2624.3879, 2672.3879, 2688.3962,
2927.4697, 3036.6570, 3177.6499, 3645.8721
18. gi|253722152 Mass: 39585 Score: 78 Expect: 0.015 Matches: 10
Chain A, Fructose 1,6-Bisphosphate Aldolase From Rabbit Muscle
Observed Mr(expt) Mr(calc) ppm Start End Miss Peptide
1093.6052 1092.5979 1092.5563 38.1 322 - 330 1 K.AAQEEYVKR.A
1342.7589 1341.7516 1341.7041 35.4 87 - 98 0 K.ADDGRPFPQVIK.S
1646.8860 1645.8787 1645.8019 46.7 43 - 56 1 R.LQSIGTENTEENRR.F
1671.9034 1670.8961 1670.8702 15.5 201 - 214 1 R.CQYVTEKVLAAVYK.A
1691.8939 1690.8866 1690.8348 30.6 243 - 257 0 K.YSHEEIAMATVTALR.R
1809.0049 1807.9976 1807.9443 29.5 289 - 303 0 K.CPLLKPWALTFSYGR.A
1848.0056 1846.9983 1846.9359 33.8 243 - 258 1 K.YSHEEIAMATVTALRR.T
2123.1536 2122.1463 2122.0840 29.3 153 - 172 0 K.IGEHTPSALAIMENANVLAR.Y + Oxidation (M)
2272.2007 2271.1934 2271.1343 26.0 111 - 133 0 K.GVVPLAGTNGETTTQGLDGLSER.C
3113.6899 3112.6826 3112.6379 14.4 60 - 86 1 R.QLLLTADDRVNPCIGGVILFHETLYQK.A
No match to: 907.5164, 1007.5800, 1033.6152, 1157.5983, 1198.7487, 1231.6832, 1269.7450, 1408.7764, 1475.8400, 1503.8708, 1507.7773, 1515.8118,
1541.8221, 1542.8124, 1556.8605, 1558.8262, 1609.8159, 1615.9413, 1633.8643, 1634.8557, 1643.8777, 1763.8562, 1769.0546, 1784.9873, 1790.9573,
1791.9679, 1797.0793, 1801.0057, 1805.0021, 1833.0280, 1838.9164, 1849.0176, 1862.0222, 1865.0133, 1897.0234, 1913.0203, 1929.0089, 1954.0553,
1995.0031, 2075.1692, 2105.1135, 2139.1492, 2151.1697, 2333.2825, 2463.3313, 2572.3237, 2608.3215, 2615.3911, 2624.3879, 2672.3879, 2688.3962,
2927.4697, 3036.6570, 3177.6499, 3645.8721
19. gi|126722869 Mass: 39774 Score: 78 Expect: 0.016 Matches: 10
fructose-bisphosphate aldolase A [Oryctolagus cuniculus]
Observed Mr(expt) Mr(calc) ppm Start End Miss Peptide
1093.6052 1092.5979 1092.5563 38.1 323 - 331 1 K.AAQEEYVKR.A
1342.7589 1341.7516 1341.7041 35.4 88 - 99 0 K.ADDGRPFPQVIK.S
1646.8860 1645.8787 1645.8019 46.7 44 - 57 1 R.LQSIGTENTEENRR.F
1671.9034 1670.8961 1670.8702 15.5 202 - 215 1 R.CQYVTEKVLAAVYK.A
1691.8939 1690.8866 1690.8348 30.6 244 - 258 0 K.YSHEEIAMATVTALR.R
1809.0049 1807.9976 1807.9443 29.5 290 - 304 0 K.CPLLKPWALTFSYGR.A
1848.0056 1846.9983 1846.9359 33.8 244 - 259 1 K.YSHEEIAMATVTALRR.T
2123.1536 2122.1463 2122.0840 29.3 154 - 173 0 K.IGEHTPSALAIMENANVLAR.Y + Oxidation (M)

2272.2007 2271.1934 2271.1343 26.0 112 - 134 0 K.GVVPLAGTNGETTTQGLDGLSER.C
3113.6899 3112.6826 3112.6379 14.4 61 - 87 1 R.QLLLTADDRVNPCIGGVILFHETLYQK.A
No match to: 907.5164, 1007.5800, 1033.6152, 1157.5983, 1198.7487, 1231.6832, 1269.7450, 1408.7764, 1475.8400, 1503.8708, 1507.7773, 1515.8118,
1541.8221, 1542.8124, 1556.8605, 1558.8262, 1609.8159, 1615.9413, 1633.8643, 1634.8557, 1643.8777, 1763.8562, 1769.0546, 1784.9873, 1790.9573,
1791.9679, 1797.0793, 1801.0057, 1805.0021, 1833.0280, 1838.9164, 1849.0176, 1862.0222, 1865.0133, 1897.0234, 1913.0203, 1929.0089, 1954.0553,
1995.0031, 2075.1692, 2105.1135, 2139.1492, 2151.1697, 2333.2825, 2463.3313, 2572.3237, 2608.3215, 2615.3911, 2624.3879, 2672.3879, 2688.3962,
2927.4697, 3036.6570, 3177.6499, 3645.8721
20. gi|4557305 Mass: 39851 Score: 78 Expect: 0.016 Matches: 10
fructose-bisphosphate aldolase A isoform 1 [Homo sapiens]
Observed Mr(expt) Mr(calc) ppm Start End Miss Peptide
1093.6052 1092.5979 1092.5563 38.1 323 - 331 1 K.AAQEEYVKR.A
1342.7589 1341.7516 1341.7041 35.4 88 - 99 0 K.ADDGRPFPQVIK.S
1646.8860 1645.8787 1645.8019 46.7 44 - 57 1 R.LQSIGTENTEENRR.F
1671.9034 1670.8961 1670.8702 15.5 202 - 215 1 R.CQYVTEKVLAAVYK.A
1691.8939 1690.8866 1690.8348 30.6 244 - 258 0 K.FSHEEIAMATVTALR.R + Oxidation (M)
1809.0049 1807.9976 1807.9443 29.5 290 - 304 0 K.CPLLKPWALTFSYGR.A
1848.0056 1846.9983 1846.9359 33.8 244 - 259 1 K.FSHEEIAMATVTALRR.T + Oxidation (M)
2123.1536 2122.1463 2122.0840 29.3 154 - 173 0 K.IGEHTPSALAIMENANVLAR.Y + Oxidation (M)
2272.2007 2271.1934 2271.1343 26.0 112 - 134 0 K.GVVPLAGTNGETTTQGLDGLSER.C
3113.6899 3112.6826 3112.6379 14.4 61 - 87 1 R.QLLLTADDRVNPCIGGVILFHETLYQK.A
No match to: 907.5164, 1007.5800, 1033.6152, 1157.5983, 1198.7487, 1231.6832, 1269.7450, 1408.7764, 1475.8400, 1503.8708, 1507.7773, 1515.8118,
1541.8221, 1542.8124, 1556.8605, 1558.8262, 1609.8159, 1615.9413, 1633.8643, 1634.8557, 1643.8777, 1763.8562, 1769.0546, 1784.9873, 1790.9573,
1791.9679, 1797.0793, 1801.0057, 1805.0021, 1833.0280, 1838.9164, 1849.0176, 1862.0222, 1865.0133, 1897.0234, 1913.0203, 1929.0089, 1954.0553,
1995.0031, 2075.1692, 2105.1135, 2139.1492, 2151.1697, 2333.2825, 2463.3313, 2572.3237, 2608.3215, 2615.3911, 2624.3879, 2672.3879, 2688.3962,
2927.4697, 3036.6570, 3177.6499, 3645.8721

Search Parameters

Type of search : Peptide Mass Fingerprint
Enzyme : Trypsin
Fixed modifications : Carbamidomethyl (C)
Variable modifications : Oxidation (M)
Mass values : Monoisotopic
Protein Mass : Unrestricted
Peptide Mass Tolerance : ± 50 ppm
Peptide Charge State : 1+
Max Missed Cleavages : 1
Number of queries : 65

Mascot: http://www.matrixscience.com/

COVERAGE BAND 3

Mascot Search Results

Protein View

Match to: gi|156120479 Score: 101 Expect: 8.4e-005
fructose-bisphosphate aldolase A [Bos taurus]
Nominal mass (Mr): 39925; Calculated pI value: 8.45
NCBI BLAST search of gi|156120479 against nr
Unformatted sequence string for pasting into other applications
Taxonomy: Bos taurus
Links to retrieve other entries containing this sequence from NCBI Entrez:
gi|151554749 from Bos taurus
gi|296473262 from Bos taurus
Fixed modifications: Carbamidomethyl (C)
Variable modifications: Oxidation (M)
Cleavage by Trypsin: cuts C-term side of KR unless next residue is P
Number of mass values searched: 65
Number of mass values matched: 13
Sequence Coverage: 45%
Matched peptides shown in **Bold Red**
1 M**PHQYPALTP EQK**KELCDIA HRIVAPGKGI LAADESTGSI AKR**LQSIGTE**
51 **NTEENRR**FYR **QLLLTADDRV NPCIGGVILF HETLYQKADD GRPFPQVIKA**
101 KGGVVGIKVD K**GVVPLAGTN GETTTQGLDG LSER**CAQYKK DGADFAKWRC
151 VLK**IGEHTPS SLAIMENANV LAR**YASICQQ NGIVPIVEPE ILPDGDHDLK
201 R**CQYVTEKVL AAVYK**ALSDH HIYLEGTLLK PNMVTPGHAC TQK**YSHEEIA**
251 **MATVTALRR**T VPPAVPGITF LSGGQSEEEA SINLNAINK**C PLLKPWALTF**
301 **SYGR**ALQASA LKAWGGKKEN LK**AAQEEYVK R**ALANSLACQ GKYTPSGKAG
351 AAASESLFIS NHAY
Show predicted peptides also
Sort Peptides By Residue Number Increasing Mass Decreasing Mass
Start - End Observed Mr(expt) Mr(calc) ppm Miss Sequence
2 - 13 1408.7764 1407.7691 1407.7146 39 0 M.PHQYPALTPEQK.K
44 - 57 1646.8860 1645.8787 1645.8019 47 1 R.LQSIGTENTEENRR.F

61 - 87 3113.6899 3112.6826 3112.6379 14 1 R.QLLLTADDRVNPCIGGVILFHETLYQK.A
88 - 99 1342.7589 1341.7516 1341.7041 35 0 K.ADDGRPFPQVIK.A
88 - 101 1541.8221 1540.8148 1540.8362 -14 1 K.ADDGRPFPQVIKAK.G
112 - 134 2272.2007 2271.1934 2271.1343 26 0 K.GVVPLAGTNGETTTQGLDGLSER.C
154 - 173 2123.1536 2122.1463 2122.0840 29 0 K.IGEHTPSSLAIMENANVLAR.Y
154 - 173 2139.1492 2138.1419 2138.0790 29 0 K.IGEHTPSSLAIMENANVLAR.Y Oxidation (M)
202 - 215 1671.9034 1670.8961 1670.8702 16 1 R.CQYVTEKVLAAVYK.A
244 - 258 1691.8939 1690.8866 1690.8348 31 0 K.YSHEEIAMATVTALR.R
244 - 259 1848.0056 1846.9983 1846.9359 34 1 K.YSHEEIAMATVTALRR.T
290 - 304 1809.0049 1807.9976 1807.9443 29 0 K.CPLLKPWALTFSYGR.A
323 - 331 1093.6052 1092.5979 1092.5563 38 1 K.AAQEEYVKR.A
No match to: 907.5164, 1007.5800, 1033.6152, 1157.5983, 1198.7487, 1231.6832, 1269.7450, 1475.8400, 1503.8708, 1507.7773, 1515.8

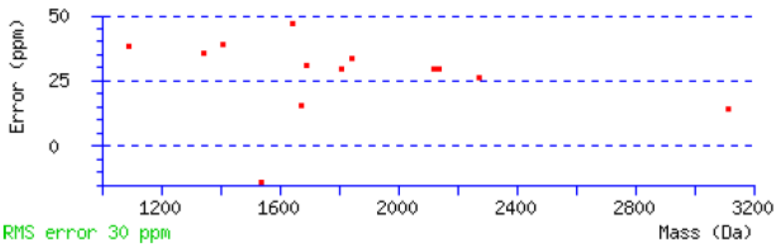

BAND 4

{MATRIX SCIENCE} Mascot Search Results

User :
Email :
Search title : SampleSetID: 825, AnalysisID: 7244, MaldiWellID: 70022, SpectrumID: 154638, Path=\180719\MSMS\18-106 combined NCBI Mammalia
Database : NCBInr 20120508 (17919084 sequences; 6150218869 residues)
Taxonomy : Mammalia (mammals) (1061927 sequences)
Timestamp : 19 Jul 2018 at 19:34:57 GMT
Warning : A Peptide summary report will usually give a much clearer picture of MS/MS search results.
Top Score : 257 for gi|4838363, creatine kinase M chain [Bos taurus]

Mascot Score Histogram

Protein score is -10*Log(P), where P is the probability that the observed match is a random event.
Protein scores greater than 73 are significant (p<0.05).
Protein scores are derived from ions scores as a non-probabilistic basis for ranking protein hits.

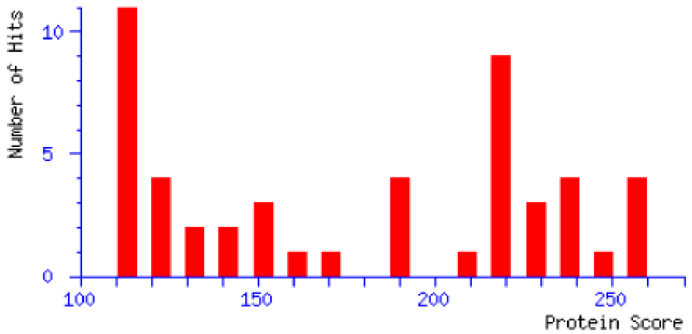

Protein Summary Report

Format As Protein Summary (deprecated) Help
Significance threshold p< 0.05 Max. number of hits 20

Re-Search All Search Unmatched

Index

Accession Mass Score Description

1. gi|4838363 43172 257 creatine kinase M chain [Bos taurus]
2. gi|194018722 43260 257 creatine kinase M-type [Sus scrofa]
3. gi|60097925 43190 257 creatine kinase M-type [Bos taurus]
4. gi|126344225 43205 256 PREDICTED: creatine kinase M-type-like [Monodelphis domestica]
5. gi|149512678 34374 243 PREDICTED: creatine kinase M-type-like [Ornithorhynchus anatinus]
6. gi|21536288 43302 242 creatine kinase M-type [Homo sapiens]
7. gi|189053833 43230 242 unnamed protein product [Homo sapiens]
8. gi|355703653 43302 242 hypothetical protein EGK_10748 [Macaca mulatta]
9. gi|148596827 43254 240 cytoplasmic creatine kinases [Physeter catodon]
10. gi|149722203 43370 232 PREDICTED: creatine kinase M-type-like [Equus caballus]
11. gi|119577741 45971 229 creatine kinase, muscle [Homo sapiens]
12. gi|301777736 43353 225 PREDICTED: creatine kinase M-type-like [Ailuropoda melanoleuca]
13. gi|194374181 30037 221 unnamed protein product [Homo sapiens]
14. gi|297705152 43287 219 PREDICTED: creatine kinase M-type-like isoform 2 [Pongo abelii]
15. gi|297705150 39717 219 PREDICTED: creatine kinase M-type-like isoform 1 [Pongo abelii]
16. gi|296234097 39747 218 PREDICTED: creatine kinase M-type [Callithrix jacchus]
17. gi|6671762 43246 215 creatine kinase M-type [Mus musculus]
18. gi|203480 33423 214 creatine kinase, partial [Rattus norvegicus]
19. gi|6978661 43220 214 creatine kinase M-type [Rattus norvegicus]
20. gi|348557636 43189 214 PREDICTED: creatine kinase M-type-like [Cavia porcellus]

Results List

1. gi|4838363 Mass: 43172 Score: 257 Expect: 2.1e-020 Matches: 17
creatine kinase M chain [Bos taurus]
Observed Mr(expt) Mr(calc) ppm Start End Miss Ions Peptide
914.4037 913.3964 913.4406 -48.38 2 - 9 0 --- M.PFGNTHNK.H
1507.6179 1506.6106 1506.6951 -56.04 117 - 130 0 --- K.GGDDLDPNYVLSSR.V
1643.7501 1642.7428 1642.8103 -41.10 224 - 236 0 --- K.SFLVWVNEEDHLR.V
1643.7501 1642.7428 1642.8103 -41.10 224 - 236 0 85 K.SFLVWVNEEDHLR.V
1785.9064 1784.8991 1784.9520 -29.63 342 - 358 0 --- R.LGSSEVEQVQLVVDGVK.L
1994.9225 1993.9152 1993.9342 -9.50 321 - 341 0 --- R.GTGGVDTAAVGSVFDVSNADR.L
2151.1016 2150.0943 2150.0353 27.5 320 - 341 1 --- K.RGTGGVDTAAVGSVFDVSNADR.L
2331.2039 2330.1966 2330.1729 10.2 224 - 242 1 --- K.SFLVWVNEEDHLRVISMEK.G
2572.3552 2571.3479 2571.2201 49.7 108 - 130 1 --- K.TDLNHENLKGGDDLDPNYVLSSR.V
2608.3289 2607.3216 2607.2619 22.9 216 - 236 1 --- R.GIWHNDNKSFLVWVNEEDHLR.V
2608.3289 2607.3216 2607.2619 22.9 216 - 236 1 51 R.GIWHNDNKSFLVWVNEEDHLR.V
2927.5188 2926.5115 2926.4007 37.9 267 - 292 0 --- K.AGHPFMWNEHLGYVLTCPSNLGTGLR.G
2943.5281 2942.5208 2942.3956 42.6 267 - 292 0 --- K.AGHPFMWNEHLGYVLTCPSNLGTGLR.G + Oxidation (M)
3055.6707 3054.6634 3054.4957 54.9 266 - 292 1 --- K.KAGHPFMWNEHLGYVLTCPSNLGTGLR.G
3645.0417 3644.0344 3643.8014 63.9 178 - 209 0 --- K.SMTEQEQQQLIDDHFLFDKPVSPLLLASGMAR.D
3661.0911 3660.0838 3659.7964 78.5 178 - 209 0 --- K.SMTEQEQQQLIDDHFLFDKPVSPLLLASGMAR.D + Oxidation (M)
3762.1592 3761.1519 3760.8756 73.5 321 - 358 1 --- R.GTGGVDTAAVGSVFDVSNADRLGSSEVEQVQLVVDGVK.L
No match to: 907.3469, 1007.4115, 1093.4382, 1157.4269, 1171.4481, 1231.5177, 1254.4310, 1269.5830, 1269.5830, 1342.6084, 1556.7295,
1609.6913, 1615.8054, 1671.7825, 1691.7800, 1700.7789, 1738.8986, 1763.7489, 1763.7489, 1768.9083, 1791.8480, 1808.9016, 1820.8134,
1847.8977, 1896.9609, 2075.0852, 2122.1492, 2123.0898, 2123.0898, 2139.0859, 2333.2327, 2349.2458, 2570.4192, 2590.3506, 2607.2808,
2615.3701, 2618.4346, 2665.3682, 2672.4224, 2879.5249, 2942.5737, 2955.5688, 2971.5481, 2984.5430, 3096.7930, 3103.7168, 3112.6802,
3113.7756, 3176.7761, 3177.7612, 3225.8472, 3597.0471, 3674.0859
2. gi|194018722 Mass: 43260 Score: 257 Expect: 2.1e-020 Matches: 17
creatine kinase M-type [Sus scrofa]
Observed Mr(expt) Mr(calc) ppm Start End Miss Ions Peptide
914.4037 913.3964 913.4406 -48.38 2 - 9 0 --- M.PFGNTHNK.Y
1507.6179 1506.6106 1506.6951 -56.04 117 - 130 0 --- K.GGDDLDPNYVLSSR.V
1643.7501 1642.7428 1642.8103 -41.10 224 - 236 0 --- K.SFLVWVNEEDHLR.V
1643.7501 1642.7428 1642.8103 -41.10 224 - 236 0 85 K.SFLVWVNEEDHLR.V
1785.9064 1784.8991 1784.9520 -29.63 342 - 358 0 --- R.LGSSEVEQVQLVVDGVK.L
1994.9225 1993.9152 1993.9342 -9.50 321 - 341 0 --- R.GTGGVDTAAVGSVFDVSNADR.L
2151.1016 2150.0943 2150.0353 27.5 320 - 341 1 --- K.RGTGGVDTAAVGSVFDVSNADR.L
2331.2039 2330.1966 2330.1729 10.2 224 - 242 1 --- K.SFLVWVNEEDHLRVISMEK.G
2572.3552 2571.3479 2571.2201 49.7 108 - 130 1 --- K.TDLNHENLKGGDDLDPNYVLSSR.V
2608.3289 2607.3216 2607.2619 22.9 216 - 236 1 --- R.GIWHNDNKSFLVWVNEEDHLR.V
2608.3289 2607.3216 2607.2619 22.9 216 - 236 1 51 R.GIWHNDNKSFLVWVNEEDHLR.V
2927.5188 2926.5115 2926.4007 37.9 267 - 292 0 --- K.AGHPFMWNEHLGYVLTCPSNLGTGLR.G
2943.5281 2942.5208 2942.3956 42.6 267 - 292 0 --- K.AGHPFMWNEHLGYVLTCPSNLGTGLR.G + Oxidation (M)
3055.6707 3054.6634 3054.4957 54.9 266 - 292 1 --- K.KAGHPFMWNEHLGYVLTCPSNLGTGLR.G
3645.0417 3644.0344 3643.8014 63.9 178 - 209 0 --- K.SMTEQEQQQLIDDHFLFDKPVSPLLLASGMAR.D
3661.0911 3660.0838 3659.7964 78.5 178 - 209 0 --- K.SMTEQEQQQLIDDHFLFDKPVSPLLLASGMAR.D + Oxidation (M)
3762.1592 3761.1519 3760.8756 73.5 321 - 358 1 --- R.GTGGVDTAAVGSVFDVSNADRLGSSEVEQVQLVVDGVK.L
No match to: 907.3469, 1007.4115, 1093.4382, 1157.4269, 1171.4481, 1231.5177, 1254.4310, 1269.5830, 1269.5830, 1342.6084, 1556.7295,
1609.6913, 1615.8054, 1671.7825, 1691.7800, 1700.7789, 1738.8986, 1763.7489, 1763.7489, 1768.9083, 1791.8480, 1808.9016, 1820.8134,

1847.8977, 1896.9609, 2075.0852, 2122.1492, 2123.0898, 2123.0898, 2139.0859, 2333.2327, 2349.2458, 2570.4192, 2590.3506, 2607.2808,
2615.3701, 2618.4346, 2665.3682, 2672.4224, 2879.5249, 2942.5737, 2955.5688, 2971.5481, 2984.5430, 3096.7930, 3103.7168, 3112.6802,
3113.7756, 3176.7761, 3177.7612, 3225.8472, 3597.0471, 3674.0859
3. gi|60097925 Mass: 43190 Score: 257 Expect: 2.1e-020 Matches: 17
creatine kinase M-type [Bos taurus]
Observed Mr(expt) Mr(calc) ppm Start End Miss Ions Peptide
914.4037 913.3964 913.4406 -48.38 2 - 9 0 --- M.PFGNTHNK.H
1507.6179 1506.6106 1506.6951 -56.04 117 - 130 0 --- K.GGDDLDPNYVLSSR.V
1643.7501 1642.7428 1642.8103 -41.10 224 - 236 0 --- K.SFLVWVNEEDHLR.V
1643.7501 1642.7428 1642.8103 -41.10 224 - 236 0 85 K.SFLVWVNEEDHLR.V
1785.9064 1784.8991 1784.9520 -29.63 342 - 358 0 --- R.LGSSEVEQVQLVVDGVK.L
1994.9225 1993.9152 1993.9342 -9.50 321 - 341 0 --- R.GTGGVDTAAVGSVFDVSNADR.L
2151.1016 2150.0943 2150.0353 27.5 320 - 341 1 --- K.RGTGGVDTAAVGSVFDVSNADR.L
2331.2039 2330.1966 2330.1729 10.2 224 - 242 1 --- K.SFLVWVNEEDHLRVISMEK.G
2572.3552 2571.3479 2571.2201 49.7 108 - 130 1 --- K.TDLNHENLKGGDDLDPNYVLSSR.V
2608.3289 2607.3216 2607.2619 22.9 216 - 236 1 --- R.GIWHNDNKSFLVWVNEEDHLR.V
2608.3289 2607.3216 2607.2619 22.9 216 - 236 1 51 R.GIWHNDNKSFLVWVNEEDHLR.V
2927.5188 2926.5115 2926.4007 37.9 267 - 292 0 --- K.AGHPFMWNEHLGYVLTCPSNLGTGLR.G
2943.5281 2942.5208 2942.3956 42.6 267 - 292 0 --- K.AGHPFMWNEHLGYVLTCPSNLGTGLR.G + Oxidation (M)
3055.6707 3054.6634 3054.4957 54.9 266 - 292 1 --- K.KAGHPFMWNEHLGYVLTCPSNLGTGLR.G
3645.0417 3644.0344 3643.8014 63.9 178 - 209 0 --- K.SMTEQEQQQLIDDHFLFDKPVSPLLLASGMAR.D
3661.0911 3660.0838 3659.7964 78.5 178 - 209 0 --- K.SMTEQEQQQLIDDHFLFDKPVSPLLLASGMAR.D + Oxidation (M)
3762.1592 3761.1519 3760.8756 73.5 321 - 358 1 --- R.GTGGVDTAAVGSVFDVSNADRLGSSEVEQVQLVVDGVK.L
No match to: 907.3469, 1007.4115, 1093.4382, 1157.4269, 1171.4481, 1231.5177, 1254.4310, 1269.5830, 1269.5830, 1342.6084, 1556.7295,
1609.6913, 1615.8054, 1671.7825, 1691.7800, 1700.7789, 1738.8986, 1763.7489, 1763.7489, 1768.9083, 1791.8480, 1808.9016, 1820.8134,
1847.8977, 1896.9609, 2075.0852, 2122.1492, 2123.0898, 2123.0898, 2139.0859, 2333.2327, 2349.2458, 2570.4192, 2590.3506, 2607.2808,
2615.3701, 2618.4346, 2665.3682, 2672.4224, 2879.5249, 2942.5737, 2955.5688, 2971.5481, 2984.5430, 3096.7930, 3103.7168, 3112.6802,
3113.7756, 3176.7761, 3177.7612, 3225.8472, 3597.0471, 3674.0859
4. gi|126344225 Mass: 43205 Score: 256 Expect: 2.7e-020 Matches: 17
PREDICTED: creatine kinase M-type-like [Monodelphis domestica]
Observed Mr(expt) Mr(calc) ppm Start End Miss Ions Peptide
914.4037 913.3964 913.4406 -48.38 2 - 9 0 --- M.PFGNTHNK.Y
1507.6179 1506.6106 1506.6951 -56.04 117 - 130 0 --- K.GGDDLDPNYVLSSR.V
1643.7501 1642.7428 1642.8103 -41.10 224 - 236 0 --- K.SFLVWVNEEDHLR.V
1643.7501 1642.7428 1642.8103 -41.10 224 - 236 0 85 K.SFLVWVNEEDHLR.V
1785.9064 1784.8991 1784.9520 -29.63 342 - 358 0 --- R.LGSSEVEQVQLVVDGVK.L
1994.9225 1993.9152 1993.9342 -9.50 321 - 341 0 --- R.GTGGVDTAAVGSVFDVSNADR.L
2151.1016 2150.0943 2150.0353 27.5 320 - 341 1 --- K.RGTGGVDTAAVGSVFDVSNADR.L
2331.2039 2330.1966 2330.1729 10.2 224 - 242 1 --- K.SFLVWVNEEDHLRVISMEK.G
2572.3552 2571.3479 2571.2201 49.7 108 - 130 1 --- K.TDLNHENLKGGDDLDPNYVLSSR.V
2608.3289 2607.3216 2607.2619 22.9 216 - 236 1 --- R.GIWHNDNKSFLVWVNEEDHLR.V
2608.3289 2607.3216 2607.2619 22.9 216 - 236 1 51 R.GIWHNDNKSFLVWVNEEDHLR.V
2927.5188 2926.5115 2926.4007 37.9 267 - 292 0 --- K.AGHPFMWNEHLGYVLTCPSNLGTGLR.G
2943.5281 2942.5208 2942.3956 42.6 267 - 292 0 --- K.AGHPFMWNEHLGYVLTCPSNLGTGLR.G + Oxidation (M)
3055.6707 3054.6634 3054.4957 54.9 266 - 292 1 --- K.KAGHPFMWNEHLGYVLTCPSNLGTGLR.G
3645.0417 3644.0344 3643.8014 63.9 178 - 209 0 --- K.SMTEQEQQQLIDDHFLFDKPVSPLLLASGMAR.D
3661.0911 3660.0838 3659.7964 78.5 178 - 209 0 --- K.SMTEQEQQQLIDDHFLFDKPVSPLLLASGMAR.D + Oxidation (M)
3762.1592 3761.1519 3760.8756 73.5 321 - 358 1 --- R.GTGGVDTAAVGSVFDVSNADRLGSSEVEQVQLVVDGVK.L
No match to: 907.3469, 1007.4115, 1093.4382, 1157.4269, 1171.4481, 1231.5177, 1254.4310, 1269.5830, 1269.5830, 1342.6084, 1556.7295,
1609.6913, 1615.8054, 1671.7825, 1691.7800, 1700.7789, 1738.8986, 1763.7489, 1763.7489, 1768.9083, 1791.8480, 1808.9016, 1820.8134,
1847.8977, 1896.9609, 2075.0852, 2122.1492, 2123.0898, 2123.0898, 2139.0859, 2333.2327, 2349.2458, 2570.4192, 2590.3506, 2607.2808,
2615.3701, 2618.4346, 2665.3682, 2672.4224, 2879.5249, 2942.5737, 2955.5688, 2971.5481, 2984.5430, 3096.7930, 3103.7168, 3112.6802,
3113.7756, 3176.7761, 3177.7612, 3225.8472, 3597.0471, 3674.0859
5. gi|149512678 Mass: 34374 Score: 243 Expect: 5.3e-019 Matches: 15
PREDICTED: creatine kinase M-type-like [Ornithorhynchus anatinus]
Observed Mr(expt) Mr(calc) ppm Start End Miss Ions Peptide
914.4037 913.3964 913.4406 -48.38 2 - 9 0 --- M.PFGNTHNK.Y
1643.7501 1642.7428 1642.8103 -41.10 146 - 158 0 --- K.SFLVWVNEEDHLR.V
1643.7501 1642.7428 1642.8103 -41.10 146 - 158 0 85 K.SFLVWVNEEDHLR.V
1785.9064 1784.8991 1784.9520 -29.63 264 - 280 0 --- R.LGSSEVEQVQLVVDGVK.L
1994.9225 1993.9152 1993.9342 -9.50 243 - 263 0 --- R.GTGGVDTAAVGSVFDVSNADR.L
2151.1016 2150.0943 2150.0353 27.5 242 - 263 1 --- K.RGTGGVDTAAVGSVFDVSNADR.L
2331.2039 2330.1966 2330.1729 10.2 146 - 164 1 --- K.SFLVWVNEEDHLRVISMEK.G
2608.3289 2607.3216 2607.2619 22.9 138 - 158 1 --- R.GIWHNDNKSFLVWVNEEDHLR.V
2608.3289 2607.3216 2607.2619 22.9 138 - 158 1 51 R.GIWHNDNKSFLVWVNEEDHLR.V
2927.5188 2926.5115 2926.4007 37.9 189 - 214 0 --- K.AGHPFMWNEHLGYVLTCPSNLGTGLR.G
2943.5281 2942.5208 2942.3956 42.6 189 - 214 0 --- K.AGHPFMWNEHLGYVLTCPSNLGTGLR.G + Oxidation (M)
3055.6707 3054.6634 3054.4957 54.9 188 - 214 1 --- K.KAGHPFMWNEHLGYVLTCPSNLGTGLR.G
3645.0417 3644.0344 3643.8014 63.9 100 - 131 0 --- K.SMTEQEQQQLIDDHFLFDKPVSPLLLASGMAR.D

3661.0911 3660.0838 3659.7964 78.5 100 - 131 0 --- K.SMTEQEQQQLIDDHFLFDKPVSPLLLASGMAR.D + Oxidation (M)
3762.1592 3761.1519 3760.8756 73.5 243 - 280 1 --- R.GTGGVDTAAVGSVFDVSNADRLGSSEVEQVQLVVDGVK.L
No match to: 907.3469, 1007.4115, 1093.4382, 1157.4269, 1171.4481, 1231.5177, 1254.4310, 1269.5830, 1269.5830, 1342.6084, 1507.6179,
1556.7295, 1609.6913, 1615.8054, 1671.7825, 1691.7800, 1700.7789, 1738.8986, 1763.7489, 1763.7489, 1768.9083, 1791.8480, 1808.9016,
1820.8134, 1847.8977, 1896.9609, 2075.0852, 2122.1492, 2123.0898, 2123.0898, 2139.0859, 2333.2327, 2349.2458, 2570.4192, 2572.3552,
2590.3506, 2607.2808, 2615.3701, 2618.4346, 2665.3682, 2672.4224, 2879.5249, 2942.5737, 2955.5688, 2971.5481, 2984.5430, 3096.7930,
3103.7168, 3112.6802, 3113.7756, 3176.7761, 3177.7612, 3225.8472, 3597.0471, 3674.0859
6. gi|21536288 Mass: 43302 Score: 242 Expect: 6.7e-019 Matches: 15
creatine kinase M-type [Homo sapiens]
Observed Mr(expt) Mr(calc) ppm Start End Miss Ions Peptide
914.4037 913.3964 913.4406 -48.38 2 - 9 0 --- M.PFGNTHNK.F
1507.6179 1506.6106 1506.6951 -56.04 117 - 130 0 --- K.GGDDLDPNYVLSSR.V
1643.7501 1642.7428 1642.8103 -41.10 224 - 236 0 --- K.SFLVWVNEEDHLR.V
1643.7501 1642.7428 1642.8103 -41.10 224 - 236 0 85 K.SFLVWVNEEDHLR.V
1785.9064 1784.8991 1784.9520 -29.63 342 - 358 0 --- R.LGSSEVEQVQLVVDGVK.L
1994.9225 1993.9152 1993.9342 -9.50 321 - 341 0 --- R.GTGGVDTAAVGSVFDVSNADR.L
2151.1016 2150.0943 2150.0353 27.5 320 - 341 1 --- K.RGTGGVDTAAVGSVFDVSNADR.L
2331.2039 2330.1966 2330.1729 10.2 224 - 242 1 --- K.SFLVWVNEEDHLRVISMEK.G
2572.3552 2571.3479 2571.2201 49.7 108 - 130 1 --- K.TDLNHENLKGGDDLDPNYVLSSR.V
2608.3289 2607.3216 2607.2619 22.9 216 - 236 1 --- R.GIWHNDNKSFLVWVNEEDHLR.V
2608.3289 2607.3216 2607.2619 22.9 216 - 236 1 51 R.GIWHNDNKSFLVWVNEEDHLR.V
2942.5737 2941.5664 2941.4116 52.6 267 - 292 0 --- K.AGHPFMWNQHLGYVLTCPSNLGTGLR.G + Oxidation (M)
3645.0417 3644.0344 3643.8378 54.0 178 - 209 1 --- K.SMTEKEQQQLIDDHFLFDKPVSPLLLASGMAR.D
3661.0911 3660.0838 3659.8327 68.6 178 - 209 1 --- K.SMTEKEQQQLIDDHFLFDKPVSPLLLASGMAR.D + Oxidation (M)
3762.1592 3761.1519 3760.8756 73.5 321 - 358 1 --- R.GTGGVDTAAVGSVFDVSNADRLGSSEVEQVQLVVDGVK.L
No match to: 907.3469, 1007.4115, 1093.4382, 1157.4269, 1171.4481, 1231.5177, 1254.4310, 1269.5830, 1269.5830, 1342.6084, 1556.7295,
1609.6913, 1615.8054, 1671.7825, 1691.7800, 1700.7789, 1738.8986, 1763.7489, 1763.7489, 1768.9083, 1791.8480, 1808.9016, 1820.8134,
1847.8977, 1896.9609, 2075.0852, 2122.1492, 2123.0898, 2123.0898, 2139.0859, 2333.2327, 2349.2458, 2570.4192, 2590.3506, 2607.2808,
2615.3701, 2618.4346, 2665.3682, 2672.4224, 2879.5249, 2927.5188, 2943.5281, 2955.5688, 2971.5481, 2984.5430, 3055.6707, 3096.7930,
3103.7168, 3112.6802, 3113.7756, 3176.7761, 3177.7612, 3225.8472, 3597.0471, 3674.0859
7. gi|189053833 Mass: 43230 Score: 242 Expect: 6.7e-019 Matches: 15
unnamed protein product [Homo sapiens]
Observed Mr(expt) Mr(calc) ppm Start End Miss Ions Peptide
914.4037 913.3964 913.4406 -48.38 2 - 9 0 --- M.PFGNTHNK.F
1507.6179 1506.6106 1506.6951 -56.04 117 - 130 0 --- K.GGDDLDPNYVLSSR.V
1643.7501 1642.7428 1642.8103 -41.10 224 - 236 0 --- K.SFLVWVNEEDHLR.V
1643.7501 1642.7428 1642.8103 -41.10 224 - 236 0 85 K.SFLVWVNEEDHLR.V
1785.9064 1784.8991 1784.9520 -29.63 342 - 358 0 --- R.LGSSEVEQVQLVVDGVK.L
1994.9225 1993.9152 1993.9342 -9.50 321 - 341 0 --- R.GTGGVDTAAVGSVFDVSNADR.L
2151.1016 2150.0943 2150.0353 27.5 320 - 341 1 --- K.RGTGGVDTAAVGSVFDVSNADR.L
2331.2039 2330.1966 2330.1729 10.2 224 - 242 1 --- K.SFLVWVNEEDHLRVISMEK.G
2572.3552 2571.3479 2571.2201 49.7 108 - 130 1 --- K.TDLNHENLKGGDDLDPNYVLSSR.V
2608.3289 2607.3216 2607.2619 22.9 216 - 236 1 --- R.GIWHNDNKSFLVWVNEEDHLR.V
2608.3289 2607.3216 2607.2619 22.9 216 - 236 1 51 R.GIWHNDNKSFLVWVNEEDHLR.V
2942.5737 2941.5664 2941.4116 52.6 267 - 292 0 --- K.AGHPFMWNQHLGYVLTCPSNLGTGLR.G + Oxidation (M)
3645.0417 3644.0344 3643.8378 54.0 178 - 209 1 --- K.SMTEKEQQQLIDDHFLFDKPVSPLLLASGMAR.D
3661.0911 3660.0838 3659.8327 68.6 178 - 209 1 --- K.SMTEKEQQQLIDDHFLFDKPVSPLLLASGMAR.D + Oxidation (M)
3762.1592 3761.1519 3760.8756 73.5 321 - 358 1 --- R.GTGGVDTAAVGSVFDVSNADRLGSSEVEQVQLVVDGVK.L
No match to: 907.3469, 1007.4115, 1093.4382, 1157.4269, 1171.4481, 1231.5177, 1254.4310, 1269.5830, 1269.5830, 1342.6084, 1556.7295,
1609.6913, 1615.8054, 1671.7825, 1691.7800, 1700.7789, 1738.8986, 1763.7489, 1763.7489, 1768.9083, 1791.8480, 1808.9016, 1820.8134,
1847.8977, 1896.9609, 2075.0852, 2122.1492, 2123.0898, 2123.0898, 2139.0859, 2333.2327, 2349.2458, 2570.4192, 2590.3506, 2607.2808,
2615.3701, 2618.4346, 2665.3682, 2672.4224, 2879.5249, 2927.5188, 2943.5281, 2955.5688, 2971.5481, 2984.5430, 3055.6707, 3096.7930,
3103.7168, 3112.6802, 3113.7756, 3176.7761, 3177.7612, 3225.8472, 3597.0471, 3674.0859
8. gi|355703653 Mass: 43302 Score: 242 Expect: 6.7e-019 Matches: 15
hypothetical protein EGK_10748 [Macaca mulatta]
Observed Mr(expt) Mr(calc) ppm Start End Miss Ions Peptide
914.4037 913.3964 913.4406 -48.38 2 - 9 0 --- M.PFGNTHNK.F
1507.6179 1506.6106 1506.6951 -56.04 117 - 130 0 --- K.GGDDLDPNYVLSSR.V
1643.7501 1642.7428 1642.8103 -41.10 224 - 236 0 --- K.SFLVWVNEEDHLR.V
1643.7501 1642.7428 1642.8103 -41.10 224 - 236 0 85 K.SFLVWVNEEDHLR.V
1785.9064 1784.8991 1784.9520 -29.63 342 - 358 0 --- R.LGSSEVEQVQLVVDGVK.L
1994.9225 1993.9152 1993.9342 -9.50 321 - 341 0 --- R.GTGGVDTAAVGSVFDVSNADR.L
2151.1016 2150.0943 2150.0353 27.5 320 - 341 1 --- K.RGTGGVDTAAVGSVFDVSNADR.L
2331.2039 2330.1966 2330.1729 10.2 224 - 242 1 --- K.SFLVWVNEEDHLRVISMEK.G
2572.3552 2571.3479 2571.2201 49.7 108 - 130 1 --- K.TDLNHENLKGGDDLDPNYVLSSR.V
2608.3289 2607.3216 2607.2619 22.9 216 - 236 1 --- R.GIWHNDNKSFLVWVNEEDHLR.V
2608.3289 2607.3216 2607.2619 22.9 216 - 236 1 51 R.GIWHNDNKSFLVWVNEEDHLR.V

2942.5737 2941.5664 2941.4116 52.6 267 - 292 0 --- K.AGHPFMWNQHLGYVLTCPSNLGTGLR.G + Oxidation (M)
3645.0417 3644.0344 3643.8378 54.0 178 - 209 1 --- K.SMTEKEQQQLIDDHFLFDKPVSPLLLASGMAR.D
3661.0911 3660.0838 3659.8327 68.6 178 - 209 1 --- K.SMTEKEQQQLIDDHFLFDKPVSPLLLASGMAR.D + Oxidation (M)
3762.1592 3761.1519 3760.8756 73.5 321 - 358 1 --- R.GTGGVDTAAVGSVFDVSNADRLGSSEVEQVQLVVDGVK.L
No match to: 907.3469, 1007.4115, 1093.4382, 1157.4269, 1171.4481, 1231.5177, 1254.4310, 1269.5830, 1269.5830, 1342.6084, 1556.7295,
1609.6913, 1615.8054, 1671.7825, 1691.7800, 1700.7789, 1738.8986, 1763.7489, 1763.7489, 1768.9083, 1791.8480, 1808.9016, 1820.8134,
1847.8977, 1896.9609, 2075.0852, 2122.1492, 2123.0898, 2123.0898, 2139.0859, 2333.2327, 2349.2458, 2570.4192, 2590.3506, 2607.2808,
2615.3701, 2618.4346, 2665.3682, 2672.4224, 2879.5249, 2927.5188, 2943.5281, 2955.5688, 2971.5481, 2984.5430, 3055.6707, 3096.7930,
3103.7168, 3112.6802, 3113.7756, 3176.7761, 3177.7612, 3225.8472, 3597.0471, 3674.0859
9. gi|148596827 Mass: 43254 Score: 240 Expect: 1.1e-018 Matches: 15
cytoplasmic creatine kinases [Physeter catodon]
Observed Mr(expt) Mr(calc) ppm Start End Miss Ions Peptide
914.4037 913.3964 913.4406 -48.38 2 - 9 0 --- M.PFGNTHNK.Y
1507.6179 1506.6106 1506.6951 -56.04 117 - 130 0 --- K.GGDDLDPNYVISSR.V
1643.7501 1642.7428 1642.8103 -41.10 224 - 236 0 --- K.SFLVWVNEEDHLR.V
1643.7501 1642.7428 1642.8103 -41.10 224 - 236 0 85 K.SFLVWVNEEDHLR.V
1785.9064 1784.8991 1784.9520 -29.63 342 - 358 0 --- R.LGSSEVEQVQLVVDGVK.L
1994.9225 1993.9152 1993.9342 -9.50 321 - 341 0 --- R.GTGGVDTAAVGSVFDVSNADR.L
2151.1016 2150.0943 2150.0353 27.5 320 - 341 1 --- K.RGTGGVDTAAVGSVFDVSNADR.L
2331.2039 2330.1966 2330.1729 10.2 224 - 242 1 --- K.SFLVWVNEEDHLRVISMEK.G
2572.3552 2571.3479 2571.2201 49.7 108 - 130 1 --- K.TDLNHENLKGGDDLDPNYVISSR.V
2608.3289 2607.3216 2607.2619 22.9 216 - 236 1 --- R.GIWHNDNKSFLVWVNEEDHLR.V
2608.3289 2607.3216 2607.2619 22.9 216 - 236 1 51 R.GIWHNDNKSFLVWVNEEDHLR.V
2927.5188 2926.5115 2926.4007 37.9 267 - 292 0 --- K.AGHPFMWNEHLGYVLTCPSNLGTGLR.G
2943.5281 2942.5208 2942.3956 42.6 267 - 292 0 --- K.AGHPFMWNEHLGYVLTCPSNLGTGLR.G + Oxidation (M)
3055.6707 3054.6634 3054.4957 54.9 266 - 292 1 --- K.KAGHPFMWNEHLGYVLTCPSNLGTGLR.G
3762.1592 3761.1519 3760.8756 73.5 321 - 358 1 --- R.GTGGVDTAAVGSVFDVSNADRLGSSEVEQVQLVVDGVK.L
No match to: 907.3469, 1007.4115, 1093.4382, 1157.4269, 1171.4481, 1231.5177, 1254.4310, 1269.5830, 1269.5830, 1342.6084, 1556.7295,
1609.6913, 1615.8054, 1671.7825, 1691.7800, 1700.7789, 1738.8986, 1763.7489, 1763.7489, 1768.9083, 1791.8480, 1808.9016, 1820.8134,
1847.8977, 1896.9609, 2075.0852, 2122.1492, 2123.0898, 2123.0898, 2139.0859, 2333.2327, 2349.2458, 2570.4192, 2590.3506, 2607.2808,
2615.3701, 2618.4346, 2665.3682, 2672.4224, 2879.5249, 2942.5737, 2955.5688, 2971.5481, 2984.5430, 3096.7930, 3103.7168, 3112.6802,
3113.7756, 3176.7761, 3177.7612, 3225.8472, 3597.0471, 3645.0417, 3661.0911, 3674.0859
10. gi|149722203 Mass: 43370 Score: 232 Expect: 6.7e-018 Matches: 15
PREDICTED: creatine kinase M-type-like [Equus caballus]
Observed Mr(expt) Mr(calc) ppm Start End Miss Ions Peptide
914.4037 913.3964 913.4406 -48.38 2 - 9 0 --- M.PFGNTHNK.F
1643.7501 1642.7428 1642.8103 -41.10 224 - 236 0 --- K.SFLVWVNEEDHLR.V
1643.7501 1642.7428 1642.8103 -41.10 224 - 236 0 85 K.SFLVWVNEEDHLR.V
1785.9064 1784.8991 1784.8806 10.4 117 - 132 1 --- K.GGDDLDPHYVLSSRVR.T
1994.9225 1993.9152 1993.9342 -9.50 321 - 341 0 --- R.GTGGVDTAAVGSVFDVSNADR.L
2151.1016 2150.0943 2150.0353 27.5 320 - 341 1 --- K.RGTGGVDTAAVGSVFDVSNADR.L
2331.2039 2330.1966 2330.1729 10.2 224 - 242 1 --- K.SFLVWVNEEDHLRVISMEK.G
2608.3289 2607.3216 2607.2619 22.9 216 - 236 1 --- R.GIWHNDNKSFLVWVNEEDHLR.V
2608.3289 2607.3216 2607.2619 22.9 216 - 236 1 51 R.GIWHNDNKSFLVWVNEEDHLR.V
2927.5188 2926.5115 2926.4007 37.9 267 - 292 0 --- K.AGHPFMWNEHLGYVLTCPSNLGTGLR.G
2943.5281 2942.5208 2942.3956 42.6 267 - 292 0 --- K.AGHPFMWNEHLGYVLTCPSNLGTGLR.G + Oxidation (M)
3055.6707 3054.6634 3054.4957 54.9 266 - 292 1 --- K.KAGHPFMWNEHLGYVLTCPSNLGTGLR.G
3645.0417 3644.0344 3643.8014 63.9 178 - 209 0 --- K.SMTEQEQQQLIDDHFLFDKPVSPLLLASGMAR.D
3661.0911 3660.0838 3659.7964 78.5 178 - 209 0 --- K.SMTEQEQQQLIDDHFLFDKPVSPLLLASGMAR.D + Oxidation (M)
3762.1592 3761.1519 3760.8756 73.5 321 - 358 1 --- R.GTGGVDTAAVGSVFDVSNADRLGSSEVEQVQLVVDGVK.L
No match to: 907.3469, 1007.4115, 1093.4382, 1157.4269, 1171.4481, 1231.5177, 1254.4310, 1269.5830, 1269.5830, 1342.6084, 1507.6179,
1556.7295, 1609.6913, 1615.8054, 1671.7825, 1691.7800, 1700.7789, 1738.8986, 1763.7489, 1763.7489, 1768.9083, 1791.8480, 1808.9016,
1820.8134, 1847.8977, 1896.9609, 2075.0852, 2122.1492, 2123.0898, 2123.0898, 2139.0859, 2333.2327, 2349.2458, 2570.4192, 2572.3552,
2590.3506, 2607.2808, 2615.3701, 2618.4346, 2665.3682, 2672.4224, 2879.5249, 2942.5737, 2955.5688, 2971.5481, 2984.5430, 3096.7930,
3103.7168, 3112.6802, 3113.7756, 3176.7761, 3177.7612, 3225.8472, 3597.0471, 3674.0859
11. gi|119577741 Mass: 45971 Score: 229 Expect: 1.3e-017 Matches: 14
creatine kinase, muscle [Homo sapiens]
Observed Mr(expt) Mr(calc) ppm Start End Miss Ions Peptide
1507.6179 1506.6106 1506.6951 -56.04 142 - 155 0 --- K.GGDDLDPNYVLSSR.V
1643.7501 1642.7428 1642.8103 -41.10 249 - 261 0 --- K.SFLVWVNEEDHLR.V
1643.7501 1642.7428 1642.8103 -41.10 249 - 261 0 85 K.SFLVWVNEEDHLR.V
1785.9064 1784.8991 1784.9520 -29.63 367 - 383 0 --- R.LGSSEVEQVQLVVDGVK.L
1994.9225 1993.9152 1993.9342 -9.50 346 - 366 0 --- R.GTGGVDTAAVGSVFDVSNADR.L
2151.1016 2150.0943 2150.0353 27.5 345 - 366 1 --- K.RGTGGVDTAAVGSVFDVSNADR.L
2331.2039 2330.1966 2330.1729 10.2 249 - 267 1 --- K.SFLVWVNEEDHLRVISMEK.G
2572.3552 2571.3479 2571.2201 49.7 133 - 155 1 --- K.TDLNHENLKGGDDLDPNYVLSSR.V
2608.3289 2607.3216 2607.2619 22.9 241 - 261 1 --- R.GIWHNDNKSFLVWVNEEDHLR.V

2608.3289 2607.3216 2607.2619 22.9 241 - 261 1 51 R.GIWHNDNKSFLVWVNEEDHLR.V
2942.5737 2941.5664 2941.4116 52.6 292 - 317 0 --- K.AGHPFMWNQHLGYVLTCPSNLGTGLR.G + Oxidation (M)
3645.0417 3644.0344 3643.8378 54.0 203 - 234 1 --- K.SMTEKEQQQLIDDHFLFDKPVSPLLLASGMAR.D
3661.0911 3660.0838 3659.8327 68.6 203 - 234 1 --- K.SMTEKEQQQLIDDHFLFDKPVSPLLLASGMAR.D + Oxidation (M)
3762.1592 3761.1519 3760.8756 73.5 346 - 383 1 --- R.GTGGVDTAAVGSVFDVSNADRLGSSEVEQVQLVVDGVK.L
No match to: 907.3469, 914.4037, 1007.4115, 1093.4382, 1157.4269, 1171.4481, 1231.5177, 1254.4310, 1269.5830, 1269.5830, 1342.6084,
1556.7295, 1609.6913, 1615.8054, 1671.7825, 1691.7800, 1700.7789, 1738.8986, 1763.7489, 1763.7489, 1768.9083, 1791.8480, 1808.9016,
1820.8134, 1847.8977, 1896.9609, 2075.0852, 2122.1492, 2123.0898, 2123.0898, 2139.0859, 2333.2327, 2349.2458, 2570.4192, 2590.3506,
2607.2808, 2615.3701, 2618.4346, 2665.3682, 2672.4224, 2879.5249, 2927.5188, 2943.5281, 2955.5688, 2971.5481, 2984.5430, 3055.6707,
3096.7930, 3103.7168, 3112.6802, 3113.7756, 3176.7761, 3177.7612, 3225.8472, 3597.0471, 3674.0859
12. gi|301777736 Mass: 43353 Score: 225 Expect: 3.4e-017 Matches: 15
PREDICTED: creatine kinase M-type-like [Ailuropoda melanoleuca]
Observed Mr(expt) Mr(calc) ppm Start End Miss Ions Peptide
914.4037 913.3964 913.4406 -48.38 2 - 9 0 --- M.PFGNTHNK.F
1507.6179 1506.6106 1506.6951 -56.04 117 - 130 0 --- K.GGDDLDPNYVISSR.V
1643.7501 1642.7428 1642.8103 -41.10 224 - 236 0 --- K.SFLVWVNEEDHLR.V
1643.7501 1642.7428 1642.8103 -41.10 224 - 236 0 85 K.SFLVWVNEEDHLR.V
1738.8986 1737.8913 1737.8461 26.0 12 - 25 0 --- K.LNYKPEEEYPDLTK.H
1785.9064 1784.8991 1784.9520 -29.63 342 - 358 0 --- R.LGSSEVEQVQLVVDGVK.L
2331.2039 2330.1966 2330.1729 10.2 224 - 242 1 --- K.SFLVWVNEEDHLRVISMEK.G
2572.3552 2571.3479 2571.2201 49.7 108 - 130 1 --- K.TDLNHENLKGGDDLDPNYVISSR.V
2608.3289 2607.3216 2607.2619 22.9 216 - 236 1 --- R.GIWHNDNKSFLVWVNEEDHLR.V
2608.3289 2607.3216 2607.2619 22.9 216 - 236 1 51 R.GIWHNDNKSFLVWVNEEDHLR.V
2927.5188 2926.5115 2926.4007 37.9 267 - 292 0 --- K.AGHPFMWNEHLGYVLTCPSNLGTGLR.G
2943.5281 2942.5208 2942.3956 42.6 267 - 292 0 --- K.AGHPFMWNEHLGYVLTCPSNLGTGLR.G + Oxidation (M)
3055.6707 3054.6634 3054.4957 54.9 266 - 292 1 --- K.KAGHPFMWNEHLGYVLTCPSNLGTGLR.G
3645.0417 3644.0344 3643.8378 54.0 178 - 209 1 --- K.SMTEKEQQQLIDDHFLFDKPVSPLLLASGMAR.D
3661.0911 3660.0838 3659.8327 68.6 178 - 209 1 --- K.SMTEKEQQQLIDDHFLFDKPVSPLLLASGMAR.D + Oxidation (M)
No match to: 907.3469, 1007.4115, 1093.4382, 1157.4269, 1171.4481, 1231.5177, 1254.4310, 1269.5830, 1269.5830, 1342.6084, 1556.7295,
1609.6913, 1615.8054, 1671.7825, 1691.7800, 1700.7789, 1763.7489, 1763.7489, 1768.9083, 1791.8480, 1808.9016, 1820.8134, 1847.8977,
1896.9609, 1994.9225, 2075.0852, 2122.1492, 2123.0898, 2123.0898, 2139.0859, 2151.1016, 2333.2327, 2349.2458, 2570.4192, 2590.3506,
2607.2808, 2615.3701, 2618.4346, 2665.3682, 2672.4224, 2879.5249, 2942.5737, 2955.5688, 2971.5481, 2984.5430, 3096.7930, 3103.7168,
3112.6802, 3113.7756, 3176.7761, 3177.7612, 3225.8472, 3597.0471, 3674.0859, 3762.1592
13. gi|194374181 Mass: 30037 Score: 221 Expect: 8.4e-017 Matches: 12
unnamed protein product [Homo sapiens]
Observed Mr(expt) Mr(calc) ppm Start End Miss Ions Peptide
1643.7501 1642.7428 1642.8103 -41.10 110 - 122 0 --- K.SFLVWVNEEDHLR.V
1643.7501 1642.7428 1642.8103 -41.10 110 - 122 0 85 K.SFLVWVNEEDHLR.V
1785.9064 1784.8991 1784.9520 -29.63 228 - 244 0 --- R.LGSSEVEQVQLVVDGVK.L
1994.9225 1993.9152 1993.9342 -9.50 207 - 227 0 --- R.GTGGVDTAAVGSVFDVSNADR.L
2151.1016 2150.0943 2150.0353 27.5 206 - 227 1 --- K.RGTGGVDTAAVGSVFDVSNADR.L
2331.2039 2330.1966 2330.1729 10.2 110 - 128 1 --- K.SFLVWVNEEDHLRVISMEK.G
2608.3289 2607.3216 2607.2619 22.9 102 - 122 1 --- R.GIWHNDNKSFLVWVNEEDHLR.V
2608.3289 2607.3216 2607.2619 22.9 102 - 122 1 51 R.GIWHNDNKSFLVWVNEEDHLR.V
2942.5737 2941.5664 2941.4116 52.6 153 - 178 0 --- K.AGHPFMWNQHLGYVLTCPSNLGTGLR.G + Oxidation (M)
3645.0417 3644.0344 3643.8378 54.0 64 - 95 1 --- K.SMTEKEQQQLIDDHFLFDKPVSPLLLASGMAR.D
3661.0911 3660.0838 3659.8327 68.6 64 - 95 1 --- K.SMTEKEQQQLIDDHFLFDKPVSPLLLASGMAR.D + Oxidation (M)
3762.1592 3761.1519 3760.8756 73.5 207 - 244 1 --- R.GTGGVDTAAVGSVFDVSNADRLGSSEVEQVQLVVDGVK.L
No match to: 907.3469, 914.4037, 1007.4115, 1093.4382, 1157.4269, 1171.4481, 1231.5177, 1254.4310, 1269.5830, 1269.5830, 1342.6084,
1507.6179, 1556.7295, 1609.6913, 1615.8054, 1671.7825, 1691.7800, 1700.7789, 1738.8986, 1763.7489, 1763.7489, 1768.9083, 1791.8480,
1808.9016, 1820.8134, 1847.8977, 1896.9609, 2075.0852, 2122.1492, 2123.0898, 2123.0898, 2139.0859, 2333.2327, 2349.2458, 2570.4192,
2572.3552, 2590.3506, 2607.2808, 2615.3701, 2618.4346, 2665.3682, 2672.4224, 2879.5249, 2927.5188, 2943.5281, 2955.5688, 2971.5481,
2984.5430, 3055.6707, 3096.7930, 3103.7168, 3112.6802, 3113.7756, 3176.7761, 3177.7612, 3225.8472, 3597.0471, 3674.0859
14. gi|297705152 Mass: 43287 Score: 219 Expect: 1.3e-016 Matches: 13
PREDICTED: creatine kinase M-type-like isoform 2 [Pongo abelii]
Observed Mr(expt) Mr(calc) ppm Start End Miss Ions Peptide
914.4037 913.3964 913.4406 -48.38 2 - 9 0 --- M.PFGNTHNK.F
1643.7501 1642.7428 1642.8103 -41.10 223 - 235 0 --- K.SFLVWVNEEDHLR.V
1643.7501 1642.7428 1642.8103 -41.10 223 - 235 0 85 K.SFLVWVNEEDHLR.V
1785.9064 1784.8991 1784.9520 -29.63 341 - 357 0 --- R.LGSSEVEQVQLVVDGVK.L
1994.9225 1993.9152 1993.9342 -9.50 320 - 340 0 --- R.GTGGVDTAAVGSVFDVSNADR.L
2151.1016 2150.0943 2150.0353 27.5 319 - 340 1 --- K.RGTGGVDTAAVGSVFDVSNADR.L
2331.2039 2330.1966 2330.1729 10.2 223 - 241 1 --- K.SFLVWVNEEDHLRVISMEK.G
2608.3289 2607.3216 2607.2619 22.9 215 - 235 1 --- R.GIWHNDNKSFLVWVNEEDHLR.V
2608.3289 2607.3216 2607.2619 22.9 215 - 235 1 51 R.GIWHNDNKSFLVWVNEEDHLR.V
2942.5737 2941.5664 2941.4116 52.6 266 - 291 0 --- K.AGHPFMWNQHLGYVLTCPSNLGTGLR.G + Oxidation (M)

3645.0417 3644.0344 3643.8378 54.0 177 - 208 1 --- K.SMTEKEQQQLIDDHFLFDKPVSPLLLASGMAR.D
3661.0911 3660.0838 3659.8327 68.6 177 - 208 1 --- K.SMTEKEQQQLIDDHFLFDKPVSPLLLASGMAR.D + Oxidation (M)
3762.1592 3761.1519 3760.8756 73.5 320 - 357 1 --- R.GTGGVDTAAVGSVFDVSNADRLGSSEVEQVQLVVDGVK.L
No match to: 907.3469, 1007.4115, 1093.4382, 1157.4269, 1171.4481, 1231.5177, 1254.4310, 1269.5830, 1269.5830, 1342.6084, 1507.6179,
1556.7295, 1609.6913, 1615.8054, 1671.7825, 1691.7800, 1700.7789, 1738.8986, 1763.7489, 1763.7489, 1768.9083, 1791.8480, 1808.9016,
1820.8134, 1847.8977, 1896.9609, 2075.0852, 2122.1492, 2123.0898, 2123.0898, 2139.0859, 2333.2327, 2349.2458, 2570.4192, 2572.3552,
2590.3506, 2607.2808, 2615.3701, 2618.4346, 2665.3682, 2672.4224, 2879.5249, 2927.5188, 2943.5281, 2955.5688, 2971.5481, 2984.5430,
3055.6707, 3096.7930, 3103.7168, 3112.6802, 3113.7756, 3176.7761, 3177.7612, 3225.8472, 3597.0471, 3674.0859
15. gi|297705150 Mass: 39717 Score: 219 Expect: 1.3e-016 Matches: 13
PREDICTED: creatine kinase M-type-like isoform 1 [Pongo abelii]
Observed Mr(expt) Mr(calc) ppm Start End Miss Ions Peptide
914.4037 913.3964 913.4406 -48.38 2 - 9 0 --- M.PFGNTHNK.F
1643.7501 1642.7428 1642.8103 -41.10 192 - 204 0 --- K.SFLVWVNEEDHLR.V
1643.7501 1642.7428 1642.8103 -41.10 192 - 204 0 85 K.SFLVWVNEEDHLR.V
1785.9064 1784.8991 1784.9520 -29.63 310 - 326 0 --- R.LGSSEVEQVQLVVDGVK.L
1994.9225 1993.9152 1993.9342 -9.50 289 - 309 0 --- R.GTGGVDTAAVGSVFDVSNADR.L
2151.1016 2150.0943 2150.0353 27.5 288 - 309 1 --- K.RGTGGVDTAAVGSVFDVSNADR.L
2331.2039 2330.1966 2330.1729 10.2 192 - 210 1 --- K.SFLVWVNEEDHLRVISMEK.G
2608.3289 2607.3216 2607.2619 22.9 184 - 204 1 --- R.GIWHNDNKSFLVWVNEEDHLR.V
2608.3289 2607.3216 2607.2619 22.9 184 - 204 1 51 R.GIWHNDNKSFLVWVNEEDHLR.V
2942.5737 2941.5664 2941.4116 52.6 235 - 260 0 --- K.AGHPFMWNQHLGYVLTCPSNLGTGLR.G + Oxidation (M)
3645.0417 3644.0344 3643.8378 54.0 146 - 177 1 --- K.SMTEKEQQQLIDDHFLFDKPVSPLLLASGMAR.D
3661.0911 3660.0838 3659.8327 68.6 146 - 177 1 --- K.SMTEKEQQQLIDDHFLFDKPVSPLLLASGMAR.D + Oxidation (M)
3762.1592 3761.1519 3760.8756 73.5 289 - 326 1 --- R.GTGGVDTAAVGSVFDVSNADRLGSSEVEQVQLVVDGVK.L
No match to: 907.3469, 1007.4115, 1093.4382, 1157.4269, 1171.4481, 1231.5177, 1254.4310, 1269.5830, 1269.5830, 1342.6084, 1507.6179,
1556.7295, 1609.6913, 1615.8054, 1671.7825, 1691.7800, 1700.7789, 1738.8986, 1763.7489, 1763.7489, 1768.9083, 1791.8480, 1808.9016,
1820.8134, 1847.8977, 1896.9609, 2075.0852, 2122.1492, 2123.0898, 2123.0898, 2139.0859, 2333.2327, 2349.2458, 2570.4192, 2572.3552,
2590.3506, 2607.2808, 2615.3701, 2618.4346, 2665.3682, 2672.4224, 2879.5249, 2927.5188, 2943.5281, 2955.5688, 2971.5481, 2984.5430,
3055.6707, 3096.7930, 3103.7168, 3112.6802, 3113.7756, 3176.7761, 3177.7612, 3225.8472, 3597.0471, 3674.0859
16. gi|296234097 Mass: 39747 Score: 218 Expect: 1.7e-016 Matches: 13
PREDICTED: creatine kinase M-type [Callithrix jacchus]
Observed Mr(expt) Mr(calc) ppm Start End Miss Ions Peptide
914.4037 913.3964 913.4406 -48.38 2 - 9 0 --- M.PFGNTHNK.Y
1643.7501 1642.7428 1642.8103 -41.10 192 - 204 0 --- K.SFLVWVNEEDHLR.V
1643.7501 1642.7428 1642.8103 -41.10 192 - 204 0 85 K.SFLVWVNEEDHLR.V
1785.9064 1784.8991 1784.9520 -29.63 310 - 326 0 --- R.LGSSEVEQVQLVVDGVK.L
1994.9225 1993.9152 1993.9342 -9.50 289 - 309 0 --- R.GTGGVDTAAVGSVFDVSNADR.L
2151.1016 2150.0943 2150.0353 27.5 288 - 309 1 --- K.RGTGGVDTAAVGSVFDVSNADR.L
2331.2039 2330.1966 2330.1729 10.2 192 - 210 1 --- K.SFLVWVNEEDHLRVISMEK.G
2608.3289 2607.3216 2607.2619 22.9 184 - 204 1 --- R.GIWHNDNKSFLVWVNEEDHLR.V
2608.3289 2607.3216 2607.2619 22.9 184 - 204 1 51 R.GIWHNDNKSFLVWVNEEDHLR.V
2942.5737 2941.5664 2941.4116 52.6 235 - 260 0 --- K.AGHPFMWNQHLGYVLTCPSNLGTGLR.G + Oxidation (M)
3645.0417 3644.0344 3643.8014 63.9 146 - 177 0 --- K.SMTEQEQQQLIDDHFLFDKPVSPLLLASGMAR.D
3661.0911 3660.0838 3659.7964 78.5 146 - 177 0 --- K.SMTEQEQQQLIDDHFLFDKPVSPLLLASGMAR.D + Oxidation (M)
3762.1592 3761.1519 3760.8756 73.5 289 - 326 1 --- R.GTGGVDTAAVGSVFDVSNADRLGSSEVEQVQLVVDGVK.L
No match to: 907.3469, 1007.4115, 1093.4382, 1157.4269, 1171.4481, 1231.5177, 1254.4310, 1269.5830, 1269.5830, 1342.6084, 1507.6179,
1556.7295, 1609.6913, 1615.8054, 1671.7825, 1691.7800, 1700.7789, 1738.8986, 1763.7489, 1763.7489, 1768.9083, 1791.8480, 1808.9016,
1820.8134, 1847.8977, 1896.9609, 2075.0852, 2122.1492, 2123.0898, 2123.0898, 2139.0859, 2333.2327, 2349.2458, 2570.4192, 2572.3552,
2590.3506, 2607.2808, 2615.3701, 2618.4346, 2665.3682, 2672.4224, 2879.5249, 2927.5188, 2943.5281, 2955.5688, 2971.5481, 2984.5430,
3055.6707, 3096.7930, 3103.7168, 3112.6802, 3113.7756, 3176.7761, 3177.7612, 3225.8472, 3597.0471, 3674.0859
17. gi|6671762 Mass: 43246 Score: 215 Expect: 3.4e-016 Matches: 14
creatine kinase M-type [Mus musculus]
Observed Mr(expt) Mr(calc) ppm Start End Miss Ions Peptide
914.4037 913.3964 913.4406 -48.38 2 - 9 0 --- M.PFGNTHNK.F
1507.6179 1506.6106 1506.6951 -56.04 117 - 130 0 --- K.GGDDLDPNYVLSSR.V
1643.7501 1642.7428 1642.8103 -41.10 224 - 236 0 --- K.SFLVWVNEEDHLR.V
1643.7501 1642.7428 1642.8103 -41.10 224 - 236 0 85 K.SFLVWVNEEDHLR.V
1785.9064 1784.8991 1784.9520 -29.63 342 - 358 0 --- R.LGSSEVEQVQLVVDGVK.L
2331.2039 2330.1966 2330.1729 10.2 224 - 242 1 --- K.SFLVWVNEEDHLRVISMEK.G
2572.3552 2571.3479 2571.2201 49.7 108 - 130 1 --- K.TDLNHENLKGGDDLDPNYVLSSR.V
2608.3289 2607.3216 2607.2619 22.9 216 - 236 1 --- R.GIWHNDNKSFLVWVNEEDHLR.V
2608.3289 2607.3216 2607.2619 22.9 216 - 236 1 51 R.GIWHNDNKSFLVWVNEEDHLR.V
2927.5188 2926.5115 2926.4007 37.9 267 - 292 0 --- K.AGHPFMWNEHLGYVLTCPSNLGTGLR.G
2943.5281 2942.5208 2942.3956 42.6 267 - 292 0 --- K.AGHPFMWNEHLGYVLTCPSNLGTGLR.G + Oxidation (M)
3055.6707 3054.6634 3054.4957 54.9 266 - 292 1 --- K.KAGHPFMWNEHLGYVLTCPSNLGTGLR.G
3645.0417 3644.0344 3643.8014 63.9 178 - 209 0 --- K.SMTEQEQQQLIDDHFLFDKPVSPLLLASGMAR.D
3661.0911 3660.0838 3659.7964 78.5 178 - 209 0 --- K.SMTEQEQQQLIDDHFLFDKPVSPLLLASGMAR.D + Oxidation (M)

No match to: 907.3469, 1007.4115, 1093.4382, 1157.4269, 1171.4481, 1231.5177, 1254.4310, 1269.5830, 1269.5830, 1342.6084, 1556.7295,
1609.6913, 1615.8054, 1671.7825, 1691.7800, 1700.7789, 1738.8986, 1763.7489, 1763.7489, 1768.9083, 1791.8480, 1808.9016, 1820.8134,
1847.8977, 1896.9609, 1994.9225, 2075.0852, 2122.1492, 2123.0898, 2123.0898, 2139.0859, 2151.1016, 2333.2327, 2349.2458, 2570.4192,
2590.3506, 2607.2808, 2615.3701, 2618.4346, 2665.3682, 2672.4224, 2879.5249, 2942.5737, 2955.5688, 2971.5481, 2984.5430, 3096.7930,
3103.7168, 3112.6802, 3113.7756, 3176.7761, 3177.7612, 3225.8472, 3597.0471, 3674.0859, 3762.1592
18. gi|203480 Mass: 33423 Score: 214 Expect: 4.2e-016 Matches: 13
creatine kinase, partial [Rattus norvegicus]
Observed Mr(expt) Mr(calc) ppm Start End Miss Ions Peptide
1507.6179 1506.6106 1506.6951 -56.04 31 - 44 0 --- K.GGDDLDPNYVLSSR.V
1643.7501 1642.7428 1642.8103 -41.10 138 - 150 0 --- K.SFLVWVNEEDHLR.V
1643.7501 1642.7428 1642.8103 -41.10 138 - 150 0 85 K.SFLVWVNEEDHLR.V
1785.9064 1784.8991 1784.9520 -29.63 256 - 272 0 --- R.LGSSEVEQVQLVVDGVK.L
2331.2039 2330.1966 2330.1729 10.2 138 - 156 1 --- K.SFLVWVNEEDHLRVISMEK.G
2572.3552 2571.3479 2571.2201 49.7 22 - 44 1 --- K.TDLNHENLKGGDDLDPNYVLSSR.V
2608.3289 2607.3216 2607.2619 22.9 130 - 150 1 --- R.GIWHNDNKSFLVWVNEEDHLR.V
2608.3289 2607.3216 2607.2619 22.9 130 - 150 1 51 R.GIWHNDNKSFLVWVNEEDHLR.V
2927.5188 2926.5115 2926.4007 37.9 181 - 206 0 --- K.AGHPFMWNEHLGYVLTCPSNLGTGLR.G
2943.5281 2942.5208 2942.3956 42.6 181 - 206 0 --- K.AGHPFMWNEHLGYVLTCPSNLGTGLR.G + Oxidation (M)
3055.6707 3054.6634 3054.4957 54.9 180 - 206 1 --- K.KAGHPFMWNEHLGYVLTCPSNLGTGLR.G
3645.0417 3644.0344 3643.8014 63.9 92 - 123 0 --- K.SMTEQEQQQLIDDHFLFDKPVSPLLLASGMAR.D
3661.0911 3660.0838 3659.7964 78.5 92 - 123 0 --- K.SMTEQEQQQLIDDHFLFDKPVSPLLLASGMAR.D + Oxidation (M)
No match to: 907.3469, 914.4037, 1007.4115, 1093.4382, 1157.4269, 1171.4481, 1231.5177, 1254.4310, 1269.5830, 1269.5830, 1342.6084,
1556.7295, 1609.6913, 1615.8054, 1671.7825, 1691.7800, 1700.7789, 1738.8986, 1763.7489, 1763.7489, 1768.9083, 1791.8480, 1808.9016,
1820.8134, 1847.8977, 1896.9609, 1994.9225, 2075.0852, 2122.1492, 2123.0898, 2123.0898, 2139.0859, 2151.1016, 2333.2327, 2349.2458,
2570.4192, 2590.3506, 2607.2808, 2615.3701, 2618.4346, 2665.3682, 2672.4224, 2879.5249, 2942.5737, 2955.5688, 2971.5481, 2984.5430,
3096.7930, 3103.7168, 3112.6802, 3113.7756, 3176.7761, 3177.7612, 3225.8472, 3597.0471, 3674.0859, 3762.1592
19. gi|6978661 Mass: 43220 Score: 214 Expect: 4.2e-016 Matches: 14
creatine kinase M-type [Rattus norvegicus]
Observed Mr(expt) Mr(calc) ppm Start End Miss Ions Peptide
914.4037 913.3964 913.4406 -48.38 2 - 9 0 --- M.PFGNTHNK.F
1507.6179 1506.6106 1506.6951 -56.04 117 - 130 0 --- K.GGDDLDPNYVLSSR.V
1643.7501 1642.7428 1642.8103 -41.10 224 - 236 0 --- K.SFLVWVNEEDHLR.V
1643.7501 1642.7428 1642.8103 -41.10 224 - 236 0 85 K.SFLVWVNEEDHLR.V
1785.9064 1784.8991 1784.9520 -29.63 342 - 358 0 --- R.LGSSEVEQVQLVVDGVK.L
2331.2039 2330.1966 2330.1729 10.2 224 - 242 1 --- K.SFLVWVNEEDHLRVISMEK.G
2572.3552 2571.3479 2571.2201 49.7 108 - 130 1 --- K.TDLNHENLKGGDDLDPNYVLSSR.V
2608.3289 2607.3216 2607.2619 22.9 216 - 236 1 --- R.GIWHNDNKSFLVWVNEEDHLR.V
2608.3289 2607.3216 2607.2619 22.9 216 - 236 1 51 R.GIWHNDNKSFLVWVNEEDHLR.V
2927.5188 2926.5115 2926.4007 37.9 267 - 292 0 --- K.AGHPFMWNEHLGYVLTCPSNLGTGLR.G
2943.5281 2942.5208 2942.3956 42.6 267 - 292 0 --- K.AGHPFMWNEHLGYVLTCPSNLGTGLR.G + Oxidation (M)
3055.6707 3054.6634 3054.4957 54.9 266 - 292 1 --- K.KAGHPFMWNEHLGYVLTCPSNLGTGLR.G
3645.0417 3644.0344 3643.8014 63.9 178 - 209 0 --- K.SMTEQEQQQLIDDHFLFDKPVSPLLLASGMAR.D
3661.0911 3660.0838 3659.7964 78.5 178 - 209 0 --- K.SMTEQEQQQLIDDHFLFDKPVSPLLLASGMAR.D + Oxidation (M)
No match to: 907.3469, 1007.4115, 1093.4382, 1157.4269, 1171.4481, 1231.5177, 1254.4310, 1269.5830, 1269.5830, 1342.6084, 1556.7295,
1609.6913, 1615.8054, 1671.7825, 1691.7800, 1700.7789, 1738.8986, 1763.7489, 1763.7489, 1768.9083, 1791.8480, 1808.9016, 1820.8134,
1847.8977, 1896.9609, 1994.9225, 2075.0852, 2122.1492, 2123.0898, 2123.0898, 2139.0859, 2151.1016, 2333.2327, 2349.2458, 2570.4192,
2590.3506, 2607.2808, 2615.3701, 2618.4346, 2665.3682, 2672.4224, 2879.5249, 2942.5737, 2955.5688, 2971.5481, 2984.5430, 3096.7930,
3103.7168, 3112.6802, 3113.7756, 3176.7761, 3177.7612, 3225.8472, 3597.0471, 3674.0859, 3762.1592
20. gi|348557636 Mass: 43189 Score: 214 Expect: 4.2e-016 Matches: 14
PREDICTED: creatine kinase M-type-like [Cavia porcellus]
Observed Mr(expt) Mr(calc) ppm Start End Miss Ions Peptide
914.4037 913.3964 913.4406 -48.38 2 - 9 0 --- M.PFGNTHNK.F
1507.6179 1506.6106 1506.6951 -56.04 117 - 130 0 --- K.GGDDLDPNYVLSSR.V
1643.7501 1642.7428 1642.8103 -41.10 224 - 236 0 --- K.SFLVWVNEEDHLR.V
1643.7501 1642.7428 1642.8103 -41.10 224 - 236 0 85 K.SFLVWVNEEDHLR.V
1785.9064 1784.8991 1784.9520 -29.63 342 - 358 0 --- R.LGSSEVEQVQLVVDGVK.L
2331.2039 2330.1966 2330.1729 10.2 224 - 242 1 --- K.SFLVWVNEEDHLRVISMEK.G
2572.3552 2571.3479 2571.2201 49.7 108 - 130 1 --- K.TDLNHENLKGGDDLDPNYVLSSR.V
2608.3289 2607.3216 2607.2619 22.9 216 - 236 1 --- R.GIWHNDNKSFLVWVNEEDHLR.V
2608.3289 2607.3216 2607.2619 22.9 216 - 236 1 51 R.GIWHNDNKSFLVWVNEEDHLR.V
2927.5188 2926.5115 2926.4007 37.9 267 - 292 0 --- K.AGHPFMWNEHLGYVLTCPSNLGTGLR.G
2943.5281 2942.5208 2942.3956 42.6 267 - 292 0 --- K.AGHPFMWNEHLGYVLTCPSNLGTGLR.G + Oxidation (M)
3055.6707 3054.6634 3054.4957 54.9 266 - 292 1 --- K.KAGHPFMWNEHLGYVLTCPSNLGTGLR.G
3645.0417 3644.0344 3643.8014 63.9 178 - 209 0 --- K.SMTEQEQQQLIDDHFLFDKPVSPLLLASGMAR.D
3661.0911 3660.0838 3659.7964 78.5 178 - 209 0 --- K.SMTEQEQQQLIDDHFLFDKPVSPLLLASGMAR.D + Oxidation (M)
No match to: 907.3469, 1007.4115, 1093.4382, 1157.4269, 1171.4481, 1231.5177, 1254.4310, 1269.5830, 1269.5830, 1342.6084, 1556.7295,

1609.6913, 1615.8054, 1671.7825, 1691.7800, 1700.7789, 1738.8986, 1763.7489, 1763.7489, 1768.9083, 1791.8480, 1808.9016, 1820.8134,
1847.8977, 1896.9609, 1994.9225, 2075.0852, 2122.1492, 2123.0898, 2123.0898, 2139.0859, 2151.1016, 2333.2327, 2349.2458, 2570.4192,
2590.3506, 2607.2808, 2615.3701, 2618.4346, 2665.3682, 2672.4224, 2879.5249, 2942.5737, 2955.5688, 2971.5481, 2984.5430, 3096.7930,
3103.7168, 3112.6802, 3113.7756, 3176.7761, 3177.7612, 3225.8472, 3597.0471, 3674.0859, 3762.1592

Search Parameters

Type of search : Sequence Query
Enzyme : Trypsin
Fixed modifications : Carbamidomethyl (C)
Variable modifications : Oxidation (M)
Mass values : Monoisotopic
Protein Mass : Unrestricted
Peptide Mass Tolerance : ± 80 ppm
Fragment Mass Tolerance: ± 0.3 Da
Max Missed Cleavages : 1
Instrument type : MALDI-TOF-TOF
Query1 (907.3469,1+) : <no title>
Query2 (914.4037,1+) : <no title>
Query3 (1007.4115,1+) : <no title>
Query4 (1093.4382,1+) : <no title>
Query5 (1157.4269,1+) : <no title>
Query6 (1171.4481,1+) : <no title>
Query7 (1231.5177,1+) : <no title>
Query8 (1254.4310,1+) : <no title>
Query9 (1269.5830,1+) : <no title>
Query10 (1269.5830,1+) : MaldiWellID: 70022, SpectrumID: 154641,
Query11 (1342.6084,1+) : <no title>
Query12 (1507.6179,1+) : <no title>
Query13 (1556.7295,1+) : <no title>
Query14 (1609.6913,1+) : <no title>
Query15 (1615.8054,1+) : <no title>
Query16 (1643.7501,1+) : <no title>
Query17 (1643.7501,1+) : MaldiWellID: 70022, SpectrumID: 154639,
Query18 (1671.7825,1+) : <no title>
Query19 (1691.7800,1+) : <no title>
Query20 (1700.7789,1+) : <no title>
Query21 (1738.8986,1+) : <no title>
Query22 (1763.7489,1+) : <no title>
Query23 (1763.7489,1+) : MaldiWellID: 70022, SpectrumID: 154640,
Query24 (1768.9083,1+) : <no title>
Query25 (1785.9064,1+) : <no title>
Query26 (1791.8480,1+) : <no title>
Query27 (1808.9016,1+) : <no title>
Query28 (1820.8134,1+) : <no title>
Query29 (1847.8977,1+) : <no title>
Query30 (1896.9609,1+) : <no title>
Query31 (1994.9225,1+) : <no title>
Query32 (2075.0852,1+) : <no title>
Query33 (2122.1492,1+) : <no title>
Query34 (2123.0898,1+) : <no title>
Query35 (2123.0898,1+) : MaldiWellID: 70022, SpectrumID: 154642,
Query36 (2139.0859,1+) : <no title>
Query37 (2151.1016,1+) : <no title>
Query38 (2331.2039,1+) : <no title>
Query39 (2333.2327,1+) : <no title>
Query40 (2349.2458,1+) : <no title>
Query41 (2570.4192,1+) : <no title>
Query42 (2572.3552,1+) : <no title>
Query43 (2590.3506,1+) : <no title>
Query44 (2607.2808,1+) : <no title>
Query45 (2608.3289,1+) : <no title>
Query46 (2608.3289,1+) : MaldiWellID: 70022, SpectrumID: 154643,
Query47 (2615.3701,1+) : <no title>
Query48 (2618.4346,1+) : <no title>
Query49 (2665.3682,1+) : <no title>
Query50 (2672.4224,1+) : <no title>
Query51 (2879.5249,1+) : <no title>
Query52 (2927.5188,1+) : <no title>
Query53 (2942.5737,1+) : <no title>
Query54 (2943.5281,1+) : <no title>
Query55 (2955.5688,1+) : <no title>
Query56 (2971.5481,1+) : <no title>
Query57 (2984.5430,1+) : <no title>
Query58 (3055.6707,1+) : <no title>
Query59 (3096.7930,1+) : <no title>
Query60 (3103.7168,1+) : <no title>

Query61 (3112.6802,1+) : <no title>
Query62 (3113.7756,1+) : <no title>
Query63 (3176.7761,1+) : <no title>
Query64 (3177.7612,1+) : <no title>
Query65 (3225.8472,1+) : <no title>
Query66 (3597.0471,1+) : <no title>
Query67 (3645.0417,1+) : <no title>
Query68 (3661.0911,1+) : <no title>
Query69 (3674.0859,1+) : <no title>
Query70 (3762.1592,1+) : <no title>

Mascot: http://www.matrixscience.com/

COVERAGE BAND 4

Mascot Search Results

Protein View

Match to: gi|4838363 Score: 75 Expect: 0.032
creatine kinase M chain [Bos taurus]
Nominal mass (M_r): 43172; Calculated pI value: 6.63
NCBI BLAST search of gi|4838363 against nr
Unformatted sequence string for pasting into other applications
Taxonomy: Bos taurus
Fixed modifications: Carbamidomethyl (C)
Variable modifications: Oxidation (M)
Cleavage by Trypsin: cuts C-term side of KR unless next residue is P
Number of mass values searched: 65
Number of mass values matched: 11
Sequence Coverage: 37%
Matched peptides shown in **Bold Red**

1 MPFGNTHNKH KLNFKAEEEY PDLSKHNNHM AKALTLEIYK KLRDKETPSG
51 FTLDDVIQTG VDNPGHPFIM TVGCVAGDEE SYTVFKDLFD PIIQDRHGGF
101 KPTDKHKTDL NHENLKGGDD LDPNYVLSSR VRTGRSIKGY ALPPHCSRGE
151 RRAVEKLSVE ALNSLTGEFK GKYYPLKSMT EQEQQQLIDD HFLFDKPVSP
201 LLLASGMARD WPDARGIWHN DNKSFLVWVN EEDHLRVISM EKGGNMKEVF
251 RRFCVGLQKI EEIFKKAGHP FMWNEHLGYV LTCPSNLGTG LRGGVHVKLA
301 HLSKHPKFEE ILTRLRLQKR GTGGVDTAAV GSVFDVSNAD RLGSSEVEQV
351 QLVVDGVKLM VEMEKKLEKG QSIDDLIPAQ K

Show predicted peptides also
Sort Peptides By Residue Number Increasing Mass Decreasing Mass

Start - End Observed Mr(expt) Mr(calc) ppm Miss Sequence
87 - 96 1231.6832 1230.6759 1230.6245 42 0 K.DLFDPIIQDR.H
108 - 130 2572.3237 2571.3164 2571.2201 37 1 K.TDLNHENLKGGDDLDPNYVLSSR.V
139 - 148 1157.5983 1156.5910 1156.5448 40 0 K.GYALPPHCSR.G
157 - 170 1507.7773 1506.7700 1506.7930 -15 0 K.LSVEALNSLTGEFK.G
216 - 236 2608.3215 2607.3142 2607.2619 20 1 R.GIWHNDNKSFLVWVNEEDHLR.V
224 - 236 1643.8777 1642.8704 1642.8103 37 0 K.SFLVWVNEEDHLR.V
252 - 259 1007.5800 1006.5727 1006.5382 34 1 R.RFCVGLQK.I
267 - 292 2927.4697 2926.4624 2926.4007 21 0 K.AGHPFMWNEHLGYVLTCPSNLGTGLR.G
305 - 314 1269.7450 1268.7377 1268.6877 39 1 K.HPKFEEILTR.L
308 - 314 907.5164 906.5091 906.4811 31 0 K.FEEILTR.L
321 - 341 1995.0031 1993.9958 1993.9342 31 0 R.GTGGVDTAAVGSVFDVSNADR.L
No match to: 1033.6152, 1093.6052, 1198.7487, 1342.7589, 1408.7764, 1475.8400, 1503.8708, 1515.8118, 1541.8221, 1542.8124, 1556.

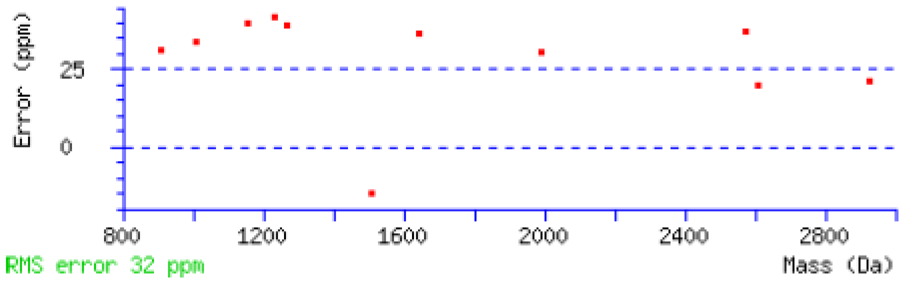

BAND 5

Mascot Search Results

User :
Email :
Search title : SampleSetID: 824, AnalysisID: 7270, MaldiWellID: 69640, SpectrumID: 154838, Path=\180719\MS\18-106 NCBI Mammalia run4
Database : NCBInr 20120508 (17919084 sequences; 6150218869 residues)
Taxonomy : Mammalia (mammals) (1061927 sequences)
Timestamp : 23 Jul 2018 at 17:16:29 GMT
Top Score : 130 for gi|156120479, fructose-bisphosphate aldolase A [Bos taurus]

Mascot Score Histogram

Protein score is -10*Log(P), where P is the probability that the observed match is a random event.
Protein scores greater than 73 are significant (p<0.05).

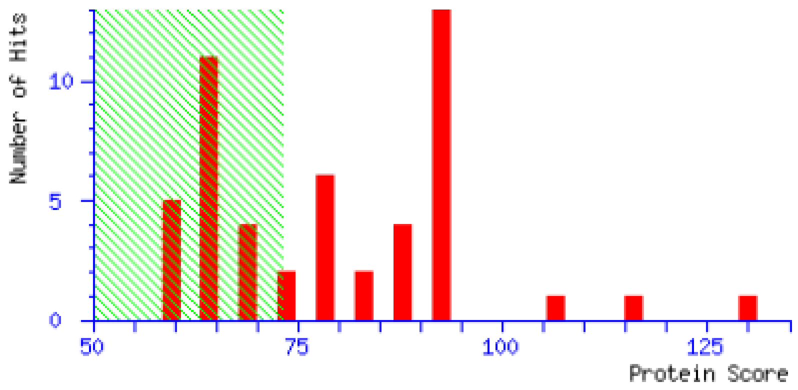

Protein Summary Report

Format As Protein Summary Help
Significance threshold p< 0.05 Max. number of hits 20
Re-Search All Search Unmatched

Index

Accession Mass Score Description
1. gi|156120479 39925 130 fructose-bisphosphate aldolase A [Bos taurus]
2. gi|194219069 39889 117 PREDICTED: fructose-bisphosphate aldolase A isoform 1 [Equus caballus]
3. gi|348584274 39838 105 PREDICTED: fructose-bisphosphate aldolase A-like [Cavia porcellus]
4. gi|4557976 39720 93 Chain A, Human Muscle Fructose 1,6-Bisphosphate Aldolase Complexed With Fructose 1,6-Bisphosphate
5. gi|2781027 39647 93 Chain A, Fructose 1,6-Bisphosphate Aldolase From Rabbit Muscle
6. gi|13096351 39646 93 Chain A, Fructose 1,6-Bisphosphate Aldolase From Rabbit Muscle
7. gi|13096347 39636 93 Chain A, Fructose 1,6-Bisphosphate Aldolase From Rabbit Muscle
8. gi|6730618 39586 93 Chain A, Rabbit Muscle Aldolase AFRUCTOSE-1,6-Bisphosphate Complex
9. gi|67464529 39643 93 Chain A, Fructose-1,6-Bisphosphate Aldolase From Rabbit Muscle
10. gi|158430448 39646 93 Chain A, Dihydroxyacetone Phosphate Schiff Base Intermediate In Mutant Fructose-1,6-Bisphosphate Aldolase From Rabbit Muscle
11. gi|228311978 39642 93 Chain A, D33n Mutant Fructose-1,6-Bisphosphate Aldolase From Rabbit Muscle
12. gi|228311995 39615 93 Chain A, D33s Mutant Fructose-1,6-Bisphosphate Aldolase From Rabbit Muscle
13. gi|126722869 39774 93 fructose-bisphosphate aldolase A [Oryctolagus cuniculus]
14. gi|4557305 39851 93 fructose-bisphosphate aldolase A isoform 1 [Homo sapiens]
15. gi|90078570 39860 93 unnamed protein product [Macaca fascicularis]
16. gi|49456715 39880 93 ALDOA [Homo sapiens]
17. gi|338712747 45488 88 PREDICTED: fructose-bisphosphate aldolase A isoform 4 [Equus caballus]
18. gi|342187211 45688 87 fructose-bisphosphate aldolase A isoform 2 [Homo sapiens]
19. gi|332265962 45656 87 PREDICTED: fructose-bisphosphate aldolase A isoform 4 [Nomascus leucogenys]
20. gi|355756689 45711 87 Fructose-bisphosphate aldolase A [Macaca fascicularis]

Results List

1. gi|156120479 Mass: 39925 Score: 130 Expect: 1.1e-007 Matches: 15
fructose-bisphosphate aldolase A [Bos taurus]
Observed Mr(expt) Mr(calc) ppm Start End Miss Peptide
1093.5890 1092.5817 1092.5563 23.2 323 - 331 1 K.AAQEEYVKR.A
1342.7362 1341.7289 1341.7041 18.5 88 - 99 0 K.ADDGRPFPQVIK.A
1408.7529 1407.7456 1407.7146 22.0 2 - 13 0 M.PHQYPALTPEQK.K
1536.8516 1535.8443 1535.8096 22.6 2 - 14 1 M.PHQYPALTPEQKK.E
1608.7865 1607.7792 1607.7579 13.2 349 - 364 0 K.AGAAASESLFISNHAY.-
1671.8793 1670.8720 1670.8702 1.11 202 - 215 1 R.CQYVTEKVLAAVYK.A
1691.8704 1690.8631 1690.8348 16.7 244 - 258 0 K.YSHEEIAMATVTALR.R
1808.9792 1807.9719 1807.9443 15.3 290 - 304 0 K.CPLLKPWALTFSYGR.A
1847.9763 1846.9690 1846.9359 17.9 244 - 259 1 K.YSHEEIAMATVTALRR.T
2088.1143 2087.1070 2087.0874 9.41 70 - 87 0 R.VNPCIGGVILFHETLYQK.A

2123.1194 2122.1121 2122.0840 13.2 154 - 173 0 K.IGEHTPSSLAIMENANVLAR.Y
2139.1208 2138.1135 2138.0790 16.2 154 - 173 0 K.IGEHTPSSLAIMENANVLAR.Y + Oxidation (M)
2272.1853 2271.1780 2271.1343 19.3 112 - 134 0 K.GVVPLAGTNGETTTQGLDGLSER.C
3113.6753 3112.6680 3112.6379 9.67 61 - 87 1 R.QLLLTADDRVNPCIGGVILFHETLYQK.A
3176.6453 3175.6380 3175.5972 12.9 174 - 201 1 R.YASICQQNGIVPIVEPEILPDGDHDLKR.C
No match to: 854.3170, 876.3025, 914.5256, 1033.5979, 1231.6539, 1269.7175, 1609.7772, 1615.9065, 1643.8455, 1665.8262, 1700.8666, 1718.9393,
1763.8304, 1791.9521, 1799.9424, 1807.9392, 1820.8546, 1823.9868, 1840.9702, 1864.9916, 1865.9926, 1905.0056, 2027.1447, 2075.1184, 2151.1465,
2166.1360, 2180.1448, 2333.2385, 2608.2981, 2615.3550, 2666.3325, 2879.4438, 2927.4353, 2943.4583, 2985.4792, 3022.5537, 3054.6343, 3096.6506,
3170.7014, 3177.6328, 3205.6589, 3210.7000, 3225.7222, 3234.6455, 3412.8198, 3596.8699, 3644.8401, 3661.8950, 3761.9360, 3874.9299
2. gi|194219069 Mass: 39889 Score: 117 Expect: 2.1e-006 Matches: 14
PREDICTED: fructose-bisphosphate aldolase A isoform 1 [Equus caballus]
Observed Mr(expt) Mr(calc) ppm Start End Miss Peptide
1093.5890 1092.5817 1092.5563 23.2 323 - 331 1 K.AAQEEYVKR.A
1342.7362 1341.7289 1341.7041 18.5 88 - 99 0 K.ADDGRPFPQVIK.S
1408.7529 1407.7456 1407.7146 22.0 2 - 13 0 M.PHQYPALTPEQK.K
1536.8516 1535.8443 1535.8096 22.6 2 - 14 1 M.PHQYPALTPEQKK.E
1671.8793 1670.8720 1670.8702 1.11 202 - 215 1 R.CQYVTEKVLAAVYK.A
1691.8704 1690.8631 1690.8348 16.7 244 - 258 0 K.YSHEEIAMATVTALR.R
1808.9792 1807.9719 1807.9443 15.3 290 - 304 0 K.CPLLKPWALTFSYGR.A
1847.9763 1846.9690 1846.9359 17.9 244 - 259 1 K.YSHEEIAMATVTALRR.T
2088.1143 2087.1070 2087.0874 9.41 70 - 87 0 R.VNPCIGGVILFHETLYQK.A
2123.1194 2122.1121 2122.0840 13.2 154 - 173 0 K.IGEHTPSSLAIMENANVLAR.Y
2139.1208 2138.1135 2138.0790 16.2 154 - 173 0 K.IGEHTPSSLAIMENANVLAR.Y + Oxidation (M)
2272.1853 2271.1780 2271.1343 19.3 112 - 134 0 K.GVVPLAGTNGETTTQGLDGLSER.C
3113.6753 3112.6680 3112.6379 9.67 61 - 87 1 R.QLLLTADDRVNPCIGGVILFHETLYQK.A
3176.6453 3175.6380 3175.5972 12.9 174 - 201 1 R.YASICQQNGIVPIVEPEILPDGDHDLKR.C
No match to: 854.3170, 876.3025, 914.5256, 1033.5979, 1231.6539, 1269.7175, 1608.7865, 1609.7772, 1615.9065, 1643.8455, 1665.8262, 1700.8666,
1718.9393, 1763.8304, 1791.9521, 1799.9424, 1807.9392, 1820.8546, 1823.9868, 1840.9702, 1864.9916, 1865.9926, 1905.0056, 2027.1447, 2075.1184,
2151.1465, 2166.1360, 2180.1448, 2333.2385, 2608.2981, 2615.3550, 2666.3325, 2879.4438, 2927.4353, 2943.4583, 2985.4792, 3022.5537, 3054.6343,
3096.6506, 3170.7014, 3177.6328, 3205.6589, 3210.7000, 3225.7222, 3234.6455, 3412.8198, 3596.8699, 3644.8401, 3661.8950, 3761.9360, 3874.9299
3. gi|348584274 Mass: 39838 Score: 105 Expect: 3.4e-005 Matches: 12
PREDICTED: fructose-bisphosphate aldolase A-like [Cavia porcellus]
Observed Mr(expt) Mr(calc) ppm Start End Miss Peptide
1342.7362 1341.7289 1341.7041 18.5 88 - 99 0 K.ADDGRPFPQVIK.S
1408.7529 1407.7456 1407.7146 22.0 2 - 13 0 M.PHQYPALTPEQK.K
1536.8516 1535.8443 1535.8096 22.6 2 - 14 1 M.PHQYPALTPEQKK.E
1671.8793 1670.8720 1670.8702 1.11 202 - 215 1 R.CQYVTEKVLAAVYK.A
1691.8704 1690.8631 1690.8348 16.7 244 - 258 0 K.YSHEEIAMATVTALR.R
1808.9792 1807.9719 1807.9443 15.3 290 - 304 0 K.CPLLKPWALTFSYGR.A
1847.9763 1846.9690 1846.9359 17.9 244 - 259 1 K.YSHEEIAMATVTALRR.T
2088.1143 2087.1070 2087.0874 9.41 70 - 87 0 R.VNPCIGGVILFHETLYQK.A
2123.1194 2122.1121 2122.0840 13.2 154 - 173 0 K.IGEHTPSALAIMENANVLAR.Y + Oxidation (M)
2272.1853 2271.1780 2271.1343 19.3 112 - 134 0 K.GVVPLAGTNGETTTQGLDGLSER.C
3113.6753 3112.6680 3112.6379 9.67 61 - 87 1 R.QLLLTADDRVNPCIGGVILFHETLYQK.A
3176.6453 3175.6380 3175.5972 12.9 174 - 201 1 R.YASICQQNGIVPIVEPEILPDGDHDLKR.C
No match to: 854.3170, 876.3025, 914.5256, 1033.5979, 1093.5890, 1231.6539, 1269.7175, 1608.7865, 1609.7772, 1615.9065, 1643.8455, 1665.8262,
1700.8666, 1718.9393, 1763.8304, 1791.9521, 1799.9424, 1807.9392, 1820.8546, 1823.9868, 1840.9702, 1864.9916, 1865.9926, 1905.0056, 2027.1447,
2075.1184, 2139.1208, 2151.1465, 2166.1360, 2180.1448, 2333.2385, 2608.2981, 2615.3550, 2666.3325, 2879.4438, 2927.4353, 2943.4583, 2985.4792,
3022.5537, 3054.6343, 3096.6506, 3170.7014, 3177.6328, 3205.6589, 3210.7000, 3225.7222, 3234.6455, 3412.8198, 3596.8699, 3644.8401, 3661.8950,
3761.9360, 3874.9299
4. gi|4557976 Mass: 39720 Score: 93 Expect: 0.00051 Matches: 11
Chain A, Human Muscle Fructose 1,6-Bisphosphate Aldolase Complexed With Fructose 1,6-Bisphosphate
Observed Mr(expt) Mr(calc) ppm Start End Miss Peptide
1093.5890 1092.5817 1092.5563 23.2 322 - 330 1 K.AAQEEYVKR.A
1342.7362 1341.7289 1341.7041 18.5 87 - 98 0 K.ADDGRPFPQVIK.S
1671.8793 1670.8720 1670.8702 1.11 201 - 214 1 R.CQYVTEKVLAAVYK.A
1691.8704 1690.8631 1690.8348 16.7 243 - 257 0 K.FSHEEIAMATVTALR.R + Oxidation (M)
1808.9792 1807.9719 1807.9443 15.3 289 - 303 0 K.CPLLKPWALTFSYGR.A
1847.9763 1846.9690 1846.9359 17.9 243 - 258 1 K.FSHEEIAMATVTALRR.T + Oxidation (M)
2088.1143 2087.1070 2087.0874 9.41 69 - 86 0 R.VNPCIGGVILFHETLYQK.A
2123.1194 2122.1121 2122.0840 13.2 153 - 172 0 K.IGEHTPSALAIMENANVLAR.Y + Oxidation (M)
2272.1853 2271.1780 2271.1343 19.3 111 - 133 0 K.GVVPLAGTNGETTTQGLDGLSER.C
3113.6753 3112.6680 3112.6379 9.67 60 - 86 1 R.QLLLTADDRVNPCIGGVILFHETLYQK.A
3176.6453 3175.6380 3175.5972 12.9 173 - 200 1 R.YASICQQNGIVPIVEPEILPDGDHDLKR.C
No match to: 854.3170, 876.3025, 914.5256, 1033.5979, 1231.6539, 1269.7175, 1408.7529, 1536.8516, 1608.7865, 1609.7772, 1615.9065, 1643.8455,
1665.8262, 1700.8666, 1718.9393, 1763.8304, 1791.9521, 1799.9424, 1807.9392, 1820.8546, 1823.9868, 1840.9702, 1864.9916, 1865.9926, 1905.0056,
2027.1447, 2075.1184, 2139.1208, 2151.1465, 2166.1360, 2180.1448, 2333.2385, 2608.2981, 2615.3550, 2666.3325, 2879.4438, 2927.4353, 2943.4583,
2985.4792, 3022.5537, 3054.6343, 3096.6506, 3170.7014, 3177.6328, 3205.6589, 3210.7000, 3225.7222, 3234.6455, 3412.8198, 3596.8699, 3644.8401,
3661.8950, 3761.9360, 3874.9299

5. gi|2781027 Mass: 39647 Score: 93 Expect: 0.00051 Matches: 11
Chain A, Fructose 1,6-Bisphosphate Aldolase From Rabbit Muscle
Observed Mr(expt) Mr(calc) ppm Start End Miss Peptide
1093.5890 1092.5817 1092.5563 23.2 322 - 330 1 K.AAQEEYVKR.A
1342.7362 1341.7289 1341.7041 18.5 87 - 98 0 K.ADDGRPFPQVIK.S
1671.8793 1670.8720 1670.8702 1.11 201 - 214 1 R.CQYVTEKVLAAVYK.A
1691.8704 1690.8631 1690.8348 16.7 243 - 257 0 K.YSHEEIAMATVTALR.R
1808.9792 1807.9719 1807.9443 15.3 289 - 303 0 K.CPLLKPWALTFSYGR.A
1847.9763 1846.9690 1846.9359 17.9 243 - 258 1 K.YSHEEIAMATVTALRR.T
2088.1143 2087.1070 2087.0874 9.41 69 - 86 0 R.VNPCIGGVILFHETLYQK.A
2123.1194 2122.1121 2122.0840 13.2 153 - 172 0 K.IGEHTPSALAIMENANVLAR.Y + Oxidation (M)
2272.1853 2271.1780 2271.1343 19.3 111 - 133 0 K.GVVPLAGTNGETTTQGLDGLSER.C
3113.6753 3112.6680 3112.6379 9.67 60 - 86 1 R.QLLLTADDRVNPCIGGVILFHETLYQK.A
3176.6453 3175.6380 3175.5972 12.9 173 - 200 1 R.YASICQQNGIVPIVEPEILPDGDHDLKR.C
No match to: 854.3170, 876.3025, 914.5256, 1033.5979, 1231.6539, 1269.7175, 1408.7529, 1536.8516, 1608.7865, 1609.7772, 1615.9065, 1643.8455,
1665.8262, 1700.8666, 1718.9393, 1763.8304, 1791.9521, 1799.9424, 1807.9392, 1820.8546, 1823.9868, 1840.9702, 1864.9916, 1865.9926, 1905.0056,
2027.1447, 2075.1184, 2139.1208, 2151.1465, 2166.1360, 2180.1448, 2333.2385, 2608.2981, 2615.3550, 2666.3325, 2879.4438, 2927.4353, 2943.4583,
2985.4792, 3022.5537, 3054.6343, 3096.6506, 3170.7014, 3177.6328, 3205.6589, 3210.7000, 3225.7222, 3234.6455, 3412.8198, 3596.8699, 3644.8401,
3661.8950, 3761.9360, 3874.9299
6. gi|13096351 Mass: 39646 Score: 93 Expect: 0.00051 Matches: 11
Chain A, Fructose 1,6-Bisphosphate Aldolase From Rabbit Muscle
Observed Mr(expt) Mr(calc) ppm Start End Miss Peptide
1093.5890 1092.5817 1092.5563 23.2 322 - 330 1 K.AAQEEYVKR.A
1342.7362 1341.7289 1341.7041 18.5 87 - 98 0 K.ADDGRPFPQVIK.S
1671.8793 1670.8720 1670.8702 1.11 201 - 214 1 R.CQYVTEKVLAAVYK.A
1691.8704 1690.8631 1690.8348 16.7 243 - 257 0 K.YSHEEIAMATVTALR.R
1808.9792 1807.9719 1807.9443 15.3 289 - 303 0 K.CPLLKPWALTFSYGR.A
1847.9763 1846.9690 1846.9359 17.9 243 - 258 1 K.YSHEEIAMATVTALRR.T
2088.1143 2087.1070 2087.0874 9.41 69 - 86 0 R.VNPCIGGVILFHETLYQK.A
2123.1194 2122.1121 2122.0840 13.2 153 - 172 0 K.IGEHTPSALAIMENANVLAR.Y + Oxidation (M)
2272.1853 2271.1780 2271.1343 19.3 111 - 133 0 K.GVVPLAGTNGETTTQGLDGLSER.C
3113.6753 3112.6680 3112.6379 9.67 60 - 86 1 R.QLLLTADDRVNPCIGGVILFHETLYQK.A
3176.6453 3175.6380 3175.5972 12.9 173 - 200 1 R.YASICQQNGIVPIVEPEILPDGDHDLKR.C
No match to: 854.3170, 876.3025, 914.5256, 1033.5979, 1231.6539, 1269.7175, 1408.7529, 1536.8516, 1608.7865, 1609.7772, 1615.9065, 1643.8455,
1665.8262, 1700.8666, 1718.9393, 1763.8304, 1791.9521, 1799.9424, 1807.9392, 1820.8546, 1823.9868, 1840.9702, 1864.9916, 1865.9926, 1905.0056,
2027.1447, 2075.1184, 2139.1208, 2151.1465, 2166.1360, 2180.1448, 2333.2385, 2608.2981, 2615.3550, 2666.3325, 2879.4438, 2927.4353, 2943.4583,
2985.4792, 3022.5537, 3054.6343, 3096.6506, 3170.7014, 3177.6328, 3205.6589, 3210.7000, 3225.7222, 3234.6455, 3412.8198, 3596.8699, 3644.8401,
3661.8950, 3761.9360, 3874.9299
7. gi|13096347 Mass: 39636 Score: 93 Expect: 0.00051 Matches: 11
Chain A, Fructose 1,6-Bisphosphate Aldolase From Rabbit Muscle
Observed Mr(expt) Mr(calc) ppm Start End Miss Peptide
1093.5890 1092.5817 1092.5563 23.2 322 - 330 1 K.AAQEEYVKR.A
1342.7362 1341.7289 1341.7041 18.5 87 - 98 0 K.ADDGRPFPQVIK.S
1671.8793 1670.8720 1670.8702 1.11 201 - 214 1 R.CQYVTEKVLAAVYK.A
1691.8704 1690.8631 1690.8348 16.7 243 - 257 0 K.YSHEEIAMATVTALR.R
1808.9792 1807.9719 1807.9443 15.3 289 - 303 0 K.CPLLKPWALTFSYGR.A
1847.9763 1846.9690 1846.9359 17.9 243 - 258 1 K.YSHEEIAMATVTALRR.T
2088.1143 2087.1070 2087.0874 9.41 69 - 86 0 R.VNPCIGGVILFHETLYQK.A
2123.1194 2122.1121 2122.0840 13.2 153 - 172 0 K.IGEHTPSALAIMENANVLAR.Y + Oxidation (M)
2272.1853 2271.1780 2271.1343 19.3 111 - 133 0 K.GVVPLAGTNGETTTQGLDGLSER.C
3113.6753 3112.6680 3112.6379 9.67 60 - 86 1 R.QLLLTADDRVNPCIGGVILFHETLYQK.A
3176.6453 3175.6380 3175.5972 12.9 173 - 200 1 R.YASICQQNGIVPIVEPEILPDGDHDLKR.C
No match to: 854.3170, 876.3025, 914.5256, 1033.5979, 1231.6539, 1269.7175, 1408.7529, 1536.8516, 1608.7865, 1609.7772, 1615.9065, 1643.8455,
1665.8262, 1700.8666, 1718.9393, 1763.8304, 1791.9521, 1799.9424, 1807.9392, 1820.8546, 1823.9868, 1840.9702, 1864.9916, 1865.9926, 1905.0056,
2027.1447, 2075.1184, 2139.1208, 2151.1465, 2166.1360, 2180.1448, 2333.2385, 2608.2981, 2615.3550, 2666.3325, 2879.4438, 2927.4353, 2943.4583,
2985.4792, 3022.5537, 3054.6343, 3096.6506, 3170.7014, 3177.6328, 3205.6589, 3210.7000, 3225.7222, 3234.6455, 3412.8198, 3596.8699, 3644.8401,
3661.8950, 3761.9360, 3874.9299
8. gi|6730618 Mass: 39586 Score: 93 Expect: 0.00051 Matches: 11
Chain A, Rabbit Muscle Aldolase AFRUCTOSE-1,6-Bisphosphate Complex
Observed Mr(expt) Mr(calc) ppm Start End Miss Peptide
1093.5890 1092.5817 1092.5563 23.2 322 - 330 1 K.AAQEEYVKR.A
1342.7362 1341.7289 1341.7041 18.5 87 - 98 0 K.ADDGRPFPQVIK.S
1671.8793 1670.8720 1670.8702 1.11 201 - 214 1 R.CQYVTEKVLAAVYK.A
1691.8704 1690.8631 1690.8348 16.7 243 - 257 0 K.YSHEEIAMATVTALR.R
1808.9792 1807.9719 1807.9443 15.3 289 - 303 0 K.CPLLKPWALTFSYGR.A
1847.9763 1846.9690 1846.9359 17.9 243 - 258 1 K.YSHEEIAMATVTALRR.T
2088.1143 2087.1070 2087.0874 9.41 69 - 86 0 R.VNPCIGGVILFHETLYQK.A
2123.1194 2122.1121 2122.0840 13.2 153 - 172 0 K.IGEHTPSALAIMENANVLAR.Y + Oxidation (M)
2272.1853 2271.1780 2271.1343 19.3 111 - 133 0 K.GVVPLAGTNGETTTQGLDGLSER.C
3113.6753 3112.6680 3112.6379 9.67 60 - 86 1 R.QLLLTADDRVNPCIGGVILFHETLYQK.A
3176.6453 3175.6380 3175.5972 12.9 173 - 200 1 R.YASICQQNGIVPIVEPEILPDGDHDLKR.C
No match to: 854.3170, 876.3025, 914.5256, 1033.5979, 1231.6539, 1269.7175, 1408.7529, 1536.8516, 1608.7865, 1609.7772, 1615.9065, 1643.8455,

1665.8262, 1700.8666, 1718.9393, 1763.8304, 1791.9521, 1799.9424, 1807.9392, 1820.8546, 1823.9868, 1840.9702, 1864.9916, 1865.9926, 1905.0056,
2027.1447, 2075.1184, 2139.1208, 2151.1465, 2166.1360, 2180.1448, 2333.2385, 2608.2981, 2615.3550, 2666.3325, 2879.4438, 2927.4353, 2943.4583,
2985.4792, 3022.5537, 3054.6343, 3096.6506, 3170.7014, 3177.6328, 3205.6589, 3210.7000, 3225.7222, 3234.6455, 3412.8198, 3596.8699, 3644.8401,
3661.8950, 3761.9360, 3874.9299
9. gi|67464529 Mass: 39643 Score: 93 Expect: 0.00051 Matches: 11
Chain A, Fructose-1,6-Bisphosphate Aldolase From Rabbit Muscle
Observed Mr(expt) Mr(calc) ppm Start End Miss Peptide
1093.5890 1092.5817 1092.5563 23.2 322 - 330 1 K.AAQEEYVKR.A
1342.7362 1341.7289 1341.7041 18.5 87 - 98 0 K.ADDGRPFPQVIK.S
1671.8793 1670.8720 1670.8702 1.11 201 - 214 1 R.CQYVTEKVLAAVYK.A
1691.8704 1690.8631 1690.8348 16.7 243 - 257 0 K.YSHEEIAMATVTALR.R
1808.9792 1807.9719 1807.9443 15.3 289 - 303 0 K.CPLLKPWALTFSYGR.A
1847.9763 1846.9690 1846.9359 17.9 243 - 258 1 K.YSHEEIAMATVTALRR.T
2088.1143 2087.1070 2087.0874 9.41 69 - 86 0 R.VNPCIGGVILFHETLYQK.A
2123.1194 2122.1121 2122.0840 13.2 153 - 172 0 K.IGEHTPSALAIMENANVLAR.Y + Oxidation (M)
2272.1853 2271.1780 2271.1343 19.3 111 - 133 0 K.GVVPLAGTNGETTTQGLDGLSER.C
3113.6753 3112.6680 3112.6379 9.67 60 - 86 1 R.QLLLTADDRVNPCIGGVILFHETLYQK.A
3176.6453 3175.6380 3175.5972 12.9 173 - 200 1 R.YASICQQNGIVPIVEPEILPDGDHDLKR.C
No match to: 854.3170, 876.3025, 914.5256, 1033.5979, 1231.6539, 1269.7175, 1408.7529, 1536.8516, 1608.7865, 1609.7772, 1615.9065, 1643.8455,
1665.8262, 1700.8666, 1718.9393, 1763.8304, 1791.9521, 1799.9424, 1807.9392, 1820.8546, 1823.9868, 1840.9702, 1864.9916, 1865.9926, 1905.0056,
2027.1447, 2075.1184, 2139.1208, 2151.1465, 2166.1360, 2180.1448, 2333.2385, 2608.2981, 2615.3550, 2666.3325, 2879.4438, 2927.4353, 2943.4583,
2985.4792, 3022.5537, 3054.6343, 3096.6506, 3170.7014, 3177.6328, 3205.6589, 3210.7000, 3225.7222, 3234.6455, 3412.8198, 3596.8699, 3644.8401,
3661.8950, 3761.9360, 3874.9299
10. gi|158430448 Mass: 39646 Score: 93 Expect: 0.00051 Matches: 11
Chain A, Dihydroxyacetone Phosphate Schiff Base Intermediate In Mutant Fructose-1,6-Bisphosphate Aldolase From Rabbit Muscle
Observed Mr(expt) Mr(calc) ppm Start End Miss Peptide
1093.5890 1092.5817 1092.5563 23.2 322 - 330 1 K.AAQEEYVKR.A
1342.7362 1341.7289 1341.7041 18.5 87 - 98 0 K.ADDGRPFPQVIK.S
1671.8793 1670.8720 1670.8702 1.11 201 - 214 1 R.CQYVTEKVLAAVYK.A
1691.8704 1690.8631 1690.8348 16.7 243 - 257 0 K.YSHEEIAMATVTALR.R
1808.9792 1807.9719 1807.9443 15.3 289 - 303 0 K.CPLLKPWALTFSYGR.A
1847.9763 1846.9690 1846.9359 17.9 243 - 258 1 K.YSHEEIAMATVTALRR.T
2088.1143 2087.1070 2087.0874 9.41 69 - 86 0 R.VNPCIGGVILFHETLYQK.A
2123.1194 2122.1121 2122.0840 13.2 153 - 172 0 K.IGEHTPSALAIMENANVLAR.Y + Oxidation (M)
2272.1853 2271.1780 2271.1343 19.3 111 - 133 0 K.GVVPLAGTNGETTTQGLDGLSER.C
3113.6753 3112.6680 3112.6379 9.67 60 - 86 1 R.QLLLTADDRVNPCIGGVILFHETLYQK.A
3176.6453 3175.6380 3175.5972 12.9 173 - 200 1 R.YASICQQNGIVPIVEPEILPDGDHDLKR.C
No match to: 854.3170, 876.3025, 914.5256, 1033.5979, 1231.6539, 1269.7175, 1408.7529, 1536.8516, 1608.7865, 1609.7772, 1615.9065, 1643.8455,
1665.8262, 1700.8666, 1718.9393, 1763.8304, 1791.9521, 1799.9424, 1807.9392, 1820.8546, 1823.9868, 1840.9702, 1864.9916, 1865.9926, 1905.0056,
2027.1447, 2075.1184, 2139.1208, 2151.1465, 2166.1360, 2180.1448, 2333.2385, 2608.2981, 2615.3550, 2666.3325, 2879.4438, 2927.4353, 2943.4583,
2985.4792, 3022.5537, 3054.6343, 3096.6506, 3170.7014, 3177.6328, 3205.6589, 3210.7000, 3225.7222, 3234.6455, 3412.8198, 3596.8699, 3644.8401,
3661.8950, 3761.9360, 3874.9299
11. gi|228311978 Mass: 39642 Score: 93 Expect: 0.00051 Matches: 11
Chain A, D33n Mutant Fructose-1,6-Bisphosphate Aldolase From Rabbit Muscle
Observed Mr(expt) Mr(calc) ppm Start End Miss Peptide
1093.5890 1092.5817 1092.5563 23.2 322 - 330 1 K.AAQEEYVKR.A
1342.7362 1341.7289 1341.7041 18.5 87 - 98 0 K.ADDGRPFPQVIK.S
1671.8793 1670.8720 1670.8702 1.11 201 - 214 1 R.CQYVTEKVLAAVYK.A
1691.8704 1690.8631 1690.8348 16.7 243 - 257 0 K.YSHEEIAMATVTALR.R
1808.9792 1807.9719 1807.9443 15.3 289 - 303 0 K.CPLLKPWALTFSYGR.A
1847.9763 1846.9690 1846.9359 17.9 243 - 258 1 K.YSHEEIAMATVTALRR.T
2088.1143 2087.1070 2087.0874 9.41 69 - 86 0 R.VNPCIGGVILFHETLYQK.A
2123.1194 2122.1121 2122.0840 13.2 153 - 172 0 K.IGEHTPSALAIMENANVLAR.Y + Oxidation (M)
2272.1853 2271.1780 2271.1343 19.3 111 - 133 0 K.GVVPLAGTNGETTTQGLDGLSER.C
3113.6753 3112.6680 3112.6379 9.67 60 - 86 1 R.QLLLTADDRVNPCIGGVILFHETLYQK.A
3176.6453 3175.6380 3175.5972 12.9 173 - 200 1 R.YASICQQNGIVPIVEPEILPDGDHDLKR.C
No match to: 854.3170, 876.3025, 914.5256, 1033.5979, 1231.6539, 1269.7175, 1408.7529, 1536.8516, 1608.7865, 1609.7772, 1615.9065, 1643.8455,
1665.8262, 1700.8666, 1718.9393, 1763.8304, 1791.9521, 1799.9424, 1807.9392, 1820.8546, 1823.9868, 1840.9702, 1864.9916, 1865.9926, 1905.0056,
2027.1447, 2075.1184, 2139.1208, 2151.1465, 2166.1360, 2180.1448, 2333.2385, 2608.2981, 2615.3550, 2666.3325, 2879.4438, 2927.4353, 2943.4583,
2985.4792, 3022.5537, 3054.6343, 3096.6506, 3170.7014, 3177.6328, 3205.6589, 3210.7000, 3225.7222, 3234.6455, 3412.8198, 3596.8699, 3644.8401,
3661.8950, 3761.9360, 3874.9299
12. gi|228311995 Mass: 39615 Score: 93 Expect: 0.00051 Matches: 11
Chain A, D33s Mutant Fructose-1,6-Bisphosphate Aldolase From Rabbit Muscle
Observed Mr(expt) Mr(calc) ppm Start End Miss Peptide
1093.5890 1092.5817 1092.5563 23.2 322 - 330 1 K.AAQEEYVKR.A
1342.7362 1341.7289 1341.7041 18.5 87 - 98 0 K.ADDGRPFPQVIK.S
1671.8793 1670.8720 1670.8702 1.11 201 - 214 1 R.CQYVTEKVLAAVYK.A
1691.8704 1690.8631 1690.8348 16.7 243 - 257 0 K.YSHEEIAMATVTALR.R
1808.9792 1807.9719 1807.9443 15.3 289 - 303 0 K.CPLLKPWALTFSYGR.A

1847.9763 1846.9690 1846.9359 17.9 243 - 258 1 K.YSHEEIAMATVTALRR.T
2088.1143 2087.1070 2087.0874 9.41 69 - 86 0 R.VNPCIGGVILFHETLYQK.A
2123.1194 2122.1121 2122.0840 13.2 153 - 172 0 K.IGEHTPSALAIMENANVLAR.Y + Oxidation (M)
2272.1853 2271.1780 2271.1343 19.3 111 - 133 0 K.GVVPLAGTNGETTTQGLDGLSER.C
3113.6753 3112.6680 3112.6379 9.67 60 - 86 1 R.QLLLTADDRVNPCIGGVILFHETLYQK.A
3176.6453 3175.6380 3175.5972 12.9 173 - 200 1 R.YASICQQNGIVPIVEPEILPDGDHDLKR.C
No match to: 854.3170, 876.3025, 914.5256, 1033.5979, 1231.6539, 1269.7175, 1408.7529, 1536.8516, 1608.7865, 1609.7772, 1615.9065, 1643.8455,
1665.8262, 1700.8666, 1718.9393, 1763.8304, 1791.9521, 1799.9424, 1807.9392, 1820.8546, 1823.9868, 1840.9702, 1864.9916, 1865.9926, 1905.0056,
2027.1447, 2075.1184, 2139.1208, 2151.1465, 2166.1360, 2180.1448, 2333.2385, 2608.2981, 2615.3550, 2666.3325, 2879.4438, 2927.4353, 2943.4583,
2985.4792, 3022.5537, 3054.6343, 3096.6506, 3170.7014, 3177.6328, 3205.6589, 3210.7000, 3225.7222, 3234.6455, 3412.8198, 3596.8699, 3644.8401,
3661.8950, 3761.9360, 3874.9299
13. gi|126722869 Mass: 39774 Score: 93 Expect: 0.00053 Matches: 11
fructose-bisphosphate aldolase A [Oryctolagus cuniculus]
Observed Mr(expt) Mr(calc) ppm Start End Miss Peptide
1093.5890 1092.5817 1092.5563 23.2 323 - 331 1 K.AAQEEYVKR.A
1342.7362 1341.7289 1341.7041 18.5 88 - 99 0 K.ADDGRPFPQVIK.S
1671.8793 1670.8720 1670.8702 1.11 202 - 215 1 R.CQYVTEKVLAAVYK.A
1691.8704 1690.8631 1690.8348 16.7 244 - 258 0 K.YSHEEIAMATVTALR.R
1808.9792 1807.9719 1807.9443 15.3 290 - 304 0 K.CPLLKPWALTFSYGR.A
1847.9763 1846.9690 1846.9359 17.9 244 - 259 1 K.YSHEEIAMATVTALRR.T
2088.1143 2087.1070 2087.0874 9.41 70 - 87 0 R.VNPCIGGVILFHETLYQK.A
2123.1194 2122.1121 2122.0840 13.2 154 - 173 0 K.IGEHTPSALAIMENANVLAR.Y + Oxidation (M)
2272.1853 2271.1780 2271.1343 19.3 112 - 134 0 K.GVVPLAGTNGETTTQGLDGLSER.C
3113.6753 3112.6680 3112.6379 9.67 61 - 87 1 R.QLLLTADDRVNPCIGGVILFHETLYQK.A
3176.6453 3175.6380 3175.5972 12.9 174 - 201 1 R.YASICQQNGIVPIVEPEILPDGDHDLKR.C
No match to: 854.3170, 876.3025, 914.5256, 1033.5979, 1231.6539, 1269.7175, 1408.7529, 1536.8516, 1608.7865, 1609.7772, 1615.9065, 1643.8455,
1665.8262, 1700.8666, 1718.9393, 1763.8304, 1791.9521, 1799.9424, 1807.9392, 1820.8546, 1823.9868, 1840.9702, 1864.9916, 1865.9926, 1905.0056,
2027.1447, 2075.1184, 2139.1208, 2151.1465, 2166.1360, 2180.1448, 2333.2385, 2608.2981, 2615.3550, 2666.3325, 2879.4438, 2927.4353, 2943.4583,
2985.4792, 3022.5537, 3054.6343, 3096.6506, 3170.7014, 3177.6328, 3205.6589, 3210.7000, 3225.7222, 3234.6455, 3412.8198, 3596.8699, 3644.8401,
3661.8950, 3761.9360, 3874.9299
14. gi|4557305 Mass: 39851 Score: 93 Expect: 0.00053 Matches: 11
fructose-bisphosphate aldolase A isoform 1 [Homo sapiens]
Observed Mr(expt) Mr(calc) ppm Start End Miss Peptide
1093.5890 1092.5817 1092.5563 23.2 323 - 331 1 K.AAQEEYVKR.A
1342.7362 1341.7289 1341.7041 18.5 88 - 99 0 K.ADDGRPFPQVIK.S
1671.8793 1670.8720 1670.8702 1.11 202 - 215 1 R.CQYVTEKVLAAVYK.A
1691.8704 1690.8631 1690.8348 16.7 244 - 258 0 K.FSHEEIAMATVTALR.R + Oxidation (M)
1808.9792 1807.9719 1807.9443 15.3 290 - 304 0 K.CPLLKPWALTFSYGR.A
1847.9763 1846.9690 1846.9359 17.9 244 - 259 1 K.FSHEEIAMATVTALRR.T + Oxidation (M)
2088.1143 2087.1070 2087.0874 9.41 70 - 87 0 R.VNPCIGGVILFHETLYQK.A
2123.1194 2122.1121 2122.0840 13.2 154 - 173 0 K.IGEHTPSALAIMENANVLAR.Y + Oxidation (M)
2272.1853 2271.1780 2271.1343 19.3 112 - 134 0 K.GVVPLAGTNGETTTQGLDGLSER.C
3113.6753 3112.6680 3112.6379 9.67 61 - 87 1 R.QLLLTADDRVNPCIGGVILFHETLYQK.A
3176.6453 3175.6380 3175.5972 12.9 174 - 201 1 R.YASICQQNGIVPIVEPEILPDGDHDLKR.C
No match to: 854.3170, 876.3025, 914.5256, 1033.5979, 1231.6539, 1269.7175, 1408.7529, 1536.8516, 1608.7865, 1609.7772, 1615.9065, 1643.8455,
1665.8262, 1700.8666, 1718.9393, 1763.8304, 1791.9521, 1799.9424, 1807.9392, 1820.8546, 1823.9868, 1840.9702, 1864.9916, 1865.9926, 1905.0056,
2027.1447, 2075.1184, 2139.1208, 2151.1465, 2166.1360, 2180.1448, 2333.2385, 2608.2981, 2615.3550, 2666.3325, 2879.4438, 2927.4353, 2943.4583,
2985.4792, 3022.5537, 3054.6343, 3096.6506, 3170.7014, 3177.6328, 3205.6589, 3210.7000, 3225.7222, 3234.6455, 3412.8198, 3596.8699, 3644.8401,
3661.8950, 3761.9360, 3874.9299
15. gi|90078570 Mass: 39860 Score: 93 Expect: 0.00053 Matches: 11
unnamed protein product [Macaca fascicularis]
Observed Mr(expt) Mr(calc) ppm Start End Miss Peptide
1093.5890 1092.5817 1092.5563 23.2 323 - 331 1 K.AAQEEYVKR.A
1342.7362 1341.7289 1341.7041 18.5 88 - 99 0 K.ADDGRPFPQVIK.S
1671.8793 1670.8720 1670.8702 1.11 202 - 215 1 R.CQYVTEKVLAAVYK.A
1691.8704 1690.8631 1690.8348 16.7 244 - 258 0 K.FSHEEIAMATVTALR.R + Oxidation (M)
1808.9792 1807.9719 1807.9443 15.3 290 - 304 0 K.CPLLKPWALTFSYGR.A
1847.9763 1846.9690 1846.9359 17.9 244 - 259 1 K.FSHEEIAMATVTALRR.T + Oxidation (M)
2088.1143 2087.1070 2087.0874 9.41 70 - 87 0 R.VNPCIGGVILFHETLYQK.A
2123.1194 2122.1121 2122.0840 13.2 154 - 173 0 K.IGEHTPSALAIMENANVLAR.Y + Oxidation (M)
2272.1853 2271.1780 2271.1343 19.3 112 - 134 0 K.GVVPLAGTNGETTTQGLDGLSER.C
3113.6753 3112.6680 3112.6379 9.67 61 - 87 1 R.QLLLTADDRVNPCIGGVILFHETLYQK.A
3176.6453 3175.6380 3175.5972 12.9 174 - 201 1 R.YASICQQNGIVPIVEPEILPDGDHDLKR.C
No match to: 854.3170, 876.3025, 914.5256, 1033.5979, 1231.6539, 1269.7175, 1408.7529, 1536.8516, 1608.7865, 1609.7772, 1615.9065, 1643.8455,
1665.8262, 1700.8666, 1718.9393, 1763.8304, 1791.9521, 1799.9424, 1807.9392, 1820.8546, 1823.9868, 1840.9702, 1864.9916, 1865.9926, 1905.0056,
2027.1447, 2075.1184, 2139.1208, 2151.1465, 2166.1360, 2180.1448, 2333.2385, 2608.2981, 2615.3550, 2666.3325, 2879.4438, 2927.4353, 2943.4583,
2985.4792, 3022.5537, 3054.6343, 3096.6506, 3170.7014, 3177.6328, 3205.6589, 3210.7000, 3225.7222, 3234.6455, 3412.8198, 3596.8699, 3644.8401,
3661.8950, 3761.9360, 3874.9299
16. gi|49456715 Mass: 39880 Score: 93 Expect: 0.00056 Matches: 11

ALDOA [Homo sapiens]
Observed Mr(expt) Mr(calc) ppm Start End Miss Peptide
1093.5890 1092.5817 1092.5563 23.2 323 - 331 1 K.AAQEEYVKR.A
1342.7362 1341.7289 1341.7041 18.5 88 - 99 0 K.ADDGRPFPQVIK.S
1671.8793 1670.8720 1670.8702 1.11 202 - 215 1 R.CQYVTEKVLAAVYK.A
1691.8704 1690.8631 1690.8348 16.7 244 - 258 0 K.FSHEEIAMATVTALR.R + Oxidation (M)
1808.9792 1807.9719 1807.9443 15.3 290 - 304 0 K.CPLLKPWALTFSYGR.A
1847.9763 1846.9690 1846.9359 17.9 244 - 259 1 K.FSHEEIAMATVTALRR.T + Oxidation (M)
2088.1143 2087.1070 2087.0874 9.41 70 - 87 0 R.VNPCIGGVILFHETLYQK.A
2123.1194 2122.1121 2122.0840 13.2 154 - 173 0 K.IGEHTPSALAIMENANVLAR.Y + Oxidation (M)
2272.1853 2271.1780 2271.1343 19.3 112 - 134 0 K.GVVPLAGTNGETTTQGLDGLSER.C
2985.4792 2984.4719 2984.4960 -8.06 154 - 180 1 K.IGEHTPSALAIMENANVLARYASICQR.N
3113.6753 3112.6680 3112.6379 9.67 61 - 87 1 R.QLLLTADDRVNPCIGGVILFHETLYQK.A
No match to: 854.3170, 876.3025, 914.5256, 1033.5979, 1231.6539, 1269.7175, 1408.7529, 1536.8516, 1608.7865, 1609.7772, 1615.9065, 1643.8455,
1665.8262, 1700.8666, 1718.9393, 1763.8304, 1791.9521, 1799.9424, 1807.9392, 1820.8546, 1823.9868, 1840.9702, 1864.9916, 1865.9926, 1905.0056,
2027.1447, 2075.1184, 2139.1208, 2151.1465, 2166.1360, 2180.1448, 2333.2385, 2608.2981, 2615.3550, 2666.3325, 2879.4438, 2927.4353, 2943.4583,
3022.5537, 3054.6343, 3096.6506, 3170.7014, 3176.6453, 3177.6328, 3205.6589, 3210.7000, 3225.7222, 3234.6455, 3412.8198, 3596.8699, 3644.8401,
3661.8950, 3761.9360, 3874.9299
17. gi|338712747 Mass: 45488 Score: 88 Expect: 0.0018 Matches: 12
PREDICTED: fructose-bisphosphate aldolase A isoform 4 [Equus caballus]
Observed Mr(expt) Mr(calc) ppm Start End Miss Peptide
1093.5890 1092.5817 1092.5563 23.2 375 - 383 1 K.AAQEEYVKR.A
1342.7362 1341.7289 1341.7041 18.5 140 - 151 0 K.ADDGRPFPQVIK.S
1671.8793 1670.8720 1670.8702 1.11 254 - 267 1 R.CQYVTEKVLAAVYK.A
1691.8704 1690.8631 1690.8348 16.7 296 - 310 0 K.YSHEEIAMATVTALR.R
1808.9792 1807.9719 1807.9443 15.3 342 - 356 0 K.CPLLKPWALTFSYGR.A
1847.9763 1846.9690 1846.9359 17.9 296 - 311 1 K.YSHEEIAMATVTALRR.T
2088.1143 2087.1070 2087.0874 9.41 122 - 139 0 R.VNPCIGGVILFHETLYQK.A
2123.1194 2122.1121 2122.0840 13.2 206 - 225 0 K.IGEHTPSSLAIMENANVLAR.Y
2139.1208 2138.1135 2138.0790 16.2 206 - 225 0 K.IGEHTPSSLAIMENANVLAR.Y + Oxidation (M)
2272.1853 2271.1780 2271.1343 19.3 164 - 186 0 K.GVVPLAGTNGETTTQGLDGLSER.C
3113.6753 3112.6680 3112.6379 9.67 113 - 139 1 R.QLLLTADDRVNPCIGGVILFHETLYQK.A
3176.6453 3175.6380 3175.5972 12.9 226 - 253 1 R.YASICQQNGIVPIVEPEILPDGDHDLKR.C
No match to: 854.3170, 876.3025, 914.5256, 1033.5979, 1231.6539, 1269.7175, 1408.7529, 1536.8516, 1608.7865, 1609.7772, 1615.9065, 1643.8455,
1665.8262, 1700.8666, 1718.9393, 1763.8304, 1791.9521, 1799.9424, 1807.9392, 1820.8546, 1823.9868, 1840.9702, 1864.9916, 1865.9926, 1905.0056,
2027.1447, 2075.1184, 2151.1465, 2166.1360, 2180.1448, 2333.2385, 2608.2981, 2615.3550, 2666.3325, 2879.4438, 2927.4353, 2943.4583, 2985.4792,
3022.5537, 3054.6343, 3096.6506, 3170.7014, 3177.6328, 3205.6589, 3210.7000, 3225.7222, 3234.6455, 3412.8198, 3596.8699, 3644.8401, 3661.8950,
3761.9360, 3874.9299
18. gi|342187211 Mass: 45688 Score: 87 Expect: 0.0022 Matches: 11
fructose-bisphosphate aldolase A isoform 2 [Homo sapiens]
Observed Mr(expt) Mr(calc) ppm Start End Miss Peptide
1093.5890 1092.5817 1092.5563 23.2 377 - 385 1 K.AAQEEYVKR.A
1342.7362 1341.7289 1341.7041 18.5 142 - 153 0 K.ADDGRPFPQVIK.S
1671.8793 1670.8720 1670.8702 1.11 256 - 269 1 R.CQYVTEKVLAAVYK.A
1691.8704 1690.8631 1690.8348 16.7 298 - 312 0 K.FSHEEIAMATVTALR.R + Oxidation (M)
1808.9792 1807.9719 1807.9443 15.3 344 - 358 0 K.CPLLKPWALTFSYGR.A
1847.9763 1846.9690 1846.9359 17.9 298 - 313 1 K.FSHEEIAMATVTALRR.T + Oxidation (M)
2088.1143 2087.1070 2087.0874 9.41 124 - 141 0 R.VNPCIGGVILFHETLYQK.A
2123.1194 2122.1121 2122.0840 13.2 208 - 227 0 K.IGEHTPSALAIMENANVLAR.Y + Oxidation (M)
2272.1853 2271.1780 2271.1343 19.3 166 - 188 0 K.GVVPLAGTNGETTTQGLDGLSER.C
3113.6753 3112.6680 3112.6379 9.67 115 - 141 1 R.QLLLTADDRVNPCIGGVILFHETLYQK.A
3176.6453 3175.6380 3175.5972 12.9 228 - 255 1 R.YASICQQNGIVPIVEPEILPDGDHDLKR.C
No match to: 854.3170, 876.3025, 914.5256, 1033.5979, 1231.6539, 1269.7175, 1408.7529, 1536.8516, 1608.7865, 1609.7772, 1615.9065, 1643.8455,
1665.8262, 1700.8666, 1718.9393, 1763.8304, 1791.9521, 1799.9424, 1807.9392, 1820.8546, 1823.9868, 1840.9702, 1864.9916, 1865.9926, 1905.0056,
2027.1447, 2075.1184, 2139.1208, 2151.1465, 2166.1360, 2180.1448, 2333.2385, 2608.2981, 2615.3550, 2666.3325, 2879.4438, 2927.4353, 2943.4583,
2985.4792, 3022.5537, 3054.6343, 3096.6506, 3170.7014, 3177.6328, 3205.6589, 3210.7000, 3225.7222, 3234.6455, 3412.8198, 3596.8699, 3644.8401,
3661.8950, 3761.9360, 3874.9299
19. gi|332265962 Mass: 45656 Score: 87 Expect: 0.0022 Matches: 11
PREDICTED: fructose-bisphosphate aldolase A isoform 4 [Nomascus leucogenys]
Observed Mr(expt) Mr(calc) ppm Start End Miss Peptide
1093.5890 1092.5817 1092.5563 23.2 377 - 385 1 K.AAQEEYVKR.A
1342.7362 1341.7289 1341.7041 18.5 142 - 153 0 K.ADDGRPFPQVIK.S
1671.8793 1670.8720 1670.8702 1.11 256 - 269 1 R.CQYVTEKVLAAVYK.A
1691.8704 1690.8631 1690.8348 16.7 298 - 312 0 K.FSHEEIAMATVTALR.R + Oxidation (M)
1808.9792 1807.9719 1807.9443 15.3 344 - 358 0 K.CPLLKPWALTFSYGR.A
1847.9763 1846.9690 1846.9359 17.9 298 - 313 1 K.FSHEEIAMATVTALRR.T + Oxidation (M)
2088.1143 2087.1070 2087.0874 9.41 124 - 141 0 R.VNPCIGGVILFHETLYQK.A
2123.1194 2122.1121 2122.0840 13.2 208 - 227 0 K.IGEHTPSALAIMENANVLAR.Y + Oxidation (M)
2272.1853 2271.1780 2271.1343 19.3 166 - 188 0 K.GVVPLAGTNGETTTQGLDGLSER.C
3113.6753 3112.6680 3112.6379 9.67 115 - 141 1 R.QLLLTADDRVNPCIGGVILFHETLYQK.A
3176.6453 3175.6380 3175.5972 12.9 228 - 255 1 R.YASICQQNGIVPIVEPEILPDGDHDLKR.C
No match to: 854.3170, 876.3025, 914.5256, 1033.5979, 1231.6539, 1269.7175, 1408.7529, 1536.8516, 1608.7865, 1609.7772, 1615.9065, 1643.8455,

1665.8262, 1700.8666, 1718.9393, 1763.8304, 1791.9521, 1799.9424, 1807.9392, 1820.8546, 1823.9868, 1840.9702, 1864.9916, 1865.9926, 1905.0056,
2027.1447, 2075.1184, 2139.1208, 2151.1465, 2166.1360, 2180.1448, 2333.2385, 2608.2981, 2615.3550, 2666.3325, 2879.4438, 2927.4353, 2943.4583,
2985.4792, 3022.5537, 3054.6343, 3096.6506, 3170.7014, 3177.6328, 3205.6589, 3210.7000, 3225.7222, 3234.6455, 3412.8198, 3596.8699, 3644.8401,
3661.8950, 3761.9360, 3874.9299

20. gi|355756689 Mass: 45711 Score: 87 Expect: 0.0022 Matches: 11
Fructose-bisphosphate aldolase A [Macaca fascicularis]
Observed Mr(expt) Mr(calc) ppm Start End Miss Peptide
1093.5890 1092.5817 1092.5563 23.2 377 - 385 1 K.AAQEEYVKR.A
1342.7362 1341.7289 1341.7041 18.5 142 - 153 0 K.ADDGRPFPQVIK.S
1671.8793 1670.8720 1670.8702 1.11 256 - 269 1 R.CQYVTEKVLAAVYK.A
1691.8704 1690.8631 1690.8348 16.7 298 - 312 0 K.FSHEEIAMATVTALR.R + Oxidation (M)
1808.9792 1807.9719 1807.9443 15.3 344 - 358 0 K.CPLLKPWALTFSYGR.A
1847.9763 1846.9690 1846.9359 17.9 298 - 313 1 K.FSHEEIAMATVTALRR.T + Oxidation (M)
2088.1143 2087.1070 2087.0874 9.41 124 - 141 0 R.VNPCIGGVILFHETLYQK.A
2123.1194 2122.1121 2122.0840 13.2 208 - 227 0 K.IGEHTPSALAIMENANVLAR.Y + Oxidation (M)
2272.1853 2271.1780 2271.1343 19.3 166 - 188 0 K.GVVPLAGTNGETTTQGLDGLSER.C
3113.6753 3112.6680 3112.6379 9.67 115 - 141 1 R.QLLLTADDRVNPCIGGVILFHETLYQK.A
3176.6453 3175.6380 3175.5972 12.9 228 - 255 1 R.YASICQQNGIVPIVEPEILPDGDHDLKR.C
No match to: 854.3170, 876.3025, 914.5256, 1033.5979, 1231.6539, 1269.7175, 1408.7529, 1536.8516, 1608.7865, 1609.7772, 1615.9065, 1643.8455,
1665.8262, 1700.8666, 1718.9393, 1763.8304, 1791.9521, 1799.9424, 1807.9392, 1820.8546, 1823.9868, 1840.9702, 1864.9916, 1865.9926, 1905.0056,
2027.1447, 2075.1184, 2139.1208, 2151.1465, 2166.1360, 2180.1448, 2333.2385, 2608.2981, 2615.3550, 2666.3325, 2879.4438, 2927.4353, 2943.4583,
2985.4792, 3022.5537, 3054.6343, 3096.6506, 3170.7014, 3177.6328, 3205.6589, 3210.7000, 3225.7222, 3234.6455, 3412.8198, 3596.8699, 3644.8401,
3661.8950, 3761.9360, 3874.9299

Search Parameters

Type of search : Peptide Mass Fingerprint
Enzyme : Trypsin
Fixed modifications : Carbamidomethyl (C)
Variable modifications : Oxidation (M)
Mass values : Monoisotopic
Protein Mass : Unrestricted
Peptide Mass Tolerance : ± 50 ppm
Peptide Charge State : 1+
Max Missed Cleavages : 1
Number of queries : 65

Mascot: http://www.matrixscience.com/

COVERAGE BAND 5

Mascot Search Results

Protein View

Match to: gi|156120479 Score: 130 Expect: 1.1e-007
fructose-bisphosphate aldolase A [Bos taurus]
Nominal mass (Mr): 39925; Calculated pI value: 8.45
NCBI BLAST search of gi|156120479 against nr
Unformatted sequence string for pasting into other applications
Taxonomy: Bos taurus
Links to retrieve other entries containing this sequence from NCBI Entrez:
gi|151554749 from Bos taurus
gi|296473262 from Bos taurus
Fixed modifications: Carbamidomethyl (C)
Variable modifications: Oxidation (M)
Cleavage by Trypsin: cuts C-term side of KR unless next residue is P
Number of mass values searched: 65
Number of mass values matched: 15
Sequence Coverage: 53%
Matched peptides shown in **Bold Red**

1 MPHQYPALTP EQKKELCDIA HRIVAPGKGI LAADESTGSI AKRLQSIGTE
51 NTEENRRFYR QLLLTADDRV NPCIGGVILF HETLYQKADD GRPFPQVIKA
101 KGGVVGIKVD KGVVPLAGTN GETTTQGLDG LSERCAQYKK DGADFAKWRC
151 VLKIGEHTPS SLAIMENANV LARYASICQQ NGIVPIVEPE ILPDGDHDLK
201 RCQYVTEKVL AAVYKALSDH HIYLEGTLLK PNMVTPGHAC TQKYSHEEIA
251 MATVTALRRT VPPAVPGITF LSGGQSEEEA SINLNAINKC PLLKPWALTF
301 SYGRALQASA LKAWGGKKEN LKAAQEEYVK RALANSLACQ GKYTPSGKAG
351 AAASESLFIS NHAY

Show predicted peptides also
Sort Peptides By Residue Number Increasing Mass Decreasing Mass

Start - End Observed Mr(expt) Mr(calc) ppm Miss Sequence
2 - 13 1408.7529 1407.7456 1407.7146 22 0 M.PHQYPALTPEQK.K
2 - 14 1536.8516 1535.8443 1535.8096 23 1 M.PHQYPALTPEQKK.E
61 - 87 3113.6753 3112.6680 3112.6379 10 1 R.QLLLTADDRVNPCIGGVILFHETLYQK.A
70 - 87 2088.1143 2087.1070 2087.0874 9 0 R.VNPCIGGVILFHETLYQK.A
88 - 99 1342.7362 1341.7289 1341.7041 19 0 K.ADDGRPFPQVIK.A

112 - 134 2272.1853 2271.1780 2271.1343 19 0 K.GVVPLAGTNGETTTQGLDGLSER.C
154 - 173 2123.1194 2122.1121 2122.0840 13 0 K.IGEHTPSSLAIMENANVLAR.Y
154 - 173 2139.1208 2138.1135 2138.0790 16 0 K.IGEHTPSSLAIMENANVLAR.Y Oxidation (M)
174 - 201 3176.6453 3175.6380 3175.5972 13 1 R.YASICQQNGIVPIVEPEILPDGDHDLKR.C
202 - 215 1671.8793 1670.8720 1670.8702 1 1 R.CQYVTEKVLAAVYK.A
244 - 258 1691.8704 1690.8631 1690.8348 17 0 K.YSHEEIAMATVTALR.R
244 - 259 1847.9763 1846.9690 1846.9359 18 1 K.YSHEEIAMATVTALRR.T
290 - 304 1808.9792 1807.9719 1807.9443 15 0 K.CPLLKPWALTFSYGR.A
323 - 331 1093.5890 1092.5817 1092.5563 23 1 K.AAQEEYVKR.A
349 - 364 1608.7865 1607.7792 1607.7579 13 0 K.AGAAASESLFISNHAY.-
No match to: 854.3170, 876.3025, 914.5256, 1033.5979, 1231.6539, 1269.7175, 1609.7772, 1615.9065, 1643.8455, 1665.8262, 1700.866

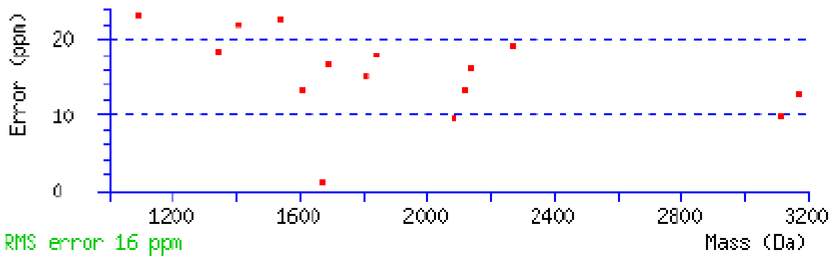

BAND 6

Mascot Search Results

User :
Email :
Search title : SampleSetID: 824, AnalysisID: 7242, MaldiWellID: 69628, SpectrumID: 154553, Path=\180719\MS\18-106 NCBI Mammalia
Database : NCBInr 20120508 (17919084 sequences; 6150218869 residues)
Taxonomy : Mammalia (mammals) (1061927 sequences)
Timestamp : 19 Jul 2018 at 13:01:08 GMT
Top Score : 139 for Mixture 1, gi|84000195 + gi|262263205

Mascot Score Histogram

Protein score is -10*Log(P), where P is the probability that the observed match is a random event.
Protein scores greater than 73 are significant (p<0.05).}

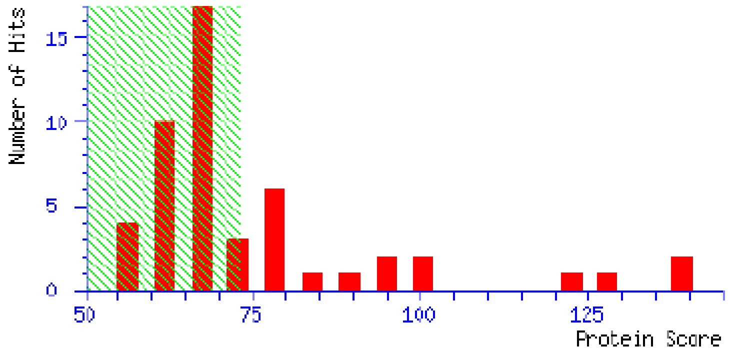

Protein Summary Report

Format As Protein Summary Help
Significance threshold p< 0.05 Max. number of hits 20
Re-Search All Search Unmatched

Index

Accession Mass Score Description
1. Mixture 1 139 gi|84000195 + gi|262263205
2. Mixture 2 139 gi|84000195 + gi|61888856
3. Mixture 3 126 gi|84000195 + gi|38512111
4. Mixture 4 121 gi|84000195 + gi|296439800
5. gi|84000195 28838 100 phosphoglycerate mutase 2 [Bos taurus]
6. gi|296475149 28852 100 phosphoglycerate mutase 2 [Bos taurus]
7. gi|262263205 26879 93 triosephosphate isomerase 1 [Sus scrofa]
8. gi|61888856 26901 93 triosephosphate isomerase [Bos taurus]
9. gi|348554958 30916 87 PREDICTED: triosephosphate isomerase-like [Cavia porcellus]
10. gi|201066358 28830 85 phosphoglycerate mutase 2 [Sus scrofa]
11. gi|90200404 26863 81 triosephosphate isomerase 1 [Sus scrofa]
12. gi|38512111 27214 80 Tpi1 protein, partial [Rattus norvegicus]

13. gi|538426 27417 80 triosephosphate isomerase [Rattus norvegicus]
14. gi|117935064 27345 80 triosephosphate isomerase [Rattus norvegicus]
15. gi|80971510 26941 77 triosephosphate isomerase [Sus scrofa]
16. gi|296439800 20636 77 RecName: Full=Triosephosphate isomerase; Short=TIM; AltName: Full=Triose-phosphate isomerase
17. gi|149049470 31676 75 rCG29914, isoform CRA_b [Rattus norvegicus]
18. gi|149704608 28773 74 PREDICTED: phosphoglycerate mutase 2-like [Equus caballus]
19. gi|291394897 28820 74 PREDICTED: phosphoglycerate mutase 2 [Oryctolagus cuniculus]
20. gi|999892 26807 69 Chain A, Crystal Structure Of Recombinant Human Triosephosphate Isomerase At 2.8 Angstroms Resolution. Triosephosphate Isomerase Related Hu

Results List

1. Mixture 1 Total score: 139 Expect: 1.3e-008 Matches: 25
Components: 1. gi|84000195 phosphoglycerate mutase 2 [Bos taurus]
2. gi|262263205 triosephosphate isomerase 1 [Sus scrofa]
Observed Mr(expt) Mr(calc) ppm Start End Miss Comp Peptide
954.5139 953.5066 953.4760 32.2 7 - 14 0 2 K.FFVGGNWK.M
1065.5342 1064.5269 1064.5138 12.3 232 - 240 0 1 R.FLGDEETVR.K
1082.5940 1081.5867 1081.5709 14.6 6 - 14 1 2 R.KFFVGGNWK.M
1150.6758 1149.6685 1149.6618 5.83 181 - 191 0 1 R.VLIAAHGNSLR.G
1357.5730 1356.5657 1356.5807 -11.02 11 - 21 0 1 R.HGESTWNQENR.F
1458.7003 1457.6930 1457.7151 -15.11 101 - 113 0 2 R.HVFGESDELIGQK.V
1488.6267 1487.6194 1487.6391 -13.24 22 - 33 0 1 R.FCGWFDAELSEK.G
1539.7455 1538.7382 1538.7841 -29.84 86 - 99 0 2 K.DLGATWVVLGHSER.R
1602.8644 1601.8571 1601.8817 -15.36 161 - 175 0 2 K.VVLAYEPVWAIGTGK.T
1641.8361 1640.8288 1640.8562 -16.69 163 - 176 0 1 R.ALPFWNDEIAPQIK.A
1661.7781 1660.7708 1660.7953 -14.73 50 - 62 1 1 K.MEFDICYTSVLKR.A
1677.7762 1676.7689 1676.7902 -12.69 50 - 62 1 1 K.MEFDICYTSVLKR.A + Oxidation (M)
1695.8630 1694.8557 1694.8853 -17.42 86 - 100 1 2 K.DLGATWVVLGHSERR.H
1735.8790 1734.8717 1734.9013 -17.03 176 - 190 1 2 K.TATPQQAQEVHEKLR.G
1760.8582 1759.8509 1759.8774 -15.06 147 - 162 1 1 K.AGELPTCESLKDTIAR.A
1956.0295 1955.0222 1954.9432 40.4 6 - 21 1 1 R.LVMVRHGESTWNQENR.F
2060.9666 2059.9593 2059.9713 -5.83 118 - 134 1 1 R.SFDIPPPPMDEKHPYYK.S
2073.9148 2072.9075 2072.9149 -3.57 22 - 39 1 1 R.FCGWFDAELSEKGAEEAK.K
2076.9561 2075.9488 2075.9662 -8.39 118 - 134 1 1 R.SFDIPPPPMDEKHPYYK.S + Oxidation (M)
2144.1042 2143.0969 2143.1136 -7.79 66 - 83 0 1 R.TLWTILDGTDQMWLPVVR.T
2160.0996 2159.0923 2159.1085 -7.51 66 - 83 0 1 R.TLWTILDGTDQMWLPVVR.T + Oxidation (M)
2206.0796 2205.0723 2205.0776 -2.40 34 - 53 0 2 K.LPADTEVVCAPPTAYIDFAR.Q
2321.2261 2320.2188 2320.2209 -0.89 114 - 135 1 2 K.VAHALAEGLGVIACIGEKLDER.E
2827.2913 2826.2840 2826.2092 26.5 11 - 33 1 1 R.HGESTWNQENRFCGWFDAELSEK.G
3029.6843 3028.6770 3028.5757 33.5 220 - 248 0 2 K.ELASQPDVDGFLVGGASLKPEFVDIINAK.-
No match to: 913.6208, 1015.5994, 1135.6008, 1538.7101, 1554.7062, 1556.7716, 1565.7947, 1567.7885, 1615.8529, 1633.7645, 1643.8328, 1665.7703, 1666.7883,
1704.8333, 1734.8981, 1763.7804, 1771.9028, 1873.9642, 1880.8859, 1927.8328, 1991.9366, 2011.9348, 2040.8979, 2046.9888, 2096.0842, 2112.0864, 2175.0806,
2176.0938, 2187.1350, 2192.0671, 2217.1011, 2230.0159, 2249.0798, 2287.0930, 2320.1489, 2349.2085, 2393.2429, 2502.3918, 3153.6504, 3651.1663

2. Mixture 2 Total score: 139 Expect: 1.3e-008 Matches: 25
Components: 1. gi|84000195 phosphoglycerate mutase 2 [Bos taurus]
2. gi|61888856 triosephosphate isomerase [Bos taurus]
Observed Mr(expt) Mr(calc) ppm Start End Miss Comp Peptide
954.5139 953.5066 953.4760 32.2 7 - 14 0 2 K.FFVGGNWK.M
1065.5342 1064.5269 1064.5138 12.3 232 - 240 0 1 R.FLGDEETVR.K
1082.5940 1081.5867 1081.5709 14.6 6 - 14 1 2 R.KFFVGGNWK.M
1150.6758 1149.6685 1149.6618 5.83 181 - 191 0 1 R.VLIAAHGNSLR.G
1357.5730 1356.5657 1356.5807 -11.02 11 - 21 0 1 R.HGESTWNQENR.F
1458.7003 1457.6930 1457.7151 -15.11 101 - 113 0 2 R.HVFGESDELIGQK.V
1488.6267 1487.6194 1487.6391 -13.24 22 - 33 0 1 R.FCGWFDAELSEK.G
1539.7455 1538.7382 1538.7841 -29.84 86 - 99 0 2 K.DLGATWVVLGHSER.R
1602.8644 1601.8571 1601.8817 -15.36 161 - 175 0 2 K.VVLAYEPVWAIGTGK.T
1641.8361 1640.8288 1640.8562 -16.69 163 - 176 0 1 R.ALPFWNDEIAPQIK.A
1661.7781 1660.7708 1660.7953 -14.73 50 - 62 1 1 K.MEFDICYTSVLKR.A
1677.7762 1676.7689 1676.7902 -12.69 50 - 62 1 1 K.MEFDICYTSVLKR.A + Oxidation (M)
1695.8630 1694.8557 1694.8853 -17.42 86 - 100 1 2 K.DLGATWVVLGHSERR.H
1735.8790 1734.8717 1734.9013 -17.03 176 - 190 1 2 K.TATPQQAQEVHEKLR.G
1760.8582 1759.8509 1759.8774 -15.06 147 - 162 1 1 K.AGELPTCESLKDTIAR.A
1956.0295 1955.0222 1954.9432 40.4 6 - 21 1 1 R.LVMVRHGESTWNQENR.F
2060.9666 2059.9593 2059.9713 -5.83 118 - 134 1 1 R.SFDIPPPPMDEKHPYYK.S
2073.9148 2072.9075 2072.9149 -3.57 22 - 39 1 1 R.FCGWFDAELSEKGAEEAK.K
2076.9561 2075.9488 2075.9662 -8.39 118 - 134 1 1 R.SFDIPPPPMDEKHPYYK.S + Oxidation (M)
2144.1042 2143.0969 2143.1136 -7.79 66 - 83 0 1 R.TLWTILDGTDQMWLPVVR.T
2160.0996 2159.0923 2159.1085 -7.51 66 - 83 0 1 R.TLWTILDGTDQMWLPVVR.T + Oxidation (M)
2192.0671 2191.0598 2191.0620 -0.98 34 - 53 0 2 K.VPADTEVVCAPPTAYIDFAR.Q
2321.2261 2320.2188 2320.2209 -0.89 114 - 135 1 2 K.VAHALAEGLGVIACIGEKLDER.E
2827.2913 2826.2840 2826.2092 26.5 11 - 33 1 1 R.HGESTWNQENRFCGWFDAELSEK.G
3029.6843 3028.6770 3028.5757 33.5 220 - 248 0 2 K.ELASQPDVDGFLVGGASLKPEFVDIINAK.Q
No match to: 913.6208, 1015.5994, 1135.6008, 1538.7101, 1554.7062, 1556.7716, 1565.7947, 1567.7885, 1615.8529, 1633.7645, 1643.8328, 1665.7703, 1666.7883,
1704.8333, 1734.8981, 1763.7804, 1771.9028, 1873.9642, 1880.8859, 1927.8328, 1991.9366, 2011.9348, 2040.8979, 2046.9888, 2096.0842, 2112.0864, 2175.0806,
2176.0938, 2187.1350, 2206.0796, 2217.1011, 2230.0159, 2249.0798, 2287.0930, 2320.1489, 2349.2085, 2393.2429, 2502.3918, 3153.6504, 3651.1663

3. Mixture 3 Total score: 126 Expect: 2.7e-007 Matches: 24
Components: 1. gi|84000195 phosphoglycerate mutase 2 [Bos taurus]
2. gi|38512111 Tpi1 protein, partial [Rattus norvegicus]
Observed Mr(expt) Mr(calc) ppm Start End Miss Comp Peptide
954.5139 953.5066 953.4760 32.2 6 - 13 0 2 K.FFVGGNWK.M
1065.5342 1064.5269 1064.5138 12.3 232 - 240 0 1 R.FLGDEETVR.K
1082.5940 1081.5867 1081.5709 14.6 5 - 13 1 2 R.KFFVGGNWK.M
1150.6758 1149.6685 1149.6618 5.83 181 - 191 0 1 R.VLIAAHGNSLR.G
1357.5730 1356.5657 1356.5807 -11.02 11 - 21 0 1 R.HGESTWNQENR.F
1488.6267 1487.6194 1487.6391 -13.24 22 - 33 0 1 R.FCGWFDAELSEK.G
1539.7455 1538.7382 1538.7841 -29.84 85 - 98 0 2 K.DLGATWVVLGHSER.R
1602.8644 1601.8571 1601.8817 -15.36 160 - 174 0 2 K.VVLAYEPVWAIGTGK.T

1641.8361 1640.8288 1640.8562 -16.69 163 - 176 0 1 R.ALPFWNDEIAPQIK.A
1661.7781 1660.7708 1660.7953 -14.73 50 - 62 1 1 K.MEFDICYTSVLKR.A
1677.7762 1676.7689 1676.7902 -12.69 50 - 62 1 1 K.MEFDICYTSVLKR.A + Oxidation (M)
1695.8630 1694.8557 1694.8853 -17.42 85 - 99 1 2 K.DLGATWVVLGHSERR.H
1735.8790 1734.8717 1734.9013 -17.03 175 - 189 1 2 K.TATPQQAQEVHEKLR.G
1760.8582 1759.8509 1759.8774 -15.06 147 - 162 1 1 K.AGELPTCESLKDTIAR.A
1956.0295 1955.0222 1954.9432 40.4 6 - 21 1 1 R.LVMVRHGESTWNQENR.F
2060.9666 2059.9593 2059.9713 -5.83 118 - 134 1 1 R.SFDIPPPPMDEKHPYYK.S
2073.9148 2072.9075 2072.9149 -3.57 22 - 39 1 1 R.FCGWFDAELSEKGAEEAK.K
2076.9561 2075.9488 2075.9662 -8.39 118 - 134 1 1 R.SFDIPPPPMDEKHPYYK.S + Oxidation (M)
2144.1042 2143.0969 2143.1136 -7.79 66 - 83 0 1 R.TLWTILDGTDQMWLPVVR.T
2160.0996 2159.0923 2159.1085 -7.51 66 - 83 0 1 R.TLWTILDGTDQMWLPVVR.T + Oxidation (M)
2192.0671 2191.0598 2191.1136 -24.54 156 - 174 1 2 K.DWCKVVLAYEPVWAIGTGK.T
2206.0796 2205.0723 2205.0776 -2.40 33 - 52 0 2 K.LPADTEVVCAPPTAYIDFAR.Q
2827.2913 2826.2840 2826.2092 26.5 11 - 33 1 1 R.HGESTWNQENRFCGWFDAELSEK.G
3029.6843 3028.6770 3028.5757 33.5 219 - 247 0 2 K.ELASQPDVDGFLVGGASLKPEFVDIINAK.Q
No match to: 913.6208, 1015.5994, 1135.6008, 1458.7003, 1538.7101, 1554.7062, 1556.7716, 1565.7947, 1567.7885, 1615.8529, 1633.7645, 1643.8328, 1665.7703,
1666.7883, 1704.8333, 1734.8981, 1763.7804, 1771.9028, 1873.9642, 1880.8859, 1927.8328, 1991.9366, 2011.9348, 2040.8979, 2046.9888, 2096.0842, 2112.0864,
2175.0806, 2176.0938, 2187.1350, 2217.1011, 2230.0159, 2249.0798, 2287.0930, 2320.1489, 2321.2261, 2349.2085, 2393.2429, 2502.3918, 3153.6504, 3651.1663
4. Mixture 4 Total score: 121 Expect: 8.4e-007 Matches: 23
Components: 1. gi|84000195 phosphoglycerate mutase 2 [Bos taurus]
2. gi|296439800 RecName: Full=Triosephosphate isomerase; Short=TIM; AltName: Full=Triose-phosphate isomerase
Observed Mr(expt) Mr(calc) ppm Start End Miss Comp Peptide
954.5139 953.5066 953.4760 32.2 2 - 9 0 2 K.FFVGGNWK.M
1065.5342 1064.5269 1064.5138 12.3 232 - 240 0 1 R.FLGDEETVR.K
1082.5940 1081.5867 1081.5709 14.6 1 - 9 1 2 -.KFFVGGNWK.M
1150.6758 1149.6685 1149.6618 5.83 181 - 191 0 1 R.VLIAAHGNSLR.G
1357.5730 1356.5657 1356.5807 -11.02 11 - 21 0 1 R.HGESTWNQENR.F
1458.7003 1457.6930 1457.7151 -15.11 74 - 86 0 2 R.HVFGESDELIGQK.L
1488.6267 1487.6194 1487.6391 -13.24 22 - 33 0 1 R.FCGWFDAELSEK.G
1602.8644 1601.8571 1601.8817 -15.36 116 - 130 0 2 K.VVLAYEPVWAIGTGK.T
1641.8361 1640.8288 1640.8562 -16.69 163 - 176 0 1 R.ALPFWNDEIAPQIK.A
1661.7781 1660.7708 1660.7953 -14.73 50 - 62 1 1 K.MEFDICYTSVLKR.A
1677.7762 1676.7689 1676.7902 -12.69 50 - 62 1 1 K.MEFDICYTSVLKR.A + Oxidation (M)
1735.8790 1734.8717 1734.9013 -17.03 131 - 145 1 2 K.TATPQQAQEVHEKLR.I
1760.8582 1759.8509 1759.8774 -15.06 147 - 162 1 1 K.AGELPTCESLKDTIAR.A
1956.0295 1955.0222 1954.9432 40.4 6 - 21 1 1 R.LVMVRHGESTWNQENR.F
2060.9666 2059.9593 2059.9713 -5.83 118 - 134 1 1 R.SFDIPPPPMDEKHPYYK.S
2073.9148 2072.9075 2072.9149 -3.57 22 - 39 1 1 R.FCGWFDAELSEKGAEEAK.K
2076.9561 2075.9488 2075.9662 -8.39 118 - 134 1 1 R.SFDIPPPPMDEKHPYYK.S + Oxidation (M)
2144.1042 2143.0969 2143.1136 -7.79 66 - 83 0 1 R.TLWTILDGTDQMWLPVVR.T
2160.0996 2159.0923 2159.1085 -7.51 66 - 83 0 1 R.TLWTILDGTDQMWLPVVR.T + Oxidation (M)
2192.0671 2191.0598 2191.1136 -24.54 112 - 130 1 2 K.DWCKVVLAYEPVWAIGTGK.T
2206.0796 2205.0723 2205.0776 -2.40 27 - 46 0 2 K.LPADTEVVCAPPTAYIDFAR.I
2827.2913 2826.2840 2826.2092 26.5 11 - 33 1 1 R.HGESTWNQENRFCGWFDAELSEK.G
3029.6843 3028.6770 3028.5757 33.5 159 - 187 0 2 K.ELASQPDVDGFLVGGASLKPEFVDIINAK.Q
No match to: 913.6208, 1015.5994, 1135.6008, 1538.7101, 1539.7455, 1554.7062, 1556.7716, 1565.7947, 1567.7885, 1615.8529, 1633.7645, 1643.8328, 1665.7703,
1666.7883, 1695.8630, 1704.8333, 1734.8981, 1763.7804, 1771.9028, 1873.9642, 1880.8859, 1927.8328, 1991.9366, 2011.9348, 2040.8979, 2046.9888, 2096.0842,
2112.0864, 2175.0806, 2176.0938, 2187.1350, 2217.1011, 2230.0159, 2249.0798, 2287.0930, 2320.1489, 2321.2261, 2349.2085, 2393.2429, 2502.3918, 3153.6504, 3651.1663
5. gi|84000195 Mass: 28838 Score: 100 Expect: 0.00011 Matches: 15
phosphoglycerate mutase 2 [Bos taurus]
Observed Mr(expt) Mr(calc) ppm Start End Miss Peptide
1065.5342 1064.5269 1064.5138 12.3 232 - 240 0 R.FLGDEETVR.K
1150.6758 1149.6685 1149.6618 5.83 181 - 191 0 R.VLIAAHGNSLR.G
1357.5730 1356.5657 1356.5807 -11.02 11 - 21 0 R.HGESTWNQENR.F
1488.6267 1487.6194 1487.6391 -13.24 22 - 33 0 R.FCGWFDAELSEK.G
1641.8361 1640.8288 1640.8562 -16.69 163 - 176 0 R.ALPFWNDEIAPQIK.A
1661.7781 1660.7708 1660.7953 -14.73 50 - 62 1 K.MEFDICYTSVLKR.A
1677.7762 1676.7689 1676.7902 -12.69 50 - 62 1 K.MEFDICYTSVLKR.A + Oxidation (M)
1760.8582 1759.8509 1759.8774 -15.06 147 - 162 1 K.AGELPTCESLKDTIAR.A
1956.0295 1955.0222 1954.9432 40.4 6 - 21 1 R.LVMVRHGESTWNQENR.F
2060.9666 2059.9593 2059.9713 -5.83 118 - 134 1 R.SFDIPPPPMDEKHPYYK.S
2073.9148 2072.9075 2072.9149 -3.57 22 - 39 1 R.FCGWFDAELSEKGAEEAK.K
2076.9561 2075.9488 2075.9662 -8.39 118 - 134 1 R.SFDIPPPPMDEKHPYYK.S + Oxidation (M)
2144.1042 2143.0969 2143.1136 -7.79 66 - 83 0 R.TLWTILDGTDQMWLPVVR.T
2160.0996 2159.0923 2159.1085 -7.51 66 - 83 0 R.TLWTILDGTDQMWLPVVR.T + Oxidation (M)
2827.2913 2826.2840 2826.2092 26.5 11 - 33 1 R.HGESTWNQENRFCGWFDAELSEK.G
No match to: 913.6208, 954.5139, 1015.5994, 1082.5940, 1135.6008, 1458.7003, 1538.7101, 1539.7455, 1554.7062, 1556.7716, 1565.7947, 1567.7885, 1602.8644,
1615.8529, 1633.7645, 1643.8328, 1665.7703, 1666.7883, 1695.8630, 1704.8333, 1734.8981, 1735.8790, 1763.7804, 1771.9028, 1873.9642, 1880.8859, 1927.8328,
1991.9366, 2011.9348, 2040.8979, 2046.9888, 2096.0842, 2112.0864, 2175.0806, 2176.0938, 2187.1350, 2192.0671, 2206.0796, 2217.1011, 2230.0159, 2249.0798,
2287.0930, 2320.1489, 2321.2261, 2349.2085, 2393.2429, 2502.3918, 3029.6843, 3153.6504, 3651.1663
6. gi|296475149 Mass: 28852 Score: 100 Expect: 0.00011 Matches: 15
phosphoglycerate mutase 2 [Bos taurus]
Observed Mr(expt) Mr(calc) ppm Start End Miss Peptide
1065.5342 1064.5269 1064.5138 12.3 232 - 240 0 R.FLGDEETVR.K
1150.6758 1149.6685 1149.6618 5.83 181 - 191 0 R.VLIAAHGNSLR.G
1357.5730 1356.5657 1356.5807 -11.02 11 - 21 0 R.HGESTWNQENR.F
1488.6267 1487.6194 1487.6391 -13.24 22 - 33 0 R.FCGWFDAELSEK.G
1641.8361 1640.8288 1640.8562 -16.69 163 - 176 0 R.ALPFWNDEIAPQIK.A
1661.7781 1660.7708 1660.7953 -14.73 50 - 62 1 K.MEFDICYTSVLKR.A
1677.7762 1676.7689 1676.7902 -12.69 50 - 62 1 K.MEFDICYTSVLKR.A + Oxidation (M)
1760.8582 1759.8509 1759.8774 -15.06 147 - 162 1 K.AGELPTCESLKDTIAR.A
1956.0295 1955.0222 1954.9432 40.4 6 - 21 1 R.LVMVRHGESTWNQENR.F

2060.9666 2059.9593 2059.9713 -5.83 118 - 134 1 R.SFDIPPPPMDEKHPYYK.S
2073.9148 2072.9075 2072.9149 -3.57 22 - 39 1 R.FCGWFDAELSEKGAEEAK.R
2076.9561 2075.9488 2075.9662 -8.39 118 - 134 1 R.SFDIPPPPMDEKHPYYK.S + Oxidation (M)
2144.1042 2143.0969 2143.1136 -7.79 66 - 83 0 R.TLWTILDGTDQMWLPVVR.T
2160.0996 2159.0923 2159.1085 -7.51 66 - 83 0 R.TLWTILDGTDQMWLPVVR.T + Oxidation (M)
2827.2913 2826.2840 2826.2092 26.5 11 - 33 1 R.HGESTWNQENRFCGWFDAELSEK.G
No match to: 913.6208, 954.5139, 1015.5994, 1082.5940, 1135.6008, 1458.7003, 1538.7101, 1539.7455, 1554.7062, 1556.7716, 1565.7947, 1567.7885, 1602.8644,
1615.8529, 1633.7645, 1643.8328, 1665.7703, 1666.7883, 1695.8630, 1704.8333, 1734.8981, 1735.8790, 1763.7804, 1771.9028, 1873.9642, 1880.8859, 1927.8328,
1991.9366, 2011.9348, 2040.8979, 2046.9888, 2096.0842, 2112.0864, 2175.0806, 2176.0938, 2187.1350, 2192.0671, 2206.0796, 2217.1011, 2230.0159, 2249.0798,
2287.0930, 2320.1489, 2321.2261, 2349.2085, 2393.2429, 2502.3918, 3029.6843, 3153.6504, 3651.1663
7. gi|262263205 Mass: 26879 Score: 93 Expect: 0.00052 Matches: 10
triosephosphate isomerase 1 [Sus scrofa]
Observed Mr(expt) Mr(calc) ppm Start End Miss Peptide
954.5139 953.5066 953.4760 32.2 7 - 14 0 K.FFVGGNWK.M
1082.5940 1081.5867 1081.5709 14.6 6 - 14 1 R.KFFVGGNWK.M
1458.7003 1457.6930 1457.7151 -15.11 101 - 113 0 R.HVFGESDELIGQK.V
1539.7455 1538.7382 1538.7841 -29.84 86 - 99 0 K.DLGATWVVLGHSER.R
1602.8644 1601.8571 1601.8817 -15.36 161 - 175 0 K.VVLAYEPVWAIGTGK.T
1695.8630 1694.8557 1694.8853 -17.42 86 - 100 1 K.DLGATWVVLGHSERR.H
1735.8790 1734.8717 1734.9013 -17.03 176 - 190 1 K.TATPQQAQEVHEKLR.G
2206.0796 2205.0723 2205.0776 -2.40 34 - 53 0 K.LPADTEVVCAPPTAYIDFAR.Q
2321.2261 2320.2188 2320.2209 -0.89 114 - 135 1 K.VAHALAEGLGVIACIGEKLDER.E
3029.6843 3028.6770 3028.5757 33.5 220 - 248 0 K.ELASQPDVDGFLVGGASLKPEFVDIINAK.-
No match to: 913.6208, 1015.5994, 1065.5342, 1135.6008, 1150.6758, 1357.5730, 1488.6267, 1538.7101, 1554.7062, 1556.7716, 1565.7947, 1567.7885, 1615.8529,
1633.7645, 1641.8361, 1643.8328, 1661.7781, 1665.7703, 1666.7883, 1677.7762, 1704.8333, 1734.8981, 1760.8582, 1763.7804, 1771.9028, 1873.9642, 1880.8859,
1927.8328, 1956.0295, 1991.9366, 2011.9348, 2040.8979, 2046.9888, 2060.9666, 2073.9148, 2076.9561, 2096.0842, 2112.0864, 2144.1042, 2160.0996, 2175.0806,
2176.0938, 2187.1350, 2192.0671, 2217.1011, 2230.0159, 2249.0798, 2287.0930, 2320.1489, 2349.2085, 2393.2429, 2502.3918, 2827.2913, 3153.6504, 3651.1663
8. gi|61888856 Mass: 26901 Score: 93 Expect: 0.00053 Matches: 10
triosephosphate isomerase [Bos taurus]
Observed Mr(expt) Mr(calc) ppm Start End Miss Peptide
954.5139 953.5066 953.4760 32.2 7 - 14 0 K.FFVGGNWK.M
1082.5940 1081.5867 1081.5709 14.6 6 - 14 1 R.KFFVGGNWK.M
1458.7003 1457.6930 1457.7151 -15.11 101 - 113 0 R.HVFGESDELIGQK.V
1539.7455 1538.7382 1538.7841 -29.84 86 - 99 0 K.DLGATWVVLGHSER.R
1602.8644 1601.8571 1601.8817 -15.36 161 - 175 0 K.VVLAYEPVWAIGTGK.T
1695.8630 1694.8557 1694.8853 -17.42 86 - 100 1 K.DLGATWVVLGHSERR.H
1735.8790 1734.8717 1734.9013 -17.03 176 - 190 1 K.TATPQQAQEVHEKLR.G
2192.0671 2191.0598 2191.0620 -0.98 34 - 53 0 K.VPADTEVVCAPPTAYIDFAR.Q
2321.2261 2320.2188 2320.2209 -0.89 114 - 135 1 K.VAHALAEGLGVIACIGEKLDER.E
3029.6843 3028.6770 3028.5757 33.5 220 - 248 0 K.ELASQPDVDGFLVGGASLKPEFVDIINAK.Q
No match to: 913.6208, 1015.5994, 1065.5342, 1135.6008, 1150.6758, 1357.5730, 1488.6267, 1538.7101, 1554.7062, 1556.7716, 1565.7947, 1567.7885, 1615.8529,
1633.7645, 1641.8361, 1643.8328, 1661.7781, 1665.7703, 1666.7883, 1677.7762, 1704.8333, 1734.8981, 1760.8582, 1763.7804, 1771.9028, 1873.9642, 1880.8859,
1927.8328, 1956.0295, 1991.9366, 2011.9348, 2040.8979, 2046.9888, 2060.9666, 2073.9148, 2076.9561, 2096.0842, 2112.0864, 2144.1042, 2160.0996, 2175.0806,
2176.0938, 2187.1350, 2206.0796, 2217.1011, 2230.0159, 2249.0798, 2287.0930, 2320.1489, 2349.2085, 2393.2429, 2502.3918, 2827.2913, 3153.6504, 3651.1663
9. gi|348554958 Mass: 30916 Score: 87 Expect: 0.002 Matches: 10
PREDICTED: triosephosphate isomerase-like [Cavia porcellus]
Observed Mr(expt) Mr(calc) ppm Start End Miss Peptide
954.5139 953.5066 953.4760 32.2 44 - 51 0 K.FFVGGNWK.M
1082.5940 1081.5867 1081.5709 14.6 43 - 51 1 R.KFFVGGNWK.M
1458.7003 1457.6930 1457.7151 -15.11 138 - 150 0 R.HVFGESDELIGQK.V
1539.7455 1538.7382 1538.7841 -29.84 123 - 136 0 K.DLGATWVVLGHSER.R
1602.8644 1601.8571 1601.8817 -15.36 198 - 212 0 K.VVLAYEPVWAIGTGK.T
1695.8630 1694.8557 1694.8853 -17.42 123 - 137 1 K.DLGATWVVLGHSERR.H
1735.8790 1734.8717 1734.9013 -17.03 213 - 227 1 K.TATPQQAQEVHEKLR.G
2192.0671 2191.0598 2191.0620 -0.98 71 - 90 0 K.VPADTEVVCAPPTAYIDFAR.Q
2321.2261 2320.2188 2320.2209 -0.89 151 - 172 1 K.VAHALAEGLGVIACIGEKLDER.E
3029.6843 3028.6770 3028.5757 33.5 257 - 285 0 K.ELASQPDVDGFLVGGASLKPEFVDIINAK.H
No match to: 913.6208, 1015.5994, 1065.5342, 1135.6008, 1150.6758, 1357.5730, 1488.6267, 1538.7101, 1554.7062, 1556.7716, 1565.7947, 1567.7885, 1615.8529,
1633.7645, 1641.8361, 1643.8328, 1661.7781, 1665.7703, 1666.7883, 1677.7762, 1704.8333, 1734.8981, 1760.8582, 1763.7804, 1771.9028, 1873.9642, 1880.8859,
1927.8328, 1956.0295, 1991.9366, 2011.9348, 2040.8979, 2046.9888, 2060.9666, 2073.9148, 2076.9561, 2096.0842, 2112.0864, 2144.1042, 2160.0996, 2175.0806,
2176.0938, 2187.1350, 2206.0796, 2217.1011, 2230.0159, 2249.0798, 2287.0930, 2320.1489, 2349.2085, 2393.2429, 2502.3918, 2827.2913, 3153.6504, 3651.1663
10. gi|201066358 Mass: 28830 Score: 85 Expect: 0.0033 Matches: 13
phosphoglycerate mutase 2 [Sus scrofa]
Observed Mr(expt) Mr(calc) ppm Start End Miss Peptide
1065.5342 1064.5269 1064.5138 12.3 232 - 240 0 R.FLGDEETVR.K
1082.5940 1081.5867 1081.6179 -28.79 2 - 10 1 M.ATHRLVMVR.H
1150.6758 1149.6685 1149.6618 5.83 181 - 191 0 R.VLIAAHGNSLR.G
1357.5730 1356.5657 1356.5807 -11.02 11 - 21 0 R.HGESTWNQENR.F
1488.6267 1487.6194 1487.6391 -13.24 22 - 33 0 R.FCGWFDAELSEK.G
1641.8361 1640.8288 1640.8562 -16.69 163 - 176 0 R.ALPFWNDEIAPQIK.A
1661.7781 1660.7708 1660.7953 -14.73 50 - 62 1 K.MEFDICYTSVLKR.A
1677.7762 1676.7689 1676.7902 -12.69 50 - 62 1 K.MEFDICYTSVLKR.A + Oxidation (M)
1956.0295 1955.0222 1954.9432 40.4 6 - 21 1 R.LVMVRHGESTWNQENR.F
2073.9148 2072.9075 2072.9149 -3.57 22 - 39 1 R.FCGWFDAELSEKGAEEAK.R
2144.1042 2143.0969 2143.1136 -7.79 66 - 83 0 R.TLWTILDGTDQMWLPVVR.T
2160.0996 2159.0923 2159.1085 -7.51 66 - 83 0 R.TLWTILDGTDQMWLPVVR.T + Oxidation (M)
2827.2913 2826.2840 2826.2092 26.5 11 - 33 1 R.HGESTWNQENRFCGWFDAELSEK.G

No match to: 913.6208, 954.5139, 1015.5994, 1135.6008, 1458.7003, 1538.7101, 1539.7455, 1554.7062, 1556.7716, 1565.7947, 1567.7885, 1602.8644, 1615.8529,
1633.7645, 1643.8328, 1665.7703, 1666.7883, 1695.8630, 1704.8333, 1734.8981, 1735.8790, 1760.8582, 1763.7804, 1771.9028, 1873.9642, 1880.8859, 1927.8328,
1991.9366, 2011.9348, 2040.8979, 2046.9888, 2060.9666, 2076.9561, 2096.0842, 2112.0864, 2175.0806, 2176.0938, 2187.1350, 2192.0671, 2206.0796, 2217.1011,
2230.0159, 2249.0798, 2287.0930, 2320.1489, 2321.2261, 2349.2085, 2393.2429, 2502.3918, 3029.6843, 3153.6504, 3651.1663
11. gi|90200404 Mass: 26863 Score: 81 Expect: 0.0084 Matches: 9
triosephosphate isomerase 1 [Sus scrofa]
Observed Mr(expt) Mr(calc) ppm Start End Miss Peptide
954.5139 953.5066 953.4760 32.2 7 - 14 0 K.FFVGGNWK.M
1082.5940 1081.5867 1081.5709 14.6 6 - 14 1 R.KFFVGGNWK.M
1539.7455 1538.7382 1538.7841 -29.84 86 - 99 0 K.DLGATWVVLGHSER.R
1602.8644 1601.8571 1601.8817 -15.36 161 - 175 0 K.VVLAYEPVWAIGTGK.T
1695.8630 1694.8557 1694.8853 -17.42 86 - 100 1 K.DLGATWVVLGHSERR.H
1735.8790 1734.8717 1734.9013 -17.03 176 - 190 1 K.TATPQQAQEVHEKLR.G
2206.0796 2205.0723 2205.0776 -2.40 34 - 53 0 K.LPADTEVVCAPPTAYIDFAR.Q
2321.2261 2320.2188 2320.2209 -0.89 114 - 135 1 K.VAHALAEGLGVIACIGEKLDER.E
3029.6843 3028.6770 3028.5757 33.5 220 - 248 0 K.ELASQPDVDGFLVGGASLKPEFVDIINAK.-
No match to: 913.6208, 1015.5994, 1065.5342, 1135.6008, 1150.6758, 1357.5730, 1458.7003, 1488.6267, 1538.7101, 1554.7062, 1556.7716, 1565.7947, 1567.7885,
1615.8529, 1633.7645, 1641.8361, 1643.8328, 1661.7781, 1665.7703, 1666.7883, 1677.7762, 1704.8333, 1734.8981, 1760.8582, 1763.7804, 1771.9028, 1873.9642,
1880.8859, 1927.8328, 1956.0295, 1991.9366, 2011.9348, 2040.8979, 2046.9888, 2060.9666, 2073.9148, 2076.9561, 2096.0842, 2112.0864, 2144.1042, 2160.0996,
2175.0806, 2176.0938, 2187.1350, 2192.0671, 2217.1011, 2230.0159, 2249.0798, 2287.0930, 2320.1489, 2349.2085, 2393.2429, 2502.3918, 2827.2913, 3153.6504, 3651.1663
12. gi|38512111 Mass: 27214 Score: 80 Expect: 0.0099 Matches: 9
Tpi1 protein, partial [Rattus norvegicus]
Observed Mr(expt) Mr(calc) ppm Start End Miss Peptide
954.5139 953.5066 953.4760 32.2 6 - 13 0 K.FFVGGNWK.M
1082.5940 1081.5867 1081.5709 14.6 5 - 13 1 R.KFFVGGNWK.M
1539.7455 1538.7382 1538.7841 -29.84 85 - 98 0 K.DLGATWVVLGHSER.R
1602.8644 1601.8571 1601.8817 -15.36 160 - 174 0 K.VVLAYEPVWAIGTGK.T
1695.8630 1694.8557 1694.8853 -17.42 85 - 99 1 K.DLGATWVVLGHSERR.H
1735.8790 1734.8717 1734.9013 -17.03 175 - 189 1 K.TATPQQAQEVHEKLR.G
2192.0671 2191.0598 2191.1136 -24.54 156 - 174 1 K.DWCKVVLAYEPVWAIGTGK.T
2206.0796 2205.0723 2205.0776 -2.40 33 - 52 0 K.LPADTEVVCAPPTAYIDFAR.Q
3029.6843 3028.6770 3028.5757 33.5 219 - 247 0 K.ELASQPDVDGFLVGGASLKPEFVDIINAK.Q
No match to: 913.6208, 1015.5994, 1065.5342, 1135.6008, 1150.6758, 1357.5730, 1458.7003, 1488.6267, 1538.7101, 1554.7062, 1556.7716, 1565.7947, 1567.7885,
1615.8529, 1633.7645, 1641.8361, 1643.8328, 1661.7781, 1665.7703, 1666.7883, 1677.7762, 1704.8333, 1734.8981, 1760.8582, 1763.7804, 1771.9028, 1873.9642,
1880.8859, 1927.8328, 1956.0295, 1991.9366, 2011.9348, 2040.8979, 2046.9888, 2060.9666, 2073.9148, 2076.9561, 2096.0842, 2112.0864, 2144.1042, 2160.0996,
2175.0806, 2176.0938, 2187.1350, 2217.1011, 2230.0159, 2249.0798, 2287.0930, 2320.1489, 2321.2261, 2349.2085, 2393.2429, 2502.3918, 2827.2913, 3153.6504, 3651.1663
13. gi|538426 Mass: 27417 Score: 80 Expect: 0.01 Matches: 9
triosephosphate isomerase [Rattus norvegicus]
Observed Mr(expt) Mr(calc) ppm Start End Miss Peptide
954.5139 953.5066 953.4760 32.2 7 - 14 0 K.FFVGGNWK.M
1082.5940 1081.5867 1081.5709 14.6 6 - 14 1 R.KFFVGGNWK.M
1539.7455 1538.7382 1538.7841 -29.84 86 - 99 0 K.DLGATWVVLGHSER.R
1602.8644 1601.8571 1601.8817 -15.36 161 - 175 0 K.VVLAYEPVWAIGTGK.T
1695.8630 1694.8557 1694.8853 -17.42 86 - 100 1 K.DLGATWVVLGHSERR.H
1735.8790 1734.8717 1734.9013 -17.03 176 - 190 1 K.TATPQQAQEVHEKLR.G
2192.0671 2191.0598 2191.1136 -24.54 157 - 175 1 K.DWCKVVLAYEPVWAIGTGK.T
2206.0796 2205.0723 2205.0776 -2.40 34 - 53 0 K.LPADTEVVCAPPTAYIDFAR.Q
3029.6843 3028.6770 3028.5757 33.5 220 - 248 0 K.ELASQPDVDGFLVGGASLKPEFVDIINAK.Q
No match to: 913.6208, 1015.5994, 1065.5342, 1135.6008, 1150.6758, 1357.5730, 1458.7003, 1488.6267, 1538.7101, 1554.7062, 1556.7716, 1565.7947, 1567.7885,
1615.8529, 1633.7645, 1641.8361, 1643.8328, 1661.7781, 1665.7703, 1666.7883, 1677.7762, 1704.8333, 1734.8981, 1760.8582, 1763.7804, 1771.9028, 1873.9642,
1880.8859, 1927.8328, 1956.0295, 1991.9366, 2011.9348, 2040.8979, 2046.9888, 2060.9666, 2073.9148, 2076.9561, 2096.0842, 2112.0864, 2144.1042, 2160.0996,
2175.0806, 2176.0938, 2187.1350, 2217.1011, 2230.0159, 2249.0798, 2287.0930, 2320.1489, 2321.2261, 2349.2085, 2393.2429, 2502.3918, 2827.2913, 3153.6504, 3651.1663
14. gi|117935064 Mass: 27345 Score: 80 Expect: 0.01 Matches: 9
triosephosphate isomerase [Rattus norvegicus]
Observed Mr(expt) Mr(calc) ppm Start End Miss Peptide
954.5139 953.5066 953.4760 32.2 7 - 14 0 K.FFVGGNWK.M
1082.5940 1081.5867 1081.5709 14.6 6 - 14 1 R.KFFVGGNWK.M
1539.7455 1538.7382 1538.7841 -29.84 86 - 99 0 K.DLGATWVVLGHSER.R
1602.8644 1601.8571 1601.8817 -15.36 161 - 175 0 K.VVLAYEPVWAIGTGK.T
1695.8630 1694.8557 1694.8853 -17.42 86 - 100 1 K.DLGATWVVLGHSERR.H
1735.8790 1734.8717 1734.9013 -17.03 176 - 190 1 K.TATPQQAQEVHEKLR.G
2192.0671 2191.0598 2191.1136 -24.54 157 - 175 1 K.DWCKVVLAYEPVWAIGTGK.T
2206.0796 2205.0723 2205.0776 -2.40 34 - 53 0 K.LPADTEVVCAPPTAYIDFAR.Q
3029.6843 3028.6770 3028.5757 33.5 220 - 248 0 K.ELASQPDVDGFLVGGASLKPEFVDIINAK.Q
No match to: 913.6208, 1015.5994, 1065.5342, 1135.6008, 1150.6758, 1357.5730, 1458.7003, 1488.6267, 1538.7101, 1554.7062, 1556.7716, 1565.7947, 1567.7885,
1615.8529, 1633.7645, 1641.8361, 1643.8328, 1661.7781, 1665.7703, 1666.7883, 1677.7762, 1704.8333, 1734.8981, 1760.8582, 1763.7804, 1771.9028, 1873.9642,
1880.8859, 1927.8328, 1956.0295, 1991.9366, 2011.9348, 2040.8979, 2046.9888, 2060.9666, 2073.9148, 2076.9561, 2096.0842, 2112.0864, 2144.1042, 2160.0996,
2175.0806, 2176.0938, 2187.1350, 2217.1011, 2230.0159, 2249.0798, 2287.0930, 2320.1489, 2321.2261, 2349.2085, 2393.2429, 2502.3918, 2827.2913, 3153.6504, 3651.1663
15. gi|80971510 Mass: 26941 Score: 77 Expect: 0.02 Matches: 9
triosephosphate isomerase [Sus scrofa]
Observed Mr(expt) Mr(calc) ppm Start End Miss Peptide
954.5139 953.5066 953.4760 32.2 7 - 14 0 K.FFVGGNWK.M
1082.5940 1081.5867 1081.5709 14.6 6 - 14 1 R.KFFVGGNWK.M

1458.7003 1457.6930 1457.7151 -15.11 101 - 113 0 R.HVFGESDELIGQK.V
1539.7455 1538.7382 1538.7841 -29.84 86 - 99 0 K.DLGATWVVLGHSER.R
1602.8644 1601.8571 1601.8817 -15.36 161 - 175 0 K.VVLAYEPVWAIGTGK.T
1695.8630 1694.8557 1694.8853 -17.42 86 - 100 1 K.DLGATWVVLGHSERR.H
1735.8790 1734.8717 1734.9013 -17.03 176 - 190 1 K.TATPQQAQEVHEKLR.G
2206.0796 2205.0723 2205.0776 -2.40 34 - 53 0 K.LPADTEVVCAPPTAYIDFAR.Q
2321.2261 2320.2188 2320.2209 -0.89 114 - 135 1 K.VAHALAEGLGVIACIGEKLDER.E
No match to: 913.6208, 1015.5994, 1065.5342, 1135.6008, 1150.6758, 1357.5730, 1488.6267, 1538.7101, 1554.7062, 1556.7716, 1565.7947, 1567.7885, 1615.8529,
1633.7645, 1641.8361, 1643.8328, 1661.7781, 1665.7703, 1666.7883, 1677.7762, 1704.8333, 1734.8981, 1760.8582, 1763.7804, 1771.9028, 1873.9642, 1880.8859,
1927.8328, 1956.0295, 1991.9366, 2011.9348, 2040.8979, 2046.9888, 2060.9666, 2073.9148, 2076.9561, 2096.0842, 2112.0864, 2144.1042, 2160.0996, 2175.0806,
2176.0938, 2187.1350, 2192.0671, 2217.1011, 2230.0159, 2249.0798, 2287.0930, 2320.1489, 2349.2085, 2393.2429, 2502.3918, 2827.2913, 3029.6843, 3153.6504, 3651.1663
16. gi|296439800 Mass: 20636 Score: 77 Expect: 0.02 Matches: 8
RecName: Full=Triosephosphate isomerase; Short=TIM; AltName: Full=Triose-phosphate isomerase
Observed Mr(expt) Mr(calc) ppm Start End Miss Peptide
954.5139 953.5066 953.4760 32.2 2 - 9 0 K.FFVGGNWK.M
1082.5940 1081.5867 1081.5709 14.6 1 - 9 1 -.KFFVGGNWK.M
1458.7003 1457.6930 1457.7151 -15.11 74 - 86 0 R.HVFGESDELIGQK.L
1602.8644 1601.8571 1601.8817 -15.36 116 - 130 0 K.VVLAYEPVWAIGTGK.T
1735.8790 1734.8717 1734.9013 -17.03 131 - 145 1 K.TATPQQAQEVHEKLR.I
2192.0671 2191.0598 2191.1136 -24.54 112 - 130 1 K.DWCKVVLAYEPVWAIGTGK.T
2206.0796 2205.0723 2205.0776 -2.40 27 - 46 0 K.LPADTEVVCAPPTAYIDFAR.I
3029.6843 3028.6770 3028.5757 33.5 159 - 187 0 K.ELASQPDVDGFLVGGASLKPEFVDIINAK.Q
No match to: 913.6208, 1015.5994, 1065.5342, 1135.6008, 1150.6758, 1357.5730, 1488.6267, 1538.7101, 1539.7455, 1554.7062, 1556.7716, 1565.7947, 1567.7885,
1615.8529, 1633.7645, 1641.8361, 1643.8328, 1661.7781, 1665.7703, 1666.7883, 1677.7762, 1695.8630, 1704.8333, 1734.8981, 1760.8582, 1763.7804, 1771.9028,
1873.9642, 1880.8859, 1927.8328, 1956.0295, 1991.9366, 2011.9348, 2040.8979, 2046.9888, 2060.9666, 2073.9148, 2076.9561, 2096.0842, 2112.0864, 2144.1042,
2160.0996, 2175.0806, 2176.0938, 2187.1350, 2217.1011, 2230.0159, 2249.0798, 2287.0930, 2320.1489, 2321.2261, 2349.2085, 2393.2429, 2502.3918, 2827.2913,
3153.6504, 3651.1663
17. gi|149049470 Mass: 31676 Score: 75 Expect: 0.033 Matches: 9
rCG29914, isoform CRA_b [Rattus norvegicus]
Observed Mr(expt) Mr(calc) ppm Start End Miss Peptide
954.5139 953.5066 953.4760 32.2 44 - 51 0 K.FFVGGNWK.M
1082.5940 1081.5867 1081.5709 14.6 43 - 51 1 R.KFFVGGNWK.M
1539.7455 1538.7382 1538.7841 -29.84 123 - 136 0 K.DLGATWVVLGHSER.R
1602.8644 1601.8571 1601.8817 -15.36 198 - 212 0 K.VVLAYEPVWAIGTGK.T
1695.8630 1694.8557 1694.8853 -17.42 123 - 137 1 K.DLGATWVVLGHSERR.H
1735.8790 1734.8717 1734.9013 -17.03 213 - 227 1 K.TATPQQAQEVHEKLR.G
2192.0671 2191.0598 2191.1136 -24.54 194 - 212 1 K.DWCKVVLAYEPVWAIGTGK.T
2206.0796 2205.0723 2205.0776 -2.40 71 - 90 0 K.LPADTEVVCAPPTAYIDFAR.Q
3029.6843 3028.6770 3028.5757 33.5 257 - 285 0 K.ELASQPDVDGFLVGGASLKPEFVDIINAK.Q
No match to: 913.6208, 1015.5994, 1065.5342, 1135.6008, 1150.6758, 1357.5730, 1458.7003, 1488.6267, 1538.7101, 1554.7062, 1556.7716, 1565.7947, 1567.7885,
1615.8529, 1633.7645, 1641.8361, 1643.8328, 1661.7781, 1665.7703, 1666.7883, 1677.7762, 1704.8333, 1734.8981, 1760.8582, 1763.7804, 1771.9028, 1873.9642,
1880.8859, 1927.8328, 1956.0295, 1991.9366, 2011.9348, 2040.8979, 2046.9888, 2060.9666, 2073.9148, 2076.9561, 2096.0842, 2112.0864, 2144.1042, 2160.0996,
2175.0806, 2176.0938, 2187.1350, 2217.1011, 2230.0159, 2249.0798, 2287.0930, 2320.1489, 2321.2261, 2349.2085, 2393.2429, 2502.3918, 2827.2913, 3153.6504, 3651.1663
18. gi|149704608 Mass: 28773 Score: 74 Expect: 0.045 Matches: 12
PREDICTED: phosphoglycerate mutase 2-like [Equus caballus]
Observed Mr(expt) Mr(calc) ppm Start End Miss Peptide
1065.5342 1064.5269 1064.5138 12.3 232 - 240 0 R.FLGDEETVR.K
1082.5940 1081.5867 1081.6179 -28.79 2 - 10 1 M.ATHRLVMVR.H
1150.6758 1149.6685 1149.6618 5.83 181 - 191 0 R.VLIAAHGNSLR.G
1357.5730 1356.5657 1356.5807 -11.02 11 - 21 0 R.HGESTWNQENR.F
1488.6267 1487.6194 1487.6391 -13.24 22 - 33 0 R.FCGWFDAELSEK.G
1661.7781 1660.7708 1660.7953 -14.73 50 - 62 1 K.MEFDICYTSVLKR.A
1677.7762 1676.7689 1676.7902 -12.69 50 - 62 1 K.MEFDICYTSVLKR.A + Oxidation (M)
1956.0295 1955.0222 1954.9432 40.4 6 - 21 1 R.LVMVRHGESTWNQENR.F
2073.9148 2072.9075 2072.9149 -3.57 22 - 39 1 R.FCGWFDAELSEKGAEEAK.R
2144.1042 2143.0969 2143.1136 -7.79 66 - 83 0 R.TLWTILDGTDQMWLPVVR.T
2160.0996 2159.0923 2159.1085 -7.51 66 - 83 0 R.TLWTILDGTDQMWLPVVR.T + Oxidation (M)
2827.2913 2826.2840 2826.2092 26.5 11 - 33 1 R.HGESTWNQENRFCGWFDAELSEK.G
No match to: 913.6208, 954.5139, 1015.5994, 1135.6008, 1458.7003, 1538.7101, 1539.7455, 1554.7062, 1556.7716, 1565.7947, 1567.7885, 1602.8644, 1615.8529,
1633.7645, 1641.8361, 1643.8328, 1665.7703, 1666.7883, 1695.8630, 1704.8333, 1734.8981, 1735.8790, 1760.8582, 1763.7804, 1771.9028, 1873.9642, 1880.8859,
1927.8328, 1991.9366, 2011.9348, 2040.8979, 2046.9888, 2060.9666, 2076.9561, 2096.0842, 2112.0864, 2175.0806, 2176.0938, 2187.1350, 2192.0671, 2206.0796,
2217.1011, 2230.0159, 2249.0798, 2287.0930, 2320.1489, 2321.2261, 2349.2085, 2393.2429, 2502.3918, 3029.6843, 3153.6504, 3651.1663
19. gi|291394897 Mass: 28820 Score: 74 Expect: 0.045 Matches: 12
PREDICTED: phosphoglycerate mutase 2 [Oryctolagus cuniculus]
Observed Mr(expt) Mr(calc) ppm Start End Miss Peptide
1065.5342 1064.5269 1064.5138 12.3 232 - 240 0 R.FLGDEETVR.K
1082.5940 1081.5867 1081.6179 -28.79 2 - 10 1 M.ATHRLVMVR.H
1150.6758 1149.6685 1149.6618 5.83 181 - 191 0 R.VLIAAHGNSLR.G
1357.5730 1356.5657 1356.5807 -11.02 11 - 21 0 R.HGESTWNQENR.F
1488.6267 1487.6194 1487.6391 -13.24 22 - 33 0 R.FCGWFDAELSEK.G
1661.7781 1660.7708 1660.7953 -14.73 50 - 62 1 K.MEFDICYTSVLKR.A
1677.7762 1676.7689 1676.7902 -12.69 50 - 62 1 K.MEFDICYTSVLKR.A + Oxidation (M)
1956.0295 1955.0222 1954.9432 40.4 6 - 21 1 R.LVMVRHGESTWNQENR.F
2073.9148 2072.9075 2072.9149 -3.57 22 - 39 1 R.FCGWFDAELSEKGAEEAK.R
2144.1042 2143.0969 2143.1136 -7.79 66 - 83 0 R.TLWTILDGTDQMWLPVVR.T
2160.0996 2159.0923 2159.1085 -7.51 66 - 83 0 R.TLWTILDGTDQMWLPVVR.T + Oxidation (M)
2827.2913 2826.2840 2826.2092 26.5 11 - 33 1 R.HGESTWNQENRFCGWFDAELSEK.G

No match to: 913.6208, 954.5139, 1015.5994, 1135.6008, 1458.7003, 1538.7101, 1539.7455, 1554.7062, 1556.7716, 1565.7947, 1567.7885, 1602.8644, 1615.8529,
1633.7645, 1641.8361, 1643.8328, 1665.7703, 1666.7883, 1695.8630, 1704.8333, 1734.8981, 1735.8790, 1760.8582, 1763.7804, 1771.9028, 1873.9642, 1880.8859,
1927.8328, 1991.9366, 2011.9348, 2040.8979, 2046.9888, 2060.9666, 2076.9561, 2096.0842, 2112.0864, 2175.0806, 2176.0938, 2187.1350, 2192.0671, 2206.0796,
2217.1011, 2230.0159, 2249.0798, 2287.0930, 2320.1489, 2321.2261, 2349.2085, 2393.2429, 2502.3918, 3029.6843, 3153.6504, 3651.1663
20. gi|999892 Mass: 26807 Score: 69 Expect: 0.14 Matches: 8
Chain A, Crystal Structure Of Recombinant Human Triosephosphate Isomerase At 2.8 Angstroms Resolution. Triosephosphate Isomerase Related Human Genetic Disorders And
Observed Mr(expt) Mr(calc) ppm Start End Miss Peptide
954.5139 953.5066 953.4760 32.2 6 - 13 0 K.FFVGGNWK.M
1082.5940 1081.5867 1081.5709 14.6 5 - 13 1 R.KFFVGGNWK.M
1458.7003 1457.6930 1457.7151 -15.11 100 - 112 0 R.HVFGESDELIGQK.V
1602.8644 1601.8571 1601.8817 -15.36 160 - 174 0 K.VVLAYEPVWAIGTGK.T
1735.8790 1734.8717 1734.9013 -17.03 175 - 189 1 K.TATPQQAQEVHEKLR.G
2192.0671 2191.0598 2191.0620 -0.98 33 - 52 0 K.VPADTEVVCAPPTAYIDFAR.Q
2321.2261 2320.2188 2320.2209 -0.89 113 - 134 1 K.VAHALAEGLGVIACIGEKLDER.E
3029.6843 3028.6770 3028.5757 33.5 219 - 247 0 K.ELASQPDVDGFLVGGASLKPEFVDIINAK.Q
No match to: 913.6208, 1015.5994, 1065.5342, 1135.6008, 1150.6758, 1357.5730, 1488.6267, 1538.7101, 1539.7455, 1554.7062, 1556.7716, 1565.7947, 1567.7885,
1615.8529, 1633.7645, 1641.8361, 1643.8328, 1661.7781, 1665.7703, 1666.7883, 1677.7762, 1695.8630, 1704.8333, 1734.8981, 1760.8582, 1763.7804, 1771.9028,
1873.9642, 1880.8859, 1927.8328, 1956.0295, 1991.9366, 2011.9348, 2040.8979, 2046.9888, 2060.9666, 2073.9148, 2076.9561, 2096.0842, 2112.0864, 2144.1042,
2160.0996, 2175.0806, 2176.0938, 2187.1350, 2206.0796, 2217.1011, 2230.0159, 2249.0798, 2287.0930, 2320.1489, 2349.2085, 2393.2429, 2502.3918, 2827.2913,
3153.6504, 3651.1663

Search Parameters

Type of search : Peptide Mass Fingerprint
Enzyme : Trypsin
Fixed modifications : Carbamidomethyl (C)
Variable modifications : Oxidation (M)
Mass values : Monoisotopic
Protein Mass : Unrestricted
Peptide Mass Tolerance : ± 50 ppm
Peptide Charge State : 1+
Max Missed Cleavages : 1
Number of queries : 65
Mascot: http://www.matrixscience.com/

COVERAGE BAND 6

Mascot Search Results

Protein View

Match to: gi|84000195 Score: 84 Expect: 0.0039
phosphoglycerate mutase 2 [Bos taurus]
Nominal mass (Mr): 28838; Calculated pI value: 8.99
NCBI BLAST search of gi|84000195 against nr
Unformatted sequence string for pasting into other applications
Taxonomy: Bos taurus
Links to retrieve other entries containing this sequence from NCBI Entrez:
gi|122138663 from Bos taurus
gi|81673731 from Bos taurus
Fixed modifications: Carbamidomethyl (C)
Variable modifications: Oxidation (M)
Cleavage by Trypsin: cuts C-term side of KR unless next residue is P
Number of mass values searched: 65
Number of mass values matched: 14
Sequence Coverage: 50%
Matched peptides shown in **Bold Red**
1 MSTHRLVMVR **HGESTWNQEN RFCGWFDAEL SEKGAEEAK**K AAQAIKDAK**M**
51 **EFDICYTSVL KR**AIR**TLWTI LDGTDQMWLP VVR**TWRLNER HYGGLTGLNK
101 AETAAKHGEE QVKIWRR**SFD IPPPPMDEKH PYYK**SISKER RYAGLK**AGEL**
151 **PTCESLKDTI ARALPFWNDE IAPQIK**AGKR **VLIAAHGNSL R**GIVKHLEGM
201 SDQAIMELNL PTGIPIVYEL DQALKPTKPM R**FLGDEETVR** KAMEAVAAQG
251 KAK
Show predicted peptides also
Sort Peptides By Residue Number Increasing Mass Decreasing Mass
Start - End Observed Mr(expt) Mr(calc) ppm Miss Sequence
11 - 21 1357.5366 1356.5293 1356.5807 -38 0 R.HGESTWNQENR.F
22 - 33 1488.5796 1487.5723 1487.6391 -45 0 R.FCGWFDAELSEK.G
22 - 39 2073.8813 2072.8740 2072.9149 -20 1 R.FCGWFDAELSEKGAEEAK.K
50 - 62 1661.7306 1660.7233 1660.7953 -43 1 K.MEFDICYTSVLKR.A
50 - 62 1677.7246 1676.7173 1676.7902 -43 1 K.MEFDICYTSVLKR.A Oxidation (M)
66 - 83 2144.0859 2143.0786 2143.1136 -16 0 R.TLWTILDGTDQMWLPVVR.T
66 - 83 2160.0793 2159.0720 2159.1085 -17 0 R.TLWTILDGTDQMWLPVVR.T Oxidation (M)
118 - 129 1388.5970 1387.5897 1387.6330 -31 0 R.SFDIPPPPMDEK.H Oxidation (M)
118 - 134 2060.9321 2059.9248 2059.9713 -23 1 R.SFDIPPPPMDEKHPYYK.S
118 - 134 2076.9326 2075.9253 2075.9662 -20 1 R.SFDIPPPPMDEKHPYYK.S Oxidation (M)
147 - 162 1760.8190 1759.8117 1759.8774 -37 1 K.AGELPTCESLKDTIAR.A

163 - 176 1641.7878 1640.7805 1640.8562 -46 0 R.ALPFWNDEIAPQIK.A
181 - 191 1150.6451 1149.6378 1149.6618 -21 0 R.VLIAAHGNSLR.G
232 - 240 1065.5251 1064.5178 1064.5138 4 0 R.FLGDEETVR.K
No match to: 855.1088, 954.5054, 1037.5459, 1082.5668, 1129.4976, 1135.6187, 1232.5219, 1250.5270, 1278.5583, 1369.5300, 1385.57

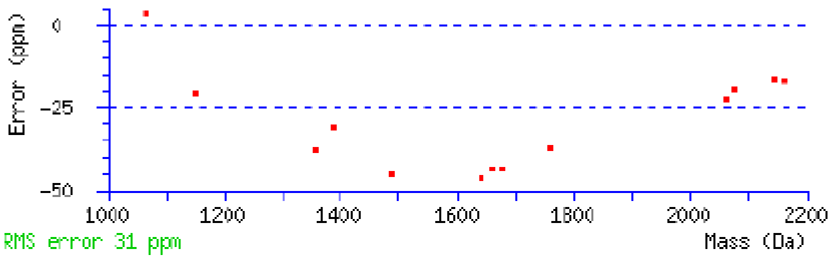

BAND 7

Mascot Search Results

User :
Email :
Search title : SampleSetID: 824, AnalysisID: 7242, MaldiWellID: 69630, SpectrumID: 154555, Path=\180719\MS\18-106 NCBI Mammalia
Database : NCBInr 20120508 (17919084 sequences; 6150218869 residues)
Taxonomy : Mammalia (mammals) (1061927 sequences)
Timestamp : 19 Jul 2018 at 13:06:53 GMT
Top Score : 246 for gi|61888856, triosephosphate isomerase [Bos taurus]

Mascot Score Histogram

Protein score is -10*Log(P), where P is the probability that the observed match is a random event.
Protein scores greater than 73 are significant (p<0.05).

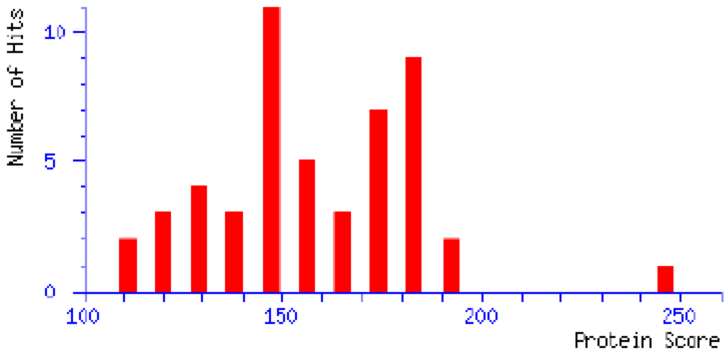

Protein Summary Report

Format As Protein Summary Help
Significance threshold p< 0.05 Max. number of hits 20
Re-Search All Search Unmatched

Index

Accession Mass Score Description
1. gi|61888856 26901 246 triosephosphate isomerase [Bos taurus]
2. gi|281338378 28423 191 hypothetical protein PANDA_011247 [Ailuropoda melanoleuca]
3. gi|301773724 28536 191 PREDICTED: triosephosphate isomerase-like, partial [Ailuropoda melanoleuca]
4. gi|296439800 20636 181 RecName: Full=Triosephosphate isomerase; Short=TIM; AltName: Full=Triose-phosphate isomerase
5. gi|999892 26807 179 Chain A, Crystal Structure Of Recombinant Human Triosephosphate Isomerase At 2.8 Angstroms Resolution. Triosephosphate Isomerase Related Hu
6. gi|4507645 26938 179 triosephosphate isomerase isoform 1 [Homo sapiens]
7. gi|136062 26979 179 RecName: Full=Triosephosphate isomerase; Short=TIM; AltName: Full=Triose-phosphate isomerase
8. gi|197097648 26941 179 triosephosphate isomerase [Pongo abelii]
9. gi|308082020 26983 179 triosephosphate isomerase [Canis lupus familiaris]
10. gi|62896835 26981 179 triosephosphate isomerase 1 variant [Homo sapiens]
11. gi|74222020 27048 179 unnamed protein product [Mus musculus]
12. gi|66360365 26951 179 Chain A, Human Triosephosphate Isomerase Of New Crystal Form
13. gi|262263205 26879 176 triosephosphate isomerase 1 [Sus scrofa]
14. gi|380798455 30922 173 triosephosphate isomerase isoform 2, partial [Macaca mulatta]
15. gi|194374069 23085 173 unnamed protein product [Homo sapiens]
16. gi|297261683 23198 170 PREDICTED: triosephosphate isomerase [Macaca mulatta]
17. gi|226529917 31057 170 triosephosphate isomerase isoform 2 [Homo sapiens]
18. gi|332249303 31055 170 PREDICTED: triosephosphate isomerase-like isoform 1 [Nomascus leucogenys]

19. gi|383421217 31124 170 triosephosphate isomerase isoform 2 [Macaca mulatta]
20. gi|348554958 30916 166 PREDICTED: triosephosphate isomerase-like [Cavia porcellus]

Results List

1. gi|61888856 Mass: 26901 Score: 246 Expect: 2.7e-019 Matches: 20
triosephosphate isomerase [Bos taurus]
Observed Mr(expt) Mr(calc) ppm Start End Miss Peptide
954.4771 953.4698 953.4760 -6.43 7 - 14 0 K.FFVGGNWK.M
1082.5686 1081.5613 1081.5709 -8.86 6 - 14 1 R.KFFVGGNWK.M
1137.5664 1136.5591 1136.5648 -5.00 60 - 69 0 K.IAVAAQNCYK.V
1204.5892 1203.5819 1203.5844 -2.03 195 - 206 0 K.SNVSDAVAQSAR.I
1274.6716 1273.6643 1273.6666 -1.82 150 - 160 1 K.VIADNVKDWSK.V
1326.6693 1325.6620 1325.6649 -2.21 207 - 219 0 R.IIYGGSVTGATCK.E
1458.7162 1457.7089 1457.7151 -4.21 101 - 113 0 R.HVFGESDELIGQK.V
1466.7181 1465.7108 1465.7161 -3.61 176 - 188 0 K.TATPQQAQEVHEK.L
1539.7876 1538.7803 1538.7841 -2.49 86 - 99 0 K.DLGATWVVLGHSER.R
1578.8268 1577.8195 1577.8301 -6.69 136 - 149 1 R.EAGITEKVVFEQTK.V
1602.8783 1601.8710 1601.8817 -6.68 161 - 175 0 K.VVLAYEPVWAIGTGK.T
1614.8206 1613.8133 1613.8162 -1.76 100 - 113 1 R.RHVFGESDELIGQK.V
1695.8857 1694.8784 1694.8853 -4.03 86 - 100 1 K.DLGATWVVLGHSERR.H
1735.9070 1734.8997 1734.9013 -0.89 176 - 190 1 K.TATPQQAQEVHEKLR.G
1807.9661 1806.9588 1806.9662 -4.07 114 - 131 0 K.VAHALAEGLGVIACIGEK.L
2192.0649 2191.0576 2191.0620 -1.98 34 - 53 0 K.VPADTEVVCAPPTAYIDFAR.Q
2321.2197 2320.2124 2320.2209 -3.65 114 - 135 1 K.VAHALAEGLGVIACIGEKLDER.E
3029.5950 3028.5877 3028.5757 3.97 220 - 248 0 K.ELASQPDVDGFLVGGASLKPEFVDIINAK.Q
3157.6272 3156.6199 3156.6343 -4.55 220 - 249 1 K.ELASQPDVDGFLVGGASLKPEFVDIINAKQ.-
3657.9189 3656.9116 3656.8508 16.6 20 - 53 1 K.NNLGELINTLNAAKVPADTEVVCAPPTAYIDFAR.Q
No match to: 855.0426, 1011.5015, 1263.6469, 1468.7056, 1470.7128, 1486.7546, 1560.8185, 1567.8143, 1571.7916, 1592.8057, 1596.8124,
1608.7935, 1630.8856,
1656.8295, 1658.7972, 1678.8551, 1685.8447, 1711.8246, 1727.8298, 1731.7592, 1752.9095, 1758.8915, 1763.8604, 1774.8783, 1825.8589,
1853.8964, 1903.9783,
1937.0145, 1956.0645, 2060.0796, 2101.0581, 2115.0203, 2206.0828, 2220.0999, 2225.1509, 2249.0862, 2271.0527, 2320.1787, 2329.1379,
2443.2695, 2708.3479,
3049.5991, 3136.5378, 3153.4795, 3785.9722
2. gi|281338378 Mass: 28423 Score: 191 Expect: 8.4e-014 Matches: 17
hypothetical protein PANDA_011247 [Ailuropoda melanoleuca]
Observed Mr(expt) Mr(calc) ppm Start End Miss Peptide
954.4771 953.4698 953.4760 -6.43 21 - 28 0 K.FFVGGNWK.M
1082.5686 1081.5613 1081.5709 -8.86 20 - 28 1 R.KFFVGGNWK.M
1137.5664 1136.5591 1136.5648 -5.00 74 - 83 0 K.IAVAAQNCYK.V
1274.6716 1273.6643 1273.6666 -1.82 164 - 174 1 K.VIADNVKDWSK.V
1326.6693 1325.6620 1325.6649 -2.21 221 - 233 0 R.IIYGGSVTGATCK.E
1458.7162 1457.7089 1457.7151 -4.21 115 - 127 0 R.HVFGESDELIGQK.V
1466.7181 1465.7108 1465.7161 -3.61 190 - 202 0 K.TATPQQAQEVHEK.L
1578.8268 1577.8195 1577.8301 -6.69 150 - 163 1 R.EAGITEKVVFEQTK.V
1602.8783 1601.8710 1601.8817 -6.68 175 - 189 0 K.VVLAYEPVWAIGTGK.T
1614.8206 1613.8133 1613.8162 -1.76 114 - 127 1 R.RHVFGESDELIGQK.V
1731.7592 1730.7519 1730.8080 -32.39 3 - 19 0 R.GAPDLQCVTASAMAPSR.K
1735.9070 1734.8997 1734.9013 -0.89 190 - 204 1 K.TATPQQAQEVHEKLR.G
1807.9661 1806.9588 1806.9662 -4.07 128 - 145 0 K.VAHALAEGLGVIACIGEK.L
2192.0649 2191.0576 2191.0620 -1.98 48 - 67 0 K.VPADTEVVCAPPTAYIDFAR.Q
2321.2197 2320.2124 2320.2209 -3.65 128 - 149 1 K.VAHALAEGLGVIACIGEKLDER.E
3029.5950 3028.5877 3028.5757 3.97 234 - 262 0 K.ELASQPDVDGFLVGGASLKPEFVDIINAK.Q
3157.6272 3156.6199 3156.6343 -4.55 234 - 263 1 K.ELASQPDVDGFLVGGASLKPEFVDIINAKQ.-
No match to: 855.0426, 1011.5015, 1204.5892, 1263.6469, 1468.7056, 1470.7128, 1486.7546, 1539.7876, 1560.8185, 1567.8143, 1571.7916,
1592.8057, 1596.8124,
1608.7935, 1630.8856, 1656.8295, 1658.7972, 1678.8551, 1685.8447, 1695.8857, 1711.8246, 1727.8298, 1752.9095, 1758.8915, 1763.8604,
1774.8783, 1825.8589,
1853.8964, 1903.9783, 1937.0145, 1956.0645, 2060.0796, 2101.0581, 2115.0203, 2206.0828, 2220.0999, 2225.1509, 2249.0862, 2271.0527,
2320.1787, 2329.1379,
2443.2695, 2708.3479, 3049.5991, 3136.5378, 3153.4795, 3657.9189, 3785.9722
3. gi|301773724 Mass: 28536 Score: 191 Expect: 8.4e-014 Matches: 17
PREDICTED: triosephosphate isomerase-like, partial [Ailuropoda melanoleuca]
Observed Mr(expt) Mr(calc) ppm Start End Miss Peptide
954.4771 953.4698 953.4760 -6.43 22 - 29 0 K.FFVGGNWK.M
1082.5686 1081.5613 1081.5709 -8.86 21 - 29 1 R.KFFVGGNWK.M
1137.5664 1136.5591 1136.5648 -5.00 75 - 84 0 K.IAVAAQNCYK.V
1274.6716 1273.6643 1273.6666 -1.82 165 - 175 1 K.VIADNVKDWSK.V
1326.6693 1325.6620 1325.6649 -2.21 222 - 234 0 R.IIYGGSVTGATCK.E
1458.7162 1457.7089 1457.7151 -4.21 116 - 128 0 R.HVFGESDELIGQK.V
1466.7181 1465.7108 1465.7161 -3.61 191 - 203 0 K.TATPQQAQEVHEK.L
1578.8268 1577.8195 1577.8301 -6.69 151 - 164 1 R.EAGITEKVVFEQTK.V
1602.8783 1601.8710 1601.8817 -6.68 176 - 190 0 K.VVLAYEPVWAIGTGK.T
1614.8206 1613.8133 1613.8162 -1.76 115 - 128 1 R.RHVFGESDELIGQK.V
1731.7592 1730.7519 1730.8080 -32.39 4 - 20 0 R.GAPDLQCVTASAMAPSR.K
1735.9070 1734.8997 1734.9013 -0.89 191 - 205 1 K.TATPQQAQEVHEKLR.G
1807.9661 1806.9588 1806.9662 -4.07 129 - 146 0 K.VAHALAEGLGVIACIGEK.L
2192.0649 2191.0576 2191.0620 -1.98 49 - 68 0 K.VPADTEVVCAPPTAYIDFAR.Q
2321.2197 2320.2124 2320.2209 -3.65 129 - 150 1 K.VAHALAEGLGVIACIGEKLDER.E
3029.5950 3028.5877 3028.5757 3.97 235 - 263 0 K.ELASQPDVDGFLVGGASLKPEFVDIINAK.Q
3157.6272 3156.6199 3156.6343 -4.55 235 - 264 1 K.ELASQPDVDGFLVGGASLKPEFVDIINAKQ.-
No match to: 855.0426, 1011.5015, 1204.5892, 1263.6469, 1468.7056, 1470.7128, 1486.7546, 1539.7876, 1560.8185, 1567.8143, 1571.7916,
1592.8057, 1596.8124,
1608.7935, 1630.8856, 1656.8295, 1658.7972, 1678.8551, 1685.8447, 1695.8857, 1711.8246, 1727.8298, 1752.9095, 1758.8915, 1763.8604,
1774.8783, 1825.8589,
1853.8964, 1903.9783, 1937.0145, 1956.0645, 2060.0796, 2101.0581, 2115.0203, 2206.0828, 2220.0999, 2225.1509, 2249.0862, 2271.0527,
2320.1787, 2329.1379,
2443.2695, 2708.3479, 3049.5991, 3136.5378, 3153.4795, 3657.9189, 3785.9722
4. gi|296439800 Mass: 20636 Score: 181 Expect: 8.4e-013 Matches: 15
RecName: Full=Triosephosphate isomerase; Short=TIM; AltName: Full=Triose-phosphate isomerase
Observed Mr(expt) Mr(calc) ppm Start End Miss Peptide
954.4771 953.4698 953.4760 -6.43 2 - 9 0 K.FFVGGNWK.M

1082.5686 1081.5613 1081.5709 -8.86 1 - 9 1 -.KFFVGGNWK.M
1137.5664 1136.5591 1136.5648 -5.00 47 - 56 0 R.IAVAAQNCYK.V
1326.6693 1325.6620 1325.6649 -2.21 146 - 158 0 R.IIYGGSVTGATCK.E
1458.7162 1457.7089 1457.7151 -4.21 74 - 86 0 R.HVFGESDELIGQK.L
1466.7181 1465.7108 1465.7161 -3.61 131 - 143 0 K.TATPQQAQEVHEK.L
1578.8268 1577.8195 1577.8301 -6.69 91 - 104 1 R.EAGITEKVVFEQTK.V
1602.8783 1601.8710 1601.8817 -6.68 116 - 130 0 K.VVLAYEPVWAIGTGK.T
1614.8206 1613.8133 1613.8162 -1.76 73 - 86 1 K.RHVFGESDELIGQK.L
1735.9070 1734.8997 1734.9013 -0.89 131 - 145 1 K.TATPQQAQEVHEKLR.I
1937.0145 1936.0072 1935.9328 38.4 10 - 26 1 K.MNGRCLGELICTLNAAK.L + Oxidation (M)
2192.0649 2191.0576 2191.1136 -25.55 112 - 130 1 K.DWCKVVLAYEPVWAIGTGK.T
2206.0828 2205.0755 2205.0776 -0.94 27 - 46 0 K.LPADTEVVCAPPTAYIDFAR.I
3029.5950 3028.5877 3028.5757 3.97 159 - 187 0 K.ELASQPDVDGFLVGGASLKPEFVDIINAK.Q
3157.6272 3156.6199 3156.6343 -4.55 159 - 188 1 K.ELASQPDVDGFLVGGASLKPEFVDIINAKQ.-
No match to: 855.0426, 1011.5015, 1204.5892, 1263.6469, 1274.6716, 1468.7056, 1470.7128, 1486.7546, 1539.7876, 1560.8185, 1567.8143, 1571.7916, 1592.8057,
1596.8124, 1608.7935, 1630.8856, 1656.8295, 1658.7972, 1678.8551, 1685.8447, 1695.8857, 1711.8246, 1727.8298, 1731.7592, 1752.9095, 1758.8915, 1763.8604,
1774.8783, 1807.9661, 1825.8589, 1853.8964, 1903.9783, 1956.0645, 2060.0796, 2101.0581, 2115.0203, 2220.0999, 2225.1509, 2249.0862, 2271.0527, 2320.1787,
2321.2197, 2329.1379, 2443.2695, 2708.3479, 3049.5991, 3136.5378, 3153.4795, 3657.9189, 3785.9722
5. gi|999892 Mass: 26807 Score: 179 Expect: 1.3e-012 Matches: 16
Chain A, Crystal Structure Of Recombinant Human Triosephosphate Isomerase At 2.8 Angstroms Resolution. Triosephosphate Isomerase Related Human Genetic Disorders And
Observed Mr(expt) Mr(calc) ppm Start End Miss Peptide
954.4771 953.4698 953.4760 -6.43 6 - 13 0 K.FFVGGNWK.M
1082.5686 1081.5613 1081.5709 -8.86 5 - 13 1 R.KFFVGGNWK.M
1137.5664 1136.5591 1136.5648 -5.00 59 - 68 0 K.IAVAAQNCYK.V
1274.6716 1273.6643 1273.6666 -1.82 149 - 159 1 K.VIADNVKDWSK.V
1326.6693 1325.6620 1325.6649 -2.21 206 - 218 0 R.IIYGGSVTGATCK.E
1458.7162 1457.7089 1457.7151 -4.21 100 - 112 0 R.HVFGESDELIGQK.V
1466.7181 1465.7108 1465.7161 -3.61 175 - 187 0 K.TATPQQAQEVHEK.L
1578.8268 1577.8195 1577.8301 -6.69 135 - 148 1 R.EAGITEKVVFEQTK.V
1602.8783 1601.8710 1601.8817 -6.68 160 - 174 0 K.VVLAYEPVWAIGTGK.T
1614.8206 1613.8133 1613.8162 -1.76 99 - 112 1 R.RHVFGESDELIGQK.V
1735.9070 1734.8997 1734.9013 -0.89 175 - 189 1 K.TATPQQAQEVHEKLR.G
1807.9661 1806.9588 1806.9662 -4.07 113 - 130 0 K.VAHALAEGLGVIACIGEK.L
2192.0649 2191.0576 2191.0620 -1.98 33 - 52 0 K.VPADTEVVCAPPTAYIDFAR.Q
2321.2197 2320.2124 2320.2209 -3.65 113 - 134 1 K.VAHALAEGLGVIACIGEKLDER.E
3029.5950 3028.5877 3028.5757 3.97 219 - 247 0 K.ELASQPDVDGFLVGGASLKPEFVDIINAK.Q
3157.6272 3156.6199 3156.6343 -4.55 219 - 248 1 K.ELASQPDVDGFLVGGASLKPEFVDIINAKQ.-
No match to: 855.0426, 1011.5015, 1204.5892, 1263.6469, 1468.7056, 1470.7128, 1486.7546, 1539.7876, 1560.8185, 1567.8143, 1571.7916, 1592.8057, 1596.8124,
1608.7935, 1630.8856, 1656.8295, 1658.7972, 1678.8551, 1685.8447, 1695.8857, 1711.8246, 1727.8298, 1731.7592, 1752.9095, 1758.8915, 1763.8604, 1774.8783,
1825.8589, 1853.8964, 1903.9783, 1937.0145, 1956.0645, 2060.0796, 2101.0581, 2115.0203, 2206.0828, 2220.0999, 2225.1509, 2249.0862, 2271.0527, 2320.1787,
2329.1379, 2443.2695, 2708.3479, 3049.5991, 3136.5378, 3153.4795, 3657.9189, 3785.9722
6. gi|4507645 Mass: 26938 Score: 179 Expect: 1.3e-012 Matches: 16
triosephosphate isomerase isoform 1 [Homo sapiens]
Observed Mr(expt) Mr(calc) ppm Start End Miss Peptide
954.4771 953.4698 953.4760 -6.43 7 - 14 0 K.FFVGGNWK.M
1082.5686 1081.5613 1081.5709 -8.86 6 - 14 1 R.KFFVGGNWK.M
1137.5664 1136.5591 1136.5648 -5.00 60 - 69 0 K.IAVAAQNCYK.V
1274.6716 1273.6643 1273.6666 -1.82 150 - 160 1 K.VIADNVKDWSK.V
1326.6693 1325.6620 1325.6649 -2.21 207 - 219 0 R.IIYGGSVTGATCK.E
1458.7162 1457.7089 1457.7151 -4.21 101 - 113 0 R.HVFGESDELIGQK.V
1466.7181 1465.7108 1465.7161 -3.61 176 - 188 0 K.TATPQQAQEVHEK.L
1578.8268 1577.8195 1577.8301 -6.69 136 - 149 1 R.EAGITEKVVFEQTK.V
1602.8783 1601.8710 1601.8817 -6.68 161 - 175 0 K.VVLAYEPVWAIGTGK.T
1614.8206 1613.8133 1613.8162 -1.76 100 - 113 1 R.RHVFGESDELIGQK.V
1735.9070 1734.8997 1734.9013 -0.89 176 - 190 1 K.TATPQQAQEVHEKLR.G
1807.9661 1806.9588 1806.9662 -4.07 114 - 131 0 K.VAHALAEGLGVIACIGEK.L
2192.0649 2191.0576 2191.0620 -1.98 34 - 53 0 K.VPADTEVVCAPPTAYIDFAR.Q
2321.2197 2320.2124 2320.2209 -3.65 114 - 135 1 K.VAHALAEGLGVIACIGEKLDER.E
3029.5950 3028.5877 3028.5757 3.97 220 - 248 0 K.ELASQPDVDGFLVGGASLKPEFVDIINAK.Q
3157.6272 3156.6199 3156.6343 -4.55 220 - 249 1 K.ELASQPDVDGFLVGGASLKPEFVDIINAKQ.-
No match to: 855.0426, 1011.5015, 1204.5892, 1263.6469, 1468.7056, 1470.7128, 1486.7546, 1539.7876, 1560.8185, 1567.8143, 1571.7916, 1592.8057, 1596.8124,
1608.7935, 1630.8856, 1656.8295, 1658.7972, 1678.8551, 1685.8447, 1695.8857, 1711.8246, 1727.8298, 1731.7592, 1752.9095, 1758.8915, 1763.8604, 1774.8783,
1825.8589, 1853.8964, 1903.9783, 1937.0145, 1956.0645, 2060.0796, 2101.0581, 2115.0203, 2206.0828, 2220.0999, 2225.1509, 2249.0862, 2271.0527, 2320.1787,
2329.1379, 2443.2695, 2708.3479, 3049.5991, 3136.5378, 3153.4795, 3657.9189, 3785.9722
7. gi|136062 Mass: 26979 Score: 179 Expect: 1.3e-012 Matches: 16
RecName: Full=Triosephosphate isomerase; Short=TIM; AltName: Full=Triose-phosphate isomerase
Observed Mr(expt) Mr(calc) ppm Start End Miss Peptide
954.4771 953.4698 953.4760 -6.43 7 - 14 0 K.FFVGGNWK.M
1082.5686 1081.5613 1081.5709 -8.86 6 - 14 1 R.KFFVGGNWK.M
1137.5664 1136.5591 1136.5648 -5.00 60 - 69 0 K.IAVAAQNCYK.V
1274.6716 1273.6643 1273.6666 -1.82 150 - 160 1 K.VIADNVKDWSK.V
1326.6693 1325.6620 1325.6649 -2.21 207 - 219 0 R.IIYGGSVTGATCK.E
1458.7162 1457.7089 1457.7151 -4.21 101 - 113 0 R.HVFGESDELIGQK.V
1466.7181 1465.7108 1465.7161 -3.61 176 - 188 0 K.TATPQQAQEVHEK.L
1578.8268 1577.8195 1577.8301 -6.69 136 - 149 1 R.EAGITEKVVFEQTK.V
1602.8783 1601.8710 1601.8817 -6.68 161 - 175 0 K.VVLAYEPVWAIGTGK.T
1614.8206 1613.8133 1613.8162 -1.76 100 - 113 1 R.RHVFGESDELIGQK.V
1735.9070 1734.8997 1734.9013 -0.89 176 - 190 1 K.TATPQQAQEVHEKLR.G
1807.9661 1806.9588 1806.9662 -4.07 114 - 131 0 K.VAHALAEGLGVIACIGEK.L
2192.0649 2191.0576 2191.0620 -1.98 34 - 53 0 K.VPADTEVVCAPPTAYIDFAR.Q
2321.2197 2320.2124 2320.2209 -3.65 114 - 135 1 K.VAHALAEGLGVIACIGEKLDER.E
3029.5950 3028.5877 3028.5757 3.97 220 - 248 0 K.ELASQPDVDGFLVGGASLKPEFVDIINAK.Q

3157.6272 3156.6199 3156.6343 -4.55 220 - 249 1 K.ELASQPDVDGFLVGGASLKPEFVDIINAKQ.-
No match to: 855.0426, 1011.5015, 1204.5892, 1263.6469, 1468.7056, 1470.7128, 1486.7546, 1539.7876, 1560.8185, 1567.8143, 1571.7916, 1592.8057, 1596.8124,
1608.7935, 1630.8856, 1656.8295, 1658.7972, 1678.8551, 1685.8447, 1695.8857, 1711.8246, 1727.8298, 1731.7592, 1752.9095, 1758.8915, 1763.8604, 1774.8783,
1825.8589, 1853.8964, 1903.9783, 1937.0145, 1956.0645, 2060.0796, 2101.0581, 2115.0203, 2206.0828, 2220.0999, 2225.1509, 2249.0862, 2271.0527, 2320.1787,
2329.1379, 2443.2695, 2708.3479, 3049.5991, 3136.5378, 3153.4795, 3657.9189, 3785.9722
8. gi|197097648 Mass: 26941 Score: 179 Expect: 1.3e-012 Matches: 16
triosephosphate isomerase [Pongo abelii]
Observed Mr(expt) Mr(calc) ppm Start End Miss Peptide
954.4771 953.4698 953.4760 -6.43 7 - 14 0 K.FFVGGNWK.M
1082.5686 1081.5613 1081.5709 -8.86 6 - 14 1 R.KFFVGGNWK.M
1137.5664 1136.5591 1136.5648 -5.00 60 - 69 0 K.IAVAAQNCYK.V
1274.6716 1273.6643 1273.6666 -1.82 150 - 160 1 K.VIADNVKDWSK.V
1326.6693 1325.6620 1325.6649 -2.21 207 - 219 0 R.IIYGGSVTGATCK.E
1458.7162 1457.7089 1457.7151 -4.21 101 - 113 0 R.HVFGESDELIGQK.V
1466.7181 1465.7108 1465.7161 -3.61 176 - 188 0 K.TATPQQAQEVHEK.L
1578.8268 1577.8195 1577.8301 -6.69 136 - 149 1 R.EAGITEKVVFEQTK.V
1602.8783 1601.8710 1601.8817 -6.68 161 - 175 0 K.VVLAYEPVWAIGTGK.T
1614.8206 1613.8133 1613.8162 -1.76 100 - 113 1 R.RHVFGESDELIGQK.V
1735.9070 1734.8997 1734.9013 -0.89 176 - 190 1 K.TATPQQAQEVHEKLR.G
1807.9661 1806.9588 1806.9662 -4.07 114 - 131 0 K.VAHALAEGLGVIACIGEK.L
2192.0649 2191.0576 2191.0620 -1.98 34 - 53 0 K.VPADTEVVCAPPTAYIDFAR.Q
2321.2197 2320.2124 2320.2209 -3.65 114 - 135 1 K.VAHALAEGLGVIACIGEKLDER.E
3029.5950 3028.5877 3028.5757 3.97 220 - 248 0 K.ELASQPDVDGFLVGGASLKPEFVDIINAK.Q
3157.6272 3156.6199 3156.6343 -4.55 220 - 249 1 K.ELASQPDVDGFLVGGASLKPEFVDIINAKQ.-
No match to: 855.0426, 1011.5015, 1204.5892, 1263.6469, 1468.7056, 1470.7128, 1486.7546, 1539.7876, 1560.8185, 1567.8143, 1571.7916, 1592.8057, 1596.8124,
1608.7935, 1630.8856, 1656.8295, 1658.7972, 1678.8551, 1685.8447, 1695.8857, 1711.8246, 1727.8298, 1731.7592, 1752.9095, 1758.8915, 1763.8604, 1774.8783,
1825.8589, 1853.8964, 1903.9783, 1937.0145, 1956.0645, 2060.0796, 2101.0581, 2115.0203, 2206.0828, 2220.0999, 2225.1509, 2249.0862, 2271.0527, 2320.1787,
2329.1379, 2443.2695, 2708.3479, 3049.5991, 3136.5378, 3153.4795, 3657.9189, 3785.9722
9. gi|308082020 Mass: 26983 Score: 179 Expect: 1.3e-012 Matches: 16
triosephosphate isomerase [Canis lupus familiaris]
Observed Mr(expt) Mr(calc) ppm Start End Miss Peptide
954.4771 953.4698 953.4760 -6.43 7 - 14 0 K.FFVGGNWK.M
1082.5686 1081.5613 1081.5709 -8.86 6 - 14 1 R.KFFVGGNWK.M
1137.5664 1136.5591 1136.5648 -5.00 60 - 69 0 K.IAVAAQNCYK.V
1274.6716 1273.6643 1273.6666 -1.82 150 - 160 1 K.VIADNVKDWSK.V
1326.6693 1325.6620 1325.6649 -2.21 207 - 219 0 R.IIYGGSVTGATCK.E
1458.7162 1457.7089 1457.7151 -4.21 101 - 113 0 R.HVFGESDELIGQK.V
1466.7181 1465.7108 1465.7161 -3.61 176 - 188 0 K.TATPQQAQEVHEK.L
1578.8268 1577.8195 1577.8301 -6.69 136 - 149 1 R.EAGITEKVVFEQTK.V
1602.8783 1601.8710 1601.8817 -6.68 161 - 175 0 K.VVLAYEPVWAIGTGK.T
1614.8206 1613.8133 1613.8162 -1.76 100 - 113 1 R.RHVFGESDELIGQK.V
1735.9070 1734.8997 1734.9013 -0.89 176 - 190 1 K.TATPQQAQEVHEKLR.G
1807.9661 1806.9588 1806.9662 -4.07 114 - 131 0 K.VAHALAEGLGVIACIGEK.L
2192.0649 2191.0576 2191.0620 -1.98 34 - 53 0 K.VPADTEVVCAPPTAYIDFAR.Q
2321.2197 2320.2124 2320.2209 -3.65 114 - 135 1 K.VAHALAEGLGVIACIGEKLDER.E
3029.5950 3028.5877 3028.5757 3.97 220 - 248 0 K.ELASQPDVDGFLVGGASLKPEFVDIINAK.Q
3157.6272 3156.6199 3156.6343 -4.55 220 - 249 1 K.ELASQPDVDGFLVGGASLKPEFVDIINAKQ.-
No match to: 855.0426, 1011.5015, 1204.5892, 1263.6469, 1468.7056, 1470.7128, 1486.7546, 1539.7876, 1560.8185, 1567.8143, 1571.7916, 1592.8057, 1596.8124,
1608.7935, 1630.8856, 1656.8295, 1658.7972, 1678.8551, 1685.8447, 1695.8857, 1711.8246, 1727.8298, 1731.7592, 1752.9095, 1758.8915, 1763.8604, 1774.8783,
1825.8589, 1853.8964, 1903.9783, 1937.0145, 1956.0645, 2060.0796, 2101.0581, 2115.0203, 2206.0828, 2220.0999, 2225.1509, 2249.0862, 2271.0527, 2320.1787,
2329.1379, 2443.2695, 2708.3479, 3049.5991, 3136.5378, 3153.4795, 3657.9189, 3785.9722
10. gi|62896835 Mass: 26981 Score: 179 Expect: 1.3e-012 Matches: 16
triosephosphate isomerase 1 variant [Homo sapiens]
Observed Mr(expt) Mr(calc) ppm Start End Miss Peptide
954.4771 953.4698 953.4760 -6.43 7 - 14 0 K.FFVGGNWK.M
1082.5686 1081.5613 1081.5709 -8.86 6 - 14 1 R.KFFVGGNWK.M
1137.5664 1136.5591 1136.5648 -5.00 60 - 69 0 K.IAVAAQNCYK.V
1274.6716 1273.6643 1273.6666 -1.82 150 - 160 1 K.VIADNVKDWSK.V
1326.6693 1325.6620 1325.6649 -2.21 207 - 219 0 R.IIYGGSVTGATCK.E
1458.7162 1457.7089 1457.7151 -4.21 101 - 113 0 R.HVFGESDELIGQK.V
1466.7181 1465.7108 1465.7161 -3.61 176 - 188 0 K.TATPQQAQEVHEK.L
1578.8268 1577.8195 1577.8301 -6.69 136 - 149 1 R.EAGITEKVVFEQTK.V
1602.8783 1601.8710 1601.8817 -6.68 161 - 175 0 K.VVLAYEPVWAIGTGK.T
1614.8206 1613.8133 1613.8162 -1.76 100 - 113 1 R.RHVFGESDELIGQK.V
1735.9070 1734.8997 1734.9013 -0.89 176 - 190 1 K.TATPQQAQEVHEKLR.G
1807.9661 1806.9588 1806.9662 -4.07 114 - 131 0 K.VAHALAEGLGVIACIGEK.L
2192.0649 2191.0576 2191.0620 -1.98 34 - 53 0 K.VPADTEVVCAPPTAYIDFAR.Q
2321.2197 2320.2124 2320.2209 -3.65 114 - 135 1 K.VAHALAEGLGVIACIGEKLDER.E
3029.5950 3028.5877 3028.5757 3.97 220 - 248 0 K.ELASQPDVDGFLVGGASLKPEFVDIINAK.Q
3157.6272 3156.6199 3156.6343 -4.55 220 - 249 1 K.ELASQPDVDGFLVGGASLKPEFVDIINAKQ.-
No match to: 855.0426, 1011.5015, 1204.5892, 1263.6469, 1468.7056, 1470.7128, 1486.7546, 1539.7876, 1560.8185, 1567.8143, 1571.7916, 1592.8057, 1596.8124,
1608.7935, 1630.8856, 1656.8295, 1658.7972, 1678.8551, 1685.8447, 1695.8857, 1711.8246, 1727.8298, 1731.7592, 1752.9095, 1758.8915, 1763.8604, 1774.8783,
1825.8589, 1853.8964, 1903.9783, 1937.0145, 1956.0645, 2060.0796, 2101.0581, 2115.0203, 2206.0828, 2220.0999, 2225.1509, 2249.0862, 2271.0527, 2320.1787,
2329.1379, 2443.2695, 2708.3479, 3049.5991, 3136.5378, 3153.4795, 3657.9189, 3785.9722
11. gi|74222020 Mass: 27048 Score: 179 Expect: 1.3e-012 Matches: 16
unnamed protein product [Mus musculus]
Observed Mr(expt) Mr(calc) ppm Start End Miss Peptide
954.4771 953.4698 953.4760 -6.43 7 - 14 0 K.FFVGGNWK.M
1082.5686 1081.5613 1081.5709 -8.86 6 - 14 1 R.KFFVGGNWK.M
1137.5664 1136.5591 1136.5648 -5.00 60 - 69 0 K.IAVAAQNCYK.V

1274.6716 1273.6643 1273.6666 -1.82 150 - 160 1 K.VIADNVKDWSK.V
1326.6693 1325.6620 1325.6649 -2.21 207 - 219 0 R.IIYGGSVTGATCK.E
1458.7162 1457.7089 1457.7151 -4.21 101 - 113 0 R.HVFGESDELIGQK.V
1466.7181 1465.7108 1465.7161 -3.61 176 - 188 0 K.TATPQQAQEVHEK.L
1539.7876 1538.7803 1538.7841 -2.49 86 - 99 0 K.DLGATWVVLGHSER.R
1578.8268 1577.8195 1577.8301 -6.69 136 - 149 1 R.EAGITEKVVFEQTK.V
1602.8783 1601.8710 1601.8817 -6.68 161 - 175 0 K.VVLAYEPVWAIGTGK.T
1614.8206 1613.8133 1613.8162 -1.76 100 - 113 1 R.RHVFGESDELIGQK.V
1695.8857 1694.8784 1694.8853 -4.03 86 - 100 1 K.DLGATWVVLGHSERR.H
1735.9070 1734.8997 1734.9013 -0.89 176 - 190 1 K.TATPQQAQEVHEKLR.G
3029.5950 3028.5877 3028.5757 3.97 220 - 248 0 K.ELASQPDVDGFLVGGASLKPEFVDIINAK.Q
3049.5991 3048.5918 3048.5451 15.3 191 - 219 1 R.GWLKPNVNDGVAQSTRIIYGGSVTGATCK.E
3157.6272 3156.6199 3156.6343 -4.55 220 - 249 1 K.ELASQPDVDGFLVGGASLKPEFVDIINAKQ.-
No match to: 855.0426, 1011.5015, 1204.5892, 1263.6469, 1468.7056, 1470.7128, 1486.7546, 1560.8185, 1567.8143, 1571.7916, 1592.8057, 1596.8124, 1608.7935,
1630.8856, 1656.8295, 1658.7972, 1678.8551, 1685.8447, 1711.8246, 1727.8298, 1731.7592, 1752.9095, 1758.8915, 1763.8604, 1774.8783, 1807.9661, 1825.8589,
1853.8964, 1903.9783, 1937.0145, 1956.0645, 2060.0796, 2101.0581, 2115.0203, 2192.0649, 2206.0828, 2220.0999, 2225.1509, 2249.0862, 2271.0527, 2320.1787,
2321.2197, 2329.1379, 2443.2695, 2708.3479, 3136.5378, 3153.4795, 3657.9189, 3785.9722
12. gi|66360365 Mass: 26951 Score: 179 Expect: 1.3e-012 Matches: 16
Chain A, Human Triosephosphate Isomerase Of New Crystal Form
Observed Mr(expt) Mr(calc) ppm Start End Miss Peptide
954.4771 953.4698 953.4760 -6.43 8 - 15 0 K.FFVGGNWK.M
1082.5686 1081.5613 1081.5709 -8.86 7 - 15 1 R.KFFVGGNWK.M
1137.5664 1136.5591 1136.5648 -5.00 61 - 70 0 K.IAVAAQNCYK.V
1274.6716 1273.6643 1273.6666 -1.82 151 - 161 1 K.VIADNVKDWSK.V
1326.6693 1325.6620 1325.6649 -2.21 208 - 220 0 R.IIYGGSVTGATCK.E
1458.7162 1457.7089 1457.7151 -4.21 102 - 114 0 R.HVFGESDELIGQK.V
1466.7181 1465.7108 1465.7161 -3.61 177 - 189 0 K.TATPQQAQEVHEK.L
1578.8268 1577.8195 1577.8301 -6.69 137 - 150 1 R.EAGITEKVVFEQTK.V
1602.8783 1601.8710 1601.8817 -6.68 162 - 176 0 K.VVLAYEPVWAIGTGK.T
1614.8206 1613.8133 1613.8162 -1.76 101 - 114 1 R.RHVFGESDELIGQK.V
1735.9070 1734.8997 1734.9013 -0.89 177 - 191 1 K.TATPQQAQEVHEKLR.G
1807.9661 1806.9588 1806.9662 -4.07 115 - 132 0 K.VAHALAEGLGVIACIGEK.L
2192.0649 2191.0576 2191.0620 -1.98 35 - 54 0 K.VPADTEVVCAPPTAYIDFAR.Q
2321.2197 2320.2124 2320.2209 -3.65 115 - 136 1 K.VAHALAEGLGVIACIGEKLDER.E
3029.5950 3028.5877 3028.5757 3.97 221 - 249 0 K.ELASQPDVDGFLVGGASLKPEFVDIINAK.Q
3157.6272 3156.6199 3156.6343 -4.55 221 - 250 1 K.ELASQPDVDGFLVGGASLKPEFVDIINAKQ.-
No match to: 855.0426, 1011.5015, 1204.5892, 1263.6469, 1468.7056, 1470.7128, 1486.7546, 1539.7876, 1560.8185, 1567.8143, 1571.7916, 1592.8057, 1596.8124,
1608.7935, 1630.8856, 1656.8295, 1658.7972, 1678.8551, 1685.8447, 1695.8857, 1711.8246, 1727.8298, 1731.7592, 1752.9095, 1758.8915, 1763.8604, 1774.8783,
1825.8589, 1853.8964, 1903.9783, 1937.0145, 1956.0645, 2060.0796, 2101.0581, 2115.0203, 2206.0828, 2220.0999, 2225.1509, 2249.0862, 2271.0527, 2320.1787,
2329.1379, 2443.2695, 2708.3479, 3049.5991, 3136.5378, 3153.4795, 3657.9189, 3785.9722
13. gi|262263205 Mass: 26879 Score: 176 Expect: 2.7e-012 Matches: 16
triosephosphate isomerase 1 [Sus scrofa]
Observed Mr(expt) Mr(calc) ppm Start End Miss Peptide
954.4771 953.4698 953.4760 -6.43 7 - 14 0 K.FFVGGNWK.M
1082.5686 1081.5613 1081.5709 -8.86 6 - 14 1 R.KFFVGGNWK.M
1137.5664 1136.5591 1136.5648 -5.00 60 - 69 0 K.IAVAAQNCYK.V
1326.6693 1325.6620 1325.6649 -2.21 207 - 219 0 R.IIYGGSVTGATCK.E
1458.7162 1457.7089 1457.7151 -4.21 101 - 113 0 R.HVFGESDELIGQK.V
1466.7181 1465.7108 1465.7161 -3.61 176 - 188 0 K.TATPQQAQEVHEK.L
1539.7876 1538.7803 1538.7841 -2.49 86 - 99 0 K.DLGATWVVLGHSER.R
1578.8268 1577.8195 1577.8301 -6.69 136 - 149 1 R.EAGITEKVVFEQTK.V
1602.8783 1601.8710 1601.8817 -6.68 161 - 175 0 K.VVLAYEPVWAIGTGK.T
1614.8206 1613.8133 1613.8162 -1.76 100 - 113 1 R.RHVFGESDELIGQK.V
1695.8857 1694.8784 1694.8853 -4.03 86 - 100 1 K.DLGATWVVLGHSERR.H
1735.9070 1734.8997 1734.9013 -0.89 176 - 190 1 K.TATPQQAQEVHEKLR.G
1807.9661 1806.9588 1806.9662 -4.07 114 - 131 0 K.VAHALAEGLGVIACIGEK.L
2206.0828 2205.0755 2205.0776 -0.94 34 - 53 0 K.LPADTEVVCAPPTAYIDFAR.Q
2321.2197 2320.2124 2320.2209 -3.65 114 - 135 1 K.VAHALAEGLGVIACIGEKLDER.E
3029.5950 3028.5877 3028.5757 3.97 220 - 248 0 K.ELASQPDVDGFLVGGASLKPEFVDIINAK.-
No match to: 855.0426, 1011.5015, 1204.5892, 1263.6469, 1274.6716, 1468.7056, 1470.7128, 1486.7546, 1560.8185, 1567.8143, 1571.7916, 1592.8057, 1596.8124,
1608.7935, 1630.8856, 1656.8295, 1658.7972, 1678.8551, 1685.8447, 1711.8246, 1727.8298, 1731.7592, 1752.9095, 1758.8915, 1763.8604, 1774.8783, 1825.8589,
1853.8964, 1903.9783, 1937.0145, 1956.0645, 2060.0796, 2101.0581, 2115.0203, 2192.0649, 2220.0999, 2225.1509, 2249.0862, 2271.0527, 2320.1787, 2329.1379,
2443.2695, 2708.3479, 3049.5991, 3136.5378, 3153.4795, 3157.6272, 3657.9189, 3785.9722
14. gi|380798455 Mass: 30922 Score: 173 Expect: 5.3e-012 Matches: 16
triosephosphate isomerase isoform 2, partial [Macaca mulatta]
Observed Mr(expt) Mr(calc) ppm Start End Miss Peptide
954.4771 953.4698 953.4760 -6.43 42 - 49 0 K.FFVGGNWK.M
1082.5686 1081.5613 1081.5709 -8.86 41 - 49 1 R.KFFVGGNWK.M
1137.5664 1136.5591 1136.5648 -5.00 95 - 104 0 K.IAVAAQNCYK.V
1274.6716 1273.6643 1273.6666 -1.82 185 - 195 1 K.VIADNVKDWSK.V
1326.6693 1325.6620 1325.6649 -2.21 242 - 254 0 R.IIYGGSVTGATCK.E
1458.7162 1457.7089 1457.7151 -4.21 136 - 148 0 R.HVFGESDELIGQK.V
1466.7181 1465.7108 1465.7161 -3.61 211 - 223 0 K.TATPQQAQEVHEK.L
1578.8268 1577.8195 1577.8301 -6.69 171 - 184 1 R.EAGITEKVVFEQTK.V
1602.8783 1601.8710 1601.8817 -6.68 196 - 210 0 K.VVLAYEPVWAIGTGK.T
1614.8206 1613.8133 1613.8162 -1.76 135 - 148 1 R.RHVFGESDELIGQK.V
1735.9070 1734.8997 1734.9013 -0.89 211 - 225 1 K.TATPQQAQEVHEKLR.G
2192.0649 2191.0576 2191.0620 -1.98 69 - 88 0 K.VPADTEVVCAPPTAYIDFAR.Q
2321.2197 2320.2124 2320.1845 12.0 149 - 170 0 K.VAHALAEGLGVIACIGEQLDER.E
3029.5950 3028.5877 3028.5757 3.97 255 - 283 0 K.ELASQPDVDGFLVGGASLKPEFVDIINAK.Q
3049.5991 3048.5918 3048.5550 12.1 149 - 177 1 K.VAHALAEGLGVIACIGEQLDEREAGITEK.V
3157.6272 3156.6199 3156.6343 -4.55 255 - 284 1 K.ELASQPDVDGFLVGGASLKPEFVDIINAKQ.-

No match to: 855.0426, 1011.5015, 1204.5892, 1263.6469, 1468.7056, 1470.7128, 1486.7546, 1539.7876, 1560.8185, 1567.8143, 1571.7916, 1592.8057, 1596.8124,
1608.7935, 1630.8856, 1656.8295, 1658.7972, 1678.8551, 1685.8447, 1695.8857, 1711.8246, 1727.8298, 1731.7592, 1752.9095, 1758.8915, 1763.8604, 1774.8783,
1807.9661, 1825.8589, 1853.8964, 1903.9783, 1937.0145, 1956.0645, 2060.0796, 2101.0581, 2115.0203, 2206.0828, 2220.0999, 2225.1509, 2249.0862, 2271.0527,
2320.1787, 2329.1379, 2443.2695, 2708.3479, 3136.5378, 3153.4795, 3657.9189, 3785.9722
15. gi|194374069 Mass: 23085 Score: 173 Expect: 5.3e-012 Matches: 15
unnamed protein product [Homo sapiens]
Observed Mr(expt) Mr(calc) ppm Start End Miss Peptide
954.4771 953.4698 953.4760 -6.43 7 - 14 0 K.FFVGGNWK.M
1082.5686 1081.5613 1081.5709 -8.86 6 - 14 1 R.KFFVGGNWK.M
1137.5664 1136.5591 1136.5648 -5.00 24 - 33 0 K.IAVAAQNCYK.V
1274.6716 1273.6643 1273.6666 -1.82 114 - 124 1 K.VIADNVKDWSK.V
1326.6693 1325.6620 1325.6649 -2.21 171 - 183 0 R.IIYGGSVTGATCK.E
1458.7162 1457.7089 1457.7151 -4.21 65 - 77 0 R.HVFGESDELIGQK.V
1466.7181 1465.7108 1465.7161 -3.61 140 - 152 0 K.TATPQQAQEVHEK.L
1578.8268 1577.8195 1577.8301 -6.69 100 - 113 1 R.EAGITEKVVFEQTK.V
1602.8783 1601.8710 1601.8817 -6.68 125 - 139 0 K.VVLAYEPVWAIGTGK.T
1614.8206 1613.8133 1613.8162 -1.76 64 - 77 1 R.RHVFGESDELIGQK.V
1735.9070 1734.8997 1734.9013 -0.89 140 - 154 1 K.TATPQQAQEVHEKLR.G
1807.9661 1806.9588 1806.9662 -4.07 78 - 95 0 K.VAHALAEGLGVIACIGEK.L
2321.2197 2320.2124 2320.2209 -3.65 78 - 99 1 K.VAHALAEGLGVIACIGEKLDER.E
3029.5950 3028.5877 3028.5757 3.97 184 - 212 0 K.ELASQPDVDGFLVGGASLKPEFVDIINAK.Q
3157.6272 3156.6199 3156.6343 -4.55 184 - 213 1 K.ELASQPDVDGFLVGGASLKPEFVDIINAKQ.-
No match to: 855.0426, 1011.5015, 1204.5892, 1263.6469, 1468.7056, 1470.7128, 1486.7546, 1539.7876, 1560.8185, 1567.8143, 1571.7916, 1592.8057, 1596.8124,
1608.7935, 1630.8856, 1656.8295, 1658.7972, 1678.8551, 1685.8447, 1695.8857, 1711.8246, 1727.8298, 1731.7592, 1752.9095, 1758.8915, 1763.8604, 1774.8783,
1825.8589, 1853.8964, 1903.9783, 1937.0145, 1956.0645, 2060.0796, 2101.0581, 2115.0203, 2192.0649, 2206.0828, 2220.0999, 2225.1509, 2249.0862, 2271.0527,
2320.1787, 2329.1379, 2443.2695, 2708.3479, 3049.5991, 3136.5378, 3153.4795, 3657.9189, 3785.9722
16. gi|297261683 Mass: 23198 Score: 170 Expect: 1.1e-011 Matches: 15
PREDICTED: triosephosphate isomerase [Macaca mulatta]
Observed Mr(expt) Mr(calc) ppm Start End Miss Peptide
954.4771 953.4698 953.4760 -6.43 7 - 14 0 K.FFVGGNWK.M
1082.5686 1081.5613 1081.5709 -8.86 6 - 14 1 R.KFFVGGNWK.M
1137.5664 1136.5591 1136.5648 -5.00 24 - 33 0 K.IAVAAQNCYK.V
1274.6716 1273.6643 1273.6666 -1.82 114 - 124 1 K.VIADNVKDWSK.V
1326.6693 1325.6620 1325.6649 -2.21 171 - 183 0 R.IIYGGSVTGATCK.E
1458.7162 1457.7089 1457.7151 -4.21 65 - 77 0 R.HVFGESDELIGQK.V
1466.7181 1465.7108 1465.7161 -3.61 140 - 152 0 K.TATPQQAQEVHEK.L
1578.8268 1577.8195 1577.8301 -6.69 100 - 113 1 R.EAGITEKVVFEQTK.V
1602.8783 1601.8710 1601.8817 -6.68 125 - 139 0 K.VVLAYEPVWAIGTGK.T
1614.8206 1613.8133 1613.8162 -1.76 64 - 77 1 R.RHVFGESDELIGQK.V
1735.9070 1734.8997 1734.9013 -0.89 140 - 154 1 K.TATPQQAQEVHEKLR.G
1807.9661 1806.9588 1806.9662 -4.07 78 - 95 0 K.VAHALAEGLGVIACIGEK.L
2321.2197 2320.2124 2320.2209 -3.65 78 - 99 1 K.VAHALAEGLGVIACIGEKLDER.E
3029.5950 3028.5877 3028.5757 3.97 184 - 212 0 K.ELASQPDVDGFLVGGASLKPEFVDIINAK.Q
3157.6272 3156.6199 3156.6343 -4.55 184 - 213 1 K.ELASQPDVDGFLVGGASLKPEFVDIINAKQ.-
No match to: 855.0426, 1011.5015, 1204.5892, 1263.6469, 1468.7056, 1470.7128, 1486.7546, 1539.7876, 1560.8185, 1567.8143, 1571.7916, 1592.8057, 1596.8124,
1608.7935, 1630.8856, 1656.8295, 1658.7972, 1678.8551, 1685.8447, 1695.8857, 1711.8246, 1727.8298, 1731.7592, 1752.9095, 1758.8915, 1763.8604, 1774.8783,
1825.8589, 1853.8964, 1903.9783, 1937.0145, 1956.0645, 2060.0796, 2101.0581, 2115.0203, 2192.0649, 2206.0828, 2220.0999, 2225.1509, 2249.0862, 2271.0527,
2320.1787, 2329.1379, 2443.2695, 2708.3479, 3049.5991, 3136.5378, 3153.4795, 3657.9189, 3785.9722
17. gi|226529917 Mass: 31057 Score: 170 Expect: 1.1e-011 Matches: 16
triosephosphate isomerase isoform 2 [Homo sapiens]
Observed Mr(expt) Mr(calc) ppm Start End Miss Peptide
954.4771 953.4698 953.4760 -6.43 44 - 51 0 K.FFVGGNWK.M
1082.5686 1081.5613 1081.5709 -8.86 43 - 51 1 R.KFFVGGNWK.M
1137.5664 1136.5591 1136.5648 -5.00 97 - 106 0 K.IAVAAQNCYK.V
1274.6716 1273.6643 1273.6666 -1.82 187 - 197 1 K.VIADNVKDWSK.V
1326.6693 1325.6620 1325.6649 -2.21 244 - 256 0 R.IIYGGSVTGATCK.E
1458.7162 1457.7089 1457.7151 -4.21 138 - 150 0 R.HVFGESDELIGQK.V
1466.7181 1465.7108 1465.7161 -3.61 213 - 225 0 K.TATPQQAQEVHEK.L
1578.8268 1577.8195 1577.8301 -6.69 173 - 186 1 R.EAGITEKVVFEQTK.V
1602.8783 1601.8710 1601.8817 -6.68 198 - 212 0 K.VVLAYEPVWAIGTGK.T
1614.8206 1613.8133 1613.8162 -1.76 137 - 150 1 R.RHVFGESDELIGQK.V
1735.9070 1734.8997 1734.9013 -0.89 213 - 227 1 K.TATPQQAQEVHEKLR.G
1807.9661 1806.9588 1806.9662 -4.07 151 - 168 0 K.VAHALAEGLGVIACIGEK.L
2192.0649 2191.0576 2191.0620 -1.98 71 - 90 0 K.VPADTEVVCAPPTAYIDFAR.Q
2321.2197 2320.2124 2320.2209 -3.65 151 - 172 1 K.VAHALAEGLGVIACIGEKLDER.E
3029.5950 3028.5877 3028.5757 3.97 257 - 285 0 K.ELASQPDVDGFLVGGASLKPEFVDIINAK.Q
3157.6272 3156.6199 3156.6343 -4.55 257 - 286 1 K.ELASQPDVDGFLVGGASLKPEFVDIINAKQ.-
No match to: 855.0426, 1011.5015, 1204.5892, 1263.6469, 1468.7056, 1470.7128, 1486.7546, 1539.7876, 1560.8185, 1567.8143, 1571.7916, 1592.8057, 1596.8124,
1608.7935, 1630.8856, 1656.8295, 1658.7972, 1678.8551, 1685.8447, 1695.8857, 1711.8246, 1727.8298, 1731.7592, 1752.9095, 1758.8915, 1763.8604, 1774.8783,
1825.8589, 1853.8964, 1903.9783, 1937.0145, 1956.0645, 2060.0796, 2101.0581, 2115.0203, 2206.0828, 2220.0999, 2225.1509, 2249.0862, 2271.0527, 2320.1787,
2329.1379, 2443.2695, 2708.3479, 3049.5991, 3136.5378, 3153.4795, 3657.9189, 3785.9722
18. gi|332249303 Mass: 31055 Score: 170 Expect: 1.1e-011 Matches: 16
PREDICTED: triosephosphate isomerase-like isoform 1 [Nomascus leucogenys]
Observed Mr(expt) Mr(calc) ppm Start End Miss Peptide
954.4771 953.4698 953.4760 -6.43 44 - 51 0 K.FFVGGNWK.M
1082.5686 1081.5613 1081.5709 -8.86 43 - 51 1 R.KFFVGGNWK.M
1137.5664 1136.5591 1136.5648 -5.00 97 - 106 0 K.IAVAAQNCYK.V
1274.6716 1273.6643 1273.6666 -1.82 187 - 197 1 K.VIADNVKDWSK.V
1326.6693 1325.6620 1325.6649 -2.21 244 - 256 0 R.IIYGGSVTGATCK.E
1458.7162 1457.7089 1457.7151 -4.21 138 - 150 0 R.HVFGESDELIGQK.V

1466.7181 1465.7108 1465.7161 -3.61 213 - 225 0 K.TATPQQAQEVHEK.L
1578.8268 1577.8195 1577.8301 -6.69 173 - 186 1 R.EAGITEKVVFEQTK.V
1602.8783 1601.8710 1601.8817 -6.68 198 - 212 0 K.VVLAYEPVWAIGTGK.T
1614.8206 1613.8133 1613.8162 -1.76 137 - 150 1 R.RHVFGESDELIGQK.V
1735.9070 1734.8997 1734.9013 -0.89 213 - 227 1 K.TATPQQAQEVHEKLR.G
1807.9661 1806.9588 1806.9662 -4.07 151 - 168 0 K.VAHALAEGLGVIACIGEK.L
2192.0649 2191.0576 2191.0620 -1.98 71 - 90 0 K.VPADTEVVCAPPTAYIDFAR.Q
2321.2197 2320.2124 2320.2209 -3.65 151 - 172 1 K.VAHALAEGLGVIACIGEKLDER.E
3029.5950 3028.5877 3028.5757 3.97 257 - 285 0 K.ELASQPDVDGFLVGGASLKPEFVDIINAK.Q
3157.6272 3156.6199 3156.6343 -4.55 257 - 286 1 K.ELASQPDVDGFLVGGASLKPEFVDIINAKQ.-
No match to: 855.0426, 1011.5015, 1204.5892, 1263.6469, 1468.7056, 1470.7128, 1486.7546, 1539.7876, 1560.8185, 1567.8143, 1571.7916, 1592.8057, 1596.8124,
1608.7935, 1630.8856, 1656.8295, 1658.7972, 1678.8551, 1685.8447, 1695.8857, 1711.8246, 1727.8298, 1731.7592, 1752.9095, 1758.8915, 1763.8604, 1774.8783,
1825.8589, 1853.8964, 1903.9783, 1937.0145, 1956.0645, 2060.0796, 2101.0581, 2115.0203, 2206.0828, 2220.0999, 2225.1509, 2249.0862, 2271.0527, 2320.1787,
2329.1379, 2443.2695, 2708.3479, 3049.5991, 3136.5378, 3153.4795, 3657.9189, 3785.9722
19. gi|383421217 Mass: 31124 Score: 170 Expect: 1.1e-011 Matches: 16
triosephosphate isomerase isoform 2 [Macaca mulatta]
Observed Mr(expt) Mr(calc) ppm Start End Miss Peptide
954.4771 953.4698 953.4760 -6.43 44 - 51 0 K.FFVGGNWK.M
1082.5686 1081.5613 1081.5709 -8.86 43 - 51 1 R.KFFVGGNWK.M
1137.5664 1136.5591 1136.5648 -5.00 97 - 106 0 K.IAVAAQNCYK.V
1274.6716 1273.6643 1273.6666 -1.82 187 - 197 1 K.VIADNVKDWSK.V
1326.6693 1325.6620 1325.6649 -2.21 244 - 256 0 R.IIYGGSVTGATCK.E
1458.7162 1457.7089 1457.7151 -4.21 138 - 150 0 R.HVFGESDELIGQK.V
1466.7181 1465.7108 1465.7161 -3.61 213 - 225 0 K.TATPQQAQEVHEK.L
1578.8268 1577.8195 1577.8301 -6.69 173 - 186 1 R.EAGITEKVVFEQTK.V
1602.8783 1601.8710 1601.8817 -6.68 198 - 212 0 K.VVLAYEPVWAIGTGK.T
1614.8206 1613.8133 1613.8162 -1.76 137 - 150 1 R.RHVFGESDELIGQK.V
1735.9070 1734.8997 1734.9013 -0.89 213 - 227 1 K.TATPQQAQEVHEKLR.G
1807.9661 1806.9588 1806.9662 -4.07 151 - 168 0 K.VAHALAEGLGVIACIGEK.L
2192.0649 2191.0576 2191.0620 -1.98 71 - 90 0 K.VPADTEVVCAPPTAYIDFAR.Q
2321.2197 2320.2124 2320.2209 -3.65 151 - 172 1 K.VAHALAEGLGVIACIGEKLDER.E
3029.5950 3028.5877 3028.5757 3.97 257 - 285 0 K.ELASQPDVDGFLVGGASLKPEFVDIINAK.Q
3157.6272 3156.6199 3156.6343 -4.55 257 - 286 1 K.ELASQPDVDGFLVGGASLKPEFVDIINAKQ.-
No match to: 855.0426, 1011.5015, 1204.5892, 1263.6469, 1468.7056, 1470.7128, 1486.7546, 1539.7876, 1560.8185, 1567.8143, 1571.7916, 1592.8057, 1596.8124,
1608.7935, 1630.8856, 1656.8295, 1658.7972, 1678.8551, 1685.8447, 1695.8857, 1711.8246, 1727.8298, 1731.7592, 1752.9095, 1758.8915, 1763.8604, 1774.8783,
1825.8589, 1853.8964, 1903.9783, 1937.0145, 1956.0645, 2060.0796, 2101.0581, 2115.0203, 2206.0828, 2220.0999, 2225.1509, 2249.0862, 2271.0527, 2320.1787,
2329.1379, 2443.2695, 2708.3479, 3049.5991, 3136.5378, 3153.4795, 3657.9189, 3785.9722
20. gi|348554958 Mass: 30916 Score: 166 Expect: 2.7e-011 Matches: 16
PREDICTED: triosephosphate isomerase-like [Cavia porcellus]
Observed Mr(expt) Mr(calc) ppm Start End Miss Peptide
954.4771 953.4698 953.4760 -6.43 44 - 51 0 K.FFVGGNWK.M
1082.5686 1081.5613 1081.5709 -8.86 43 - 51 1 R.KFFVGGNWK.M
1137.5664 1136.5591 1136.5648 -5.00 97 - 106 0 K.IAVAAQNCYK.V
1326.6693 1325.6620 1325.6649 -2.21 244 - 256 0 R.IIYGGSVTGATCK.E
1458.7162 1457.7089 1457.7151 -4.21 138 - 150 0 R.HVFGESDELIGQK.V
1466.7181 1465.7108 1465.7161 -3.61 213 - 225 0 K.TATPQQAQEVHEK.L
1539.7876 1538.7803 1538.7841 -2.49 123 - 136 0 K.DLGATWVVLGHSER.R
1578.8268 1577.8195 1577.8301 -6.69 173 - 186 1 R.EAGITEKVVFEQTK.V
1602.8783 1601.8710 1601.8817 -6.68 198 - 212 0 K.VVLAYEPVWAIGTGK.T
1614.8206 1613.8133 1613.8162 -1.76 137 - 150 1 R.RHVFGESDELIGQK.V
1695.8857 1694.8784 1694.8853 -4.03 123 - 137 1 K.DLGATWVVLGHSERR.H
1735.9070 1734.8997 1734.9013 -0.89 213 - 227 1 K.TATPQQAQEVHEKLR.G
1807.9661 1806.9588 1806.9662 -4.07 151 - 168 0 K.VAHALAEGLGVIACIGEK.L
2192.0649 2191.0576 2191.0620 -1.98 71 - 90 0 K.VPADTEVVCAPPTAYIDFAR.Q
2321.2197 2320.2124 2320.2209 -3.65 151 - 172 1 K.VAHALAEGLGVIACIGEKLDER.E
3029.5950 3028.5877 3028.5757 3.97 257 - 285 0 K.ELASQPDVDGFLVGGASLKPEFVDIINAK.H
No match to: 855.0426, 1011.5015, 1204.5892, 1263.6469, 1274.6716, 1468.7056, 1470.7128, 1486.7546, 1560.8185, 1567.8143, 1571.7916, 1592.8057, 1596.8124,
1608.7935, 1630.8856, 1656.8295, 1658.7972, 1678.8551, 1685.8447, 1711.8246, 1727.8298, 1731.7592, 1752.9095, 1758.8915, 1763.8604, 1774.8783, 1825.8589,
1853.8964, 1903.9783, 1937.0145, 1956.0645, 2060.0796, 2101.0581, 2115.0203, 2206.0828, 2220.0999, 2225.1509, 2249.0862, 2271.0527, 2320.1787, 2329.1379,
2443.2695, 2708.3479, 3049.5991, 3136.5378, 3153.4795, 3157.6272, 3657.9189, 3785.9722

Search Parameters

Type of search : Peptide Mass Fingerprint
Enzyme : Trypsin
Fixed modifications : Carbamidomethyl (C)
Variable modifications : Oxidation (M)
Mass values : Monoisotopic
Protein Mass : Unrestricted
Peptide Mass Tolerance : ± 50 ppm
Peptide Charge State : 1+
Max Missed Cleavages : 1
Number of queries : 65

Mascot: http://www.matrixscience.com/

COVERAGE BAND 7

Mascot Search Results

Protein View

triosephosphate isomerase [Bos taurus]
Nominal mass (Mr): 26901; Calculated pI value: 6.45
NCBI BLAST search of gi|61888856 against nr
Unformatted sequence string for pasting into other applications
Taxonomy: Bos taurus
Links to retrieve other entries containing this sequence from NCBI Entrez:
gi|75070019 from Bos taurus
gi|59858493 from Bos taurus
gi|74267824 from Bos taurus
gi|296487109 from Bos taurus
Fixed modifications: Carbamidomethyl (C)
Variable modifications: Oxidation (M)
Cleavage by Trypsin: cuts C-term side of KR unless next residue is P
Number of mass values searched: 65
Number of mass values matched: 20
Sequence Coverage: 85%
Matched peptides shown in **Bold Red**

```
1 MAPSRKFFVG GNWKMNGRKN NLGELINTLN AAKVPADTEV VCAPPTAYID
51 FARQKLDPKI AVAAQNCYKV ANGAFTGEIS PGMIKDLGAT WVVLGHSERR
101 HVFGESDELI GQKVAHALAE GLGVIACIGE KLDEREAGIT EKVVFEQTKV
151 IADNVKDWSK VVLAYEPVWA IGTGKTATPQ QAQEVHEKLR GWLKSNVSDA
201 VAQSARIIYG GSVTGATCKE LASQPDVDGF LVGGASLKPE FVDIINAKQ
```

Show predicted peptides also
Sort Peptides By Residue Number Increasing Mass Decreasing Mass

Start - End	Observed	Mr(expt)	Mr(calc)	ppm	Miss	Sequence
6 - 14	1082.5686	1081.5613	1081.5709	-9	1	R.KFFVGGNWK.M
7 - 14	954.4771	953.4698	953.4760	-6	0	K.FFVGGNWK.M
20 - 53	3657.9189	3656.9116	3656.8508	17	1	K.NNLGELINTLNAAKVPADTEVVCAPPTAYIDFAR.Q
34 - 53	2192.0649	2191.0576	2191.0620	-2	0	K.VPADTEVVCAPPTAYIDFAR.Q
60 - 69	1137.5664	1136.5591	1136.5648	-5	0	K.IAVAAQNCYK.V
86 - 99	1539.7876	1538.7803	1538.7841	-2	0	K.DLGATWVVLGHSER.R
86 - 100	1695.8857	1694.8784	1694.8853	-4	1	K.DLGATWVVLGHSERR.H
100 - 113	1614.8206	1613.8133	1613.8162	-2	1	R.RHVFGESDELIGQK.V
101 - 113	1458.7162	1457.7089	1457.7151	-4	0	R.HVFGESDELIGQK.V
114 - 131	1807.9661	1806.9588	1806.9662	-4	0	K.VAHALAEGLGVIACIGEK.L
114 - 135	2321.2197	2320.2124	2320.2209	-4	1	K.VAHALAEGLGVIACIGEKLDER.E
136 - 149	1578.8268	1577.8195	1577.8301	-7	1	R.EAGITEKVVFEQTK.V
150 - 160	1274.6716	1273.6643	1273.6666	-2	1	K.VIADNVKDWSK.V
161 - 175	1602.8783	1601.8710	1601.8817	-7	0	K.VVLAYEPVWAIGTGK.T
176 - 188	1466.7181	1465.7108	1465.7161	-4	0	K.TATPQQAQEVHEK.L
176 - 190	1735.9070	1734.8997	1734.9013	-1	1	K.TATPQQAQEVHEKLR.G
195 - 206	1204.5892	1203.5819	1203.5844	-2	0	K.SNVSDAVAQSAR.I
207 - 219	1326.6693	1325.6620	1325.6649	-2	0	R.IIYGGSVTGATCK.E
220 - 248	3029.5950	3028.5877	3028.5757	4	0	K.ELASQPDVDGFLVGGASLKPEFVDIINAK.Q
220 - 249	3157.6272	3156.6199	3156.6343	-5	1	K.ELASQPDVDGFLVGGASLKPEFVDIINAKQ.-

No match to: 855.0426, 1011.5015, 1263.6469, 1468.7056, 1470.7128, 1486.7546, 1560.8185, 1567.8143, 1571.7916, 1592.8057, 1596.8

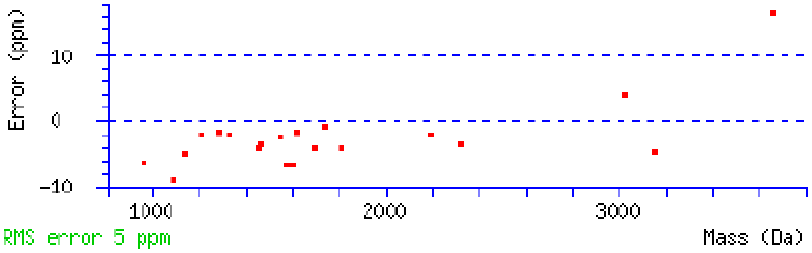

Supplement: Supplementary file 1 [file foods-09-00170-s001.zip › Supplementary Material 2.pdf]
