# Supplementary material for: Residues of Fluoroquinolone Antibiotics Induce Carbonylation and Reduce In Vitro Digestion of Sarcoplasmic and Myofibrillar Beef Proteins
Source: Foods. 2020 Feb 11;9(2):170. doi: 10.3390/foods9020170 (PMC7074055; doi:10.3390/foods9020170)

Supplementary Material 3

Results of search in Mascot for Peptide Mass Fingerprint (PMF) and MS/MS Data of sarcoplasmic proteins from beef

Residues of fluoroquinolone antibiotics induce carbonylation and reduce in vitro digestion of sarcoplasmic and myofibrillar beef proteins

Johana Márquez-Lázaro[1], Darío Méndez-Cuadro[1] a and Erika Rodríguez-Cavallo [1*]

[1]Analytical Chemistry and Biomedicine Group, University of Cartagena, Cartagena de Indias, Colombia; jmarquezl1@unicartagena.edu.co (J.M.-L); dmendezc@unicartagena.edu.co (D.M.-C); erodriguezc1@unicartagena.edu.co (E.R.-C)

*Correspondence: erodriguezc1@unicartagena.edu.co

MYOFIBRILLAR PROTEINS

BAND 3

Mascot Search Results

Email :
Search title : SampleSetID: 824, AnalysisID: 7242, MaldiWellID: 69606, SpectrumID: 154531, Path=\180719\MS\18-106 NCBI Mammalia
Database : NCBInr 20120508 (17919084 sequences; 6150218869 residues)
Taxonomy : Mammalia (mammals) (1061927 sequences)
Timestamp : 19 Jul 2018 at 11:57:03 GMT
Top Score : 111 for gi|160425243, myosin-binding protein C, slow-type [Bos taurus]

Mascot Score Histogram

Protein score is -10*Log(P), where P is the probability that the observed match is a random event.
Protein scores greater than 73 are significant (p<0.05).

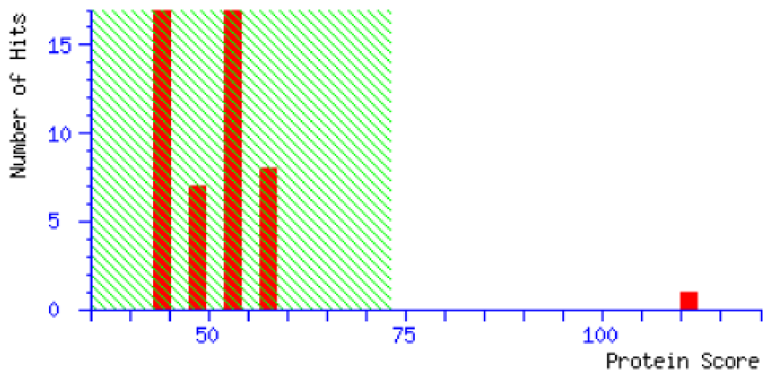

Protein Summary Report

Format As Protein Summary Help
Significance threshold p< 0.05 Max. number of hits 20
Re-Search All Search Unmatched

Index

Accession Mass Score Description
1. gi|160425243 134923 111 myosin-binding protein C, slow-type [Bos taurus]
2. gi|293351026 9162 58 PREDICTED: 40S ribosomal protein S21-like [Rattus norvegicus]
3. gi|281354139 123222 58 hypothetical protein PANDA_017349 [Ailuropoda melanoleuca]
4. gi|355564610 132769 58 hypothetical protein EGK_04106 [Macaca mulatta]
5. gi|355786447 132797 58 hypothetical protein EGM_03664 [Macaca fascicularis]
6. gi|109098449 132640 58 PREDICTED: myosin-binding protein C, slow-type-like [Macaca mulatta]
7. gi|2735295 5517 57 deoxyuridine triphosphate nucleotidohydrolase, nuclear isoform [Homo sapiens]
8. gi|301784296 133817 56 PREDICTED: myosin-binding protein C, slow-type-like [Ailuropoda melanoleuca]
9. gi|351695270 132081 56 Zinc finger protein 91, partial [Heterocephalus glaber]
10. gi|26334151 78962 55 unnamed protein product [Mus musculus]
11. gi|344267648 134226 55 PREDICTED: myosin-binding protein C, slow-type [Loxodonta africana]
12. gi|115496194 34281 54 complement C1q and tumor necrosis factor-related protein 9 precursor [Bos taurus]
13. gi|332257525 96495 54 PREDICTED: FYVE, RhoGEF and PH domain-containing protein 4 isoform 3 [Nomascus leucogenys]
14. gi|120952829 55358 54 zinc finger protein 331 [Homo sapiens]
15. gi|332221246 55427 54 PREDICTED: zinc finger protein 331 isoform 1 [Nomascus leucogenys]
16. gi|332857190 55461 54 PREDICTED: zinc finger protein 331 isoform 1 [Pan troglodytes]
17. gi|343958786 55431 54 zinc finger protein 331 [Pan troglodytes]
18. gi|348562043 87299 54 PREDICTED: FYVE, RhoGEF and PH domain-containing protein 4-like [Cavia porcellus]
19. gi|351699086 24574 54 Putative deoxyribonuclease TATDN1 [Heterocephalus glaber]
20. gi|297692730 96695 54 PREDICTED: myosin-binding protein C, slow-type-like, partial [Pongo abelii]

Results List

1. gi|160425243 Mass: 134923 Score: 111 Expect: 8.4e-006 Matches: 23
myosin-binding protein C, slow-type [Bos taurus]
Observed Mr(expt) Mr(calc) ppm Start End Miss Peptide
986.4511 985.4438 985.4253 18.8 191 - 198 0 K.ENYAGNYR.C
1001.5549 1000.5476 1000.5454 2.23 446 - 453 1 R.YHIRVEGK.K
1150.5973 1149.5900 1149.6030 -11.27 975 - 984 0 K.FVETASIDIR.V
1207.6119 1206.6046 1206.6245 -16.44 852 - 862 0 K.FTITGLPTDSR.I
1294.6444 1293.6371 1293.6565 -14.96 946 - 956 0 R.NSETDTIVFIR.K
1296.6675 1295.6602 1295.6833 -17.84 434 - 445 1 K.NGEEIIPGPKSR.Y
1300.6644 1299.6571 1299.6935 -28.00 166 - 175 1 K.HLQLKETFER.H
1310.6544 1309.6471 1309.6700 -17.47 740 - 750 1 K.KMIEGVAYEVR.I + Oxidation (M)
1318.7582 1317.7509 1317.7656 -11.16 128 - 139 1 K.VGENITFIAKVK.A
1431.7261 1430.7188 1430.7518 -23.03 314 - 326 1 R.ILDPAYQVDKGGR.V
1436.7306 1435.7233 1435.7671 -30.49 850 - 862 1 K.TKFTITGLPTDSR.I
1453.7384 1452.7311 1452.7685 -25.70 933 - 945 1 K.DGAPIDKNQINIR.N
1486.8057 1485.7984 1485.7901 5.59 179 - 190 1 R.VYTFEMQIIKAK.E + Oxidation (M)
1545.8552 1544.8479 1544.8926 -28.91 600 - 613 1 K.LRLEIPISGEPPPK.A
1554.7094 1553.7021 1553.7330 -19.89 1152 - 1164 0 R.MFSNQGVCTLEIR.K
1570.7126 1569.7053 1569.7280 -14.41 1152 - 1164 0 R.MFSNQGVCTLEIR.K + Oxidation (M)
1734.7813 1733.7740 1733.7984 -14.06 1032 - 1044 0 K.SMEWFTVIEHYHR.T
1750.7714 1749.7641 1749.7933 -16.69 1032 - 1044 0 K.SMEWFTVIEHYHR.T + Oxidation (M)
1837.8254 1836.8181 1836.8378 -10.69 235 - 252 0 R.SGEGQDDAGELDFSGLLK.R
1976.9685 1975.9612 1975.9891 -14.10 1045 - 1061 0 R.TSATITELVIGNEYYFR.V
1993.9484 1992.9411 1992.9389 1.14 235 - 253 1 R.SGEGQDDAGELDFSGLLKR.R
2195.1038 2194.0965 2194.1157 -8.76 255 - 272 0 R.EVKPQEEQPEVDVWELLK.N
2605.5559 2604.5486 2604.4792 26.7 910 - 932 0 R.VGEAINLVIPFQGKPRPELIWTK.D
No match to: 855.0688, 971.4968, 980.5079, 1028.5073, 1082.5900, 1088.5948, 1109.5083, 1110.5568,
1232.5300, 1234.6511,
1314.6636, 1320.5607, 1332.6127, 1333.6133, 1345.6831, 1361.6106, 1377.6110, 1419.7086, 1434.7136,
1452.6949, 1473.7191,
1475.7252, 1489.7124, 1516.7328, 1528.7238, 1558.7601, 1584.8186, 1607.7816, 1638.8127, 1658.8298,
1675.8585, 1686.7861,
1766.8878, 1774.8828, 1835.8412, 1871.9175, 1880.8855, 2129.0681, 2177.1699, 2225.1597, 2612.2476,
3153.6775
2. gi|293351026 Mass: 9162 Score: 58 Expect: 1.5 Matches: 6
PREDICTED: 40S ribosomal protein S21-like [Rattus norvegicus]
Observed Mr(expt) Mr(calc) ppm Start End Miss Peptide
1001.5549 1000.5476 1000.5917 -44.02 72 - 81 1 R.LAKADGIVSK.N
1150.5973 1149.5900 1149.5679 19.2 42 - 51 1 R.STGRFNGHFK.T
1434.7136 1433.7063 1433.7184 -8.42 62 - 74 1 R.MGESDDSILRLAK.A
1584.8186 1583.8113 1583.7362 47.4 28 - 41 0 K.DHASIQMNVAEVDR.S
1638.8127 1637.8054 1637.8049 0.30 2 - 15 0 M.QNDAGEFVDLYVLR.K
1766.8878 1765.8805 1765.8999 -10.96 2 - 16 1 M.QNDAGEFVDLYVLRK.C
No match to: 855.0688, 971.4968, 980.5079, 986.4511, 1028.5073, 1082.5900, 1088.5948, 1109.5083, 1110.5568,
1207.6119,
1232.5300, 1234.6511, 1294.6444, 1296.6675, 1300.6644, 1310.6544, 1314.6636, 1318.7582, 1320.5607,
1332.6127, 1333.6133,
1345.6831, 1361.6106, 1377.6110, 1419.7086, 1431.7261, 1436.7306, 1452.6949, 1453.7384, 1473.7191,
1475.7252, 1486.8057,
1489.7124, 1516.7328, 1528.7238, 1545.8552, 1554.7094, 1558.7601, 1570.7126, 1607.7816, 1658.8298,
1675.8585, 1686.7861,
1734.7813, 1750.7714, 1774.8828, 1835.8412, 1837.8254, 1871.9175, 1880.8855, 1976.9685, 1993.9484,
2129.0681, 2177.1699,
2195.1038, 2225.1597, 2605.5559, 2612.2476, 3153.6775
3. gi|281354139 Mass: 123222 Score: 58 Expect: 1.6 Matches: 17
hypothetical protein PANDA_017349 [Ailuropoda melanoleuca]
Observed Mr(expt) Mr(calc) ppm Start End Miss Peptide
986.4511 985.4438 985.4253 18.8 101 - 108 0 K.ENYAGNYR.C
1150.5973 1149.5900 1149.5448 39.3 529 - 539 1 R.ADKAIMEGSGR.I + Oxidation (M)
1294.6444 1293.6371 1293.6751 -29.34 650 - 660 1 K.KMIEGVAYEVR.I
1296.6675 1295.6602 1295.6833 -17.84 344 - 355 1 K.NGEEIIPGPKSR.Y
1300.6644 1299.6571 1299.6935 -28.00 76 - 85 1 K.HLQLKETFER.H
1310.6544 1309.6471 1309.6700 -17.47 650 - 660 1 K.KMIEGVAYEVR.I + Oxidation (M)
1318.7582 1317.7509 1317.7656 -11.16 38 - 49 1 K.VGENITFIAKVK.A
1431.7261 1430.7188 1430.7518 -23.03 224 - 236 1 K.ILDPAYQVDKGGR.V
1436.7306 1435.7233 1435.7671 -30.47 838 - 849 1 R.NSETDTIIFIRK.A
1486.8057 1485.7984 1485.7537 30.1 89 - 100 0 R.VYTFEMQIIQAK.E + Oxidation (M)
1545.8552 1544.8479 1544.8926 -28.91 510 - 523 1 K.LRLEIPISGEPPPK.A
1554.7094 1553.7021 1553.7330 -19.89 1044 - 1056 0 R.MFSNQGVCTLEIR.K
1570.7126 1569.7053 1569.7280 -14.41 1044 - 1056 0 R.MFSNQGVCTLEIR.K + Oxidation (M)
1734.7813 1733.7740 1733.7984 -14.06 924 - 936 0 K.SMEWFTVIEHYHR.T
1750.7714 1749.7641 1749.7933 -16.69 924 - 936 0 K.SMEWFTVIEHYHR.T + Oxidation (M)
1837.8254 1836.8181 1836.8378 -10.69 145 - 162 0 R.SGEGQDDAGELDFSGLLK.R
1993.9484 1992.9411 1992.9389 1.14 145 - 163 1 R.SGEGQDDAGELDFSGLLKR.R

No match to: 855.0688, 971.4968, 980.5079, 1001.5549, 1028.5073, 1082.5900, 1088.5948, 1109.5083,
1110.5568, 1207.6119,
1232.5300, 1234.6511, 1314.6636, 1320.5607, 1332.6127, 1333.6133, 1345.6831, 1361.6106, 1377.6110,
1419.7086, 1434.7136,
1452.6949, 1453.7384, 1473.7191, 1475.7252, 1489.7124, 1516.7328, 1528.7238, 1558.7601, 1584.8186,
1607.7816, 1638.8127,
1658.8298, 1675.8585, 1686.7861, 1766.8878, 1774.8828, 1835.8412, 1871.9175, 1880.8855, 1976.9685,
2129.0681, 2177.1699,
2195.1038, 2225.1597, 2605.5559, 2612.2476, 3153.6775
4. gi|355564610 Mass: 132769 Score: 58 Expect: 1.7 Matches: 18
hypothetical protein EGK_04106 [Macaca mulatta]
Observed Mr(expt) Mr(calc) ppm Start End Miss Peptide
986.4511 985.4438 985.4253 18.8 134 - 141 0 K.ENYAGNYR.C
1028.5073 1027.5000 1027.5298 -28.96 812 - 822 0 K.AINAAGASEPK.Y
1150.5973 1149.5900 1149.5448 39.3 562 - 572 1 R.ADKAIMEGSGR.I + Oxidation (M)
1294.6444 1293.6371 1293.6751 -29.34 683 - 693 1 K.KMIEGVAYEVR.I
1296.6675 1295.6602 1295.6833 -17.84 377 - 388 1 K.NGEEIIPGPKSR.Y
1300.6644 1299.6571 1299.6935 -28.00 109 - 118 1 K.HLQLKETFER.H
1310.6544 1309.6471 1309.6700 -17.47 683 - 693 1 K.KMIEGVAYEVR.I + Oxidation (M)
1318.7582 1317.7509 1317.7656 -11.16 71 - 82 1 K.VGENITFIAKVK.A
1431.7261 1430.7188 1430.7518 -23.03 257 - 269 1 K.ILDPAYQVDKGGR.V
1436.7306 1435.7233 1435.7671 -30.47 889 - 900 1 R.NSETDTIIFIRK.A
1486.8057 1485.7984 1485.7901 5.59 122 - 133 1 R.VYTFEMQIIKAK.E + Oxidation (M)
1545.8552 1544.8479 1544.8926 -28.91 543 - 556 1 K.LRLEIPISGEPPPK.A
1554.7094 1553.7021 1553.7330 -19.89 1095 - 1107 0 R.MFSNQGVCTLEIR.K
1570.7126 1569.7053 1569.7280 -14.41 1095 - 1107 0 R.MFSNQGVCTLEIR.K + Oxidation (M)
1734.7813 1733.7740 1733.7984 -14.06 975 - 987 0 K.SMEWFTVIEHYHR.T
1750.7714 1749.7641 1749.7933 -16.69 975 - 987 0 K.SMEWFTVIEHYHR.T + Oxidation (M)
1837.8254 1836.8181 1836.8378 -10.69 178 - 195 0 R.SGEGQDDAGELDFSGLLK.R
1993.9484 1992.9411 1992.9389 1.14 178 - 196 1 R.SGEGQDDAGELDFSGLLKR.R
No match to: 855.0688, 971.4968, 980.5079, 1001.5549, 1082.5900, 1088.5948, 1109.5083, 1110.5568,
1207.6119, 1232.5300,
1234.6511, 1314.6636, 1320.5607, 1332.6127, 1333.6133, 1345.6831, 1361.6106, 1377.6110, 1419.7086,
1434.7136, 1452.6949,
1453.7384, 1473.7191, 1475.7252, 1489.7124, 1516.7328, 1528.7238, 1558.7601, 1584.8186, 1607.7816,
1638.8127, 1658.8298,
1675.8585, 1686.7861, 1766.8878, 1774.8828, 1835.8412, 1871.9175, 1880.8855, 1976.9685, 2129.0681,
2177.1699, 2195.1038,
2225.1597, 2605.5559, 2612.2476, 3153.6775
5. gi|355786447 Mass: 132797 Score: 58 Expect: 1.7 Matches: 18
hypothetical protein EGM_03664 [Macaca fascicularis]
Observed Mr(expt) Mr(calc) ppm Start End Miss Peptide
986.4511 985.4438 985.4253 18.8 134 - 141 0 K.ENYAGNYR.C
1028.5073 1027.5000 1027.5298 -28.96 812 - 822 0 K.AINAAGASEPK.Y
1150.5973 1149.5900 1149.5448 39.3 562 - 572 1 R.ADKAIMEGSGR.I + Oxidation (M)
1294.6444 1293.6371 1293.6751 -29.34 683 - 693 1 K.KMIEGVAYEVR.I
1296.6675 1295.6602 1295.6833 -17.84 377 - 388 1 K.NGEEIIPGPKSR.Y
1300.6644 1299.6571 1299.6935 -28.00 109 - 118 1 K.HLQLKETFER.H
1310.6544 1309.6471 1309.6700 -17.47 683 - 693 1 K.KMIEGVAYEVR.I + Oxidation (M)
1318.7582 1317.7509 1317.7656 -11.16 71 - 82 1 K.VGENITFIAKVK.A
1431.7261 1430.7188 1430.7518 -23.03 257 - 269 1 K.ILDPAYQVDKGGR.V
1436.7306 1435.7233 1435.7671 -30.47 889 - 900 1 R.NSETDTIIFIRK.A
1486.8057 1485.7984 1485.7901 5.59 122 - 133 1 R.VYTFEMQIIKAK.E + Oxidation (M)
1545.8552 1544.8479 1544.8926 -28.91 543 - 556 1 K.LRLEIPISGEPPPK.A
1554.7094 1553.7021 1553.7330 -19.89 1095 - 1107 0 R.MFSNQGVCTLEIR.K
1570.7126 1569.7053 1569.7280 -14.41 1095 - 1107 0 R.MFSNQGVCTLEIR.K + Oxidation (M)
1734.7813 1733.7740 1733.7984 -14.06 975 - 987 0 K.SMEWFTVIEHYHR.T
1750.7714 1749.7641 1749.7933 -16.69 975 - 987 0 K.SMEWFTVIEHYHR.T + Oxidation (M)
1837.8254 1836.8181 1836.8378 -10.69 178 - 195 0 R.SGEGQDDAGELDFSGLLK.R
1993.9484 1992.9411 1992.9389 1.14 178 - 196 1 R.SGEGQDDAGELDFSGLLKR.R
No match to: 855.0688, 971.4968, 980.5079, 1001.5549, 1082.5900, 1088.5948, 1109.5083, 1110.5568,
1207.6119, 1232.5300,
1234.6511, 1314.6636, 1320.5607, 1332.6127, 1333.6133, 1345.6831, 1361.6106, 1377.6110, 1419.7086,
1434.7136, 1452.6949,
1453.7384, 1473.7191, 1475.7252, 1489.7124, 1516.7328, 1528.7238, 1558.7601, 1584.8186, 1607.7816,
1638.8127, 1658.8298,
1675.8585, 1686.7861, 1766.8878, 1774.8828, 1835.8412, 1871.9175, 1880.8855, 1976.9685, 2129.0681,
2177.1699, 2195.1038,
2225.1597, 2605.5559, 2612.2476, 3153.6775
6. gi|109098449 Mass: 132640 Score: 58 Expect: 1.8 Matches: 18
PREDICTED: myosin-binding protein C, slow-type-like [Macaca mulatta]
Observed Mr(expt) Mr(calc) ppm Start End Miss Peptide
986.4511 985.4438 985.4253 18.8 159 - 166 0 K.ENYAGNYR.C
1028.5073 1027.5000 1027.5298 -28.96 819 - 829 0 K.AINAAGASEPK.Y

1150.5973 1149.5900 1149.5448 39.3 587 - 597 1 R.ADKAIMEGSGR.I + Oxidation (M)
1294.6444 1293.6371 1293.6751 -29.34 708 - 718 1 K.KMIEGVAYEVR.I
1296.6675 1295.6602 1295.6833 -17.84 402 - 413 1 K.NGEEIIPGPKSR.Y
1300.6644 1299.6571 1299.6935 -28.00 134 - 143 1 K.HLQLKETFER.H
1310.6544 1309.6471 1309.6700 -17.47 708 - 718 1 K.KMIEGVAYEVR.I + Oxidation (M)
1318.7582 1317.7509 1317.7656 -11.16 96 - 107 1 K.VGENITFIAKVK.A
1431.7261 1430.7188 1430.7518 -23.03 282 - 294 1 K.ILDPAYQVDKGGR.V
1436.7306 1435.7233 1435.7671 -30.47 896 - 907 1 R.NSETDTIIFIRK.A
1486.8057 1485.7984 1485.7901 5.59 147 - 158 1 R.VYTFEMQIIKAK.E + Oxidation (M)
1545.8552 1544.8479 1544.8926 -28.91 568 - 581 1 K.LRLEIPISGEPPPK.A
1554.7094 1553.7021 1553.7330 -19.89 1102 - 1114 0 R.MFSNQGVCTLEIR.K
1570.7126 1569.7053 1569.7280 -14.41 1102 - 1114 0 R.MFSNQGVCTLEIR.K + Oxidation (M)
1734.7813 1733.7740 1733.7984 -14.06 982 - 994 0 K.SMEWFTVIEHYHR.T
1750.7714 1749.7641 1749.7933 -16.69 982 - 994 0 K.SMEWFTVIEHYHR.T + Oxidation (M)
1837.8254 1836.8181 1836.8378 -10.69 203 - 220 0 R.SGEGQDDAGELDFSGLLK.R
1993.9484 1992.9411 1992.9389 1.14 203 - 221 1 R.SGEGQDDAGELDFSGLLKR.R
No match to: 855.0688, 971.4968, 980.5079, 1001.5549, 1082.5900, 1088.5948, 1109.5083, 1110.5568,
1207.6119, 1232.5300,
1234.6511, 1314.6636, 1320.5607, 1332.6127, 1333.6133, 1345.6831, 1361.6106, 1377.6110, 1419.7086,
1434.7136, 1452.6949,
1453.7384, 1473.7191, 1475.7252, 1489.7124, 1516.7328, 1528.7238, 1558.7601, 1584.8186, 1607.7816,
1638.8127, 1658.8298,
1675.8585, 1686.7861, 1766.8878, 1774.8828, 1835.8412, 1871.9175, 1880.8855, 1976.9685, 2129.0681,
2177.1699, 2195.1038,
2225.1597, 2605.5559, 2612.2476, 3153.6775
7. gi|2735295 Mass: 5517 Score: 57 Expect: 1.9 Matches: 5
deoxyuridine triphosphate nucleotidohydrolase, nuclear isoform [Homo sapiens]
Observed Mr(expt) Mr(calc) ppm Start End Miss Peptide
1082.5900 1081.5827 1081.5516 28.8 31 - 40 0 R.LSEHATAPTR.G
1300.6644 1299.6571 1299.6717 -11.24 16 - 27 0 R.ARPAEVGGMQLR.F + Oxidation (M)
1453.7384 1452.7311 1452.7433 -8.39 31 - 44 1 R.LSEHATAPTRGSAR.A
1558.7601 1557.7528 1557.7457 4.58 2 - 15 1 M.PCSEETPAISPSKR.A
1658.8298 1657.8225 1657.8835 -36.75 16 - 30 1 R.ARPAEVGGMQLRFAR.L
No match to: 855.0688, 971.4968, 980.5079, 986.4511, 1001.5549, 1028.5073, 1088.5948, 1109.5083, 1110.5568,
1150.5973,
1207.6119, 1232.5300, 1234.6511, 1294.6444, 1296.6675, 1310.6544, 1314.6636, 1318.7582, 1320.5607,
1332.6127, 1333.6133,
1345.6831, 1361.6106, 1377.6110, 1419.7086, 1431.7261, 1434.7136, 1436.7306, 1452.6949, 1473.7191,
1475.7252, 1486.8057,
1489.7124, 1516.7328, 1528.7238, 1545.8552, 1554.7094, 1570.7126, 1584.8186, 1607.7816, 1638.8127,
1675.8585, 1686.7861,
1734.7813, 1750.7714, 1766.8878, 1774.8828, 1835.8412, 1837.8254, 1871.9175, 1880.8855, 1976.9685,
1993.9484, 2129.0681,
2177.1699, 2195.1038, 2225.1597, 2605.5559, 2612.2476, 3153.6775
8. gi|301784296 Mass: 133817 Score: 56 Expect: 2.6 Matches: 18
PREDICTED: myosin-binding protein C, slow-type-like [Ailuropoda melanoleuca]
Observed Mr(expt) Mr(calc) ppm Start End Miss Peptide
986.4511 985.4438 985.4253 18.8 163 - 170 0 K.ENYAGNYR.C
1150.5973 1149.5900 1149.5448 39.3 591 - 601 1 R.ADKAIMEGSGR.I + Oxidation (M)
1294.6444 1293.6371 1293.6751 -29.34 712 - 722 1 K.KMIEGVAYEVR.I
1296.6675 1295.6602 1295.6833 -17.84 406 - 417 1 K.NGEEIIPGPKSR.Y
1300.6644 1299.6571 1299.6935 -28.00 138 - 147 1 K.HLQLKETFER.H
1310.6544 1309.6471 1309.6700 -17.47 712 - 722 1 K.KMIEGVAYEVR.I + Oxidation (M)
1318.7582 1317.7509 1317.7656 -11.16 100 - 111 1 K.VGENITFIAKVK.A
1332.6127 1331.6054 1331.6478 -31.84 16 - 25 1 R.NCQLQKMPWK.L
1431.7261 1430.7188 1430.7518 -23.03 286 - 298 1 K.ILDPAYQVDKGGR.V
1436.7306 1435.7233 1435.7644 -28.62 2 - 15 1 M.QTVLRGVGEAGHGR.N
1486.8057 1485.7984 1485.7537 30.1 151 - 162 0 R.VYTFEMQIIQAK.E + Oxidation (M)
1545.8552 1544.8479 1544.8926 -28.91 572 - 585 1 K.LRLEIPISGEPPPK.A
1554.7094 1553.7021 1553.7330 -19.89 1106 - 1118 0 R.MFSNQGVCTLEIR.K
1570.7126 1569.7053 1569.7280 -14.41 1106 - 1118 0 R.MFSNQGVCTLEIR.K + Oxidation (M)
1734.7813 1733.7740 1733.7984 -14.06 986 - 998 0 K.SMEWFTVIEHYHR.T
1750.7714 1749.7641 1749.7933 -16.69 986 - 998 0 K.SMEWFTVIEHYHR.T + Oxidation (M)
1837.8254 1836.8181 1836.8378 -10.69 207 - 224 0 R.SGEGQDDAGELDFSGLLK.R
1993.9484 1992.9411 1992.9389 1.14 207 - 225 1 R.SGEGQDDAGELDFSGLLKR.R
No match to: 855.0688, 971.4968, 980.5079, 1001.5549, 1028.5073, 1082.5900, 1088.5948, 1109.5083,
1110.5568, 1207.6119,
1232.5300, 1234.6511, 1314.6636, 1320.5607, 1333.6133, 1345.6831, 1361.6106, 1377.6110, 1419.7086,
1434.7136, 1452.6949,
1453.7384, 1473.7191, 1475.7252, 1489.7124, 1516.7328, 1528.7238, 1558.7601, 1584.8186, 1607.7816,
1638.8127, 1658.8298,
1675.8585, 1686.7861, 1766.8878, 1774.8828, 1835.8412, 1871.9175, 1880.8855, 1976.9685, 2129.0681,
2177.1699, 2195.1038,
2225.1597, 2605.5559, 2612.2476, 3153.6775

9. gi|351695270 Mass: 132081 Score: 56 Expect: 2.7 Matches: 15
Zinc finger protein 91, partial [Heterocephalus glaber]
Observed Mr(expt) Mr(calc) ppm Start End Miss Peptide
980.5079 979.5006 979.4909 9.91 760 - 767 1 K.ECGKAFIR.S
986.4511 985.4438 985.3997 44.7 588 - 594 1 R.FECKDCK.K
1310.6544 1309.6471 1309.6271 15.3 614 - 623 1 K.KLFICQECGR.A
1361.6106 1360.6033 1360.6445 -30.27 1001 - 1011 0 R.LHTGEKPYECK.E
1431.7261 1430.7188 1430.7531 -23.97 207 - 218 0 K.FSSGHQLILHHR.F
1545.8552 1544.8479 1544.8059 27.2 624 - 636 1 R.AYTTRSNLVQHQK.T
1558.7601 1557.7528 1557.7834 -19.64 816 - 828 1 K.ECGKAFSRPTHLR.L
1570.7126 1569.7053 1569.7800 -47.60 652 - 664 0 K.AFSLHGYLNQHQR.I
1584.8186 1583.8113 1583.8168 -3.48 377 - 390 0 R.DHQLIAHQSIHTGK.K
1607.7816 1606.7743 1606.7634 6.79 439 - 451 1 R.HQNLHTGDKLECR.Q
1638.8127 1637.8054 1637.8096 -2.57 956 - 969 1 K.ECGKAFSHPSHLIR.H
1766.8878 1765.8805 1765.8747 3.27 792 - 806 1 K.TFRYDSSLTLHQGNK.Y
1774.8828 1773.8755 1773.8291 26.2 615 - 628 1 K.LFICQECGRAYTTR.S
1835.8412 1834.8339 1834.8342 -0.14 1001 - 1015 1 R.LHTGEKPYECKECGK.A
2195.1038 2194.0965 2194.1469 -22.98 568 - 586 1 K.AFSLHGYLSQHQKIHLGMK.R
No match to: 855.0688, 971.4968, 1001.5549, 1028.5073, 1082.5900, 1088.5948, 1109.5083, 1110.5568,
1150.5973, 1207.6119,
1232.5300, 1234.6511, 1294.6444, 1296.6675, 1300.6644, 1314.6636, 1318.7582, 1320.5607, 1332.6127,
1333.6133, 1345.6831,
1377.6110, 1419.7086, 1434.7136, 1436.7306, 1452.6949, 1453.7384, 1473.7191, 1475.7252, 1486.8057,
1489.7124, 1516.7328,
1528.7238, 1554.7094, 1658.8298, 1675.8585, 1686.7861, 1734.7813, 1750.7714, 1837.8254, 1871.9175,
1880.8855, 1976.9685,
1993.9484, 2129.0681, 2177.1699, 2225.1597, 2605.5559, 2612.2476, 3153.6775
10. gi|26334151 Mass: 78962 Score: 55 Expect: 3.1 Matches: 11
unnamed protein product [Mus musculus]
Observed Mr(expt) Mr(calc) ppm Start End Miss Peptide
1001.5549 1000.5476 1000.5553 -7.65 621 - 628 1 K.VERELVEK.E
1110.5568 1109.5495 1109.5829 -30.08 589 - 597 1 R.LVKNHEQDK.E
1296.6675 1295.6602 1295.6115 37.6 313 - 322 0 K.IMHTQHCEIK.E
1320.5607 1319.5534 1319.5850 -23.90 376 - 386 0 K.VMTDHNMSLEK.L + Oxidation (M)
1434.7136 1433.7063 1433.6885 12.4 652 - 664 0 R.EDSSSLVAELQEK.L
1436.7306 1435.7233 1435.7406 -12.04 29 - 42 0 R.IESTTGITTTTSPK.T
1473.7191 1472.7118 1472.7219 -6.86 14 - 28 0 K.VSTSQASPQSAASPR.I
1486.8057 1485.7984 1485.7674 20.9 270 - 282 0 K.ENLVSLEEALQNK.D
1516.7328 1515.7255 1515.7140 7.61 348 - 359 0 R.AEFQCLEQNHLK.E
1686.7861 1685.7788 1685.7064 43.0 150 - 164 1 R.MQDTSTRGNEGFGDR.A + Oxidation (M)
1774.8828 1773.8755 1773.8996 -13.55 438 - 452 1 K.EAETDEIKILLEESR.T
No match to: 855.0688, 971.4968, 980.5079, 986.4511, 1028.5073, 1082.5900, 1088.5948, 1109.5083, 1150.5973,
1207.6119,
1232.5300, 1234.6511, 1294.6444, 1300.6644, 1310.6544, 1314.6636, 1318.7582, 1332.6127, 1333.6133,
1345.6831, 1361.6106,
1377.6110, 1419.7086, 1431.7261, 1452.6949, 1453.7384, 1475.7252, 1489.7124, 1528.7238, 1545.8552,
1554.7094, 1558.7601,
1570.7126, 1584.8186, 1607.7816, 1638.8127, 1658.8298, 1675.8585, 1734.7813, 1750.7714, 1766.8878,
1835.8412, 1837.8254,
1871.9175, 1880.8855, 1976.9685, 1993.9484, 2129.0681, 2177.1699, 2195.1038, 2225.1597, 2605.5559,
2612.2476, 3153.6775
11. gi|344267648 Mass: 134226 Score: 55 Expect: 3.7 Matches: 18
PREDICTED: myosin-binding protein C, slow-type [Loxodonta africana]
Observed Mr(expt) Mr(calc) ppm Start End Miss Peptide
986.4511 985.4438 985.4253 18.8 171 - 178 0 K.ENYAGNYR.C
1028.5073 1027.5000 1027.5298 -28.96 831 - 841 0 K.AINAAGASEPK.Y
1150.5973 1149.5900 1149.5448 39.3 599 - 609 1 R.ADKAIMEGSGR.I + Oxidation (M)
1294.6444 1293.6371 1293.6751 -29.34 720 - 730 1 K.KMIEGVAYEVR.I
1296.6675 1295.6602 1295.6874 -20.96 262 - 272 0 K.IAFQYGITDLR.G
1300.6644 1299.6571 1299.6935 -28.00 146 - 155 1 K.HLQLKETFER.H
1310.6544 1309.6471 1309.6700 -17.47 720 - 730 1 K.KMIEGVAYEVR.I + Oxidation (M)
1431.7261 1430.7188 1430.7518 -23.03 294 - 306 1 R.ILDPAYQVDKGGR.V
1436.7306 1435.7233 1435.7671 -30.47 908 - 919 1 R.NSETDTIIFIRK.A
1486.8057 1485.7984 1485.7537 30.1 159 - 170 0 R.VYTFEMQIIQAK.E + Oxidation (M)
1545.8552 1544.8479 1544.8926 -28.91 580 - 593 1 K.LRLEIPISGEPPPK.A
1554.7094 1553.7021 1553.7330 -19.89 1114 - 1126 0 R.MFSNQGVCTLEIR.K
1570.7126 1569.7053 1569.7280 -14.41 1114 - 1126 0 R.MFSNQGVCTLEIR.K + Oxidation (M)
1734.7813 1733.7740 1733.7984 -14.06 994 - 1006 0 K.SMEWFTVIEHYHR.T
1750.7714 1749.7641 1749.7933 -16.69 994 - 1006 0 K.SMEWFTVIEHYHR.T + Oxidation (M)
1837.8254 1836.8181 1836.8378 -10.69 215 - 232 0 R.SGEGQDDAGELDFSGLLK.R
1976.9685 1975.9612 1975.9891 -14.10 1007 - 1023 0 R.TSATITELVIGNEYYFR.V
1993.9484 1992.9411 1992.9389 1.14 215 - 233 1 R.SGEGQDDAGELDFSGLLKR.R
No match to: 855.0688, 971.4968, 980.5079, 1001.5549, 1082.5900, 1088.5948, 1109.5083, 1110.5568,
1207.6119, 1232.5300,

1234.6511, 1314.6636, 1318.7582, 1320.5607, 1332.6127, 1333.6133, 1345.6831, 1361.6106, 1377.6110, 1419.7086, 1434.7136,
1452.6949, 1453.7384, 1473.7191, 1475.7252, 1489.7124, 1516.7328, 1528.7238, 1558.7601, 1584.8186, 1607.7816, 1638.8127,
1658.8298, 1675.8585, 1686.7861, 1766.8878, 1774.8828, 1835.8412, 1871.9175, 1880.8855, 2129.0681, 2177.1699, 2195.1038,
2225.1597, 2605.5559, 2612.2476, 3153.6775
12. gi|115496194 Mass: 34281 Score: 54 Expect: 4 Matches: 8
complement C1q and tumor necrosis factor-related protein 9 precursor [Bos taurus]
Observed Mr(expt) Mr(calc) ppm Start End Miss Peptide
971.4968 970.4895 970.5157 -27.00 193 - 201 0 K.IGEMPALPK.S + Oxidation (M)
1001.5549 1000.5476 1000.5229 24.7 214 - 222 0 K.FPPSDTPIK.F
1232.5300 1231.5227 1231.5582 -28.79 53 - 66 0 K.GDAGEPGHPGGPGK.D
1294.6444 1293.6371 1293.6313 4.49 73 - 85 1 K.KGEPGADGHVEAK.G
1419.7086 1418.7013 1418.7194 -12.76 214 - 225 1 K.FPPSDTPIKFDR.I
1486.8057 1485.7984 1485.7423 37.7 113 - 127 1 K.GLTGETGPQGQKGEK.G
1558.7601 1557.7528 1557.7787 -16.63 297 - 310 0 K.LGDEVWLQVTGGER.F
1837.8254 1836.8181 1836.8061 6.54 53 - 72 1 K.GDAGEPGHPGGPGKDGMTGK.K + Oxidation (M)
No match to: 855.0688, 980.5079, 986.4511, 1028.5073, 1082.5900, 1088.5948, 1109.5083, 1110.5568, 1150.5973, 1207.6119,
1234.6511, 1296.6675, 1300.6644, 1310.6544, 1314.6636, 1318.7582, 1320.5607, 1332.6127, 1333.6133, 1345.6831, 1361.6106,
1377.6110, 1431.7261, 1434.7136, 1436.7306, 1452.6949, 1453.7384, 1473.7191, 1475.7252, 1489.7124, 1516.7328, 1528.7238,
1545.8552, 1554.7094, 1570.7126, 1584.8186, 1607.7816, 1638.8127, 1658.8298, 1675.8585, 1686.7861, 1734.7813, 1750.7714,
1766.8878, 1774.8828, 1835.8412, 1871.9175, 1880.8855, 1976.9685, 1993.9484, 2129.0681, 2177.1699, 2195.1038, 2225.1597,
2605.5559, 2612.2476, 3153.6775
13. gi|332257525 Mass: 96495 Score: 54 Expect: 4.1 Matches: 12
PREDICTED: FYVE, RhoGEF and PH domain-containing protein 4 isoform 3 [Nomascus leucogenys]
Observed Mr(expt) Mr(calc) ppm Start End Miss Peptide
980.5079 979.5006 979.4611 40.4 683 - 691 0 K.AQLEYDGGK.L
1001.5549 1000.5476 1000.4978 49.8 787 - 795 0 K.SADLPHSFK.L
1110.5568 1109.5495 1109.5829 -30.11 141 - 150 0 R.HGLTTTPQQK.L
1333.6133 1332.6060 1332.6245 -13.83 360 - 369 1 K.RMQEWETTPR.I
1419.7086 1418.7013 1418.6678 23.7 801 - 813 0 K.SVHSFAADSEELK.Q
1453.7384 1452.7311 1452.7685 -25.70 127 - 140 1 K.ESAVNLNAPKTPGR.H
1558.7601 1557.7528 1557.8151 -39.97 787 - 800 1 K.SADLPHSFKLTQSK.S
1658.8298 1657.8225 1657.7947 16.8 112 - 126 1 R.FEGGSSLSNYNDLKK.E
1675.8585 1674.8512 1674.8213 17.9 801 - 815 1 K.SVHSFAADSEELKQK.W
1880.8855 1879.8782 1879.9138 -18.94 754 - 769 0 K.QDPLVLYMYGAPQDVR.A + Oxidation (M)
1976.9685 1975.9612 1975.9851 -12.06 796 - 813 1 K.LTQSKSVHSFAADSEELK.Q
1993.9484 1992.9411 1992.9325 4.33 383 - 399 1 K.MYGEYVKGFDNAMELVK.N
No match to: 855.0688, 971.4968, 986.4511, 1028.5073, 1082.5900, 1088.5948, 1109.5083, 1150.5973, 1207.6119, 1232.5300,
1234.6511, 1294.6444, 1296.6675, 1300.6644, 1310.6544, 1314.6636, 1318.7582, 1320.5607, 1332.6127, 1345.6831, 1361.6106,
1377.6110, 1431.7261, 1434.7136, 1436.7306, 1452.6949, 1473.7191, 1475.7252, 1486.8057, 1489.7124, 1516.7328, 1528.7238,
1545.8552, 1554.7094, 1570.7126, 1584.8186, 1607.7816, 1638.8127, 1686.7861, 1734.7813, 1750.7714, 1766.8878, 1774.8828,
1835.8412, 1837.8254, 1871.9175, 2129.0681, 2177.1699, 2195.1038, 2225.1597, 2605.5559, 2612.2476, 3153.6775
14. gi|120952829 Mass: 55358 Score: 54 Expect: 4.1 Matches: 10
zinc finger protein 331 [Homo sapiens]
Observed Mr(expt) Mr(calc) ppm Start End Miss Peptide
1088.5948 1087.5875 1087.5410 42.7 143 - 151 0 R.GYQLSQHQK.I
1150.5973 1149.5900 1149.6295 -34.31 335 - 344 1 K.AFRWGSSLVK.H
1333.6133 1332.6060 1332.6496 -32.71 376 - 386 1 R.IHTGETPYKCK.E
1361.6106 1360.6033 1360.6445 -30.27 208 - 218 0 R.IHTGEKPYECK.D
1436.7306 1435.7233 1435.7783 -38.28 55 - 66 1 K.SLPTEKNIHEIR.A
1516.7328 1515.7255 1515.7980 -47.82 279 - 291 1 K.AFICGSSLIQHKR.I
1558.7601 1557.7528 1557.7861 -21.36 387 - 400 1 K.ECGKAFIYGSSLVK.H
1658.8298 1657.8225 1657.7995 13.9 82 - 94 0 R.NWICEGTLERPQR.S
1835.8412 1834.8339 1834.7978 19.7 292 - 306 0 R.IHTGEKPYECQECGK.A
2129.0681 2128.0608 2127.9942 31.3 401 - 418 1 K.HERIHTGVKPYGCTECGK.S
No match to: 855.0688, 971.4968, 980.5079, 986.4511, 1001.5549, 1028.5073, 1082.5900, 1109.5083, 1110.5568, 1207.6119,
1232.5300, 1234.6511, 1294.6444, 1296.6675, 1300.6644, 1310.6544, 1314.6636, 1318.7582, 1320.5607, 1332.6127, 1345.6831,
1377.6110, 1419.7086, 1431.7261, 1434.7136, 1452.6949, 1453.7384, 1473.7191, 1475.7252, 1486.8057, 1489.7124, 1528.7238,

1545.8552, 1554.7094, 1570.7126, 1584.8186, 1607.7816, 1638.8127, 1675.8585, 1686.7861, 1734.7813,
1750.7714, 1766.8878,
1774.8828, 1837.8254, 1871.9175, 1880.8855, 1976.9685, 1993.9484, 2177.1699, 2195.1038, 2225.1597,
2605.5559, 2612.2476,
3153.6775
15. gi|332221246 Mass: 55427 Score: 54 Expect: 4.1 Matches: 10
PREDICTED: zinc finger protein 331 isoform 1 [Nomascus leucogenys]
Observed Mr(expt) Mr(calc) ppm Start End Miss Peptide
1088.5948 1087.5875 1087.5410 42.7 143 - 151 0 R.GYQLSQHQK.I
1150.5973 1149.5900 1149.6295 -34.31 335 - 344 1 K.AFRWGSSLVK.H
1333.6133 1332.6060 1332.6496 -32.71 376 - 386 1 R.IHTGETPYKCK.E
1361.6106 1360.6033 1360.6445 -30.27 208 - 218 0 R.IHTGEKPYECK.D
1436.7306 1435.7233 1435.7783 -38.28 55 - 66 1 K.SLPTEKNIHEIR.A
1516.7328 1515.7255 1515.7980 -47.82 279 - 291 1 K.AFICGSSLIQHKR.I
1558.7601 1557.7528 1557.7861 -21.36 387 - 400 1 K.ECGKAFIYGSSLVK.H
1658.8298 1657.8225 1657.7995 13.9 82 - 94 0 R.NWICEGTLERPQR.S
1835.8412 1834.8339 1834.7978 19.7 292 - 306 0 R.IHTGEKPYECQECGK.A
2129.0681 2128.0608 2127.9942 31.3 401 - 418 1 K.HERIHTGVKPYGCTECGK.S
No match to: 855.0688, 971.4968, 980.5079, 986.4511, 1001.5549, 1028.5073, 1082.5900, 1109.5083, 1110.5568,
1207.6119,
1232.5300, 1234.6511, 1294.6444, 1296.6675, 1300.6644, 1310.6544, 1314.6636, 1318.7582, 1320.5607,
1332.6127, 1345.6831,
1377.6110, 1419.7086, 1431.7261, 1434.7136, 1452.6949, 1453.7384, 1473.7191, 1475.7252, 1486.8057,
1489.7124, 1528.7238,
1545.8552, 1554.7094, 1570.7126, 1584.8186, 1607.7816, 1638.8127, 1675.8585, 1686.7861, 1734.7813,
1750.7714, 1766.8878,
1774.8828, 1837.8254, 1871.9175, 1880.8855, 1976.9685, 1993.9484, 2177.1699, 2195.1038, 2225.1597,
2605.5559, 2612.2476,
3153.6775
16. gi|332857190 Mass: 55461 Score: 54 Expect: 4.1 Matches: 10
PREDICTED: zinc finger protein 331 isoform 1 [Pan troglodytes]
Observed Mr(expt) Mr(calc) ppm Start End Miss Peptide
1088.5948 1087.5875 1087.5410 42.7 143 - 151 0 R.GYQLSQHQK.I
1150.5973 1149.5900 1149.6295 -34.31 335 - 344 1 K.AFRWGSSLVK.H
1333.6133 1332.6060 1332.6496 -32.71 376 - 386 1 R.IHTGETPYKCK.E
1361.6106 1360.6033 1360.6445 -30.27 208 - 218 0 R.IHTGEKPYECK.D
1436.7306 1435.7233 1435.7783 -38.28 55 - 66 1 K.SLPTEKNIHEIR.A
1516.7328 1515.7255 1515.7980 -47.82 279 - 291 1 K.AFICGSSLIQHKR.I
1558.7601 1557.7528 1557.7861 -21.36 387 - 400 1 K.ECGKAFIYGSSLVK.H
1658.8298 1657.8225 1657.7995 13.9 82 - 94 0 R.NWICEGTLERPQR.S
1835.8412 1834.8339 1834.7978 19.7 292 - 306 0 R.IHTGEKPYECQECGK.A
2129.0681 2128.0608 2127.9942 31.3 401 - 418 1 K.HERIHTGVKPYGCTECGK.S
No match to: 855.0688, 971.4968, 980.5079, 986.4511, 1001.5549, 1028.5073, 1082.5900, 1109.5083, 1110.5568,
1207.6119,
1232.5300, 1234.6511, 1294.6444, 1296.6675, 1300.6644, 1310.6544, 1314.6636, 1318.7582, 1320.5607,
1332.6127, 1345.6831,
1377.6110, 1419.7086, 1431.7261, 1434.7136, 1452.6949, 1453.7384, 1473.7191, 1475.7252, 1486.8057,
1489.7124, 1528.7238,
1545.8552, 1554.7094, 1570.7126, 1584.8186, 1607.7816, 1638.8127, 1675.8585, 1686.7861, 1734.7813,
1750.7714, 1766.8878,
1774.8828, 1837.8254, 1871.9175, 1880.8855, 1976.9685, 1993.9484, 2177.1699, 2195.1038, 2225.1597,
2605.5559, 2612.2476,
3153.6775
17. gi|343958786 Mass: 55431 Score: 54 Expect: 4.1 Matches: 10
zinc finger protein 331 [Pan troglodytes]
Observed Mr(expt) Mr(calc) ppm Start End Miss Peptide
1088.5948 1087.5875 1087.5410 42.7 143 - 151 0 R.GYQLSQHQK.I
1150.5973 1149.5900 1149.6295 -34.31 335 - 344 1 K.AFRWGSSLVK.H
1333.6133 1332.6060 1332.6496 -32.71 376 - 386 1 R.IHTGETPYKCK.E
1361.6106 1360.6033 1360.6445 -30.27 208 - 218 0 R.IHTGEKPYECK.D
1436.7306 1435.7233 1435.7783 -38.28 55 - 66 1 K.SLPTEKNIHEIR.A
1516.7328 1515.7255 1515.7980 -47.82 279 - 291 1 K.AFICGSSLIQHKR.I
1558.7601 1557.7528 1557.7861 -21.36 387 - 400 1 K.ECGKAFIYGSSLVK.H
1658.8298 1657.8225 1657.7995 13.9 82 - 94 0 R.NWICEGTLERPQR.S
1835.8412 1834.8339 1834.7978 19.7 292 - 306 0 R.IHTGEKPYECQECGK.A
2129.0681 2128.0608 2127.9942 31.3 401 - 418 1 K.HERIHTGVKPYGCTECGK.S
No match to: 855.0688, 971.4968, 980.5079, 986.4511, 1001.5549, 1028.5073, 1082.5900, 1109.5083, 1110.5568,
1207.6119,
1232.5300, 1234.6511, 1294.6444, 1296.6675, 1300.6644, 1310.6544, 1314.6636, 1318.7582, 1320.5607,
1332.6127, 1345.6831,
1377.6110, 1419.7086, 1431.7261, 1434.7136, 1452.6949, 1453.7384, 1473.7191, 1475.7252, 1486.8057,
1489.7124, 1528.7238,
1545.8552, 1554.7094, 1570.7126, 1584.8186, 1607.7816, 1638.8127, 1675.8585, 1686.7861, 1734.7813,
1750.7714, 1766.8878,

1774.8828, 1837.8254, 1871.9175, 1880.8855, 1976.9685, 1993.9484, 2177.1699, 2195.1038, 2225.1597,
2605.5559, 2612.2476,
3153.6775
18. gi|348562043 Mass: 87299 Score: 54 Expect: 4.1 Matches: 11
PREDICTED: FYVE, RhoGEF and PH domain-containing protein 4-like [Cavia porcellus]
Observed Mr(expt) Mr(calc) ppm Start End Miss Peptide
980.5079 979.5006 979.4611 40.4 604 - 612 0 K.AQLEYDGGK.W
1001.5549 1000.5476 1000.4978 49.8 708 - 716 0 R.SADLPHSFK.L
1333.6133 1332.6060 1332.6245 -13.83 281 - 290 1 K.RMQEWETTPR.I
1419.7086 1418.7013 1418.6678 23.7 722 - 734 0 K.SVHSFAADSEELK.Q
1558.7601 1557.7528 1557.8151 -39.97 708 - 721 1 R.SADLPHSFKLTQSK.S
1675.8585 1674.8512 1674.8213 17.9 722 - 736 1 K.SVHSFAADSEELKQK.W
1871.9175 1870.9102 1870.9975 -46.64 691 - 707 0 R.AQATIPLLGYLVDNMPR.S
1880.8855 1879.8782 1879.9138 -18.94 675 - 690 0 K.QDPLVLYMYGAPQDVR.A + Oxidation (M)
1976.9685 1975.9612 1975.9851 -12.06 717 - 734 1 K.LTQSKSVHSFAADSEELK.Q
1993.9484 1992.9411 1992.9325 4.33 304 - 320 1 K.MYGEYVKGFDNAMELVK.N
2129.0681 2128.0608 2128.0225 18.0 484 - 501 0 K.IIETHNEEYPHTFQVSGK.E
No match to: 855.0688, 971.4968, 986.4511, 1028.5073, 1082.5900, 1088.5948, 1109.5083, 1110.5568,
1150.5973, 1207.6119,
1232.5300, 1234.6511, 1294.6444, 1296.6675, 1300.6644, 1310.6544, 1314.6636, 1318.7582, 1320.5607,
1332.6127, 1345.6831,
1361.6106, 1377.6110, 1431.7261, 1434.7136, 1436.7306, 1452.6949, 1453.7384, 1473.7191, 1475.7252,
1486.8057, 1489.7124,
1516.7328, 1528.7238, 1545.8552, 1554.7094, 1570.7126, 1584.8186, 1607.7816, 1638.8127, 1658.8298,
1686.7861, 1734.7813,
1750.7714, 1766.8878, 1774.8828, 1835.8412, 1837.8254, 2177.1699, 2195.1038, 2225.1597, 2605.5559,
2612.2476, 3153.6775
19. gi|351699086 Mass: 24574 Score: 54 Expect: 4.2 Matches: 8
Putative deoxyribonuclease TATDN1 [Heterocephalus glaber]
Observed Mr(expt) Mr(calc) ppm Start End Miss Peptide
1001.5549 1000.5476 1000.5553 -7.65 43 - 51 0 K.EALQLAQTK.D
1314.6636 1313.6563 1313.5935 47.8 164 - 173 1 K.WENGHCLKDR.N
1436.7306 1435.7233 1435.6766 32.6 91 - 103 0 K.VVAVGECGLDFDR.L
1554.7094 1553.7021 1553.6756 17.1 52 - 64 0 K.DMFFSTVGCHPTR.C
1570.7126 1569.7053 1569.6705 22.2 52 - 64 0 K.DMFFSTVGCHPTR.C + Oxidation (M)
1976.9685 1975.9612 1975.9738 -6.36 191 - 207 0 R.DEEPLELANTLYNNTIK.L
2129.0681 2128.0608 2128.1415 -37.91 71 - 88 1 K.KNPDLYLEELLNLAEINK.G
2177.1699 2176.1626 2176.0544 49.7 127 - 145 1 K.SIPSEKLMIETDAPWCGVK.S + Oxidation (M)
No match to: 855.0688, 971.4968, 980.5079, 986.4511, 1028.5073, 1082.5900, 1088.5948, 1109.5083, 1110.5568,
1150.5973,
1207.6119, 1232.5300, 1234.6511, 1294.6444, 1296.6675, 1300.6644, 1310.6544, 1318.7582, 1320.5607,
1332.6127, 1333.6133,
1345.6831, 1361.6106, 1377.6110, 1419.7086, 1431.7261, 1434.7136, 1452.6949, 1453.7384, 1473.7191,
1475.7252, 1486.8057,
1489.7124, 1516.7328, 1528.7238, 1545.8552, 1558.7601, 1584.8186, 1607.7816, 1638.8127, 1658.8298,
1675.8585, 1686.7861,
1734.7813, 1750.7714, 1766.8878, 1774.8828, 1835.8412, 1837.8254, 1871.9175, 1880.8855, 1993.9484,
2195.1038, 2225.1597,
2605.5559, 2612.2476, 3153.6775
20. gi|297692730 Mass: 96695 Score: 54 Expect: 4.5 Matches: 13
PREDICTED: myosin-binding protein C, slow-type-like, partial [Pongo abelii]
Observed Mr(expt) Mr(calc) ppm Start End Miss Peptide
980.5079 979.5006 979.4545 47.1 582 - 589 1 K.AMWSRADK.A + Oxidation (M)
1150.5973 1149.5900 1149.5448 39.3 587 - 597 1 R.ADKAIMEGSGR.I + Oxidation (M)
1294.6444 1293.6371 1293.6751 -29.34 708 - 718 1 K.KMIEGVAYEVR.I
1296.6675 1295.6602 1295.6833 -17.84 402 - 413 1 K.NGEEIIPGPKSR.Y
1300.6644 1299.6571 1299.6935 -28.00 134 - 143 1 K.HLQLKETFER.H
1310.6544 1309.6471 1309.6700 -17.47 708 - 718 1 K.KMIEGVAYEVR.I + Oxidation (M)
1431.7261 1430.7188 1430.7518 -23.03 282 - 294 1 K.ILDPAYQVDKGGR.V
1486.8057 1485.7984 1485.7901 5.59 147 - 158 1 R.VYTFEMQIIKAK.E + Oxidation (M)
1545.8552 1544.8479 1544.8926 -28.91 568 - 581 1 K.LRLEIPISGEPPPK.A
1750.7714 1749.7641 1749.7780 -7.96 159 - 172 1 K.ENFAGNYRCEVTYK.D
1837.8254 1836.8181 1836.8378 -10.69 203 - 220 0 R.SGEGQDDAGELDFSGLLK.R
1871.9175 1870.9102 1870.8585 27.7 58 - 74 0 K.DSDWTLVEPPAGEEQAK.Q
1993.9484 1992.9411 1992.9389 1.14 203 - 221 1 R.SGEGQDDAGELDFSGLLKR.R
No match to: 855.0688, 971.4968, 986.4511, 1001.5549, 1028.5073, 1082.5900, 1088.5948, 1109.5083,
1110.5568, 1207.6119,
1232.5300, 1234.6511, 1314.6636, 1318.7582, 1320.5607, 1332.6127, 1333.6133, 1345.6831, 1361.6106,
1377.6110, 1419.7086,
1434.7136, 1436.7306, 1452.6949, 1453.7384, 1473.7191, 1475.7252, 1489.7124, 1516.7328, 1528.7238,
1554.7094, 1558.7601,
1570.7126, 1584.8186, 1607.7816, 1638.8127, 1658.8298, 1675.8585, 1686.7861, 1734.7813, 1766.8878,
1774.8828, 1835.8412,
1880.8855, 1976.9685, 2129.0681, 2177.1699, 2195.1038, 2225.1597, 2605.5559, 2612.2476, 3153.6775

Search Parameters

Type of search : Peptide Mass Fingerprint
Enzyme : Trypsin
Fixed modifications : Carbamidomethyl (C)
Variable modifications : Oxidation (M)
Mass values : Monoisotopic
Protein Mass : Unrestricted
Peptide Mass Tolerance : ± 50 ppm
Peptide Charge State : 1+
Max Missed Cleavages : 1
Number of queries : 65

Mascot: http://www.matrixscience.com

COVERAGE BAND 3

Mascot Search Results

Protein View

Match to: gi|160425243 Score: 111 Expect: 8.4e-006
myosin-binding protein C, slow-type [Bos taurus]
Nominal mass (Mr): 134923; Calculated pI value: 5.60
NCBI BLAST search of gi|160425243 against nr
Unformatted sequence string for pasting into other applications
Taxonomy: Bos taurus
Links to retrieve other entries containing this sequence from NCBI Entrez:
gi|151555754 from Bos taurus
gi|296487652 from Bos taurus
Fixed modifications: Carbamidomethyl (C)
Variable modifications: Oxidation (M)
Cleavage by Trypsin: cuts C-term side of KR unless next residue is P
Number of mass values searched: 65
Number of mass values matched: 23
Sequence Coverage: 20%
Matched peptides shown in **Bold Red**

```
1 MPEPTKKEEN EVLAPAPAPP PEEPNKEKEA GTAPVKEPNK EKEAGTAPAK
51 EPNKEKEAGT APAKEPNKEK EAGTSPGKDE EDASPPGGLP PEWSIGESPA
101 GEEQDKQNAN SQLSILFVEK PQGGTVKVGE NITFIAKVKA EDLLRKPTVK
151 WFKGKWMDLA SKAGKHLQLK ETFERHSRVY TFEMQIIKAK ENYAGNYRCE
201 VTYKDKFDSC SFDLEVLEST GTTPNIDIRS AFKRSGEGQD DAGELDFSGL
251 LKRREVKPQE EQPEVDVWEL LKNAKPSEYE KIAFQYGITD LRGMLRRLKR
301 MRRVEKKSAA FARILDPAYQ VDKGGRVRFV VELADPKLEV KWYKNGQEIR
351 PSTKYIFEHK GCERIMFINN CVLTDDSEYY VTAGDEKCST ELFVREPPVM
401 VTKQLEDTKA YCGERVELEC EVSEDDANVK WFKNGEEIIP GPKSRYHIRV
451 EGKKHILIID GATKADSAEY SVMTTGGQSS AKLSVDLKPL KILTPLTDQT
501 VNLGKEICLK CEVSENITGK WTKNGLPVQE TDHLKIVHKG RIHKLVIANA
551 LVEDEGDYVF TPDDYSVTLP AKVHVVDPPK INLDGLDADN TVTVIAGNKL
601 RLEIPISGEP PPKALWSRAD KAIMEGSGRI RSENYPDSTT LVIDIAEKDD
651 SGVYHINLKN EAGEAHASIK IKVVDIPDPP VAPNVTDVGD DWCIMTWDPP
701 LYDGGSPILG YFIERKKKQS SRWMRLNFDL CKETIFEPKK MIEGVAYEVR
751 IFAVNAVGIS KPSMPSKPFV PLAVTSPPTL LTVDSVTDTT VTMKWRPPDQ
801 IGAAGLDGYV LEYCFEGSTS AKQSDENGEA AYDLPADDWI AANTELIEKT
851 KFTITGLPTD SRIYVRVKAV NAAGASEPKY YSQPILVKEI IEAPKIRIPR
901 HLKQTYIRRV GEAINLVIPF QGKPRPELIW TKDGAPIDKN QINIRNSETD
951 TIVFIRKAER SHSGKYDLQV KVEKFVETAS IDIRVIDRPG PPQLVKIEDV
1001 WGENVALSWT PPRDDGNTAI TGYTIQKADK KSMEWFTVIE HYHRTSATIT
1051 ELVIGNEYYF RVFAENMCGL SEDATMTKES AVIAKDGKIY KNPVYEDFDF
1101 TEAPMFTQPL VNTYAVAGYN ATLNCSVRGN PKPKITWMKN KVVIKDDPRY
1151 RMFSNQGVCT LEIRKPSPYD GGTYCCKAVN DLGTVEIECK LEVKVVAQ
```

Show predicted peptides also

Sort Peptides By Residue Number Increasing Mass Decreasing Mass

Start	-	End	Observed	Mr(expt)	Mr(calc)	ppm	Miss	Sequence
128	-	139	1318.7582	1317.7509	1317.7656	-11	1	K.VGENITFIAKVK.A
166	-	175	1300.6644	1299.6571	1299.6935	-28	1	K.HLQLKETFER.H
179	-	190	1486.8057	1485.7984	1485.7901	6	1	R.VYTFEMQIIKAK.E Oxidation (M)
191	-	198	986.4511	985.4438	985.4253	19	0	K.ENYAGNYR.C
235	-	252	1837.8254	1836.8181	1836.8378	-11	0	R.SGEGQDDAGELDFSGLLK.R
235	-	253	1993.9484	1992.9411	1992.9389	1	1	R.SGEGQDDAGELDFSGLLKR.R
255	-	272	2195.1038	2194.0965	2194.1157	-9	0	R.EVKPQEEQPEVDVWELLK.N
314	-	326	1431.7261	1430.7188	1430.7518	-23	1	R.ILDPAYQVDKGGR.V
434	-	445	1296.6675	1295.6602	1295.6833	-18	1	K.NGEEIIPGPKSR.Y
446	-	453	1001.5549	1000.5476	1000.5454	2	1	R.YHIRVEGK.K
600	-	613	1545.8552	1544.8479	1544.8926	-29	1	K.LRLEIPISGEPPPK.A
740	-	750	1310.6544	1309.6471	1309.6700	-17	1	K.KMIEGVAYEVR.I Oxidation (M)
850	-	862	1436.7306	1435.7233	1435.7671	-30	1	K.TKFTITGLPTDSR.I
852	-	862	1207.6119	1206.6046	1206.6245	-16	0	K.FTITGLPTDSR.I
910	-	932	2605.5559	2604.5486	2604.4792	27	0	R.VGEAINLVIPFQGKPRPELIWTK.D
933	-	945	1453.7384	1452.7311	1452.7685	-26	1	K.DGAPIDKNQINIR.N
946	-	956	1294.6444	1293.6371	1293.6565	-15	0	R.NSETDTIVFIR.K

975 - 984 1150.5973 1149.5900 1149.6030 -11 0 K.FVETASIDIR.V
1032 - 1044 1734.7813 1733.7740 1733.7984 -14 0 K.SMEWFTVIEHYHR.T
1032 - 1044 1750.7714 1749.7641 1749.7933 -17 0 K.SMEWFTVIEHYHR.T Oxidation (M)
1045 - 1061 1976.9685 1975.9612 1975.9891 -14 0 R.TSATITELVIGNEYYFR.V
1152 - 1164 1554.7094 1553.7021 1553.7330 -20 0 R.MFSNQGVCTLEIR.K
1152 - 1164 1570.7126 1569.7053 1569.7280 -14 0 R.MFSNQGVCTLEIR.K Oxidation (M)
No match to: 855.0688, 971.4968, 980.5079, 1028.5073, 1082.5900, 1088.5948, 1109.5083, 1110.5568, 1232.5300, 1234.6511, 1314.663

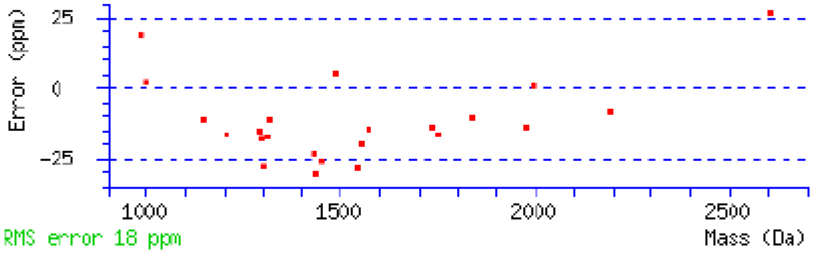

BAND 4

Mascot Search Results

Email :
Search title : SampleSetID: 824, AnalysisID: 7242, MaldiWellID: 69607, SpectrumID: 154532, Path=\180719\MS\18-106 NCBI Mammalia
Database : NCBInr 20120508 (17919084 sequences; 6150218869 residues)
Taxonomy : Mammalia (mammals) (1061927 sequences)
Timestamp : 19 Jul 2018 at 12:00:08 GMT
Top Score : 463 for gi|154426116, Phosphorylase, glycogen, muscle [Bos taurus]

Mascot Score Histogram

Protein score is -10*Log(P), where P is the probability that the observed match is a random event.
Protein scores greater than 73 are significant (p<0.05).

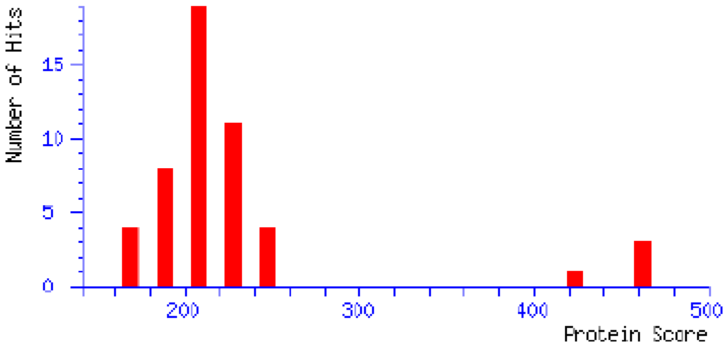

Protein Summary Report

Format As Protein Summary Help
Significance threshold p< 0.05 Max. number of hits 20
Re-Search All Search Unmatched

Index

Accession Mass Score Description
1. gi|154426116 97683 463 Phosphorylase, glycogen, muscle [Bos taurus]
2. gi|296471575 97674 463 glycogen phosphorylase, muscle form [Bos taurus]
3. gi|57163939 97702 459 glycogen phosphorylase, muscle form [Ovis aries]
4. gi|28461197 97688 414 glycogen phosphorylase, muscle form [Bos taurus]
5. gi|335281568 93593 248 PREDICTED: glycogen phosphorylase, muscle form isoform 2 [Sus scrofa]
6. gi|335281566 97665 244 PREDICTED: glycogen phosphorylase, muscle form isoform 1 [Sus scrofa]
7. gi|106073338 84382 242 muscle glycogen phosphorylase [Sus scrofa]
8. gi|335281570 87764 237 PREDICTED: glycogen phosphorylase, muscle form isoform 3 [Sus scrofa]
9. gi|300119711 97650 232 muscle glycogen phosphorylase [Sus scrofa]
10. gi|6730143 96157 222 Chain A, Identification And Structural Characterization Of A Novel Allosteric Binding Site Of Glycogen Phosphorylase B
11. gi|134104402 95535 220 Chain A, Structure Of Rabbit Muscle Glycogen Phosphorylase In Complex With Ligand
12. gi|134104414 95606 220 Chain A, Structure Of Rabbit Muscle Glycogen Phosphorylase In Complex With Thienopyrrole
13. gi|316983251 97506 220 Chain A, The Binding Of Beta-D-Glucopyranosyl-Thiosemicarbazone Derivatives To Glycogen Phosphorylase: A New Class Of Inhibitors
14. gi|194388822 96356 218 unnamed protein product [Homo sapiens]
15. gi|442605 95987 218 Chain A, Control Of Phosphorylase B Conformation By A Modified Cofactor: Crystallographic Studies On R-State Glycogen Phosphorylase Reconst
16. gi|297688261 96248 218 PREDICTED: glycogen phosphorylase, muscle form-like isoform 2 [Pongo abelii]

17. gi|66361339 97593 218 Chain A, Glycogen Phosphorylase Amp Site Inhibitor Complex
18. gi|126030531 97593 218 Chain A, The Crystal Structure Of The Glycogen Phosphorylase B- 1ab Complex
19. gi|93278542 97494 218 Chain A, Crystal Structure Of Human Muscle Glycogen Phosphorylase A With Amp And Glucose
20. gi|8569323 97634 217 Chain A, Synergistic Inhibition Of Glycogen Phosphorylase A By A Potential Antidiabetic Drug And Caffeine

Results List

1. gi|154426116 Mass: 97683 Score: 463 Expect: 5.3e-041 Matches: 47
Phosphorylase, glycogen, muscle [Bos taurus]
Observed Mr(expt) Mr(calc) ppm Start End Miss Peptide
872.4761 871.4688 871.4763 -8.60 353 - 359 0 R.ILVDQER.L
893.4103 892.4030 892.4079 -5.46 186 - 192 0 R.YGNPWEK.A
963.4651 962.4578 962.4604 -2.63 236 - 243 0 R.NNVVNTMR.L + Oxidation (M)
994.5660 993.5587 993.5760 -17.38 35 - 42 0 R.HLHFTLVK.D
1053.5698 1052.5625 1052.5655 -2.79 643 - 650 0 R.VIFLENYR.V
1086.5554 1085.5481 1085.5506 -2.24 824 - 832 0 R.EIWGVEPTR.Q
1117.5605 1116.5532 1116.5564 -2.83 415 - 425 0 R.VAAAFPGDVDR.L
1133.6079 1132.6006 1132.6029 -2.03 594 - 602 1 K.EPNKFFVPR.T
1145.5627 1144.5554 1144.5553 0.11 162 - 170 0 R.YEFGIFNQK.I
1262.6217 1261.6144 1261.5867 22.0 774 - 783 0 K.VFADYEEYVK.C
1277.6423 1276.6350 1276.6339 0.87 546 - 555 1 K.FSAYLEKEYK.V
1381.6793 1380.6720 1380.6714 0.46 51 - 61 0 R.DYYFALAYTVR.D
1386.7540 1385.7467 1385.7415 3.74 415 - 427 1 R.VAAAFPGDVDRLR.R
1426.7781 1425.7708 1425.7728 -1.39 400 - 410 0 R.HLQIIYEINQR.F
1442.6910 1441.6837 1441.6878 -2.81 279 - 290 0 R.VLYPNDNFFEGK.E
1456.7322 1455.7249 1455.7245 0.26 522 - 533 0 K.LLSYVDDESFIR.D
1473.8547 1472.8474 1472.8504 -1.99 388 - 399 0 R.WPVHLIETLLPR.H
1534.7764 1533.7691 1533.7675 1.09 508 - 520 0 R.IGEEYIADLDQLR.K
1536.7903 1535.7830 1535.8024 -12.61 83 - 94 0 R.IYYLSLEFYIGR.T
1537.7914 1536.7841 1536.7500 22.2 772 - 783 1 R.FKVFADYEEYVK.C
1557.8268 1556.8195 1556.8198 -0.19 353 - 364 1 R.ILVDQERLEWEK.A
1561.7968 1560.7895 1560.7937 -2.65 741 - 754 0 R.HVIDQLSSGFFSPK.Q
1566.7914 1565.7841 1565.7838 0.20 257 - 270 0 K.DFNVGGYIQAVLDR.N
1584.8268 1583.8195 1583.8195 0.01 521 - 533 1 R.KLLSYVDDESFIR.D
1623.8685 1622.8612 1622.8780 -10.36 556 - 569 0 K.VHINPNSLFDIQVK.R
1662.8694 1661.8621 1661.8624 -0.17 508 - 521 1 R.IGEEYIADLDQLRK.L
1689.8866 1688.8793 1688.8787 0.36 193 - 206 0 K.ARPEFTLPVHFYGR.V
1756.9471 1755.9398 1755.9454 -3.17 577 - 590 0 R.QLLNCLHVITLYNR.I
1792.7952 1791.7879 1791.7886 -0.39 171 - 185 0 K.ISGGWQMEEADDWLR.Y
1808.7908 1807.7835 1807.7835 -0.01 171 - 185 0 K.ISGGWQMEEADDWLR.Y + Oxidation (M)
1835.8369 1834.8296 1834.8196 5.47 774 - 787 1 K.VFADYEEYVKCQER.V
1840.9187 1839.9114 1839.9155 -2.23 279 - 293 1 R.VLYPNDNFFEGKELR.L
1869.9556 1868.9483 1868.9520 -1.96 522 - 537 1 K.LLSYVDDESFIRDVAK.V
1874.9188 1873.9115 1873.8992 6.56 372 - 387 0 K.TCAYTNHTVLPEALER.W
1886.9027 1885.8954 1885.8958 -0.21 726 - 740 1 K.GYNAQEYYDRIPELR.H
1889.9683 1888.9610 1888.9795 -9.79 817 - 832 1 R.TIAQYAREIWGVEPTR.Q
2007.1241 2006.1168 2006.1200 -1.60 294 - 310 1 R.LKQEYFVVAATLQDIIR.R
2028.0472 2027.0399 2027.0444 -2.22 491 - 507 1 R.RWLVMCNPGLAEIIAER.I
2044.0458 2043.0385 2043.0394 -0.41 491 - 507 1 R.RWLVMCNPGLAEIIAER.I + Oxidation (M)
2059.0444 2058.0371 2058.0323 2.34 51 - 67 1 R.DYYFALAYTVRDHLVGR.W
2118.1248 2117.1175 2117.1303 -6.03 334 - 352 0 K.VAIQLNDTHPSLAIPELMR.I
2159.2029 2158.1956 2158.1859 4.52 623 - 642 1 K.LITAIGDVVNHDPVVGDRLR.V
2307.1401 2306.1328 2306.1406 -3.36 216 - 235 0 K.WVDTQVVLAMPYDTPVPGYR.N
2323.1353 2322.1280 2322.1355 -3.21 216 - 235 0 K.WVDTQVVLAMPYDTPVPGYR.N + Oxidation (M)
2464.2288 2463.2215 2463.2506 -11.81 257 - 278 1 K.DFNVGGYIQAVLDRNLAENISR.V
2466.2324 2465.2251 2465.2339 -3.57 249 - 270 1 K.APNDFNLKDFNVGGYIQAVLDR.N
3153.5139 3152.5066 3152.5965 -28.49 325 - 352 1 R.TNFDAFPDKVAIQLNDTHPSLAIPELMR.I
No match to: 1068.5479, 1115.5763, 1174.6033, 1261.7012, 1271.6656, 1320.6438, 1372.7607, 1672.8704, 1716.8770, 1739.9244, 1744.8021, 1868.9391, 1914.9526,
1943.9541, 2184.1106, 2225.1584, 2259.1499, 2371.2534

2. gi|296471575 Mass: 97674 Score: 463 Expect: 5.3e-041 Matches: 47
glycogen phosphorylase, muscle form [Bos taurus]
Observed Mr(expt) Mr(calc) ppm Start End Miss Peptide
872.4761 871.4688 871.4763 -8.60 353 - 359 0 R.ILVDQER.L
893.4103 892.4030 892.4079 -5.46 186 - 192 0 R.YGNPWEK.A
963.4651 962.4578 962.4604 -2.63 236 - 243 0 R.NNVVNTMR.L + Oxidation (M)
994.5660 993.5587 993.5760 -17.38 35 - 42 0 R.HLHFTLVK.D
1053.5698 1052.5625 1052.5655 -2.79 643 - 650 0 R.VIFLENYR.V
1086.5554 1085.5481 1085.5506 -2.24 824 - 832 0 R.EIWGVEPTR.Q
1117.5605 1116.5532 1116.5564 -2.83 415 - 425 0 R.VAAAFPGDVDR.L
1133.6079 1132.6006 1132.6029 -2.03 594 - 602 1 K.EPNKFFVPR.T
1145.5627 1144.5554 1144.5553 0.11 162 - 170 0 R.YEFGIFNQK.I
1262.6217 1261.6144 1261.5867 22.0 774 - 783 0 K.VFADYEEYVK.C
1277.6423 1276.6350 1276.6339 0.87 546 - 555 1 K.FSAYLEKEYK.V
1381.6793 1380.6720 1380.6714 0.46 51 - 61 0 R.DYYFALAYTVR.D
1386.7540 1385.7467 1385.7415 3.74 415 - 427 1 R.VAAAFPGDVDRLR.R
1426.7781 1425.7708 1425.7728 -1.39 400 - 410 0 R.HLQIIYEINQR.F
1442.6910 1441.6837 1441.6878 -2.81 279 - 290 0 R.VLYPNDNFFEGK.E
1456.7322 1455.7249 1455.7245 0.26 522 - 533 0 K.LLSYVDDESFIR.D
1473.8547 1472.8474 1472.8504 -1.99 388 - 399 0 R.WPVHLIETLLPR.H
1534.7764 1533.7691 1533.7675 1.09 508 - 520 0 R.IGEEYIADLDQLR.K
1536.7903 1535.7830 1535.8024 -12.61 83 - 94 0 R.IYYLSLEFYIGR.T
1537.7914 1536.7841 1536.7500 22.2 772 - 783 1 R.FKVFADYEEYVK.C
1557.8268 1556.8195 1556.8198 -0.19 353 - 364 1 R.ILVDQERLEWEK.A
1561.7968 1560.7895 1560.7937 -2.65 741 - 754 0 R.HVIDQLSSGFFSPK.Q
1566.7914 1565.7841 1565.7838 0.20 257 - 270 0 K.DFNVGGYIQAVLDR.N
1584.8268 1583.8195 1583.8195 0.01 521 - 533 1 R.KLLSYVDDESFIR.D
1623.8685 1622.8612 1622.8780 -10.36 556 - 569 0 K.VHINPNSLFDIQVK.R
1662.8694 1661.8621 1661.8624 -0.17 508 - 521 1 R.IGEEYIADLDQLRK.L
1689.8866 1688.8793 1688.8787 0.36 193 - 206 0 K.ARPEFTLPVHFYGR.V
1756.9471 1755.9398 1755.9454 -3.17 577 - 590 0 R.QLLNCLHVITLYNR.I
1792.7952 1791.7879 1791.7886 -0.39 171 - 185 0 K.ISGGWQMEEADDWLR.Y
1808.7908 1807.7835 1807.7835 -0.01 171 - 185 0 K.ISGGWQMEEADDWLR.Y + Oxidation (M)
1835.8369 1834.8296 1834.8196 5.47 774 - 787 1 K.VFADYEEYVKCQER.V

1840.9187 1839.9114 1839.9155 -2.23 279 - 293 1 R.VLYPNDNFFEGKELR.L
1869.9556 1868.9483 1868.9520 -1.96 522 - 537 1 K.LLSYVDDESFIRDVAK.V
1874.9188 1873.9115 1873.8992 6.56 372 - 387 0 K.TCAYTNHTVLPEALER.W
1886.9027 1885.8954 1885.8958 -0.21 726 - 740 1 K.GYNAQEYYDRIPELR.H
1889.9683 1888.9610 1888.9795 -9.79 817 - 832 1 R.TIAQYAREIWGVEPTR.Q
2007.1241 2006.1168 2006.1200 -1.60 294 - 310 1 R.LKQEYFVVAATLQDIIR.R
2028.0472 2027.0399 2027.0444 -2.22 491 - 507 1 R.RWLVMCNPGLAEIIAER.I
2044.0458 2043.0385 2043.0394 -0.41 491 - 507 1 R.RWLVMCNPGLAEIIAER.I + Oxidation (M)
2059.0444 2058.0371 2058.0323 2.34 51 - 67 1 R.DYYFALAYTVRDHLVGR.W
2118.1248 2117.1175 2117.1303 -6.03 334 - 352 0 K.VAIQLNDTHPSLAIPELMR.I
2159.2029 2158.1956 2158.1859 4.52 623 - 642 1 K.LITAIGDVVNHDPVVGDRLR.V
2307.1401 2306.1328 2306.1406 -3.36 216 - 235 0 K.WVDTQVVLAMPYDTPVPGYR.N
2323.1353 2322.1280 2322.1355 -3.21 216 - 235 0 K.WVDTQVVLAMPYDTPVPGYR.N + Oxidation (M)
2464.2288 2463.2215 2463.2506 -11.81 257 - 278 1 K.DFNVGGYIQAVLDRNLAENISR.V
2466.2324 2465.2251 2465.2339 -3.57 249 - 270 1 K.APNDFNLKDFNVGGYIQAVLDR.N
3153.5139 3152.5066 3152.5965 -28.49 325 - 352 1 R.TNFDAFPDKVAIQLNDTHPSLAIPELMR.I
No match to: 1068.5479, 1115.5763, 1174.6033, 1261.7012, 1271.6656, 1320.6438, 1372.7607, 1672.8704, 1716.8770, 1739.9244, 1744.8021, 1868.9391, 1914.9526,
1943.9541, 2184.1106, 2225.1584, 2259.1499, 2371.2534
3. gi|57163939 Mass: 97702 Score: 459 Expect: 1.3e-040 Matches: 46
glycogen phosphorylase, muscle form [Ovis aries]
Observed Mr(expt) Mr(calc) ppm Start End Miss Peptide
872.4761 871.4688 871.4763 -8.60 353 - 359 0 R.ILVDQER.L
893.4103 892.4030 892.4079 -5.46 186 - 192 0 R.YGNPWEK.A
963.4651 962.4578 962.4604 -2.63 236 - 243 0 R.NNVVNTMR.L + Oxidation (M)
994.5660 993.5587 993.5760 -17.38 35 - 42 0 R.HLHFTLVK.D
1053.5698 1052.5625 1052.5655 -2.79 643 - 650 0 R.VIFLENYR.V
1086.5554 1085.5481 1085.5506 -2.24 824 - 832 0 R.EIWGVEPTR.Q
1117.5605 1116.5532 1116.5564 -2.83 415 - 425 0 R.VAAAFPGDVDR.L
1133.6079 1132.6006 1132.6029 -2.03 594 - 602 1 K.EPNKFFVPR.T
1145.5627 1144.5554 1144.5553 0.11 162 - 170 0 R.YEFGIFNQK.I
1262.6217 1261.6144 1261.5867 22.0 774 - 783 0 K.VFADYEEYVK.C
1277.6423 1276.6350 1276.6339 0.87 546 - 555 1 K.FSAYLEKEYK.V
1381.6793 1380.6720 1380.6714 0.46 51 - 61 0 R.DYYFALAYTVR.D
1386.7540 1385.7467 1385.7415 3.74 415 - 427 1 R.VAAAFPGDVDRLR.R
1426.7781 1425.7708 1425.7728 -1.39 400 - 410 0 R.HLQIIYEINQR.F
1442.6910 1441.6837 1441.6878 -2.81 279 - 290 0 R.VLYPNDNFFEGK.E
1456.7322 1455.7249 1455.7245 0.26 522 - 533 0 K.LLSYVDDESFIR.D
1473.8547 1472.8474 1472.8504 -1.99 388 - 399 0 R.WPVHLIETLLPR.H
1534.7764 1533.7691 1533.7675 1.09 508 - 520 0 R.IGEEYIADLDQLR.K
1536.7903 1535.7830 1535.8024 -12.61 83 - 94 0 R.IYYLSLEFYIGR.T
1537.7914 1536.7841 1536.7500 22.2 772 - 783 1 R.FKVFADYEEYVK.C
1557.8268 1556.8195 1556.8198 -0.19 353 - 364 1 R.ILVDQERLEWEK.A
1566.7914 1565.7841 1565.7838 0.20 257 - 270 0 K.DFNVGGYIQAVLDR.N
1584.8268 1583.8195 1583.8195 0.01 521 - 533 1 R.KLLSYVDDESFIR.D
1623.8685 1622.8612 1622.8780 -10.36 556 - 569 0 K.VHINPNSLFDIQVK.R
1662.8694 1661.8621 1661.8624 -0.17 508 - 521 1 R.IGEEYIADLDQLRK.L
1689.8866 1688.8793 1688.8787 0.36 193 - 206 0 K.ARPEFTLPVHFYGR.V
1756.9471 1755.9398 1755.9454 -3.17 577 - 590 0 R.QLLNCLHVITLYNR.I
1792.7952 1791.7879 1791.7886 -0.39 171 - 185 0 K.ISGGWQMEEADDWLR.Y
1808.7908 1807.7835 1807.7835 -0.01 171 - 185 0 K.ISGGWQMEEADDWLR.Y + Oxidation (M)
1835.8369 1834.8296 1834.8196 5.47 774 - 787 1 K.VFADYEEYVKCQER.V
1840.9187 1839.9114 1839.9155 -2.23 279 - 293 1 R.VLYPNDNFFEGKELR.L
1869.9556 1868.9483 1868.9520 -1.96 522 - 537 1 K.LLSYVDDESFIRDVAK.V
1874.9188 1873.9115 1873.8992 6.56 372 - 387 0 K.TCAYTNHTVLPEALER.W
1886.9027 1885.8954 1885.8958 -0.21 726 - 740 1 K.GYNAQEYYDRIPELR.H
1889.9683 1888.9610 1888.9795 -9.79 817 - 832 1 R.TIAQYAREIWGVEPTR.Q
2007.1241 2006.1168 2006.1200 -1.60 294 - 310 1 R.LKQEYFVVAATLQDIIR.R
2044.0458 2043.0385 2043.0789 -19.76 553 - 569 1 K.EYKVHINPNSLFDIQVK.R
2059.0444 2058.0371 2058.0323 2.34 51 - 67 1 R.DYYFALAYTVRDHLVGR.W
2118.1248 2117.1175 2117.1303 -6.03 334 - 352 0 K.VAIQLNDTHPSLAIPELMR.I
2159.2029 2158.1956 2158.1859 4.52 623 - 642 1 R.LITAIGDVVNHDPVVGDRLR.V
2184.1106 2183.1033 2183.1739 -32.32 736 - 754 1 R.IPELRHIIDQLSSGFFSPK.Q
2307.1401 2306.1328 2306.1406 -3.36 216 - 235 0 K.WVDTQVVLAMPYDTPVPGYR.N
2323.1353 2322.1280 2322.1355 -3.21 216 - 235 0 K.WVDTQVVLAMPYDTPVPGYR.N + Oxidation (M)
2464.2288 2463.2215 2463.2506 -11.81 257 - 278 1 K.DFNVGGYIQAVLDRNLAENISR.V
2466.2324 2465.2251 2465.2339 -3.57 249 - 270 1 K.APNDFNLKDFNVGGYIQAVLDR.N
3153.5139 3152.5066 3152.5965 -28.49 325 - 352 1 R.TNFDAFPDKVAIQLNDTHPSLAIPELMR.I
No match to: 1068.5479, 1115.5763, 1174.6033, 1261.7012, 1271.6656, 1320.6438, 1372.7607, 1561.7968, 1672.8704, 1716.8770, 1739.9244, 1744.8021, 1868.9391,
1914.9526, 1943.9541, 2028.0472, 2225.1584, 2259.1499, 2371.2534
4. gi|28461197 Mass: 97688 Score: 414 Expect: 4.2e-036 Matches: 44
glycogen phosphorylase, muscle form [Bos taurus]
Observed Mr(expt) Mr(calc) ppm Start End Miss Peptide
872.4761 871.4688 871.4763 -8.60 353 - 359 0 R.ILVDQER.L
893.4103 892.4030 892.4079 -5.46 186 - 192 0 R.YGNPWEK.A
963.4651 962.4578 962.4604 -2.63 236 - 243 0 R.NNVVNTMR.L + Oxidation (M)
994.5660 993.5587 993.5760 -17.38 35 - 42 0 R.HLHFTLVK.D
1053.5698 1052.5625 1052.5655 -2.79 643 - 650 0 R.VIFLENYR.V
1086.5554 1085.5481 1085.5506 -2.24 824 - 832 0 R.EIWGVEPTR.Q
1117.5605 1116.5532 1116.5564 -2.83 415 - 425 0 R.VAAAFPGDVDR.L
1133.6079 1132.6006 1132.6029 -2.03 594 - 602 1 K.EPNKFFVPR.T
1145.5627 1144.5554 1144.5553 0.11 162 - 170 0 R.YEFGIFNQK.I
1277.6423 1276.6350 1276.6339 0.87 546 - 555 1 K.FSAYLEKEYK.V
1381.6793 1380.6720 1380.6714 0.46 51 - 61 0 R.DYYFALAYTVR.D
1386.7540 1385.7467 1385.7415 3.74 415 - 427 1 R.VAAAFPGDVDRLR.R
1426.7781 1425.7708 1425.7728 -1.39 400 - 410 0 R.HLQIIYEINQR.F
1442.6910 1441.6837 1441.6878 -2.81 279 - 290 0 R.VLYPNDNFFEGK.E
1456.7322 1455.7249 1455.7245 0.26 522 - 533 0 K.LLSYVDDESFIR.D
1473.8547 1472.8474 1472.8504 -1.99 388 - 399 0 R.WPVHLIETLLPR.H
1534.7764 1533.7691 1533.7675 1.09 508 - 520 0 R.IGEEYIADLDQLR.K
1536.7903 1535.7830 1535.8024 -12.61 83 - 94 0 R.IYYLSLEFYIGR.T

1557.8268 1556.8195 1556.8198 -0.19 353 - 364 1 R.ILVDQERLEWEK.A
1561.7968 1560.7895 1560.7937 -2.65 741 - 754 0 R.HVIDQLSSGFFSPK.Q
1566.7914 1565.7841 1565.7838 0.20 257 - 270 0 K.DFNVGGYIQAVLDR.N
1584.8268 1583.8195 1583.8195 0.01 521 - 533 1 R.KLLSYVDDESFIR.D
1623.8685 1622.8612 1622.8780 -10.36 556 - 569 0 K.VHINPNSLFDIQVK.R
1662.8694 1661.8621 1661.8624 -0.17 508 - 521 1 R.IGEEYIADLDQLRK.L
1689.8866 1688.8793 1688.8787 0.36 193 - 206 0 K.ARPEFTLPVHFYGR.V
1756.9471 1755.9398 1755.9454 -3.17 577 - 590 0 R.QLLNCLHVITLYNR.I
1792.7952 1791.7879 1791.7886 -0.39 171 - 185 0 K.ISGGWQMEEADDWLR.Y
1808.7908 1807.7835 1807.7835 -0.01 171 - 185 0 K.ISGGWQMEEADDWLR.Y + Oxidation (M)
1840.9187 1839.9114 1839.9155 -2.23 279 - 293 1 R.VLYPNDNFFEGKELR.L
1869.9556 1868.9483 1868.9520 -1.96 522 - 537 1 K.LLSYVDDESFIRDVAK.V
1874.9188 1873.9115 1873.8992 6.56 372 - 387 0 K.TCAYTNHTVLPEALER.W
1886.9027 1885.8954 1885.8958 -0.21 726 - 740 1 K.GYNAQEYYDRIPELR.H
1889.9683 1888.9610 1888.9795 -9.79 817 - 832 1 R.TIAQYAREIWGVEPTR.Q
2007.1241 2006.1168 2006.1200 -1.60 294 - 310 1 R.LKQEYFVVAATLQDIIR.R
2028.0472 2027.0399 2027.0444 -2.22 491 - 507 1 R.RWLVMCNPGLAEIIAER.I
2044.0458 2043.0385 2043.0394 -0.41 491 - 507 1 R.RWLVMCNPGLAEIIAER.I + Oxidation (M)
2059.0444 2058.0371 2058.0323 2.34 51 - 67 1 R.DYYFALAYTVRDHLVGR.W
2118.1248 2117.1175 2117.1303 -6.03 334 - 352 0 K.VAIQLNDTHPSLAIPELMR.I
2159.2029 2158.1956 2158.1859 4.52 623 - 642 1 K.LITAIGDVVNHDPVVGDRLR.V
2307.1401 2306.1328 2306.1406 -3.36 216 - 235 0 K.WVDTQVVLAMPYDTPVPGYR.N
2323.1353 2322.1280 2322.1355 -3.21 216 - 235 0 K.WVDTQVVLAMPYDTPVPGYR.N + Oxidation (M)
2464.2288 2463.2215 2463.2506 -11.81 257 - 278 1 K.DFNVGGYIQAVLDRNLAENISR.V
2466.2324 2465.2251 2465.2339 -3.57 249 - 270 1 K.APNDFNLKDFNVGGYIQAVLDR.N
3153.5139 3152.5066 3152.5965 -28.49 325 - 352 1 R.TNFDAFPDKVAIQLNDTHPSLAIPELMR.I
No match to: 1068.5479, 1115.5763, 1174.6033, 1261.7012, 1262.6217, 1271.6656, 1320.6438, 1372.7607, 1537.7914, 1672.8704, 1716.8770, 1739.9244, 1744.8021,
1835.8369, 1868.9391, 1914.9526, 1943.9541, 2184.1106, 2225.1584, 2259.1499, 2371.2534
5. gi|335281568 Mass: 93593 Score: 248 Expect: 1.7e-019 Matches: 31
PREDICTED: glycogen phosphorylase, muscle form isoform 2 [Sus scrofa]
Observed Mr(expt) Mr(calc) ppm Start End Miss Peptide
963.4651 962.4578 962.4604 -2.63 202 - 209 0 R.NNVVNTMR.L + Oxidation (M)
994.5660 993.5587 993.5760 -17.38 35 - 42 0 R.HLHFTLVK.D
1053.5698 1052.5625 1052.5614 1.04 799 - 807 1 R.QRLPAPDEK.I
1133.6079 1132.6006 1132.5513 43.6 381 - 391 0 R.VAAAYPGDVDR.L
1145.5627 1144.5554 1144.5553 0.11 128 - 136 0 R.YEFGIFNQK.I
1262.6217 1261.6144 1261.5867 22.0 740 - 749 0 K.VFADYEDYIK.C
1381.6793 1380.6720 1380.6714 0.46 51 - 61 0 R.DYYFALAYTVR.D
1426.7781 1425.7708 1425.7728 -1.39 366 - 376 0 R.HLQIIYEINQR.F
1442.6910 1441.6837 1441.6878 -2.81 245 - 256 0 R.VLYPNDNFFEGK.E
1534.7764 1533.7691 1533.7675 1.09 474 - 486 0 R.IGEEYIADLDQLR.K
1537.7914 1536.7841 1536.7500 22.2 738 - 749 1 R.FKVFADYEDYIK.C
1561.7968 1560.7895 1560.7937 -2.65 707 - 720 0 R.HVIDQLSSGFFSPK.Q
1566.7914 1565.7841 1565.7838 0.20 223 - 236 0 K.DFNVGGYIQAVLDR.N
1623.8685 1622.8612 1622.8780 -10.36 522 - 535 0 K.VHINPNSLFDIQVK.R
1662.8694 1661.8621 1661.8624 -0.17 474 - 487 1 R.IGEEYIADLDQLRK.L
1689.8866 1688.8793 1688.8787 0.36 159 - 172 0 K.ARPEFTLPVHFYGR.V
1756.9471 1755.9398 1755.9454 -3.17 543 - 556 0 R.QLLNCLHVITLYNR.I
1835.8369 1834.8296 1834.8196 5.47 740 - 753 1 K.VFADYEDYIKCQER.V
1840.9187 1839.9114 1839.9155 -2.23 245 - 259 1 R.VLYPNDNFFEGKELR.L
1874.9188 1873.9115 1873.8992 6.56 338 - 353 0 R.TCAYTNHTVLPEALER.W
1886.9027 1885.8954 1885.8958 -0.21 692 - 706 1 K.GYNAQEYYDRIPELR.H
1889.9683 1888.9610 1889.0007 -21.00 589 - 606 0 K.LITAIGDVVNHDPVVGDR.L
2007.1241 2006.1168 2006.1200 -1.60 260 - 276 1 R.LKQEYFVVAATLQDIIR.R
2044.0458 2043.0385 2043.0789 -19.76 519 - 535 1 R.EYKVHINPNSLFDIQVK.R
2059.0444 2058.0371 2058.0323 2.34 51 - 67 1 R.DYYFALAYTVRDHLVGR.W
2118.1248 2117.1175 2117.1303 -6.03 300 - 318 0 K.VAIQLNDTHPSLAIPELMR.I
2159.2029 2158.1956 2158.1859 4.52 589 - 608 1 K.LITAIGDVVNHDPVVGDRLR.V
2307.1401 2306.1328 2306.1406 -3.36 182 - 201 0 K.WVDTQVVLAMPYDTPVPGYR.N
2323.1353 2322.1280 2322.1355 -3.21 182 - 201 0 K.WVDTQVVLAMPYDTPVPGYR.N + Oxidation (M)
2464.2288 2463.2215 2463.2506 -11.81 223 - 244 1 K.DFNVGGYIQAVLDRNLAENISR.V
2466.2324 2465.2251 2465.2339 -3.57 215 - 236 1 K.APNDFNLKDFNVGGYIQAVLDR.N
No match to: 872.4761, 893.4103, 1068.5479, 1086.5554, 1115.5763, 1117.5605, 1174.6033, 1261.7012, 1271.6656, 1277.6423, 1320.6438,
1372.7607, 1386.7540,
1456.7322, 1473.8547, 1536.7903, 1557.8268, 1584.8268, 1672.8704, 1716.8770, 1739.9244, 1744.8021, 1792.7952, 1808.7908, 1868.9391,
1869.9556, 1914.9526,
1943.9541, 2028.0472, 2184.1106, 2225.1584, 2259.1499, 2371.2534, 3153.5139
6. gi|335281566 Mass: 97665 Score: 244 Expect: 4.2e-019 Matches: 31
PREDICTED: glycogen phosphorylase, muscle form isoform 1 [Sus scrofa]
Observed Mr(expt) Mr(calc) ppm Start End Miss Peptide
963.4651 962.4578 962.4604 -2.63 236 - 243 0 R.NNVVNTMR.L + Oxidation (M)
994.5660 993.5587 993.5760 -17.38 35 - 42 0 R.HLHFTLVK.D
1053.5698 1052.5625 1052.5614 1.04 833 - 841 1 R.QRLPAPDEK.I
1133.6079 1132.6006 1132.5513 43.6 415 - 425 0 R.VAAAYPGDVDR.L
1145.5627 1144.5554 1144.5553 0.11 162 - 170 0 R.YEFGIFNQK.I
1262.6217 1261.6144 1261.5867 22.0 774 - 783 0 K.VFADYEDYIK.C
1381.6793 1380.6720 1380.6714 0.46 51 - 61 0 R.DYYFALAYTVR.D
1426.7781 1425.7708 1425.7728 -1.39 400 - 410 0 R.HLQIIYEINQR.F
1442.6910 1441.6837 1441.6878 -2.81 279 - 290 0 R.VLYPNDNFFEGK.E
1534.7764 1533.7691 1533.7675 1.09 508 - 520 0 R.IGEEYIADLDQLR.K
1537.7914 1536.7841 1536.7500 22.2 772 - 783 1 R.FKVFADYEDYIK.C
1561.7968 1560.7895 1560.7937 -2.65 741 - 754 0 R.HVIDQLSSGFFSPK.Q
1566.7914 1565.7841 1565.7838 0.20 257 - 270 0 K.DFNVGGYIQAVLDR.N
1623.8685 1622.8612 1622.8780 -10.36 556 - 569 0 K.VHINPNSLFDIQVK.R
1662.8694 1661.8621 1661.8624 -0.17 508 - 521 1 R.IGEEYIADLDQLRK.L
1689.8866 1688.8793 1688.8787 0.36 193 - 206 0 K.ARPEFTLPVHFYGR.V
1756.9471 1755.9398 1755.9454 -3.17 577 - 590 0 R.QLLNCLHVITLYNR.I
1835.8369 1834.8296 1834.8196 5.47 774 - 787 1 K.VFADYEDYIKCQER.V
1840.9187 1839.9114 1839.9155 -2.23 279 - 293 1 R.VLYPNDNFFEGKELR.L
1874.9188 1873.9115 1873.8992 6.56 372 - 387 0 R.TCAYTNHTVLPEALER.W
1886.9027 1885.8954 1885.8958 -0.21 726 - 740 1 K.GYNAQEYYDRIPELR.H

1889.9683 1888.9610 1889.0007 -21.00 623 - 640 0 K.LITAIGDVVNHDPVVGDR.L
2007.1241 2006.1168 2006.1200 -1.60 294 - 310 1 R.LKQEYFVVAATLQDIIR.R
2044.0458 2043.0385 2043.0789 -19.76 553 - 569 1 R.EYKVHINPNSLFDIQVK.R
2059.0444 2058.0371 2058.0323 2.34 51 - 67 1 R.DYYFALAYTVRDHLVGR.W
2118.1248 2117.1175 2117.1303 -6.03 334 - 352 0 K.VAIQLNDTHPSLAIPELMR.I
2159.2029 2158.1956 2158.1859 4.52 623 - 642 1 K.LITAIGDVVNHDPVVGDRLR.V
2307.1401 2306.1328 2306.1406 -3.36 216 - 235 0 K.WVDTQVVLAMPYDTPVPGYR.N
2323.1353 2322.1280 2322.1355 -3.21 216 - 235 0 K.WVDTQVVLAMPYDTPVPGYR.N + Oxidation (M)
2464.2288 2463.2215 2463.2506 -11.81 257 - 278 1 K.DFNVGGYIQAVLDRNLAENISR.V
2466.2324 2465.2251 2465.2339 -3.57 249 - 270 1 K.APNDFNLKDFNVGGYIQAVLDR.N
No match to: 872.4761, 893.4103, 1068.5479, 1086.5554, 1115.5763, 1117.5605, 1174.6033, 1261.7012, 1271.6656, 1277.6423, 1320.6438, 1372.7607, 1386.7540,
1456.7322, 1473.8547, 1536.7903, 1557.8268, 1584.8268, 1672.8704, 1716.8770, 1739.9244, 1744.8021, 1792.7952, 1808.7908, 1868.9391, 1869.9556, 1914.9526,
1943.9541, 2028.0472, 2184.1106, 2225.1584, 2259.1499, 2371.2534, 3153.5139
7. gi|106073338 Mass: 84382 Score: 242 Expect: 6.7e-019 Matches: 29
muscle glycogen phosphorylase [Sus scrofa]
Observed Mr(expt) Mr(calc) ppm Start End Miss Peptide
963.4651 962.4578 962.4604 -2.63 125 - 132 0 R.NNVVNTMR.L + Oxidation (M)
1053.5698 1052.5625 1052.5614 1.04 722 - 730 1 R.QRLPAPDEK.I
1133.6079 1132.6006 1132.5513 43.6 304 - 314 0 R.VAAAYPGDVDR.L
1145.5627 1144.5554 1144.5553 0.11 51 - 59 0 R.YEFGIFNQK.I
1262.6217 1261.6144 1261.5867 22.0 663 - 672 0 K.VFADYEDYIK.C
1320.6438 1319.6365 1319.7020 -49.60 492 - 504 1 R.TVMIGGKAAPGYR.M
1426.7781 1425.7708 1425.7728 -1.39 289 - 299 0 R.HLQIIYEINQR.F
1442.6910 1441.6837 1441.6878 -2.81 168 - 179 0 R.VLYPNDNFFEGK.E
1534.7764 1533.7691 1533.7675 1.09 397 - 409 0 R.IGEEYIADLDQLR.K
1537.7914 1536.7841 1536.7500 22.2 661 - 672 1 R.FKVFADYEDYIK.C
1566.7914 1565.7841 1565.7838 0.20 146 - 159 0 K.DFNVGGYIQAVLDR.N
1623.8685 1622.8612 1622.8780 -10.36 445 - 458 0 K.VHINPNSLFDIQVK.R
1662.8694 1661.8621 1661.8624 -0.17 397 - 410 1 R.IGEEYIADLDQLRK.L
1689.8866 1688.8793 1688.8787 0.36 82 - 95 0 K.ARPEFTLPVHFYGR.V
1756.9471 1755.9398 1755.9454 -3.17 466 - 479 0 R.QLLNCLHVITLYNR.I
1835.8369 1834.8296 1834.8196 5.47 663 - 676 1 K.VFADYEDYIKCQER.V
1840.9187 1839.9114 1839.9155 -2.23 168 - 182 1 R.VLYPNDNFFEGKELR.L
1874.9188 1873.9115 1873.8992 6.56 261 - 276 0 R.TCAYTNHTVLPEALER.W
1886.9027 1885.8954 1885.8958 -0.21 615 - 629 1 K.GYNAQEYYDRIPELR.H
1889.9683 1888.9610 1889.0007 -21.00 512 - 529 0 K.LITAIGDVVNHDPVVGDR.L
2007.1241 2006.1168 2006.1200 -1.60 183 - 199 1 R.LKQEYFVVAATLQDIIR.R
2044.0458 2043.0385 2043.0789 -19.76 442 - 458 1 R.EYKVHINPNSLFDIQVK.R
2118.1248 2117.1175 2117.1303 -6.03 223 - 241 0 K.VAIQLNDTHPSLAIPELMR.I
2159.2029 2158.1956 2158.1859 4.52 512 - 531 1 K.LITAIGDVVNHDPVVGDRLR.V
2184.1106 2183.1033 2183.1739 -32.32 625 - 643 1 R.IPELRHIIDQLSSGFFSPK.Q
2307.1401 2306.1328 2306.1406 -3.36 105 - 124 0 K.WVDTQVVLAMPYDTPVPGYR.N
2323.1353 2322.1280 2322.1355 -3.21 105 - 124 0 K.WVDTQVVLAMPYDTPVPGYR.N + Oxidation (M)
2464.2288 2463.2215 2463.2506 -11.81 146 - 167 1 K.DFNVGGYIQAVLDRNLAENISR.V
2466.2324 2465.2251 2465.2339 -3.57 138 - 159 1 K.APNDFNLKDFNVGGYIQAVLDR.N
No match to: 872.4761, 893.4103, 994.5660, 1068.5479, 1086.5554, 1115.5763, 1117.5605, 1174.6033, 1261.7012, 1271.6656, 1277.6423, 1372.7607, 1381.6793, 1386.7540,
1456.7322, 1473.8547, 1536.7903, 1557.8268, 1561.7968, 1584.8268, 1672.8704, 1716.8770, 1739.9244, 1744.8021, 1792.7952, 1808.7908, 1868.9391, 1869.9556,
1914.9526, 1943.9541, 2028.0472, 2059.0444, 2225.1584, 2259.1499, 2371.2534, 3153.5139
8. gi|335281570 Mass: 87764 Score: 237 Expect: 2.1e-018 Matches: 30
PREDICTED: glycogen phosphorylase, muscle form isoform 3 [Sus scrofa]
Observed Mr(expt) Mr(calc) ppm Start End Miss Peptide
963.4651 962.4578 962.4604 -2.63 148 - 155 0 R.NNVVNTMR.L + Oxidation (M)
994.5660 993.5587 993.5760 -17.38 35 - 42 0 R.HLHFTLVK.D
1053.5698 1052.5625 1052.5614 1.04 745 - 753 1 R.QRLPAPDEK.I
1133.6079 1132.6006 1132.5513 43.6 327 - 337 0 R.VAAAYPGDVDR.L
1262.6217 1261.6144 1261.5867 22.0 686 - 695 0 K.VFADYEDYIK.C
1381.6793 1380.6720 1380.6714 0.46 51 - 61 0 R.DYYFALAYTVR.D
1426.7781 1425.7708 1425.7728 -1.39 312 - 322 0 R.HLQIIYEINQR.F
1442.6910 1441.6837 1441.6878 -2.81 191 - 202 0 R.VLYPNDNFFEGK.E
1534.7764 1533.7691 1533.7675 1.09 420 - 432 0 R.IGEEYIADLDQLR.K
1537.7914 1536.7841 1536.7500 22.2 684 - 695 1 R.FKVFADYEDYIK.C
1561.7968 1560.7895 1560.7937 -2.65 653 - 666 0 R.HVIDQLSSGFFSPK.Q
1566.7914 1565.7841 1565.7838 0.20 169 - 182 0 K.DFNVGGYIQAVLDR.N
1623.8685 1622.8612 1622.8780 -10.36 468 - 481 0 K.VHINPNSLFDIQVK.R
1662.8694 1661.8621 1661.8624 -0.17 420 - 433 1 R.IGEEYIADLDQLRK.L
1689.8866 1688.8793 1688.8787 0.36 105 - 118 0 K.ARPEFTLPVHFYGR.V
1756.9471 1755.9398 1755.9454 -3.17 489 - 502 0 R.QLLNCLHVITLYNR.I
1835.8369 1834.8296 1834.8196 5.47 686 - 699 1 K.VFADYEDYIKCQER.V
1840.9187 1839.9114 1839.9155 -2.23 191 - 205 1 R.VLYPNDNFFEGKELR.L
1874.9188 1873.9115 1873.8992 6.56 284 - 299 0 R.TCAYTNHTVLPEALER.W
1886.9027 1885.8954 1885.8958 -0.21 638 - 652 1 K.GYNAQEYYDRIPELR.H
1889.9683 1888.9610 1889.0007 -21.00 535 - 552 0 K.LITAIGDVVNHDPVVGDR.L
2007.1241 2006.1168 2006.1200 -1.60 206 - 222 1 R.LKQEYFVVAATLQDIIR.R
2044.0458 2043.0385 2043.0789 -19.76 465 - 481 1 R.EYKVHINPNSLFDIQVK.R
2059.0444 2058.0371 2058.0323 2.34 51 - 67 1 R.DYYFALAYTVRDHLVGR.W
2118.1248 2117.1175 2117.1303 -6.03 246 - 264 0 K.VAIQLNDTHPSLAIPELMR.I
2159.2029 2158.1956 2158.1859 4.52 535 - 554 1 K.LITAIGDVVNHDPVVGDRLR.V
2307.1401 2306.1328 2306.1406 -3.36 128 - 147 0 K.WVDTQVVLAMPYDTPVPGYR.N
2323.1353 2322.1280 2322.1355 -3.21 128 - 147 0 K.WVDTQVVLAMPYDTPVPGYR.N + Oxidation (M)
2464.2288 2463.2215 2463.2506 -11.81 169 - 190 1 K.DFNVGGYIQAVLDRNLAENISR.V
2466.2324 2465.2251 2465.2339 -3.57 161 - 182 1 K.APNDFNLKDFNVGGYIQAVLDR.N
No match to: 872.4761, 893.4103, 1068.5479, 1086.5554, 1115.5763, 1117.5605, 1145.5627, 1174.6033, 1261.7012, 1271.6656, 1277.6423, 1320.6438, 1372.7607,
1386.7540, 1456.7322, 1473.8547, 1536.7903, 1557.8268, 1584.8268, 1672.8704, 1716.8770, 1739.9244, 1744.8021, 1792.7952, 1808.7908, 1868.9391, 1869.9556,
1914.9526, 1943.9541, 2028.0472, 2184.1106, 2225.1584, 2259.1499, 2371.2534, 3153.5139
9. gi|300119711 Mass: 97650 Score: 232 Expect: 6.7e-018 Matches: 30
muscle glycogen phosphorylase [Sus scrofa]

Observed Mr(expt) Mr(calc) ppm Start End Miss Peptide
963.4651 962.4578 962.4604 -2.63 236 - 243 0 R.NNVVNTMR.L + Oxidation (M)
994.5660 993.5587 993.5760 -17.38 35 - 42 0 R.HLHFTLVK.D
1053.5698 1052.5625 1052.5614 1.04 833 - 841 1 R.QRLPAPDEK.I
1133.6079 1132.6006 1132.5513 43.6 415 - 425 0 R.VAAAYPGDVDR.L
1145.5627 1144.5554 1144.5553 0.11 162 - 170 0 R.YEFGIFNQK.I
1262.6217 1261.6144 1261.5867 22.0 774 - 783 0 K.VFADYEDYIK.C
1426.7781 1425.7708 1425.7728 -1.39 400 - 410 0 R.HLQIIYEINQR.F
1442.6910 1441.6837 1441.6878 -2.81 279 - 290 0 R.VLYPNDNFFEGK.E
1534.7764 1533.7691 1533.7675 1.09 508 - 520 0 R.IGEEYIADLDQLR.K
1536.7903 1535.7830 1535.8024 -12.61 83 - 94 0 R.IYYLSLEFYIGR.T
1537.7914 1536.7841 1536.7500 22.2 772 - 783 1 R.FKVFADYEDYIK.C
1561.7968 1560.7895 1560.7937 -2.65 741 - 754 0 R.HVIDQLSSGFFSPK.Q
1566.7914 1565.7841 1565.7838 0.20 257 - 270 0 K.DFNVGGYIQAVLDR.N
1623.8685 1622.8612 1622.8780 -10.36 556 - 569 0 K.VHINPNSLFDIQVK.R
1662.8694 1661.8621 1661.8624 -0.17 508 - 521 1 R.IGEEYIADLDQLRK.L
1689.8866 1688.8793 1688.8787 0.36 193 - 206 0 K.ARPEFTLPVHFYGR.V
1756.9471 1755.9398 1755.9454 -3.17 577 - 590 0 R.QLLNCLHVITLYNR.I
1835.8369 1834.8296 1834.8196 5.47 774 - 787 1 K.VFADYEDYIKCQER.V
1840.9187 1839.9114 1839.9155 -2.23 279 - 293 1 R.VLYPNDNFFEGKELR.L
1874.9188 1873.9115 1873.8992 6.56 372 - 387 0 R.TCAYTNHTVLPEALER.W
1886.9027 1885.8954 1885.8958 -0.21 726 - 740 1 K.GYNAQEYYDRIPELR.H
1889.9683 1888.9610 1889.0007 -21.00 623 - 640 0 K.LITAIGDVVNHDPVVGDR.L
2007.1241 2006.1168 2006.1200 -1.60 294 - 310 1 R.LKQEYFVVAATLQDIIR.R
2044.0458 2043.0385 2043.0789 -19.76 553 - 569 1 R.EYKVHINPNSLFDIQVK.R
2118.1248 2117.1175 2117.1303 -6.03 334 - 352 0 K.VAIQLNDTHPSLAIPELMR.I
2159.2029 2158.1956 2158.1859 4.52 623 - 642 1 K.LITAIGDVVNHDPVVGDRLR.V
2307.1401 2306.1328 2306.1406 -3.36 216 - 235 0 K.WVDTQVVLAMPYDTPVPGYR.N
2323.1353 2322.1280 2322.1355 -3.21 216 - 235 0 K.WVDTQVVLAMPYDTPVPGYR.N + Oxidation (M)
2464.2288 2463.2215 2463.2506 -11.81 257 - 278 1 K.DFNVGGYIQAVLDRNLAENISR.V
2466.2324 2465.2251 2465.2339 -3.57 249 - 270 1 K.APNDFNLKDFNVGGYIQAVLDR.N
No match to: 872.4761, 893.4103, 1068.5479, 1086.5554, 1115.5763, 1117.5605, 1174.6033, 1261.7012, 1271.6656, 1277.6423, 1320.6438, 1372.7607, 1381.6793,
1386.7540, 1456.7322, 1473.8547, 1557.8268, 1584.8268, 1672.8704, 1716.8770, 1739.9244, 1744.8021, 1792.7952, 1808.7908, 1868.9391, 1869.9556, 1914.9526,
1943.9541, 2028.0472, 2059.0444, 2184.1106, 2225.1584, 2259.1499, 2371.2534, 3153.5139
10. gi|6730143 Mass: 96157 Score: 222 Expect: 6.7e-017 Matches: 28
Chain A, Identification And Structural Characterization Of A Novel Allosteric Binding Site Of Glycogen Phosphorylase B
Observed Mr(expt) Mr(calc) ppm Start End Miss Peptide
893.4103 892.4030 892.4079 -5.46 173 - 179 0 R.YGNPWEK.A
963.4651 962.4578 962.4604 -2.63 223 - 230 0 R.NNVVNTMR.L + Oxidation (M)
994.5660 993.5587 993.5760 -17.38 22 - 29 0 R.HLHFTLVK.D
1053.5698 1052.5625 1052.5614 1.04 820 - 828 1 R.QRLPAPDEK.I
1117.5605 1116.5532 1116.5564 -2.83 402 - 412 0 R.VAAAFPGDVDR.L
1145.5627 1144.5554 1144.5553 0.11 149 - 157 0 R.YEFGIFNQK.I
1262.6217 1261.6144 1261.5867 22.0 761 - 770 0 K.VFADYEEYVK.C
1386.7540 1385.7467 1385.7415 3.74 402 - 414 1 R.VAAAFPGDVDRLR.R
1426.7781 1425.7708 1425.7728 -1.39 387 - 397 0 R.HLQIIYEINQR.F
1442.6910 1441.6837 1441.6878 -2.81 266 - 277 0 R.VLYPNDNFFEGK.E
1473.8547 1472.8474 1472.8504 -1.99 375 - 386 0 R.WPVHLLETLLPR.H
1537.7914 1536.7841 1536.7500 22.2 759 - 770 1 R.FKVFADYEEYVK.C
1566.7914 1565.7841 1565.7838 0.20 244 - 257 0 K.DFNVGGYIQAVLDR.N
1689.8866 1688.8793 1688.8787 0.36 180 - 193 0 K.ARPEFTLPVHFYGR.V
1756.9471 1755.9398 1755.9454 -3.17 564 - 577 0 R.QLLNCLHVITLYNR.I
1835.8369 1834.8296 1834.8196 5.47 761 - 774 1 K.VFADYEEYVKCQER.V
1840.9187 1839.9114 1839.9155 -2.23 266 - 280 1 R.VLYPNDNFFEGKELR.L
1874.9188 1873.9115 1873.8992 6.56 359 - 374 0 K.TCAYTNHTVIPEALER.W
1886.9027 1885.8954 1885.8958 -0.21 713 - 727 1 R.GYNAQEYYDRIPELR.Q
1889.9683 1888.9610 1889.0007 -21.00 610 - 627 0 K.LITAIGDVVNHDPVVGDR.L
2007.1241 2006.1168 2006.1200 -1.60 281 - 297 1 R.LKQEYFVVAATLQDIIR.R
2118.1248 2117.1175 2117.1303 -6.03 321 - 339 0 K.VAIQLNDTHPSLAIPELMR.V
2159.2029 2158.1956 2158.1859 4.52 610 - 629 1 K.LITAIGDVVNHDPVVGDRLR.V
2307.1401 2306.1328 2306.1406 -3.36 203 - 222 0 K.WVDTQVVLAMPYDTPVPGYR.N
2323.1353 2322.1280 2322.1355 -3.21 203 - 222 0 K.WVDTQVVLAMPYDTPVPGYR.N + Oxidation (M)
2464.2288 2463.2215 2463.2506 -11.81 244 - 265 1 K.DFNVGGYIQAVLDRNLAENISR.V
2466.2324 2465.2251 2465.2339 -3.57 236 - 257 1 K.APNDFNLKDFNVGGYIQAVLDR.N
3153.5139 3152.5066 3152.5965 -28.49 312 - 339 1 R.TNFDAFPDKVAIQLNDTHPSLAIPELMR.V
No match to: 872.4761, 1068.5479, 1086.5554, 1115.5763, 1133.6079, 1174.6033, 1261.7012, 1271.6656, 1277.6423, 1320.6438, 1372.7607, 1381.6793, 1456.7322,
1534.7764, 1536.7903, 1557.8268, 1561.7968, 1584.8268, 1623.8685, 1662.8694, 1672.8704, 1716.8770, 1739.9244, 1744.8021, 1792.7952, 1808.7908, 1868.9391,
1869.9556, 1914.9526, 1943.9541, 2028.0472, 2044.0458, 2059.0444, 2184.1106, 2225.1584, 2259.1499, 2371.2534
11. gi|134104402 Mass: 95535 Score: 220 Expect: 1.1e-016 Matches: 28
Chain A, Structure Of Rabbit Muscle Glycogen Phosphorylase In Complex With Ligand
Observed Mr(expt) Mr(calc) ppm Start End Miss Peptide
893.4103 892.4030 892.4079 -5.46 174 - 180 0 R.YGNPWEK.A
963.4651 962.4578 962.4604 -2.63 224 - 231 0 R.NNVVNTMR.L + Oxidation (M)
994.5660 993.5587 993.5760 -17.38 23 - 30 0 R.HLHFTLVK.D
1053.5698 1052.5625 1052.5655 -2.79 631 - 638 0 R.VIFLENYR.V
1117.5605 1116.5532 1116.5564 -2.83 403 - 413 0 R.VAAAFPGDVDR.L
1145.5627 1144.5554 1144.5553 0.11 150 - 158 0 R.YEFGIFNQK.I
1262.6217 1261.6144 1261.5867 22.0 762 - 771 0 K.VFADYEEYVK.C
1386.7540 1385.7467 1385.7415 3.74 403 - 415 1 R.VAAAFPGDVDRLR.R
1426.7781 1425.7708 1425.7728 -1.39 388 - 398 0 R.HLQIIYEINQR.F
1442.6910 1441.6837 1441.6878 -2.81 267 - 278 0 R.VLYPNDNFFEGK.E
1473.8547 1472.8474 1472.8504 -1.99 376 - 387 0 R.WPVHLLETLLPR.H
1537.7914 1536.7841 1536.7500 22.2 760 - 771 1 R.FKVFADYEEYVK.C
1566.7914 1565.7841 1565.7838 0.20 245 - 258 0 K.DFNVGGYIQAVLDR.N
1689.8866 1688.8793 1688.8787 0.36 181 - 194 0 K.ARPEFTLPVHFYGR.V
1756.9471 1755.9398 1755.9454 -3.17 565 - 578 0 R.QLLNCLHVITLYNR.I
1835.8369 1834.8296 1834.8196 5.47 762 - 775 1 K.VFADYEEYVKCQER.V
1840.9187 1839.9114 1839.9155 -2.23 267 - 281 1 R.VLYPNDNFFEGKELR.L

1874.9188 1873.9115 1873.8992 6.56 360 - 375 0 K.TCAYTNHTVLPEALER.W
1886.9027 1885.8954 1885.8958 -0.21 714 - 728 1 R.GYNAQEYYDRIPELR.Q
1889.9683 1888.9610 1889.0007 -21.00 611 - 628 0 K.LITAIGDVVNHDPVVGDR.L
2007.1241 2006.1168 2006.1200 -1.60 282 - 298 1 R.LKQEYFVVAATLQDIIR.R
2118.1248 2117.1175 2117.1303 -6.03 322 - 340 0 K.VAIQLNDTHPSLAIPELMR.V
2159.2029 2158.1956 2158.1859 4.52 611 - 630 1 K.LITAIGDVVNHDPVVGDRLR.V
2307.1401 2306.1328 2306.1406 -3.36 204 - 223 0 K.WVDTQVVLAMPYDTPVPGYR.N
2323.1353 2322.1280 2322.1355 -3.21 204 - 223 0 K.WVDTQVVLAMPYDTPVPGYR.N + Oxidation (M)
2464.2288 2463.2215 2463.2506 -11.81 245 - 266 1 K.DFNVGGYIQAVLDRNLAENISR.V
2466.2324 2465.2251 2465.2339 -3.57 237 - 258 1 K.APNDFNLKDFNVGGYIQAVLDR.N
3153.5139 3152.5066 3152.5965 -28.49 313 - 340 1 R.TNFDAFPDKVAIQLNDTHPSLAIPELMR.V
No match to: 872.4761, 1068.5479, 1086.5554, 1115.5763, 1133.6079, 1174.6033, 1261.7012, 1271.6656, 1277.6423, 1320.6438, 1372.7607, 1381.6793, 1456.7322,
1534.7764, 1536.7903, 1557.8268, 1561.7968, 1584.8268, 1623.8685, 1662.8694, 1672.8704, 1716.8770, 1739.9244, 1744.8021, 1792.7952, 1808.7908, 1868.9391,
1869.9556, 1914.9526, 1943.9541, 2028.0472, 2044.0458, 2059.0444, 2184.1106, 2225.1584, 2259.1499, 2371.2534
12. gi|134104414 Mass: 95606 Score: 220 Expect: 1.1e-016 Matches: 28
Chain A, Structure Of Rabbit Muscle Glycogen Phosphorylase In Complex With Thienopyrrole
Observed Mr(expt) Mr(calc) ppm Start End Miss Peptide
893.4103 892.4030 892.4079 -5.46 174 - 180 0 R.YGNPWEK.A
963.4651 962.4578 962.4604 -2.63 224 - 231 0 R.NNVVNTMR.L + Oxidation (M)
994.5660 993.5587 993.5760 -17.38 23 - 30 0 R.HLHFTLVK.D
1053.5698 1052.5625 1052.5655 -2.79 631 - 638 0 R.VIFLENYR.V
1117.5605 1116.5532 1116.5564 -2.83 403 - 413 0 R.VAAAFPGDVDR.L
1145.5627 1144.5554 1144.5553 0.11 150 - 158 0 R.YEFGIFNQK.I
1262.6217 1261.6144 1261.5867 22.0 762 - 771 0 K.VFADYEEYVK.C
1386.7540 1385.7467 1385.7415 3.74 403 - 415 1 R.VAAAFPGDVDRLR.R
1426.7781 1425.7708 1425.7728 -1.39 388 - 398 0 R.HLQIIYEINQR.F
1442.6910 1441.6837 1441.6878 -2.81 267 - 278 0 R.VLYPNDNFFEGK.E
1473.8547 1472.8474 1472.8504 -1.99 376 - 387 0 R.WPVHLLETLLPR.H
1537.7914 1536.7841 1536.7500 22.2 760 - 771 1 R.FKVFADYEEYVK.C
1566.7914 1565.7841 1565.7838 0.20 245 - 258 0 K.DFNVGGYIQAVLDR.N
1689.8866 1688.8793 1688.8787 0.36 181 - 194 0 K.ARPEFTLPVHFYGR.V
1756.9471 1755.9398 1755.9454 -3.17 565 - 578 0 R.QLLNCLHVITLYNR.I
1835.8369 1834.8296 1834.8196 5.47 762 - 775 1 K.VFADYEEYVKCQER.V
1840.9187 1839.9114 1839.9155 -2.23 267 - 281 1 R.VLYPNDNFFEGKELR.L
1874.9188 1873.9115 1873.8992 6.56 360 - 375 0 K.TCAYTNHTVLPEALER.W
1886.9027 1885.8954 1885.8958 -0.21 714 - 728 1 R.GYNAQEYYDRIPELR.Q
1889.9683 1888.9610 1889.0007 -21.00 611 - 628 0 K.LITAIGDVVNHDPVVGDR.L
2007.1241 2006.1168 2006.1200 -1.60 282 - 298 1 R.LKQEYFVVAATLQDIIR.R
2118.1248 2117.1175 2117.1303 -6.03 322 - 340 0 K.VAIQLNDTHPSLAIPELMR.V
2159.2029 2158.1956 2158.1859 4.52 611 - 630 1 K.LITAIGDVVNHDPVVGDRLR.V
2307.1401 2306.1328 2306.1406 -3.36 204 - 223 0 K.WVDTQVVLAMPYDTPVPGYR.N
2323.1353 2322.1280 2322.1355 -3.21 204 - 223 0 K.WVDTQVVLAMPYDTPVPGYR.N + Oxidation (M)
2464.2288 2463.2215 2463.2506 -11.81 245 - 266 1 K.DFNVGGYIQAVLDRNLAENISR.V
2466.2324 2465.2251 2465.2339 -3.57 237 - 258 1 K.APNDFNLKDFNVGGYIQAVLDR.N
3153.5139 3152.5066 3152.5965 -28.49 313 - 340 1 R.TNFDAFPDKVAIQLNDTHPSLAIPELMR.V
No match to: 872.4761, 1068.5479, 1086.5554, 1115.5763, 1133.6079, 1174.6033, 1261.7012, 1271.6656, 1277.6423, 1320.6438, 1372.7607, 1381.6793, 1456.7322,
1534.7764, 1536.7903, 1557.8268, 1561.7968, 1584.8268, 1623.8685, 1662.8694, 1672.8704, 1716.8770, 1739.9244, 1744.8021, 1792.7952, 1808.7908, 1868.9391,
1869.9556, 1914.9526, 1943.9541, 2028.0472, 2044.0458, 2059.0444, 2184.1106, 2225.1584, 2259.1499, 2371.2534
13. gi|316983251 Mass: 97506 Score: 220 Expect: 1.1e-016 Matches: 28
Chain A, The Binding Of Beta-D-Glucopyranosyl-Thiosemicarbazone Derivatives To Glycogen Phosphorylase: A New Class Of Inhibitors
Observed Mr(expt) Mr(calc) ppm Start End Miss Peptide
893.4103 892.4030 892.4079 -5.46 184 - 190 0 R.YGNPWEK.A
963.4651 962.4578 962.4604 -2.63 234 - 241 0 R.NNVVNTMR.L + Oxidation (M)
994.5660 993.5587 993.5760 -17.38 33 - 40 0 R.HLHFTLVK.D
1053.5698 1052.5625 1052.5614 1.04 831 - 839 1 R.QRLPAPDEK.I
1117.5605 1116.5532 1116.5564 -2.83 413 - 423 0 R.VAAAFPGDVDR.L
1145.5627 1144.5554 1144.5553 0.11 160 - 168 0 R.YEFGIFNQK.I
1262.6217 1261.6144 1261.5867 22.0 772 - 781 0 K.VFADYEEYVK.C
1386.7540 1385.7467 1385.7415 3.74 413 - 425 1 R.VAAAFPGDVDRLR.R
1426.7781 1425.7708 1425.7728 -1.39 398 - 408 0 R.HLQIIYEINQR.F
1442.6910 1441.6837 1441.6878 -2.81 277 - 288 0 R.VLYPNDNFFEGK.E
1473.8547 1472.8474 1472.8504 -1.99 386 - 397 0 R.WPVHLLETLLPR.H
1537.7914 1536.7841 1536.7500 22.2 770 - 781 1 R.FKVFADYEEYVK.C
1566.7914 1565.7841 1565.7838 0.20 255 - 268 0 K.DFNVGGYIQAVLDR.N
1689.8866 1688.8793 1688.8787 0.36 191 - 204 0 K.ARPEFTLPVHFYGR.V
1756.9471 1755.9398 1755.9454 -3.17 575 - 588 0 R.QLLNCLHVITLYNR.I
1835.8369 1834.8296 1834.8196 5.47 772 - 785 1 K.VFADYEEYVKCQER.V
1840.9187 1839.9114 1839.9155 -2.23 277 - 291 1 R.VLYPNDNFFEGKELR.L
1874.9188 1873.9115 1873.8992 6.56 370 - 385 0 K.TCAYTNHTVLPEALER.W
1886.9027 1885.8954 1885.8958 -0.21 724 - 738 1 R.GYNAQEYYDRIPELR.Q
1889.9683 1888.9610 1889.0007 -21.00 621 - 638 0 K.LITAIGDVVNHDPVVGDR.L
2007.1241 2006.1168 2006.1200 -1.60 292 - 308 1 R.LKQEYFVVAATLQDIIR.R
2118.1248 2117.1175 2117.1303 -6.03 332 - 350 0 K.VAIQLNDTHPSLAIPELMR.V
2159.2029 2158.1956 2158.1859 4.52 621 - 640 1 K.LITAIGDVVNHDPVVGDRLR.V
2307.1401 2306.1328 2306.1406 -3.36 214 - 233 0 K.WVDTQVVLAMPYDTPVPGYR.N
2323.1353 2322.1280 2322.1355 -3.21 214 - 233 0 K.WVDTQVVLAMPYDTPVPGYR.N + Oxidation (M)
2464.2288 2463.2215 2463.2506 -11.81 255 - 276 1 K.DFNVGGYIQAVLDRNLAENISR.V
2466.2324 2465.2251 2465.2339 -3.57 247 - 268 1 K.APNDFNLKDFNVGGYIQAVLDR.N
3153.5139 3152.5066 3152.5965 -28.49 323 - 350 1 R.TNFDAFPDKVAIQLNDTHPSLAIPELMR.V
No match to: 872.4761, 1068.5479, 1086.5554, 1115.5763, 1133.6079, 1174.6033, 1261.7012, 1271.6656, 1277.6423, 1320.6438, 1372.7607, 1381.6793, 1456.7322,
1534.7764, 1536.7903, 1557.8268, 1561.7968, 1584.8268, 1623.8685, 1662.8694, 1672.8704, 1716.8770, 1739.9244, 1744.8021, 1792.7952, 1808.7908, 1868.9391,
1869.9556, 1914.9526, 1943.9541, 2028.0472, 2044.0458, 2059.0444, 2184.1106, 2225.1584, 2259.1499, 2371.2534
14. gi|194388822 Mass: 96356 Score: 218 Expect: 1.7e-016 Matches: 29
unnamed protein product [Homo sapiens]
Observed Mr(expt) Mr(calc) ppm Start End Miss Peptide
893.4103 892.4030 892.4079 -5.46 167 - 173 0 R.YGNPWEK.A

963.4651 962.4578 962.4604 -2.63 217 - 224 0 R.NNVVNTMR.L + Oxidation (M)
994.5660 993.5587 993.5760 -17.38 35 - 42 0 R.HLHFTLVK.D
1053.5698 1052.5625 1052.5655 -2.79 624 - 631 0 R.VIFLENYR.V
1117.5605 1116.5532 1116.5564 -2.83 396 - 406 0 R.VAAAFPGDVDR.L
1133.6079 1132.6006 1132.6029 -2.03 575 - 583 1 R.EPNKFFVPR.T
1145.5627 1144.5554 1144.5553 0.11 143 - 151 0 R.YEFGIFNQK.I
1262.6217 1261.6144 1261.5867 22.0 755 - 764 0 K.VFADYEDYIK.C
1386.7540 1385.7467 1385.7415 3.74 396 - 408 1 R.VAAAFPGDVDRLR.R
1426.7781 1425.7708 1425.7728 -1.39 381 - 391 0 R.HLQIIYEINQR.F
1442.6910 1441.6837 1441.6878 -2.81 260 - 271 0 R.VLYPNDNFFEGK.E
1473.8547 1472.8474 1472.8504 -1.99 369 - 380 0 R.WPVHLLETLLPR.H
1537.7914 1536.7841 1536.7500 22.2 753 - 764 1 R.FKVFADYEDYIK.C
1566.7914 1565.7841 1565.7838 0.20 238 - 251 0 K.DFNVGGYIQAVLDR.N
1623.8685 1622.8612 1622.8780 -10.36 537 - 550 0 K.VHINPNSLFDIQVK.R
1756.9471 1755.9398 1755.9454 -3.17 558 - 571 0 R.QLLNCLHVITLYNR.I
1792.7952 1791.7879 1791.7886 -0.39 152 - 166 0 K.ISGGWQMEEADDWLR.Y
1808.7908 1807.7835 1807.7835 -0.01 152 - 166 0 K.ISGGWQMEEADDWLR.Y + Oxidation (M)
1840.9187 1839.9114 1839.9155 -2.23 260 - 274 1 R.VLYPNDNFFEGKELR.L
1874.9188 1873.9115 1873.8992 6.56 353 - 368 0 R.TCAYTNHTVLPEALER.W
1886.9027 1885.8954 1885.8958 -0.21 707 - 721 1 R.GYNAQEYYDRIPELR.Q
2007.1241 2006.1168 2006.1200 -1.60 275 - 291 1 R.LKQEYFVVAATLQDIIR.R
2044.0458 2043.0385 2043.0789 -19.76 534 - 550 1 R.EYKVHINPNSLFDIQVK.R
2118.1248 2117.1175 2117.1303 -6.03 315 - 333 0 K.VAIQLNDTHPSLAIPELMR.I
2307.1401 2306.1328 2306.1406 -3.36 197 - 216 0 K.WVDTQVVLAMPYDTPVPGYR.N
2323.1353 2322.1280 2322.1355 -3.21 197 - 216 0 K.WVDTQVVLAMPYDTPVPGYR.N + Oxidation (M)
2464.2288 2463.2215 2463.2506 -11.81 238 - 259 1 K.DFNVGGYIQAVLDRNLAENISR.V
2466.2324 2465.2251 2465.2339 -3.57 230 - 251 1 K.APNDFNLKDFNVGGYIQAVLDR.N
3153.5139 3152.5066 3152.5965 -28.49 306 - 333 1 R.TNFDAFPDKVAIQLNDTHPSLAIPELMR.I
No match to: 872.4761, 1068.5479, 1086.5554, 1115.5763, 1174.6033, 1261.7012, 1271.6656, 1277.6423, 1320.6438, 1372.7607, 1381.6793, 1456.7322, 1534.7764,
1536.7903, 1557.8268, 1561.7968, 1584.8268, 1662.8694, 1672.8704, 1689.8866, 1716.8770, 1739.9244, 1744.8021, 1835.8369, 1868.9391, 1869.9556, 1889.9683,
1914.9526, 1943.9541, 2028.0472, 2059.0444, 2159.2029, 2184.1106, 2225.1584, 2259.1499, 2371.2534
15. gi|442605 Mass: 95987 Score: 218 Expect: 1.7e-016 Matches: 28
Chain A, Control Of Phosphorylase B Conformation By A Modified Cofactor: Crystallographic Studies On R-State Glycogen Phosphorylase Reconstituted With Pyridoxal 5'-D
Observed Mr(expt) Mr(calc) ppm Start End Miss Peptide
893.4103 892.4030 892.4079 -5.46 176 - 182 0 R.YGNPWEK.A
963.4651 962.4578 962.4604 -2.63 226 - 233 0 R.NNVVNTMR.L + Oxidation (M)
994.5660 993.5587 993.5760 -17.38 25 - 32 0 R.HLHFTLVK.D
1053.5698 1052.5625 1052.5655 -2.79 633 - 640 0 R.VIFLENYR.V
1117.5605 1116.5532 1116.5564 -2.83 405 - 415 0 R.VAAAFPGDVDR.L
1145.5627 1144.5554 1144.5553 0.11 152 - 160 0 R.YEFGIFNQK.I
1262.6217 1261.6144 1261.5867 22.0 764 - 773 0 K.VFADYEEYVK.C
1386.7540 1385.7467 1385.7415 3.74 405 - 417 1 R.VAAAFPGDVDRLR.R
1426.7781 1425.7708 1425.7728 -1.39 390 - 400 0 R.HLQIIYEINQR.F
1442.6910 1441.6837 1441.6878 -2.81 269 - 280 0 R.VLYPNDNFFEGK.E
1473.8547 1472.8474 1472.8504 -1.99 378 - 389 0 R.WPVHLLETLLPR.H
1537.7914 1536.7841 1536.7500 22.2 762 - 773 1 R.FKVFADYEEYVK.C
1566.7914 1565.7841 1565.7838 0.20 247 - 260 0 K.DFNVGGYIQAVLDR.N
1689.8866 1688.8793 1688.8787 0.36 183 - 196 0 K.ARPEFTLPVHFYGR.V
1756.9471 1755.9398 1755.9454 -3.17 567 - 580 0 R.QLLNCLHVITLYNR.I
1835.8369 1834.8296 1834.8196 5.47 764 - 777 1 K.VFADYEEYVKCQER.V
1840.9187 1839.9114 1839.9155 -2.23 269 - 283 1 R.VLYPNDNFFEGKELR.L
1874.9188 1873.9115 1873.8992 6.56 362 - 377 0 K.TCAYTNHTVIPEALER.W
1886.9027 1885.8954 1885.8958 -0.21 716 - 730 1 R.GYNAQEYYDRIPELR.Q
1889.9683 1888.9610 1889.0007 -21.00 613 - 630 0 K.LITAIGDVVNHDPVVGDR.L
2007.1241 2006.1168 2006.1200 -1.60 284 - 300 1 R.LKQEYFVVAATLQDIIR.R
2118.1248 2117.1175 2117.1303 -6.03 324 - 342 0 K.VAIQLNDTHPSLAIPELMR.V
2159.2029 2158.1956 2158.1859 4.52 613 - 632 1 K.LITAIGDVVNHDPVVGDRLR.V
2307.1401 2306.1328 2306.1406 -3.36 206 - 225 0 K.WVDTQVVLAMPYDTPVPGYR.N
2323.1353 2322.1280 2322.1355 -3.21 206 - 225 0 K.WVDTQVVLAMPYDTPVPGYR.N + Oxidation (M)
2464.2288 2463.2215 2463.2506 -11.81 247 - 268 1 K.DFNVGGYIQAVLDRNLAENISR.V
2466.2324 2465.2251 2465.2339 -3.57 239 - 260 1 K.APNDFNLKDFNVGGYIQAVLDR.N
3153.5139 3152.5066 3152.5965 -28.49 315 - 342 1 R.TNFDAFPDKVAIQLNDTHPSLAIPELMR.V
No match to: 872.4761, 1068.5479, 1086.5554, 1115.5763, 1133.6079, 1174.6033, 1261.7012, 1271.6656, 1277.6423, 1320.6438, 1372.7607, 1381.6793, 1456.7322,
1534.7764, 1536.7903, 1557.8268, 1561.7968, 1584.8268, 1623.8685, 1662.8694, 1672.8704, 1716.8770, 1739.9244, 1744.8021, 1792.7952, 1808.7908, 1868.9391,
1869.9556, 1914.9526, 1943.9541, 2028.0472, 2044.0458, 2059.0444, 2184.1106, 2225.1584, 2259.1499, 2371.2534
16. gi|297688261 Mass: 96248 Score: 218 Expect: 1.7e-016 Matches: 29
PREDICTED: glycogen phosphorylase, muscle form-like isoform 2 [Pongo abelii]
Observed Mr(expt) Mr(calc) ppm Start End Miss Peptide
893.4103 892.4030 892.4079 -5.46 167 - 173 0 R.YGNPWEK.A
963.4651 962.4578 962.4604 -2.63 217 - 224 0 R.NNVVNTMR.L + Oxidation (M)
994.5660 993.5587 993.5760 -17.38 35 - 42 0 R.HLHFTLVK.D
1053.5698 1052.5625 1052.5655 -2.79 624 - 631 0 R.VIFLENYR.V
1117.5605 1116.5532 1116.5564 -2.83 396 - 406 0 R.VAAAFPGDVDR.L
1133.6079 1132.6006 1132.6029 -2.03 575 - 583 1 R.EPNKFFVPR.T
1145.5627 1144.5554 1144.5553 0.11 143 - 151 0 R.YEFGIFNQK.I
1262.6217 1261.6144 1261.5867 22.0 755 - 764 0 K.VFADYEDYIK.C
1386.7540 1385.7467 1385.7415 3.74 396 - 408 1 R.VAAAFPGDVDRLR.R
1426.7781 1425.7708 1425.7728 -1.39 381 - 391 0 R.HLQIIYEINQR.F
1442.6910 1441.6837 1441.6878 -2.81 260 - 271 0 R.VLYPNDNFFEGK.E
1473.8547 1472.8474 1472.8504 -1.99 369 - 380 0 R.WPVHLLETLLPR.H
1537.7914 1536.7841 1536.7500 22.2 753 - 764 1 R.FKVFADYEDYIK.C
1566.7914 1565.7841 1565.7838 0.20 238 - 251 0 K.DFNVGGYIQAVLDR.N
1623.8685 1622.8612 1622.8780 -10.36 537 - 550 0 K.VHINPNSLFDIQVK.R
1756.9471 1755.9398 1755.9454 -3.17 558 - 571 0 R.QLLNCLHVITLYNR.I
1792.7952 1791.7879 1791.7886 -0.39 152 - 166 0 K.ISGGWQMEEADDWLR.Y
1808.7908 1807.7835 1807.7835 -0.01 152 - 166 0 K.ISGGWQMEEADDWLR.Y + Oxidation (M)
1840.9187 1839.9114 1839.9155 -2.23 260 - 274 1 R.VLYPNDNFFEGKELR.L

1874.9188 1873.9115 1873.8992 6.56 353 - 368 0 R.TCAYTNHTVLPEALER.W
1886.9027 1885.8954 1885.8958 -0.21 707 - 721 1 R.GYNAQEYYDRIPELR.Q
2007.1241 2006.1168 2006.1200 -1.60 275 - 291 1 R.LKQEYFVVAATLQDIIR.R
2044.0458 2043.0385 2043.0789 -19.76 534 - 550 1 R.EYKVHINPNSLFDIQVK.R
2118.1248 2117.1175 2117.1303 -6.03 315 - 333 0 K.VAIQLNDTHPSLAIPELMR.I
2307.1401 2306.1328 2306.1406 -3.36 197 - 216 0 K.WVDTQVVLAMPYDTPVPGYR.N
2323.1353 2322.1280 2322.1355 -3.21 197 - 216 0 K.WVDTQVVLAMPYDTPVPGYR.N + Oxidation (M)
2464.2288 2463.2215 2463.2506 -11.81 238 - 259 1 K.DFNVGGYIQAVLDRNLAENISR.V
2466.2324 2465.2251 2465.2339 -3.57 230 - 251 1 K.APNDFNLKDFNVGGYIQAVLDR.N
3153.5139 3152.5066 3152.5965 -28.49 306 - 333 1 R.TNFDAFPDKVAIQLNDTHPSLAIPELMR.I
No match to: 872.4761, 1068.5479, 1086.5554, 1115.5763, 1174.6033, 1261.7012, 1271.6656, 1277.6423, 1320.6438, 1372.7607, 1381.6793, 1456.7322, 1534.7764,
1536.7903, 1557.8268, 1561.7968, 1584.8268, 1662.8694, 1672.8704, 1689.8866, 1716.8770, 1739.9244, 1744.8021, 1835.8369, 1868.9391, 1869.9556, 1889.9683,
1914.9526, 1943.9541, 2028.0472, 2059.0444, 2159.2029, 2184.1106, 2225.1584, 2259.1499, 2371.2534
17. gi|66361339 Mass: 97593 Score: 218 Expect: 1.7e-016 Matches: 28
Chain A, Glycogen Phosphorylase Amp Site Inhibitor Complex
Observed Mr(expt) Mr(calc) ppm Start End Miss Peptide
893.4103 892.4030 892.4079 -5.46 185 - 191 0 R.YGNPWEK.A
963.4651 962.4578 962.4604 -2.63 235 - 242 0 R.NNVVNTMR.L + Oxidation (M)
994.5660 993.5587 993.5760 -17.38 34 - 41 0 R.HLHFTLVK.D
1053.5698 1052.5625 1052.5614 1.04 832 - 840 1 R.QRLPAPDEK.I
1117.5605 1116.5532 1116.5564 -2.83 414 - 424 0 R.VAAAFPGDVDR.L
1145.5627 1144.5554 1144.5553 0.11 161 - 169 0 R.YEFGIFNQK.I
1262.6217 1261.6144 1261.5867 22.0 773 - 782 0 K.VFADYEEYVK.C
1386.7540 1385.7467 1385.7415 3.74 414 - 426 1 R.VAAAFPGDVDRLR.R
1426.7781 1425.7708 1425.7728 -1.39 399 - 409 0 R.HLQIIYEINQR.F
1442.6910 1441.6837 1441.6878 -2.81 278 - 289 0 R.VLYPNDNFFEGK.E
1473.8547 1472.8474 1472.8504 -1.99 387 - 398 0 R.WPVHLLETLLPR.H
1537.7914 1536.7841 1536.7500 22.2 771 - 782 1 R.FKVFADYEEYVK.C
1566.7914 1565.7841 1565.7838 0.20 256 - 269 0 K.DFNVGGYIQAVLDR.N
1689.8866 1688.8793 1688.8787 0.36 192 - 205 0 K.ARPEFTLPVHFYGR.V
1756.9471 1755.9398 1755.9454 -3.17 576 - 589 0 R.QLLNCLHVITLYNR.I
1835.8369 1834.8296 1834.8196 5.47 773 - 786 1 K.VFADYEEYVKCQER.V
1840.9187 1839.9114 1839.9155 -2.23 278 - 292 1 R.VLYPNDNFFEGKELR.L
1874.9188 1873.9115 1873.8992 6.56 371 - 386 0 K.TCAYTNHTVIPEALER.W
1886.9027 1885.8954 1885.8958 -0.21 725 - 739 1 R.GYNAQEYYDRIPELR.Q
1889.9683 1888.9610 1889.0007 -21.00 622 - 639 0 K.LITAIGDVVNHDPVVGDR.L
2007.1241 2006.1168 2006.1200 -1.60 293 - 309 1 R.LKQEYFVVAATLQDIIR.R
2118.1248 2117.1175 2117.1303 -6.03 333 - 351 0 K.VAIQLNDTHPSLAIPELMR.V
2159.2029 2158.1956 2158.1859 4.52 622 - 641 1 K.LITAIGDVVNHDPVVGDRLR.V
2307.1401 2306.1328 2306.1406 -3.36 215 - 234 0 K.WVDTQVVLAMPYDTPVPGYR.N
2323.1353 2322.1280 2322.1355 -3.21 215 - 234 0 K.WVDTQVVLAMPYDTPVPGYR.N + Oxidation (M)
2464.2288 2463.2215 2463.2506 -11.81 256 - 277 1 K.DFNVGGYIQAVLDRNLAENISR.V
2466.2324 2465.2251 2465.2339 -3.57 248 - 269 1 K.APNDFNLKDFNVGGYIQAVLDR.N
3153.5139 3152.5066 3152.5965 -28.49 324 - 351 1 R.TNFDAFPDKVAIQLNDTHPSLAIPELMR.V
No match to: 872.4761, 1068.5479, 1086.5554, 1115.5763, 1133.6079, 1174.6033, 1261.7012, 1271.6656, 1277.6423, 1320.6438, 1372.7607, 1381.6793, 1456.7322,
1534.7764, 1536.7903, 1557.8268, 1561.7968, 1584.8268, 1623.8685, 1662.8694, 1672.8704, 1716.8770, 1739.9244, 1744.8021, 1792.7952, 1808.7908, 1868.9391,
1869.9556, 1914.9526, 1943.9541, 2028.0472, 2044.0458, 2059.0444, 2184.1106, 2225.1584, 2259.1499, 2371.2534
18. gi|126030531 Mass: 97593 Score: 218 Expect: 1.7e-016 Matches: 28
Chain A, The Crystal Structure Of The Glycogen Phosphorylase B- 1ab Complex
Observed Mr(expt) Mr(calc) ppm Start End Miss Peptide
893.4103 892.4030 892.4079 -5.46 185 - 191 0 R.YGNPWEK.A
963.4651 962.4578 962.4604 -2.63 235 - 242 0 R.NNVVNTMR.L + Oxidation (M)
994.5660 993.5587 993.5760 -17.38 34 - 41 0 R.HLHFTLVK.D
1053.5698 1052.5625 1052.5614 1.04 832 - 840 1 R.QRLPAPDEK.I
1117.5605 1116.5532 1116.5564 -2.83 414 - 424 0 R.VAAAFPGDVDR.L
1145.5627 1144.5554 1144.5553 0.11 161 - 169 0 R.YEFGIFNQK.I
1262.6217 1261.6144 1261.5867 22.0 773 - 782 0 K.VFADYEEYVK.C
1386.7540 1385.7467 1385.7415 3.74 414 - 426 1 R.VAAAFPGDVDRLR.R
1426.7781 1425.7708 1425.7728 -1.39 399 - 409 0 R.HLQIIYEINQR.F
1442.6910 1441.6837 1441.6878 -2.81 278 - 289 0 R.VLYPNDNFFEGK.E
1473.8547 1472.8474 1472.8504 -1.99 387 - 398 0 R.WPVHLLETLLPR.H
1537.7914 1536.7841 1536.7500 22.2 771 - 782 1 R.FKVFADYEEYVK.C
1566.7914 1565.7841 1565.7838 0.20 256 - 269 0 K.DFNVGGYIQAVLDR.N
1689.8866 1688.8793 1688.8787 0.36 192 - 205 0 K.ARPEFTLPVHFYGR.V
1756.9471 1755.9398 1755.9454 -3.17 576 - 589 0 R.QLLNCLHVITLYNR.I
1835.8369 1834.8296 1834.8196 5.47 773 - 786 1 K.VFADYEEYVKCQER.V
1840.9187 1839.9114 1839.9155 -2.23 278 - 292 1 R.VLYPNDNFFEGKELR.L
1874.9188 1873.9115 1873.8992 6.56 371 - 386 0 K.TCAYTNHTVLPEALER.W
1886.9027 1885.8954 1885.8958 -0.21 725 - 739 1 R.GYNAQEYYDRIPELR.Q
1889.9683 1888.9610 1889.0007 -21.00 622 - 639 0 K.LITAIGDVVNHDPVVGDR.L
2007.1241 2006.1168 2006.1200 -1.60 293 - 309 1 R.LKQEYFVVAATLQDIIR.R
2118.1248 2117.1175 2117.1303 -6.03 333 - 351 0 K.VAIQLNDTHPSLAIPELMR.V
2159.2029 2158.1956 2158.1859 4.52 622 - 641 1 K.LITAIGDVVNHDPVVGDRLR.V
2307.1401 2306.1328 2306.1406 -3.36 215 - 234 0 K.WVDTQVVLAMPYDTPVPGYR.N
2323.1353 2322.1280 2322.1355 -3.21 215 - 234 0 K.WVDTQVVLAMPYDTPVPGYR.N + Oxidation (M)
2464.2288 2463.2215 2463.2506 -11.81 256 - 277 1 K.DFNVGGYIQAVLDRNLAENISR.V
2466.2324 2465.2251 2465.2339 -3.57 248 - 269 1 K.APNDFNLKDFNVGGYIQAVLDR.N
3153.5139 3152.5066 3152.5965 -28.49 324 - 351 1 R.TNFDAFPDKVAIQLNDTHPSLAIPELMR.V
No match to: 872.4761, 1068.5479, 1086.5554, 1115.5763, 1133.6079, 1174.6033, 1261.7012, 1271.6656, 1277.6423, 1320.6438, 1372.7607, 1381.6793, 1456.7322,
1534.7764, 1536.7903, 1557.8268, 1561.7968, 1584.8268, 1623.8685, 1662.8694, 1672.8704, 1716.8770, 1739.9244, 1744.8021, 1792.7952, 1808.7908, 1868.9391,
1869.9556, 1914.9526, 1943.9541, 2028.0472, 2044.0458, 2059.0444, 2184.1106, 2225.1584, 2259.1499, 2371.2534
19. gi|93278542 Mass: 97494 Score: 218 Expect: 1.7e-016 Matches: 29
Chain A, Crystal Structure Of Human Muscle Glycogen Phosphorylase A With Amp And Glucose
Observed Mr(expt) Mr(calc) ppm Start End Miss Peptide
893.4103 892.4030 892.4079 -5.46 186 - 192 0 R.YGNPWEK.A
963.4651 962.4578 962.4604 -2.63 236 - 243 0 R.NNVVNTMR.L + Oxidation (M)

994.5660 993.5587 993.5760 -17.38 35 - 42 0 R.HLHFTLVK.D
1053.5698 1052.5625 1052.5655 -2.79 643 - 650 0 R.VIFLENYR.V
1117.5605 1116.5532 1116.5564 -2.83 415 - 425 0 R.VAAAFPGDVDR.L
1133.6079 1132.6006 1132.6029 -2.03 594 - 602 1 R.EPNKFFVPR.T
1145.5627 1144.5554 1144.5553 0.11 162 - 170 0 R.YEFGIFNQK.I
1262.6217 1261.6144 1261.5867 22.0 774 - 783 0 K.VFADYEDYIK.C
1386.7540 1385.7467 1385.7415 3.74 415 - 427 1 R.VAAAFPGDVDRLR.R
1426.7781 1425.7708 1425.7728 -1.39 400 - 410 0 R.HLQIIYEINQR.F
1442.6910 1441.6837 1441.6878 -2.81 279 - 290 0 R.VLYPNDNFFEGK.E
1473.8547 1472.8474 1472.8504 -1.99 388 - 399 0 R.WPVHLLETLLPR.H
1537.7914 1536.7841 1536.7500 22.2 772 - 783 1 R.FKVFADYEDYIK.C
1566.7914 1565.7841 1565.7838 0.20 257 - 270 0 K.DFNVGGYIQAVLDR.N
1623.8685 1622.8612 1622.8780 -10.36 556 - 569 0 K.VHINPNSLFDIQVK.R
1756.9471 1755.9398 1755.9454 -3.17 577 - 590 0 R.QLLNCLHVITLYNR.I
1792.7952 1791.7879 1791.7886 -0.39 171 - 185 0 K.ISGGWQMEEADDWLR.Y
1808.7908 1807.7835 1807.7835 -0.01 171 - 185 0 K.ISGGWQMEEADDWLR.Y + Oxidation (M)
1840.9187 1839.9114 1839.9155 -2.23 279 - 293 1 R.VLYPNDNFFEGKELR.L
1874.9188 1873.9115 1873.8992 6.56 372 - 387 0 R.TCAYTNHTVLPEALER.W
1886.9027 1885.8954 1885.8958 -0.21 726 - 740 1 R.GYNAQEYYDRIPELR.Q
2007.1241 2006.1168 2006.1200 -1.60 294 - 310 1 R.LKQEYFVVAATLQDIIR.R
2044.0458 2043.0385 2043.0789 -19.76 553 - 569 1 R.EYKVHINPNSLFDIQVK.R
2118.1248 2117.1175 2117.1303 -6.03 334 - 352 0 K.VAIQLNDTHPSLAIPELMR.I
2307.1401 2306.1328 2306.1406 -3.36 216 - 235 0 K.WVDTQVVLAMPYDTPVPGYR.N
2323.1353 2322.1280 2322.1355 -3.21 216 - 235 0 K.WVDTQVVLAMPYDTPVPGYR.N + Oxidation (M)
2464.2288 2463.2215 2463.2506 -11.81 257 - 278 1 K.DFNVGGYIQAVLDRNLAENISR.V
2466.2324 2465.2251 2465.2339 -3.57 249 - 270 1 K.APNDFNLKDFNVGGYIQAVLDR.N
3153.5139 3152.5066 3152.5965 -28.49 325 - 352 1 R.TNFDAFPDKVAIQLNDTHPSLAIPELMR.I
No match to: 872.4761, 1068.5479, 1086.5554, 1115.5763, 1174.6033, 1261.7012, 1271.6656, 1277.6423, 1320.6438, 1372.7607, 1381.6793, 1456.7322, 1534.7764,
1536.7903, 1557.8268, 1561.7968, 1584.8268, 1662.8694, 1672.8704, 1689.8866, 1716.8770, 1739.9244, 1744.8021, 1835.8369, 1868.9391, 1869.9556, 1889.9683,
1914.9526, 1943.9541, 2028.0472, 2059.0444, 2159.2029, 2184.1106, 2225.1584, 2259.1499, 2371.2534
20. gi|8569323 Mass: 97634 Score: 217 Expect: 2.1e-016 Matches: 28
Chain A, Synergistic Inhibition Of Glycogen Phosphorylase A By A Potential Antidiabetic Drug And Caffeine
Observed Mr(expt) Mr(calc) ppm Start End Miss Peptide
893.4103 892.4030 892.4079 -5.46 185 - 191 0 R.YGNPWEK.A
963.4651 962.4578 962.4604 -2.63 235 - 242 0 R.NNVVNTMR.L + Oxidation (M)
994.5660 993.5587 993.5760 -17.38 34 - 41 0 R.HLHFTLVK.D
1053.5698 1052.5625 1052.5614 1.04 832 - 840 1 R.QRLPAPDEK.I
1117.5605 1116.5532 1116.5564 -2.83 414 - 424 0 R.VAAAFPGDVDR.L
1145.5627 1144.5554 1144.5553 0.11 161 - 169 0 R.YEFGIFNQK.I
1262.6217 1261.6144 1261.5867 22.0 773 - 782 0 K.VFADYEEYVK.C
1386.7540 1385.7467 1385.7415 3.74 414 - 426 1 R.VAAAFPGDVDRLR.R
1426.7781 1425.7708 1425.7728 -1.39 399 - 409 0 R.HLQIIYEINQR.F
1442.6910 1441.6837 1441.6878 -2.81 278 - 289 0 R.VLYPNDNFFEGK.E
1473.8547 1472.8474 1472.8504 -1.99 387 - 398 0 R.WPVHLLETLLPR.H
1537.7914 1536.7841 1536.7500 22.2 771 - 782 1 R.FKVFADYEEYVK.C
1566.7914 1565.7841 1565.7838 0.20 256 - 269 0 K.DFNVGGYIQAVLDR.N
1689.8866 1688.8793 1688.8787 0.36 192 - 205 0 K.ARPEFTLPVHFYGR.V
1756.9471 1755.9398 1755.9454 -3.17 576 - 589 0 R.QLLNCLHVITLYNR.I
1835.8369 1834.8296 1834.8196 5.47 773 - 786 1 K.VFADYEEYVKCQER.V
1840.9187 1839.9114 1839.9155 -2.23 278 - 292 1 R.VLYPNDNFFEGKELR.L
1874.9188 1873.9115 1873.8992 6.56 371 - 386 0 K.TCAYTNHTVLPEALER.W
1886.9027 1885.8954 1885.8958 -0.21 725 - 739 1 R.GYNAQEYYDRIPELR.Q
1889.9683 1888.9610 1889.0007 -21.00 622 - 639 0 K.LITAIGDVVNHDPVVGDR.L
2007.1241 2006.1168 2006.1200 -1.60 293 - 309 1 R.LKQEYFVVAATLQDIIR.R
2118.1248 2117.1175 2117.1303 -6.03 333 - 351 0 K.VAIQLNDTHPSLAIPELMR.V
2159.2029 2158.1956 2158.1859 4.52 622 - 641 1 K.LITAIGDVVNHDPVVGDRLR.V
2307.1401 2306.1328 2306.1406 -3.36 215 - 234 0 K.WVDTQVVLAMPYDTPVPGYR.N
2323.1353 2322.1280 2322.1355 -3.21 215 - 234 0 K.WVDTQVVLAMPYDTPVPGYR.N + Oxidation (M)
2464.2288 2463.2215 2463.2506 -11.81 256 - 277 1 K.DFNVGGYIQAVLDRNLAENISR.V
2466.2324 2465.2251 2465.2339 -3.57 248 - 269 1 K.APNDFNLKDFNVGGYIQAVLDR.N
3153.5139 3152.5066 3152.5965 -28.49 324 - 351 1 R.TNFDAFPDKVAIQLNDTHPSLAIPELMR.V
No match to: 872.4761, 1068.5479, 1086.5554, 1115.5763, 1133.6079, 1174.6033, 1261.7012, 1271.6656, 1277.6423, 1320.6438, 1372.7607, 1381.6793, 1456.7322,
1534.7764, 1536.7903, 1557.8268, 1561.7968, 1584.8268, 1623.8685, 1662.8694, 1672.8704, 1716.8770, 1739.9244, 1744.8021, 1792.7952, 1808.7908, 1868.9391,
1869.9556, 1914.9526, 1943.9541, 2028.0472, 2044.0458, 2059.0444, 2184.1106, 2225.1584, 2259.1499, 2371.2534

Search Parameters

Type of search : Peptide Mass Fingerprint
Enzyme : Trypsin
Fixed modifications : Carbamidomethyl (C)
Variable modifications : Oxidation (M)
Mass values : Monoisotopic
Protein Mass : Unrestricted
Peptide Mass Tolerance : ± 50 ppm
Peptide Charge State : 1+
Max Missed Cleavages : 1
Number of queries : 65

COVERAGE BAND 4

Mascot Search Results

Protein View

Match to: gi|154426116 Score: 463 Expect: 5.3e-041
Phosphorylase, glycogen, muscle [Bos taurus]
Nominal mass (Mr): 97683; Calculated pI value: 6.67
NCBI BLAST search of gi|154426116 against nr
Unformatted sequence string for pasting into other applications
Taxonomy: Bos taurus
Fixed modifications: Carbamidomethyl (C)
Variable modifications: Oxidation (M)
Cleavage by Trypsin: cuts C-term side of KR unless next residue is P
Number of mass values searched: 65
Number of mass values matched: 47
Sequence Coverage: 53%
Matched peptides shown in **Bold Red**

```
1 MSRPLTDQEK RKQISVRGLA GVENVTELKK NFNRHLHFTL VKDRNVATPR
51 DYYFALAYTV RDHLVGRWIR TQQHYYEKDP KRIYYLSLEF YIGRTLQNTM
101 VNLALENACD EATYQLGLDM EELEEIEEDA GLGNGGLGRL AACFLDSMAT
151 LGLAAYGYGI RYEFGIFNQK ISGGWQMEEA DDWLRYGNPW EKARPEFTLP
201 VHFYGRVEHT SQGAKWVDTQ VVLAMPYDTP VPGYRNNVVN TMRLWSAKAP
251 NDFNLKDFNV GGYIQAVLDR NLAENISRVL YPNDNFFEGK ELRLKQEYFV
301 VAATLQDIIR RFKSSKFGCL DPVRTNFDAF PDKVAIQLND THPSLAIPEL
351 MRILVDQERL EWEKAWEVTV KTCAYTNHTV LPEALERWPV HLIETLLPRH
401 LQIIYEINQR FLNRVAAAFP GDVDRLRRMS LVEEGAVKRI NMAHLCIAGS
451 HAVNGVARIH SEILKKTIFK DFYELEPHKF QNKTNGITPR RWLVMCNPGL
501 AEIIAERIGE EYIADLDQLR KLLSYVDDES FIRDVAKVKQ ENKLKFSAYL
551 EKEYKVHINP NSLFDIQVKR IHEYKRQLLN CLHVITLYNR IKKEPNKFFV
601 PRTVMIGGKA APGYHMAKMI IKLITAIGDV VNHDPVVGDR LRVIFLENYR
651 VSLAEKVIPA ADLSEQISTA GTEASGTGNM KFMLNGALTI GTMDGANVEM
701 AEEAGEENFF IFGMRVEDVE RLDHKGYNAQ EYYDRIPELR HVIDQLSSGF
751 FSPKQPDLFK DIVNMLMHHD RFKVFADYEE YVKCQERVSA LYKNPREWTR
801 MVIRNIATSG KFSSDRTIAQ YAREIWGVEP TRQRMPAPDE KI
```

Show predicted peptides also
Sort Peptides By Residue Number Increasing Mass Decreasing Mass

Start - End	Observed	Mr(expt)	Mr(calc)	ppm	Miss	Sequence
35 - 42	994.5660	993.5587	993.5760	-17	0	R.HLHFTLVK.D
51 - 61	1381.6793	1380.6720	1380.6714	0	0	R.DYYFALAYTVR.D
51 - 67	2059.0444	2058.0371	2058.0323	2	1	R.DYYFALAYTVRDHLVGR.W
83 - 94	1536.7903	1535.7830	1535.8024	-13	0	R.IYYLSLEFYIGR.T
162 - 170	1145.5627	1144.5554	1144.5553	0	0	R.YEFGIFNQK.I
171 - 185	1792.7952	1791.7879	1791.7886	-0	0	K.ISGGWQMEEADDWLR.Y
171 - 185	1808.7908	1807.7835	1807.7835	-0	0	K.ISGGWQMEEADDWLR.Y Oxidation (M)
186 - 192	893.4103	892.4030	892.4079	-5	0	R.YGNPWEK.A
193 - 206	1689.8866	1688.8793	1688.8787	0	0	K.ARPEFTLPVHFYGR.V
216 - 235	2307.1401	2306.1328	2306.1406	-3	0	K.WVDTQVVLAMPYDTPVPGYR.N
216 - 235	2323.1353	2322.1280	2322.1355	-3	0	K.WVDTQVVLAMPYDTPVPGYR.N Oxidation (M)
236 - 243	963.4651	962.4578	962.4604	-3	0	R.NNVVNTMR.L Oxidation (M)
249 - 270	2466.2324	2465.2251	2465.2339	-4	1	K.APNDFNLKDFNVGGYIQAVLDR.N
257 - 270	1566.7914	1565.7841	1565.7838	0	0	K.DFNVGGYIQAVLDR.N
257 - 278	2464.2288	2463.2215	2463.2506	-12	1	K.DFNVGGYIQAVLDRNLAENISR.V
279 - 290	1442.6910	1441.6837	1441.6878	-3	0	R.VLYPNDNFFEGK.E
279 - 293	1840.9187	1839.9114	1839.9155	-2	1	R.VLYPNDNFFEGKELR.L
294 - 310	2007.1241	2006.1168	2006.1200	-2	1	R.LKQEYFVVAATLQDIIR.R
325 - 352	3153.5139	3152.5066	3152.5965	-28	1	R.TNFDAFPDKVAIQLNDTHPSLAIPELMR.I
334 - 352	2118.1248	2117.1175	2117.1303	-6	0	K.VAIQLNDTHPSLAIPELMR.I
353 - 359	872.4761	871.4688	871.4763	-9	0	R.ILVDQER.L
353 - 364	1557.8268	1556.8195	1556.8198	-0	1	R.ILVDQERLEWEK.A
372 - 387	1874.9188	1873.9115	1873.8992	7	0	K.TCAYTNHTVLPEALER.W
388 - 399	1473.8547	1472.8474	1472.8504	-2	0	R.WPVHLIETLLPR.H
400 - 410	1426.7781	1425.7708	1425.7728	-1	0	R.HLQIIYEINQR.F
415 - 425	1117.5605	1116.5532	1116.5564	-3	0	R.VAAAFPGDVDR.L
415 - 427	1386.7540	1385.7467	1385.7415	4	1	R.VAAAFPGDVDRLR.R
491 - 507	2028.0472	2027.0399	2027.0444	-2	1	R.RWLVMCNPGLAEIIAER.I
491 - 507	2044.0458	2043.0385	2043.0394	-0	1	R.RWLVMCNPGLAEIIAER.I Oxidation (M)
508 - 520	1534.7764	1533.7691	1533.7675	1	0	R.IGEEYIADLDQLR.K
508 - 521	1662.8694	1661.8621	1661.8624	-0	1	R.IGEEYIADLDQLRK.L
521 - 533	1584.8268	1583.8195	1583.8195	0	1	R.KLLSYVDDESFIR.D
522 - 533	1456.7322	1455.7249	1455.7245	0	0	K.LLSYVDDESFIR.D
522 - 537	1869.9556	1868.9483	1868.9520	-2	1	K.LLSYVDDESFIRDVAK.V
546 - 555	1277.6423	1276.6350	1276.6339	1	1	K.FSAYLEKEYK.V
556 - 569	1623.8685	1622.8612	1622.8780	-10	0	K.VHINPNSLFDIQVK.R
577 - 590	1756.9471	1755.9398	1755.9454	-3	0	R.QLLNCLHVITLYNR.I
594 - 602	1133.6079	1132.6006	1132.6029	-2	1	K.EPNKFFVPR.T
623 - 642	2159.2029	2158.1956	2158.1859	5	1	K.LITAIGDVVNHDPVVGDRLR.V
643 - 650	1053.5698	1052.5625	1052.5655	-3	0	R.VIFLENYR.V
726 - 740	1886.9027	1885.8954	1885.8958	-0	1	K.GYNAQEYYDRIPELR.H
741 - 754	1561.7968	1560.7895	1560.7937	-3	0	R.HVIDQLSSGFFSPK.Q

772 - 783 1537.7914 1536.7841 1536.7500 22 1 R.FKVFADYEEYVK.C
774 - 783 1262.6217 1261.6144 1261.5867 22 0 K.VFADYEEYVK.C
774 - 787 1835.8369 1834.8296 1834.8196 5 1 K.VFADYEEYVKCQER.V
817 - 832 1889.9683 1888.9610 1888.9795 -10 1 R.TIAQYAREIWGVEPTR.Q
824 - 832 1086.5554 1085.5481 1085.5506 -2 0 R.EIWGVEPTR.Q
No match to: 1068.5479, 1115.5763, 1174.6033, 1261.7012, 1271.6656, 1320.6438, 1372.7607, 1672.8704, 1716.8770, 1739.9244, 1744.

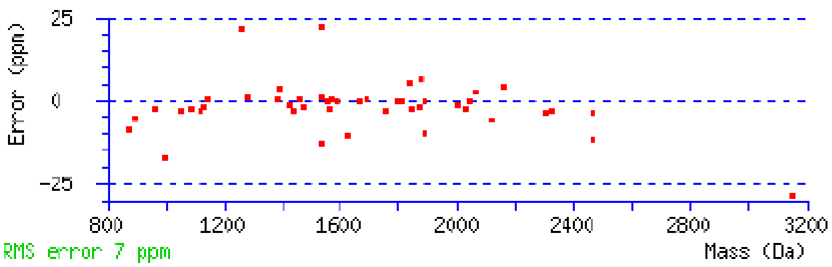

BAND 5

Mascot Search Results

User :
Email :
Search title : SampleSetID: 824, AnalysisID: 7242, MaldiWellID: 69609, SpectrumID: 154534, Path=\180719\MS\18-106 NCBI Mammalia
Database : NCBInr 20120508 (17919084 sequences; 6150218869 residues)
Taxonomy : Mammalia (mammals) (1061927 sequences)
Timestamp : 19 Jul 2018 at 12:05:50 GMT
Top Score : 146 for Mixture 1, gi|261825070 + gi|148693577

Mascot Score Histogram

Protein score is -10*Log(P), where P is the probability that the observed match is a random event.
Protein scores greater than 73 are significant (p<0.05).

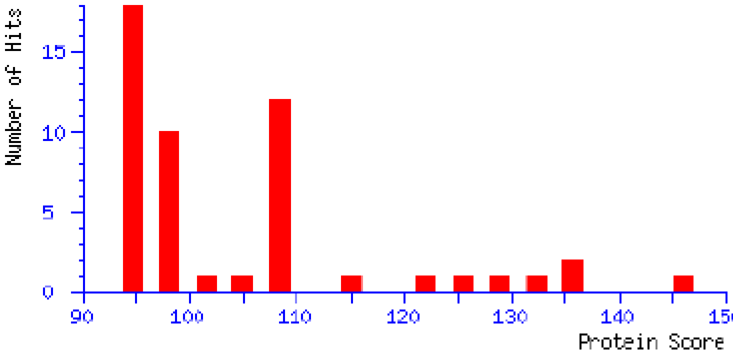

Protein Summary Report

Format As Protein Summary Help
Significance threshold p< 0.05 Max. number of hits 20
Re-Search All Search Unmatched

Index

Accession Mass Score Description
1. Mixture 1 146 gi|261825070 + gi|148693577
2. Mixture 2 135 gi|2495339 + gi|148693577
3. Mixture 3 135 gi|371767260 + gi|148693577
4. Mixture 4 132 gi|348576464 + gi|148693577
5. Mixture 5 128 gi|462326 + gi|225698069
6. Mixture 6 124 gi|62089222 + gi|148693577
7. gi|261825070 45299 121 Chain A, Crystal Structure Of The Human 70kda Heat Shock Protein 1a (Hsp70-1) Atpase Domain In Complex With Adp And Inorganic Phosphate
8. Mixture 7 114 gi|194388088 + gi|148693577
9. Mixture 8 109 gi|6016261 + gi|148693577
10. gi|2495339 70470 109 RecName: Full=Heat shock 70 kDa protein 1B; AltName: Full=Heat shock 70 kDa protein 2; Short=HSP70.2
11. gi|56757663 70500 109 RecName: Full=Heat shock 70 kDa protein 1A; AltName: Full=Heat shock 70 kDa protein 1; Short=HSP70.1
12. gi|40254806 70492 109 heat shock 70 kDa protein 1A [Bos taurus]
13. gi|312064069 70514 109 heat shock 70 kDa protein 1A [Bos indicus]
14. gi|332078832 70385 109 heat shock protein 70 1A [Camelus dromedarius]

15. gi|334904119 70471 109 heat shock protein 70.1 [Capra hircus]
16. gi|343432731 70485 109 heat shock protein 70.1 [Capra hircus]
17. gi|371767260 70596 109 heat shock protein 70 [Capra hircus]
18. gi|166007012 43182 109 Chain A, Crystal Structure Of The Human Hsp70 Atpase Domain In The Apo Form
19. gi|166007013 43182 109 Chain A, Crystal Structure Of The Human Hsp70 Atpase Domain In Complex With Amp-Pnp
20. gi|292659561 43239 109 Chain A, Crystal Structure Of The Complex Between The Bag5 Bd5 And Hsp70 Nbd

Results List

1. Mixture 1 Total score: 146 Expect: 2.7e-009 Matches: 24
Components: 1. gi|261825070 Chain A, Crystal Structure Of The Human 70kda Heat Shock Protein 1a (Hsp70-1) Atpase Domain In Complex With Adp And Inorganic Phosphate
2. gi|148693577 mCG5074, isoform CRA_a [Mus musculus]
Observed Mr(expt) Mr(calc) ppm Start End Miss Comp Peptide
1109.5812 1108.5739 1108.5665 6.66 371 - 379 0 1 K.LLQDFFNGR.D
1197.6906 1196.6833 1196.6877 -3.67 182 - 193 0 1 K.DAGVIAGLNVLR.I
1252.6086 1251.6013 1251.6533 -41.49 62 - 72 1 2 K.MKEIAEAYLGK.T
1253.6166 1252.6093 1252.6088 0.43 237 - 246 0 2 R.FEELNADLFR.G
1417.7695 1416.7622 1416.7514 7.66 259 - 269 1 1 R.LVNHFVEEFKR.K
1480.7639 1479.7566 1479.7470 6.51 235 - 246 1 2 R.ARFEELNADLFR.G
1487.7126 1486.7053 1486.6940 7.62 59 - 71 0 1 R.TTPSYVAFTDTER.L
1542.7499 1541.7426 1541.7296 8.42 322 - 333 1 1 R.ARFEELCSDLFR.S
1565.8301 1564.8228 1564.8249 -1.34 284 - 296 1 2 K.LLQDFFNGKELNK.S
1579.8413 1578.8340 1578.8154 11.8 371 - 383 1 1 K.LLQDFFNGRDLNK.S
1630.8326 1629.8253 1629.7960 18.0 135 - 148 0 1 K.AFYPEEISSMVLTK.M + Oxidation (M)
1675.7352 1674.7279 1674.7234 2.70 243 - 258 0 1 K.ATAGDTHLGGEDFDNR.L
1687.9100 1686.9027 1686.8940 5.15 194 - 209 0 1 R.IINEPTAAAIAYGLDR.T
1691.8064 1690.7991 1690.7183 47.8 156 - 171 0 2 K.STAGDTHLGGEDFDNR.M
1814.9541 1813.9468 1813.9435 1.84 79 - 94 1 1 K.NQVALNPQNTVFDAKR.L
1822.0240 1821.0167 1821.0108 3.23 348 - 364 1 1 K.LDKAQIHDLVLVGGSTR.I
1838.0254 1837.0181 1837.0058 6.73 261 - 277 1 2 K.LDKSQIHDIVLVGGSTR.I
1907.9373 1906.9300 1906.9056 12.8 48 - 63 1 2 K.SFYPEEVSSMVLTKMK.E + 2 Oxidation (M)
1982.0004 1980.9931 1980.9905 1.31 73 - 90 0 2 K.TVTNAVVTVPAYFNDSQR.Q
2769.3118 2768.3045 2768.2626 15.1 2 - 25 0 1 M.HHHHHHSSGVDLGTENLYFQSMAK.A
2774.3113 2773.3040 2773.3956 -33.02 380 - 406 1 1 R.DLNKSINPDEAVAYGAAVQAAILMGDK.S
2981.4358 2980.4285 2980.4553 -8.99 295 - 321 0 1 R.TLSSSTQASLEIDSLFEGIDFYTSITR.A
2997.4368 2996.4295 2996.4502 -6.91 208 - 234 0 2 R.TLSSSTQASIEIDSLYEGIDFYTSITR.A
3153.4426 3152.4353 3152.5513 -36.80 207 - 234 1 2 K.RTLSSSTQASIEIDSLYEGIDFYTSITR.A
No match to: 855.0549, 887.0070, 927.4994, 1056.5944, 1110.5702, 1163.6331, 1232.5702, 1320.5953, 1326.6932, 1377.6681, 1439.8186, 1456.7246, 1463.7484,
1472.7527, 1473.8469, 1537.8105, 1567.7526, 1580.8241, 1638.8689, 1639.9293, 1642.8138, 1690.8757, 1694.8363, 1710.8218, 1735.8954, 1782.8805, 1880.9231,
1915.9832, 1980.0317, 1994.0042, 2045.0317, 2225.1553, 2289.1572, 2492.2678, 2545.2520, 2570.1982, 2588.2024, 2612.1765, 2786.3582, 2975.4814, 3136.4468

2. Mixture 2 Total score: 135 Expect: 3.4e-008 Matches: 25
Components: 1. gi|2495339 RecName: Full=Heat shock 70 kDa protein 1B; AltName: Full=Heat shock 70 kDa protein 2; Short=HSP70.2
2. gi|148693577 mCG5074, isoform CRA_a [Mus musculus]
Observed Mr(expt) Mr(calc) ppm Start End Miss Comp Peptide
1109.5812 1108.5739 1108.5665 6.66 349 - 357 0 1 K.LLQDFFNGR.D
1197.6906 1196.6833 1196.6877 -3.67 160 - 171 0 1 K.DAGVIAGLNVLR.I
1252.6086 1251.6013 1251.6533 -41.49 62 - 72 1 2 K.MKEIAEAYLGK.T
1253.6166 1252.6093 1252.6088 0.43 237 - 246 0 2 R.FEELNADLFR.G
1417.7695 1416.7622 1416.7514 7.66 237 - 247 1 1 R.LVNHFVEEFKR.K
1480.7639 1479.7566 1479.7470 6.51 235 - 246 1 2 R.ARFEELNADLFR.G
1487.7126 1486.7053 1486.6940 7.62 37 - 49 0 1 R.TTPSYVAFTDTER.L
1542.7499 1541.7426 1541.7296 8.42 300 - 311 1 1 R.ARFEELCSDLFR.S
1565.8301 1564.8228 1564.8249 -1.34 284 - 296 1 2 K.LLQDFFNGKELNK.S
1579.8413 1578.8340 1578.8154 11.8 349 - 361 1 1 K.LLQDFFNGRDLNK.S
1630.8326 1629.8253 1629.7960 18.0 113 - 126 0 1 K.AFYPEEISSMVLTK.M + Oxidation (M)
1675.7352 1674.7279 1674.7234 2.70 221 - 236 0 1 K.ATAGDTHLGGEDFDNR.L
1687.9100 1686.9027 1686.8940 5.15 172 - 187 0 1 R.IINEPTAAAIAYGLDR.T
1691.8064 1690.7991 1690.7183 47.8 156 - 171 0 2 K.STAGDTHLGGEDFDNR.M
1814.9541 1813.9468 1813.9435 1.84 57 - 72 1 1 K.NQVALNPQNTVFDAKR.L
1822.0240 1821.0167 1821.0108 3.23 326 - 342 1 1 K.LDKAQIHDLVLVGGSTR.I
1838.0254 1837.0181 1837.0058 6.73 261 - 277 1 2 K.LDKSQIHDIVLVGGSTR.I
1907.9373 1906.9300 1906.9056 12.8 48 - 63 1 2 K.SFYPEEVSSMVLTKMK.E + 2 Oxidation (M)
1982.0004 1980.9931 1980.9905 1.31 73 - 90 0 2 K.TVTNAVVTVPAYFNDSQR.Q
2774.3113 2773.3040 2773.3956 -33.02 358 - 384 1 1 R.DLNKSINPDEAVAYGAAVQAAILMGDK.S
2786.3582 2785.3509 2785.3559 -1.79 424 - 447 0 1 K.QTQIFTTYSDNQPGVLIQVYEGER.A
2975.4814 2974.4741 2974.4825 -2.81 129 - 155 0 1 K.EIAEAYLGHPVTNAVITVPAYFNDSQR.Q
2981.4358 2980.4285 2980.4553 -8.99 273 - 299 0 1 R.TLSSSTQASLEIDSLFEGIDFYTSITR.A
2997.4368 2996.4295 2996.4502 -6.91 208 - 234 0 2 R.TLSSSTQASIEIDSLYEGIDFYTSITR.A
3153.4426 3152.4353 3152.5513 -36.80 207 - 234 1 2 K.RTLSSSTQASIEIDSLYEGIDFYTSITR.A
No match to: 855.0549, 887.0070, 927.4994, 1056.5944, 1110.5702, 1163.6331, 1232.5702, 1320.5953, 1326.6932, 1377.6681, 1439.8186, 1456.7246, 1463.7484,
1472.7527, 1473.8469, 1537.8105, 1567.7526, 1580.8241, 1638.8689, 1639.9293, 1642.8138, 1690.8757, 1694.8363, 1710.8218, 1735.8954, 1782.8805, 1880.9231,
1915.9832, 1980.0317, 1994.0042, 2045.0317, 2225.1553, 2289.1572, 2492.2678, 2545.2520, 2570.1982, 2588.2024, 2612.1765, 2769.3118, 3136.4468

3. Mixture 3 Total score: 135 Expect: 3.4e-008 Matches: 25
Components: 1. gi|371767260 heat shock protein 70 [Capra hircus]
2. gi|148693577 mCG5074, isoform CRA_a [Mus musculus]
Observed Mr(expt) Mr(calc) ppm Start End Miss Comp Peptide
1109.5812 1108.5739 1108.5665 6.66 349 - 357 0 1 K.LLQDFFNGR.D
1197.6906 1196.6833 1196.6877 -3.67 160 - 171 0 1 K.DAGVIAGLNVLR.I
1252.6086 1251.6013 1251.6533 -41.49 62 - 72 1 2 K.MKEIAEAYLGK.T
1253.6166 1252.6093 1252.6088 0.43 237 - 246 0 2 R.FEELNADLFR.G
1417.7695 1416.7622 1416.7514 7.66 237 - 247 1 1 R.LVNHFVEEFKR.K
1480.7639 1479.7566 1479.7470 6.51 235 - 246 1 2 R.ARFEELNADLFR.G
1487.7126 1486.7053 1486.6940 7.62 37 - 49 0 1 R.TTPSYVAFTDTER.L
1542.7499 1541.7426 1541.7296 8.42 300 - 311 1 1 R.ARFEELCSDLFR.S
1565.8301 1564.8228 1564.8249 -1.34 284 - 296 1 2 K.LLQDFFNGKELNK.S
1579.8413 1578.8340 1578.8154 11.8 349 - 361 1 1 K.LLQDFFNGRDLNK.S

1675.7352 1674.7279 1674.7234 2.70 221 - 236 0 1 K.ATAGDTHLGGEDFDNR.L
1690.8757 1689.8684 1689.8574 6.55 94 - 108 1 1 R.VINDGDEPKVQVSYK.G
1691.8064 1690.7991 1690.7183 47.8 156 - 171 0 2 K.STAGDTHLGGEDFDNR.M
1814.9541 1813.9468 1813.9435 1.84 57 - 72 1 1 K.NQVALNPQNTVFDAKR.L
1822.0240 1821.0167 1821.0108 3.23 326 - 342 1 1 K.LDKAQIHDLVLVGGSTR.I
1838.0254 1837.0181 1837.0058 6.73 261 - 277 1 2 K.LDKSQIHDIVLVGGSTR.I
1907.9373 1906.9300 1906.9056 12.8 48 - 63 1 2 K.SFYPEEVSSMVLTKMK.E + 2 Oxidation (M)
1915.9832 1914.9759 1914.9835 -3.93 113 - 128 1 1 K.AFYPEEISLMVLTKMK.E + Oxidation (M)
1982.0004 1980.9931 1980.9905 1.31 73 - 90 0 2 K.TVTNAVVTVPAYFNDSQR.Q
2774.3113 2773.3040 2773.3956 -33.02 358 - 384 1 1 R.DLNKSINPDEAVAYGAAVQAAILMGDK.S
2786.3582 2785.3509 2785.3559 -1.79 424 - 447 0 1 K.QTQIFTTYSDNQPGVLIQVYEGER.A
2975.4814 2974.4741 2974.4825 -2.81 129 - 155 0 1 K.EIAEAYLGHPVTNAVITVPAYFNDSQR.Q
2981.4358 2980.4285 2980.4553 -8.99 273 - 299 0 1 R.TLSSSTQASLEIDSLFEGIDFYTSITR.A
2997.4368 2996.4295 2996.4502 -6.91 208 - 234 0 2 R.TLSSSTQASIEIDSLYEGIDFYTSITR.A
3153.4426 3152.4353 3152.5513 -36.80 207 - 234 1 2 K.RTLSSSTQASIEIDSLYEGIDFYTSITR.A
No match to: 855.0549, 887.0070, 927.4994, 1056.5944, 1110.5702, 1163.6331, 1232.5702, 1320.5953, 1326.6932, 1377.6681, 1439.8186, 1456.7246, 1463.7484,
1472.7527, 1473.8469, 1537.8105, 1567.7526, 1580.8241, 1630.8326, 1638.8689, 1639.9293, 1642.8138, 1687.9100, 1694.8363, 1710.8218, 1735.8954, 1782.8805,
1880.9231, 1980.0317, 1994.0042, 2045.0317, 2225.1553, 2289.1572, 2492.2678, 2545.2520, 2570.1982, 2588.2024, 2612.1765, 2769.3118, 3136.4468
4. Mixture 4 Total score: 132 Expect: 6.7e-008 Matches: 25
Components: 1. gi|348576464 PREDICTED: heat shock 70 kDa protein 1B-like [Cavia porcellus]
2. gi|148693577 mCG5074, isoform CRA_a [Mus musculus]
Observed Mr(expt) Mr(calc) ppm Start End Miss Comp Peptide
1109.5812 1108.5739 1108.5665 6.66 349 - 357 0 1 K.LLQDFFNGR.D
1197.6906 1196.6833 1196.6877 -3.67 160 - 171 0 1 K.DAGVIAGLNVLR.I
1252.6086 1251.6013 1251.6533 -41.49 62 - 72 1 2 K.MKEIAEAYLGK.T
1253.6166 1252.6093 1252.6088 0.43 237 - 246 0 2 R.FEELNADLFR.G
1417.7695 1416.7622 1416.7514 7.66 237 - 247 1 1 R.LVNHFVEEFKR.K
1480.7639 1479.7566 1479.7470 6.51 235 - 246 1 2 R.ARFEELNADLFR.G
1487.7126 1486.7053 1486.6940 7.62 37 - 49 0 1 R.TTPSYVAFTDTER.L
1542.7499 1541.7426 1541.7296 8.42 300 - 311 1 1 R.ARFEELCSDLFR.G
1565.8301 1564.8228 1564.8249 -1.34 284 - 296 1 2 K.LLQDFFNGKELNK.S
1579.8413 1578.8340 1578.8154 11.8 349 - 361 1 1 K.LLQDFFNGRDLNK.S
1630.8326 1629.8253 1629.7960 18.0 113 - 126 0 1 K.AFYPEEISSMVLTK.M + Oxidation (M)
1675.7352 1674.7279 1674.7234 2.70 221 - 236 0 1 K.ATAGDTHLGGEDFDNR.L
1687.9100 1686.9027 1686.8940 5.15 172 - 187 0 1 R.IINEPTAAAIAYGLDR.T
1691.8064 1690.7991 1690.7183 47.8 156 - 171 0 2 K.STAGDTHLGGEDFDNR.M
1694.8363 1693.8290 1693.8576 -16.89 89 - 102 0 1 K.HWPFQVINEGDKPK.V
1814.9541 1813.9468 1813.9435 1.84 57 - 72 1 1 K.NQVALNPQNTVFDAKR.L
1822.0240 1821.0167 1821.0108 3.23 326 - 342 1 1 K.LDKAQIHDLVLVGGSTR.I
1838.0254 1837.0181 1837.0058 6.73 261 - 277 1 2 K.LDKSQIHDIVLVGGSTR.I
1907.9373 1906.9300 1906.9056 12.8 48 - 63 1 2 K.SFYPEEVSSMVLTKMK.E + 2 Oxidation (M)
1982.0004 1980.9931 1980.9905 1.31 73 - 90 0 2 K.TVTNAVVTVPAYFNDSQR.Q
2774.3113 2773.3040 2773.3956 -33.02 358 - 384 1 1 R.DLNKSINPDEAVAYGAAVQAAILMGDK.S
2786.3582 2785.3509 2785.3559 -1.79 424 - 447 0 1 K.QTQIFTTYSDNQPGVLIQVYEGER.A
2981.4358 2980.4285 2980.4553 -8.99 273 - 299 0 1 R.TLSSSTQASLEIDSLFEGIDFYTSITR.A
2997.4368 2996.4295 2996.4502 -6.91 208 - 234 0 2 R.TLSSSTQASIEIDSLYEGIDFYTSITR.A
3153.4426 3152.4353 3152.5513 -36.80 207 - 234 1 2 K.RTLSSSTQASIEIDSLYEGIDFYTSITR.A
No match to: 855.0549, 887.0070, 927.4994, 1056.5944, 1110.5702, 1163.6331, 1232.5702, 1320.5953, 1326.6932, 1377.6681, 1439.8186, 1456.7246, 1463.7484,
1472.7527, 1473.8469, 1537.8105, 1567.7526, 1580.8241, 1638.8689, 1639.9293, 1642.8138, 1690.8757, 1710.8218, 1735.8954, 1782.8805, 1880.9231, 1915.9832,
1980.0317, 1994.0042, 2045.0317, 2225.1553, 2289.1572, 2492.2678, 2545.2520, 2570.1982, 2588.2024, 2612.1765, 2769.3118, 2975.4814, 3136.4468
5. Mixture 5 Total score: 128 Expect: 1.7e-007 Matches: 25
Components: 1. gi|462326 RecName: Full=Heat shock 70 kDa protein 1A; AltName: Full=Heat shock 70 kDa protein 1; Short=HSP70.1
2. gi|225698069 Chain A, Crystal Structure Of Hsc70BAG1 IN COMPLEX WITH ATP
Observed Mr(expt) Mr(calc) ppm Start End Miss Comp Peptide
1109.5812 1108.5739 1108.5665 6.66 349 - 357 0 1 K.LLQDFFNGR.D
1197.6906 1196.6833 1196.6877 -3.67 160 - 171 0 1 K.DAGVIAGLNVLR.I
1252.6086 1251.6013 1251.6533 -41.49 127 - 137 1 2 K.MKEIAEAYLGK.T
1253.6166 1252.6093 1252.6088 0.43 302 - 311 0 2 R.FEELNADLFR.G
1417.7695 1416.7622 1416.7514 7.66 237 - 247 1 1 R.LVNHFVEEFKR.K
1480.7639 1479.7566 1479.7470 6.51 300 - 311 1 2 R.ARFEELNADLFR.G
1487.7126 1486.7053 1486.6940 7.62 37 - 49 0 2 R.TTPSYVAFTDTER.L
1542.7499 1541.7426 1541.7296 8.42 300 - 311 1 1 R.ARFEELCSDLFR.S
1565.8301 1564.8228 1564.8249 -1.34 349 - 361 1 2 K.LLQDFFNGKELNK.S
1579.8413 1578.8340 1578.8154 11.8 349 - 361 1 1 K.LLQDFFNGRDLNK.S
1642.8138 1641.8065 1641.8184 -7.26 536 - 550 1 1 R.VGAKNALESYAFNMK.S
1675.7352 1674.7279 1674.7234 2.70 221 - 236 0 1 K.ATAGDTHLGGEDFDNR.L
1687.9100 1686.9027 1686.8940 5.15 172 - 187 0 1 R.IINEPTAAAIAYGLDR.T
1691.8064 1690.7991 1690.7183 47.8 221 - 236 0 2 K.STAGDTHLGGEDFDNR.M
1814.9541 1813.9468 1813.9435 1.84 57 - 72 1 1 K.NQVALNPQNTVFDAKR.L
1822.0240 1821.0167 1821.0108 3.23 326 - 342 1 1 K.LDKAQIHDLVLVGGSTR.I
1838.0254 1837.0181 1837.0058 6.73 326 - 342 1 2 K.LDKSQIHDIVLVGGSTR.I
1907.9373 1906.9300 1906.9056 12.8 113 - 128 1 2 K.SFYPEEVSSMVLTKMK.E + 2 Oxidation (M)
1915.9832 1914.9759 1914.9200 29.2 78 - 93 1 1 K.FGDPVVQGDMKHWPFR.V
1982.0004 1980.9931 1980.9905 1.31 138 - 155 0 2 K.TVTNAVVTVPAYFNDSQR.Q
2774.3113 2773.3040 2773.3956 -33.02 358 - 384 1 1 R.DLNKSINPDEAVAYGAAVQAAILMGDK.S
2786.3582 2785.3509 2785.3559 -1.79 424 - 447 0 1 K.QTQIFTTYSDNQPGVLIQVYEGER.A
2981.4358 2980.4285 2980.4553 -8.99 273 - 299 0 1 R.TLSSSTQASLEIDSLFEGIDFYTSITR.A
2997.4368 2996.4295 2996.4502 -6.91 273 - 299 0 2 R.TLSSSTQASIEIDSLYEGIDFYTSITR.A
3153.4426 3152.4353 3152.5513 -36.80 272 - 299 1 2 K.RTLSSSTQASIEIDSLYEGIDFYTSITR.A
No match to: 855.0549, 887.0070, 927.4994, 1056.5944, 1110.5702, 1163.6331, 1232.5702, 1320.5953, 1326.6932, 1377.6681, 1439.8186, 1456.7246, 1463.7484,
1472.7527, 1473.8469, 1537.8105, 1567.7526, 1580.8241, 1630.8326, 1638.8689, 1639.9293, 1690.8757, 1694.8363, 1710.8218, 1735.8954, 1782.8805, 1880.9231,
1980.0317, 1994.0042, 2045.0317, 2225.1553, 2289.1572, 2492.2678, 2545.2520, 2570.1982, 2588.2024, 2612.1765, 2769.3118, 2975.4814, 3136.4468
6. Mixture 6 Total score: 124 Expect: 4.2e-007 Matches: 25

Components: 1. gi|62089222 heat shock 70kDa protein 1A variant [Homo sapiens]
2. gi|148693577 mCG5074, isoform CRA_a [Mus musculus]
Observed Mr(expt) Mr(calc) ppm Start End Miss Comp Peptide
1109.5812 1108.5739 1108.5665 6.66 417 - 425 0 1 K.LLQDFFNGR.D
1197.6906 1196.6833 1196.6877 -3.67 228 - 239 0 1 K.DAGVIAGLNVLR.I
1252.6086 1251.6013 1251.6533 -41.49 62 - 72 1 2 K.MKEIAEAYLGK.T
1253.6166 1252.6093 1252.6088 0.43 237 - 246 0 2 R.FEELNADLFR.G
1417.7695 1416.7622 1416.7514 7.66 305 - 315 1 1 R.LVNHFVEEFKR.K
1463.7484 1462.7411 1462.6834 39.4 48 - 60 0 1 R.IQCSVSSPQSQSR.A
1480.7639 1479.7566 1479.7470 6.51 235 - 246 1 2 R.ARFEELNADLFR.G
1487.7126 1486.7053 1486.6940 7.62 105 - 117 0 1 R.TTPSYVAFTDTER.L
1542.7499 1541.7426 1541.7296 8.42 368 - 379 1 1 R.ARFEELCSDLFR.S
1565.8301 1564.8228 1564.8249 -1.34 284 - 296 1 2 K.LLQDFFNGKELNK.S
1579.8413 1578.8340 1578.8154 11.8 417 - 429 1 1 K.LLQDFFNGRDLNK.S
1630.8326 1629.8253 1629.7960 18.0 181 - 194 0 1 K.AFYPEEISSMVLTK.M + Oxidation (M)
1675.7352 1674.7279 1674.7234 2.70 289 - 304 0 1 K.ATAGDTHLGGEDFDNR.L
1687.9100 1686.9027 1686.8940 5.15 240 - 255 0 1 R.IINEPTAAAIAYGLDR.T
1691.8064 1690.7991 1690.7183 47.8 156 - 171 0 2 K.STAGDTHLGGEDFDNR.M
1814.9541 1813.9468 1813.9435 1.84 125 - 140 1 1 K.NQVALNPQNTVFDAKR.L
1822.0240 1821.0167 1821.0108 3.23 394 - 410 1 1 K.LDKAQIHDLVLVGGSTR.I
1838.0254 1837.0181 1837.0058 6.73 261 - 277 1 2 K.LDKSQIHDIVLVGGSTR.I
1907.9373 1906.9300 1906.9056 12.8 48 - 63 1 2 K.SFYPEEVSSMVLTKMK.E + 2 Oxidation (M)
1982.0004 1980.9931 1980.9905 1.31 73 - 90 0 2 K.TVTNAVVTVPAYFNDSQR.Q
2774.3113 2773.3040 2773.3956 -33.02 426 - 452 1 1 R.DLNKSINPDEAVAYGAAVQAAILMGDK.S
2786.3582 2785.3509 2785.3559 -1.79 492 - 515 0 1 K.QTQIFTTYSDNQPGVLIQVYEGER.A
2981.4358 2980.4285 2980.4553 -8.99 341 - 367 0 1 R.TLSSSTQASLEIDSLFEGIDFYTSITR.A
2997.4368 2996.4295 2996.4502 -6.91 208 - 234 0 2 R.TLSSSTQASIEIDSLYEGIDFYTSITR.A
3153.4426 3152.4353 3152.5513 -36.80 207 - 234 1 2 K.RTLSSSTQASIEIDSLYEGIDFYTSITR.A
No match to: 855.0549, 887.0070, 927.4994, 1056.5944, 1110.5702, 1163.6331, 1232.5702, 1320.5953, 1326.6932, 1377.6681, 1439.8186,
1456.7246, 1472.7527,
1473.8469, 1537.8105, 1567.7526, 1580.8241, 1638.8689, 1639.9293, 1642.8138, 1690.8757, 1694.8363, 1710.8218, 1735.8954, 1782.8805,
1880.9231, 1915.9832,
1980.0317, 1994.0042, 2045.0317, 2225.1553, 2289.1572, 2492.2678, 2545.2520, 2570.1982, 2588.2024, 2612.1765, 2769.3118, 2975.4814,
3136.4468
7. gi|261825070 Mass: 45299 Score: 121 Expect: 8.4e-007 Matches: 14
Chain A, Crystal Structure Of The Human 70kda Heat Shock Protein 1a (Hsp70-1) Atpase Domain In Complex With Adp And Inorganic
Phosphate
Observed Mr(expt) Mr(calc) ppm Start End Miss Peptide
1109.5812 1108.5739 1108.5665 6.66 371 - 379 0 K.LLQDFFNGR.D
1197.6906 1196.6833 1196.6877 -3.67 182 - 193 0 K.DAGVIAGLNVLR.I
1417.7695 1416.7622 1416.7514 7.66 259 - 269 1 R.LVNHFVEEFKR.K
1487.7126 1486.7053 1486.6940 7.62 59 - 71 0 R.TTPSYVAFTDTER.L
1542.7499 1541.7426 1541.7296 8.42 322 - 333 1 R.ARFEELCSDLFR.S
1579.8413 1578.8340 1578.8154 11.8 371 - 383 1 K.LLQDFFNGRDLNK.S
1630.8326 1629.8253 1629.7960 18.0 135 - 148 0 K.AFYPEEISSMVLTK.M + Oxidation (M)
1675.7352 1674.7279 1674.7234 2.70 243 - 258 0 K.ATAGDTHLGGEDFDNR.L
1687.9100 1686.9027 1686.8940 5.15 194 - 209 0 R.IINEPTAAAIAYGLDR.T
1814.9541 1813.9468 1813.9435 1.84 79 - 94 1 K.NQVALNPQNTVFDAKR.L
1822.0240 1821.0167 1821.0108 3.23 348 - 364 1 K.LDKAQIHDLVLVGGSTR.I
2769.3118 2768.3045 2768.2626 15.1 2 - 25 0 M.HHHHHHSSGVDLGTENLYFQSMAK.A
2774.3113 2773.3040 2773.3956 -33.02 380 - 406 1 R.DLNKSINPDEAVAYGAAVQAAILMGDK.S
2981.4358 2980.4285 2980.4553 -8.99 295 - 321 0 R.TLSSSTQASLEIDSLFEGIDFYTSITR.A
No match to: 855.0549, 887.0070, 927.4994, 1056.5944, 1110.5702, 1163.6331, 1232.5702, 1252.6086, 1253.6166, 1320.5953, 1326.6932,
1377.6681, 1439.8186,
1456.7246, 1463.7484, 1472.7527, 1473.8469, 1480.7639, 1537.8105, 1565.8301, 1567.7526, 1580.8241, 1638.8689, 1639.9293, 1642.8138,
1690.8757, 1691.8064,
1694.8363, 1710.8218, 1735.8954, 1782.8805, 1838.0254, 1880.9231, 1907.9373, 1915.9832, 1980.0317, 1982.0004, 1994.0042, 2045.0317,
2225.1553, 2289.1572,
2492.2678, 2545.2520, 2570.1982, 2588.2024, 2612.1765, 2786.3582, 2975.4814, 2997.4368, 3136.4468, 3153.4426
8. Mixture 7 Total score: 114 Expect: 4.2e-006 Matches: 23
Components: 1. gi|194388088 unnamed protein product [Homo sapiens]
2. gi|148693577 mCG5074, isoform CRA_a [Mus musculus]
Observed Mr(expt) Mr(calc) ppm Start End Miss Comp Peptide
1110.5702 1109.5629 1109.5506 11.1 294 - 302 0 1 K.LLQDFFDGR.D
1197.6906 1196.6833 1196.6877 -3.67 105 - 116 0 1 K.DAGVIAGLNVLR.I
1252.6086 1251.6013 1251.6533 -41.49 62 - 72 1 2 K.MKEIAEAYLGK.T
1253.6166 1252.6093 1252.6088 0.43 237 - 246 0 2 R.FEELNADLFR.G
1417.7695 1416.7622 1416.7514 7.66 182 - 192 1 1 R.LVNHFVEEFKR.K
1480.7639 1479.7566 1479.7470 6.51 235 - 246 1 2 R.ARFEELNADLFR.G
1487.7126 1486.7053 1486.6940 7.62 37 - 49 0 1 R.TTPSYVAFTDTER.L
1542.7499 1541.7426 1541.7296 8.42 245 - 256 1 1 R.ARFEELCSDLFR.S
1565.8301 1564.8228 1564.8249 -1.34 284 - 296 1 2 K.LLQDFFNGKELNK.S
1580.8241 1579.8168 1579.7995 11.0 294 - 306 1 1 K.LLQDFFDGRDLNK.S
1675.7352 1674.7279 1674.7234 2.70 166 - 181 0 1 K.ATAGDTHLGGEDFDNR.L
1687.9100 1686.9027 1686.8940 5.15 117 - 132 0 1 R.IINEPTAAAIAYGLDR.T
1691.8064 1690.7991 1690.7183 47.8 156 - 171 0 2 K.STAGDTHLGGEDFDNR.M
1814.9541 1813.9468 1813.9435 1.84 57 - 72 1 1 K.NQVALNPQNTVFDAKR.L
1822.0240 1821.0167 1821.0108 3.23 271 - 287 1 1 K.LDKAQIHDLVLVGGSTR.I
1838.0254 1837.0181 1837.0058 6.73 261 - 277 1 2 K.LDKSQIHDIVLVGGSTR.I
1907.9373 1906.9300 1906.9056 12.8 48 - 63 1 2 K.SFYPEEVSSMVLTKMK.E + 2 Oxidation (M)
1982.0004 1980.9931 1980.9905 1.31 73 - 90 0 2 K.TVTNAVVTVPAYFNDSQR.Q
2774.3113 2773.3040 2773.3956 -33.02 303 - 329 1 1 R.DLNKSINPDEAVAYGAAVQAAILMGDK.S
2786.3582 2785.3509 2785.3559 -1.79 369 - 392 0 1 K.QTQIFTTYSDNQPGVLIQVYEGER.A
2981.4358 2980.4285 2980.4553 -8.99 218 - 244 0 1 R.TLSSSTQASLEIDSLFEGIDFYTSITR.A
2997.4368 2996.4295 2996.4502 -6.91 208 - 234 0 2 R.TLSSSTQASIEIDSLYEGIDFYTSITR.A
3153.4426 3152.4353 3152.5513 -36.80 207 - 234 1 2 K.RTLSSSTQASIEIDSLYEGIDFYTSITR.A
No match to: 855.0549, 887.0070, 927.4994, 1056.5944, 1109.5812, 1163.6331, 1232.5702, 1320.5953, 1326.6932, 1377.6681, 1439.8186,
1456.7246, 1463.7484,
1472.7527, 1473.8469, 1537.8105, 1567.7526, 1579.8413, 1630.8326, 1638.8689, 1639.9293, 1642.8138, 1690.8757, 1694.8363, 1710.8218,
1735.8954, 1782.8805,
1880.9231, 1915.9832, 1980.0317, 1994.0042, 2045.0317, 2225.1553, 2289.1572, 2492.2678, 2545.2520, 2570.1982, 2588.2024, 2612.1765,
2769.3118, 2975.4814,

3136.4468
9. Mixture 8 Total score: 109 Expect: 1.3e-005 Matches: 23
Components: 1. gi|6016261 RecName: Full=Heat shock 70 kDa protein 1
2. gi|148693577 mCG5074, isoform CRA_a [Mus musculus]
Observed Mr(expt) Mr(calc) ppm Start End Miss Comp Peptide
1109.5812 1108.5739 1108.5665 6.66 347 - 355 0 1 K.LLQDFFNGR.D
1197.6906 1196.6833 1196.6877 -3.67 160 - 171 0 1 K.DAGVIAGLNVLR.I
1252.6086 1251.6013 1251.6533 -41.49 62 - 72 1 2 K.MKEIAEAYLGK.T
1253.6166 1252.6093 1252.6121 -2.23 537 - 547 0 1 K.NALESYALNMK.S
1417.7695 1416.7622 1416.7514 7.66 235 - 245 1 1 R.LVNHFVEEFKR.K
1480.7639 1479.7566 1479.7470 6.51 235 - 246 1 2 R.ARFEELNADLFR.G
1487.7126 1486.7053 1486.6940 7.62 37 - 49 0 1 R.TTPSYVAFTDTER.L
1542.7499 1541.7426 1541.7296 8.42 298 - 309 1 1 R.ARFEELCSDLFR.S
1565.8301 1564.8228 1564.8249 -1.34 284 - 296 1 2 K.LLQDFFNGKELNK.S
1579.8413 1578.8340 1578.8154 11.8 347 - 359 1 1 K.LLQDFFNGRDLNK.S
1630.8326 1629.8253 1629.7960 18.0 113 - 126 0 1 K.AFYPEEISSMVLTK.M + Oxidation (M)
1638.8689 1637.8616 1637.8446 10.4 533 - 547 1 1 R.VSAKNALESYALNMK.S
1691.8064 1690.7991 1690.7183 47.8 156 - 171 0 2 K.STAGDTHLGGEDFDNR.M
1814.9541 1813.9468 1813.9435 1.84 57 - 72 1 1 K.NQVALNPQNTVFDAKR.L
1822.0240 1821.0167 1821.0108 3.23 324 - 340 1 1 K.LDKAQIHDLVLVGGSTR.I
1838.0254 1837.0181 1837.0058 6.73 261 - 277 1 2 K.LDKSQIHDIVLVGGSTR.I
1907.9373 1906.9300 1906.9056 12.8 48 - 63 1 2 K.SFYPEEVSSMVLTKMK.E + 2 Oxidation (M)
1982.0004 1980.9931 1980.9905 1.31 73 - 90 0 2 K.TVTNAVVTVPAYFNDSQR.Q
2774.3113 2773.3040 2773.3956 -33.02 356 - 382 1 1 R.DLNKSINPDEAVAYGAAVQAAILMGDK.S
2786.3582 2785.3509 2785.3559 -1.79 422 - 445 0 1 K.QTQIFTTYSDNQPGVLIQVYEGER.A
2981.4358 2980.4285 2980.4553 -8.99 271 - 297 0 1 R.TLSSSTQASLEIDSLFEGIDFYTSITR.A
2997.4368 2996.4295 2996.4502 -6.91 208 - 234 0 2 R.TLSSSTQASIEIDSLYEGIDFYTSITR.A
3153.4426 3152.4353 3152.5513 -36.80 207 - 234 1 2 K.RTLSSSTQASIEIDSLYEGIDFYTSITR.A
No match to: 855.0549, 887.0070, 927.4994, 1056.5944, 1110.5702, 1163.6331, 1232.5702, 1320.5953, 1326.6932, 1377.6681, 1439.8186,
1456.7246, 1463.7484,
1472.7527, 1473.8469, 1537.8105, 1567.7526, 1580.8241, 1639.9293, 1642.8138, 1675.7352, 1687.9100, 1690.8757, 1694.8363, 1710.8218,
1735.8954, 1782.8805,
1880.9231, 1915.9832, 1980.0317, 1994.0042, 2045.0317, 2225.1553, 2289.1572, 2492.2678, 2545.2520, 2570.1982, 2588.2024, 2612.1765,
2769.3118, 2975.4814,
3136.4468
10. gi|2495339 Mass: 70470 Score: 109 Expect: 1.3e-005 Matches: 15
RecName: Full=Heat shock 70 kDa protein 1B; AltName: Full=Heat shock 70 kDa protein 2; Short=HSP70.2
Observed Mr(expt) Mr(calc) ppm Start End Miss Peptide
1109.5812 1108.5739 1108.5665 6.66 349 - 357 0 K.LLQDFFNGR.D
1197.6906 1196.6833 1196.6877 -3.67 160 - 171 0 K.DAGVIAGLNVLR.I
1417.7695 1416.7622 1416.7514 7.66 237 - 247 1 R.LVNHFVEEFKR.K
1487.7126 1486.7053 1486.6940 7.62 37 - 49 0 R.TTPSYVAFTDTER.L
1542.7499 1541.7426 1541.7296 8.42 300 - 311 1 R.ARFEELCSDLFR.S
1579.8413 1578.8340 1578.8154 11.8 349 - 361 1 K.LLQDFFNGRDLNK.S
1630.8326 1629.8253 1629.7960 18.0 113 - 126 0 K.AFYPEEISSMVLTK.M + Oxidation (M)
1675.7352 1674.7279 1674.7234 2.70 221 - 236 0 K.ATAGDTHLGGEDFDNR.L
1687.9100 1686.9027 1686.8940 5.15 172 - 187 0 R.IINEPTAAAIAYGLDR.T
1814.9541 1813.9468 1813.9435 1.84 57 - 72 1 K.NQVALNPQNTVFDAKR.L
1822.0240 1821.0167 1821.0108 3.23 326 - 342 1 K.LDKAQIHDLVLVGGSTR.I
2774.3113 2773.3040 2773.3956 -33.02 358 - 384 1 R.DLNKSINPDEAVAYGAAVQAAILMGDK.S
2786.3582 2785.3509 2785.3559 -1.79 424 - 447 0 K.QTQIFTTYSDNQPGVLIQVYEGER.A
2975.4814 2974.4741 2974.4825 -2.81 129 - 155 0 K.EIAEAYLGHPVTNAVITVPAYFNDSQR.Q
2981.4358 2980.4285 2980.4553 -8.99 273 - 299 0 R.TLSSSTQASLEIDSLFEGIDFYTSITR.A
No match to: 855.0549, 887.0070, 927.4994, 1056.5944, 1110.5702, 1163.6331, 1232.5702, 1252.6086, 1253.6166, 1320.5953, 1326.6932,
1377.6681, 1439.8186,
1456.7246, 1463.7484, 1472.7527, 1473.8469, 1480.7639, 1537.8105, 1565.8301, 1567.7526, 1580.8241, 1638.8689, 1639.9293, 1642.8138,
1690.8757, 1691.8064,
1694.8363, 1710.8218, 1735.8954, 1782.8805, 1838.0254, 1880.9231, 1907.9373, 1915.9832, 1980.0317, 1982.0004, 1994.0042, 2045.0317,
2225.1553, 2289.1572,
2492.2678, 2545.2520, 2570.1982, 2588.2024, 2612.1765, 2769.3118, 2997.4368, 3136.4468, 3153.4426
11. gi|56757663 Mass: 70500 Score: 109 Expect: 1.3e-005 Matches: 15
RecName: Full=Heat shock 70 kDa protein 1A; AltName: Full=Heat shock 70 kDa protein 1; Short=HSP70.1
Observed Mr(expt) Mr(calc) ppm Start End Miss Peptide
1109.5812 1108.5739 1108.5665 6.66 349 - 357 0 K.LLQDFFNGR.D
1197.6906 1196.6833 1196.6877 -3.67 160 - 171 0 K.DAGVIAGLNVLR.I
1417.7695 1416.7622 1416.7514 7.66 237 - 247 1 R.LVNHFVEEFKR.K
1487.7126 1486.7053 1486.6940 7.62 37 - 49 0 R.TTPSYVAFTDTER.L
1542.7499 1541.7426 1541.7296 8.42 300 - 311 1 R.ARFEELCSDLFR.S
1579.8413 1578.8340 1578.8154 11.8 349 - 361 1 K.LLQDFFNGRDLNK.S
1630.8326 1629.8253 1629.7960 18.0 113 - 126 0 K.AFYPEEISSMVLTK.M + Oxidation (M)
1675.7352 1674.7279 1674.7234 2.70 221 - 236 0 K.ATAGDTHLGGEDFDNR.L
1687.9100 1686.9027 1686.8940 5.15 172 - 187 0 R.IINEPTAAAIAYGLDR.T
1814.9541 1813.9468 1813.9435 1.84 57 - 72 1 K.NQVALNPQNTVFDAKR.L
1822.0240 1821.0167 1821.0108 3.23 326 - 342 1 K.LDKAQIHDLVLVGGSTR.I
2774.3113 2773.3040 2773.3956 -33.02 358 - 384 1 R.DLNKSINPDEAVAYGAAVQAAILMGDK.S
2786.3582 2785.3509 2785.3559 -1.79 424 - 447 0 K.QTQIFTTYSDNQPGVLIQVYEGER.A
2975.4814 2974.4741 2974.4825 -2.81 129 - 155 0 K.EIAEAYLGHPVTNAVITVPAYFNDSQR.Q
2981.4358 2980.4285 2980.4553 -8.99 273 - 299 0 R.TLSSSTQASLEIDSLFEGIDFYTSITR.A
No match to: 855.0549, 887.0070, 927.4994, 1056.5944, 1110.5702, 1163.6331, 1232.5702, 1252.6086, 1253.6166, 1320.5953, 1326.6932,
1377.6681, 1439.8186,
1456.7246, 1463.7484, 1472.7527, 1473.8469, 1480.7639, 1537.8105, 1565.8301, 1567.7526, 1580.8241, 1638.8689, 1639.9293, 1642.8138,
1690.8757, 1691.8064,
1694.8363, 1710.8218, 1735.8954, 1782.8805, 1838.0254, 1880.9231, 1907.9373, 1915.9832, 1980.0317, 1982.0004, 1994.0042, 2045.0317,
2225.1553, 2289.1572,
2492.2678, 2545.2520, 2570.1982, 2588.2024, 2612.1765, 2769.3118, 2997.4368, 3136.4468, 3153.4426
12. gi|40254806 Mass: 70492 Score: 109 Expect: 1.3e-005 Matches: 15
heat shock 70 kDa protein 1A [Bos taurus]
Observed Mr(expt) Mr(calc) ppm Start End Miss Peptide
1109.5812 1108.5739 1108.5665 6.66 349 - 357 0 K.LLQDFFNGR.D
1197.6906 1196.6833 1196.6877 -3.67 160 - 171 0 K.DAGVIAGLNVLR.I
1417.7695 1416.7622 1416.7514 7.66 237 - 247 1 R.LVNHFVEEFKR.K
1487.7126 1486.7053 1486.6940 7.62 37 - 49 0 R.TTPSYVAFTDTER.L

1542.7499 1541.7426 1541.7296 8.42 300 - 311 1 R.ARFEELCSDLFR.S
1579.8413 1578.8340 1578.8154 11.8 349 - 361 1 K.LLQDFFNGRDLNK.S
1630.8326 1629.8253 1629.7960 18.0 113 - 126 0 K.AFYPEEISSMVLTK.M + Oxidation (M)
1675.7352 1674.7279 1674.7234 2.70 221 - 236 0 K.ATAGDTHLGGEDFDNR.L
1687.9100 1686.9027 1686.8940 5.15 172 - 187 0 R.IINEPTAAAIAYGLDR.T
1814.9541 1813.9468 1813.9435 1.84 57 - 72 1 K.NQVALNPQNTVFDAKR.L
1822.0240 1821.0167 1821.0108 3.23 326 - 342 1 K.LDKAQIHDLVLVGGSTR.I
2774.3113 2773.3040 2773.3956 -33.02 358 - 384 1 R.DLNKSINPDEAVAYGAAVQAAILMGDK.S
2786.3582 2785.3509 2785.3559 -1.79 424 - 447 0 K.QTQIFTTYSDNQPGVLIQVYEGER.A
2975.4814 2974.4741 2974.4825 -2.81 129 - 155 0 K.EIAEAYLGHPVTNAVITVPAYFNDSQR.Q
2981.4358 2980.4285 2980.4553 -8.99 273 - 299 0 R.TLSSSTQASLEIDSLFEGIDFYTSITR.A
No match to: 855.0549, 887.0070, 927.4994, 1056.5944, 1110.5702, 1163.6331, 1232.5702, 1252.6086, 1253.6166, 1320.5953, 1326.6932, 1377.6681, 1439.8186,
1456.7246, 1463.7484, 1472.7527, 1473.8469, 1480.7639, 1537.8105, 1565.8301, 1567.7526, 1580.8241, 1638.8689, 1639.9293, 1642.8138, 1690.8757, 1691.8064,
1694.8363, 1710.8218, 1735.8954, 1782.8805, 1838.0254, 1880.9231, 1907.9373, 1915.9832, 1980.0317, 1982.0004, 1994.0042, 2045.0317, 2225.1553, 2289.1572,
2492.2678, 2545.2520, 2570.1982, 2588.2024, 2612.1765, 2769.3118, 2997.4368, 3136.4468, 3153.4426
13. gi|312064069 Mass: 70514 Score: 109 Expect: 1.3e-005 Matches: 15
heat shock 70 kDa protein 1A [Bos indicus]
Observed Mr(expt) Mr(calc) ppm Start End Miss Peptide
1109.5812 1108.5739 1108.5665 6.66 349 - 357 0 K.LLQDFFNGR.D
1197.6906 1196.6833 1196.6877 -3.67 160 - 171 0 K.DAGVIAGLNVLR.I
1417.7695 1416.7622 1416.7514 7.66 237 - 247 1 R.LVNHFVEEFKR.K
1487.7126 1486.7053 1486.6940 7.62 37 - 49 0 R.TTPSYVAFTDTER.L
1542.7499 1541.7426 1541.7296 8.42 300 - 311 1 R.ARFEELCSDLFR.S
1579.8413 1578.8340 1578.8154 11.8 349 - 361 1 K.LLQDFFNGRDLNK.S
1630.8326 1629.8253 1629.7960 18.0 113 - 126 0 K.AFYPEEISSMVLTK.M + Oxidation (M)
1675.7352 1674.7279 1674.7234 2.70 221 - 236 0 K.ATAGDTHLGGEDFDNR.L
1687.9100 1686.9027 1686.8940 5.15 172 - 187 0 R.IINEPTAAAIAYGLDR.T
1814.9541 1813.9468 1813.9435 1.84 57 - 72 1 K.NQVALNPQNTVFDAKR.L
1822.0240 1821.0167 1821.0108 3.23 326 - 342 1 K.LDKAQIHDLVLVGGSTR.I
2774.3113 2773.3040 2773.3956 -33.02 358 - 384 1 R.DLNKSINPDEAVAYGAAVQAAILMGDK.S
2786.3582 2785.3509 2785.3559 -1.79 424 - 447 0 K.QTQIFTTYSDNQPGVLIQVYEGER.A
2975.4814 2974.4741 2974.4825 -2.81 129 - 155 0 K.EIAEAYLGHPVTNAVITVPAYFNDSQR.Q
2981.4358 2980.4285 2980.4553 -8.99 273 - 299 0 R.TLSSSTQASLEIDSLFEGIDFYTSITR.A
No match to: 855.0549, 887.0070, 927.4994, 1056.5944, 1110.5702, 1163.6331, 1232.5702, 1252.6086, 1253.6166, 1320.5953, 1326.6932, 1377.6681, 1439.8186,
1456.7246, 1463.7484, 1472.7527, 1473.8469, 1480.7639, 1537.8105, 1565.8301, 1567.7526, 1580.8241, 1638.8689, 1639.9293, 1642.8138, 1690.8757, 1691.8064,
1694.8363, 1710.8218, 1735.8954, 1782.8805, 1838.0254, 1880.9231, 1907.9373, 1915.9832, 1980.0317, 1982.0004, 1994.0042, 2045.0317, 2225.1553, 2289.1572,
2492.2678, 2545.2520, 2570.1982, 2588.2024, 2612.1765, 2769.3118, 2997.4368, 3136.4468, 3153.4426
14. gi|332078832 Mass: 70385 Score: 109 Expect: 1.3e-005 Matches: 15
heat shock protein 70 1A [Camelus dromedarius]
Observed Mr(expt) Mr(calc) ppm Start End Miss Peptide
1109.5812 1108.5739 1108.5665 6.66 349 - 357 0 K.LLQDFFNGR.D
1197.6906 1196.6833 1196.6877 -3.67 160 - 171 0 K.DAGVIAGLNVLR.I
1417.7695 1416.7622 1416.7514 7.66 237 - 247 1 R.LVNHFVEEFKR.K
1487.7126 1486.7053 1486.6940 7.62 37 - 49 0 R.TTPSYVAFTDTER.L
1542.7499 1541.7426 1541.7296 8.42 300 - 311 1 R.ARFEELCSDLFR.S
1579.8413 1578.8340 1578.8154 11.8 349 - 361 1 K.LLQDFFNGRDLNK.S
1630.8326 1629.8253 1629.7960 18.0 113 - 126 0 K.AFYPEEISSMVLTK.M + Oxidation (M)
1675.7352 1674.7279 1674.7234 2.70 221 - 236 0 K.ATAGDTHLGGEDFDNR.L
1687.9100 1686.9027 1686.8940 5.15 172 - 187 0 R.IINEPTAAAIAYGLDR.T
1814.9541 1813.9468 1813.9435 1.84 57 - 72 1 K.NQVALNPQNTVFDAKR.L
1822.0240 1821.0167 1821.0108 3.23 326 - 342 1 K.LDKAQIHDLVLVGGSTR.I
2774.3113 2773.3040 2773.3956 -33.02 358 - 384 1 R.DLNKSINPDEAVAYGAAVQAAILMGDK.S
2786.3582 2785.3509 2785.3559 -1.79 424 - 447 0 K.QTQIFTTYSDNQPGVLIQVYEGER.A
2975.4814 2974.4741 2974.4825 -2.81 129 - 155 0 K.EIAEAYLGHPVTNAVITVPAYFNDSQR.Q
2981.4358 2980.4285 2980.4553 -8.99 273 - 299 0 R.TLSSSTQASLEIDSLFEGIDFYTSITR.A
No match to: 855.0549, 887.0070, 927.4994, 1056.5944, 1110.5702, 1163.6331, 1232.5702, 1252.6086, 1253.6166, 1320.5953, 1326.6932, 1377.6681, 1439.8186,
1456.7246, 1463.7484, 1472.7527, 1473.8469, 1480.7639, 1537.8105, 1565.8301, 1567.7526, 1580.8241, 1638.8689, 1639.9293, 1642.8138, 1690.8757, 1691.8064,
1694.8363, 1710.8218, 1735.8954, 1782.8805, 1838.0254, 1880.9231, 1907.9373, 1915.9832, 1980.0317, 1982.0004, 1994.0042, 2045.0317, 2225.1553, 2289.1572,
2492.2678, 2545.2520, 2570.1982, 2588.2024, 2612.1765, 2769.3118, 2997.4368, 3136.4468, 3153.4426
15. gi|334904119 Mass: 70471 Score: 109 Expect: 1.3e-005 Matches: 15
heat shock protein 70.1 [Capra hircus]
Observed Mr(expt) Mr(calc) ppm Start End Miss Peptide
1109.5812 1108.5739 1108.5665 6.66 349 - 357 0 K.LLQDFFNGR.D
1197.6906 1196.6833 1196.6877 -3.67 160 - 171 0 K.DAGVIAGLNVLR.I
1417.7695 1416.7622 1416.7514 7.66 237 - 247 1 R.LVNHFVEEFKR.K
1487.7126 1486.7053 1486.6940 7.62 37 - 49 0 R.TTPSYVAFTDTER.L
1542.7499 1541.7426 1541.7296 8.42 300 - 311 1 R.ARFEELCSDLFR.S
1579.8413 1578.8340 1578.8154 11.8 349 - 361 1 K.LLQDFFNGRDLNK.S
1630.8326 1629.8253 1629.7960 18.0 113 - 126 0 K.AFYPEEISSMVLTK.M + Oxidation (M)
1675.7352 1674.7279 1674.7234 2.70 221 - 236 0 K.ATAGDTHLGGEDFDNR.L
1687.9100 1686.9027 1686.8940 5.15 172 - 187 0 R.IINEPTAAAIAYGLDR.T
1814.9541 1813.9468 1813.9435 1.84 57 - 72 1 K.NQVALNPQNTVFDAKR.L
1822.0240 1821.0167 1821.0108 3.23 326 - 342 1 K.LDKAQIHDLVLVGGSTR.I
2774.3113 2773.3040 2773.3956 -33.02 358 - 384 1 R.DLNKSINPDEAVAYGAAVQAAILMGDK.S
2786.3582 2785.3509 2785.3559 -1.79 424 - 447 0 K.QTQIFTTYSDNQPGVLIQVYEGER.A
2975.4814 2974.4741 2974.4825 -2.81 129 - 155 0 K.EIAEAYLGHPVTNAVITVPAYFNDSQR.Q
2981.4358 2980.4285 2980.4553 -8.99 273 - 299 0 R.TLSSSTQASLEIDSLFEGIDFYTSITR.A
No match to: 855.0549, 887.0070, 927.4994, 1056.5944, 1110.5702, 1163.6331, 1232.5702, 1252.6086, 1253.6166, 1320.5953, 1326.6932, 1377.6681, 1439.8186,
1456.7246, 1463.7484, 1472.7527, 1473.8469, 1480.7639, 1537.8105, 1565.8301, 1567.7526, 1580.8241, 1638.8689, 1639.9293, 1642.8138, 1690.8757, 1691.8064,
1694.8363, 1710.8218, 1735.8954, 1782.8805, 1838.0254, 1880.9231, 1907.9373, 1915.9832, 1980.0317, 1982.0004, 1994.0042, 2045.0317, 2225.1553, 2289.1572,

2492.2678, 2545.2520, 2570.1982, 2588.2024, 2612.1765, 2769.3118, 2997.4368, 3136.4468, 3153.4426
16. gi|343432731 Mass: 70485 Score: 109 Expect: 1.3e-005 Matches: 15
heat shock protein 70.1 [Capra hircus]
Observed Mr(expt) Mr(calc) ppm Start End Miss Peptide
1109.5812 1108.5739 1108.5665 6.66 349 - 357 0 K.LLQDFFNGR.D
1197.6906 1196.6833 1196.6877 -3.67 160 - 171 0 K.DAGVIAGLNVLR.I
1417.7695 1416.7622 1416.7514 7.66 237 - 247 1 R.LVNHFVEEFKR.K
1487.7126 1486.7053 1486.6940 7.62 37 - 49 0 R.TTPSYVAFTDTER.L
1542.7499 1541.7426 1541.7296 8.42 300 - 311 1 R.ARFEELCSDLFR.S
1579.8413 1578.8340 1578.8154 11.8 349 - 361 1 K.LLQDFFNGRDLNK.S
1630.8326 1629.8253 1629.7960 18.0 113 - 126 0 K.AFYPEEISSMVLTK.M + Oxidation (M)
1675.7352 1674.7279 1674.7234 2.70 221 - 236 0 K.ATAGDTHLGGEDFDNR.L
1687.9100 1686.9027 1686.8940 5.15 172 - 187 0 R.IINEPTAAAIAYGLDR.T
1814.9541 1813.9468 1813.9435 1.84 57 - 72 1 K.NQVALNPQNTVFDAKR.L
1822.0240 1821.0167 1821.0108 3.23 326 - 342 1 K.LDKAQIHDLVLVGGSTR.I
2774.3113 2773.3040 2773.3956 -33.02 358 - 384 1 R.DLNKSINPDEAVAYGAAVQAAILMGDK.S
2786.3582 2785.3509 2785.3559 -1.79 424 - 447 0 K.QTQIFTTYSDNQPGVLIQVYEGER.A
2975.4814 2974.4741 2974.4825 -2.81 129 - 155 0 K.EIAEAYLGHPVTNAVITVPAYFNDSQR.Q
2981.4358 2980.4285 2980.4553 -8.99 273 - 299 0 R.TLSSSTQASLEIDSLFEGIDFYTSITR.A
No match to: 855.0549, 887.0070, 927.4994, 1056.5944, 1110.5702, 1163.6331, 1232.5702, 1252.6086, 1253.6166, 1320.5953, 1326.6932, 1377.6681, 1439.8186,
1456.7246, 1463.7484, 1472.7527, 1473.8469, 1480.7639, 1537.8105, 1565.8301, 1567.7526, 1580.8241, 1638.8689, 1639.9293, 1642.8138, 1690.8757, 1691.8064,
1694.8363, 1710.8218, 1735.8954, 1782.8805, 1838.0254, 1880.9231, 1907.9373, 1915.9832, 1980.0317, 1982.0004, 1994.0042, 2045.0317, 2225.1553, 2289.1572,
2492.2678, 2545.2520, 2570.1982, 2588.2024, 2612.1765, 2769.3118, 2997.4368, 3136.4468, 3153.4426
17. gi|371767260 Mass: 70596 Score: 109 Expect: 1.3e-005 Matches: 15
heat shock protein 70 [Capra hircus]
Observed Mr(expt) Mr(calc) ppm Start End Miss Peptide
1109.5812 1108.5739 1108.5665 6.66 349 - 357 0 K.LLQDFFNGR.D
1197.6906 1196.6833 1196.6877 -3.67 160 - 171 0 K.DAGVIAGLNVLR.I
1417.7695 1416.7622 1416.7514 7.66 237 - 247 1 R.LVNHFVEEFKR.K
1487.7126 1486.7053 1486.6940 7.62 37 - 49 0 R.TTPSYVAFTDTER.L
1542.7499 1541.7426 1541.7296 8.42 300 - 311 1 R.ARFEELCSDLFR.S
1579.8413 1578.8340 1578.8154 11.8 349 - 361 1 K.LLQDFFNGRDLNK.S
1675.7352 1674.7279 1674.7234 2.70 221 - 236 0 K.ATAGDTHLGGEDFDNR.L
1690.8757 1689.8684 1689.8574 6.55 94 - 108 1 R.VINDGDEPKVQVSYK.G
1814.9541 1813.9468 1813.9435 1.84 57 - 72 1 K.NQVALNPQNTVFDAKR.L
1822.0240 1821.0167 1821.0108 3.23 326 - 342 1 K.LDKAQIHDLVLVGGSTR.I
1915.9832 1914.9759 1914.9835 -3.93 113 - 128 1 K.AFYPEEISLMVLTKMK.E + Oxidation (M)
2774.3113 2773.3040 2773.3956 -33.02 358 - 384 1 R.DLNKSINPDEAVAYGAAVQAAILMGDK.S
2786.3582 2785.3509 2785.3559 -1.79 424 - 447 0 K.QTQIFTTYSDNQPGVLIQVYEGER.A
2975.4814 2974.4741 2974.4825 -2.81 129 - 155 0 K.EIAEAYLGHPVTNAVITVPAYFNDSQR.Q
2981.4358 2980.4285 2980.4553 -8.99 273 - 299 0 R.TLSSSTQASLEIDSLFEGIDFYTSITR.A
No match to: 855.0549, 887.0070, 927.4994, 1056.5944, 1110.5702, 1163.6331, 1232.5702, 1252.6086, 1253.6166, 1320.5953, 1326.6932, 1377.6681, 1439.8186,
1456.7246, 1463.7484, 1472.7527, 1473.8469, 1480.7639, 1537.8105, 1565.8301, 1567.7526, 1580.8241, 1630.8326, 1638.8689, 1639.9293, 1642.8138, 1687.9100,
1691.8064, 1694.8363, 1710.8218, 1735.8954, 1782.8805, 1838.0254, 1880.9231, 1907.9373, 1980.0317, 1982.0004, 1994.0042, 2045.0317, 2225.1553, 2289.1572,
2492.2678, 2545.2520, 2570.1982, 2588.2024, 2612.1765, 2769.3118, 2997.4368, 3136.4468, 3153.4426
18. gi|166007012 Mass: 43182 Score: 109 Expect: 1.3e-005 Matches: 13
Chain A, Crystal Structure Of The Human Hsp70 Atpase Domain In The Apo Form
Observed Mr(expt) Mr(calc) ppm Start End Miss Peptide
1109.5812 1108.5739 1108.5665 6.66 352 - 360 0 K.LLQDFFNGR.D
1197.6906 1196.6833 1196.6877 -3.67 163 - 174 0 K.DAGVIAGLNVLR.I
1417.7695 1416.7622 1416.7514 7.66 240 - 250 1 R.LVNHFVEEFKR.K
1487.7126 1486.7053 1486.6940 7.62 40 - 52 0 R.TTPSYVAFTDTER.L
1542.7499 1541.7426 1541.7296 8.42 303 - 314 1 R.ARFEELCSDLFR.S
1579.8413 1578.8340 1578.8154 11.8 352 - 364 1 K.LLQDFFNGRDLNK.S
1630.8326 1629.8253 1629.7960 18.0 116 - 129 0 K.AFYPEEISSMVLTK.M + Oxidation (M)
1675.7352 1674.7279 1674.7234 2.70 224 - 239 0 K.ATAGDTHLGGEDFDNR.L
1687.9100 1686.9027 1686.8940 5.15 175 - 190 0 R.IINEPTAAAIAYGLDR.T
1814.9541 1813.9468 1813.9435 1.84 60 - 75 1 K.NQVALNPQNTVFDAKR.L
1822.0240 1821.0167 1821.0108 3.23 329 - 345 1 K.LDKAQIHDLVLVGGSTR.I
2774.3113 2773.3040 2773.3956 -33.02 361 - 387 1 R.DLNKSINPDEAVAYGAAVQAAILMGDK.S
2981.4358 2980.4285 2980.4553 -8.99 276 - 302 0 R.TLSSSTQASLEIDSLFEGIDFYTSITR.A
No match to: 855.0549, 887.0070, 927.4994, 1056.5944, 1110.5702, 1163.6331, 1232.5702, 1252.6086, 1253.6166, 1320.5953, 1326.6932, 1377.6681, 1439.8186,
1456.7246, 1463.7484, 1472.7527, 1473.8469, 1480.7639, 1537.8105, 1565.8301, 1567.7526, 1580.8241, 1638.8689, 1639.9293, 1642.8138, 1690.8757, 1691.8064,
1694.8363, 1710.8218, 1735.8954, 1782.8805, 1838.0254, 1880.9231, 1907.9373, 1915.9832, 1980.0317, 1982.0004, 1994.0042, 2045.0317, 2225.1553, 2289.1572,
2492.2678, 2545.2520, 2570.1982, 2588.2024, 2612.1765, 2769.3118, 2786.3582, 2975.4814, 2997.4368, 3136.4468, 3153.4426
19. gi|166007013 Mass: 43182 Score: 109 Expect: 1.3e-005 Matches: 13
Chain A, Crystal Structure Of The Human Hsp70 Atpase Domain In Complex With Amp-Pnp
Observed Mr(expt) Mr(calc) ppm Start End Miss Peptide
1109.5812 1108.5739 1108.5665 6.66 352 - 360 0 K.LLQDFFNGR.D
1197.6906 1196.6833 1196.6877 -3.67 163 - 174 0 K.DAGVIAGLNVLR.I
1417.7695 1416.7622 1416.7514 7.66 240 - 250 1 R.LVNHFVEEFKR.K
1487.7126 1486.7053 1486.6940 7.62 40 - 52 0 R.TTPSYVAFTDTER.L
1542.7499 1541.7426 1541.7296 8.42 303 - 314 1 R.ARFEELCSDLFR.S
1579.8413 1578.8340 1578.8154 11.8 352 - 364 1 K.LLQDFFNGRDLNK.S
1630.8326 1629.8253 1629.7960 18.0 116 - 129 0 K.AFYPEEISSMVLTK.M + Oxidation (M)
1675.7352 1674.7279 1674.7234 2.70 224 - 239 0 K.ATAGDTHLGGEDFDNR.L
1687.9100 1686.9027 1686.8940 5.15 175 - 190 0 R.IINEPTAAAIAYGLDR.T
1814.9541 1813.9468 1813.9435 1.84 60 - 75 1 K.NQVALNPQNTVFDAKR.L
1822.0240 1821.0167 1821.0108 3.23 329 - 345 1 K.LDKAQIHDLVLVGGSTR.I
2774.3113 2773.3040 2773.3956 -33.02 361 - 387 1 R.DLNKSINPDEAVAYGAAVQAAILMGDK.S
2981.4358 2980.4285 2980.4553 -8.99 276 - 302 0 R.TLSSSTQASLEIDSLFEGIDFYTSITR.A
No match to: 855.0549, 887.0070, 927.4994, 1056.5944, 1110.5702, 1163.6331, 1232.5702, 1252.6086, 1253.6166, 1320.5953, 1326.6932, 1377.6681, 1439.8186,

1456.7246, 1463.7484, 1472.7527, 1473.8469, 1480.7639, 1537.8105, 1565.8301, 1567.7526, 1580.8241, 1638.8689, 1639.9293, 1642.8138, 1690.8757, 1691.8064,
1694.8363, 1710.8218, 1735.8954, 1782.8805, 1838.0254, 1880.9231, 1907.9373, 1915.9832, 1980.0317, 1982.0004, 1994.0042, 2045.0317, 2225.1553, 2289.1572,
2492.2678, 2545.2520, 2570.1982, 2588.2024, 2612.1765, 2769.3118, 2786.3582, 2975.4814, 2997.4368, 3136.4468, 3153.4426
20. gi|292659561 Mass: 43239 Score: 109 Expect: 1.3e-005 Matches: 13
Chain A, Crystal Structure Of The Complex Between The Bag5 Bd5 And Hsp70 Nbd
Observed Mr(expt) Mr(calc) ppm Start End Miss Peptide
1109.5812 1108.5739 1108.5665 6.66 353 - 361 0 K.LLQDFFNGR.D
1197.6906 1196.6833 1196.6877 -3.67 164 - 175 0 K.DAGVIAGLNVLR.I
1417.7695 1416.7622 1416.7514 7.66 241 - 251 1 R.LVNHFVEEFKR.K
1487.7126 1486.7053 1486.6940 7.62 41 - 53 0 R.TTPSYVAFTDTER.L
1542.7499 1541.7426 1541.7296 8.42 304 - 315 1 R.ARFEELCSDLFR.S
1579.8413 1578.8340 1578.8154 11.8 353 - 365 1 K.LLQDFFNGRDLNK.S
1630.8326 1629.8253 1629.7960 18.0 117 - 130 0 K.AFYPEEISSMVLTK.M + Oxidation (M)
1675.7352 1674.7279 1674.7234 2.70 225 - 240 0 K.ATAGDTHLGGEDFDNR.L
1687.9100 1686.9027 1686.8940 5.15 176 - 191 0 R.IINEPTAAAIAYGLDR.T
1814.9541 1813.9468 1813.9435 1.84 61 - 76 1 K.NQVALNPQNTVFDAKR.L
1822.0240 1821.0167 1821.0108 3.23 330 - 346 1 K.LDKAQIHDLVLVGGSTR.I
2774.3113 2773.3040 2773.3956 -33.02 362 - 388 1 R.DLNKSINPDEAVAYGAAVQAAILMGDK.S
2981.4358 2980.4285 2980.4553 -8.99 277 - 303 0 R.TLSSSTQASLEIDSLFEGIDFYTSITR.A
No match to: 855.0549, 887.0070, 927.4994, 1056.5944, 1110.5702, 1163.6331, 1232.5702, 1252.6086, 1253.6166, 1320.5953, 1326.6932, 1377.6681, 1439.8186,
1456.7246, 1463.7484, 1472.7527, 1473.8469, 1480.7639, 1537.8105, 1565.8301, 1567.7526, 1580.8241, 1638.8689, 1639.9293, 1642.8138, 1690.8757, 1691.8064,
1694.8363, 1710.8218, 1735.8954, 1782.8805, 1838.0254, 1880.9231, 1907.9373, 1915.9832, 1980.0317, 1982.0004, 1994.0042, 2045.0317, 2225.1553, 2289.1572,
2492.2678, 2545.2520, 2570.1982, 2588.2024, 2612.1765, 2769.3118, 2786.3582, 2975.4814, 2997.4368, 3136.4468, 3153.4426

Search Parameters

Type of search : Peptide Mass Fingerprint
Enzyme : Trypsin
Fixed modifications : Carbamidomethyl (C)
Variable modifications : Oxidation (M)
Mass values : Monoisotopic
Protein Mass : Unrestricted
Peptide Mass Tolerance : ± 50 ppm
Peptide Charge State : 1+
Max Missed Cleavages : 1
Number of queries : 65

Mascot: http://www.matrixscience.com/

COVERAGE BAND 5

MATRIX SCIENCE Mascot Search Results

Protein View

Match to: gi|261825070 Score: 121 Expect: 8.4e-007
Chain A, Crystal Structure Of The Human 70kda Heat Shock Protein 1a (Hsp70-1) Atpase Domain In Complex With Adp And Inorganic Phosphate
Nominal mass (M_r): 45299; Calculated pI value: 6.33
NCBI BLAST search of gi|261825070 against nr
Unformatted sequence string for pasting into other applications
Taxonomy: Homo sapiens
Fixed modifications: Carbamidomethyl (C)
Variable modifications: Oxidation (M)
Cleavage by Trypsin: cuts C-term side of KR unless next residue is P
Number of mass values searched: 65
Number of mass values matched: 14
Sequence Coverage: 52%
Matched peptides shown in **Bold Red**

```
1 MHHHHHHSSG VDLGTENLYF QSMAKAAAIG IDLGTTYSCV GVFQHGKVEI
51 IANDQGNRTT PSYVAFTDTE RLIGDAAKNQ VALNPQNTVF DAKRLIGRKF
101 GDPVVQSDMK HWPFQVINDG DKPKVQVSYK GETKAFYPEE ISSMVLTKMK
151 EIAEAYLGYP VTNAVITVPA YFNDSQRQAT KDAGVIAGLN VLRIINEPTA
201 AAIAYGLDRT GKGERNVLIF DLGGGTFDVS ILTIDDGIFE VKATAGDTHL
251 GGEDFDNRLV NHFVEEFKRK HKKDISQNKR AVRRLRTACE RAKRTLSSST
301 QASLEIDSLF EGIDFYTSIT RARFEELCSD LFRSTLEPVE KALRDAKLDK
351 AQIHDLVLVG GSTRIPKVQK LLQDFFNGRD LNKSINPDEA VAYGAAVQAA
401 ILMGDKSEN
```

Show predicted peptides also
Sort Peptides By Residue Number Increasing Mass Decreasing Mass

```
Start - End Observed Mr(expt) Mr(calc) ppm Miss Sequence
2 - 25 2769.3118 2768.3045 2768.2626 15 0 M.HHHHHHSSGVDLGTENLYFQSMAK.A
59 - 71 1487.7126 1486.7053 1486.6940 8 0 R.TTPSYVAFTDTER.L
79 - 94 1814.9541 1813.9468 1813.9435 2 1 K.NQVALNPQNTVFDAKR.L
135 - 148 1630.8326 1629.8253 1629.7960 18 0 K.AFYPEEISSMVLTK.M Oxidation (M)
182 - 193 1197.6906 1196.6833 1196.6877 -4 0 K.DAGVIAGLNVLR.I
194 - 209 1687.9100 1686.9027 1686.8940 5 0 R.IINEPTAAAIAYGLDR.T
243 - 258 1675.7352 1674.7279 1674.7234 3 0 K.ATAGDTHLGGEDFDNR.L
259 - 269 1417.7695 1416.7622 1416.7514 8 1 R.LVNHFVEEFKR.K
295 - 321 2981.4358 2980.4285 2980.4553 -9 0 R.TLSSSTQASLEIDSLFEGIDFYTSITR.A
322 - 333 1542.7499 1541.7426 1541.7296 8 1 R.ARFEELCSDLFR.S
```

348 - 364 1822.0240 1821.0167 1821.0108 3 1 K.LDKAQIHDLVLVGGSTR.I
371 - 379 1109.5812 1108.5739 1108.5665 7 0 K.LLQDFFNGR.D
371 - 383 1579.8413 1578.8340 1578.8154 12 1 K.LLQDFFNGRDLNK.S
380 - 406 2774.3113 2773.3040 2773.3956 -33 1 R.DLNKSINPDEAVAYGAAVQAAILMGDK.S
No match to: 855.0549, 887.0070, 927.4994, 1056.5944, 1110.5702, 1163.6331, 1232.5702, 1252.6086, 1253.6166, 1320.5953, 1326.693

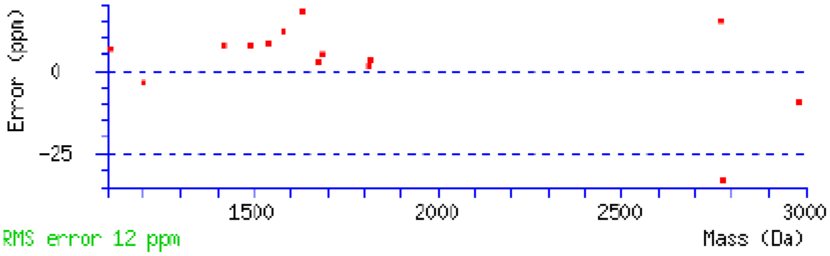

BAND 7

Mascot Search Results

Mascot Search Results

User :
Email :
Search title : SampleSetID: 824, AnalysisID: 7242, MaldiWellID: 69610, SpectrumID: 154535, Path=\180719\MS\18-106 NCBI Mammalia
Database : NCBInr 20120508 (17919084 sequences; 6150218869 residues)
Taxonomy : Mammalia (mammals) (1061927 sequences)
Timestamp : 19 Jul 2018 at 12:09:08 GMT
Top Score : 151 for gi|329664500, pyruvate kinase isozymes M1/M2 [Bos taurus]

Mascot Score Histogram

Protein score is -10*Log(P), where P is the probability that the observed match is a random event.
Protein scores greater than 73 are significant (p<0.05).

Protein Summary Report

Format As Protein Summary Help
Significance threshold p< 0.05 Max. number of hits 20
Re-Search All Search Unmatched

Index

Accession Mass Score Description
1. gi|329664500 58482 151 pyruvate kinase isozymes M1/M2 [Bos taurus]
2. gi|73587283 62016 146 PKM2 protein [Bos taurus]
3. gi|359807367 58461 141 pyruvate kinase isozymes M1/M2 isoform 2 [Mus musculus]
4. gi|1405933 58448 135 M2-type pyruvate kinase [Mus musculus]
5. gi|74221210 58379 123 unnamed protein product [Mus musculus]
6. gi|551295 58394 123 pyruvate kinase M [Mus musculus]
7. gi|31981562 58378 122 pyruvate kinase isozymes M1/M2 isoform 1 [Mus musculus]
8. gi|74151988 58390 122 unnamed protein product [Mus musculus]
9. gi|74196318 58377 112 unnamed protein product [Mus musculus]
10. gi|74222653 58320 112 unnamed protein product [Mus musculus]
11. gi|74212815 43537 108 unnamed protein product [Mus musculus]
12. gi|227908865 58465 94 pyruvate kinase isozymes M1/M2 isoform M1 [Equus caballus]
13. gi|219689076 58359 88 pyruvate kinase isozymes M1/M2 isoform M2 [Equus caballus]
14. gi|109157779 58365 86 Chain A, The Location Of The Allosteric Amino Acid Binding Site Of Muscle Pyruvate Kinase.
15. gi|3659945 58393 86 Chain A, Pyruvate Kinase From Rabbit Muscle With Mg, K, And L- Phospholactate
16. gi|15987978 58387 86 Chain A, S402p Mutant Of Rabbit Muscle Pyruvate Kinase
17. gi|15987970 58377 86 Chain A, Recombinant Rabbit Muscle Pyruvate Kinase
18. gi|301598638 58496 85 Chain A, The Structure Of Muscle Pyruvate Kinase In Complex With Proline, Pyruvate, And Mn2+
19. gi|2851533 58524 85 RecName: Full=Pyruvate kinase isozymes M1/M2; AltName: Full=Pyruvate kinase muscle isozyme

20. gi|307548866 58508 85 pyruvate kinase isozymes M1/M2 isoform 1 [Oryctolagus cuniculus]

Results List

1. gi|329664500 Mass: 58482 Score: 151 Expect: 8.4e-010 Matches: 23
pyruvate kinase isozymes M1/M2 [Bos taurus]
Observed Mr(expt) Mr(calc) ppm Start End Miss Peptide
868.4825 867.4752 867.4749 0.41 377 - 383 0 R.MQHLIAR.E
884.4753 883.4680 883.4698 -2.00 377 - 383 0 R.MQHLIAR.E + Oxidation (M)
1019.5126 1018.5053 1018.5083 -2.97 368 - 376 0 K.GDYPLEAVR.M
1197.6399 1196.6326 1196.6401 -6.24 33 - 43 0 R.LDIDSPPITAR.N
1359.6973 1358.6900 1358.6976 -5.60 44 - 56 0 R.NTGIICTIGPASR.A
1394.7686 1393.7613 1393.7677 -4.60 267 - 278 1 K.IISKIENHEGVR.R
1447.8284 1446.8211 1446.8334 -8.47 174 - 186 0 K.IYVDDGLISLLVK.Q
1681.8047 1680.7974 1680.8029 -3.24 280 - 294 0 R.FDEILEASDGIMVAR.G + Oxidation (M)
1703.9412 1702.9339 1702.9869 -31.11 174 - 188 1 K.IYVDDGLISLLVKQK.G
1821.9152 1820.9079 1820.9091 -0.62 279 - 294 1 R.RFDEILEASDGIMVAR.G
1828.0106 1827.0033 1827.0142 -5.94 295 - 311 1 R.GDLGIEIPAEKVFLAQK.M
1837.9033 1836.8960 1836.9040 -4.33 279 - 294 1 R.RFDEILEASDGIMVAR.G + Oxidation (M)
1858.9102 1857.9029 1857.9084 -2.93 231 - 246 0 K.FGVEQNVDMVFASFIR.K
1874.9021 1873.8948 1873.9033 -4.52 231 - 246 0 K.FGVEQNVDMVFASFIR.K + Oxidation (M)
1883.8992 1882.8919 1882.8962 -2.27 74 - 89 0 R.LNFSHGTHEYHAETIK.N
1987.0120 1986.0047 1986.0033 0.70 231 - 247 1 K.FGVEQNVDMVFASFIRK.A
2003.0203 2002.0130 2001.9982 7.38 231 - 247 1 K.FGVEQNVDMVFASFIRK.A + Oxidation (M)
2391.2585 2390.2512 2390.2529 -0.71 505 - 526 1 K.KGDVVIVLTGWRPGSGFTNTMR.V
2407.2490 2406.2417 2406.2478 -2.54 505 - 526 1 K.KGDVVIVLTGWRPGSGFTNTMR.V + Oxidation (M)
2493.2866 2492.2793 2492.2798 -0.21 93 - 115 0 R.EATESFASDPILYRPVAVALDTK.G
2543.2600 2542.2527 2542.2526 0.04 468 - 489 1 R.GIFPVVCKDPVQEAWAEDVDLR.V
2571.2886 2570.2813 2570.2839 -1.01 225 - 246 1 K.DIQDLKFGVEQNVDMVFASFIR.K
3045.6150 3044.6077 3044.5818 8.50 93 - 120 1 R.EATESFASDPILYRPVAVALDTKGPEIR.T
No match to: 877.4737, 1005.5708, 1061.4825, 1077.4830, 1232.5408, 1320.5782, 1456.7817, 1473.8091, 1496.8522, 1564.7028,
1583.8815, 1586.8365, 1618.7668, 1622.6923, 1640.6997, 1643.7584, 1668.7452, 1693.7264, 1708.8972, 1719.9355, 1790.8999,
1803.9180, 2045.0212, 2051.0466, 2059.0266, 2063.0315, 2146.0615, 2250.1206, 2268.1143, 2286.1282, 2314.1538, 2347.1199,
2365.1323, 2373.1650, 2475.2803, 2501.2695, 2529.3208, 2595.1741, 2612.1775, 2623.2139, 3153.4890, 3596.6897

2. gi|73587283 Mass: 62016 Score: 146 Expect: 2.7e-009 Matches: 23
PKM2 protein [Bos taurus]
Observed Mr(expt) Mr(calc) ppm Start End Miss Peptide
868.4825 867.4752 867.4749 0.41 411 - 417 0 R.MQHLIAR.E
884.4753 883.4680 883.4698 -2.00 411 - 417 0 R.MQHLIAR.E + Oxidation (M)
1019.5126 1018.5053 1018.5083 -2.97 402 - 410 0 K.GDYPLEAVR.M
1197.6399 1196.6326 1196.6401 -6.24 67 - 77 0 R.LDIDSPPITAR.N
1359.6973 1358.6900 1358.6976 -5.60 78 - 90 0 R.NTGIICTIGPASR.A
1394.7686 1393.7613 1393.7677 -4.60 301 - 312 1 K.IISKIENHEGVR.R
1447.8284 1446.8211 1446.8334 -8.47 208 - 220 0 K.IYVDDGLISLLVK.Q
1681.8047 1680.7974 1680.8029 -3.24 314 - 328 0 R.FDEILEASDGIMVAR.G + Oxidation (M)
1703.9412 1702.9339 1702.9869 -31.11 208 - 222 1 K.IYVDDGLISLLVKQK.G
1821.9152 1820.9079 1820.9091 -0.62 313 - 328 1 R.RFDEILEASDGIMVAR.G
1828.0106 1827.0033 1827.0142 -5.94 329 - 345 1 R.GDLGIEIPAEKVFLAQK.M
1837.9033 1836.8960 1836.9040 -4.33 313 - 328 1 R.RFDEILEASDGIMVAR.G + Oxidation (M)
1858.9102 1857.9029 1857.9084 -2.93 265 - 280 0 K.FGVEQNVDMVFASFIR.K
1874.9021 1873.8948 1873.9033 -4.52 265 - 280 0 K.FGVEQNVDMVFASFIR.K + Oxidation (M)
1883.8992 1882.8919 1882.8962 -2.27 108 - 123 0 R.LNFSHGTHEYHAETIK.N
1987.0120 1986.0047 1986.0033 0.70 265 - 281 1 K.FGVEQNVDMVFASFIRK.A
2003.0203 2002.0130 2001.9982 7.38 265 - 281 1 K.FGVEQNVDMVFASFIRK.A + Oxidation (M)
2391.2585 2390.2512 2390.2529 -0.71 539 - 560 1 K.KGDVVIVLTGWRPGSGFTNTMR.V
2407.2490 2406.2417 2406.2478 -2.54 539 - 560 1 K.KGDVVIVLTGWRPGSGFTNTMR.V + Oxidation (M)
2493.2866 2492.2793 2492.2798 -0.21 127 - 149 0 R.EATESFASDPILYRPVAVALDTK.G
2543.2600 2542.2527 2542.2526 0.04 502 - 523 1 R.GIFPVVCKDPVQEAWAEDVDLR.V
2571.2886 2570.2813 2570.2839 -1.01 259 - 280 1 K.DIQDLKFGVEQNVDMVFASFIR.K
3045.6150 3044.6077 3044.5818 8.50 127 - 154 1 R.EATESFASDPILYRPVAVALDTKGPEIR.T
No match to: 877.4737, 1005.5708, 1061.4825, 1077.4830, 1232.5408, 1320.5782, 1456.7817, 1473.8091, 1496.8522, 1564.7028,
1583.8815, 1586.8365, 1618.7668, 1622.6923, 1640.6997, 1643.7584, 1668.7452, 1693.7264, 1708.8972, 1719.9355, 1790.8999,
1803.9180, 2045.0212, 2051.0466, 2059.0266, 2063.0315, 2146.0615, 2250.1206, 2268.1143, 2286.1282, 2314.1538, 2347.1199,
2365.1323, 2373.1650, 2475.2803, 2501.2695, 2529.3208, 2595.1741, 2612.1775, 2623.2139, 3153.4890, 3596.6897

3. gi|359807367 Mass: 58461 Score: 141 Expect: 8.4e-009 Matches: 22
pyruvate kinase isozymes M1/M2 isoform 2 [Mus musculus]
Observed Mr(expt) Mr(calc) ppm Start End Miss Peptide

868.4825 867.4752 867.4749 0.41 377 - 383 0 R.MQHLIAR.E
884.4753 883.4680 883.4698 -2.00 377 - 383 0 R.MQHLIAR.E + Oxidation (M)
1019.5126 1018.5053 1018.5083 -2.97 368 - 376 0 K.GDYPLEAVR.M
1061.4825 1060.4752 1060.4760 -0.72 384 - 392 0 R.EAEAAMFHR.L
1077.4830 1076.4757 1076.4709 4.48 384 - 392 0 R.EAEAAMFHR.L + Oxidation (M)
1197.6399 1196.6326 1196.5747 48.4 142 - 151 0 K.ITLDNAYMEK.C
1359.6973 1358.6900 1358.6976 -5.60 44 - 56 0 R.NTGIICTIGPASR.S
1394.7686 1393.7613 1393.7677 -4.60 267 - 278 1 K.IISKIENHEGVR.R
1473.8091 1472.8018 1472.8021 -0.17 423 - 436 0 K.CLAAALIVLTESGR.S
1586.8365 1585.8292 1585.7736 35.1 476 - 489 0 K.DAVLNAWAEDVDLR.V
1681.8047 1680.7974 1680.8029 -3.24 280 - 294 0 R.FDEILEASDGIMVAR.G + Oxidation (M)
1719.9355 1718.9282 1718.9454 -10.01 174 - 188 1 K.IYVDDGLISLQVKEK.G
1821.9152 1820.9079 1820.9091 -0.62 279 - 294 1 R.RFDEILEASDGIMVAR.G
1828.0106 1827.0033 1827.0142 -5.94 295 - 311 1 R.GDLGIEIPAEKVFLAQK.M
1837.9033 1836.8960 1836.9040 -4.33 279 - 294 1 R.RFDEILEASDGIMVAR.G + Oxidation (M)
1883.8992 1882.8919 1882.8962 -2.27 74 - 89 0 R.LNFSHGTHEYHAETIK.N
2063.0315 2062.0242 2062.0551 -14.97 44 - 62 1 R.NTGIICTIGPASRSVEMLK.E + Oxidation (M)
2391.2585 2390.2512 2390.2529 -0.71 505 - 526 1 K.KGDVVIVLTGWRPGSGFTNTMR.V
2407.2490 2406.2417 2406.2478 -2.54 505 - 526 1 K.KGDVVIVLTGWRPGSGFTNTMR.V + Oxidation (M)
2493.2866 2492.2793 2492.2798 -0.21 93 - 115 0 R.EATESFASDPILYRPVAVALDTK.G
2501.2695 2500.2622 2500.2784 -6.48 468 - 489 1 R.GIFPVLCKDAVLNAWAEDVDLR.V
3045.6150 3044.6077 3044.5818 8.50 93 - 120 1 R.EATESFASDPILYRPVAVALDTKGPEIR.T
No match to: 877.4737, 1005.5708, 1232.5408, 1320.5782, 1447.8284, 1456.7817, 1496.8522, 1564.7028,
1583.8815, 1618.7668,
1622.6923, 1640.6997, 1643.7584, 1668.7452, 1693.7264, 1703.9412, 1708.8972, 1790.8999, 1803.9180,
1858.9102, 1874.9021,
1987.0120, 2003.0203, 2045.0212, 2051.0466, 2059.0266, 2146.0615, 2250.1206, 2268.1143, 2286.1282,
2314.1538, 2347.1199,
2365.1323, 2373.1650, 2475.2803, 2529.3208, 2543.2600, 2571.2886, 2595.1741, 2612.1775, 2623.2139,
3153.4890, 3596.6897
4. gi|1405933 Mass: 58448 Score: 135 Expect: 3.4e-008 Matches: 20
M2-type pyruvate kinase [Mus musculus]
Observed Mr(expt) Mr(calc) ppm Start End Miss Peptide
868.4825 867.4752 867.4749 0.41 377 - 383 0 R.MQHLIAR.E
884.4753 883.4680 883.4698 -2.00 377 - 383 0 R.MQHLIAR.E + Oxidation (M)
1019.5126 1018.5053 1018.5083 -2.97 368 - 376 0 K.GDYPLEAVR.M
1197.6399 1196.6326 1196.5747 48.4 142 - 151 0 K.ITLDNAYMEK.C
1359.6973 1358.6900 1358.6976 -5.60 44 - 56 0 R.NTGIICTIGPASR.S
1394.7686 1393.7613 1393.7677 -4.60 267 - 278 1 K.IISKIENHEGVR.R
1586.8365 1585.8292 1585.7736 35.1 476 - 489 0 K.DAVLNAWAEDVDLR.V
1681.8047 1680.7974 1680.8029 -3.24 280 - 294 0 R.FDEILEASDGIMVAR.G + Oxidation (M)
1719.9355 1718.9282 1718.9454 -10.01 174 - 188 1 K.IYVDDGLISLQVKEK.G
1821.9152 1820.9079 1820.9091 -0.62 279 - 294 1 R.RFDEILEASDGIMVAR.G
1828.0106 1827.0033 1827.0142 -5.94 295 - 311 1 R.GDLGIEIPAEKVFLAQK.M
1837.9033 1836.8960 1836.9040 -4.33 279 - 294 1 R.RFDEILEASDGIMVAR.G + Oxidation (M)
1883.8992 1882.8919 1882.8962 -2.27 74 - 89 0 R.LNFSHGTHEYHAETIK.N
2063.0315 2062.0242 2062.0551 -14.97 44 - 62 1 R.NTGIICTIGPASRSVEMLK.E + Oxidation (M)
2365.1323 2364.1250 2364.1786 -22.65 317 - 336 1 R.CNRAGKPVICSTQMLEIMIK.K + Oxidation (M)
2391.2585 2390.2512 2390.2529 -0.71 505 - 526 1 K.KGDVVIVLTGWRPGSGFTNTMR.V
2407.2490 2406.2417 2406.2478 -2.54 505 - 526 1 K.KGDVVIVLTGWRPGSGFTNTMR.V + Oxidation (M)
2493.2866 2492.2793 2492.2798 -0.21 93 - 115 0 R.EATESFASDPILYRPVAVALDTK.G
2501.2695 2500.2622 2500.2784 -6.48 468 - 489 1 R.GIFPVLCKDAVLNAWAEDVDLR.V
3045.6150 3044.6077 3044.5818 8.50 93 - 120 1 R.EATESFASDPILYRPVAVALDTKGPEIR.T
No match to: 877.4737, 1005.5708, 1061.4825, 1077.4830, 1232.5408, 1320.5782, 1447.8284, 1456.7817,
1473.8091, 1496.8522,
1564.7028, 1583.8815, 1618.7668, 1622.6923, 1640.6997, 1643.7584, 1668.7452, 1693.7264, 1703.9412,
1708.8972, 1790.8999,
1803.9180, 1858.9102, 1874.9021, 1987.0120, 2003.0203, 2045.0212, 2051.0466, 2059.0266, 2146.0615,
2250.1206, 2268.1143,
2286.1282, 2314.1538, 2347.1199, 2373.1650, 2475.2803, 2529.3208, 2543.2600, 2571.2886, 2595.1741,
2612.1775, 2623.2139,
3153.4890, 3596.6897
5. gi|74221210 Mass: 58379 Score: 123 Expect: 5.3e-007 Matches: 19
unnamed protein product [Mus musculus]
Observed Mr(expt) Mr(calc) ppm Start End Miss Peptide
868.4825 867.4752 867.4749 0.41 377 - 383 0 R.MQHLIAR.E
884.4753 883.4680 883.4698 -2.00 377 - 383 0 R.MQHLIAR.E + Oxidation (M)
1019.5126 1018.5053 1018.5083 -2.97 368 - 376 0 K.GDYPLEAVR.M
1197.6399 1196.6326 1196.5747 48.4 142 - 151 0 K.ITLDNAYMEK.C
1359.6973 1358.6900 1358.6976 -5.60 44 - 56 0 R.NTGIICTIGPASR.S
1394.7686 1393.7613 1393.7677 -4.60 267 - 278 1 K.IISKIENHEGVR.R
1586.8365 1585.8292 1585.7736 35.1 476 - 489 0 K.DAVLNAWAEDVDLR.V
1681.8047 1680.7974 1680.8029 -3.24 280 - 294 0 R.FDEILEASDGIMVAR.G + Oxidation (M)
1719.9355 1718.9282 1718.9454 -10.01 174 - 188 1 K.IYVDDGLISLQVKEK.G

1821.9152 1820.9079 1820.9091 -0.62 279 - 294 1 R.RFDEILEASDGIMVAR.G
1828.0106 1827.0033 1827.0142 -5.94 295 - 311 1 R.GDLGIEIPAEKVFLAQK.M
1837.9033 1836.8960 1836.9040 -4.33 279 - 294 1 R.RFDEILEASDGIMVAR.G + Oxidation (M)
1883.8992 1882.8919 1882.8962 -2.27 74 - 89 0 R.LNFSHGTHEYHAETIK.N
2063.0315 2062.0242 2062.0551 -14.97 44 - 62 1 R.NTGIICTIGPASRSVEMLK.E + Oxidation (M)
2391.2585 2390.2512 2390.2529 -0.71 505 - 526 1 K.KGDVVIVLTGWRPGSGFTNTMR.V
2407.2490 2406.2417 2406.2478 -2.54 505 - 526 1 K.KGDVVIVLTGWRPGSGFTNTMR.V + Oxidation (M)
2493.2866 2492.2793 2492.2798 -0.21 93 - 115 0 R.EATESFASDPILYRPVAVALDTK.G
2501.2695 2500.2622 2500.2784 -6.48 468 - 489 1 R.GIFPVLCKDAVLNAWAEDVDLR.V
3045.6150 3044.6077 3044.5818 8.50 93 - 120 1 R.EATESFASDPILYRPVAVALDTKGPEIR.T
No match to: 877.4737, 1005.5708, 1061.4825, 1077.4830, 1232.5408, 1320.5782, 1447.8284, 1456.7817,
1473.8091, 1496.8522,
1564.7028, 1583.8815, 1618.7668, 1622.6923, 1640.6997, 1643.7584, 1668.7452, 1693.7264, 1703.9412,
1708.8972, 1790.8999,
1803.9180, 1858.9102, 1874.9021, 1987.0120, 2003.0203, 2045.0212, 2051.0466, 2059.0266, 2146.0615,
2250.1206, 2268.1143,
2286.1282, 2314.1538, 2347.1199, 2365.1323, 2373.1650, 2475.2803, 2529.3208, 2543.2600, 2571.2886,
2595.1741, 2612.1775,
2623.2139, 3153.4890, 3596.6897
6. gi|551295 Mass: 58394 Score: 123 Expect: 5.3e-007 Matches: 19
pyruvate kinase M [Mus musculus]
Observed Mr(expt) Mr(calc) ppm Start End Miss Peptide
868.4825 867.4752 867.4749 0.41 377 - 383 0 R.MQHLIAR.E
884.4753 883.4680 883.4698 -2.00 377 - 383 0 R.MQHLIAR.E + Oxidation (M)
1019.5126 1018.5053 1018.5083 -2.97 368 - 376 0 K.GDYPLEAVR.M
1197.6399 1196.6326 1196.5747 48.4 142 - 151 0 K.ITLDNAYMEK.C
1359.6973 1358.6900 1358.6976 -5.60 44 - 56 0 R.NTGIICTIGPASR.S
1394.7686 1393.7613 1393.7677 -4.60 267 - 278 1 K.IISKIENHEGVR.R
1586.8365 1585.8292 1585.7736 35.1 476 - 489 0 K.DAVLNAWAEDVDLR.V
1681.8047 1680.7974 1680.8029 -3.24 280 - 294 0 R.FDEILEASDGIMVAR.G + Oxidation (M)
1719.9355 1718.9282 1718.9454 -10.01 174 - 188 1 K.IYVDDGLISLQVKEK.G
1821.9152 1820.9079 1820.9091 -0.62 279 - 294 1 R.RFDEILEASDGIMVAR.G
1837.9033 1836.8960 1836.9040 -4.33 279 - 294 1 R.RFDEILEASDGIMVAR.G + Oxidation (M)
1883.8992 1882.8919 1882.8962 -2.27 74 - 89 0 R.LNFSHGTHEYHAETIK.N
2063.0315 2062.0242 2062.0551 -14.97 44 - 62 1 R.NTGIICTIGPASRSVEMLK.E + Oxidation (M)
2365.1323 2364.1250 2364.1786 -22.65 317 - 336 1 R.CNRAGKPVICSTQMLEIMIK.K + Oxidation (M)
2391.2585 2390.2512 2390.2529 -0.71 505 - 526 1 K.KGDVVIVLTGWRPGSGFTNTMR.V
2407.2490 2406.2417 2406.2478 -2.54 505 - 526 1 K.KGDVVIVLTGWRPGSGFTNTMR.V + Oxidation (M)
2493.2866 2492.2793 2492.2798 -0.21 93 - 115 0 R.EATESFASDPILYRPVAVALDTK.G
2501.2695 2500.2622 2500.2784 -6.48 468 - 489 1 R.GIFPVLCKDAVLNAWAEDVDLR.V
3045.6150 3044.6077 3044.5818 8.50 93 - 120 1 R.EATESFASDPILYRPVAVALDTKGPEIR.T
No match to: 877.4737, 1005.5708, 1061.4825, 1077.4830, 1232.5408, 1320.5782, 1447.8284, 1456.7817,
1473.8091, 1496.8522,
1564.7028, 1583.8815, 1618.7668, 1622.6923, 1640.6997, 1643.7584, 1668.7452, 1693.7264, 1703.9412,
1708.8972, 1790.8999,
1803.9180, 1828.0106, 1858.9102, 1874.9021, 1987.0120, 2003.0203, 2045.0212, 2051.0466, 2059.0266,
2146.0615, 2250.1206,
2268.1143, 2286.1282, 2314.1538, 2347.1199, 2373.1650, 2475.2803, 2529.3208, 2543.2600, 2571.2886,
2595.1741, 2612.1775,
2623.2139, 3153.4890, 3596.6897
7. gi|31981562 Mass: 58378 Score: 122 Expect: 6.7e-007 Matches: 19
pyruvate kinase isozymes M1/M2 isoform 1 [Mus musculus]
Observed Mr(expt) Mr(calc) ppm Start End Miss Peptide
868.4825 867.4752 867.4749 0.41 377 - 383 0 R.MQHLIAR.E
884.4753 883.4680 883.4698 -2.00 377 - 383 0 R.MQHLIAR.E + Oxidation (M)
1019.5126 1018.5053 1018.5083 -2.97 368 - 376 0 K.GDYPLEAVR.M
1197.6399 1196.6326 1196.5747 48.4 142 - 151 0 K.ITLDNAYMEK.C
1359.6973 1358.6900 1358.6976 -5.60 44 - 56 0 R.NTGIICTIGPASR.S
1394.7686 1393.7613 1393.7677 -4.60 267 - 278 1 K.IISKIENHEGVR.R
1586.8365 1585.8292 1585.7736 35.1 476 - 489 0 K.DAVLNAWAEDVDLR.V
1681.8047 1680.7974 1680.8029 -3.24 280 - 294 0 R.FDEILEASDGIMVAR.G + Oxidation (M)
1719.9355 1718.9282 1718.9454 -10.01 174 - 188 1 K.IYVDDGLISLQVKEK.G
1821.9152 1820.9079 1820.9091 -0.62 279 - 294 1 R.RFDEILEASDGIMVAR.G
1828.0106 1827.0033 1827.0142 -5.94 295 - 311 1 R.GDLGIEIPAEKVFLAQK.M
1837.9033 1836.8960 1836.9040 -4.33 279 - 294 1 R.RFDEILEASDGIMVAR.G + Oxidation (M)
1883.8992 1882.8919 1882.8962 -2.27 74 - 89 0 R.LNFSHGTHEYHAETIK.N
2063.0315 2062.0242 2062.0551 -14.97 44 - 62 1 R.NTGIICTIGPASRSVEMLK.E + Oxidation (M)
2391.2585 2390.2512 2390.2529 -0.71 505 - 526 1 K.KGDVVIVLTGWRPGSGFTNTMR.V
2407.2490 2406.2417 2406.2478 -2.54 505 - 526 1 K.KGDVVIVLTGWRPGSGFTNTMR.V + Oxidation (M)
2493.2866 2492.2793 2492.2798 -0.21 93 - 115 0 R.EATESFASDPILYRPVAVALDTK.G
2501.2695 2500.2622 2500.2784 -6.48 468 - 489 1 R.GIFPVLCKDAVLNAWAEDVDLR.V
3045.6150 3044.6077 3044.5818 8.50 93 - 120 1 R.EATESFASDPILYRPVAVALDTKGPEIR.T
No match to: 877.4737, 1005.5708, 1061.4825, 1077.4830, 1232.5408, 1320.5782, 1447.8284, 1456.7817,
1473.8091, 1496.8522,

1564.7028, 1583.8815, 1618.7668, 1622.6923, 1640.6997, 1643.7584, 1668.7452, 1693.7264, 1703.9412, 1708.8972, 1790.8999,
1803.9180, 1858.9102, 1874.9021, 1987.0120, 2003.0203, 2045.0212, 2051.0466, 2059.0266, 2146.0615, 2250.1206, 2268.1143,
2286.1282, 2314.1538, 2347.1199, 2365.1323, 2373.1650, 2475.2803, 2529.3208, 2543.2600, 2571.2886, 2595.1741, 2612.1775,
2623.2139, 3153.4890, 3596.6897
8. gi|74151988 Mass: 58390 Score: 122 Expect: 6.7e-007 Matches: 19
unnamed protein product [Mus musculus]
Observed Mr(expt) Mr(calc) ppm Start End Miss Peptide
868.4825 867.4752 867.4749 0.41 377 - 383 0 R.MQHLIAR.E
884.4753 883.4680 883.4698 -2.00 377 - 383 0 R.MQHLIAR.E + Oxidation (M)
1019.5126 1018.5053 1018.5083 -2.97 368 - 376 0 K.GDYPLEAVR.M
1197.6399 1196.6326 1196.5747 48.4 142 - 151 0 K.ITLDNAYMEK.C
1359.6973 1358.6900 1358.6976 -5.60 44 - 56 0 R.NTGIICTIGPASR.S
1394.7686 1393.7613 1393.7677 -4.60 267 - 278 1 K.IISKIENHEGVR.R
1586.8365 1585.8292 1585.7736 35.1 476 - 489 0 K.DAVLNAWAEDVDLR.V
1681.8047 1680.7974 1680.8029 -3.24 280 - 294 0 R.FDEILEASDGIMVAR.G + Oxidation (M)
1719.9355 1718.9282 1718.9454 -10.01 174 - 188 1 K.IYVDDGLISLQVKEK.G
1821.9152 1820.9079 1820.9091 -0.62 279 - 294 1 R.RFDEILEASDGIMVAR.G
1828.0106 1827.0033 1827.0142 -5.94 295 - 311 1 R.GDLGIEIPAEKVFLAQK.M
1837.9033 1836.8960 1836.9040 -4.33 279 - 294 1 R.RFDEILEASDGIMVAR.G + Oxidation (M)
1883.8992 1882.8919 1882.8962 -2.27 74 - 89 0 R.LNFSHGTHEYHAETIK.N
2063.0315 2062.0242 2062.0551 -14.97 44 - 62 1 R.NTGIICTIGPASRSVEMLK.E + Oxidation (M)
2391.2585 2390.2512 2390.2529 -0.71 505 - 526 1 K.KGDVVIVLTGWRPGSGFTNTMR.V
2407.2490 2406.2417 2406.2478 -2.54 505 - 526 1 K.KGDVVIVLTGWRPGSGFTNTMR.V + Oxidation (M)
2493.2866 2492.2793 2492.2798 -0.21 93 - 115 0 R.EATESFASDPILYRPVAVALDTK.G
2501.2695 2500.2622 2500.2784 -6.48 468 - 489 1 R.GIFPVLCKDAVLNAWAEDVDLR.V
3045.6150 3044.6077 3044.5818 8.50 93 - 120 1 R.EATESFASDPILYRPVAVALDTKGPEIR.A
No match to: 877.4737, 1005.5708, 1061.4825, 1077.4830, 1232.5408, 1320.5782, 1447.8284, 1456.7817, 1473.8091, 1496.8522,
1564.7028, 1583.8815, 1618.7668, 1622.6923, 1640.6997, 1643.7584, 1668.7452, 1693.7264, 1703.9412, 1708.8972, 1790.8999,
1803.9180, 1858.9102, 1874.9021, 1987.0120, 2003.0203, 2045.0212, 2051.0466, 2059.0266, 2146.0615, 2250.1206, 2268.1143,
2286.1282, 2314.1538, 2347.1199, 2365.1323, 2373.1650, 2475.2803, 2529.3208, 2543.2600, 2571.2886, 2595.1741, 2612.1775,
2623.2139, 3153.4890, 3596.6897
9. gi|74196318 Mass: 58377 Score: 112 Expect: 6.7e-006 Matches: 18
unnamed protein product [Mus musculus]
Observed Mr(expt) Mr(calc) ppm Start End Miss Peptide
868.4825 867.4752 867.4749 0.41 377 - 383 0 R.MQHLIAR.E
884.4753 883.4680 883.4698 -2.00 377 - 383 0 R.MQHLIAR.E + Oxidation (M)
1019.5126 1018.5053 1018.5083 -2.97 368 - 376 0 K.GDYPLEAVR.M
1197.6399 1196.6326 1196.5747 48.4 142 - 151 0 K.ITLDNAYMEK.C
1359.6973 1358.6900 1358.6976 -5.60 44 - 56 0 R.NTGIICTIGPASR.S
1394.7686 1393.7613 1393.7677 -4.60 267 - 278 1 K.IISKIENHEGVR.R
1681.8047 1680.7974 1680.8029 -3.24 280 - 294 0 R.FDEILEASDGIMVAR.G + Oxidation (M)
1719.9355 1718.9282 1718.9454 -10.01 174 - 188 1 K.IYVDDGLISLQVKEK.G
1821.9152 1820.9079 1820.9091 -0.62 279 - 294 1 R.RFDEILEASDGIMVAR.G
1828.0106 1827.0033 1827.0142 -5.94 295 - 311 1 R.GDLGIEIPAEKVFLAQK.M
1837.9033 1836.8960 1836.9040 -4.33 279 - 294 1 R.RFDEILEASDGIMVAR.G + Oxidation (M)
1883.8992 1882.8919 1882.8962 -2.27 74 - 89 0 R.LNFSHGTHEYHAETIK.N
2063.0315 2062.0242 2062.0551 -14.97 44 - 62 1 R.NTGIICTIGPASRSVEMLK.E + Oxidation (M)
2391.2585 2390.2512 2390.2529 -0.71 505 - 526 1 K.KGDVVIVLTGWRPGSGFTNTMR.V
2407.2490 2406.2417 2406.2478 -2.54 505 - 526 1 K.KGDVVIVLTGWRPGSGFTNTMR.V + Oxidation (M)
2493.2866 2492.2793 2492.2798 -0.21 93 - 115 0 R.EATESFASDPILYRPVAVALDTK.G
2529.3208 2528.3135 2528.2693 17.5 476 - 498 1 K.DAVLNAWAENVDLRVNLAMDVGK.A + Oxidation (M)
3045.6150 3044.6077 3044.5818 8.50 93 - 120 1 R.EATESFASDPILYRPVAVALDTKGPEIR.T
No match to: 877.4737, 1005.5708, 1061.4825, 1077.4830, 1232.5408, 1320.5782, 1447.8284, 1456.7817, 1473.8091, 1496.8522,
1564.7028, 1583.8815, 1586.8365, 1618.7668, 1622.6923, 1640.6997, 1643.7584, 1668.7452, 1693.7264, 1703.9412, 1708.8972,
1790.8999, 1803.9180, 1858.9102, 1874.9021, 1987.0120, 2003.0203, 2045.0212, 2051.0466, 2059.0266, 2146.0615, 2250.1206,
2268.1143, 2286.1282, 2314.1538, 2347.1199, 2365.1323, 2373.1650, 2475.2803, 2501.2695, 2543.2600, 2571.2886, 2595.1741,
2612.1775, 2623.2139, 3153.4890, 3596.6897
10. gi|74222653 Mass: 58320 Score: 112 Expect: 6.7e-006 Matches: 18
unnamed protein product [Mus musculus]
Observed Mr(expt) Mr(calc) ppm Start End Miss Peptide
868.4825 867.4752 867.4749 0.41 377 - 383 0 R.MQHLIAR.E
884.4753 883.4680 883.4698 -2.00 377 - 383 0 R.MQHLIAR.E + Oxidation (M)
1019.5126 1018.5053 1018.5083 -2.97 368 - 376 0 K.GDYPLEAVR.M

1197.6399 1196.6326 1196.5747 48.4 142 - 151 0 K.ITLDNAYMEK.C
1359.6973 1358.6900 1358.6976 -5.60 44 - 56 0 R.NTGIICTIGPASR.S
1394.7686 1393.7613 1393.7677 -4.60 267 - 278 1 K.IISKIENHEGVR.R
1586.8365 1585.8292 1585.7736 35.1 476 - 489 0 K.DAVLNAWAEDVDLR.V
1681.8047 1680.7974 1680.8029 -3.24 280 - 294 0 R.FDEILEASDGIMVAR.G + Oxidation (M)
1821.9152 1820.9079 1820.9091 -0.62 279 - 294 1 R.RFDEILEASDGIMVAR.G
1828.0106 1827.0033 1827.0142 -5.94 295 - 311 1 R.GDLGIEIPAEKVFLAQK.M
1837.9033 1836.8960 1836.9040 -4.33 279 - 294 1 R.RFDEILEASDGIMVAR.G + Oxidation (M)
1883.8992 1882.8919 1882.8962 -2.27 74 - 89 0 R.LNFSHGTHEYHAETIK.N
2063.0315 2062.0242 2062.0551 -14.97 44 - 62 1 R.NTGIICTIGPASRSVEMLK.E + Oxidation (M)
2391.2585 2390.2512 2390.2529 -0.71 505 - 526 1 K.KGDVVIVLTGWRPGSGFTNTMR.V
2407.2490 2406.2417 2406.2478 -2.54 505 - 526 1 K.KGDVVIVLTGWRPGSGFTNTMR.V + Oxidation (M)
2493.2866 2492.2793 2492.2798 -0.21 93 - 115 0 R.EATESFASDPILYRPVAVALDTK.G
2501.2695 2500.2622 2500.2784 -6.48 468 - 489 1 R.GIFPVLCKDAVLNAWAEDVDLR.V
3045.6150 3044.6077 3044.5818 8.50 93 - 120 1 R.EATESFASDPILYRPVAVALDTKGPEIR.T
No match to: 877.4737, 1005.5708, 1061.4825, 1077.4830, 1232.5408, 1320.5782, 1447.8284, 1456.7817,
1473.8091, 1496.8522,
1564.7028, 1583.8815, 1618.7668, 1622.6923, 1640.6997, 1643.7584, 1668.7452, 1693.7264, 1703.9412,
1708.8972, 1719.9355,
1790.8999, 1803.9180, 1858.9102, 1874.9021, 1987.0120, 2003.0203, 2045.0212, 2051.0466, 2059.0266,
2146.0615, 2250.1206,
2268.1143, 2286.1282, 2314.1538, 2347.1199, 2365.1323, 2373.1650, 2475.2803, 2529.3208, 2543.2600,
2571.2886, 2595.1741,
2612.1775, 2623.2139, 3153.4890, 3596.6897
11. gi|74212815 Mass: 43537 Score: 108 Expect: 1.7e-005 Matches: 17
unnamed protein product [Mus musculus]
Observed Mr(expt) Mr(calc) ppm Start End Miss Peptide
868.4825 867.4752 867.4749 0.41 377 - 383 0 R.MQHLIAR.E
884.4753 883.4680 883.4698 -2.00 377 - 383 0 R.MQHLIAR.E + Oxidation (M)
1019.5126 1018.5053 1018.5083 -2.97 368 - 376 0 K.GDYPLEAVR.M
1061.4825 1060.4752 1060.4760 -0.72 384 - 392 0 R.EAEAAMFHR.L
1077.4830 1076.4757 1076.4709 4.48 384 - 392 0 R.EAEAAMFHR.L + Oxidation (M)
1197.6399 1196.6326 1196.5747 48.4 142 - 151 0 K.ITLDNAYMEK.C
1359.6973 1358.6900 1358.6976 -5.60 44 - 56 0 R.NTGIICTIGPASR.S
1394.7686 1393.7613 1393.7677 -4.60 267 - 278 1 K.IISKIENHEGVR.R
1681.8047 1680.7974 1680.8029 -3.24 280 - 294 0 R.FDEILEASDGIMVAR.G + Oxidation (M)
1719.9355 1718.9282 1718.9454 -10.01 174 - 188 1 K.IYVDDGLISLQVKEK.G
1821.9152 1820.9079 1820.9091 -0.62 279 - 294 1 R.RFDEILEASDGIMVAR.G
1828.0106 1827.0033 1827.0142 -5.94 295 - 311 1 R.GDLGIEIPAEKVFLAQK.M
1837.9033 1836.8960 1836.9040 -4.33 279 - 294 1 R.RFDEILEASDGIMVAR.G + Oxidation (M)
1883.8992 1882.8919 1882.8962 -2.27 74 - 89 0 R.LNFSHGTHEYHAETIK.N
2063.0315 2062.0242 2062.0551 -14.97 44 - 62 1 R.NTGIICTIGPASRSVEMLK.E + Oxidation (M)
2493.2866 2492.2793 2492.2798 -0.21 93 - 115 0 R.EATESFASDPILYRPVAVALDTK.G
3045.6150 3044.6077 3044.5818 8.50 93 - 120 1 R.EATESFASDPILYRPVAVALDTKGPEIR.T
No match to: 877.4737, 1005.5708, 1232.5408, 1320.5782, 1447.8284, 1456.7817, 1473.8091, 1496.8522,
1564.7028, 1583.8815,
1586.8365, 1618.7668, 1622.6923, 1640.6997, 1643.7584, 1668.7452, 1693.7264, 1703.9412, 1708.8972,
1790.8999, 1803.9180,
1858.9102, 1874.9021, 1987.0120, 2003.0203, 2045.0212, 2051.0466, 2059.0266, 2146.0615, 2250.1206,
2268.1143, 2286.1282,
2314.1538, 2347.1199, 2365.1323, 2373.1650, 2391.2585, 2407.2490, 2475.2803, 2501.2695, 2529.3208,
2543.2600, 2571.2886,
2595.1741, 2612.1775, 2623.2139, 3153.4890, 3596.6897
12. gi|227908865 Mass: 58465 Score: 94 Expect: 0.00042 Matches: 18
pyruvate kinase isozymes M1/M2 isoform M1 [Equus caballus]
Observed Mr(expt) Mr(calc) ppm Start End Miss Peptide
868.4825 867.4752 867.4749 0.41 377 - 383 0 R.MQHLIAR.E
884.4753 883.4680 883.4698 -2.00 377 - 383 0 R.MQHLIAR.E + Oxidation (M)
1019.5126 1018.5053 1018.5083 -2.97 368 - 376 0 K.GDYPLEAVR.M
1061.4825 1060.4752 1060.4760 -0.72 384 - 392 0 R.EAEAAMFHR.K
1077.4830 1076.4757 1076.4709 4.48 384 - 392 0 R.EAEAAMFHR.K + Oxidation (M)
1197.6399 1196.6326 1196.6401 -6.24 33 - 43 0 R.LDIDSPPITAR.N
1359.6973 1358.6900 1358.6976 -5.60 44 - 56 0 R.NTGIICTIGPASR.S
1394.7686 1393.7613 1393.7677 -4.60 267 - 278 1 K.IISKIENHEGVR.R
1681.8047 1680.7974 1680.8029 -3.24 280 - 294 0 R.FDEILEASDGIMVAR.G + Oxidation (M)
1719.9355 1718.9282 1718.9454 -10.01 174 - 188 1 K.IYVDDGLISLQVKEK.G
1821.9152 1820.9079 1820.9091 -0.62 279 - 294 1 R.RFDEILEASDGIMVAR.G
1828.0106 1827.0033 1827.0142 -5.94 295 - 311 1 R.GDLGIEIPAEKVFLAQK.M
1837.9033 1836.8960 1836.9040 -4.33 279 - 294 1 R.RFDEILEASDGIMVAR.G + Oxidation (M)
1883.8992 1882.8919 1882.8962 -2.27 74 - 89 0 R.LNFSHGTHEYHAETIK.N
2063.0315 2062.0242 2062.0219 1.14 187 - 206 1 K.EKGPDFLVTEVENGGSLGSK.K
2391.2585 2390.2512 2390.2529 -0.71 505 - 526 1 K.KGDVVIVLTGWRPGSGFTNTMR.V
2407.2490 2406.2417 2406.2478 -2.54 505 - 526 1 K.KGDVVIVLTGWRPGSGFTNTMR.V + Oxidation (M)
2543.2600 2542.2527 2542.2526 0.04 468 - 489 1 R.GIFPVVCKDPVQEAWAEDVDLR.V

No match to: 877.4737, 1005.5708, 1232.5408, 1320.5782, 1447.8284, 1456.7817, 1473.8091, 1496.8522,
1564.7028, 1583.8815,
1586.8365, 1618.7668, 1622.6923, 1640.6997, 1643.7584, 1668.7452, 1693.7264, 1703.9412, 1708.8972,
1790.8999, 1803.9180,
1858.9102, 1874.9021, 1987.0120, 2003.0203, 2045.0212, 2051.0466, 2059.0266, 2146.0615, 2250.1206,
2268.1143, 2286.1282,
2314.1538, 2347.1199, 2365.1323, 2373.1650, 2475.2803, 2493.2866, 2501.2695, 2529.3208, 2571.2886,
2595.1741, 2612.1775,
2623.2139, 3045.6150, 3153.4890, 3596.6897
13. gi|219689076 Mass: 58359 Score: 88 Expect: 0.0018 Matches: 16
pyruvate kinase isozymes M1/M2 isoform M2 [Equus caballus]
Observed Mr(expt) Mr(calc) ppm Start End Miss Peptide
868.4825 867.4752 867.4749 0.41 377 - 383 0 R.MQHLIAR.E
884.4753 883.4680 883.4698 -2.00 377 - 383 0 R.MQHLIAR.E + Oxidation (M)
1019.5126 1018.5053 1018.5083 -2.97 368 - 376 0 K.GDYPLEAVR.M
1197.6399 1196.6326 1196.6401 -6.24 33 - 43 0 R.LDIDSPPITAR.N
1359.6973 1358.6900 1358.6976 -5.60 44 - 56 0 R.NTGIICTIGPASR.S
1394.7686 1393.7613 1393.7677 -4.60 267 - 278 1 K.IISKIENHEGVR.R
1681.8047 1680.7974 1680.8029 -3.24 280 - 294 0 R.FDEILEASDGIMVAR.G + Oxidation (M)
1719.9355 1718.9282 1718.9454 -10.01 174 - 188 1 K.IYVDDGLISLQVKEK.G
1821.9152 1820.9079 1820.9091 -0.62 279 - 294 1 R.RFDEILEASDGIMVAR.G
1828.0106 1827.0033 1827.0142 -5.94 295 - 311 1 R.GDLGIEIPAEKVFLAQK.M
1837.9033 1836.8960 1836.9040 -4.33 279 - 294 1 R.RFDEILEASDGIMVAR.G + Oxidation (M)
1883.8992 1882.8919 1882.8962 -2.27 74 - 89 0 R.LNFSHGTHEYHAETIK.N
2063.0315 2062.0242 2062.0219 1.14 187 - 206 1 K.EKGPDFLVTEVENGGSLGSK.K
2391.2585 2390.2512 2390.2529 -0.71 505 - 526 1 K.KGDVVIVLTGWRPGSGFTNTMR.V
2407.2490 2406.2417 2406.2478 -2.54 505 - 526 1 K.KGDVVIVLTGWRPGSGFTNTMR.V + Oxidation (M)
2543.2600 2542.2527 2542.2526 0.04 468 - 489 1 R.GIFPVVCKDPVQEAWAEDVDLR.V
No match to: 877.4737, 1005.5708, 1061.4825, 1077.4830, 1232.5408, 1320.5782, 1447.8284, 1456.7817,
1473.8091, 1496.8522,
1564.7028, 1583.8815, 1586.8365, 1618.7668, 1622.6923, 1640.6997, 1643.7584, 1668.7452, 1693.7264,
1703.9412, 1708.8972,
1790.8999, 1803.9180, 1858.9102, 1874.9021, 1987.0120, 2003.0203, 2045.0212, 2051.0466, 2059.0266,
2146.0615, 2250.1206,
2268.1143, 2286.1282, 2314.1538, 2347.1199, 2365.1323, 2373.1650, 2475.2803, 2493.2866, 2501.2695,
2529.3208, 2571.2886,
2595.1741, 2612.1775, 2623.2139, 3045.6150, 3153.4890, 3596.6897
14. gi|109157779 Mass: 58365 Score: 86 Expect: 0.0027 Matches: 18
Chain A, The Location Of The Allosteric Amino Acid Binding Site Of Muscle Pyruvate Kinase.
Observed Mr(expt) Mr(calc) ppm Start End Miss Peptide
868.4825 867.4752 867.4749 0.41 376 - 382 0 R.MQHLIAR.E
877.4737 876.4664 876.4705 -4.64 393 - 399 0 K.LFEELAR.A
884.4753 883.4680 883.4698 -2.00 376 - 382 0 R.MQHLIAR.E + Oxidation (M)
1005.5708 1004.5635 1004.5654 -1.91 392 - 399 1 R.KLFEELAR.A
1019.5126 1018.5053 1018.5083 -2.97 367 - 375 0 K.GDYPLEAVR.M
1061.4825 1060.4752 1060.4760 -0.72 383 - 391 0 R.EAEAAMFHR.K
1077.4830 1076.4757 1076.4709 4.48 383 - 391 0 R.EAEAAMFHR.K + Oxidation (M)
1197.6399 1196.6326 1196.5747 48.4 141 - 150 0 K.ITLDNAYMEK.C
1359.6973 1358.6900 1358.6976 -5.60 43 - 55 0 R.NTGIICTIGPASR.S
1394.7686 1393.7613 1393.7677 -4.60 266 - 277 1 K.IISKIENHEGVR.R
1473.8091 1472.8018 1472.8021 -0.17 422 - 435 0 K.CLAAALIVLTESGR.S
1681.8047 1680.7974 1680.8029 -3.24 279 - 293 0 R.FDEILEASDGIMVAR.G + Oxidation (M)
1821.9152 1820.9079 1820.9091 -0.62 278 - 293 1 R.RFDEILEASDGIMVAR.G
1828.0106 1827.0033 1827.0142 -5.94 294 - 310 1 R.GDLGIEIPAEKVFLAQK.M
1837.9033 1836.8960 1836.9040 -4.33 278 - 293 1 R.RFDEILEASDGIMVAR.G + Oxidation (M)
2391.2585 2390.2512 2390.2529 -0.71 504 - 525 1 K.KGDVVIVLTGWRPGSGFTNTMR.V
2407.2490 2406.2417 2406.2478 -2.54 504 - 525 1 K.KGDVVIVLTGWRPGSGFTNTMR.V + Oxidation (M)
2543.2600 2542.2527 2542.2526 0.04 467 - 488 1 R.GIFPVVCKDPVQEAWAEDVDLR.V
No match to: 1232.5408, 1320.5782, 1447.8284, 1456.7817, 1496.8522, 1564.7028, 1583.8815, 1586.8365,
1618.7668, 1622.6923,
1640.6997, 1643.7584, 1668.7452, 1693.7264, 1703.9412, 1708.8972, 1719.9355, 1790.8999, 1803.9180,
1858.9102, 1874.9021,
1883.8992, 1987.0120, 2003.0203, 2045.0212, 2051.0466, 2059.0266, 2063.0315, 2146.0615, 2250.1206,
2268.1143, 2286.1282,
2314.1538, 2347.1199, 2365.1323, 2373.1650, 2475.2803, 2493.2866, 2501.2695, 2529.3208, 2571.2886,
2595.1741, 2612.1775,
2623.2139, 3045.6150, 3153.4890, 3596.6897
15. gi|3659945 Mass: 58393 Score: 86 Expect: 0.003 Matches: 18
Chain A, Pyruvate Kinase From Rabbit Muscle With Mg, K, And L- Phospholactate
Observed Mr(expt) Mr(calc) ppm Start End Miss Peptide
868.4825 867.4752 867.4749 0.41 376 - 382 0 R.MQHLIAR.E
877.4737 876.4664 876.4705 -4.64 393 - 399 0 K.LFEELAR.S
884.4753 883.4680 883.4698 -2.00 376 - 382 0 R.MQHLIAR.E + Oxidation (M)
1005.5708 1004.5635 1004.5654 -1.91 392 - 399 1 R.KLFEELAR.S

1019.5126 1018.5053 1018.5083 -2.97 367 - 375 0 K.GDYPLEAVR.M
1061.4825 1060.4752 1060.4760 -0.72 383 - 391 0 R.EAEAAMFHR.K
1077.4830 1076.4757 1076.4709 4.48 383 - 391 0 R.EAEAAMFHR.K + Oxidation (M)
1197.6399 1196.6326 1196.5747 48.4 141 - 150 0 K.ITLDNAYMEK.C
1359.6973 1358.6900 1358.6976 -5.60 43 - 55 0 R.NTGIICTIGPASR.S
1394.7686 1393.7613 1393.7677 -4.60 266 - 277 1 K.IISKIENHEGVR.R
1473.8091 1472.8018 1472.8021 -0.17 422 - 435 0 K.CLAAALIVLTESGR.S
1681.8047 1680.7974 1680.8029 -3.24 279 - 293 0 R.FDEILEASDGIMVAR.G + Oxidation (M)
1821.9152 1820.9079 1820.9091 -0.62 278 - 293 1 R.RFDEILEASDGIMVAR.G
1828.0106 1827.0033 1827.0142 -5.94 294 - 310 1 R.GDLGIEIPAEKVFLAQK.M
1837.9033 1836.8960 1836.9040 -4.33 278 - 293 1 R.RFDEILEASDGIMVAR.G + Oxidation (M)
2391.2585 2390.2512 2390.2529 -0.71 504 - 525 1 K.KGDVVIVLTGWRPGSGFTNTMR.V
2407.2490 2406.2417 2406.2478 -2.54 504 - 525 1 K.KGDVVIVLTGWRPGSGFTNTMR.V + Oxidation (M)
2543.2600 2542.2527 2542.2526 0.04 467 - 488 1 R.GIFPVVCKDPVQEAWAEDVDLR.V
No match to: 1232.5408, 1320.5782, 1447.8284, 1456.7817, 1496.8522, 1564.7028, 1583.8815, 1586.8365,
1618.7668, 1622.6923,
1640.6997, 1643.7584, 1668.7452, 1693.7264, 1703.9412, 1708.8972, 1719.9355, 1790.8999, 1803.9180,
1858.9102, 1874.9021,
1883.8992, 1987.0120, 2003.0203, 2045.0212, 2051.0466, 2059.0266, 2063.0315, 2146.0615, 2250.1206,
2268.1143, 2286.1282,
2314.1538, 2347.1199, 2365.1323, 2373.1650, 2475.2803, 2493.2866, 2501.2695, 2529.3208, 2571.2886,
2595.1741, 2612.1775,
2623.2139, 3045.6150, 3153.4890, 3596.6897
16. gi|15987978 Mass: 58387 Score: 86 Expect: 0.003 Matches: 18
Chain A, S402p Mutant Of Rabbit Muscle Pyruvate Kinase
Observed Mr(expt) Mr(calc) ppm Start End Miss Peptide
868.4825 867.4752 867.4749 0.41 376 - 382 0 R.MQHLIAR.E
877.4737 876.4664 876.4705 -4.64 393 - 399 0 K.LFEELAR.A
884.4753 883.4680 883.4698 -2.00 376 - 382 0 R.MQHLIAR.E + Oxidation (M)
1005.5708 1004.5635 1004.5654 -1.91 392 - 399 1 R.KLFEELAR.A
1019.5126 1018.5053 1018.5083 -2.97 367 - 375 0 K.GDYPLEAVR.M
1061.4825 1060.4752 1060.4760 -0.72 383 - 391 0 R.EAEAAMFHR.K
1077.4830 1076.4757 1076.4709 4.48 383 - 391 0 R.EAEAAMFHR.K + Oxidation (M)
1197.6399 1196.6326 1196.5747 48.4 141 - 150 0 K.ITLDNAYMEK.C
1359.6973 1358.6900 1358.6976 -5.60 43 - 55 0 R.NTGIICTIGPASR.S
1394.7686 1393.7613 1393.7677 -4.60 266 - 277 1 K.IISKIENHEGVR.R
1473.8091 1472.8018 1472.8021 -0.17 422 - 435 0 K.CLAAALIVLTESGR.S
1681.8047 1680.7974 1680.8029 -3.24 279 - 293 0 R.FDEILEASDGIMVAR.G + Oxidation (M)
1821.9152 1820.9079 1820.9091 -0.62 278 - 293 1 R.RFDEILEASDGIMVAR.G
1828.0106 1827.0033 1827.0142 -5.94 294 - 310 1 R.GDLGIEIPAEKVFLAQK.M
1837.9033 1836.8960 1836.9040 -4.33 278 - 293 1 R.RFDEILEASDGIMVAR.G + Oxidation (M)
2391.2585 2390.2512 2390.2529 -0.71 504 - 525 1 K.KGDVVIVLTGWRPGSGFTNTMR.V
2407.2490 2406.2417 2406.2478 -2.54 504 - 525 1 K.KGDVVIVLTGWRPGSGFTNTMR.V + Oxidation (M)
2543.2600 2542.2527 2542.2526 0.04 467 - 488 1 R.GIFPVVCKDPVQEAWAEDVDLR.V
No match to: 1232.5408, 1320.5782, 1447.8284, 1456.7817, 1496.8522, 1564.7028, 1583.8815, 1586.8365,
1618.7668, 1622.6923,
1640.6997, 1643.7584, 1668.7452, 1693.7264, 1703.9412, 1708.8972, 1719.9355, 1790.8999, 1803.9180,
1858.9102, 1874.9021,
1883.8992, 1987.0120, 2003.0203, 2045.0212, 2051.0466, 2059.0266, 2063.0315, 2146.0615, 2250.1206,
2268.1143, 2286.1282,
2314.1538, 2347.1199, 2365.1323, 2373.1650, 2475.2803, 2493.2866, 2501.2695, 2529.3208, 2571.2886,
2595.1741, 2612.1775,
2623.2139, 3045.6150, 3153.4890, 3596.6897
17. gi|15987970 Mass: 58377 Score: 86 Expect: 0.003 Matches: 18
Chain A, Recombinant Rabbit Muscle Pyruvate Kinase
Observed Mr(expt) Mr(calc) ppm Start End Miss Peptide
868.4825 867.4752 867.4749 0.41 376 - 382 0 R.MQHLIAR.E
877.4737 876.4664 876.4705 -4.64 393 - 399 0 K.LFEELAR.A
884.4753 883.4680 883.4698 -2.00 376 - 382 0 R.MQHLIAR.E + Oxidation (M)
1005.5708 1004.5635 1004.5654 -1.91 392 - 399 1 R.KLFEELAR.A
1019.5126 1018.5053 1018.5083 -2.97 367 - 375 0 K.GDYPLEAVR.M
1061.4825 1060.4752 1060.4760 -0.72 383 - 391 0 R.EAEAAMFHR.K
1077.4830 1076.4757 1076.4709 4.48 383 - 391 0 R.EAEAAMFHR.K + Oxidation (M)
1197.6399 1196.6326 1196.5747 48.4 141 - 150 0 K.ITLDNAYMEK.C
1359.6973 1358.6900 1358.6976 -5.60 43 - 55 0 R.NTGIICTIGPASR.S
1394.7686 1393.7613 1393.7677 -4.60 266 - 277 1 K.IISKIENHEGVR.R
1473.8091 1472.8018 1472.8021 -0.17 422 - 435 0 K.CLAAALIVLTESGR.S
1681.8047 1680.7974 1680.8029 -3.24 279 - 293 0 R.FDEILEASDGIMVAR.G + Oxidation (M)
1821.9152 1820.9079 1820.9091 -0.62 278 - 293 1 R.RFDEILEASDGIMVAR.G
1828.0106 1827.0033 1827.0142 -5.94 294 - 310 1 R.GDLGIEIPAEKVFLAQK.M
1837.9033 1836.8960 1836.9040 -4.33 278 - 293 1 R.RFDEILEASDGIMVAR.G + Oxidation (M)
2391.2585 2390.2512 2390.2529 -0.71 504 - 525 1 K.KGDVVIVLTGWRPGSGFTNTMR.V
2407.2490 2406.2417 2406.2478 -2.54 504 - 525 1 K.KGDVVIVLTGWRPGSGFTNTMR.V + Oxidation (M)
2543.2600 2542.2527 2542.2526 0.04 467 - 488 1 R.GIFPVVCKDPVQEAWAEDVDLR.V

No match to: 1232.5408, 1320.5782, 1447.8284, 1456.7817, 1496.8522, 1564.7028, 1583.8815, 1586.8365,
1618.7668, 1622.6923,
1640.6997, 1643.7584, 1668.7452, 1693.7264, 1703.9412, 1708.8972, 1719.9355, 1790.8999, 1803.9180,
1858.9102, 1874.9021,
1883.8992, 1987.0120, 2003.0203, 2045.0212, 2051.0466, 2059.0266, 2063.0315, 2146.0615, 2250.1206,
2268.1143, 2286.1282,
2314.1538, 2347.1199, 2365.1323, 2373.1650, 2475.2803, 2493.2866, 2501.2695, 2529.3208, 2571.2886,
2595.1741, 2612.1775,
2623.2139, 3045.6150, 3153.4890, 3596.6897
18. gi|301598638 Mass: 58496 Score: 85 Expect: 0.0033 Matches: 18
Chain A, The Structure Of Muscle Pyruvate Kinase In Complex With Proline, Pyruvate, And Mn2+
Observed Mr(expt) Mr(calc) ppm Start End Miss Peptide
868.4825 867.4752 867.4749 0.41 377 - 383 0 R.MQHLIAR.E
877.4737 876.4664 876.4705 -4.64 394 - 400 0 K.LFEELAR.A
884.4753 883.4680 883.4698 -2.00 377 - 383 0 R.MQHLIAR.E + Oxidation (M)
1005.5708 1004.5635 1004.5654 -1.91 393 - 400 1 R.KLFEELAR.A
1019.5126 1018.5053 1018.5083 -2.97 368 - 376 0 K.GDYPLEAVR.M
1061.4825 1060.4752 1060.4760 -0.72 384 - 392 0 R.EAEAAMFHR.K
1077.4830 1076.4757 1076.4709 4.48 384 - 392 0 R.EAEAAMFHR.K + Oxidation (M)
1197.6399 1196.6326 1196.5747 48.4 142 - 151 0 K.ITLDNAYMEK.C
1359.6973 1358.6900 1358.6976 -5.60 44 - 56 0 R.NTGIICTIGPASR.S
1394.7686 1393.7613 1393.7677 -4.60 267 - 278 1 K.IISKIENHEGVR.R
1473.8091 1472.8018 1472.8021 -0.17 423 - 436 0 K.CLAAALIVLTESGR.S
1681.8047 1680.7974 1680.8029 -3.24 280 - 294 0 R.FDEILEASDGIMVAR.G + Oxidation (M)
1821.9152 1820.9079 1820.9091 -0.62 279 - 294 1 R.RFDEILEASDGIMVAR.G
1828.0106 1827.0033 1827.0142 -5.94 295 - 311 1 R.GDLGIEIPAEKVFLAQK.M
1837.9033 1836.8960 1836.9040 -4.33 279 - 294 1 R.RFDEILEASDGIMVAR.G + Oxidation (M)
2391.2585 2390.2512 2390.2529 -0.71 505 - 526 1 K.KGDVVIVLTGWRPGSGFTNTMR.V
2407.2490 2406.2417 2406.2478 -2.54 505 - 526 1 K.KGDVVIVLTGWRPGSGFTNTMR.V + Oxidation (M)
2543.2600 2542.2527 2542.2526 0.04 468 - 489 1 R.GIFPVVCKDPVQEAWAEDVDLR.V
No match to: 1232.5408, 1320.5782, 1447.8284, 1456.7817, 1496.8522, 1564.7028, 1583.8815, 1586.8365,
1618.7668, 1622.6923,
1640.6997, 1643.7584, 1668.7452, 1693.7264, 1703.9412, 1708.8972, 1719.9355, 1790.8999, 1803.9180,
1858.9102, 1874.9021,
1883.8992, 1987.0120, 2003.0203, 2045.0212, 2051.0466, 2059.0266, 2063.0315, 2146.0615, 2250.1206,
2268.1143, 2286.1282,
2314.1538, 2347.1199, 2365.1323, 2373.1650, 2475.2803, 2493.2866, 2501.2695, 2529.3208, 2571.2886,
2595.1741, 2612.1775,
2623.2139, 3045.6150, 3153.4890, 3596.6897
19. gi|2851533 Mass: 58524 Score: 85 Expect: 0.0037 Matches: 18
RecName: Full=Pyruvate kinase isozymes M1/M2; AltName: Full=Pyruvate kinase muscle isozyme
Observed Mr(expt) Mr(calc) ppm Start End Miss Peptide
868.4825 867.4752 867.4749 0.41 377 - 383 0 R.MQHLIAR.E
877.4737 876.4664 876.4705 -4.64 394 - 400 0 K.LFEELAR.S
884.4753 883.4680 883.4698 -2.00 377 - 383 0 R.MQHLIAR.E + Oxidation (M)
1005.5708 1004.5635 1004.5654 -1.91 393 - 400 1 R.KLFEELAR.S
1019.5126 1018.5053 1018.5083 -2.97 368 - 376 0 K.GDYPLEAVR.M
1061.4825 1060.4752 1060.4760 -0.72 384 - 392 0 R.EAEAAMFHR.K
1077.4830 1076.4757 1076.4709 4.48 384 - 392 0 R.EAEAAMFHR.K + Oxidation (M)
1197.6399 1196.6326 1196.5747 48.4 142 - 151 0 K.ITLDNAYMEK.C
1359.6973 1358.6900 1358.6976 -5.60 44 - 56 0 R.NTGIICTIGPASR.S
1394.7686 1393.7613 1393.7677 -4.60 267 - 278 1 K.IISKIENHEGVR.R
1473.8091 1472.8018 1472.8021 -0.17 423 - 436 0 K.CLAAALIVLTESGR.S
1681.8047 1680.7974 1680.8029 -3.24 280 - 294 0 R.FDEILEASDGIMVAR.G + Oxidation (M)
1821.9152 1820.9079 1820.9091 -0.62 279 - 294 1 R.RFDEILEASDGIMVAR.G
1828.0106 1827.0033 1827.0142 -5.94 295 - 311 1 R.GDLGIEIPAEKVFLAQK.M
1837.9033 1836.8960 1836.9040 -4.33 279 - 294 1 R.RFDEILEASDGIMVAR.G + Oxidation (M)
2391.2585 2390.2512 2390.2529 -0.71 505 - 526 1 K.KGDVVIVLTGWRPGSGFTNTMR.V
2407.2490 2406.2417 2406.2478 -2.54 505 - 526 1 K.KGDVVIVLTGWRPGSGFTNTMR.V + Oxidation (M)
2543.2600 2542.2527 2542.2526 0.04 468 - 489 1 R.GIFPVVCKDPVQEAWAEDVDLR.V
No match to: 1232.5408, 1320.5782, 1447.8284, 1456.7817, 1496.8522, 1564.7028, 1583.8815, 1586.8365,
1618.7668, 1622.6923,
1640.6997, 1643.7584, 1668.7452, 1693.7264, 1703.9412, 1708.8972, 1719.9355, 1790.8999, 1803.9180,
1858.9102, 1874.9021,
1883.8992, 1987.0120, 2003.0203, 2045.0212, 2051.0466, 2059.0266, 2063.0315, 2146.0615, 2250.1206,
2268.1143, 2286.1282,
2314.1538, 2347.1199, 2365.1323, 2373.1650, 2475.2803, 2493.2866, 2501.2695, 2529.3208, 2571.2886,
2595.1741, 2612.1775,
2623.2139, 3045.6150, 3153.4890, 3596.6897
20. gi|307548866 Mass: 58508 Score: 85 Expect: 0.0037 Matches: 18
pyruvate kinase isozymes M1/M2 isoform 1 [Oryctolagus cuniculus]
Observed Mr(expt) Mr(calc) ppm Start End Miss Peptide
868.4825 867.4752 867.4749 0.41 377 - 383 0 R.MQHLIAR.E
877.4737 876.4664 876.4705 -4.64 394 - 400 0 K.LFEELAR.A

884.4753 883.4680 883.4698 -2.00 377 - 383 0 R.MQHLIAR.E + Oxidation (M)
1005.5708 1004.5635 1004.5654 -1.91 393 - 400 1 R.KLFEELAR.A
1019.5126 1018.5053 1018.5083 -2.97 368 - 376 0 K.GDYPLEAVR.M
1061.4825 1060.4752 1060.4760 -0.72 384 - 392 0 R.EAEAAMFHR.K
1077.4830 1076.4757 1076.4709 4.48 384 - 392 0 R.EAEAAMFHR.K + Oxidation (M)
1197.6399 1196.6326 1196.5747 48.4 142 - 151 0 K.ITLDNAYMEK.C
1359.6973 1358.6900 1358.6976 -5.60 44 - 56 0 R.NTGIICTIGPASR.S
1394.7686 1393.7613 1393.7677 -4.60 267 - 278 1 K.IISKIENHEGVR.R
1473.8091 1472.8018 1472.8021 -0.17 423 - 436 0 K.CLAAALIVLTESGR.S
1681.8047 1680.7974 1680.8029 -3.24 280 - 294 0 R.FDEILEASDGIMVAR.G + Oxidation (M)
1821.9152 1820.9079 1820.9091 -0.62 279 - 294 1 R.RFDEILEASDGIMVAR.G
1828.0106 1827.0033 1827.0142 -5.94 295 - 311 1 R.GDLGIEIPAEKVFLAQK.M
1837.9033 1836.8960 1836.9040 -4.33 279 - 294 1 R.RFDEILEASDGIMVAR.G + Oxidation (M)
2391.2585 2390.2512 2390.2529 -0.71 505 - 526 1 K.KGDVVIVLTGWRPGSGFTNTMR.V
2407.2490 2406.2417 2406.2478 -2.54 505 - 526 1 K.KGDVVIVLTGWRPGSGFTNTMR.V + Oxidation (M)
2543.2600 2542.2527 2542.2526 0.04 468 - 489 1 R.GIFPVVCKDPVQEAWAEDVDLR.V
No match to: 1232.5408, 1320.5782, 1447.8284, 1456.7817, 1496.8522, 1564.7028, 1583.8815, 1586.8365, 1618.7668, 1622.6923,
1640.6997, 1643.7584, 1668.7452, 1693.7264, 1703.9412, 1708.8972, 1719.9355, 1790.8999, 1803.9180, 1858.9102, 1874.9021,
1883.8992, 1987.0120, 2003.0203, 2045.0212, 2051.0466, 2059.0266, 2063.0315, 2146.0615, 2250.1206, 2268.1143, 2286.1282,
2314.1538, 2347.1199, 2365.1323, 2373.1650, 2475.2803, 2493.2866, 2501.2695, 2529.3208, 2571.2886, 2595.1741, 2612.1775,
2623.2139, 3045.6150, 3153.4890, 3596.6897

Search Parameters

Type of search : Peptide Mass Fingerprint
Enzyme : Trypsin
Fixed modifications : Carbamidomethyl (C)
Variable modifications : Oxidation (M)
Mass values : Monoisotopic
Protein Mass : Unrestricted
Peptide Mass Tolerance : ± 50 ppm
Peptide Charge State : 1+
Max Missed Cleavages : 1
Number of queries : 65

Mascot: http://www.matrixscience.com/

COVERAGE BAND 7

Mascot Search Results

Protein View

Match to: gi|329664500 Score: 151 Expect: 8.4e-010
pyruvate kinase isozymes M1/M2 [Bos taurus]
Nominal mass (Mr): 58482; Calculated pI value: 7.96
NCBI BLAST search of gi|329664500 against nr
Unformatted sequence string for pasting into other applications
Taxonomy: Bos taurus
Links to retrieve other entries containing this sequence from NCBI Entrez:
gi|146231736 from Bos taurus
gi|296483716 from Bos taurus
Fixed modifications: Carbamidomethyl (C)
Variable modifications: Oxidation (M)
Cleavage by Trypsin: cuts C-term side of KR unless next residue is P
Number of mass values searched: 65
Number of mass values matched: 23
Sequence Coverage: 39%
Matched peptides shown in Bold Red
1 MSKHHSDAGT AFIQTQQLHA AMADTFLEHM CRLDIDSPPI TARNTGIICT
51 IGPASRAVET LKEMIKSGMN VARLNFSHGT HEYHAETIKN VREATESFAS
101 DPILYRPVAV ALDTKGPEIR TGLIKGSGTA EVELKKGATL KITLDNAYME
151 KCDENILWLD YKNICKVVDV GSKIYVDDGL ISLLVKQKGP DFLVTEVENG
201 GSLGSKKGVN LPGAAVDLPA VSEKDIQDLK FGVEQNVDMV FASFIRKASD
251 VHEVRKVLGE KGKNIKIISK IENHEGVRRF DEILEASDGI MVARGDLGIE
301 IPAEKVFLAQ KMMIGRCNRA GKPVICATQM LESMIKKPRP TRAEGSDVAN
351 AVLDGADCIM LSGETAKGDY PLEAVRMQHL IAREAEAAIY HLQLFEELRR
401 LSPITSDPTE AAAVGAVEAS FKCCSGAIIV LTKSGRSAHQ VARYRPRAPI
451 IAVTRNHQTA RQAHLYRGIF PVVCKDPVQE AWAEDVDLRV NLAMNVGKAR
501 GFFKKGDVVI VLTGWRPGSG FTNTMRVVPV P
Show predicted peptides also
Sort Peptides By Residue Number Increasing Mass Decreasing Mass
Start - End Observed Mr(expt) Mr(calc) ppm Miss Sequence
33 - 43 1197.6399 1196.6326 1196.6401 -6 0 R.LDIDSPPITAR.N

```
44 - 56 1359.6973 1358.6900 1358.6976 -6 0 R.NTGIICTIGPASR.A
74 - 89 1883.8992 1882.8919 1882.8962 -2 0 R.LNFSHGTHEYHAETIK.N
93 - 115 2493.2866 2492.2793 2492.2798 -0 0 R.EATESFASDPILYRPVAVALDTK.G
93 - 120 3045.6150 3044.6077 3044.5818 9 1 R.EATESFASDPILYRPVAVALDTKGPEIR.T
174 - 186 1447.8284 1446.8211 1446.8334 -8 0 K.IYVDDGLISLLVK.Q
174 - 188 1703.9412 1702.9339 1702.9869 -31 1 K.IYVDDGLISLLVKQK.G
225 - 246 2571.2886 2570.2813 2570.2839 -1 1 K.DIQDLKFGVEQNVDMVFASFIR.K
231 - 246 1858.9102 1857.9029 1857.9084 -3 0 K.FGVEQNVDMVFASFIR.K
231 - 246 1874.9021 1873.8948 1873.9033 -5 0 K.FGVEQNVDMVFASFIR.K Oxidation (M)
231 - 247 1987.0120 1986.0047 1986.0033 1 1 K.FGVEQNVDMVFASFIRK.A
231 - 247 2003.0203 2002.0130 2001.9982 7 1 K.FGVEQNVDMVFASFIRK.A Oxidation (M)
267 - 278 1394.7686 1393.7613 1393.7677 -5 1 K.IISKIENHEGVR.R
279 - 294 1821.9152 1820.9079 1820.9091 -1 1 R.RFDEILEASDGIMVAR.G
279 - 294 1837.9033 1836.8960 1836.9040 -4 1 R.RFDEILEASDGIMVAR.G Oxidation (M)
280 - 294 1681.8047 1680.7974 1680.8029 -3 0 R.FDEILEASDGIMVAR.G Oxidation (M)
295 - 311 1828.0106 1827.0033 1827.0142 -6 1 R.GDLGIEIPAEKVFLAQK.M
368 - 376 1019.5126 1018.5053 1018.5083 -3 0 K.GDYPLEAVR.M
377 - 383 868.4825 867.4752 867.4749 0 0 R.MQHLIAR.E
377 - 383 884.4753 883.4680 883.4698 -2 0 R.MQHLIAR.E Oxidation (M)
468 - 489 2543.2600 2542.2527 2542.2526 0 1 R.GIFPVVCKDPVQEAWAEDVDLR.V
505 - 526 2391.2585 2390.2512 2390.2529 -1 1 K.KGDVVIVLTGWRPGSGFTNTMR.V
505 - 526 2407.2490 2406.2417 2406.2478 -3 1 K.KGDVVIVLTGWRPGSGFTNTMR.V Oxidation (M)
No match to: 877.4737, 1005.5708, 1061.4825, 1077.4830, 1232.5408, 1320.5782, 1456.7817, 1473.8091,
1496.8522, 1564.7028, 1583.8
```

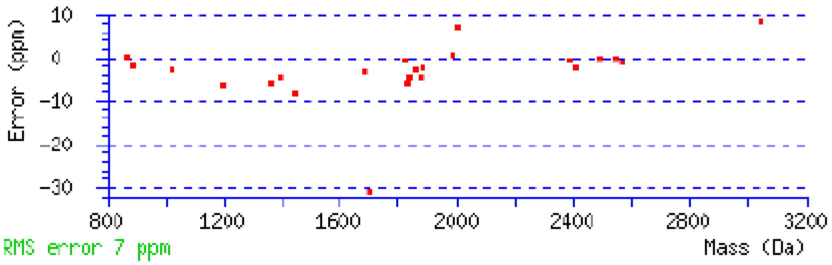

BAND 8

Mascot Search Results

Mascot Search Results

User :
Email :
Search title : SampleSetID: 824, AnalysisID: 7242, MaldiWellID: 69611, SpectrumID: 154536, Path=\180719\MS\18-106 NCBI Mammalia
Database : NCBInr 20120508 (17919084 sequences; 6150218869 residues)
Taxonomy : Mammalia (mammals) (1061927 sequences)
Timestamp : 19 Jul 2018 at 12:11:53 GMT
Top Score : 127 for Mixture 1, gi|297343122 + gi|77736349

Mascot Score Histogram

Protein score is -10*Log(P), where P is the probability that the observed match is a random event.
Protein scores greater than 73 are significant (p<0.05).

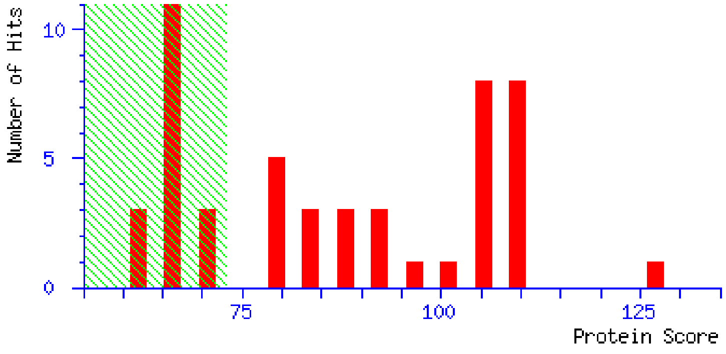

Protein Summary Report

Format As Protein Summary Help
Significance threshold p< 0.05 Max. number of hits 20
Re-Search All Search Unmatched

Index

Accession Mass Score Description
1. Mixture 1 127 gi|297343122 + gi|77736349
2. gi|297343122 40304 111 Chain A, Structures Of Actin-Bound Wh2 Domains Of Spire And The Implication For Filament Nucleation
3. gi|49864 38016 110 alpha-actin (aa 40-375) [Mus musculus]
4. gi|387082 37990 110 skeletal muscle alpha-actin, partial [Mus musculus]
5. gi|20664362 41558 109 Chain A, Polylysine Induces An Antiparallel Actin Dimer That Nucleates Filament Assembly: Crystal Structure At 3.5 A Resolution
6. gi|313507127 41725 109 Chain A, Atomic Structure Of The Actin:dnase I Complex
7. gi|114794125 41561 108 Chain A, Spvb Adp-Ribosylated Actin: Hexagonal Crystal Form
8. gi|295789238 41640 108 Chain D, Isometrically Contracting Insect Asynchronous Flight Muscle
9. gi|310942658 42160 108 Chain O, Cryo-Em Structure Of Actin Filament In The Presence Of Phosphate
10. gi|55669843 41486 107 Chain A, Structural Basis Of Actin Sequestration By Thymosin-B4: Implications For Arp23 ACTIVATION
11. gi|21730554 42075 107 Chain A, Crystal Structure Of Human Vitamin D-Binding Protein In Complex With Skeletal Actin
12. gi|39654752 42049 107 Chain A, Structure Of Rabbit Actin In Complex With Kabiramide C
13. gi|4501881 42366 106 actin, alpha skeletal muscle [Homo sapiens]
14. gi|7766848 42340 106 Chain A, Complex Between Rabbit Muscle Alpha-Actin: Human Gelsolin Domain 1
15. gi|134024776 42338 106 Actin, alpha 1, skeletal muscle [Bos taurus]
16. gi|291413356 42420 106 PREDICTED: cytoplasmic beta-actin [Oryctolagus cuniculus]
17. gi|332812177 42396 106 PREDICTED: actin, alpha skeletal muscle [Pan troglodytes]
18. gi|296230233 37576 100 PREDICTED: actin, alpha skeletal muscle isoform 4 [Callithrix jacchus]
19. gi|306440544 41484 96 Chain A, Crystal Structure Of Actin In Complex With Lobophorolide
20. gi|296230231 38909 93 PREDICTED: actin, alpha skeletal muscle isoform 3 [Callithrix jacchus]

Results List

1. Mixture 1 Total score: 127 Expect: 2.1e-007 Matches: 30
Components: 1. gi|297343122 Chain A, Structures Of Actin-Bound Wh2 Domains Of Spire And The Implication For Filament Nucleation
2. gi|77736349 beta-enolase [Bos taurus]

Observed	Mr(expt)	Mr(calc)	ppm	Start	End	Miss	Comp	Peptide
1475.7252	1474.7179	1474.7780	-40.71	413 -	426	1	2	R.IEEALGDKAVFAGR.K
1500.6478	1499.6405	1499.7005	-39.96	344 -	356	0	1	K.QEYDEAGPSIVHR.K
1515.6893	1514.6820	1514.7419	-39.51	69 -	79	0	1	K.IWHHTFYNELR.V
1541.7109	1540.7036	1540.7569	-34.57	359 -	372	0	2	K.LAQSNGWGVMVSHR.S
1556.7256	1555.7183	1555.7705	-33.51	240 -	253	0	2	K.VVIGMDVAASEFYR.N
1623.7876	1622.7803	1622.8338	-32.93	162 -	175	1	1	R.LDLAGRDLTDYLMK.I
1639.7745	1638.7672	1638.8287	-37.51	162 -	175	1	1	R.LDLAGRDLTDYLMK.I + Oxidation (M)
1790.8533	1789.8460	1789.8846	-21.57	223 -	238	0	1	K.SYELPDGQVITIGNER.F
1804.9034	1803.8961	1803.9366	-22.46	33 -	50	0	2	R.AAVPSGASTGIYEALELR.D
1896.9443	1895.9370	1895.9637	-14.09	163 -	179	0	2	K.LAMQEFMILPVGASSFR.E
1912.9381	1911.9308	1911.9587	-14.56	163 -	179	0	2	K.LAMQEFMILPVGASSFR.E + Oxidation (M)
1928.9333	1927.9260	1927.9536	-14.29	163 -	179	0	2	K.LAMQEFMILPVGASSFR.E + 2 Oxidation (M)
1956.0134	1955.0061	1955.0364	-15.47	80 -	97	0	1	R.VAPEEHPTLLTEAPLNPK.A
2094.0361	2093.0288	2093.0542	-12.10	223 -	240	1	1	K.SYELPDGQVITIGNERFR.C
2108.0635	2107.0562	2107.1062	-23.70	31 -	50	1	2	R.FRAAVPSGASTGIYEALELR.D
2246.0178	2245.0105	2245.0143	-1.70	276 -	296	0	1	K.DLYANNVMSGGTTMYPGIADR.M
2262.0215	2261.0142	2261.0093	2.19	276 -	296	0	1	K.DLYANNVMSGGTTMYPGIADR.M + Oxidation (M)
2278.0410	2277.0337	2277.0042	13.0	276 -	296	0	1	K.DLYANNVMSGGTTMYPGIADR.M + 2 Oxidation (M)
2374.1279	2373.1206	2373.1093	4.77	275 -	296	1	1	R.KDLYANNVMSGGTTMYPGIADR.M
2390.1262	2389.1189	2389.1042	6.16	275 -	296	1	1	R.KDLYANNVMSGGTTMYPGIADR.M + Oxidation (M)
2406.1243	2405.1170	2405.0991	7.44	275 -	296	1	1	R.KDLYANNVMSGGTTMYPGIADR.M + 2 Oxidation (M)
2536.1880	2535.1807	2535.1509	11.8	200 -	222	0	1	K.LCYVALDFENEMATAASSSSLEK.S
2616.3828	2615.3755	2615.3458	11.4	321 -	343	0	1	K.YSVWIGGSILASLSTFQQMWITK.Q
2672.3794	2671.3721	2671.3316	15.2	229 -	253	1	2	K.TAIQAAGYPDKVVIGMDVAASEFYR.N
2688.3799	2687.3726	2687.3265	17.2	229 -	253	1	2	K.TAIQAAGYPDKVVIGMDVAASEFYR.N + Oxidation (M)
2743.3975	2742.3902	2742.3348	20.2	203 -	228	0	2	K.DATNVGDEGGFAPNILENNEALELLK.T
3035.7068	3034.6995	3034.5988	33.2	133 -	162	0	2	R.HIADLAGNPELILPVPAFNVINGGSHAGNK.L
3196.7109	3195.7036	3195.6023	31.7	132 -	161	0	1	R.TTGIVLDSGDGVTHNVPIYEGYALPHAIMR.L
3212.7434	3211.7361	3211.5972	43.3	132 -	161	0	1	R.TTGIVLDSGDGVTHNVPIYEGYALPHAIMR.L + Oxidation (M)
3491.8062	3490.7989	3490.6360	46.7	239 -	268	1	1	R.FRCPETLFQPSFIGMESAGIHETTYNSIMK.C

No match to: 945.4556, 976.3532, 1130.4626, 1181.5842, 1198.6243, 1320.5065, 1354.5515, 1483.6229, 1528.6823, 1542.6951, 1543.7032, 1547.6823,
1558.6926, 1634.7444, 1642.6931, 1768.9558, 1772.8760, 1818.8853, 1832.9414, 1838.8187, 1848.9365, 1864.9501, 1918.9548, 1944.9376, 1964.8632,
1974.9027, 1984.0460, 2088.0190, 2105.0481, 2327.1223, 2600.3669, 3036.6846, 3224.7659, 3471.7959, 3487.8362

2. gi|297343122 Mass: 40304 Score: 111 Expect: 8.4e-006 Matches: 18
Chain A, Structures Of Actin-Bound Wh2 Domains Of Spire And The Implication For Filament Nucleation

Observed	Mr(expt)	Mr(calc)	ppm	Start	End	Miss	Peptide
1500.6478	1499.6405	1499.7005	-39.96	344 -	356	0	K.QEYDEAGPSIVHR.K
1515.6893	1514.6820	1514.7419	-39.51	69 -	79	0	K.IWHHTFYNELR.V
1623.7876	1622.7803	1622.8338	-32.93	162 -	175	1	R.LDLAGRDLTDYLMK.I
1639.7745	1638.7672	1638.8287	-37.51	162 -	175	1	R.LDLAGRDLTDYLMK.I + Oxidation (M)
1790.8533	1789.8460	1789.8846	-21.57	223 -	238	0	K.SYELPDGQVITIGNER.F
1956.0134	1955.0061	1955.0364	-15.47	80 -	97	0	R.VAPEEHPTLLTEAPLNPK.A
2094.0361	2093.0288	2093.0542	-12.10	223 -	240	1	K.SYELPDGQVITIGNERFR.C
2246.0178	2245.0105	2245.0143	-1.70	276 -	296	0	K.DLYANNVMSGGTTMYPGIADR.M

2262.0215 2261.0142 2261.0093 2.19 276 - 296 0 K.DLYANNVMSGGTTMYPGIADR.M + Oxidation (M)
2278.0410 2277.0337 2277.0042 13.0 276 - 296 0 K.DLYANNVMSGGTTMYPGIADR.M + 2 Oxidation (M)
2374.1279 2373.1206 2373.1093 4.77 275 - 296 1 R.KDLYANNVMSGGTTMYPGIADR.M
2390.1262 2389.1189 2389.1042 6.16 275 - 296 1 R.KDLYANNVMSGGTTMYPGIADR.M + Oxidation (M)
2406.1243 2405.1170 2405.0991 7.44 275 - 296 1 R.KDLYANNVMSGGTTMYPGIADR.M + 2 Oxidation (M)
2536.1880 2535.1807 2535.1509 11.8 200 - 222 0 K.LCYVALDFENEMATAASSSSLEK.S
2616.3828 2615.3755 2615.3458 11.4 321 - 343 0 K.YSVWIGGSILASLSTFQQMWITK.Q
3196.7109 3195.7036 3195.6023 31.7 132 - 161 0 R.TTGIVLDSGDGVTHNVPIYEGYALPHAIMR.L
3212.7434 3211.7361 3211.5972 43.3 132 - 161 0 R.TTGIVLDSGDGVTHNVPIYEGYALPHAIMR.L + Oxidation (M)
3491.8062 3490.7989 3490.6360 46.7 239 - 268 1 R.FRCPETLFQPSFIGMESAGIHETTYNSIMK.C
No match to: 945.4556, 976.3532, 1130.4626, 1181.5842, 1198.6243, 1320.5065, 1354.5515, 1475.7252, 1483.6229, 1528.6823, 1541.7109, 1542.6951,
1543.7032, 1547.6823, 1556.7256, 1558.6926, 1634.7444, 1642.6931, 1768.9558, 1772.8760, 1804.9034, 1818.8853, 1832.9414, 1838.8187, 1848.9365,
1864.9501, 1896.9443, 1912.9381, 1918.9548, 1928.9333, 1944.9376, 1964.8632, 1974.9027, 1984.0460, 2088.0190, 2105.0481, 2108.0635, 2327.1223,
2600.3669, 2672.3794, 2688.3799, 2743.3975, 3035.7068, 3036.6846, 3224.7659, 3471.7959, 3487.8362
3. gi|49864 Mass: 38016 Score: 110 Expect: 1.1e-005 Matches: 18
alpha-actin (aa 40-375) [Mus musculus]
Observed Mr(expt) Mr(calc) ppm Start End Miss Peptide
1500.6478 1499.6405 1499.7005 -39.96 321 - 333 0 K.QEYDEAGPSIVHR.K
1515.6893 1514.6820 1514.7419 -39.51 46 - 56 0 K.IWHHTFYNELR.V
1623.7876 1622.7803 1622.8338 -32.93 139 - 152 1 R.LDLAGRDLTDYLMK.I
1639.7745 1638.7672 1638.8287 -37.51 139 - 152 1 R.LDLAGRDLTDYLMK.I + Oxidation (M)
1790.8533 1789.8460 1789.8846 -21.57 200 - 215 0 K.SYELPDGQVITIGNER.F
1956.0134 1955.0061 1955.0364 -15.47 57 - 74 0 R.VAPEEHPTLLTEAPLNPK.A
2094.0361 2093.0288 2093.0542 -12.10 200 - 217 1 K.SYELPDGQVITIGNERFR.C
2246.0178 2245.0105 2245.0143 -1.70 253 - 273 0 K.DLYANNVMSGGTTMYPGIADR.M
2262.0215 2261.0142 2261.0093 2.19 253 - 273 0 K.DLYANNVMSGGTTMYPGIADR.M + Oxidation (M)
2278.0410 2277.0337 2277.0042 13.0 253 - 273 0 K.DLYANNVMSGGTTMYPGIADR.M + 2 Oxidation (M)
2374.1279 2373.1206 2373.1093 4.77 252 - 273 1 R.KDLYANNVMSGGTTMYPGIADR.M
2390.1262 2389.1189 2389.1042 6.16 252 - 273 1 R.KDLYANNVMSGGTTMYPGIADR.M + Oxidation (M)
2406.1243 2405.1170 2405.0991 7.44 252 - 273 1 R.KDLYANNVMSGGTTMYPGIADR.M + 2 Oxidation (M)
2536.1880 2535.1807 2535.1509 11.8 177 - 199 0 K.LCYVALDFENEMATAASSSSLEK.S
2616.3828 2615.3755 2615.3458 11.4 298 - 320 0 K.YSVWIGGSILASLSTFQQMWITK.Q
3196.7109 3195.7036 3195.6023 31.7 109 - 138 0 R.TTGIVLDSGDGVTHNVPIYEGYALPHAIMR.L
3212.7434 3211.7361 3211.5972 43.3 109 - 138 0 R.TTGIVLDSGDGVTHNVPIYEGYALPHAIMR.L + Oxidation (M)
3491.8062 3490.7989 3490.6360 46.7 216 - 245 1 R.FRCPETLFQPSFIGMESAGIHETTYNSIMK.C
No match to: 945.4556, 976.3532, 1130.4626, 1181.5842, 1198.6243, 1320.5065, 1354.5515, 1475.7252, 1483.6229, 1528.6823, 1541.7109, 1542.6951,
1543.7032, 1547.6823, 1556.7256, 1558.6926, 1634.7444, 1642.6931, 1768.9558, 1772.8760, 1804.9034, 1818.8853, 1832.9414, 1838.8187, 1848.9365,
1864.9501, 1896.9443, 1912.9381, 1918.9548, 1928.9333, 1944.9376, 1964.8632, 1974.9027, 1984.0460, 2088.0190, 2105.0481, 2108.0635, 2327.1223,
2600.3669, 2672.3794, 2688.3799, 2743.3975, 3035.7068, 3036.6846, 3224.7659, 3471.7959, 3487.8362
4. gi|387082 Mass: 37990 Score: 110 Expect: 1.1e-005 Matches: 18
skeletal muscle alpha-actin, partial [Mus musculus]
Observed Mr(expt) Mr(calc) ppm Start End Miss Peptide
1500.6478 1499.6405 1499.7005 -39.96 321 - 333 0 K.QEYDEAGPSIVHR.K
1515.6893 1514.6820 1514.7419 -39.51 46 - 56 0 K.IWHHTFYNELR.V
1623.7876 1622.7803 1622.8338 -32.93 139 - 152 1 R.LDLAGRDLTDYLMK.I
1639.7745 1638.7672 1638.8287 -37.51 139 - 152 1 R.LDLAGRDLTDYLMK.I + Oxidation (M)
1790.8533 1789.8460 1789.8846 -21.57 200 - 215 0 K.SYELPDGQVITIGNER.F
1956.0134 1955.0061 1955.0364 -15.47 57 - 74 0 R.VAPEEHPTLLTEAPLNPK.A
2094.0361 2093.0288 2093.0542 -12.10 200 - 217 1 K.SYELPDGQVITIGNERFR.C
2246.0178 2245.0105 2245.0143 -1.70 253 - 273 0 K.DLYANNVMSGGTTMYPGIADR.M
2262.0215 2261.0142 2261.0093 2.19 253 - 273 0 K.DLYANNVMSGGTTMYPGIADR.M + Oxidation (M)
2278.0410 2277.0337 2277.0042 13.0 253 - 273 0 K.DLYANNVMSGGTTMYPGIADR.M + 2 Oxidation (M)
2374.1279 2373.1206 2373.1093 4.77 252 - 273 1 R.KDLYANNVMSGGTTMYPGIADR.M
2390.1262 2389.1189 2389.1042 6.16 252 - 273 1 R.KDLYANNVMSGGTTMYPGIADR.M + Oxidation (M)
2406.1243 2405.1170 2405.0991 7.44 252 - 273 1 R.KDLYANNVMSGGTTMYPGIADR.M + 2 Oxidation (M)
2536.1880 2535.1807 2535.1509 11.8 177 - 199 0 K.LCYVALDFENEMATAASSSSLEK.S
2616.3828 2615.3755 2615.3458 11.4 298 - 320 0 K.YSVWIGGSILASLSTFQQMWITK.Q
3196.7109 3195.7036 3195.6023 31.7 109 - 138 0 R.TTGIVLDSGDGVTHNVPIYEGYALPHAIMR.L
3212.7434 3211.7361 3211.5972 43.3 109 - 138 0 R.TTGIVLDSGDGVTHNVPIYEGYALPHAIMR.L + Oxidation (M)
3491.8062 3490.7989 3490.6360 46.7 216 - 245 1 R.FRCPETLFQPSFIGMESAGIHETTYNSIMK.C
No match to: 945.4556, 976.3532, 1130.4626, 1181.5842, 1198.6243, 1320.5065, 1354.5515, 1475.7252, 1483.6229, 1528.6823, 1541.7109, 1542.6951,
1543.7032, 1547.6823, 1556.7256, 1558.6926, 1634.7444, 1642.6931, 1768.9558, 1772.8760, 1804.9034, 1818.8853, 1832.9414, 1838.8187, 1848.9365,
1864.9501, 1896.9443, 1912.9381, 1918.9548, 1928.9333, 1944.9376, 1964.8632, 1974.9027, 1984.0460, 2088.0190, 2105.0481, 2108.0635, 2327.1223,
2600.3669, 2672.3794, 2688.3799, 2743.3975, 3035.7068, 3036.6846, 3224.7659, 3471.7959, 3487.8362
5. gi|20664362 Mass: 41558 Score: 109 Expect: 1.3e-005 Matches: 18
Chain A, Polylysine Induces An Antiparallel Actin Dimer That Nucleates Filament Assembly: Crystal Structure At 3.5 A Resolution
Observed Mr(expt) Mr(calc) ppm Start End Miss Peptide
1500.6478 1499.6405 1499.7005 -39.96 356 - 368 0 K.QEYDEAGPSIVHR.K
1515.6893 1514.6820 1514.7419 -39.51 81 - 91 0 K.IWHHTFYNELR.V
1623.7876 1622.7803 1622.8338 -32.93 174 - 187 1 R.LDLAGRDLTDYLMK.I
1639.7745 1638.7672 1638.8287 -37.51 174 - 187 1 R.LDLAGRDLTDYLMK.I + Oxidation (M)
1790.8533 1789.8460 1789.8846 -21.57 235 - 250 0 K.SYELPDGQVITIGNER.F
1956.0134 1955.0061 1955.0364 -15.47 92 - 109 0 R.VAPEEHPTLLTEAPLNPK.A
2094.0361 2093.0288 2093.0542 -12.10 235 - 252 1 K.SYELPDGQVITIGNERFR.C
2246.0178 2245.0105 2245.0143 -1.70 288 - 308 0 K.DLYANNVMSGGTTMYPGIADR.M

2262.0215 2261.0142 2261.0093 2.19 288 - 308 0 K.DLYANNVMSGGTTMYPGIADR.M + Oxidation (M)
2278.0410 2277.0337 2277.0042 13.0 288 - 308 0 K.DLYANNVMSGGTTMYPGIADR.M + 2 Oxidation (M)
2374.1279 2373.1206 2373.1093 4.77 287 - 308 1 R.KDLYANNVMSGGTTMYPGIADR.M
2390.1262 2389.1189 2389.1042 6.16 287 - 308 1 R.KDLYANNVMSGGTTMYPGIADR.M + Oxidation (M)
2406.1243 2405.1170 2405.0991 7.44 287 - 308 1 R.KDLYANNVMSGGTTMYPGIADR.M + 2 Oxidation (M)
2536.1880 2535.1807 2535.1509 11.8 212 - 234 0 K.LCYVALDFENEMATAASSSSLEK.S
2616.3828 2615.3755 2615.3458 11.4 333 - 355 0 K.YSVWIGGSILASLSTFQQMWITK.Q
3196.7109 3195.7036 3195.6023 31.7 144 - 173 0 R.TTGIVLDSGDGVTHNVPIYEGYALPHAIMR.L
3212.7434 3211.7361 3211.5972 43.3 144 - 173 0 R.TTGIVLDSGDGVTHNVPIYEGYALPHAIMR.L + Oxidation (M)
3491.8062 3490.7989 3490.6360 46.7 251 - 280 1 R.FRCPETLFQPSFIGMESAGIHETTYNSIMK.C
No match to: 945.4556, 976.3532, 1130.4626, 1181.5842, 1198.6243, 1320.5065, 1354.5515, 1475.7252, 1483.6229, 1528.6823, 1541.7109, 1542.6951,
1543.7032, 1547.6823, 1556.7256, 1558.6926, 1634.7444, 1642.6931, 1768.9558, 1772.8760, 1804.9034, 1818.8853, 1832.9414, 1838.8187, 1848.9365,
1864.9501, 1896.9443, 1912.9381, 1918.9548, 1928.9333, 1944.9376, 1964.8632, 1974.9027, 1984.0460, 2088.0190, 2105.0481, 2108.0635, 2327.1223,
2600.3669, 2672.3794, 2688.3799, 2743.3975, 3035.7068, 3036.6846, 3224.7659, 3471.7959, 3487.8362
6. gi|313507127 Mass: 41725 Score: 109 Expect: 1.3e-005 Matches: 18
Chain A, Atomic Structure Of The Actin:dnase I Complex
Observed Mr(expt) Mr(calc) ppm Start End Miss Peptide
1500.6478 1499.6405 1499.7005 -39.96 361 - 373 0 K.QEYDEAGPSIVHR.-
1515.6893 1514.6820 1514.7419 -39.51 86 - 96 0 K.IWHHTFYNELR.V
1623.7876 1622.7803 1622.8338 -32.93 179 - 192 1 R.LDLAGRDLTDYLMK.I
1639.7745 1638.7672 1638.8287 -37.51 179 - 192 1 R.LDLAGRDLTDYLMK.I + Oxidation (M)
1790.8533 1789.8460 1789.8846 -21.57 240 - 255 0 K.SYELPDGQVITIGNER.F
1956.0134 1955.0061 1955.0364 -15.47 97 - 114 0 R.VAPEEHPTLLTEAPLNPK.A
2094.0361 2093.0288 2093.0542 -12.10 240 - 257 1 K.SYELPDGQVITIGNERFR.C
2246.0178 2245.0105 2245.0143 -1.70 293 - 313 0 K.DLYANNVMSGGTTMYPGIADR.M
2262.0215 2261.0142 2261.0093 2.19 293 - 313 0 K.DLYANNVMSGGTTMYPGIADR.M + Oxidation (M)
2278.0410 2277.0337 2277.0042 13.0 293 - 313 0 K.DLYANNVMSGGTTMYPGIADR.M + 2 Oxidation (M)
2374.1279 2373.1206 2373.1093 4.77 292 - 313 1 R.KDLYANNVMSGGTTMYPGIADR.M
2390.1262 2389.1189 2389.1042 6.16 292 - 313 1 R.KDLYANNVMSGGTTMYPGIADR.M + Oxidation (M)
2406.1243 2405.1170 2405.0991 7.44 292 - 313 1 R.KDLYANNVMSGGTTMYPGIADR.M + 2 Oxidation (M)
2536.1880 2535.1807 2535.1509 11.8 217 - 239 0 K.LCYVALDFENEMATAASSSSLEK.S
2616.3828 2615.3755 2615.3458 11.4 338 - 360 0 K.YSVWIGGSILASLSTFQQMWITK.Q
3196.7109 3195.7036 3195.6023 31.7 149 - 178 0 R.TTGIVLDSGDGVTHNVPIYEGYALPHAIMR.L
3212.7434 3211.7361 3211.5972 43.3 149 - 178 0 R.TTGIVLDSGDGVTHNVPIYEGYALPHAIMR.L + Oxidation (M)
3491.8062 3490.7989 3490.6360 46.7 256 - 285 1 R.FRCPETLFQPSFIGMESAGIHETTYNSIMK.C
No match to: 945.4556, 976.3532, 1130.4626, 1181.5842, 1198.6243, 1320.5065, 1354.5515, 1475.7252, 1483.6229, 1528.6823, 1541.7109, 1542.6951,
1543.7032, 1547.6823, 1556.7256, 1558.6926, 1634.7444, 1642.6931, 1768.9558, 1772.8760, 1804.9034, 1818.8853, 1832.9414, 1838.8187, 1848.9365,
1864.9501, 1896.9443, 1912.9381, 1918.9548, 1928.9333, 1944.9376, 1964.8632, 1974.9027, 1984.0460, 2088.0190, 2105.0481, 2108.0635, 2327.1223,
2600.3669, 2672.3794, 2688.3799, 2743.3975, 3035.7068, 3036.6846, 3224.7659, 3471.7959, 3487.8362
7. gi|114794125 Mass: 41561 Score: 108 Expect: 1.7e-005 Matches: 18
Chain A, Spvb Adp-Ribosylated Actin: Hexagonal Crystal Form
Observed Mr(expt) Mr(calc) ppm Start End Miss Peptide
1500.6478 1499.6405 1499.7005 -39.96 356 - 368 0 K.QEYDEAGPSIVHR.K
1515.6893 1514.6820 1514.7419 -39.51 81 - 91 0 K.IWHHTFYNELR.V
1623.7876 1622.7803 1622.8338 -32.93 174 - 187 1 R.LDLAGRDLTDYLMK.I
1639.7745 1638.7672 1638.8287 -37.51 174 - 187 1 R.LDLAGRDLTDYLMK.I + Oxidation (M)
1790.8533 1789.8460 1789.8846 -21.57 235 - 250 0 K.SYELPDGQVITIGNER.F
1956.0134 1955.0061 1955.0364 -15.47 92 - 109 0 R.VAPEEHPTLLTEAPLNPK.A
2094.0361 2093.0288 2093.0542 -12.10 235 - 252 1 K.SYELPDGQVITIGNERFR.C
2246.0178 2245.0105 2245.0143 -1.70 288 - 308 0 K.DLYANNVMSGGTTMYPGIADR.M
2262.0215 2261.0142 2261.0093 2.19 288 - 308 0 K.DLYANNVMSGGTTMYPGIADR.M + Oxidation (M)
2278.0410 2277.0337 2277.0042 13.0 288 - 308 0 K.DLYANNVMSGGTTMYPGIADR.M + 2 Oxidation (M)
2374.1279 2373.1206 2373.1093 4.77 287 - 308 1 R.KDLYANNVMSGGTTMYPGIADR.M
2390.1262 2389.1189 2389.1042 6.16 287 - 308 1 R.KDLYANNVMSGGTTMYPGIADR.M + Oxidation (M)
2406.1243 2405.1170 2405.0991 7.44 287 - 308 1 R.KDLYANNVMSGGTTMYPGIADR.M + 2 Oxidation (M)
2536.1880 2535.1807 2535.1509 11.8 212 - 234 0 K.LCYVALDFENEMATAASSSSLEK.S
2616.3828 2615.3755 2615.3458 11.4 333 - 355 0 K.YSVWIGGSILASLSTFQQMWITK.Q
3196.7109 3195.7036 3195.6023 31.7 144 - 173 0 R.TTGIVLDSGDGVTHNVPIYEGYALPHAIMR.L
3212.7434 3211.7361 3211.5972 43.3 144 - 173 0 R.TTGIVLDSGDGVTHNVPIYEGYALPHAIMR.L + Oxidation (M)
3491.8062 3490.7989 3490.6360 46.7 251 - 280 1 R.FRCPETLFQPSFIGMESAGIHETTYNSIMK.C
No match to: 945.4556, 976.3532, 1130.4626, 1181.5842, 1198.6243, 1320.5065, 1354.5515, 1475.7252, 1483.6229, 1528.6823, 1541.7109, 1542.6951,
1543.7032, 1547.6823, 1556.7256, 1558.6926, 1634.7444, 1642.6931, 1768.9558, 1772.8760, 1804.9034, 1818.8853, 1832.9414, 1838.8187, 1848.9365,
1864.9501, 1896.9443, 1912.9381, 1918.9548, 1928.9333, 1944.9376, 1964.8632, 1974.9027, 1984.0460, 2088.0190, 2105.0481, 2108.0635, 2327.1223,
2600.3669, 2672.3794, 2688.3799, 2743.3975, 3035.7068, 3036.6846, 3224.7659, 3471.7959, 3487.8362
8. gi|295789238 Mass: 41640 Score: 108 Expect: 1.7e-005 Matches: 18
Chain D, Isometrically Contracting Insect Asynchronous Flight Muscle
Observed Mr(expt) Mr(calc) ppm Start End Miss Peptide
1500.6478 1499.6405 1499.7005 -39.96 360 - 372 0 K.QEYDEAGPSIVHR.-
1515.6893 1514.6820 1514.7419 -39.51 85 - 95 0 K.IWHHTFYNELR.V
1623.7876 1622.7803 1622.8338 -32.93 178 - 191 1 R.LDLAGRDLTDYLMK.I
1639.7745 1638.7672 1638.8287 -37.51 178 - 191 1 R.LDLAGRDLTDYLMK.I + Oxidation (M)
1790.8533 1789.8460 1789.8846 -21.57 239 - 254 0 K.SYELPDGQVITIGNER.F
1956.0134 1955.0061 1955.0364 -15.47 96 - 113 0 R.VAPEEHPTLLTEAPLNPK.A
2094.0361 2093.0288 2093.0542 -12.10 239 - 256 1 K.SYELPDGQVITIGNERFR.C
2246.0178 2245.0105 2245.0143 -1.70 292 - 312 0 K.DLYANNVMSGGTTMYPGIADR.M
2262.0215 2261.0142 2261.0093 2.19 292 - 312 0 K.DLYANNVMSGGTTMYPGIADR.M + Oxidation (M)

2278.0410 2277.0337 2277.0042 13.0 292 - 312 0 K.DLYANNVMSGGTTMYPGIADR.M + 2 Oxidation (M)
2374.1279 2373.1206 2373.1093 4.77 291 - 312 1 R.KDLYANNVMSGGTTMYPGIADR.M
2390.1262 2389.1189 2389.1042 6.16 291 - 312 1 R.KDLYANNVMSGGTTMYPGIADR.M + Oxidation (M)
2406.1243 2405.1170 2405.0991 7.44 291 - 312 1 R.KDLYANNVMSGGTTMYPGIADR.M + 2 Oxidation (M)
2536.1880 2535.1807 2535.1509 11.8 216 - 238 0 K.LCYVALDFENEMATAASSSSLEK.S
2616.3828 2615.3755 2615.3458 11.4 337 - 359 0 K.YSVWIGGSILASLSTFQQMWITK.Q
3196.7109 3195.7036 3195.6023 31.7 148 - 177 0 R.TTGIVLDSGDGVTHNVPIYEGYALPHAIMR.L
3212.7434 3211.7361 3211.5972 43.3 148 - 177 0 R.TTGIVLDSGDGVTHNVPIYEGYALPHAIMR.L + Oxidation (M)
3491.8062 3490.7989 3490.6360 46.7 255 - 284 1 R.FRCPETLFQPSFIGMESAGIHETTYNSIMK.C
No match to: 945.4556, 976.3532, 1130.4626, 1181.5842, 1198.6243, 1320.5065, 1354.5515, 1475.7252, 1483.6229, 1528.6823, 1541.7109, 1542.6951,
1543.7032, 1547.6823, 1556.7256, 1558.6926, 1634.7444, 1642.6931, 1768.9558, 1772.8760, 1804.9034, 1818.8853, 1832.9414, 1838.8187, 1848.9365,
1864.9501, 1896.9443, 1912.9381, 1918.9548, 1928.9333, 1944.9376, 1964.8632, 1974.9027, 1984.0460, 2088.0190, 2105.0481, 2108.0635, 2327.1223,
2600.3669, 2672.3794, 2688.3799, 2743.3975, 3035.7068, 3036.6846, 3224.7659, 3471.7959, 3487.8362
9. gi|310942658 Mass: 42160 Score: 108 Expect: 1.7e-005 Matches: 18
Chain O, Cryo-Em Structure Of Actin Filament In The Presence Of Phosphate
Observed Mr(expt) Mr(calc) ppm Start End Miss Peptide
1500.6478 1499.6405 1499.7005 -39.96 361 - 373 0 K.QEYDEAGPSIVHR.K
1515.6893 1514.6820 1514.7419 -39.51 86 - 96 0 K.IWHHTFYNELR.V
1623.7876 1622.7803 1622.8338 -32.93 179 - 192 1 R.LDLAGRDLTDYLMK.I
1639.7745 1638.7672 1638.8287 -37.51 179 - 192 1 R.LDLAGRDLTDYLMK.I + Oxidation (M)
1790.8533 1789.8460 1789.8846 -21.57 240 - 255 0 K.SYELPDGQVITIGNER.F
1956.0134 1955.0061 1955.0364 -15.47 97 - 114 0 R.VAPEEHPTLLTEAPLNPK.A
2094.0361 2093.0288 2093.0542 -12.10 240 - 257 1 K.SYELPDGQVITIGNERFR.C
2246.0178 2245.0105 2245.0143 -1.70 293 - 313 0 K.DLYANNVMSGGTTMYPGIADR.M
2262.0215 2261.0142 2261.0093 2.19 293 - 313 0 K.DLYANNVMSGGTTMYPGIADR.M + Oxidation (M)
2278.0410 2277.0337 2277.0042 13.0 293 - 313 0 K.DLYANNVMSGGTTMYPGIADR.M + 2 Oxidation (M)
2374.1279 2373.1206 2373.1093 4.77 292 - 313 1 R.KDLYANNVMSGGTTMYPGIADR.M
2390.1262 2389.1189 2389.1042 6.16 292 - 313 1 R.KDLYANNVMSGGTTMYPGIADR.M + Oxidation (M)
2406.1243 2405.1170 2405.0991 7.44 292 - 313 1 R.KDLYANNVMSGGTTMYPGIADR.M + 2 Oxidation (M)
2536.1880 2535.1807 2535.1509 11.8 217 - 239 0 K.LCYVALDFENEMATAASSSSLEK.S
2616.3828 2615.3755 2615.3458 11.4 338 - 360 0 K.YSVWIGGSILASLSTFQQMWITK.Q
3196.7109 3195.7036 3195.6023 31.7 149 - 178 0 R.TTGIVLDSGDGVTHNVPIYEGYALPHAIMR.L
3212.7434 3211.7361 3211.5972 43.3 149 - 178 0 R.TTGIVLDSGDGVTHNVPIYEGYALPHAIMR.L + Oxidation (M)
3491.8062 3490.7989 3490.6360 46.7 256 - 285 1 R.FRCPETLFQPSFIGMESAGIHETTYNSIMK.C
No match to: 945.4556, 976.3532, 1130.4626, 1181.5842, 1198.6243, 1320.5065, 1354.5515, 1475.7252, 1483.6229, 1528.6823, 1541.7109, 1542.6951,
1543.7032, 1547.6823, 1556.7256, 1558.6926, 1634.7444, 1642.6931, 1768.9558, 1772.8760, 1804.9034, 1818.8853, 1832.9414, 1838.8187, 1848.9365,
1864.9501, 1896.9443, 1912.9381, 1918.9548, 1928.9333, 1944.9376, 1964.8632, 1974.9027, 1984.0460, 2088.0190, 2105.0481, 2108.0635, 2327.1223,
2600.3669, 2672.3794, 2688.3799, 2743.3975, 3035.7068, 3036.6846, 3224.7659, 3471.7959, 3487.8362
10. gi|55669843 Mass: 41486 Score: 107 Expect: 2.1e-005 Matches: 18
Chain A, Structural Basis Of Actin Sequestration By Thymosin-B4: Implications For Arp23 ACTIVATION
Observed Mr(expt) Mr(calc) ppm Start End Miss Peptide
1500.6478 1499.6405 1499.7005 -39.96 355 - 367 0 K.QEYDEAGPSIVHR.K
1515.6893 1514.6820 1514.7419 -39.51 80 - 90 0 K.IWHHTFYNELR.V
1623.7876 1622.7803 1622.8338 -32.93 173 - 186 1 R.LDLAGRDLTDYLMK.I
1639.7745 1638.7672 1638.8287 -37.51 173 - 186 1 R.LDLAGRDLTDYLMK.I + Oxidation (M)
1790.8533 1789.8460 1789.8846 -21.57 234 - 249 0 K.SYELPDGQVITIGNER.F
1956.0134 1955.0061 1955.0364 -15.47 91 - 108 0 R.VAPEEHPTLLTEAPLNPK.A
2094.0361 2093.0288 2093.0542 -12.10 234 - 251 1 K.SYELPDGQVITIGNERFR.C
2246.0178 2245.0105 2245.0143 -1.70 287 - 307 0 K.DLYANNVMSGGTTMYPGIADR.M
2262.0215 2261.0142 2261.0093 2.19 287 - 307 0 K.DLYANNVMSGGTTMYPGIADR.M + Oxidation (M)
2278.0410 2277.0337 2277.0042 13.0 287 - 307 0 K.DLYANNVMSGGTTMYPGIADR.M + 2 Oxidation (M)
2374.1279 2373.1206 2373.1093 4.77 286 - 307 1 R.KDLYANNVMSGGTTMYPGIADR.M
2390.1262 2389.1189 2389.1042 6.16 286 - 307 1 R.KDLYANNVMSGGTTMYPGIADR.M + Oxidation (M)
2406.1243 2405.1170 2405.0991 7.44 286 - 307 1 R.KDLYANNVMSGGTTMYPGIADR.M + 2 Oxidation (M)
2536.1880 2535.1807 2535.1509 11.8 211 - 233 0 K.LCYVALDFENEMATAASSSSLEK.S
2616.3828 2615.3755 2615.3458 11.4 332 - 354 0 K.YSVWIGGSILASLSTFQQMWITK.Q
3196.7109 3195.7036 3195.6023 31.7 143 - 172 0 R.TTGIVLDSGDGVTHNVPIYEGYALPHAIMR.L
3212.7434 3211.7361 3211.5972 43.3 143 - 172 0 R.TTGIVLDSGDGVTHNVPIYEGYALPHAIMR.L + Oxidation (M)
3491.8062 3490.7989 3490.6360 46.7 250 - 279 1 R.FRCPETLFQPSFIGMESAGIHETTYNSIMK.C
No match to: 945.4556, 976.3532, 1130.4626, 1181.5842, 1198.6243, 1320.5065, 1354.5515, 1475.7252, 1483.6229, 1528.6823, 1541.7109, 1542.6951,
1543.7032, 1547.6823, 1556.7256, 1558.6926, 1634.7444, 1642.6931, 1768.9558, 1772.8760, 1804.9034, 1818.8853, 1832.9414, 1838.8187, 1848.9365,
1864.9501, 1896.9443, 1912.9381, 1918.9548, 1928.9333, 1944.9376, 1964.8632, 1974.9027, 1984.0460, 2088.0190, 2105.0481, 2108.0635, 2327.1223,
2600.3669, 2672.3794, 2688.3799, 2743.3975, 3035.7068, 3036.6846, 3224.7659, 3471.7959, 3487.8362
11. gi|21730554 Mass: 42075 Score: 107 Expect: 2.1e-005 Matches: 18
Chain A, Crystal Structure Of Human Vitamin D-Binding Protein In Complex With Skeletal Actin
Observed Mr(expt) Mr(calc) ppm Start End Miss Peptide
1500.6478 1499.6405 1499.7005 -39.96 360 - 372 0 K.QEYDEAGPSIVHR.K
1515.6893 1514.6820 1514.7419 -39.51 85 - 95 0 K.IWHHTFYNELR.V
1623.7876 1622.7803 1622.8338 -32.93 178 - 191 1 R.LDLAGRDLTDYLMK.I
1639.7745 1638.7672 1638.8287 -37.51 178 - 191 1 R.LDLAGRDLTDYLMK.I + Oxidation (M)
1790.8533 1789.8460 1789.8846 -21.57 239 - 254 0 K.SYELPDGQVITIGNER.F
1956.0134 1955.0061 1955.0364 -15.47 96 - 113 0 R.VAPEEHPTLLTEAPLNPK.A
2094.0361 2093.0288 2093.0542 -12.10 239 - 256 1 K.SYELPDGQVITIGNERFR.C
2246.0178 2245.0105 2245.0143 -1.70 292 - 312 0 K.DLYANNVMSGGTTMYPGIADR.M
2262.0215 2261.0142 2261.0093 2.19 292 - 312 0 K.DLYANNVMSGGTTMYPGIADR.M + Oxidation (M)
2278.0410 2277.0337 2277.0042 13.0 292 - 312 0 K.DLYANNVMSGGTTMYPGIADR.M + 2 Oxidation (M)

2374.1279 2373.1206 2373.1093 4.77 291 - 312 1 R.KDLYANNVMSGGTTMYPGIADR.M
2390.1262 2389.1189 2389.1042 6.16 291 - 312 1 R.KDLYANNVMSGGTTMYPGIADR.M + Oxidation (M)
2406.1243 2405.1170 2405.0991 7.44 291 - 312 1 R.KDLYANNVMSGGTTMYPGIADR.M + 2 Oxidation (M)
2536.1880 2535.1807 2535.1509 11.8 216 - 238 0 K.LCYVALDFENEMATAASSSSLEK.S
2616.3828 2615.3755 2615.3458 11.4 337 - 359 0 K.YSVWIGGSILASLSTFQQMWITK.Q
3196.7109 3195.7036 3195.6023 31.7 148 - 177 0 R.TTGIVLDSGDGVTHNVPIYEGYALPHAIMR.L
3212.7434 3211.7361 3211.5972 43.3 148 - 177 0 R.TTGIVLDSGDGVTHNVPIYEGYALPHAIMR.L + Oxidation (M)
3491.8062 3490.7989 3490.6360 46.7 255 - 284 1 R.FRCPETLFQPSFIGMESAGIHETTYNSIMK.C
No match to: 945.4556, 976.3532, 1130.4626, 1181.5842, 1198.6243, 1320.5065, 1354.5515, 1475.7252, 1483.6229, 1528.6823, 1541.7109, 1542.6951,
1543.7032, 1547.6823, 1556.7256, 1558.6926, 1634.7444, 1642.6931, 1768.9558, 1772.8760, 1804.9034, 1818.8853, 1832.9414, 1838.8187, 1848.9365,
1864.9501, 1896.9443, 1912.9381, 1918.9548, 1928.9333, 1944.9376, 1964.8632, 1974.9027, 1984.0460, 2088.0190, 2105.0481, 2108.0635, 2327.1223,
2600.3669, 2672.3794, 2688.3799, 2743.3975, 3035.7068, 3036.6846, 3224.7659, 3471.7959, 3487.8362
12. gi|39654752 Mass: 42049 Score: 107 Expect: 2.1e-005 Matches: 18
Chain A, Structure Of Rabbit Actin In Complex With Kabiramide C
Observed Mr(expt) Mr(calc) ppm Start End Miss Peptide
1500.6478 1499.6405 1499.7005 -39.96 360 - 372 0 K.QEYDEAGPSIVHR.K
1515.6893 1514.6820 1514.7419 -39.51 85 - 95 0 K.IWHHTFYNELR.V
1623.7876 1622.7803 1622.8338 -32.93 178 - 191 1 R.LDLAGRDLTDYLMK.I
1639.7745 1638.7672 1638.8287 -37.51 178 - 191 1 R.LDLAGRDLTDYLMK.I + Oxidation (M)
1790.8533 1789.8460 1789.8846 -21.57 239 - 254 0 K.SYELPDGQVITIGNER.F
1956.0134 1955.0061 1955.0364 -15.47 96 - 113 0 R.VAPEEHPTLLTEAPLNPK.A
2094.0361 2093.0288 2093.0542 -12.10 239 - 256 1 K.SYELPDGQVITIGNERFR.C
2246.0178 2245.0105 2245.0143 -1.70 292 - 312 0 K.DLYANNVMSGGTTMYPGIADR.M
2262.0215 2261.0142 2261.0093 2.19 292 - 312 0 K.DLYANNVMSGGTTMYPGIADR.M + Oxidation (M)
2278.0410 2277.0337 2277.0042 13.0 292 - 312 0 K.DLYANNVMSGGTTMYPGIADR.M + 2 Oxidation (M)
2374.1279 2373.1206 2373.1093 4.77 291 - 312 1 R.KDLYANNVMSGGTTMYPGIADR.M
2390.1262 2389.1189 2389.1042 6.16 291 - 312 1 R.KDLYANNVMSGGTTMYPGIADR.M + Oxidation (M)
2406.1243 2405.1170 2405.0991 7.44 291 - 312 1 R.KDLYANNVMSGGTTMYPGIADR.M + 2 Oxidation (M)
2536.1880 2535.1807 2535.1509 11.8 216 - 238 0 K.LCYVALDFENEMATAASSSSLEK.S
2616.3828 2615.3755 2615.3458 11.4 337 - 359 0 K.YSVWIGGSILASLSTFQQMWITK.Q
3196.7109 3195.7036 3195.6023 31.7 148 - 177 0 R.TTGIVLDSGDGVTHNVPIYEGYALPHAIMR.L
3212.7434 3211.7361 3211.5972 43.3 148 - 177 0 R.TTGIVLDSGDGVTHNVPIYEGYALPHAIMR.L + Oxidation (M)
3491.8062 3490.7989 3490.6360 46.7 255 - 284 1 R.FRCPETLFQPSFIGMESAGIHETTYNSIMK.C
No match to: 945.4556, 976.3532, 1130.4626, 1181.5842, 1198.6243, 1320.5065, 1354.5515, 1475.7252, 1483.6229, 1528.6823, 1541.7109, 1542.6951,
1543.7032, 1547.6823, 1556.7256, 1558.6926, 1634.7444, 1642.6931, 1768.9558, 1772.8760, 1804.9034, 1818.8853, 1832.9414, 1838.8187, 1848.9365,
1864.9501, 1896.9443, 1912.9381, 1918.9548, 1928.9333, 1944.9376, 1964.8632, 1974.9027, 1984.0460, 2088.0190, 2105.0481, 2108.0635, 2327.1223,
2600.3669, 2672.3794, 2688.3799, 2743.3975, 3035.7068, 3036.6846, 3224.7659, 3471.7959, 3487.8362
13. gi|4501881 Mass: 42366 Score: 106 Expect: 2.7e-005 Matches: 18
actin, alpha skeletal muscle [Homo sapiens]
Observed Mr(expt) Mr(calc) ppm Start End Miss Peptide
1500.6478 1499.6405 1499.7005 -39.96 362 - 374 0 K.QEYDEAGPSIVHR.K
1515.6893 1514.6820 1514.7419 -39.51 87 - 97 0 K.IWHHTFYNELR.V
1623.7876 1622.7803 1622.8338 -32.93 180 - 193 1 R.LDLAGRDLTDYLMK.I
1639.7745 1638.7672 1638.8287 -37.51 180 - 193 1 R.LDLAGRDLTDYLMK.I + Oxidation (M)
1790.8533 1789.8460 1789.8846 -21.57 241 - 256 0 K.SYELPDGQVITIGNER.F
1956.0134 1955.0061 1955.0364 -15.47 98 - 115 0 R.VAPEEHPTLLTEAPLNPK.A
2094.0361 2093.0288 2093.0542 -12.10 241 - 258 1 K.SYELPDGQVITIGNERFR.C
2246.0178 2245.0105 2245.0143 -1.70 294 - 314 0 K.DLYANNVMSGGTTMYPGIADR.M
2262.0215 2261.0142 2261.0093 2.19 294 - 314 0 K.DLYANNVMSGGTTMYPGIADR.M + Oxidation (M)
2278.0410 2277.0337 2277.0042 13.0 294 - 314 0 K.DLYANNVMSGGTTMYPGIADR.M + 2 Oxidation (M)
2374.1279 2373.1206 2373.1093 4.77 293 - 314 1 R.KDLYANNVMSGGTTMYPGIADR.M
2390.1262 2389.1189 2389.1042 6.16 293 - 314 1 R.KDLYANNVMSGGTTMYPGIADR.M + Oxidation (M)
2406.1243 2405.1170 2405.0991 7.44 293 - 314 1 R.KDLYANNVMSGGTTMYPGIADR.M + 2 Oxidation (M)
2536.1880 2535.1807 2535.1509 11.8 218 - 240 0 K.LCYVALDFENEMATAASSSSLEK.S
2616.3828 2615.3755 2615.3458 11.4 339 - 361 0 K.YSVWIGGSILASLSTFQQMWITK.Q
3196.7109 3195.7036 3195.6023 31.7 150 - 179 0 R.TTGIVLDSGDGVTHNVPIYEGYALPHAIMR.L
3212.7434 3211.7361 3211.5972 43.3 150 - 179 0 R.TTGIVLDSGDGVTHNVPIYEGYALPHAIMR.L + Oxidation (M)
3491.8062 3490.7989 3490.6360 46.7 257 - 286 1 R.FRCPETLFQPSFIGMESAGIHETTYNSIMK.C
No match to: 945.4556, 976.3532, 1130.4626, 1181.5842, 1198.6243, 1320.5065, 1354.5515, 1475.7252, 1483.6229, 1528.6823, 1541.7109, 1542.6951,
1543.7032, 1547.6823, 1556.7256, 1558.6926, 1634.7444, 1642.6931, 1768.9558, 1772.8760, 1804.9034, 1818.8853, 1832.9414, 1838.8187, 1848.9365,
1864.9501, 1896.9443, 1912.9381, 1918.9548, 1928.9333, 1944.9376, 1964.8632, 1974.9027, 1984.0460, 2088.0190, 2105.0481, 2108.0635, 2327.1223,
2600.3669, 2672.3794, 2688.3799, 2743.3975, 3035.7068, 3036.6846, 3224.7659, 3471.7959, 3487.8362
14. gi|7766848 Mass: 42340 Score: 106 Expect: 2.7e-005 Matches: 18
Chain A, Complex Between Rabbit Muscle Alpha-Actin: Human Gelsolin Domain 1
Observed Mr(expt) Mr(calc) ppm Start End Miss Peptide
1500.6478 1499.6405 1499.7005 -39.96 362 - 374 0 K.QEYDEAGPSIVHR.K
1515.6893 1514.6820 1514.7419 -39.51 87 - 97 0 K.IWHHTFYNELR.V
1623.7876 1622.7803 1622.8338 -32.93 180 - 193 1 R.LDLAGRDLTDYLMK.I
1639.7745 1638.7672 1638.8287 -37.51 180 - 193 1 R.LDLAGRDLTDYLMK.I + Oxidation (M)
1790.8533 1789.8460 1789.8846 -21.57 241 - 256 0 K.SYELPDGQVITIGNER.F
1956.0134 1955.0061 1955.0364 -15.47 98 - 115 0 R.VAPEEHPTLLTEAPLNPK.A
2094.0361 2093.0288 2093.0542 -12.10 241 - 258 1 K.SYELPDGQVITIGNERFR.C
2246.0178 2245.0105 2245.0143 -1.70 294 - 314 0 K.DLYANNVMSGGTTMYPGIADR.M
2262.0215 2261.0142 2261.0093 2.19 294 - 314 0 K.DLYANNVMSGGTTMYPGIADR.M + Oxidation (M)
2278.0410 2277.0337 2277.0042 13.0 294 - 314 0 K.DLYANNVMSGGTTMYPGIADR.M + 2 Oxidation (M)
2374.1279 2373.1206 2373.1093 4.77 293 - 314 1 R.KDLYANNVMSGGTTMYPGIADR.M

2390.1262 2389.1189 2389.1042 6.16 293 - 314 1 R.KDLYANNVMSGGTTMYPGIADR.M + Oxidation (M)
2406.1243 2405.1170 2405.0991 7.44 293 - 314 1 R.KDLYANNVMSGGTTMYPGIADR.M + 2 Oxidation (M)
2536.1880 2535.1807 2535.1509 11.8 218 - 240 0 K.LCYVALDFENEMATAASSSSLEK.S
2616.3828 2615.3755 2615.3458 11.4 339 - 361 0 K.YSVWIGGSILASLSTFQQMWITK.Q
3196.7109 3195.7036 3195.6023 31.7 150 - 179 0 R.TTGIVLDSGDGVTHNVPIYEGYALPHAIMR.L
3212.7434 3211.7361 3211.5972 43.3 150 - 179 0 R.TTGIVLDSGDGVTHNVPIYEGYALPHAIMR.L + Oxidation (M)
3491.8062 3490.7989 3490.6360 46.7 257 - 286 1 R.FRCPETLFQPSFIGMESAGIHETTYNSIMK.C
No match to: 945.4556, 976.3532, 1130.4626, 1181.5842, 1198.6243, 1320.5065, 1354.5515, 1475.7252, 1483.6229, 1528.6823, 1541.7109, 1542.6951,
1543.7032, 1547.6823, 1556.7256, 1558.6926, 1634.7444, 1642.6931, 1768.9558, 1772.8760, 1804.9034, 1818.8853, 1832.9414, 1838.8187, 1848.9365,
1864.9501, 1896.9443, 1912.9381, 1918.9548, 1928.9333, 1944.9376, 1964.8632, 1974.9027, 1984.0460, 2088.0190, 2105.0481, 2108.0635, 2327.1223,
2600.3669, 2672.3794, 2688.3799, 2743.3975, 3035.7068, 3036.6846, 3224.7659, 3471.7959, 3487.8362
15. gi|134024776 Mass: 42338 Score: 106 Expect: 2.7e-005 Matches: 18
Actin, alpha 1, skeletal muscle [Bos taurus]
Observed Mr(expt) Mr(calc) ppm Start End Miss Peptide
1500.6478 1499.6405 1499.7005 -39.96 362 - 374 0 K.QEYDEAGPSIVHR.K
1515.6893 1514.6820 1514.7419 -39.51 87 - 97 0 K.IWHHTFYNELR.V
1623.7876 1622.7803 1622.8338 -32.93 180 - 193 1 R.LDLAGRDLTDYLMK.I
1639.7745 1638.7672 1638.8287 -37.51 180 - 193 1 R.LDLAGRDLTDYLMK.I + Oxidation (M)
1790.8533 1789.8460 1789.8846 -21.57 241 - 256 0 K.SYELPDGQVITIGNER.F
1956.0134 1955.0061 1955.0364 -15.47 98 - 115 0 R.VAPEEHPTLLTEAPLNPK.A
2094.0361 2093.0288 2093.0542 -12.10 241 - 258 1 K.SYELPDGQVITIGNERFR.C
2246.0178 2245.0105 2245.0143 -1.70 294 - 314 0 K.DLYANNVMSGGTTMYPGIADR.M
2262.0215 2261.0142 2261.0093 2.19 294 - 314 0 K.DLYANNVMSGGTTMYPGIADR.M + Oxidation (M)
2278.0410 2277.0337 2277.0042 13.0 294 - 314 0 K.DLYANNVMSGGTTMYPGIADR.M + 2 Oxidation (M)
2374.1279 2373.1206 2373.1093 4.77 293 - 314 1 R.KDLYANNVMSGGTTMYPGIADR.M
2390.1262 2389.1189 2389.1042 6.16 293 - 314 1 R.KDLYANNVMSGGTTMYPGIADR.M + Oxidation (M)
2406.1243 2405.1170 2405.0991 7.44 293 - 314 1 R.KDLYANNVMSGGTTMYPGIADR.M + 2 Oxidation (M)
2536.1880 2535.1807 2535.1509 11.8 218 - 240 0 K.LCYVALDFENEMATAASSSSLEK.S
2616.3828 2615.3755 2615.3458 11.4 339 - 361 0 K.YSVWIGGSILASLSTFQQMWITK.Q
3196.7109 3195.7036 3195.6023 31.7 150 - 179 0 R.TTGIVLDSGDGVTHNVPIYEGYALPHAIMR.L
3212.7434 3211.7361 3211.5972 43.3 150 - 179 0 R.TTGIVLDSGDGVTHNVPIYEGYALPHAIMR.L + Oxidation (M)
3491.8062 3490.7989 3490.6360 46.7 257 - 286 1 R.FRCPETLFQPSFIGMESAGIHETTYNSIMK.C
No match to: 945.4556, 976.3532, 1130.4626, 1181.5842, 1198.6243, 1320.5065, 1354.5515, 1475.7252, 1483.6229, 1528.6823, 1541.7109, 1542.6951,
1543.7032, 1547.6823, 1556.7256, 1558.6926, 1634.7444, 1642.6931, 1768.9558, 1772.8760, 1804.9034, 1818.8853, 1832.9414, 1838.8187, 1848.9365,
1864.9501, 1896.9443, 1912.9381, 1918.9548, 1928.9333, 1944.9376, 1964.8632, 1974.9027, 1984.0460, 2088.0190, 2105.0481, 2108.0635, 2327.1223,
2600.3669, 2672.3794, 2688.3799, 2743.3975, 3035.7068, 3036.6846, 3224.7659, 3471.7959, 3487.8362
16. gi|291413356 Mass: 42420 Score: 106 Expect: 2.7e-005 Matches: 18
PREDICTED: cytoplasmic beta-actin [Oryctolagus cuniculus]
Observed Mr(expt) Mr(calc) ppm Start End Miss Peptide
1500.6478 1499.6405 1499.7005 -39.96 362 - 374 0 K.QEYDEAGPSIVHR.K
1515.6893 1514.6820 1514.7419 -39.51 87 - 97 0 K.IWHHTFYNELR.V
1623.7876 1622.7803 1622.8338 -32.93 180 - 193 1 R.LDLAGRDLTDYLMK.I
1639.7745 1638.7672 1638.8287 -37.51 180 - 193 1 R.LDLAGRDLTDYLMK.I + Oxidation (M)
1790.8533 1789.8460 1789.8846 -21.57 241 - 256 0 K.SYELPDGQVITIGNER.F
1956.0134 1955.0061 1955.0364 -15.47 98 - 115 0 R.VAPEEHPTLLTEAPLNPK.A
2094.0361 2093.0288 2093.0542 -12.10 241 - 258 1 K.SYELPDGQVITIGNERFR.C
2246.0178 2245.0105 2245.0143 -1.70 294 - 314 0 K.DLYANNVMSGGTTMYPGIADR.M
2262.0215 2261.0142 2261.0093 2.19 294 - 314 0 K.DLYANNVMSGGTTMYPGIADR.M + Oxidation (M)
2278.0410 2277.0337 2277.0042 13.0 294 - 314 0 K.DLYANNVMSGGTTMYPGIADR.M + 2 Oxidation (M)
2374.1279 2373.1206 2373.1093 4.77 293 - 314 1 R.KDLYANNVMSGGTTMYPGIADR.M
2390.1262 2389.1189 2389.1042 6.16 293 - 314 1 R.KDLYANNVMSGGTTMYPGIADR.M + Oxidation (M)
2406.1243 2405.1170 2405.0991 7.44 293 - 314 1 R.KDLYANNVMSGGTTMYPGIADR.M + 2 Oxidation (M)
2536.1880 2535.1807 2535.1509 11.8 218 - 240 0 K.LCYVALDFENEMATAASSSSLEK.S
2616.3828 2615.3755 2615.3458 11.4 339 - 361 0 K.YSVWIGGSILASLSTFQQMWITK.Q
3196.7109 3195.7036 3195.6023 31.7 150 - 179 0 R.TTGIVLDSGDGVTHNVPIYEGYALPHAIMR.L
3212.7434 3211.7361 3211.5972 43.3 150 - 179 0 R.TTGIVLDSGDGVTHNVPIYEGYALPHAIMR.L + Oxidation (M)
3491.8062 3490.7989 3490.6360 46.7 257 - 286 1 R.FRCPETLFQPSFIGMESAGIHETTYNSIMK.C
No match to: 945.4556, 976.3532, 1130.4626, 1181.5842, 1198.6243, 1320.5065, 1354.5515, 1475.7252, 1483.6229, 1528.6823, 1541.7109, 1542.6951,
1543.7032, 1547.6823, 1556.7256, 1558.6926, 1634.7444, 1642.6931, 1768.9558, 1772.8760, 1804.9034, 1818.8853, 1832.9414, 1838.8187, 1848.9365,
1864.9501, 1896.9443, 1912.9381, 1918.9548, 1928.9333, 1944.9376, 1964.8632, 1974.9027, 1984.0460, 2088.0190, 2105.0481, 2108.0635, 2327.1223,
2600.3669, 2672.3794, 2688.3799, 2743.3975, 3035.7068, 3036.6846, 3224.7659, 3471.7959, 3487.8362
17. gi|332812177 Mass: 42396 Score: 106 Expect: 2.7e-005 Matches: 18
PREDICTED: actin, alpha skeletal muscle [Pan troglodytes]
Observed Mr(expt) Mr(calc) ppm Start End Miss Peptide
1500.6478 1499.6405 1499.7005 -39.96 362 - 374 0 K.QEYDEAGPSIVHR.K
1515.6893 1514.6820 1514.7419 -39.51 87 - 97 0 K.IWHHTFYNELR.V
1623.7876 1622.7803 1622.8338 -32.93 180 - 193 1 R.LDLAGRDLTDYLMK.I
1639.7745 1638.7672 1638.8287 -37.51 180 - 193 1 R.LDLAGRDLTDYLMK.I + Oxidation (M)
1790.8533 1789.8460 1789.8846 -21.57 241 - 256 0 K.SYELPDGQVITIGNER.F
1956.0134 1955.0061 1955.0364 -15.47 98 - 115 0 R.VAPEEHPTLLTEAPLNPK.A
2094.0361 2093.0288 2093.0542 -12.10 241 - 258 1 K.SYELPDGQVITIGNERFR.C
2246.0178 2245.0105 2245.0143 -1.70 294 - 314 0 K.DLYANNVMSGGTTMYPGIADR.M
2262.0215 2261.0142 2261.0093 2.19 294 - 314 0 K.DLYANNVMSGGTTMYPGIADR.M + Oxidation (M)
2278.0410 2277.0337 2277.0042 13.0 294 - 314 0 K.DLYANNVMSGGTTMYPGIADR.M + 2 Oxidation (M)
2374.1279 2373.1206 2373.1093 4.77 293 - 314 1 R.KDLYANNVMSGGTTMYPGIADR.M
2390.1262 2389.1189 2389.1042 6.16 293 - 314 1 R.KDLYANNVMSGGTTMYPGIADR.M + Oxidation (M)

2406.1243 2405.1170 2405.0991 7.44 293 - 314 1 R.KDLYANNVMSGGTTMYPGIADR.M + 2 Oxidation (M)
2536.1880 2535.1807 2535.1509 11.8 218 - 240 0 K.LCYVALDFENEMATAASSSSLEK.S
2616.3828 2615.3755 2615.3458 11.4 339 - 361 0 K.YSVWIGGSILASLSTFQQMWITK.Q
3196.7109 3195.7036 3195.6023 31.7 150 - 179 0 R.TTGIVLDSGDGVTHNVPIYEGYALPHAIMR.L
3212.7434 3211.7361 3211.5972 43.3 150 - 179 0 R.TTGIVLDSGDGVTHNVPIYEGYALPHAIMR.L + Oxidation (M)
3491.8062 3490.7989 3490.6360 46.7 257 - 286 1 R.FRCPETLFQPSFIGMESAGIHETTYNSIMK.C
No match to: 945.4556, 976.3532, 1130.4626, 1181.5842, 1198.6243, 1320.5065, 1354.5515, 1475.7252, 1483.6229, 1528.6823, 1541.7109, 1542.6951,
1543.7032, 1547.6823, 1556.7256, 1558.6926, 1634.7444, 1642.6931, 1768.9558, 1772.8760, 1804.9034, 1818.8853, 1832.9414, 1838.8187, 1848.9365,
1864.9501, 1896.9443, 1912.9381, 1918.9548, 1928.9333, 1944.9376, 1964.8632, 1974.9027, 1984.0460, 2088.0190, 2105.0481, 2108.0635, 2327.1223,
2600.3669, 2672.3794, 2688.3799, 2743.3975, 3035.7068, 3036.6846, 3224.7659, 3471.7959, 3487.8362
18. gi|296230233 Mass: 37576 Score: 100 Expect: 0.00012 Matches: 17
PREDICTED: actin, alpha skeletal muscle isoform 4 [Callithrix jacchus]
Observed Mr(expt) Mr(calc) ppm Start End Miss Peptide
1500.6478 1499.6405 1499.7005 -39.96 319 - 331 0 K.QEYDEAGPSIVHR.K
1623.7876 1622.7803 1622.8338 -32.93 137 - 150 1 R.LDLAGRDLTDYLMK.I
1639.7745 1638.7672 1638.8287 -37.51 137 - 150 1 R.LDLAGRDLTDYLMK.I + Oxidation (M)
1790.8533 1789.8460 1789.8846 -21.57 198 - 213 0 K.SYELPDGQVITIGNER.F
1956.0134 1955.0061 1955.0364 -15.47 55 - 72 0 R.VAPEEHPTLLTEAPLNPK.A
2094.0361 2093.0288 2093.0542 -12.10 198 - 215 1 K.SYELPDGQVITIGNERFR.C
2246.0178 2245.0105 2245.0143 -1.70 251 - 271 0 K.DLYANNVMSGGTTMYPGIADR.M
2262.0215 2261.0142 2261.0093 2.19 251 - 271 0 K.DLYANNVMSGGTTMYPGIADR.M + Oxidation (M)
2278.0410 2277.0337 2277.0042 13.0 251 - 271 0 K.DLYANNVMSGGTTMYPGIADR.M + 2 Oxidation (M)
2374.1279 2373.1206 2373.1093 4.77 250 - 271 1 R.KDLYANNVMSGGTTMYPGIADR.M
2390.1262 2389.1189 2389.1042 6.16 250 - 271 1 R.KDLYANNVMSGGTTMYPGIADR.M + Oxidation (M)
2406.1243 2405.1170 2405.0991 7.44 250 - 271 1 R.KDLYANNVMSGGTTMYPGIADR.M + 2 Oxidation (M)
2536.1880 2535.1807 2535.1509 11.8 175 - 197 0 K.LCYVALDFENEMATAASSSSLEK.S
2616.3828 2615.3755 2615.3458 11.4 296 - 318 0 K.YSVWIGGSILASLSTFQQMWITK.Q
3196.7109 3195.7036 3195.6023 31.7 107 - 136 0 R.TTGIVLDSGDGVTHNVPIYEGYALPHAIMR.L
3212.7434 3211.7361 3211.5972 43.3 107 - 136 0 R.TTGIVLDSGDGVTHNVPIYEGYALPHAIMR.L + Oxidation (M)
3491.8062 3490.7989 3490.6360 46.7 214 - 243 1 R.FRCPETLFQPSFIGMESAGIHETTYNSIMK.C
No match to: 945.4556, 976.3532, 1130.4626, 1181.5842, 1198.6243, 1320.5065, 1354.5515, 1475.7252, 1483.6229, 1515.6893, 1528.6823, 1541.7109,
1542.6951, 1543.7032, 1547.6823, 1556.7256, 1558.6926, 1634.7444, 1642.6931, 1768.9558, 1772.8760, 1804.9034, 1818.8853, 1832.9414, 1838.8187,
1848.9365, 1864.9501, 1896.9443, 1912.9381, 1918.9548, 1928.9333, 1944.9376, 1964.8632, 1974.9027, 1984.0460, 2088.0190, 2105.0481, 2108.0635,
2327.1223, 2600.3669, 2672.3794, 2688.3799, 2743.3975, 3035.7068, 3036.6846, 3224.7659, 3471.7959, 3487.8362
19. gi|306440544 Mass: 41484 Score: 96 Expect: 0.00027 Matches: 17
Chain A, Crystal Structure Of Actin In Complex With Lobophorolide
Observed Mr(expt) Mr(calc) ppm Start End Miss Peptide
1515.6893 1514.6820 1514.7419 -39.51 85 - 95 0 K.IWHHTFYNELR.V
1623.7876 1622.7803 1622.8338 -32.93 178 - 191 1 R.LDLAGRDLTDYLMK.I
1639.7745 1638.7672 1638.8287 -37.51 178 - 191 1 R.LDLAGRDLTDYLMK.I + Oxidation (M)
1790.8533 1789.8460 1789.8846 -21.57 239 - 254 0 K.SYELPDGQVITIGNER.F
1956.0134 1955.0061 1955.0364 -15.47 96 - 113 0 R.VAPEEHPTLLTEAPLNPK.A
2094.0361 2093.0288 2093.0542 -12.10 239 - 256 1 K.SYELPDGQVITIGNERFR.C
2246.0178 2245.0105 2245.0143 -1.70 292 - 312 0 K.DLYANNVMSGGTTMYPGIADR.M
2262.0215 2261.0142 2261.0093 2.19 292 - 312 0 K.DLYANNVMSGGTTMYPGIADR.M + Oxidation (M)
2278.0410 2277.0337 2277.0042 13.0 292 - 312 0 K.DLYANNVMSGGTTMYPGIADR.M + 2 Oxidation (M)
2374.1279 2373.1206 2373.1093 4.77 291 - 312 1 R.KDLYANNVMSGGTTMYPGIADR.M
2390.1262 2389.1189 2389.1042 6.16 291 - 312 1 R.KDLYANNVMSGGTTMYPGIADR.M + Oxidation (M)
2406.1243 2405.1170 2405.0991 7.44 291 - 312 1 R.KDLYANNVMSGGTTMYPGIADR.M + 2 Oxidation (M)
2536.1880 2535.1807 2535.1509 11.8 216 - 238 0 K.LCYVALDFENEMATAASSSSLEK.S
2616.3828 2615.3755 2615.3458 11.4 337 - 359 0 K.YSVWIGGSILASLSTFQQMWITK.Q
3196.7109 3195.7036 3195.6023 31.7 148 - 177 0 R.TTGIVLDSGDGVTHNVPIYEGYALPHAIMR.L
3212.7434 3211.7361 3211.5972 43.3 148 - 177 0 R.TTGIVLDSGDGVTHNVPIYEGYALPHAIMR.L + Oxidation (M)
3491.8062 3490.7989 3490.6360 46.7 255 - 284 1 R.FRCPETLFQPSFIGMESAGIHETTYNSIMK.C
No match to: 945.4556, 976.3532, 1130.4626, 1181.5842, 1198.6243, 1320.5065, 1354.5515, 1475.7252, 1483.6229, 1500.6478, 1528.6823, 1541.7109,
1542.6951, 1543.7032, 1547.6823, 1556.7256, 1558.6926, 1634.7444, 1642.6931, 1768.9558, 1772.8760, 1804.9034, 1818.8853, 1832.9414, 1838.8187,
1848.9365, 1864.9501, 1896.9443, 1912.9381, 1918.9548, 1928.9333, 1944.9376, 1964.8632, 1974.9027, 1984.0460, 2088.0190, 2105.0481, 2108.0635,
2327.1223, 2600.3669, 2672.3794, 2688.3799, 2743.3975, 3035.7068, 3036.6846, 3224.7659, 3471.7959, 3487.8362
20. gi|296230231 Mass: 38909 Score: 93 Expect: 0.00056 Matches: 16
PREDICTED: actin, alpha skeletal muscle isoform 3 [Callithrix jacchus]
Observed Mr(expt) Mr(calc) ppm Start End Miss Peptide
1500.6478 1499.6405 1499.7005 -39.96 328 - 340 0 K.QEYDEAGPSIVHR.K
1515.6893 1514.6820 1514.7419 -39.51 87 - 97 0 K.IWHHTFYNELR.V
1623.7876 1622.7803 1622.8338 -32.93 146 - 159 1 R.LDLAGRDLTDYLMK.I
1639.7745 1638.7672 1638.8287 -37.51 146 - 159 1 R.LDLAGRDLTDYLMK.I + Oxidation (M)
1790.8533 1789.8460 1789.8846 -21.57 207 - 222 0 K.SYELPDGQVITIGNER.F
1956.0134 1955.0061 1955.0364 -15.47 98 - 115 0 R.VAPEEHPTLLTEAPLNPK.A
2094.0361 2093.0288 2093.0542 -12.10 207 - 224 1 K.SYELPDGQVITIGNERFR.C
2246.0178 2245.0105 2245.0143 -1.70 260 - 280 0 K.DLYANNVMSGGTTMYPGIADR.M
2262.0215 2261.0142 2261.0093 2.19 260 - 280 0 K.DLYANNVMSGGTTMYPGIADR.M + Oxidation (M)
2278.0410 2277.0337 2277.0042 13.0 260 - 280 0 K.DLYANNVMSGGTTMYPGIADR.M + 2 Oxidation (M)
2374.1279 2373.1206 2373.1093 4.77 259 - 280 1 R.KDLYANNVMSGGTTMYPGIADR.M
2390.1262 2389.1189 2389.1042 6.16 259 - 280 1 R.KDLYANNVMSGGTTMYPGIADR.M + Oxidation (M)
2406.1243 2405.1170 2405.0991 7.44 259 - 280 1 R.KDLYANNVMSGGTTMYPGIADR.M + 2 Oxidation (M)
2536.1880 2535.1807 2535.1509 11.8 184 - 206 0 K.LCYVALDFENEMATAASSSSLEK.S
2616.3828 2615.3755 2615.3458 11.4 305 - 327 0 K.YSVWIGGSILASLSTFQQMWITK.Q

3491.8062 3490.7989 3490.6360 46.7 223 - 252 1 R.FRCPETLFQPSFIGMESAGIHETTYNSIMK.C
No match to: 945.4556, 976.3532, 1130.4626, 1181.5842, 1198.6243, 1320.5065, 1354.5515, 1475.7252, 1483.6229, 1528.6823, 1541.7109, 1542.6951,
1543.7032, 1547.6823, 1556.7256, 1558.6926, 1634.7444, 1642.6931, 1768.9558, 1772.8760, 1804.9034, 1818.8853, 1832.9414, 1838.8187, 1848.9365,
1864.9501, 1896.9443, 1912.9381, 1918.9548, 1928.9333, 1944.9376, 1964.8632, 1974.9027, 1984.0460, 2088.0190, 2105.0481, 2108.0635, 2327.1223,
2600.3669, 2672.3794, 2688.3799, 2743.3975, 3035.7068, 3036.6846, 3196.7109, 3212.7434, 3224.7659, 3471.7959, 3487.8362

Search Parameters

Type of search : Peptide Mass Fingerprint
Enzyme : Trypsin
Fixed modifications : Carbamidomethyl (C)
Variable modifications : Oxidation (M)
Mass values : Monoisotopic
Protein Mass : Unrestricted
Peptide Mass Tolerance : ± 50 ppm
Peptide Charge State : 1+
Max Missed Cleavages : 1
Number of queries : 65

Mascot: http://www.matrixscience.com/

COVERAGE BAND 8-1

Mascot Search Results

Protein View

Match to: gi|297343122 Score: 111 Expect: 8.4e-006
Chain A, Structures Of Actin-Bound Wh2 Domains Of Spire And The Implication For Filament Nucleation
Nominal mass (Mr): 40304; Calculated pI value: 5.10
NCBI BLAST search of gi|297343122 against nr
Unformatted sequence string for pasting into other applications
Taxonomy: Oryctolagus cuniculus
Fixed modifications: Carbamidomethyl (C)
Variable modifications: Oxidation (M)
Cleavage by Trypsin: cuts C-term side of KR unless next residue is P
Number of mass values searched: 65
Number of mass values matched: 18
Sequence Coverage: 55%
Matched peptides shown in **Bold Red**
1 DEDETTALVC DNGSGLVKAG FAGDDAPRAV FPSIVGRVGD EAQSKRGILT
51 LKYPIEHGII TNWDDMEK**IW HHTFYNELRV APEEHPTLLT EAPLNPK**ANR
101 EKMTQIMFET FNVPAMYVAI QAVLSLYASG R**TTGIVLDSG DGVTHNVPIY**
151 **EGYALPHAIM RLDLAGRDLT DYLMK**ILTER GYSFVTTAER EIVRDIKEK**L**
201 **CYVALDFENE MATAASSSSL EKSYELPDGQ VITIGNERFR CPETLFQPSF**
251 **IGMESAGIHE TTYNSIMK**CD IDIR**KDLYAN NVMSGGTTMY PGIADR**MQKE
301 ITALAPSTMK IKIIAPPERK **YSVWIGGSIL ASLSTFQQMW ITKQEYDEAG**
351 **PSIVHR**KCF
Show predicted peptides also
Sort Peptides By Residue Number Increasing Mass Decreasing Mass
Start - End Observed Mr(expt) Mr(calc) ppm Miss Sequence
69 - 79 1515.6893 1514.6820 1514.7419 -40 0 K.IWHHTFYNELR.V
80 - 97 1956.0134 1955.0061 1955.0364 -15 0 R.VAPEEHPTLLTEAPLNPK.A
132 - 161 3196.7109 3195.7036 3195.6023 32 0 R.TTGIVLDSGDGVTHNVPIYEGYALPHAIMR.L
132 - 161 3212.7434 3211.7361 3211.5972 43 0 R.TTGIVLDSGDGVTHNVPIYEGYALPHAIMR.L Oxidation (M)
162 - 175 1623.7876 1622.7803 1622.8338 -33 1 R.LDLAGRDLTDYLMK.I
162 - 175 1639.7745 1638.7672 1638.8287 -38 1 R.LDLAGRDLTDYLMK.I Oxidation (M)
200 - 222 2536.1880 2535.1807 2535.1509 12 0 K.LCYVALDFENEMATAASSSSLEK.S
223 - 238 1790.8533 1789.8460 1789.8846 -22 0 K.SYELPDGQVITIGNER.F
223 - 240 2094.0361 2093.0288 2093.0542 -12 1 K.SYELPDGQVITIGNERFR.C
239 - 268 3491.8062 3490.7989 3490.6360 47 1 R.FRCPETLFQPSFIGMESAGIHETTYNSIMK.C
275 - 296 2374.1279 2373.1206 2373.1093 5 1 R.KDLYANNVMSGGTTMYPGIADR.M
275 - 296 2390.1262 2389.1189 2389.1042 6 1 R.KDLYANNVMSGGTTMYPGIADR.M Oxidation (M)
275 - 296 2406.1243 2405.1170 2405.0991 7 1 R.KDLYANNVMSGGTTMYPGIADR.M 2 Oxidation (M)
276 - 296 2246.0178 2245.0105 2245.0143 -2 0 K.DLYANNVMSGGTTMYPGIADR.M
276 - 296 2262.0215 2261.0142 2261.0093 2 0 K.DLYANNVMSGGTTMYPGIADR.M Oxidation (M)
276 - 296 2278.0410 2277.0337 2277.0042 13 0 K.DLYANNVMSGGTTMYPGIADR.M 2 Oxidation (M)
321 - 343 2616.3828 2615.3755 2615.3458 11 0 K.YSVWIGGSILASLSTFQQMWITK.Q
344 - 356 1500.6478 1499.6405 1499.7005 -40 0 K.QEYDEAGPSIVHR.K
No match to: 945.4556, 976.3532, 1130.4626, 1181.5842, 1198.6243, 1320.5065, 1354.5515, 1475.7252, 1483.6229, 1528.6823, 1541.71

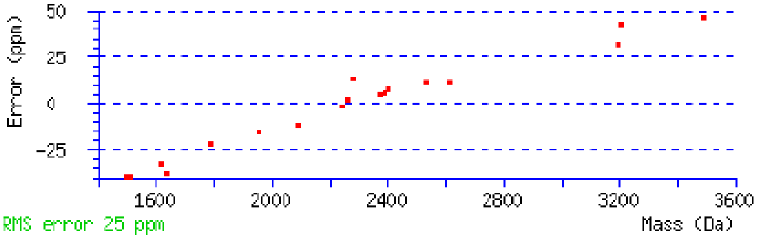

COVERAGE BAND 8-2

Mascot Search Results

Protein View

Match to: gi|77736349 Score: 80 Expect: 0.011
beta-enolase [Bos taurus]
Nominal mass (Mr): 47409; Calculated pI value: 7.60
NCBI BLAST search of gi|77736349 against nr
Unformatted sequence string for pasting into other applications
Taxonomy: Bos taurus
Links to retrieve other entries containing this sequence from NCBI Entrez:
gi|122140864 from Bos taurus
gi|73587037 from Bos taurus
gi|296476763 from Bos taurus
Fixed modifications: Carbamidomethyl (C)
Variable modifications: Oxidation (M)
Cleavage by Trypsin: cuts C-term side of KR unless next residue is P
Number of mass values searched: 65
Number of mass values matched: 13
Sequence Coverage: 38%
Matched peptides shown in **Bold Red**
1 MAMQKIFARE ILDSRGNPTV EVDLHTAKGR FRAAVPSGAS TGIYEALELR
51 DGDKSRYLGK GVLKAVEHIN KTLGPALLEK KLSVVDQEKV DKFMIELDGT
101 ENKSKFGANA ILGVSLAVCK AGAAEKGVPL YRHIADLAGN PELILPVPAF
151 NVINGGSHAG NKLAMQEFMI LPVGASSFRE AMRIGAEVYH HLKGVIKAKY
201 GKDATNVGDE GGFAPNILEN NEALELLKTA IQAAGYPDKV VIGMDVAASE
251 FYRNGKYDLD FKSPDDPARH ISGEKLGELY KNFIKNYPVV SIEDPFDQDD
301 WATWTSFLSG VNIQIVGDDL TVTNPKRIAQ AVEKKACNCL LLKVNQIGSV
351 TESIQACKLA QSNGWGVMVS HRSGETEDTF IADLVVGLCT GQIKTGAPCR
401 SERLAKYNQL MRIEEALGDK AVFAGRKFRN PKAK
Show predicted peptides also
Sort Peptides By Residue Number Increasing Mass Decreasing Mass
Start - End Observed Mr(expt) Mr(calc) ppm Miss Sequence
10 - 28 2094.0361 2093.0288 2093.0753 -22 1 R.EILDSRGNPTVEVDLHTAK.G
31 - 50 2108.0635 2107.0562 2107.1062 -24 1 R.FRAAVPSGASTGIYEALELR.D
33 - 50 1804.9034 1803.8961 1803.9366 -22 0 R.AAVPSGASTGIYEALELR.D
133 - 162 3035.7068 3034.6995 3034.5988 33 0 R.HIADLAGNPELILPVPAFNVINGGSHAGNK.L
163 - 179 1896.9443 1895.9370 1895.9637 -14 0 K.LAMQEFMILPVGASSFR.E
163 - 179 1912.9381 1911.9308 1911.9587 -15 0 K.LAMQEFMILPVGASSFR.E Oxidation (M)
163 - 179 1928.9333 1927.9260 1927.9536 -14 0 K.LAMQEFMILPVGASSFR.E 2 Oxidation (M)
203 - 228 2743.3975 2742.3902 2742.3348 20 0 K.DATNVGDEGGFAPNILENNEALELLK.T
229 - 253 2672.3794 2671.3721 2671.3316 15 1 K.TAIQAAGYPDKVVIGMDVAASEFYR.N
229 - 253 2688.3799 2687.3726 2687.3265 17 1 K.TAIQAAGYPDKVVIGMDVAASEFYR.N Oxidation (M)
240 - 253 1556.7256 1555.7183 1555.7705 -34 0 K.VVIGMDVAASEFYR.N
359 - 372 1541.7109 1540.7036 1540.7569 -35 0 K.LAQSNGWGVMVSHR.S
413 - 426 1475.7252 1474.7179 1474.7780 -41 1 R.IEEALGDKAVFAGR.K
No match to: 945.4556, 976.3532, 1130.4626, 1181.5842, 1198.6243, 1320.5065, 1354.5515, 1483.6229, 1500.6478, 1515.6893, 1528.68

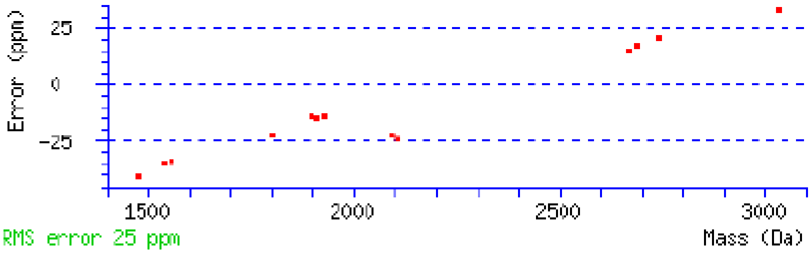

BAND 9

Mascot Search Results

Mascot Search Results

User :
Email :
Search title : SampleSetID: 824, AnalysisID: 7242, MaldiWellID: 69612, SpectrumID: 154537, Path=\180719\MS\18-106 NCBI Mammalia
Database : NCBInr 20120508 (17919084 sequences; 6150218869 residues)
Taxonomy : Mammalia (mammals) (1061927 sequences)
Timestamp : 19 Jul 2018 at 12:14:59 GMT
Top Score : 232 for Mixture 1, gi|4838363 + gi|20664362

Mascot Score Histogram

Protein score is -10*Log(P), where P is the probability that the observed match is a random event.
Protein scores greater than 73 are significant (p<0.05).

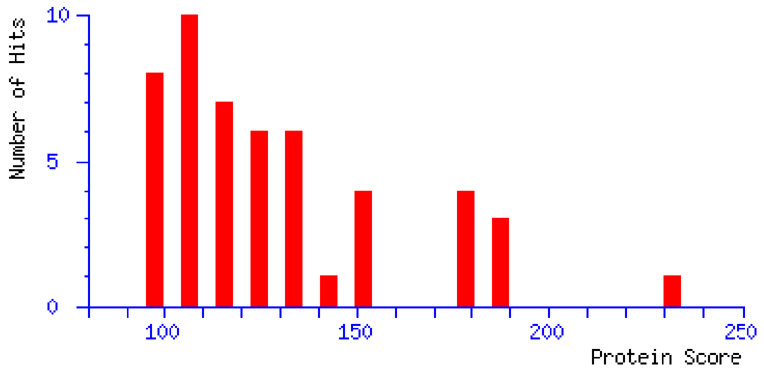

Protein Summary Report

Format As Protein Summary Help
Significance threshold p< 0.05 Max. number of hits 20
Re-Search All Search Unmatched

Index

Accession Mass Score Description
1. Mixture 1 232 gi|4838363 + gi|20664362
2. Mixture 2 185 gi|21536288 + gi|20664362
3. Mixture 3 185 gi|351706982 + gi|20664362
4. Mixture 4 184 gi|301777736 + gi|20664362
5. Mixture 5 181 gi|149722203 + gi|20664362
6. gi|4838363 43172 179 creatine kinase M chain [Bos taurus]
7. gi|194018722 43260 178 creatine kinase M-type [Sus scrofa]
8. gi|60097925 43190 177 creatine kinase M-type [Bos taurus]
9. gi|126344225 43205 152 PREDICTED: creatine kinase M-type-like [Monodelphis domestica]
10. gi|21536288 43302 150 creatine kinase M-type [Homo sapiens]
11. gi|355703653 43302 150 hypothetical protein EGK_10748 [Macaca mulatta]
12. gi|149722203 43370 148 PREDICTED: creatine kinase M-type-like [Equus caballus]
13. gi|301777736 43353 144 PREDICTED: creatine kinase M-type-like [Ailuropoda melanoleuca]
14. gi|297705152 43287 138 PREDICTED: creatine kinase M-type-like isoform 2 [Pongo abelii]
15. gi|189053833 43230 135 unnamed protein product [Homo sapiens]
16. Mixture 6 134 gi|20664362 + gi|344269321
17. gi|119577741 45971 134 creatine kinase, muscle [Homo sapiens]
18. gi|351706982 43449 132 Creatine kinase M-type [Heterocephalus glaber]
19. gi|6671762 43246 130 creatine kinase M-type [Mus musculus]
20. gi|6978661 43220 129 creatine kinase M-type [Rattus norvegicus]

Results List

1. Mixture 1 Total score: 232 Expect: 6.7e-018 Matches: 34
Components: 1. gi|4838363 creatine kinase M chain [Bos taurus]
2. gi|20664362 Chain A, Polylysine Induces An Antiparallel Actin Dimer That Nucleates Filament Assembly: Crystal Structure At 3.5 A Resolution
Observed Mr(expt) Mr(calc) ppm Start End Miss Comp Peptide
907.4688 906.4615 906.4811 -21.56 308 - 314 0 1 K.FEEILTR.L
914.4267 913.4194 913.4406 -23.20 2 - 9 0 1 M.PFGNTHNK.H
976.4352 975.4279 975.4410 -13.42 15 - 24 0 2 K.AGFAGDDAPR.A
1007.5299 1006.5226 1006.5191 3.51 359 - 366 1 1 K.LMVEMEKK.L
1130.5361 1129.5288 1129.5404 -10.24 193 - 202 0 2 R.GYSFVTTAER.E
1157.5375 1156.5302 1156.5448 -12.56 139 - 148 0 1 K.GYALPPHCSR.G
1198.6973 1197.6900 1197.6982 -6.85 25 - 35 0 2 R.AVFPSIVGRPR.H
1231.6245 1230.6172 1230.6245 -5.87 87 - 96 0 1 K.DLFDPIIQDR.H
1269.6873 1268.6800 1268.6877 -6.05 305 - 314 1 1 K.HPKFEEILTR.L
1500.7061 1499.6988 1499.7005 -1.09 356 - 368 0 2 K.QEYDEAGPSIVHR.K
1507.7008 1506.6935 1506.6951 -1.01 117 - 130 0 1 K.GGDDLDPNYVLSSR.V
1515.7449 1514.7376 1514.7419 -2.80 81 - 91 0 2 K.IWHHTFYNELR.V
1610.8282 1609.8209 1609.8538 -20.42 253 - 265 1 1 R.FCVGLQKIEEIFK.K

1643.8143 1642.8070 1642.8103 -2.02 224 - 236 0 1 K.SFLVWVNEEDHLR.V
1682.8115 1681.8042 1681.8199 -9.30 12 - 25 1 1 K.LNFKAEEEYPDLSK.H
1692.9031 1691.8958 1691.9094 -8.01 157 - 172 1 1 K.LSVEALNSLTGEFKGK.Y
1785.9584 1784.9511 1784.9520 -0.50 342 - 358 0 1 R.LGSSEVEQVQLVVDGVK.L
1790.8910 1789.8837 1789.8846 -0.51 235 - 250 0 2 K.SYELPDGQVITIGNER.F
1956.0355 1955.0282 1955.0364 -4.16 92 - 109 0 2 R.VAPEEHPTLLTEAPLNPK.A
1976.9459 1975.9386 1975.8986 20.3 65 - 80 0 2 K.YPIEHGIITNWDDMEK.I + Oxidation (M)
1994.9438 1993.9365 1993.9342 1.19 321 - 341 0 1 R.GTGGVDTAAVGSVFDVSNADR.L
2374.2097 2373.2024 2373.1093 39.2 287 - 308 1 2 R.KDLYANNVMSGGTTMYPGIADR.M
2390.1384 2389.1311 2389.1042 11.3 287 - 308 1 2 R.KDLYANNVMSGGTTMYPGIADR.M + Oxidation (M)
2608.2764 2607.2691 2607.2619 2.79 216 - 236 1 1 R.GIWHNDNKSFLVWVNEEDHLR.V
2616.3115 2615.3042 2615.3458 -15.89 333 - 355 0 2 K.YSVWIGGSILASLSTFQQMWITK.Q
2633.3447 2632.3374 2632.2084 49.0 288 - 311 1 2 K.DLYANNVMSGGTTMYPGIADRMQK.E
2927.4255 2926.4182 2926.4007 5.99 267 - 292 0 1 K.AGHPFMWNEHLGYVLTCPSNLGTGLR.G
2943.4182 2942.4109 2942.3956 5.20 267 - 292 0 1 K.AGHPFMWNEHLGYVLTCPSNLGTGLR.G + Oxidation (M)
3071.5420 3070.5347 3070.4906 14.4 266 - 292 1 1 K.KAGHPFMWNEHLGYVLTCPSNLGTGLR.G + Oxidation (M)
3196.6343 3195.6270 3195.6023 7.74 144 - 173 0 2 R.TTGIVLDSGDGVTHNVPIYEGYALPHAIMR.L
3212.6292 3211.6219 3211.5972 7.69 144 - 173 0 2 R.TTGIVLDSGDGVTHNVPIYEGYALPHAIMR.L + Oxidation (M)
3644.8499 3643.8426 3643.8014 11.3 178 - 209 0 1 K.SMTEQEQQQLIDDHFLFDKPVSPLLLASGMAR.D
3660.8306 3659.8233 3659.7964 7.37 178 - 209 0 1 K.SMTEQEQQQLIDDHFLFDKPVSPLLLASGMAR.D + Oxidation (M)
3761.8979 3760.8906 3760.8756 3.99 321 - 358 1 1 R.GTGGVDTAAVGSVFDVSNADRLGSSEVEQVQLVVDGVK.L
No match to: 854.2844, 855.0399, 886.2189, 886.9801, 935.4965, 1254.5332, 1359.7104, 1483.6857, 1511.7391, 1671.8419, 1675.8209, 1738.9460, 1789.8499,
1799.8850, 1813.9745, 1818.9233, 1984.0746, 2055.1343, 2571.3091, 2573.2319, 2601.3367, 2612.2659, 2879.5073, 2942.4229, 2955.4292, 2959.4331,
2971.4209, 3136.4556, 3153.4805, 3673.8777, 3789.9407
2. Mixture 2 Total score: 185 Expect: 3.4e-013 Matches: 30
Components: 1. gi|21536288 creatine kinase M-type [Homo sapiens]
2. gi|20664362 Chain A, Polylysine Induces An Antiparallel Actin Dimer That Nucleates Filament Assembly: Crystal Structure At 3.5 A Resolution
Observed Mr(expt) Mr(calc) ppm Start End Miss Comp Peptide
907.4688 906.4615 906.4811 -21.56 308 - 314 0 1 K.FEEILTR.L
914.4267 913.4194 913.4406 -23.20 2 - 9 0 1 M.PFGNTHNK.F
976.4352 975.4279 975.4410 -13.42 15 - 24 0 2 K.AGFAGDDAPR.A
1007.5299 1006.5226 1006.5191 3.51 359 - 366 1 1 K.LMVEMEKK.L
1130.5361 1129.5288 1129.5404 -10.24 193 - 202 0 2 R.GYSFVTTAER.E
1198.6973 1197.6900 1197.6982 -6.85 25 - 35 0 2 R.AVFPSIVGRPR.H
1269.6873 1268.6800 1268.6877 -6.05 305 - 314 1 1 K.HPKFEEILTR.L
1500.7061 1499.6988 1499.7005 -1.09 356 - 368 0 2 K.QEYDEAGPSIVHR.K
1507.7008 1506.6935 1506.6951 -1.01 117 - 130 0 1 K.GGDDLDPNYVLSSR.V
1515.7449 1514.7376 1514.7664 -18.98 136 - 148 1 1 R.SIKGYTLPPHCSR.G
1610.8282 1609.8209 1609.8538 -20.42 253 - 265 1 1 R.FCVGLQKIEEIFK.K
1643.8143 1642.8070 1642.8103 -2.02 224 - 236 0 1 K.SFLVWVNEEDHLR.V
1692.9031 1691.8958 1691.9094 -8.01 157 - 172 1 1 K.LSVEALNSLTGEFKGK.Y
1785.9584 1784.9511 1784.9520 -0.50 342 - 358 0 1 R.LGSSEVEQVQLVVDGVK.L
1790.8910 1789.8837 1789.8846 -0.51 235 - 250 0 2 K.SYELPDGQVITIGNER.F
1956.0355 1955.0282 1955.0364 -4.16 92 - 109 0 2 R.VAPEEHPTLLTEAPLNPK.A
1976.9459 1975.9386 1975.8986 20.3 65 - 80 0 2 K.YPIEHGIITNWDDMEK.I + Oxidation (M)
1994.9438 1993.9365 1993.9342 1.19 321 - 341 0 1 R.GTGGVDTAAVGSVFDVSNADR.L
2374.2097 2373.2024 2373.1093 39.2 287 - 308 1 2 R.KDLYANNVMSGGTTMYPGIADR.M
2390.1384 2389.1311 2389.1042 11.3 287 - 308 1 2 R.KDLYANNVMSGGTTMYPGIADR.M + Oxidation (M)
2573.2319 2572.2246 2572.2016 8.96 12 - 32 1 1 K.LNYKPEEEYPDLSKHNNHMAK.V + Oxidation (M)
2608.2764 2607.2691 2607.2619 2.79 216 - 236 1 1 R.GIWHNDNKSFLVWVNEEDHLR.V
2616.3115 2615.3042 2615.3458 -15.89 333 - 355 0 2 K.YSVWIGGSILASLSTFQQMWITK.Q
2633.3447 2632.3374 2632.2084 49.0 288 - 311 1 2 K.DLYANNVMSGGTTMYPGIADRMQK.E
2942.4229 2941.4156 2941.4116 1.37 267 - 292 0 1 K.AGHPFMWNQHLGYVLTCPSNLGTGLR.G + Oxidation (M)
3196.6343 3195.6270 3195.6023 7.74 144 - 173 0 2 R.TTGIVLDSGDGVTHNVPIYEGYALPHAIMR.L
3212.6292 3211.6219 3211.5972 7.69 144 - 173 0 2 R.TTGIVLDSGDGVTHNVPIYEGYALPHAIMR.L + Oxidation (M)
3644.8499 3643.8426 3643.8378 1.32 178 - 209 1 1 K.SMTEKEQQQLIDDHFLFDKPVSPLLLASGMAR.D
3660.8306 3659.8233 3659.8327 -2.57 178 - 209 1 1 K.SMTEKEQQQLIDDHFLFDKPVSPLLLASGMAR.D + Oxidation (M)
3761.8979 3760.8906 3760.8756 3.99 321 - 358 1 1 R.GTGGVDTAAVGSVFDVSNADRLGSSEVEQVQLVVDGVK.L
No match to: 854.2844, 855.0399, 886.2189, 886.9801, 935.4965, 1157.5375, 1231.6245, 1254.5332, 1359.7104, 1483.6857, 1511.7391, 1671.8419, 1675.8209,
1682.8115, 1738.9460, 1789.8499, 1799.8850, 1813.9745, 1818.9233, 1984.0746, 2055.1343, 2571.3091, 2601.3367, 2612.2659, 2879.5073, 2927.4255,
2943.4182, 2955.4292, 2959.4331, 2971.4209, 3071.5420, 3136.4556, 3153.4805, 3673.8777, 3789.9407
3. Mixture 3 Total score: 185 Expect: 3.4e-013 Matches: 31
Components: 1. gi|351706982 Creatine kinase M-type [Heterocephalus glaber]
2. gi|20664362 Chain A, Polylysine Induces An Antiparallel Actin Dimer That Nucleates Filament Assembly: Crystal Structure At 3.5 A Resolution
Observed Mr(expt) Mr(calc) ppm Start End Miss Comp Peptide
907.4688 906.4615 906.4811 -21.56 310 - 316 0 1 K.FEEILTR.L
914.4267 913.4194 913.4406 -23.20 2 - 9 0 1 M.PFGNTHNK.F
976.4352 975.4279 975.4410 -13.42 15 - 24 0 2 K.AGFAGDDAPR.A
1007.5299 1006.5226 1006.5191 3.51 361 - 368 1 1 K.LMVEMEKK.L
1130.5361 1129.5288 1129.5404 -10.24 193 - 202 0 2 R.GYSFVTTAER.E
1198.6973 1197.6900 1197.6982 -6.85 25 - 35 0 2 R.AVFPSIVGRPR.H
1231.6245 1230.6172 1230.6245 -5.87 87 - 96 0 1 K.DLFDPIIQDR.H
1269.6873 1268.6800 1268.6877 -6.05 307 - 316 1 1 K.HPKFEEILTR.L
1500.7061 1499.6988 1499.7005 -1.09 356 - 368 0 2 K.QEYDEAGPSIVHR.K
1507.7008 1506.6935 1506.6951 -1.01 117 - 130 0 1 K.GGDDLDPNYVLSSR.V
1515.7449 1514.7376 1514.7664 -18.98 136 - 148 1 1 R.SIKGYTLPPHCSR.G
1610.8282 1609.8209 1609.8385 -10.93 180 - 192 1 2 R.DLTDYLMKILTER.G
1643.8143 1642.8070 1642.8103 -2.02 224 - 236 0 1 K.SFLVWVNEEDHLR.V
1692.9031 1691.8958 1691.9094 -8.01 157 - 172 1 1 K.LSVEALNSLTGEFKGK.Y
1785.9584 1784.9511 1784.9520 -0.50 344 - 360 0 1 R.LGSSEVEQVQLVVDGVK.L
1790.8910 1789.8837 1789.8846 -0.51 235 - 250 0 2 K.SYELPDGQVITIGNER.F
1956.0355 1955.0282 1955.0364 -4.16 92 - 109 0 2 R.VAPEEHPTLLTEAPLNPK.A
1976.9459 1975.9386 1975.8986 20.3 65 - 80 0 2 K.YPIEHGIITNWDDMEK.I + Oxidation (M)
2374.2097 2373.2024 2373.1093 39.2 287 - 308 1 2 R.KDLYANNVMSGGTTMYPGIADR.M
2390.1384 2389.1311 2389.1042 11.3 287 - 308 1 2 R.KDLYANNVMSGGTTMYPGIADR.M + Oxidation (M)
2573.2319 2572.2246 2572.2016 8.96 12 - 32 1 1 K.LNYKPEEEYPDLSKHNNHMAK.V + Oxidation (M)

2608.2764 2607.2691 2607.2619 2.79 216 - 236 1 1 R.GIWHNDNKSFLVWVNEEDHLR.V
2616.3115 2615.3042 2615.3458 -15.89 333 - 355 0 2 K.YSVWIGGSILASLSTFQQMWITK.Q
2633.3447 2632.3374 2632.2084 49.0 288 - 311 1 2 K.DLYANNVMSGGTTMYPGIADRMQK.E
2927.4255 2926.4182 2926.4007 5.99 269 - 294 0 1 K.AGHPFMWNEHLGYVLTCPSNLGTGLR.G
2943.4182 2942.4109 2942.3956 5.20 269 - 294 0 1 K.AGHPFMWNEHLGYVLTCPSNLGTGLR.G + Oxidation (M)
3071.5420 3070.5347 3070.4906 14.4 268 - 294 1 1 K.KAGHPFMWNEHLGYVLTCPSNLGTGLR.G + Oxidation (M)
3196.6343 3195.6270 3195.6023 7.74 144 - 173 0 2 R.TTGIVLDSGDGVTHNVPIYEGYALPHAIMR.L
3212.6292 3211.6219 3211.5972 7.69 144 - 173 0 2 R.TTGIVLDSGDGVTHNVPIYEGYALPHAIMR.L + Oxidation (M)
3644.8499 3643.8426 3643.8014 11.3 178 - 209 0 1 K.SMTEQEQQQLIDDHFLFDKPVSPLLLASGMAR.D
3660.8306 3659.8233 3659.7964 7.37 178 - 209 0 1 K.SMTEQEQQQLIDDHFLFDKPVSPLLLASGMAR.D + Oxidation (M)
No match to: 854.2844, 855.0399, 886.2189, 886.9801, 935.4965, 1157.5375, 1254.5332, 1359.7104, 1483.6857, 1511.7391, 1671.8419, 1675.8209, 1682.8115,
1738.9460, 1789.8499, 1799.8850, 1813.9745, 1818.9233, 1984.0746, 1994.9438, 2055.1343, 2571.3091, 2601.3367, 2612.2659, 2879.5073, 2942.4229,
2955.4292, 2959.4331, 2971.4209, 3136.4556, 3153.4805, 3673.8777, 3761.8979, 3789.9407
4. Mixture 4 Total score: 184 Expect: 4.2e-013 Matches: 31
Components: 1. gi|301777736 PREDICTED: creatine kinase M-type-like [Ailuropoda melanoleuca]
2. gi|20664362 Chain A, Polylysine Induces An Antiparallel Actin Dimer That Nucleates Filament Assembly: Crystal Structure At 3.5 A Resolution
Observed Mr(expt) Mr(calc) ppm Start End Miss Comp Peptide
907.4688 906.4615 906.4811 -21.56 308 - 314 0 1 K.FEEILTR.L
914.4267 913.4194 913.4406 -23.20 2 - 9 0 1 M.PFGNTHNK.F
976.4352 975.4279 975.4410 -13.42 15 - 24 0 2 K.AGFAGDDAPR.A
1007.5299 1006.5226 1006.5191 3.51 359 - 366 1 1 K.LMVEMEKK.L
1130.5361 1129.5288 1129.5404 -10.24 193 - 202 0 2 R.GYSFVTTAER.E
1198.6973 1197.6900 1197.6982 -6.85 25 - 35 0 2 R.AVFPSIVGRPR.H
1231.6245 1230.6172 1230.6245 -5.87 87 - 96 0 1 K.DLFDPIIQDR.H
1269.6873 1268.6800 1268.6877 -6.05 305 - 314 1 1 K.HPKFEEILTR.L
1500.7061 1499.6988 1499.7005 -1.09 356 - 368 0 2 K.QEYDEAGPSIVHR.K
1507.7008 1506.6935 1506.6951 -1.01 117 - 130 0 1 K.GGDDLDPNYVISSR.V
1515.7449 1514.7376 1514.7664 -18.98 136 - 148 1 1 R.SIKGYTLPPHCSR.G
1610.8282 1609.8209 1609.8538 -20.42 253 - 265 1 1 R.FCVGLQKIEEIFK.K
1643.8143 1642.8070 1642.8103 -2.02 224 - 236 0 1 K.SFLVWVNEEDHLR.V
1692.9031 1691.8958 1691.9094 -8.01 157 - 172 1 1 K.LSVEALNSLTGEFKGK.Y
1785.9584 1784.9511 1784.9520 -0.50 342 - 358 0 1 R.LGSSEVEQVQLVVDGVK.L
1790.8910 1789.8837 1789.8846 -0.51 235 - 250 0 2 K.SYELPDGQVITIGNER.F
1956.0355 1955.0282 1955.0364 -4.16 92 - 109 0 2 R.VAPEEHPTLLTEAPLNPK.A
1976.9459 1975.9386 1975.8986 20.3 65 - 80 0 2 K.YPIEHGIITNWDDMEK.I + Oxidation (M)
2374.2097 2373.2024 2373.1093 39.2 287 - 308 1 2 R.KDLYANNVMSGGTTMYPGIADR.M
2390.1384 2389.1311 2389.1042 11.3 287 - 308 1 2 R.KDLYANNVMSGGTTMYPGIADR.M + Oxidation (M)
2571.3091 2570.3018 2570.2223 30.9 12 - 32 1 1 K.LNYKPEEEYPDLTKHNNHMAK.A
2608.2764 2607.2691 2607.2619 2.79 216 - 236 1 1 R.GIWHNDNKSFLVWVNEEDHLR.V
2616.3115 2615.3042 2615.3458 -15.89 333 - 355 0 2 K.YSVWIGGSILASLSTFQQMWITK.Q
2633.3447 2632.3374 2632.2084 49.0 288 - 311 1 2 K.DLYANNVMSGGTTMYPGIADRMQK.E
2927.4255 2926.4182 2926.4007 5.99 267 - 292 0 1 K.AGHPFMWNEHLGYVLTCPSNLGTGLR.G
2943.4182 2942.4109 2942.3956 5.20 267 - 292 0 1 K.AGHPFMWNEHLGYVLTCPSNLGTGLR.G + Oxidation (M)
3071.5420 3070.5347 3070.4906 14.4 266 - 292 1 1 K.KAGHPFMWNEHLGYVLTCPSNLGTGLR.G + Oxidation (M)
3196.6343 3195.6270 3195.6023 7.74 144 - 173 0 2 R.TTGIVLDSGDGVTHNVPIYEGYALPHAIMR.L
3212.6292 3211.6219 3211.5972 7.69 144 - 173 0 2 R.TTGIVLDSGDGVTHNVPIYEGYALPHAIMR.L + Oxidation (M)
3644.8499 3643.8426 3643.8378 1.32 178 - 209 1 1 K.SMTEKEQQQLIDDHFLFDKPVSPLLLASGMAR.D
3660.8306 3659.8233 3659.8327 -2.57 178 - 209 1 1 K.SMTEKEQQQLIDDHFLFDKPVSPLLLASGMAR.D + Oxidation (M)
No match to: 854.2844, 855.0399, 886.2189, 886.9801, 935.4965, 1157.5375, 1254.5332, 1359.7104, 1483.6857, 1511.7391, 1671.8419, 1675.8209, 1682.8115,
1738.9460, 1789.8499, 1799.8850, 1813.9745, 1818.9233, 1984.0746, 1994.9438, 2055.1343, 2573.2319, 2601.3367, 2612.2659, 2879.5073, 2942.4229,
2955.4292, 2959.4331, 2971.4209, 3136.4556, 3153.4805, 3673.8777, 3761.8979, 3789.9407
5. Mixture 5 Total score: 181 Expect: 8.4e-013 Matches: 30
Components: 1. gi|149722203 PREDICTED: creatine kinase M-type-like [Equus caballus]
2. gi|20664362 Chain A, Polylysine Induces An Antiparallel Actin Dimer That Nucleates Filament Assembly: Crystal Structure At 3.5 A Resolution
Observed Mr(expt) Mr(calc) ppm Start End Miss Comp Peptide
914.4267 913.4194 913.4406 -23.20 2 - 9 0 1 M.PFGNTHNK.F
976.4352 975.4279 975.4410 -13.42 15 - 24 0 2 K.AGFAGDDAPR.A
1007.5299 1006.5226 1006.5191 3.51 359 - 366 1 1 K.LMVEMEKK.L
1130.5361 1129.5288 1129.5404 -10.24 193 - 202 0 2 R.GYSFVTTAER.E
1198.6973 1197.6900 1197.6982 -6.85 25 - 35 0 2 R.AVFPSIVGRPR.H
1500.7061 1499.6988 1499.7005 -1.09 356 - 368 0 2 K.QEYDEAGPSIVHR.K
1515.7449 1514.7376 1514.7664 -18.98 136 - 148 1 1 R.SIKGYTLPPHCSR.G
1610.8282 1609.8209 1609.8538 -20.42 253 - 265 1 1 R.FCVGLQKIEEIFK.K
1643.8143 1642.8070 1642.8103 -2.02 224 - 236 0 1 K.SFLVWVNEEDHLR.V
1692.9031 1691.8958 1691.9094 -8.01 157 - 172 1 1 K.LSVEALNSLTGEFKGK.Y
1785.9584 1784.9511 1784.9520 -0.50 342 - 358 0 1 R.LGSSEVEQVQLVVDGVK.L
1790.8910 1789.8837 1789.8846 -0.51 235 - 250 0 2 K.SYELPDGQVITIGNER.F
1818.9233 1817.9160 1817.8883 15.3 26 - 40 1 1 K.HNNHMAKALTFDIYK.K + Oxidation (M)
1956.0355 1955.0282 1955.0364 -4.16 92 - 109 0 2 R.VAPEEHPTLLTEAPLNPK.A
1976.9459 1975.9386 1975.8986 20.3 65 - 80 0 2 K.YPIEHGIITNWDDMEK.I + Oxidation (M)
1994.9438 1993.9365 1993.9342 1.19 321 - 341 0 1 R.GTGGVDTAAVGSVFDVSNADR.L
2374.2097 2373.2024 2373.1093 39.2 287 - 308 1 2 R.KDLYANNVMSGGTTMYPGIADR.M
2390.1384 2389.1311 2389.1042 11.3 287 - 308 1 2 R.KDLYANNVMSGGTTMYPGIADR.M + Oxidation (M)
2573.2319 2572.2246 2572.2016 8.96 12 - 32 1 1 K.LNYKPEEEYPDLSKHNNHMAK.A + Oxidation (M)
2608.2764 2607.2691 2607.2619 2.79 216 - 236 1 1 R.GIWHNDNKSFLVWVNEEDHLR.V
2616.3115 2615.3042 2615.3458 -15.89 333 - 355 0 2 K.YSVWIGGSILASLSTFQQMWITK.Q
2633.3447 2632.3374 2632.2084 49.0 288 - 311 1 2 K.DLYANNVMSGGTTMYPGIADRMQK.E
2927.4255 2926.4182 2926.4007 5.99 267 - 292 0 1 K.AGHPFMWNEHLGYVLTCPSNLGTGLR.G
2943.4182 2942.4109 2942.3956 5.20 267 - 292 0 1 K.AGHPFMWNEHLGYVLTCPSNLGTGLR.G + Oxidation (M)
3071.5420 3070.5347 3070.4906 14.4 266 - 292 1 1 K.KAGHPFMWNEHLGYVLTCPSNLGTGLR.G + Oxidation (M)
3196.6343 3195.6270 3195.6023 7.74 144 - 173 0 2 R.TTGIVLDSGDGVTHNVPIYEGYALPHAIMR.L
3212.6292 3211.6219 3211.5972 7.69 144 - 173 0 2 R.TTGIVLDSGDGVTHNVPIYEGYALPHAIMR.L + Oxidation (M)
3644.8499 3643.8426 3643.8014 11.3 178 - 209 0 1 K.SMTEQEQQQLIDDHFLFDKPVSPLLLASGMAR.D
3660.8306 3659.8233 3659.7964 7.37 178 - 209 0 1 K.SMTEQEQQQLIDDHFLFDKPVSPLLLASGMAR.D + Oxidation (M)
3761.8979 3760.8906 3760.8756 3.99 321 - 358 1 1 R.GTGGVDTAAVGSVFDVSNADRLGSSEVEQVQLVVDGVK.L

No match to: 854.2844, 855.0399, 886.2189, 886.9801, 907.4688, 935.4965, 1157.5375, 1231.6245, 1254.5332, 1269.6873, 1359.7104, 1483.6857, 1507.7008,
1511.7391, 1671.8419, 1675.8209, 1682.8115, 1738.9460, 1789.8499, 1799.8850, 1813.9745, 1984.0746, 2055.1343, 2571.3091, 2601.3367, 2612.2659,
2879.5073, 2942.4229, 2955.4292, 2959.4331, 2971.4209, 3136.4556, 3153.4805, 3673.8777, 3789.9407
6. gi|4838363 Mass: 43172 Score: 179 Expect: 1.3e-012 Matches: 20
creatine kinase M chain [Bos taurus]
Observed Mr(expt) Mr(calc) ppm Start End Miss Peptide
907.4688 906.4615 906.4811 -21.56 308 - 314 0 K.FEEILTR.L
914.4267 913.4194 913.4406 -23.20 2 - 9 0 M.PFGNTHNK.H
1007.5299 1006.5226 1006.5191 3.51 359 - 366 1 K.LMVEMEKK.L
1157.5375 1156.5302 1156.5448 -12.56 139 - 148 0 K.GYALPPHCSR.G
1231.6245 1230.6172 1230.6245 -5.87 87 - 96 0 K.DLFDPIIQDR.H
1269.6873 1268.6800 1268.6877 -6.05 305 - 314 1 K.HPKFEEILTR.L
1507.7008 1506.6935 1506.6951 -1.01 117 - 130 0 K.GGDDLDPNYVLSSR.V
1610.8282 1609.8209 1609.8538 -20.42 253 - 265 1 R.FCVGLQKIEEIFK.K
1643.8143 1642.8070 1642.8103 -2.02 224 - 236 0 K.SFLVWVNEEDHLR.V
1682.8115 1681.8042 1681.8199 -9.30 12 - 25 1 K.LNFKAEEEYPDLSK.H
1692.9031 1691.8958 1691.9094 -8.01 157 - 172 1 K.LSVEALNSLTGEFKGK.Y
1785.9584 1784.9511 1784.9520 -0.50 342 - 358 0 R.LGSSEVEQVQLVVDGVK.L
1994.9438 1993.9365 1993.9342 1.19 321 - 341 0 R.GTGGVDTAAVGSVFDVSNADR.L
2608.2764 2607.2691 2607.2619 2.79 216 - 236 1 R.GIWHNDNKSFLVWVNEEDHLR.V
2927.4255 2926.4182 2926.4007 5.99 267 - 292 0 K.AGHPFMWNEHLGYVLTCPSNLGTGLR.G
2943.4182 2942.4109 2942.3956 5.20 267 - 292 0 K.AGHPFMWNEHLGYVLTCPSNLGTGLR.G + Oxidation (M)
3071.5420 3070.5347 3070.4906 14.4 266 - 292 1 K.KAGHPFMWNEHLGYVLTCPSNLGTGLR.G + Oxidation (M)
3644.8499 3643.8426 3643.8014 11.3 178 - 209 0 K.SMTEQEQQQLIDDHFLFDKPVSPLLLASGMAR.D
3660.8306 3659.8233 3659.7964 7.37 178 - 209 0 K.SMTEQEQQQLIDDHFLFDKPVSPLLLASGMAR.D + Oxidation (M)
3761.8979 3760.8906 3760.8756 3.99 321 - 358 1 R.GTGGVDTAAVGSVFDVSNADRLGSSEVEQVQLVVDGVK.L
No match to: 854.2844, 855.0399, 886.2189, 886.9801, 935.4965, 976.4352, 1130.5361, 1198.6973, 1254.5332, 1359.7104, 1483.6857, 1500.7061, 1511.7391,
1515.7449, 1671.8419, 1675.8209, 1738.9460, 1789.8499, 1790.8910, 1799.8850, 1813.9745, 1818.9233, 1956.0355, 1976.9459, 1984.0746, 2055.1343,
2374.2097, 2390.1384, 2571.3091, 2573.2319, 2601.3367, 2612.2659, 2616.3115, 2633.3447, 2879.5073, 2942.4229, 2955.4292, 2959.4331, 2971.4209,
3136.4556, 3153.4805, 3196.6343, 3212.6292, 3673.8777, 3789.9407
7. gi|194018722 Mass: 43260 Score: 178 Expect: 1.7e-012 Matches: 20
creatine kinase M-type [Sus scrofa]
Observed Mr(expt) Mr(calc) ppm Start End Miss Peptide
907.4688 906.4615 906.4811 -21.56 308 - 314 0 K.FEEILTR.L
914.4267 913.4194 913.4406 -23.20 2 - 9 0 M.PFGNTHNK.Y
1007.5299 1006.5226 1006.5191 3.51 359 - 366 1 K.LMVEMEKK.L
1231.6245 1230.6172 1230.6245 -5.87 87 - 96 0 K.DLFDPIIQDR.H
1269.6873 1268.6800 1268.6877 -6.05 305 - 314 1 K.HPKFEEILTR.L
1507.7008 1506.6935 1506.6951 -1.01 117 - 130 0 K.GGDDLDPNYVLSSR.V
1515.7449 1514.7376 1514.7664 -18.98 136 - 148 1 R.SIKGYTLPPHCSR.G
1610.8282 1609.8209 1609.8538 -20.42 253 - 265 1 R.FCVGLQKIEEIFK.K
1643.8143 1642.8070 1642.8103 -2.02 224 - 236 0 K.SFLVWVNEEDHLR.V
1682.8115 1681.8042 1681.8199 -9.30 12 - 25 1 K.LNFKAEEEYPDLSK.H
1692.9031 1691.8958 1691.9094 -8.01 157 - 172 1 K.LSVEALNSLTGEFKGK.Y
1785.9584 1784.9511 1784.9520 -0.50 342 - 358 0 R.LGSSEVEQVQLVVDGVK.L
1994.9438 1993.9365 1993.9342 1.19 321 - 341 0 R.GTGGVDTAAVGSVFDVSNADR.L
2608.2764 2607.2691 2607.2619 2.79 216 - 236 1 R.GIWHNDNKSFLVWVNEEDHLR.V
2927.4255 2926.4182 2926.4007 5.99 267 - 292 0 K.AGHPFMWNEHLGYVLTCPSNLGTGLR.G
2943.4182 2942.4109 2942.3956 5.20 267 - 292 0 K.AGHPFMWNEHLGYVLTCPSNLGTGLR.G + Oxidation (M)
3071.5420 3070.5347 3070.4906 14.4 266 - 292 1 K.KAGHPFMWNEHLGYVLTCPSNLGTGLR.G + Oxidation (M)
3644.8499 3643.8426 3643.8014 11.3 178 - 209 0 K.SMTEQEQQQLIDDHFLFDKPVSPLLLASGMAR.D
3660.8306 3659.8233 3659.7964 7.37 178 - 209 0 K.SMTEQEQQQLIDDHFLFDKPVSPLLLASGMAR.D + Oxidation (M)
3761.8979 3760.8906 3760.8756 3.99 321 - 358 1 R.GTGGVDTAAVGSVFDVSNADRLGSSEVEQVQLVVDGVK.L
No match to: 854.2844, 855.0399, 886.2189, 886.9801, 935.4965, 976.4352, 1130.5361, 1157.5375, 1198.6973, 1254.5332, 1359.7104, 1483.6857, 1500.7061,
1511.7391, 1671.8419, 1675.8209, 1738.9460, 1789.8499, 1790.8910, 1799.8850, 1813.9745, 1818.9233, 1956.0355, 1976.9459, 1984.0746, 2055.1343,
2374.2097, 2390.1384, 2571.3091, 2573.2319, 2601.3367, 2612.2659, 2616.3115, 2633.3447, 2879.5073, 2942.4229, 2955.4292, 2959.4331, 2971.4209,
3136.4556, 3153.4805, 3196.6343, 3212.6292, 3673.8777, 3789.9407
8. gi|60097925 Mass: 43190 Score: 177 Expect: 2.1e-012 Matches: 20
creatine kinase M-type [Bos taurus]
Observed Mr(expt) Mr(calc) ppm Start End Miss Peptide
907.4688 906.4615 906.4811 -21.56 308 - 314 0 K.FEEILTR.L
914.4267 913.4194 913.4406 -23.20 2 - 9 0 M.PFGNTHNK.H
1007.5299 1006.5226 1006.5191 3.51 359 - 366 1 K.LMVEMEKK.L
1157.5375 1156.5302 1156.5448 -12.56 139 - 148 0 K.GYALPPHCSR.G
1231.6245 1230.6172 1230.6245 -5.87 87 - 96 0 K.DLFDPIIQDR.H
1269.6873 1268.6800 1268.6877 -6.05 305 - 314 1 K.HPKFEEILTR.L
1507.7008 1506.6935 1506.6951 -1.01 117 - 130 0 K.GGDDLDPNYVLSSR.V
1610.8282 1609.8209 1609.8538 -20.42 253 - 265 1 R.FCVGLQKIEEIFK.K
1643.8143 1642.8070 1642.8103 -2.02 224 - 236 0 K.SFLVWVNEEDHLR.V
1682.8115 1681.8042 1681.8199 -9.30 12 - 25 1 K.LNFKAEEEYPDLSK.H
1692.9031 1691.8958 1691.9094 -8.01 157 - 172 1 K.LSVEALNSLTGEFKGK.Y
1785.9584 1784.9511 1784.9520 -0.50 342 - 358 0 R.LGSSEVEQVQLVVDGVK.L
1994.9438 1993.9365 1993.9342 1.19 321 - 341 0 R.GTGGVDTAAVGSVFDVSNADR.L
2608.2764 2607.2691 2607.2619 2.79 216 - 236 1 R.GIWHNDNKSFLVWVNEEDHLR.V
2927.4255 2926.4182 2926.4007 5.99 267 - 292 0 K.AGHPFMWNEHLGYVLTCPSNLGTGLR.G
2943.4182 2942.4109 2942.3956 5.20 267 - 292 0 K.AGHPFMWNEHLGYVLTCPSNLGTGLR.G + Oxidation (M)
3071.5420 3070.5347 3070.4906 14.4 266 - 292 1 K.KAGHPFMWNEHLGYVLTCPSNLGTGLR.G + Oxidation (M)
3644.8499 3643.8426 3643.8014 11.3 178 - 209 0 K.SMTEQEQQQLIDDHFLFDKPVSPLLLASGMAR.D
3660.8306 3659.8233 3659.7964 7.37 178 - 209 0 K.SMTEQEQQQLIDDHFLFDKPVSPLLLASGMAR.D + Oxidation (M)
3761.8979 3760.8906 3760.8756 3.99 321 - 358 1 R.GTGGVDTAAVGSVFDVSNADRLGSSEVEQVQLVVDGVK.L
No match to: 854.2844, 855.0399, 886.2189, 886.9801, 935.4965, 976.4352, 1130.5361, 1198.6973, 1254.5332, 1359.7104, 1483.6857, 1500.7061, 1511.7391,
1515.7449, 1671.8419, 1675.8209, 1738.9460, 1789.8499, 1790.8910, 1799.8850, 1813.9745, 1818.9233, 1956.0355, 1976.9459, 1984.0746, 2055.1343,

2374.2097, 2390.1384, 2571.3091, 2573.2319, 2601.3367, 2612.2659, 2616.3115, 2633.3447, 2879.5073, 2942.4229, 2955.4292, 2959.4331, 2971.4209,
3136.4556, 3153.4805, 3196.6343, 3212.6292, 3673.8777, 3789.9407
9. gi|126344225 Mass: 43205 Score: 152 Expect: 6.7e-010 Matches: 18
PREDICTED: creatine kinase M-type-like [Monodelphis domestica]
Observed Mr(expt) Mr(calc) ppm Start End Miss Peptide
907.4688 906.4615 906.4811 -21.56 308 - 314 0 K.FEEILTR.L
914.4267 913.4194 913.4406 -23.20 2 - 9 0 M.PFGNTHNK.Y
1007.5299 1006.5226 1006.5191 3.51 359 - 366 1 K.LMVEMEKK.L
1231.6245 1230.6172 1230.6245 -5.87 87 - 96 0 K.DLFDPIIQDR.H
1269.6873 1268.6800 1268.6877 -6.05 305 - 314 1 K.HPKFEEILTR.L
1507.7008 1506.6935 1506.6951 -1.01 117 - 130 0 K.GGDDLDPNYVLSSR.V
1515.7449 1514.7376 1514.7664 -18.98 136 - 148 1 R.SIKGYTLPPHCSR.G
1610.8282 1609.8209 1609.8538 -20.42 253 - 265 1 R.FCVGLQKIEEIFK.K
1643.8143 1642.8070 1642.8103 -2.02 224 - 236 0 K.SFLVWVNEEDHLR.V
1785.9584 1784.9511 1784.9520 -0.50 342 - 358 0 R.LGSSEVEQVQLVVDGVK.L
1994.9438 1993.9365 1993.9342 1.19 321 - 341 0 R.GTGGVDTAAVGSVFDVSNADR.L
2608.2764 2607.2691 2607.2619 2.79 216 - 236 1 R.GIWHNDNKSFLVWVNEEDHLR.V
2927.4255 2926.4182 2926.4007 5.99 267 - 292 0 K.AGHPFMWNEHLGYVLTCPSNLGTGLR.G
2943.4182 2942.4109 2942.3956 5.20 267 - 292 0 K.AGHPFMWNEHLGYVLTCPSNLGTGLR.G + Oxidation (M)
3071.5420 3070.5347 3070.4906 14.4 266 - 292 1 K.KAGHPFMWNEHLGYVLTCPSNLGTGLR.G + Oxidation (M)
3644.8499 3643.8426 3643.8014 11.3 178 - 209 0 K.SMTEQEQQQLIDDHFLFDKPVSPLLLASGMAR.D
3660.8306 3659.8233 3659.7964 7.37 178 - 209 0 K.SMTEQEQQQLIDDHFLFDKPVSPLLLASGMAR.D + Oxidation (M)
3761.8979 3760.8906 3760.8756 3.99 321 - 358 1 R.GTGGVDTAAVGSVFDVSNADRLGSSEVEQVQLVVDGVK.L
No match to: 854.2844, 855.0399, 886.2189, 886.9801, 935.4965, 976.4352, 1130.5361, 1157.5375, 1198.6973, 1254.5332, 1359.7104, 1483.6857, 1500.7061,
1511.7391, 1671.8419, 1675.8209, 1682.8115, 1692.9031, 1738.9460, 1789.8499, 1790.8910, 1799.8850, 1813.9745, 1818.9233, 1956.0355, 1976.9459,
1984.0746, 2055.1343, 2374.2097, 2390.1384, 2571.3091, 2573.2319, 2601.3367, 2612.2659, 2616.3115, 2633.3447, 2879.5073, 2942.4229, 2955.4292,
2959.4331, 2971.4209, 3136.4556, 3153.4805, 3196.6343, 3212.6292, 3673.8777, 3789.9407
10. gi|21536288 Mass: 43302 Score: 150 Expect: 1.1e-009 Matches: 17
creatine kinase M-type [Homo sapiens]
Observed Mr(expt) Mr(calc) ppm Start End Miss Peptide
907.4688 906.4615 906.4811 -21.56 308 - 314 0 K.FEEILTR.L
914.4267 913.4194 913.4406 -23.20 2 - 9 0 M.PFGNTHNK.F
1007.5299 1006.5226 1006.5191 3.51 359 - 366 1 K.LMVEMEKK.L
1269.6873 1268.6800 1268.6877 -6.05 305 - 314 1 K.HPKFEEILTR.L
1507.7008 1506.6935 1506.6951 -1.01 117 - 130 0 K.GGDDLDPNYVLSSR.V
1515.7449 1514.7376 1514.7664 -18.98 136 - 148 1 R.SIKGYTLPPHCSR.G
1610.8282 1609.8209 1609.8538 -20.42 253 - 265 1 R.FCVGLQKIEEIFK.K
1643.8143 1642.8070 1642.8103 -2.02 224 - 236 0 K.SFLVWVNEEDHLR.V
1692.9031 1691.8958 1691.9094 -8.01 157 - 172 1 K.LSVEALNSLTGEFKGK.Y
1785.9584 1784.9511 1784.9520 -0.50 342 - 358 0 R.LGSSEVEQVQLVVDGVK.L
1994.9438 1993.9365 1993.9342 1.19 321 - 341 0 R.GTGGVDTAAVGSVFDVSNADR.L
2573.2319 2572.2246 2572.2016 8.96 12 - 32 1 K.LNYKPEEEYPDLSKHNNHMAK.V + Oxidation (M)
2608.2764 2607.2691 2607.2619 2.79 216 - 236 1 R.GIWHNDNKSFLVWVNEEDHLR.V
2942.4229 2941.4156 2941.4116 1.37 267 - 292 0 K.AGHPFMWNQHLGYVLTCPSNLGTGLR.G + Oxidation (M)
3644.8499 3643.8426 3643.8378 1.32 178 - 209 1 K.SMTEKEQQQLIDDHFLFDKPVSPLLLASGMAR.D
3660.8306 3659.8233 3659.8327 -2.57 178 - 209 1 K.SMTEKEQQQLIDDHFLFDKPVSPLLLASGMAR.D + Oxidation (M)
3761.8979 3760.8906 3760.8756 3.99 321 - 358 1 R.GTGGVDTAAVGSVFDVSNADRLGSSEVEQVQLVVDGVK.L
No match to: 854.2844, 855.0399, 886.2189, 886.9801, 935.4965, 976.4352, 1130.5361, 1157.5375, 1198.6973, 1231.6245, 1254.5332, 1359.7104, 1483.6857,
1500.7061, 1511.7391, 1671.8419, 1675.8209, 1682.8115, 1738.9460, 1789.8499, 1790.8910, 1799.8850, 1813.9745, 1818.9233, 1956.0355, 1976.9459,
1984.0746, 2055.1343, 2374.2097, 2390.1384, 2571.3091, 2601.3367, 2612.2659, 2616.3115, 2633.3447, 2879.5073, 2927.4255, 2943.4182, 2955.4292,
2959.4331, 2971.4209, 3071.5420, 3136.4556, 3153.4805, 3196.6343, 3212.6292, 3673.8777, 3789.9407
11. gi|355703653 Mass: 43302 Score: 150 Expect: 1.1e-009 Matches: 17
hypothetical protein EGK_10748 [Macaca mulatta]
Observed Mr(expt) Mr(calc) ppm Start End Miss Peptide
907.4688 906.4615 906.4811 -21.56 308 - 314 0 K.FEEILTR.L
914.4267 913.4194 913.4406 -23.20 2 - 9 0 M.PFGNTHNK.F
1007.5299 1006.5226 1006.5191 3.51 359 - 366 1 K.LMVEMEKK.L
1269.6873 1268.6800 1268.6877 -6.05 305 - 314 1 K.HPKFEEILTR.L
1507.7008 1506.6935 1506.6951 -1.01 117 - 130 0 K.GGDDLDPNYVLSSR.V
1515.7449 1514.7376 1514.7664 -18.98 136 - 148 1 R.SIKGYTLPPHCSR.G
1610.8282 1609.8209 1609.8538 -20.42 253 - 265 1 R.FCVGLQKIEEIFK.K
1643.8143 1642.8070 1642.8103 -2.02 224 - 236 0 K.SFLVWVNEEDHLR.V
1692.9031 1691.8958 1691.9094 -8.01 157 - 172 1 K.LSVEALNSLTGEFKGK.Y
1785.9584 1784.9511 1784.9520 -0.50 342 - 358 0 R.LGSSEVEQVQLVVDGVK.L
1994.9438 1993.9365 1993.9342 1.19 321 - 341 0 R.GTGGVDTAAVGSVFDVSNADR.L
2573.2319 2572.2246 2572.2016 8.96 12 - 32 1 K.LNYKPEEEYPDLSKHNNHMAK.V + Oxidation (M)
2608.2764 2607.2691 2607.2619 2.79 216 - 236 1 R.GIWHNDNKSFLVWVNEEDHLR.V
2942.4229 2941.4156 2941.4116 1.37 267 - 292 0 K.AGHPFMWNQHLGYVLTCPSNLGTGLR.G + Oxidation (M)
3644.8499 3643.8426 3643.8378 1.32 178 - 209 1 K.SMTEKEQQQLIDDHFLFDKPVSPLLLASGMAR.D
3660.8306 3659.8233 3659.8327 -2.57 178 - 209 1 K.SMTEKEQQQLIDDHFLFDKPVSPLLLASGMAR.D + Oxidation (M)
3761.8979 3760.8906 3760.8756 3.99 321 - 358 1 R.GTGGVDTAAVGSVFDVSNADRLGSSEVEQVQLVVDGVK.L
No match to: 854.2844, 855.0399, 886.2189, 886.9801, 935.4965, 976.4352, 1130.5361, 1157.5375, 1198.6973, 1231.6245, 1254.5332, 1359.7104, 1483.6857,
1500.7061, 1511.7391, 1671.8419, 1675.8209, 1682.8115, 1738.9460, 1789.8499, 1790.8910, 1799.8850, 1813.9745, 1818.9233, 1956.0355, 1976.9459,
1984.0746, 2055.1343, 2374.2097, 2390.1384, 2571.3091, 2601.3367, 2612.2659, 2616.3115, 2633.3447, 2879.5073, 2927.4255, 2943.4182, 2955.4292,
2959.4331, 2971.4209, 3071.5420, 3136.4556, 3153.4805, 3196.6343, 3212.6292, 3673.8777, 3789.9407
12. gi|149722203 Mass: 43370 Score: 148 Expect: 1.7e-009 Matches: 17
PREDICTED: creatine kinase M-type-like [Equus caballus]
Observed Mr(expt) Mr(calc) ppm Start End Miss Peptide
914.4267 913.4194 913.4406 -23.20 2 - 9 0 M.PFGNTHNK.F
1007.5299 1006.5226 1006.5191 3.51 359 - 366 1 K.LMVEMEKK.L
1515.7449 1514.7376 1514.7664 -18.98 136 - 148 1 R.SIKGYTLPPHCSR.G
1610.8282 1609.8209 1609.8538 -20.42 253 - 265 1 R.FCVGLQKIEEIFK.K

1643.8143 1642.8070 1642.8103 -2.02 224 - 236 0 K.SFLVWVNEEDHLR.V
1692.9031 1691.8958 1691.9094 -8.01 157 - 172 1 K.LSVEALNSLTGEFKGK.Y
1785.9584 1784.9511 1784.9520 -0.50 342 - 358 0 R.LGSSEVEQVQLVVDGVK.L
1818.9233 1817.9160 1817.8883 15.3 26 - 40 1 K.HNNHMAKALTFDIYK.K + Oxidation (M)
1994.9438 1993.9365 1993.9342 1.19 321 - 341 0 R.GTGGVDTAAVGSVFDVSNADR.L
2573.2319 2572.2246 2572.2016 8.96 12 - 32 1 K.LNYKPEEEYPDLSKHNNHMAK.A + Oxidation (M)
2608.2764 2607.2691 2607.2619 2.79 216 - 236 1 R.GIWHNDNKSFLVWVNEEDHLR.V
2927.4255 2926.4182 2926.4007 5.99 267 - 292 0 K.AGHPFMWNEHLGYVLTCPSNLGTGLR.G
2943.4182 2942.4109 2942.3956 5.20 267 - 292 0 K.AGHPFMWNEHLGYVLTCPSNLGTGLR.G + Oxidation (M)
3071.5420 3070.5347 3070.4906 14.4 266 - 292 1 K.KAGHPFMWNEHLGYVLTCPSNLGTGLR.G + Oxidation (M)
3644.8499 3643.8426 3643.8014 11.3 178 - 209 0 K.SMTEQEQQQLIDDHFLFDKPVSPLLLASGMAR.D
3660.8306 3659.8233 3659.7964 7.37 178 - 209 0 K.SMTEQEQQQLIDDHFLFDKPVSPLLLASGMAR.D + Oxidation (M)
3761.8979 3760.8906 3760.8756 3.99 321 - 358 1 R.GTGGVDTAAVGSVFDVSNADRLGSSEVEQVQLVVDGVK.L
No match to: 854.2844, 855.0399, 886.2189, 886.9801, 907.4688, 935.4965, 976.4352, 1130.5361, 1157.5375, 1198.6973, 1231.6245, 1254.5332, 1269.6873,
1359.7104, 1483.6857, 1500.7061, 1507.7008, 1511.7391, 1671.8419, 1675.8209, 1682.8115, 1738.9460, 1789.8499, 1790.8910, 1799.8850, 1813.9745,
1956.0355, 1976.9459, 1984.0746, 2055.1343, 2374.2097, 2390.1384, 2571.3091, 2601.3367, 2612.2659, 2616.3115, 2633.3447, 2879.5073, 2942.4229,
2955.4292, 2959.4331, 2971.4209, 3136.4556, 3153.4805, 3196.6343, 3212.6292, 3673.8777, 3789.9407
13. gi|301777736 Mass: 43353 Score: 144 Expect: 4.2e-009 Matches: 18
PREDICTED: creatine kinase M-type-like [Ailuropoda melanoleuca]
Observed Mr(expt) Mr(calc) ppm Start End Miss Peptide
907.4688 906.4615 906.4811 -21.56 308 - 314 0 K.FEEILTR.L
914.4267 913.4194 913.4406 -23.20 2 - 9 0 M.PFGNTHNK.F
1007.5299 1006.5226 1006.5191 3.51 359 - 366 1 K.LMVEMEKK.L
1231.6245 1230.6172 1230.6245 -5.87 87 - 96 0 K.DLFDPIIQDR.H
1269.6873 1268.6800 1268.6877 -6.05 305 - 314 1 K.HPKFEEILTR.L
1507.7008 1506.6935 1506.6951 -1.01 117 - 130 0 K.GGDDLDPNYVISSR.V
1515.7449 1514.7376 1514.7664 -18.98 136 - 148 1 R.SIKGYTLPPHCSR.G
1610.8282 1609.8209 1609.8538 -20.42 253 - 265 1 R.FCVGLQKIEEIFK.K
1643.8143 1642.8070 1642.8103 -2.02 224 - 236 0 K.SFLVWVNEEDHLR.V
1692.9031 1691.8958 1691.9094 -8.01 157 - 172 1 K.LSVEALNSLTGEFKGK.Y
1785.9584 1784.9511 1784.9520 -0.50 342 - 358 0 R.LGSSEVEQVQLVVDGVK.L
2571.3091 2570.3018 2570.2223 30.9 12 - 32 1 K.LNYKPEEEYPDLTKHNNHMAK.A
2608.2764 2607.2691 2607.2619 2.79 216 - 236 1 R.GIWHNDNKSFLVWVNEEDHLR.V
2927.4255 2926.4182 2926.4007 5.99 267 - 292 0 K.AGHPFMWNEHLGYVLTCPSNLGTGLR.G
2943.4182 2942.4109 2942.3956 5.20 267 - 292 0 K.AGHPFMWNEHLGYVLTCPSNLGTGLR.G + Oxidation (M)
3071.5420 3070.5347 3070.4906 14.4 266 - 292 1 K.KAGHPFMWNEHLGYVLTCPSNLGTGLR.G + Oxidation (M)
3644.8499 3643.8426 3643.8378 1.32 178 - 209 1 K.SMTEKEQQQLIDDHFLFDKPVSPLLLASGMAR.D
3660.8306 3659.8233 3659.8327 -2.57 178 - 209 1 K.SMTEKEQQQLIDDHFLFDKPVSPLLLASGMAR.D + Oxidation (M)
No match to: 854.2844, 855.0399, 886.2189, 886.9801, 935.4965, 976.4352, 1130.5361, 1157.5375, 1198.6973, 1254.5332, 1359.7104, 1483.6857, 1500.7061,
1511.7391, 1671.8419, 1675.8209, 1682.8115, 1738.9460, 1789.8499, 1790.8910, 1799.8850, 1813.9745, 1818.9233, 1956.0355, 1976.9459, 1984.0746,
1994.9438, 2055.1343, 2374.2097, 2390.1384, 2573.2319, 2601.3367, 2612.2659, 2616.3115, 2633.3447, 2879.5073, 2942.4229, 2955.4292, 2959.4331,
2971.4209, 3136.4556, 3153.4805, 3196.6343, 3212.6292, 3673.8777, 3761.8979, 3789.9407
14. gi|297705152 Mass: 43287 Score: 138 Expect: 1.7e-008 Matches: 16
PREDICTED: creatine kinase M-type-like isoform 2 [Pongo abelii]
Observed Mr(expt) Mr(calc) ppm Start End Miss Peptide
907.4688 906.4615 906.4811 -21.56 307 - 313 0 K.FEEILTR.L
914.4267 913.4194 913.4406 -23.20 2 - 9 0 M.PFGNTHNK.F
1007.5299 1006.5226 1006.5191 3.51 358 - 365 1 K.LMVEMEKK.L
1269.6873 1268.6800 1268.6877 -6.05 304 - 313 1 K.HPKFEEILTR.L
1515.7449 1514.7376 1514.7664 -18.98 135 - 147 1 R.SIKGYTLPPHCSR.G
1610.8282 1609.8209 1609.8538 -20.42 252 - 264 1 R.FCVGLQKIEEIFK.K
1643.8143 1642.8070 1642.8103 -2.02 223 - 235 0 K.SFLVWVNEEDHLR.V
1692.9031 1691.8958 1691.9094 -8.01 156 - 171 1 K.LSVEALNSLTGEFKGK.Y
1785.9584 1784.9511 1784.9520 -0.50 341 - 357 0 R.LGSSEVEQVQLVVDGVK.L
1994.9438 1993.9365 1993.9342 1.19 320 - 340 0 R.GTGGVDTAAVGSVFDVSNADR.L
2573.2319 2572.2246 2572.2016 8.96 12 - 32 1 K.LNYKPEEEYPDLSKHNNHMAK.V + Oxidation (M)
2608.2764 2607.2691 2607.2619 2.79 215 - 235 1 R.GIWHNDNKSFLVWVNEEDHLR.V
2942.4229 2941.4156 2941.4116 1.37 266 - 291 0 K.AGHPFMWNQHLGYVLTCPSNLGTGLR.G + Oxidation (M)
3644.8499 3643.8426 3643.8378 1.32 177 - 208 1 K.SMTEKEQQQLIDDHFLFDKPVSPLLLASGMAR.D
3660.8306 3659.8233 3659.8327 -2.57 177 - 208 1 K.SMTEKEQQQLIDDHFLFDKPVSPLLLASGMAR.D + Oxidation (M)
3761.8979 3760.8906 3760.8756 3.99 320 - 357 1 R.GTGGVDTAAVGSVFDVSNADRLGSSEVEQVQLVVDGVK.L
No match to: 854.2844, 855.0399, 886.2189, 886.9801, 935.4965, 976.4352, 1130.5361, 1157.5375, 1198.6973, 1231.6245, 1254.5332, 1359.7104, 1483.6857,
1500.7061, 1507.7008, 1511.7391, 1671.8419, 1675.8209, 1682.8115, 1738.9460, 1789.8499, 1790.8910, 1799.8850, 1813.9745, 1818.9233, 1956.0355,
1976.9459, 1984.0746, 2055.1343, 2374.2097, 2390.1384, 2571.3091, 2601.3367, 2612.2659, 2616.3115, 2633.3447, 2879.5073, 2927.4255, 2943.4182,
2955.4292, 2959.4331, 2971.4209, 3071.5420, 3136.4556, 3153.4805, 3196.6343, 3212.6292, 3673.8777, 3789.9407
15. gi|189053833 Mass: 43230 Score: 135 Expect: 3.4e-008 Matches: 16
unnamed protein product [Homo sapiens]
Observed Mr(expt) Mr(calc) ppm Start End Miss Peptide
907.4688 906.4615 906.4811 -21.56 308 - 314 0 K.FEEILTR.L
914.4267 913.4194 913.4406 -23.20 2 - 9 0 M.PFGNTHNK.F
1007.5299 1006.5226 1006.5191 3.51 359 - 366 1 K.LMVEMEKK.L
1269.6873 1268.6800 1268.6877 -6.05 305 - 314 1 K.HPKFEEILTR.L
1507.7008 1506.6935 1506.6951 -1.01 117 - 130 0 K.GGDDLDPNYVLSSR.V
1515.7449 1514.7376 1514.7664 -18.98 136 - 148 1 R.SIKGYTLPPHCSR.G
1610.8282 1609.8209 1609.8538 -20.42 253 - 265 1 R.FCVGLQKIEEIFK.K
1643.8143 1642.8070 1642.8103 -2.02 224 - 236 0 K.SFLVWVNEEDHLR.V
1692.9031 1691.8958 1691.9094 -8.01 157 - 172 1 K.LSVEALNSLTGEFKGK.Y
1785.9584 1784.9511 1784.9520 -0.50 342 - 358 0 R.LGSSEVEQVQLVVDGVK.L
1994.9438 1993.9365 1993.9342 1.19 321 - 341 0 R.GTGGVDTAAVGSVFDVSNADR.L
2608.2764 2607.2691 2607.2619 2.79 216 - 236 1 R.GIWHNDNKSFLVWVNEEDHLR.V
2942.4229 2941.4156 2941.4116 1.37 267 - 292 0 K.AGHPFMWNQHLGYVLTCPSNLGTGLR.G + Oxidation (M)
3644.8499 3643.8426 3643.8378 1.32 178 - 209 1 K.SMTEKEQQQLIDDHFLFDKPVSPLLLASGMAR.D
3660.8306 3659.8233 3659.8327 -2.57 178 - 209 1 K.SMTEKEQQQLIDDHFLFDKPVSPLLLASGMAR.D + Oxidation (M)

3761.8979 3760.8906 3760.8756 3.99 321 - 358 1 R.GTGGVDTAAVGSVFDVSNADRLGSSEVEQVQLVVDGVK.L
No match to: 854.2844, 855.0399, 886.2189, 886.9801, 935.4965, 976.4352, 1130.5361, 1157.5375, 1198.6973, 1231.6245, 1254.5332, 1359.7104, 1483.6857,
1500.7061, 1511.7391, 1671.8419, 1675.8209, 1682.8115, 1738.9460, 1789.8499, 1790.8910, 1799.8850, 1813.9745, 1818.9233, 1956.0355, 1976.9459,
1984.0746, 2055.1343, 2374.2097, 2390.1384, 2571.3091, 2573.2319, 2601.3367, 2612.2659, 2616.3115, 2633.3447, 2879.5073, 2927.4255, 2943.4182,
2955.4292, 2959.4331, 2971.4209, 3071.5420, 3136.4556, 3153.4805, 3196.6343, 3212.6292, 3673.8777, 3789.9407
16. Mixture 6 Total score: 134 Expect: 4.2e-008 Matches: 25
Components: 1. gi|20664362 Chain A, Polylysine Induces An Antiparallel Actin Dimer That Nucleates Filament Assembly: Crystal Structure At 3.5 A Resolution
2. gi|344269321 PREDICTED: creatine kinase M-type-like [Loxodonta africana]
Observed Mr(expt) Mr(calc) ppm Start End Miss Comp Peptide
914.4267 913.4194 913.4406 -23.20 2 - 9 0 2 M.PFGNTHNK.F
976.4352 975.4279 975.4410 -13.42 15 - 24 0 1 K.AGFAGDDAPR.A
1130.5361 1129.5288 1129.5404 -10.24 193 - 202 0 1 R.GYSFVTTAER.E
1198.6973 1197.6900 1197.6982 -6.85 25 - 35 0 1 R.AVFPSIVGRPR.H
1231.6245 1230.6172 1230.6245 -5.87 87 - 96 0 2 K.DLFDPIIQDR.H
1500.7061 1499.6988 1499.7005 -1.09 356 - 368 0 1 K.QEYDEAGPSIVHR.K
1507.7008 1506.6935 1506.6951 -1.01 117 - 130 0 2 K.GGDDLDPNYVISSR.V
1515.7449 1514.7376 1514.7419 -2.80 81 - 91 0 1 K.IWHHTFYNELR.V
1610.8282 1609.8209 1609.8385 -10.93 180 - 192 1 1 R.DLTDYLMKILTER.G
1692.9031 1691.8958 1691.9094 -8.01 157 - 172 1 2 K.LSVEALNSLTGEFKGK.Y
1785.9584 1784.9511 1784.9520 -0.50 245 - 261 0 2 R.LGSSEVEQVQLVVDGVK.L
1790.8910 1789.8837 1789.8846 -0.51 235 - 250 0 1 K.SYELPDGQVITIGNER.F
1818.9233 1817.9160 1817.8883 15.3 26 - 40 1 2 K.HNNHMAKALTFDIYK.K + Oxidation (M)
1956.0355 1955.0282 1955.0364 -4.16 92 - 109 0 1 R.VAPEEHPTLLTEAPLNPK.A
1976.9459 1975.9386 1975.8986 20.3 65 - 80 0 1 K.YPIEHGIITNWDDMEK.I + Oxidation (M)
1994.9438 1993.9365 1993.9705 -17.05 225 - 244 1 2 R.KSGVDTAAVGSVFDVSNADR.L
2374.2097 2373.2024 2373.1093 39.2 287 - 308 1 1 R.KDLYANNVMSGGTTMYPGIADR.M
2390.1384 2389.1311 2389.1042 11.3 287 - 308 1 1 R.KDLYANNVMSGGTTMYPGIADR.M + Oxidation (M)
2571.3091 2570.3018 2570.2223 30.9 12 - 32 1 2 K.LNYKPEEEYPDLTKHNNHMAK.A
2616.3115 2615.3042 2615.3458 -15.89 333 - 355 0 1 K.YSVWIGGSILASLSTFQQMWITK.Q
2633.3447 2632.3374 2632.2084 49.0 288 - 311 1 1 K.DLYANNVMSGGTTMYPGIADRMQK.E
3196.6343 3195.6270 3195.6023 7.74 144 - 173 0 1 R.TTGIVLDSGDGVTHNVPIYEGYALPHAIMR.L
3212.6292 3211.6219 3211.5972 7.69 144 - 173 0 1 R.TTGIVLDSGDGVTHNVPIYEGYALPHAIMR.L + Oxidation (M)
3644.8499 3643.8426 3643.8014 11.3 178 - 209 0 2 K.SMTEQEQQQLIDDHFLFDKPVSPLLLASGMAR.D
3660.8306 3659.8233 3659.7964 7.37 178 - 209 0 2 K.SMTEQEQQQLIDDHFLFDKPVSPLLLASGMAR.D + Oxidation (M)
No match to: 854.2844, 855.0399, 886.2189, 886.9801, 907.4688, 935.4965, 1007.5299, 1157.5375, 1254.5332, 1269.6873, 1359.7104, 1483.6857, 1511.7391,
1643.8143, 1671.8419, 1675.8209, 1682.8115, 1738.9460, 1789.8499, 1799.8850, 1813.9745, 1984.0746, 2055.1343, 2573.2319, 2601.3367, 2608.2764,
2612.2659, 2879.5073, 2927.4255, 2942.4229, 2943.4182, 2955.4292, 2959.4331, 2971.4209, 3071.5420, 3136.4556, 3153.4805, 3673.8777, 3761.8979, 3789.9407
17. gi|119577741 Mass: 45971 Score: 134 Expect: 4.2e-008 Matches: 16
creatine kinase, muscle [Homo sapiens]
Observed Mr(expt) Mr(calc) ppm Start End Miss Peptide
907.4688 906.4615 906.4811 -21.56 333 - 339 0 K.FEEILTR.L
1007.5299 1006.5226 1006.5191 3.51 384 - 391 1 K.LMVEMEKK.L
1269.6873 1268.6800 1268.6877 -6.05 330 - 339 1 K.HPKFEEILTR.L
1507.7008 1506.6935 1506.6951 -1.01 142 - 155 0 K.GGDDLDPNYVLSSR.V
1515.7449 1514.7376 1514.7664 -18.98 161 - 173 1 R.SIKGYTLPPHCSR.G
1610.8282 1609.8209 1609.8538 -20.42 278 - 290 1 R.FCVGLQKIEEIFK.K
1643.8143 1642.8070 1642.8103 -2.02 249 - 261 0 K.SFLVWVNEEDHLR.V
1692.9031 1691.8958 1691.9094 -8.01 182 - 197 1 K.LSVEALNSLTGEFKGK.Y
1785.9584 1784.9511 1784.9520 -0.50 367 - 383 0 R.LGSSEVEQVQLVVDGVK.L
1994.9438 1993.9365 1993.9342 1.19 346 - 366 0 R.GTGGVDTAAVGSVFDVSNADR.L
2573.2319 2572.2246 2572.2016 8.96 37 - 57 1 K.LNYKPEEEYPDLSKHNNHMAK.V + Oxidation (M)
2608.2764 2607.2691 2607.2619 2.79 241 - 261 1 R.GIWHNDNKSFLVWVNEEDHLR.V
2942.4229 2941.4156 2941.4116 1.37 292 - 317 0 K.AGHPFMWNQHLGYVLTCPSNLGTGLR.G + Oxidation (M)
3644.8499 3643.8426 3643.8378 1.32 203 - 234 1 K.SMTEKEQQQLIDDHFLFDKPVSPLLLASGMAR.D
3660.8306 3659.8233 3659.8327 -2.57 203 - 234 1 K.SMTEKEQQQLIDDHFLFDKPVSPLLLASGMAR.D + Oxidation (M)
3761.8979 3760.8906 3760.8756 3.99 346 - 383 1 R.GTGGVDTAAVGSVFDVSNADRLGSSEVEQVQLVVDGVK.L
No match to: 854.2844, 855.0399, 886.2189, 886.9801, 914.4267, 935.4965, 976.4352, 1130.5361, 1157.5375, 1198.6973, 1231.6245, 1254.5332, 1359.7104,
1483.6857, 1500.7061, 1511.7391, 1671.8419, 1675.8209, 1682.8115, 1738.9460, 1789.8499, 1790.8910, 1799.8850, 1813.9745, 1818.9233, 1956.0355,
1976.9459, 1984.0746, 2055.1343, 2374.2097, 2390.1384, 2571.3091, 2601.3367, 2612.2659, 2616.3115, 2633.3447, 2879.5073, 2927.4255, 2943.4182,
2955.4292, 2959.4331, 2971.4209, 3071.5420, 3136.4556, 3153.4805, 3196.6343, 3212.6292, 3673.8777, 3789.9407
18. gi|351706982 Mass: 43449 Score: 132 Expect: 6.7e-008 Matches: 17
Creatine kinase M-type [Heterocephalus glaber]
Observed Mr(expt) Mr(calc) ppm Start End Miss Peptide
907.4688 906.4615 906.4811 -21.56 310 - 316 0 K.FEEILTR.L
914.4267 913.4194 913.4406 -23.20 2 - 9 0 M.PFGNTHNK.F
1007.5299 1006.5226 1006.5191 3.51 361 - 368 1 K.LMVEMEKK.L
1231.6245 1230.6172 1230.6245 -5.87 87 - 96 0 K.DLFDPIIQDR.H
1269.6873 1268.6800 1268.6877 -6.05 307 - 316 1 K.HPKFEEILTR.L
1507.7008 1506.6935 1506.6951 -1.01 117 - 130 0 K.GGDDLDPNYVLSSR.V
1515.7449 1514.7376 1514.7664 -18.98 136 - 148 1 R.SIKGYTLPPHCSR.G
1643.8143 1642.8070 1642.8103 -2.02 224 - 236 0 K.SFLVWVNEEDHLR.V
1692.9031 1691.8958 1691.9094 -8.01 157 - 172 1 K.LSVEALNSLTGEFKGK.Y
1785.9584 1784.9511 1784.9520 -0.50 344 - 360 0 R.LGSSEVEQVQLVVDGVK.L
2573.2319 2572.2246 2572.2016 8.96 12 - 32 1 K.LNYKPEEEYPDLSKHNNHMAK.V + Oxidation (M)
2608.2764 2607.2691 2607.2619 2.79 216 - 236 1 R.GIWHNDNKSFLVWVNEEDHLR.V
2927.4255 2926.4182 2926.4007 5.99 269 - 294 0 K.AGHPFMWNEHLGYVLTCPSNLGTGLR.G
2943.4182 2942.4109 2942.3956 5.20 269 - 294 0 K.AGHPFMWNEHLGYVLTCPSNLGTGLR.G + Oxidation (M)
3071.5420 3070.5347 3070.4906 14.4 268 - 294 1 K.KAGHPFMWNEHLGYVLTCPSNLGTGLR.G + Oxidation (M)
3644.8499 3643.8426 3643.8014 11.3 178 - 209 0 K.SMTEQEQQQLIDDHFLFDKPVSPLLLASGMAR.D
3660.8306 3659.8233 3659.7964 7.37 178 - 209 0 K.SMTEQEQQQLIDDHFLFDKPVSPLLLASGMAR.D + Oxidation (M)
No match to: 854.2844, 855.0399, 886.2189, 886.9801, 935.4965, 976.4352, 1130.5361, 1157.5375, 1198.6973, 1254.5332, 1359.7104, 1483.6857, 1500.7061,

1511.7391, 1610.8282, 1671.8419, 1675.8209, 1682.8115, 1738.9460, 1789.8499, 1790.8910, 1799.8850, 1813.9745, 1818.9233, 1956.0355, 1976.9459,
1984.0746, 1994.9438, 2055.1343, 2374.2097, 2390.1384, 2571.3091, 2601.3367, 2612.2659, 2616.3115, 2633.3447, 2879.5073, 2942.4229, 2955.4292,
2959.4331, 2971.4209, 3136.4556, 3153.4805, 3196.6343, 3212.6292, 3673.8777, 3761.8979, 3789.9407
19. gi|6671762 Mass: 43246 Score: 130 Expect: 1.1e-007 Matches: 17
creatine kinase M-type [Mus musculus]
Observed Mr(expt) Mr(calc) ppm Start End Miss Peptide
907.4688 906.4615 906.4811 -21.56 308 - 314 0 K.FEEILTR.L
914.4267 913.4194 913.4406 -23.20 2 - 9 0 M.PFGNTHNK.F
1007.5299 1006.5226 1006.5191 3.51 359 - 366 1 K.LMVEMEKK.L
1231.6245 1230.6172 1230.6245 -5.87 87 - 96 0 K.DLFDPIIQDR.H
1269.6873 1268.6800 1268.6877 -6.05 305 - 314 1 K.HPKFEEILTR.L
1507.7008 1506.6935 1506.6951 -1.01 117 - 130 0 K.GGDDLDPNYVLSSR.V
1515.7449 1514.7376 1514.7664 -18.98 136 - 148 1 R.SIKGYTLPPHCSR.G
1610.8282 1609.8209 1609.8538 -20.42 253 - 265 1 R.FCVGLQKIEEIFK.K
1643.8143 1642.8070 1642.8103 -2.02 224 - 236 0 K.SFLVWVNEEDHLR.V
1692.9031 1691.8958 1691.9094 -8.01 157 - 172 1 K.LSVEALNSLTGEFKGK.Y
1785.9584 1784.9511 1784.9520 -0.50 342 - 358 0 R.LGSSEVEQVQLVVDGVK.L
2608.2764 2607.2691 2607.2619 2.79 216 - 236 1 R.GIWHNDNKSFLVWVNEEDHLR.V
2927.4255 2926.4182 2926.4007 5.99 267 - 292 0 K.AGHPFMWNEHLGYVLTCPSNLGTGLR.G
2943.4182 2942.4109 2942.3956 5.20 267 - 292 0 K.AGHPFMWNEHLGYVLTCPSNLGTGLR.G + Oxidation (M)
3071.5420 3070.5347 3070.4906 14.4 266 - 292 1 K.KAGHPFMWNEHLGYVLTCPSNLGTGLR.G + Oxidation (M)
3644.8499 3643.8426 3643.8014 11.3 178 - 209 0 K.SMTEQEQQQLIDDHFLFDKPVSPLLLASGMAR.D
3660.8306 3659.8233 3659.7964 7.37 178 - 209 0 K.SMTEQEQQQLIDDHFLFDKPVSPLLLASGMAR.D + Oxidation (M)
No match to: 854.2844, 855.0399, 886.2189, 886.9801, 935.4965, 976.4352, 1130.5361, 1157.5375, 1198.6973, 1254.5332, 1359.7104, 1483.6857, 1500.7061,
1511.7391, 1671.8419, 1675.8209, 1682.8115, 1738.9460, 1789.8499, 1790.8910, 1799.8850, 1813.9745, 1818.9233, 1956.0355, 1976.9459, 1984.0746,
1994.9438, 2055.1343, 2374.2097, 2390.1384, 2571.3091, 2573.2319, 2601.3367, 2612.2659, 2616.3115, 2633.3447, 2879.5073, 2942.4229, 2955.4292,
2959.4331, 2971.4209, 3136.4556, 3153.4805, 3196.6343, 3212.6292, 3673.8777, 3761.8979, 3789.9407
20. gi|6978661 Mass: 43220 Score: 129 Expect: 1.3e-007 Matches: 17
creatine kinase M-type [Rattus norvegicus]
Observed Mr(expt) Mr(calc) ppm Start End Miss Peptide
907.4688 906.4615 906.4811 -21.56 308 - 314 0 K.FEEILTR.L
914.4267 913.4194 913.4406 -23.20 2 - 9 0 M.PFGNTHNK.F
1007.5299 1006.5226 1006.5191 3.51 359 - 366 1 K.LMVEMEKK.L
1231.6245 1230.6172 1230.6245 -5.87 87 - 96 0 K.DLFDPIIQDR.H
1269.6873 1268.6800 1268.6877 -6.05 305 - 314 1 K.HPKFEEILTR.L
1507.7008 1506.6935 1506.6951 -1.01 117 - 130 0 K.GGDDLDPNYVLSSR.V
1515.7449 1514.7376 1514.7664 -18.98 136 - 148 1 R.SIKGYTLPPHCSR.G
1610.8282 1609.8209 1609.8538 -20.42 253 - 265 1 R.FCVGLQKIEEIFK.K
1643.8143 1642.8070 1642.8103 -2.02 224 - 236 0 K.SFLVWVNEEDHLR.V
1692.9031 1691.8958 1691.9094 -8.01 157 - 172 1 K.LSVEALNSLTGEFKGK.Y
1785.9584 1784.9511 1784.9520 -0.50 342 - 358 0 R.LGSSEVEQVQLVVDGVK.L
2608.2764 2607.2691 2607.2619 2.79 216 - 236 1 R.GIWHNDNKSFLVWVNEEDHLR.V
2927.4255 2926.4182 2926.4007 5.99 267 - 292 0 K.AGHPFMWNEHLGYVLTCPSNLGTGLR.G
2943.4182 2942.4109 2942.3956 5.20 267 - 292 0 K.AGHPFMWNEHLGYVLTCPSNLGTGLR.G + Oxidation (M)
3071.5420 3070.5347 3070.4906 14.4 266 - 292 1 K.KAGHPFMWNEHLGYVLTCPSNLGTGLR.G + Oxidation (M)
3644.8499 3643.8426 3643.8014 11.3 178 - 209 0 K.SMTEQEQQQLIDDHFLFDKPVSPLLLASGMAR.D
3660.8306 3659.8233 3659.7964 7.37 178 - 209 0 K.SMTEQEQQQLIDDHFLFDKPVSPLLLASGMAR.D + Oxidation (M)
No match to: 854.2844, 855.0399, 886.2189, 886.9801, 935.4965, 976.4352, 1130.5361, 1157.5375, 1198.6973, 1254.5332, 1359.7104, 1483.6857, 1500.7061,
1511.7391, 1671.8419, 1675.8209, 1682.8115, 1738.9460, 1789.8499, 1790.8910, 1799.8850, 1813.9745, 1818.9233, 1956.0355, 1976.9459, 1984.0746,
1994.9438, 2055.1343, 2374.2097, 2390.1384, 2571.3091, 2573.2319, 2601.3367, 2612.2659, 2616.3115, 2633.3447, 2879.5073, 2942.4229, 2955.4292,
2959.4331, 2971.4209, 3136.4556, 3153.4805, 3196.6343, 3212.6292, 3673.8777, 3761.8979, 3789.9407

Search Parameters

Type of search : Peptide Mass Fingerprint
Enzyme : Trypsin
Fixed modifications : Carbamidomethyl (C)
Variable modifications : Oxidation (M)
Mass values : Monoisotopic
Protein Mass : Unrestricted
Peptide Mass Tolerance : ± 50 ppm
Peptide Charge State : 1+
Max Missed Cleavages : 1
Number of queries : 65

Mascot: http://www.matrixscience.com/

COVERAGE BAND 9-1

Mascot Search Results

Protein View

Match to: gi|4838363 Score: 179 Expect: 1.3e-012
creatine kinase M chain [Bos taurus]
Nominal mass (Mr): 43172; Calculated pI value: 6.63
NCBI BLAST search of gi|4838363 against nr
Unformatted sequence string for pasting into other applications
Taxonomy: Bos taurus
Fixed modifications: Carbamidomethyl (C)

Variable modifications: Oxidation (M)
Cleavage by Trypsin: cuts C-term side of KR unless next residue is P
Number of mass values searched: 65
Number of mass values matched: 20
Sequence Coverage: 58%
Matched peptides shown in **Bold Red**
1 MPFGNTHNKH KLNFKAEEEY PDLSKHNNHM AKALTLEIYK KLRDKETPSG
51 FTLDDVIQTG VDNPGHPFIM TVGCVAGDEE SYTVFKDLFD PIIQDRHGGF
101 KPTDKHKTDL NHENLKGGDD LDPNYVLSSR VRTGRSIKGY ALPPHCSRGE
151 RRAVEKLSVE ALNSLTGEFK GKYYPLKSMT EQEQQQLIDD HFLFDKPVSP
201 LLLASGMARD WPDARGIWHN DNKSFLVWVN EEDHLRVISM EKGGNMKEVF
251 RRFCVGLQKI EEIFKKAGHP FMWNEHLGYV LTCPSNLGTG LRGGVHVKLA
301 HLSKHPKFEE ILTRLRLQKR GTGGVDTAAV GSVFDVSNAD RLGSSEVEQV
351 QLVVDGVKLM VEMEKKLEKG QSIDDLIPAQ K
Show predicted peptides also
Sort Peptides By Residue Number Increasing Mass Decreasing Mass
Start - End Observed Mr(expt) Mr(calc) ppm Miss Sequence
2 - 9 914.4267 913.4194 913.4406 -23 0 M.PFGNTHNK.H
12 - 25 1682.8115 1681.8042 1681.8199 -9 1 K.LNFKAEEEYPDLSK.H
87 - 96 1231.6245 1230.6172 1230.6245 -6 0 K.DLFDPIIQDR.H
117 - 130 1507.7008 1506.6935 1506.6951 -1 0 K.GGDDLDPNYVLSSR.V
139 - 148 1157.5375 1156.5302 1156.5448 -13 0 K.GYALPPHCSR.G
157 - 172 1692.9031 1691.8958 1691.9094 -8 1 K.LSVEALNSLTGEFKGK.Y
178 - 209 3644.8499 3643.8426 3643.8014 11 0 K.SMTEQEQQQLIDDHFLFDKPVSPLLLASGMAR.D
178 - 209 3660.8306 3659.8233 3659.7964 7 0 K.SMTEQEQQQLIDDHFLFDKPVSPLLLASGMAR.D Oxidation (M)
216 - 236 2608.2764 2607.2691 2607.2619 3 1 R.GIWHNDNKSFLVWVNEEDHLR.V
224 - 236 1643.8143 1642.8070 1642.8103 -2 0 K.SFLVWVNEEDHLR.V
253 - 265 1610.8282 1609.8209 1609.8538 -20 1 R.FCVGLQKIEEIFK.K
266 - 292 3071.5420 3070.5347 3070.4906 14 1 K.KAGHPFMWNEHLGYVLTCPSNLGTGLR.G Oxidation (M)
267 - 292 2927.4255 2926.4182 2926.4007 6 0 K.AGHPFMWNEHLGYVLTCPSNLGTGLR.G
267 - 292 2943.4182 2942.4109 2942.3956 5 0 K.AGHPFMWNEHLGYVLTCPSNLGTGLR.G Oxidation (M)
305 - 314 1269.6873 1268.6800 1268.6877 -6 1 K.HPKFEEILTR.L
308 - 314 907.4688 906.4615 906.4811 -22 0 K.FEEILTR.L
321 - 341 1994.9438 1993.9365 1993.9342 1 0 R.GTGGVDTAAVGSVFDVSNADR.L
321 - 358 3761.8979 3760.8906 3760.8756 4 1 R.GTGGVDTAAVGSVFDVSNADRLGSSEVEQVQLVVDGVK.L
342 - 358 1785.9584 1784.9511 1784.9520 -0 0 R.LGSSEVEQVQLVVDGVK.L
359 - 366 1007.5299 1006.5226 1006.5191 4 1 K.LMVEMEKK.L
No match to: 854.2844, 855.0399, 886.2189, 886.9801, 935.4965, 976.4352, 1130.5361, 1198.6973, 1254.5332, 1359.7104, 1483.6857,

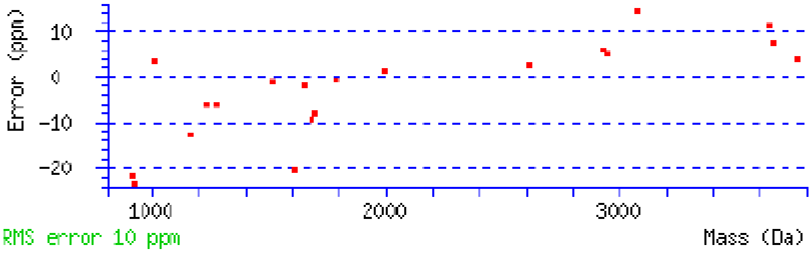

COVERAGE BAND 9-2

Mascot Search Results

Protein View

Match to: gi|20664362 Score: 114 Expect: 4.2e-006
Chain A, Polylysine Induces An Antiparallel Actin Dimer That Nucleates Filament Assembly: Crystal Structure At 3.5 A Resolution
Nominal mass (Mr): 41558; Calculated pI value: 5.46
NCBI BLAST search of gi|20664362 against nr
Unformatted sequence string for pasting into other applications
Taxonomy: Oryctolagus cuniculus
Links to retrieve other entries containing this sequence from NCBI Entrez:
gi|20664363 from Oryctolagus cuniculus
Fixed modifications: Carbamidomethyl (C)
Variable modifications: Oxidation (M)
Cleavage by Trypsin: cuts C-term side of KR unless next residue is P
Number of mass values searched: 65
Number of mass values matched: 15
Sequence Coverage: 52%
Matched peptides shown in **Bold Red**
1 TTALVCDNGS GLVKAGFAGD DAPRAVFPSI VGRPRHQGVM VVMGQGDSYV
51 GDEAQSKRGI LTLKYPIEHG IITNWDDMEK IWHHTFYNEL RVAPEEHPTL
101 LTEAPLNPKA NREKMTQIMF ETFNVPAMYV AIQAVLSLYA SGRTTGIVLD
151 SGDGVTHNVP IYEGYALPHA IMRLDLAGRD LTDYLMKILT ERGYSFVTTA
201 EREIVRDIKE KLCYVALDFE NEMATAASSS SLEKSYELPD GQVITIGNER

251 FRCPETLFQP SFIGMESAGI HETTYNSIMK CDIDIRKDLY ANNVMSGGTT
301 MYPGIADRMQ KEITALAPST MKIKIIAPPE RKYSVWIGGS ILASLSTFQQ
351 MWITKQEYDE AGPSIVHRKC F
Show predicted peptides also
Sort Peptides By Residue Number Increasing Mass Decreasing Mass

Start	-	End	Observed	Mr(expt)	Mr(calc)	ppm	Miss	Sequence
15	-	24	976.4352	975.4279	975.4410	-13	0	K.AGFAGDDAPR.A
25	-	35	1198.6973	1197.6900	1197.6982	-7	0	R.AVFPSIVGRPR.H
65	-	80	1976.9459	1975.9386	1975.8986	20	0	K.YPIEHGIITNWDDMEK.I Oxidation (M)
81	-	91	1515.7449	1514.7376	1514.7419	-3	0	K.IWHHTFYNELR.V
92	-	109	1956.0355	1955.0282	1955.0364	-4	0	R.VAPEEHPTLLTEAPLNPK.A
144	-	173	3196.6343	3195.6270	3195.6023	8	0	R.TTGIVLDSGDGVTHNVPIYEGYALPHAIMR.L
144	-	173	3212.6292	3211.6219	3211.5972	8	0	R.TTGIVLDSGDGVTHNVPIYEGYALPHAIMR.L Oxidation (M)
180	-	192	1610.8282	1609.8209	1609.8385	-11	1	R.DLTDYLMKILTER.G
193	-	202	1130.5361	1129.5288	1129.5404	-10	0	R.GYSFVTTAER.E
235	-	250	1790.8910	1789.8837	1789.8846	-1	0	K.SYELPDGQVITIGNER.F
287	-	308	2374.2097	2373.2024	2373.1093	39	1	R.KDLYANNVMSGGTTMYPGIADR.M
287	-	308	2390.1384	2389.1311	2389.1042	11	1	R.KDLYANNVMSGGTTMYPGIADR.M Oxidation (M)
288	-	311	2633.3447	2632.3374	2632.2084	49	1	K.DLYANNVMSGGTTMYPGIADRMQK.E
333	-	355	2616.3115	2615.3042	2615.3458	-16	0	K.YSVWIGGSILASLSTFQQMWITK.Q
356	-	368	1500.7061	1499.6988	1499.7005	-1	0	K.QEYDEAGPSIVHR.K

No match to: 854.2844, 855.0399, 886.2189, 886.9801, 907.4688, 914.4267, 935.4965, 1007.5299, 1157.5375, 1231.6245, 1254.5332, 1

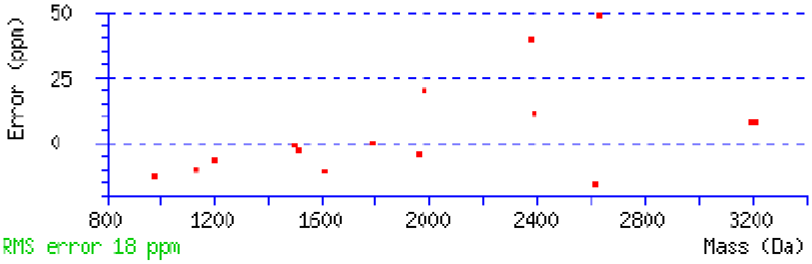

Supplement: Supplementary file 1 [file foods-09-00170-s001.zip › Supplementary Material 3.pdf]
